# Asymmetric C–H Functionalization of Bicyclo[2.1.1]hexanes and Their 2‑Oxa- and 2‑Aza Derivatives via Rhodium Carbene Intermediates

**DOI:** 10.1021/jacs.5c19070

**Published:** 2026-01-05

**Authors:** Ziyi Chen, Duc Ly, Yuzuru Kanda, Vadym V. Levterov, Yaroslav Panasiuk, Pavel K. Mykhailiuk, Djamaladdin G. Musaev, Huw M. L. Davies

**Affiliations:** † Department of Chemistry, 1371 Emory University, Atlanta, Georgia 30322, United States; ‡ Global Discovery Chemistry, Novartis Biomedical Research, 250 Massachusetts Avenue, Cambridge, Massachusetts 02139, United States; § 376198 Enamine Ltd., Winston Churchill Street 78, 02094 Kyiv, Ukraine; ∥ Taras Shevchenko National University of Kyiv, Volodymyrska Street 60, 01601 Kyiv, Ukraine; ⊥ Cherry L. Emerson Center for Scientific Computation, Emory University, 1521 Dickey Drive, Atlanta, Georgia 30322, United States

## Abstract

Bicyclo­[2.1.1]­hexanes have generated considerable interest in recent years as bioisosteres of benzene. In this article, a C–H functionalization approach is described to derivatize the bicyclo[2.1.1]­hexanes. The approach relies on dirhodium-catalyzed C–H insertion by donor/acceptor carbenes, which proceeds in a highly diastereoselective and enantioselective manner. By the appropriate choice of substrates, the reaction can also be highly site-selective. The bicyclo[2.1.1]­hexane is a difficult system for C–H functionalization via carbene intermediates because it is a strained molecule, which causes the C–H bonds to be stronger than in an unstrained system. The only catalyst that performed well in this transformation is the newly developed D_4_ symmetric catalyst, Rh_2_(*S*-megaBNP)_4_, which contains four (4,4′-dichloro-6,6′-di­(3,5-di-*tert*-butyl)­phenyl)­binaphthyl phosphate ligands. Computational studies revealed that the donor–acceptor carbene binds in a defined cleft within the bowl-shape of the dirhodium catalyst. Due to the high symmetry of the catalyst, only two orientations of the carbene are possible, and the most stable one has an open face for attack by the substrate. The substrate also needs to approach through a defined cleft causing the reaction to proceed with high levels of diastereoselectivity and enantioselectivity. These studies represent a further example of how the dirhodium catalysts can display many of the characteristics typically associated with enzymes with well-defined secondary interactions between the wall of the catalyst and the approaching substrate controlling the stereochemical outcome.

## Graphical abstract

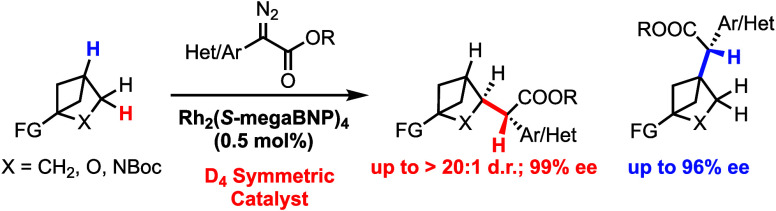

## Introduction

Bicyclic compounds have become of considerable interest in the pharmaceutical industry as bioisosteres for substituted benzene rings, commonly found in many drug candidates.^1–5^ These bicyclic scaffolds have shown improved solubility, metabolic stability, and other physiochemical properties to assist with the development of new therapeutic agents.^6–13^ Bicyclo[1.1.1]­pentanes have been extensively explored as bioisosteres for 1,4-disubstituted arenes (Scheme 1a).^5,8,14–18^ More recently, substituted bicyclo[2.1.1]­hexanes have been examined as bioisosteres for 1,2- or 1,3-disubstituted arenes or heteroarenes.^2,5,6,9,13,19–21^ Consequently, a variety of drug candidates or analogues have been developed, as illustrated in the examples of heteroatom-substituted bicyclo[2.1.1]­hexanes **1a**–**d** shown in Scheme 1b.^1–4^

**Scheme 1:**
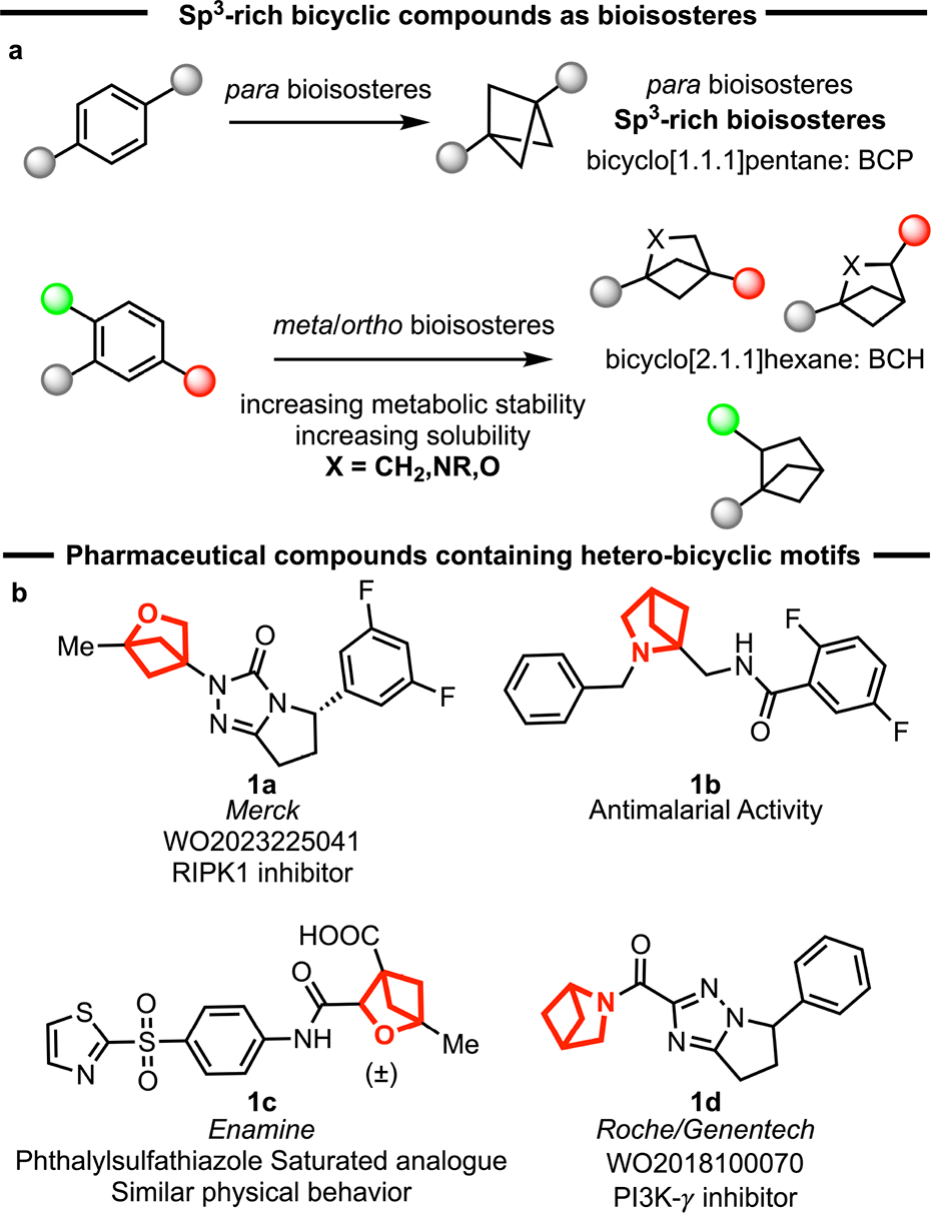
Bicyclic Motifs as Bioisosteres for Benzene

Due to the increasing significance of bicyclo[2.1.1]­hexane derivatives in drug discovery programs, there has been considerable recent interest in developing new methods to access this ring system.^5,22–25^ Some of the most significant methods are strain-release [2π + 2σ] cycloaddition with bicyclo[1.1.0]­butanes,^20,21,26–38^ photochemical [2 + 2] cycloaddition,^9,19,39,40^ and iodine mediated cyclization.^2,6^ Recently, it has been demonstrated that monosubstituted bicyclo[2.1.1]­hexanes and their 2-aza and 2-oxo analogs can be readily accessed by intramolecular [2 + 2] photocycloaddition^40^ or iodocyclization^2^ (Scheme 2a). With ready availability of the monosubstituted substrates, an attractive approach for further diversification would be a catalyst-controlled post C–H functionalization of these bicyclic compounds.

**Scheme 2:**
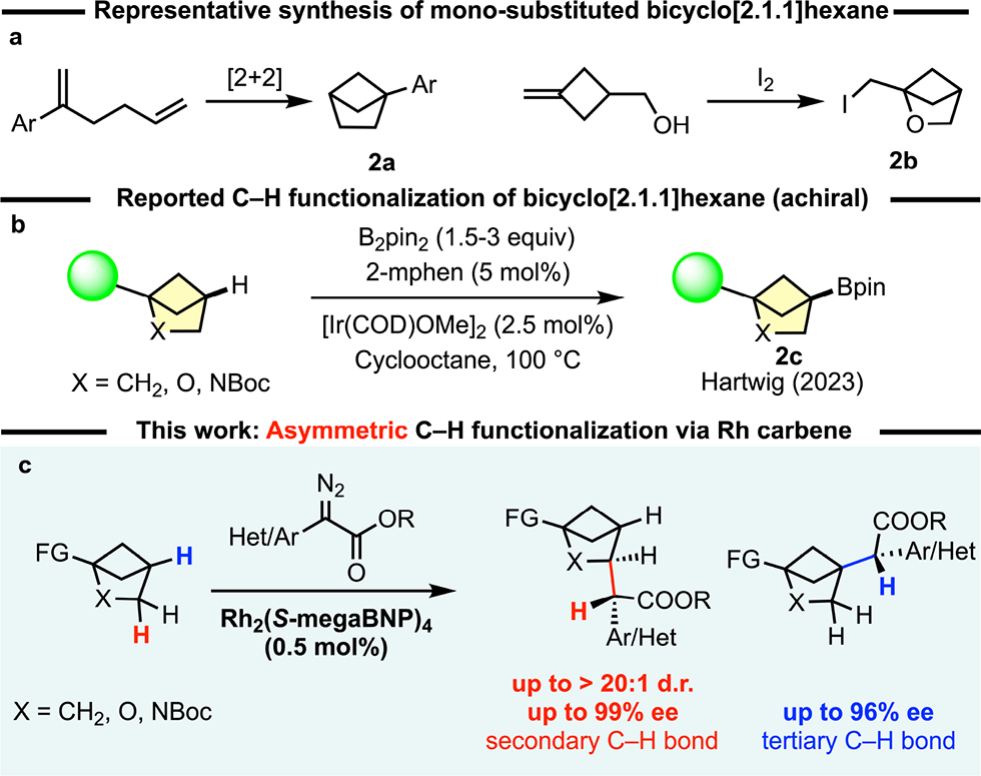
Preparation of Mono-substituted BCH and C–H Functionalization of BCH

In order to achieve this, it is necessary to develop methods that distinguish between the C–H bonds in these substrates. The Hartwig group demonstrated that iridium catalyzed borylation proceeds cleanly at the tertiary C–H bond of bicyclo[2.1.1]­hexanes and the resulting borylated products are amenable for further diversification (Scheme 2b).^41^ The products from the borylation are achiral; thus, an asymmetric C–H functionalization of bicyclo[2.1.1]­hexanes derivatives has yet to be achieved. The Davies group has developed a range of chiral catalysts to achieve site selective C–H functionalization by means of carbene-induced C–H functionalization,^42–44^ and this has been applied to various building blocks of pharmaceutical relevance.^45,46^ In this article, we report the enantioselective and diastereoselective C–H functionalization of bicyclo[2.1.1]­hexane (BCH) and its oxa- and aza-derivatives (Scheme 2c). The bicyclic hydrocarbons are challenging because the C–H bonds are unactivated and a successful outcome requires control of site-selectivity, diastereoselectivity, and enantioselectivity. The heteroatom functionalized bicyclic compounds have activated sites adjacent to the heteroatom suitable for C–H functionalization; thus, the site selectivity is not as challenging, but the diastereoselectivity and enantioselectivity still need to be controlled.

## Results and Discussion

### C–H Functionalization of Bicyclo[2.1.1]­hexanes

The Davies group demonstrated that chiral dirhodium tetracarboxylates are very effective at enantioselective catalyst-controlled C–H functionalization with rhodium-stabilized donor/acceptor carbenes.^42,44^ In addition to selective reactions at activated sites, such as *alpha* to oxygen or nitrogen, they are capable of selective C–H functionalization of acyclic hydrocarbons and various cycloalkane derivatives. Recently, we also reported that the binaphthyl phosphate catalyst, Rh_2_(*S*-megaBNP)_4_, is capable of selective C–H functionalization of unactivated tertiary C–H bonds and outperforms the dirhodium tetracarboxylate catalysts in this reaction.^47^ With a collection of chiral catalysts in hand, the current study began with a test reaction, the C–H functionalization of 1-(4-bromophenyl)­bicyclo[2.1.1]­hexane (**4a**, 2 equiv) with *p*-bromophenyldiazoacetate **3a** as the carbene precursor and a catalyst loading of 1 mol % (Table 1).

**Table 1:**
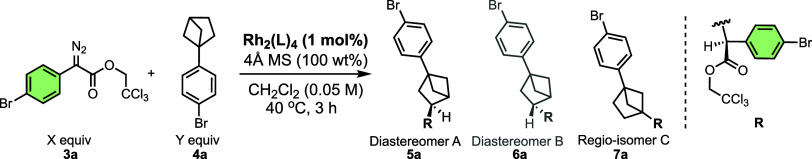
Catalyst Screening for C–H Functionalization of 1-Aryl Bicyclo[2.1.1]­hexane^a^

Entry	Catalysts	*X*:*Y*	Combined Yield^b^ (%)	r.r. (**5a** + **6a**:**7a**)	d.r. (**5a**:**6a**)	ee (%)
1	Rh_2_(*S*-DOSP)_4_	1:2	5			
2	Rh_2_(*S*-p-PhTPCP)_4_	1:2	5			
3	Rh_2_(*S*-TCPTAD)_4_	1:2	5			
4	Rh_2_(*S*-TPPTTL)_4_	1:2	5			
5	Rh_2_(*S*-NTTL)_4_	1:2	<5			
6	Rh_2_(*S*-2Cl5BrTPCP)_4_	1:2	30	15:1	3:1	–80 (*ent*-**5a**)
7	Rh_2_(*S*-BNP)_4_	1:2	5			
8^c^	Rh_2_(*S*-Ph_4_-BNP)_4_	1:2	5			
9^c^	Rh_2_(*S*-megaBNP)_4_	1:2	43(40)^d^	3:1	>20:1	99(**5a**), 93(**7a**)
10^c^	Rh_2_(*S*-megaBNP)_4_	1:1	47	2:1	>20:1	99(**5a**), 93(**7a**)
11^c^	Rh_2_(*S*-megaBNP)_4_	1.5:1	78	2:1	>20:1	99(**5a**), 93(**7a**)
12^c^	Rh_2_(*S*-megaBNP)_4_	**2:1**	**92(89** **)** ^d^	2:1	**>20:1**	**99(5a), 93(7a)**

a 0.1 mmol of substrate and 1 mol % catalyst were dissolved with 1 mL of CH_2_Cl_2_ in a 4 mL vial. 0.2 mmol of diazo was dissolved in 1 mL of CH_2_Cl_2_, and the mixture was added to the reaction vial via a syringe pump over 3 h at 40 °C (0.05 M). d.r. and NMR yields were obtained from crude ^1^H NMR with trimethoxybenzene as internal standard.

b NMR yield using 1,3,5-trimethoxybenzene as internal standard.

c 0.5 mol % catalyst was used.

d Isolation yield.

We recognized that the all-carbon bicyclic system **4a** might be difficult to functionalize because the C–H bonds are unactivated and sterically crowded. Disappointedly, a vast majority of our premier dirhodium tetracarboxylate catalysts (Scheme 3) failed to give any of the desired products (Table 1, entries 1–5). Instead, carbene dimerization was the dominant reaction pathway. These catalysts range from being sterically accessible to sterically crowded; thus, the lack of reactivity is presumably because the C–H bonds in **4a** are simply not sufficiently reactive, and carbene dimerization becomes the alternative outcome.

**Scheme 3:**
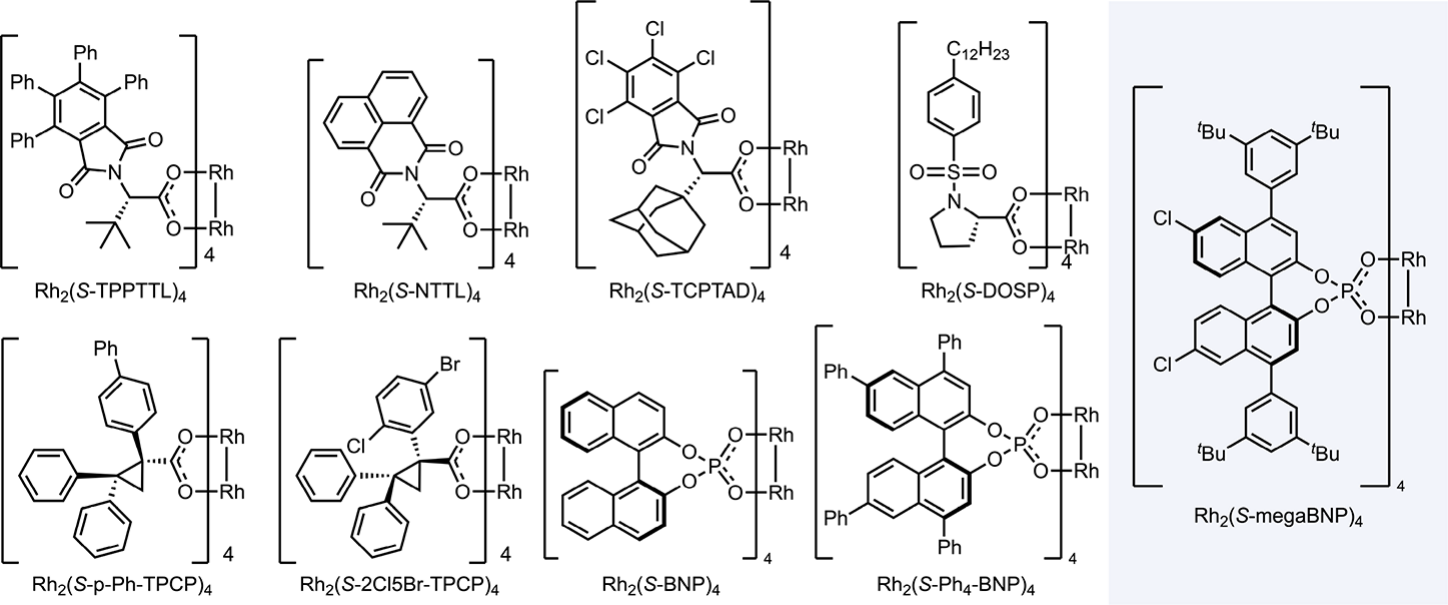
Catalysts Used in the Optimization Studies

Only one of our dirhodium tetracarboxylates, Rh_2_(*S*-2Cl-5Br-TPCP)_4_, gave a significant amount of the C–H functionalization products (entry 6, 30% NMR yield). The major product **5a** is derived from C–H functionalization at the distal methylene site and was formed with site-selectivity of 16:1 r.r. over the tertiary C–H insertion product **7a**. There was no evidence of any C–H functionalization occurring at the other three methylene sites in **4a**, presumably because the C2-methylene site adjacent to the phenyl is too sterically crowded and the two methylene sites in the four-membered ring are less reactive. The major product *ent*-**5a** is obtained with relatively low diastereoselectivity (3:1 d.r.) but reasonably high enantioselectivity (80% ee, enantiomer of **5a**). Rh_2_(*S*-2Cl-5Br-TPCP)_4_ is considered to be a sterically demanding catalyst, adopting a rigid, C_4_-symmetric bowl shape,^48^ which would help explain why the C–H functionalization preferentially occurs at the secondary site over the tertiary site. The reason the carbene is more effective at C–H functionalization but less prone to dimerization using this catalyst compared to the other dirhodium tetracarboxylate catalysts is less clear, but it is likely that the catalyst bowl shape influences how substrates can approach the carbene.

Considering the difficulties we observed in achieving an effective reaction with the dirhodium tetracarboxylate catalysts, we decided to apply our new binaphthyl phosphate catalyst, Rh_2_(*S*-megaBNP)_4_, because it is considered to generate more electrophilic carbene intermediates than the dirhodium tetracarboxylate catalysts.^47^ First, we examined the parent binaphthylphosphonate catalyst Rh_2_(*S*-BNP)_4_ and the tetraphenyl derivative Rh_2_(*S*-Ph_4_-BNP)_4_, but they both performed poorly in the reaction, resulting in only traces of the C–H functionalization products (entries 7 and 8). In contrast, when Rh_2_(*S*-megaBNP)_4_ was used as a catalyst, a great improvement in the efficiency of the desired reaction was observed and the C–H functionalization products were obtained in 43% NMR yield (entry 9). Rh_2_(*S*-megaBNP)_4_ is not as sterically demanding as Rh_2_(*S*-2Cl-5Br-TPCP)_4_, and consequently, the site selectivity for secondary **5a** over tertiary **7a** was less pronounced (3:1 r.r). The stereoselectivity, however, was greatly improved as **5a** was formed in >20:1 d.r. and in 99% ee. The minor tertiary product **7a** was also formed with a high enantioselectivity (93% ee). As there was still evidence of the carbene dimer being formed, the reaction was then examined with bicyclohexane **4a** as the limiting agent, and under these conditions, the yield was greatly improved (92% NMR yield, 89% isolated yield), whereas the stereoselectivity remained about the same (entry 12). The relative stereochemistry of **5a** was determined by ^1^H NMR in which the chemical shift of the bridgehead proton was similar to the starting material **4a**, whereas diastereomer **6a** showed strong shielding of the bridgehead proton (over 0.5 ppm upfield shift).^49^ These studies showed that Rh_2_(*S*-megaBNP)_4_ is uniquely suited for this reaction, capable of high yielding reactions and the formation of the secondary C–H functionalization product **5a** with exceptionally high levels of enantioselectivity and diastereoselectivity.

Since Rh_2_(*S*-megaBNP)_4_ was identified as the optimum catalyst, the scope of the C–H functionalization was examined with a range of aryldiazoacetates (**3a**–**h**) and arylbicyclohexanes (**4a**–**d**) (Scheme 4). In the benchmarking studies when Rh_2_(*S*-megaBNP)_4_ was originally disclosed, it was found that a *para*-substituent on the aryl ring was advantageous for high asymmetric induction,^47^ and so, *para*-substituted aryldiazoacetates were used in this study. A test reaction with the unsubstituted phenyldiazoacetate was also examined, but the C–H functionalization with this carbene source proceeded in low yield (∼20%; see Supporting Information, pages S58–S62). In general, the reactions followed the same trend observed in the optimization studies, forming about a 2:1 mixture of secondary (**5a**–**l**) and tertiary (**7a**–**l**) C–H functionalization products but with exceptionally high levels of enantioselectivity for both products and high levels of diastereoselectivity for the secondary C–H functionalization product (Scheme 4). Aryldiazoacetates with trihaloethyl ester were used throughout because functionalization of unactivated C–H bonds is far more effective with these electron deficient esters.^44^ Indeed, a test reaction with the methyl ester of **4a** gave no evidence of a C–H functionalization product (see, Supporting Information, pages S58–S62). Among the *para*-substituted aryldiazoacetates, the regioselectivity ranged from 1:1 to 2:1 r.r. and the diastereoselectivity for the secondary product varied from 9:1 to >20:1 d.r. The enantioselectivity was exceptionally high for the secondary products **5a**–**l** (98–99% ee) and still very acceptable for tertiary products **7a**–**l** (81–96% ee). A range of aryldiazoacetates (**3a**–**h**) can be used, and the reaction is compatible with halo, trifluoromethyl, and ester functionalities on the aryldiazoacetate, and even 2-chloropyridyl is tolerated. *Para*- and *meta*-substituted arylbicyclohexanes (**4a**–**d**) were also evaluated (4-*tert*-butyl, trifluoromethyl, and 3,4-dimethoxy), and they all afforded the C–H functionalization products with excellent diastereoselectivity and enantioselectivity (>20:1 d.r. and 90–99% ee), although with moderate regioselectivity (1.2–2.4:1). The 6-bromonaphthalen-2-yl substrate gave moderate diastereoselectivity (10:1 d.r. for **5l**) but high enantioselectivity (99% ee for secondary **5l** and 94% ee for tertiary **7l**). The absolute configuration of compound **5l** was confirmed by X-ray crystallography. The absolute configuration of the other products is assigned by analogy.

**Scheme 4:**
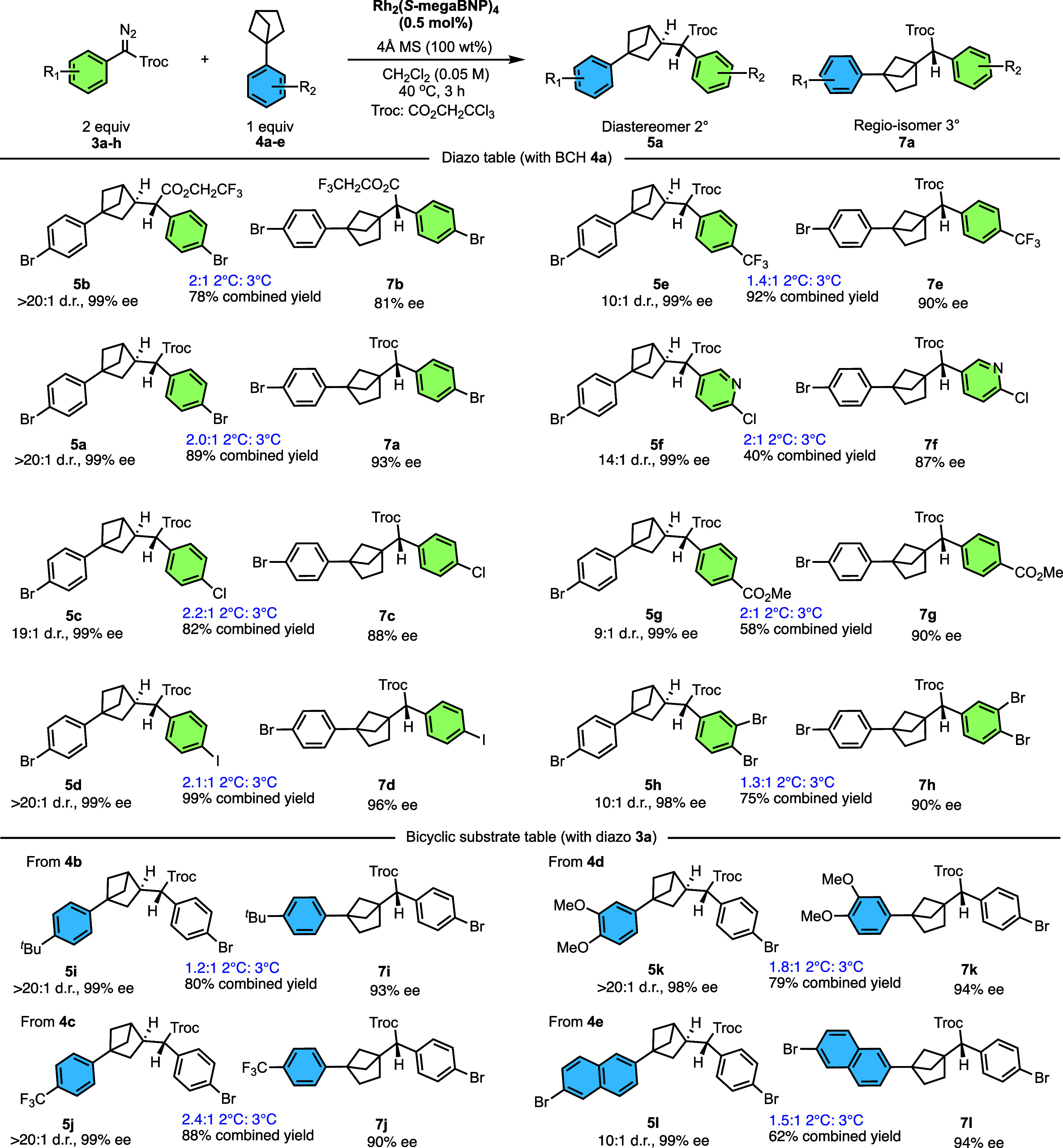
Scope of the C–H Functionalization of BCH

Only Rh_2_(*S*-megaBNP)_4_ is truly effective at achieving high levels of asymmetric induction in the binaphthyl phosphate series of catalysts. Consequently, alternative catalysts are not currently available to modify the overall site selectivity of the reaction as is typically the case in C–H functionalization reactions with dirhodium tetracarboxylates.^44^ Therefore, we decided to explore whether the use of *ortho*-substituted aryldiazoacetates would result in more steric encumbrance and drive the reaction toward C–H functionalization of secondary sites. The reaction with 2-chlorophenyldiazoacetate **3i** resulted in enhanced secondary C–H functionalization (5:1 r.r.) but with a decrease in the diastereoselectivity (4:1 d.r) and enantioselectivity (90% ee, Scheme 5). The absolute configuration of **8a** was also confirmed by X-ray crystallography. As *para*-substitution tends to enhance the enantioselectivity with Rh_2_(*S*-megaBNP)_4_-catalyzed reactions, 2-chloro-4-bromo diazo **3j** was also examined, and a much better result was obtained. The secondary product **8b** was formed with improved site selectivity (11:1 r.r.), diastereoselectivity (12:1 d.r.), and enantioselectivity (98% ee). These results demonstrate that site selectivity can be controlled by increasing the steric features of the carbene, and this is a useful alternative strategy to catalyst control.

**Scheme 5:**
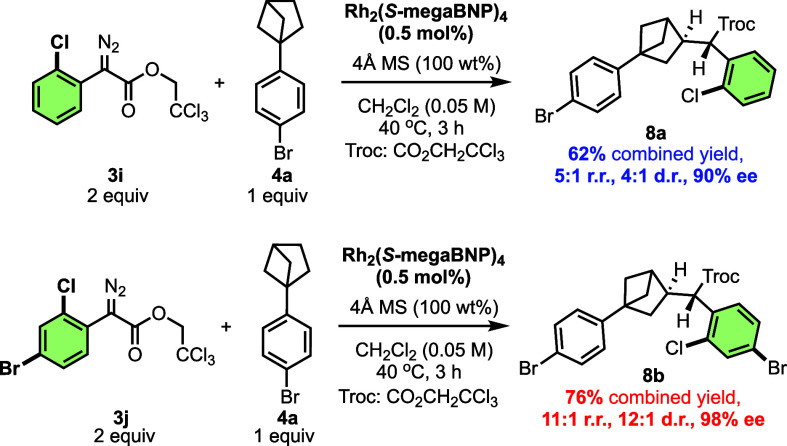
Enhancement of Site-Selectivity

### C–H Functionalization of 2-Oxa- and 2-Azabicyclo[2.1.1]­hexanes

We also investigated the C–H functionalization of 2-oxa and 2-azabicyclo[2.1.1]­hexanes because this type of scaffold has been incorporated in potential drug candidates.^1,3,12,39^ The evaluation of the same series of catalysts was conducted using the 1-substituted 2-oxabicyclo[2.1.1]­hexane **10a** as the test substrate (Table 2). Due to the activating influence of the heteroatom, the reaction preferentially occurs at C-3 and all of the dirhodium tetracarboxylate catalysts are capable of achieving C–H functionalization (Table 2, entries 1–6). However, with most of the carboxylate catalysts, the yield of **11a** is still low, and the diastereoselectivity is close to 1:1. The only carboxylate catalyst that gives a significant improvement is Rh_2_(*S*-TPPTTL)_4_, which gave a 59% yield of **11a** with moderate diastereocontrol (4:1 d.r.) and enantiocontrol (79% ee) (entry 6). The parent binaphthylphosphonate catalyst Rh_2_(*S*-BNP)_4_ also gave poor results (entry 7). Once again, Rh_2_(*S*-megaBNP)_4_ gave exceptional results, generating **11a** in >20:1 d.r. and 90% ee (entry 8). Ironically, in the Rh_2_(*S*-megaBNP)_4_ catalyzed reaction, the yield for the formation of the oxa product **11a** (43%) is lower than what was obtained with the carbocyclic products **5a** and **7a** (92%). This follows the general trend observed in the benchmarking studies that the carbene derived from a Rh_2_(*S*-megaBNP)_4_-catalyzed reaction appears more electrophilic than the dirhodium tetracarboxylate carbene and is less tolerant to nucleophilic functional groups.^47^

**Table 2:**
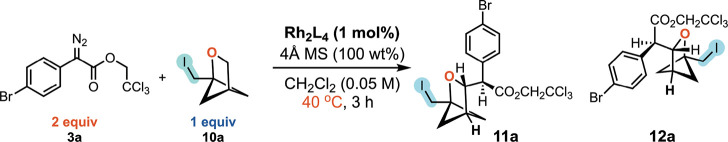
Catalyst Screening for C–H Functionalization of 1-Iodo-2-oxabicyclo[2.1.1]­hexane

Entry	Catalysts	NMR Yield of **11a** (%)	d.r. (**11a**:**12a**)	ee (%)
1	Rh_2_(*S*-DOSP)_4_	20	1:1	
2	Rh_2_(*S*-PTAD)_4_	23	1:1	
3	Rh_2_(*S*-TCPTAD)_4_	11	1:1	
4	Rh_2_(*S*-2Cl5BrTPCP)_4_	15	1:2	
5	Rh_2_(*S*-NTTL)_4_	14	1:1	
6	Rh_2_(*S*-TPPTTL)_4_	59	4:1	–79
7	Rh_2_(*S*-BNP)_4_	24	3:1	
8	**Rh**_**2**_ **(** ***S*** **-megaBNP)**_**4**_	**43(40** **)**	**>20:1**	**90**

The Rh_2_(*S*-megaBNP)_4_-catalyzed reaction can be applied to a variety of 1-substituted 2-oxabicyclohexane derivatives **10b**–**f** as illustrated in Scheme 6. Halogen, ester, ether, and N-phthalimido groups were well tolerated, generating **11b**–**f** with high diastereoselectivity (10:1 to >20:1 d.r.) and enantioselectivity (85%–92% ee). The disubstituted 2-oxabicyclohexane **10g** was also examined, and it gave the desired product **11g** with high diastereoselectivity (>20:1 d.r.) and enantioselectivity (84% ee). In general, however, the reaction with a 1,4-disubstituted 2-oxabicyclohexane is more challenging, and if the second substituent is sterically demanding or electron withdrawing, low yields of C–H functionalization products are obtained (see SI for details on unsuccessful reactions).

**Scheme 6:**
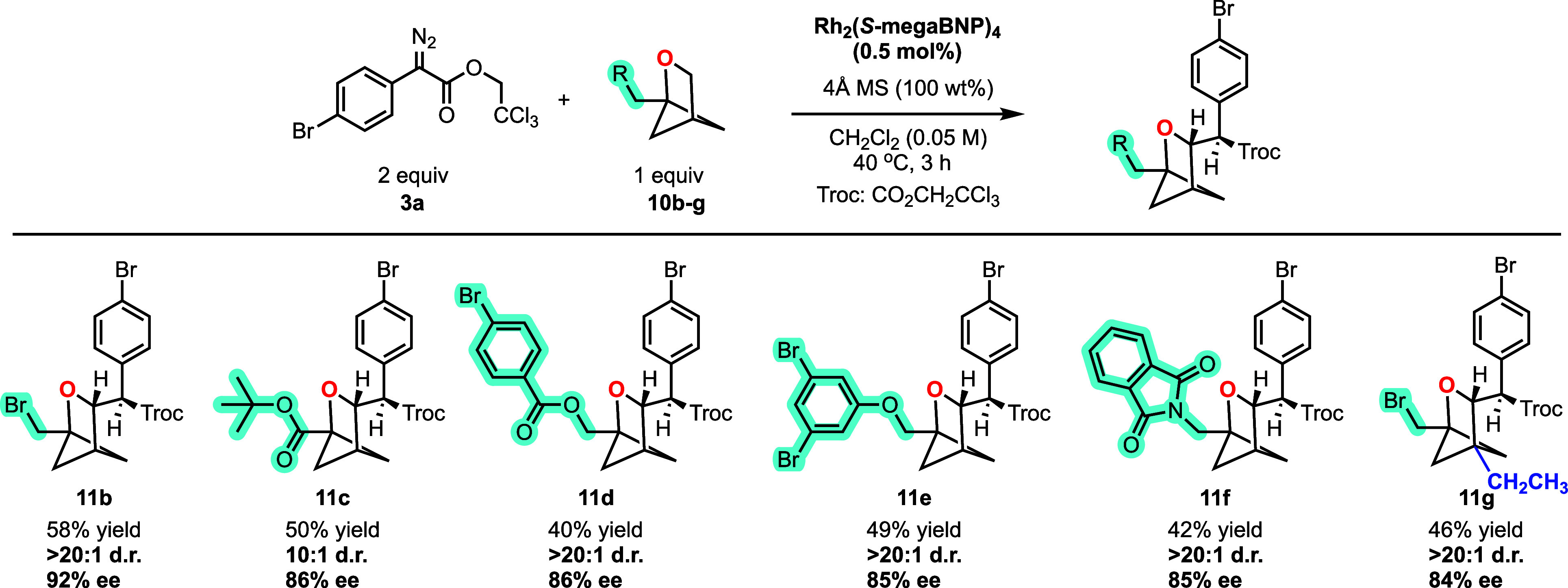
Reaction Scope of 2-Oxabicyclo[2.1.1]­hexane

Two 1-substituted 2-azabicyclo[2.1.1]­hexanes (2-azaBCH) were also examined as substrates. The reaction catalyzed by Rh_2_(*S*-megaBNP)_4_ was generally effective, favoring secondary C–H functionalization at the site adjacent to the nitrogen (Scheme 7). The reaction with the trifluoromethyl derivative **13a** gave **14a** in 32% yield with exceptionally high stereoselectivity (>20:1 d.r. 98% ee). A good reaction was also obtained with ester derivative **13b** forming product **14b** in 48% yield and 94% ee. In this case, the diastereoselectivity is conservatively estimated as >10:1 because the N-Boc hindered rotation makes analyzing the product ratio challenging.

**Scheme 7:**
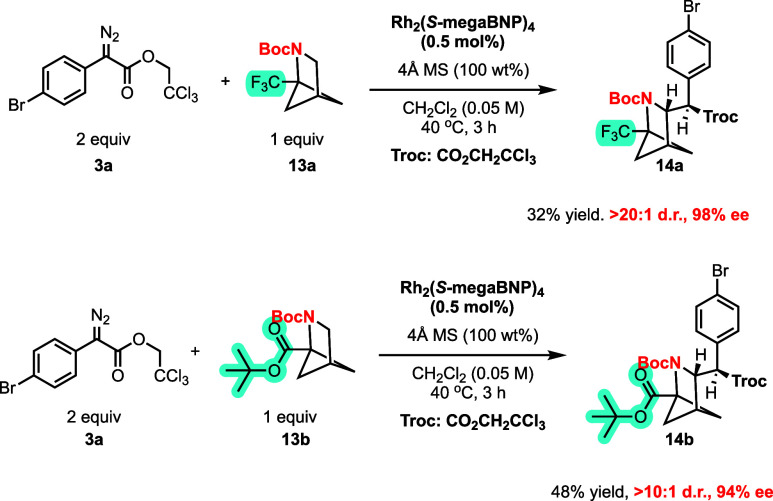
Reaction Scope of 2-Azabicyclo[2.1.1]­hexane

In general, the bicyclo[2.1.1]­hexane scaffold is a challenging system for rhodium carbene induced C–H functionalization. The dirhodium tetracarboxylate catalysts, which have performed well in a wide variety of C–H functionalization reactions, failed in this system, and only the binaphthyl phosphate catalyst Rh_2_(*S*-megaBNP)_4_ performed well. The remarkable behavior of Rh_2_(*S*-megaBNP)_4_ in this reaction deserves further analysis. X-ray crystallographic and solution NMR studies have previously revealed that the catalyst adopts a D_4_ symmetric structure and the 1,3-di-*tert*-butylphenyl functionality plays a crucial role in rigidifying this structure (Figure 1A, Top).^47^ To shed light on the above-reported selectivity of the Rh_2_(*S*-megaBNP)_4_-catalyzed C–H functionalization, we turned to computational analyses of the rhodium carbene intermediate and how it reacts with the bicyclo[2.1.1]­hexane scaffold. The large size of the catalysts (>400 atoms) makes the use of high-level DFT approaches impractical. Therefore, we applied a 2-layer ONIOM method^50–54^ where 16 *tert*-butyl groups of Rh_2_(*S*-megaBNP)_4_ were treated at the molecular mechanics (UFF)^55^ level but the rest of the system including carbene, substrate, and catalyst were described at the density functional (we used the M06 functional^56^) level (see Supporting Information for a discussion for the selection of this approach and comparison with other density functional methods). Confirmation that the ONIOM­(M06/UFF) level of calculation was appropriate for analyzing Rh_2_(*S*-megaBNP)_4_ is seen by a comparison of the calculated D_4_ symmetrical structure versus the X-ray crystallographic structure (with RMSD = 0.406 Å). The overlay of the crystal structure (blue) of Rh_2_(*S*-megaBNP)_4_ and the computationally optimized structure (green) is shown in Figure 1A, Bottom. During the computational studies, we utilized noncovalent interaction (NCI) map analyses to determine further the key interligand interactions that are involved in maintaining the high symmetric structure of the catalyst. These studies showed that inner C–H/O and outer C–H/π interactions are important contributors to the D_4_ symmetric orientation of the catalyst (see Figure S3, SI Section 7.2).^57,58^

**Figure 1:**
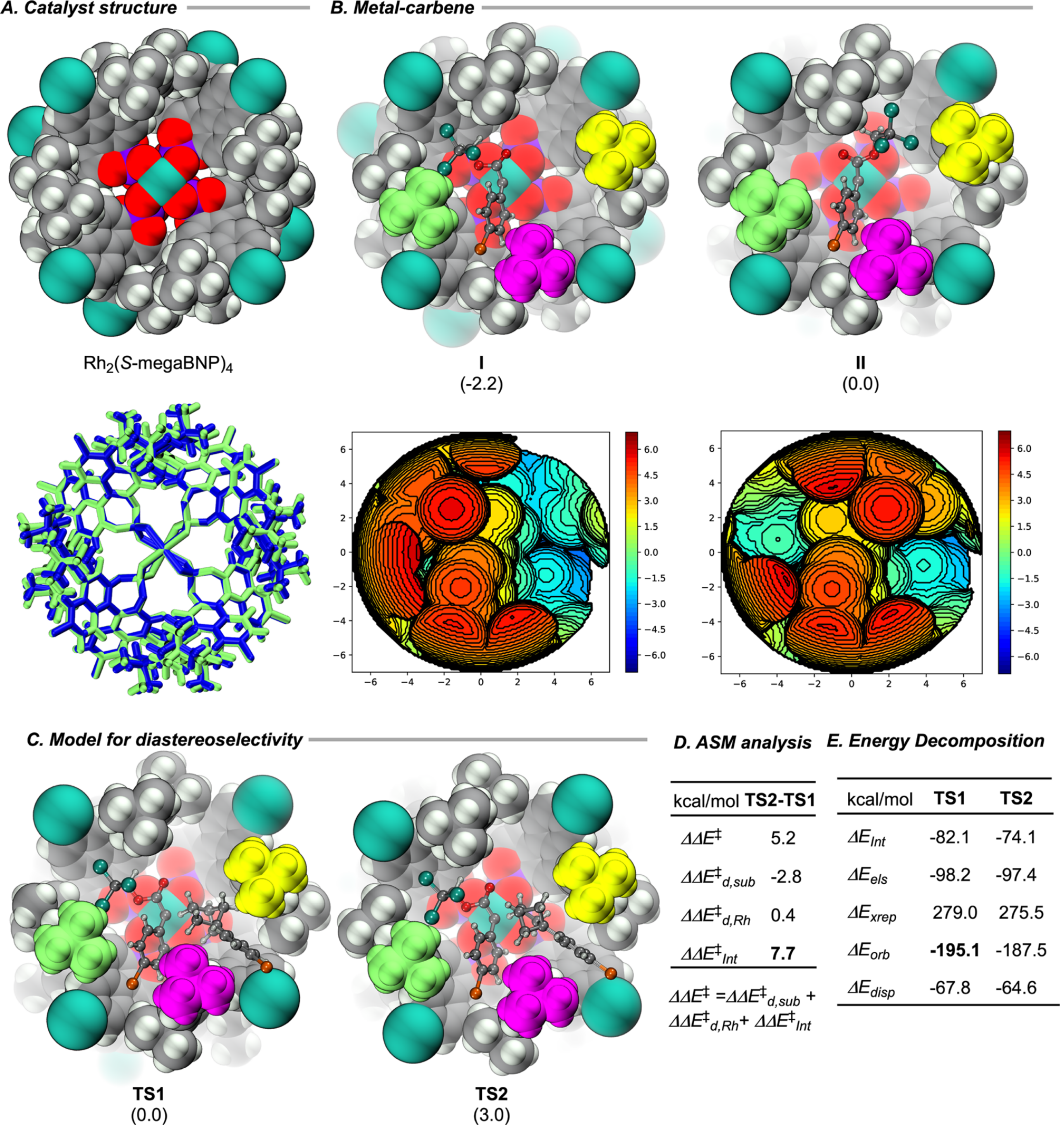
Computational study. (A) Top: DFT optimized structure of Rh_2_(*S*-megaBNP)_4_ (RMSD = 0.4 Å compared to X-ray structure); Bottom: an overlay of DFT optimized structure (green) and X-ray structure (blue). (B) Top: Two carbene isomers, reported energies are free Gibbs energy in kcal/mol. The highlighted *tert*-butyl groups in green, pink, and yellow are important for interaction with carbene and substrate; Bottom: Steric plots of carbene isomers **I** and **II** by the SambVca 2.1 program with the carbene’s carbon as the center of the map and the radius of 7 bohr. (C) Transition states of C–H insertion into 2 °C–H bond leading to **5a** from **TS1** and **6a** from **TS2**. (D) Activation-strain model analysis; the reported energies are the differences of **TS2** and **TS1**. ΔΔ*E*_d,sub_^‡^ and ΔΔ*E*_d,Rh_^‡^ distortion energy of substrate and rhodium-carbene fragments. ΔΔ*E*_Int_^‡^ interaction energy. (E) Energy decomposition analysis via the sobEDA method using the Multiwfn program. All figures were rendered by the VMD program.^68^

Empirical observations during the benchmark studies on Rh_2_(*S*-megaBNP)_4_ revealed that a *para*-substituent on the aryldiazoacetate is beneficial for high asymmetric induction.^47^ This observation led to the hypothesis that a para-substituent contributes to locking the carbene in a defined orientation between adjacent ligands. To validate this hypothesis, we turned to examine the structure of the rhodium-carbene complex generated by Rh_2_(*S*-megaBNP)_4_ at the ONIOM­(M06/UFF) level with the aim to identify orientations of the carbene in the reactive pocket of the catalyst. The study was conducted by using the carbene generated from **3a** with a *para*-bromophenyl substituent. The calculations revealed that there were only two favored orientations of the carbene in the complex (**I** and **II**) because of the high symmetry of the catalyst (Figure 1C). Generally, the aryl group of the carbene resides in the same plane as the rhodium carbene bond, and these two identified structures are differentiated by the orientation of the ester group which is aligned perpendicular to the rhodium carbene bond.^59–62^ The *p*-bromophenyl substituent on the carbene in both complexes **I** and **II** resides between two *tert*-butyl groups of adjacent ligands (colored green and purple in Figure 1B, Top). Furthermore, the *p*-bromophenyl group is tilted significantly out-of-plane toward the green *tert*-butyl group, presumably due to a favorable interaction with the naphthyl and *tert*-butyl moiety of the ligand. This tilting effect also sets the carbene into a pretransition state position opening up one face of the carbene for attack. The tilting effect could possibly explain the unique reactivity of Rh_2_(*S*-megaBNP)_4_ toward C–H functionalization of very difficult substrates such as the bicyclic substrates used in this study. Of the two possible orientations, carbene (**I**) with the trichloroethoxy group pointed toward the green *tert*-butyl side is more stable than carbene (**II**) by 2.2 kcal/mol. Furthermore, in carbene (**I**), the face of the carbene away from the green *tert*-butyl group is especially open for attack. This finding is further supported by the steric plot presented in Figure 1B, Bottom.^63^ This figure cleanly shows that the (*Si*)-face of the carbene complex (**I**) is fairly open while the (*Re*)-face is almost completely blocked. The distinction between the two faces is far more significant for the carbene complex (**I**) than the carbene complex (**II**). The observed asymmetric induction is consistent with the experimentally observed selective reaction at the (*Si*) face of the carbene

In order to validate the model generated thus far, we decided to challenge it to see if it would predict the high diastereoselectivity exhibited in the secondary C–H functionalization of the bicyclic substrates. It is well established that the C–H functionalization step proceeds in a concerted, asynchronous manner. In the case of the reaction with carbene complex (**I**), the substrate must approach between two adjacent *tert*-butyl groups colored purple and yellow (Figure 1C). Two transition states **TS1** and **TS2** leading to two possible diastereomers of the C–H functionalization products were located. The transition state **TS1** is 3.0 kcal/mol more stable than **TS2** and leads to the experimentally observed major diastereomer. A closer analysis of the two transition states reveals that the preference for **TS1** over **TS2** is because of the better orbital interaction (Figure 1D,E).^64,65^ It is well established that approach of the substrate toward the carbene has a well-defined trajectory^59^ and presumably the two *tert*-butyl groups influence the approach on the substrate. Rh_2_(*S*-megaBNP)_4_ is considered to be a relatively rigid catalyst, and so, it does not alter its shape in a significant way to accommodate the approaching substrate. Consequently, the energy difference between the two transition states depends how well aligned the substrate can be in the presence of the ligands to maximize favorable orbital overlap in the transition state during the C–H functionalization (Figure S6, SI Section 7.4).^66,67^

## Conclusion

This study illustrates that highly diastereoselective and enantioselective functionalization of secondary C–H bonds in BCHs and hetero-BCHs can be achieved by using the recently developed catalyst Rh_2_(*S*-megaBNP)_4_. The C–H functionalization is achieved by a carbene-induced C–H functionalization and demonstrates the effectiveness of rhodium-stabilized donor/acceptor carbenes in these reactions. Compared with traditional chiral dirhodium tetracarboxylate catalysts, the Rh_2_(*S*-megaBNP)_4_-catalyzed carbene reaction is far superior in this reaction to any of the dirhodium tetracarboxylates and other BNP derivatives that were tested. It can achieve the transformation in high yield and with exceptional control of the diastereoselectivity and enantioselectivity. Computational studies revealed the likely cause of its remarkable behavior. The catalyst exists in a D_4_ symmetric arrangement, and when the carbene is bound to the dirhodium, it adopts distinct orientations. Interactions between the aryl group of the bound carbene and the naphthyl and a *tert*-butyl group on the ligand causes the (*Re*) face of the carbene to tilt toward the catalyst wall opening the (*Si*) face to attack. When the bicyclic derivatives approach the carbene, they must do so with a defined orientation, resulting in a highly diastereoselective reaction. These studies illustrate the highly significant role that secondary noncovalent interactions of the catalyst wall can have on the outcome of the carbene-induced C–H functionalization. They also explain why donor/acceptor carbenes with aryl as the donor group are capable of such highly selective reactions, because the interaction of the aryl group with the catalyst is a critical element for defined orientation to the rhodium-carbene complex.

## Supplementary Material



## Notes

Complete experimental procedures, materials, computational details, compound characterizations, and crystal structure forms of compounds **5l** and **8a** (PDF)
