# Supplementary material for: Asymmetric C–H Functionalization of Bicyclo[2.1.1]hexanes and Their 2‑Oxa- and 2‑Aza Derivatives via Rhodium Carbene Intermediates
Source: J Am Chem Soc. 2026 Jan 5;148(2):2709–18. doi: 10.1021/jacs.5c19070 (PMC12833809; doi:10.1021/jacs.5c19070)
Supplement: Supplementary file 1 [file ja5c19070_si_001.pdf]

Asymmetric C–H functionalization of bicyclo[2.1.1]hexanes and their 2-oxa- and 2-aza derivatives via rhodium carbene intermediates

Ziyi Chen,[1] Duc Ly,[1] Yuzuru Kanda,[2] Vadym V. Levterov,[3] Yaroslav Panasiuk,[3,4] Pavel K. Mykhailiuk,[3,4] Djamaladdin G. Musaev[1,5,*] and Huw M. L. Davies[1,*]

[1]Department of Chemistry, Emory University, Atlanta, Georgia 30322, United States

[2]Global Discovery Chemistry, Novartis Institute of Biomedical Research, 250 Massachusetts Avenue, Cambridge MA, 02139, United States

[3]Enamine Ltd, Winston Churchill Street 78, 02094 Kyiv, Ukraine

[4]Taras Shevchenko National University of Kyiv, Volodymyrska Street 60, 01601 Kyiv, Ukraine

[5]Cherry L. Emerson Center for Scientific Computation, Emory University, 1521 Dickey Drive, Atlanta, Georgia 30322, United States

Correspondence to: hmdavie@emory.edu

Supporting Information

Table of Contents

1. **General Information** 3
2. **Substrate Synthesis and Characterization** 4
3. **General Procedure for C–H functionalization (General Procedure C)** 16
4. **C–H functionalization of bicyclic compounds via rhodium carbene** 17
   - 4.1 **Crude NMR Analysis for Catalyst Screening of Reactions with 3a and 4a** 17
   - 4.2 **Crude NMR Analysis for Catalyst Screening of Reactions with 3a and 10a** 21
   - 4.3 **C–H Functionalization of Bicyclo Compounds and Product Characterization** 23
   - 4.4 **Failed reactions with different aryl diazo acetate substrates** 58
5. **NMR Spectra** 63
6. **HPLC and SFC Chromatograms** 119
7. **Computational Calculation** 152
   - 7.1 **General information about computational study** 152
   - 7.2 **Validation of the ONIOM partitioning scheme for Rh_2(S-megaBNP)$_4$** 152
   - 7.3 **Analysis of rhodium-carbene complex of Rh_2(S-megaBNP)$_4$** 154
   - 7.4 **Analysis of the C-H functionalization transition states** 154
8. **X-ray Crystallographic Data** 176
   - 8.1 **X-ray Crystallographic Data of 5l** 176
   - 8.2 **X-ray Crystallographic Data of 8a** 190
9. **References** 208

1. General Information

Materials: If not especially mentioned, all chemicals are purchased as reagent grade from Sigma-Aldrich, AmBeed, Combi-block, Strem Chemicals, Oakwood Chemical. Bicyclic substrates (**10b, 10c, 10d, 10g, 13a and 13b**) were generously provided by ***Enamine*** and were directly used without further purification.

Instrumentation:

NMR: ^{1}H, ^{13}C and ^{19}F NMR spectra were recorded at 800 MHz (^{13}C at 201 MHz) on Bruker-800 spectrometer, 600 MHz (^{13}C at 151 MHz, ^{19}F at 565 MHz) on Bruker-600 spectrometer or Varian IVONA-600 spectrometer, or 400 MHz (^{13}C at 101 MHz, ^{19}F at 376 MHz) on Bruker-400 spectrometer and all were reported in parts per million (ppm). Unless otherwise noted, ^{1}H, ^{13}C and ^{19}F NMR spectra were performed in solutions of deuterated chloroform ($CDCl_3$) with the residue chloroform set as an internal standard (7.26 ppm for ^{1}H NMR, and 77.16 ppm for ^{13}C NMR). Abbreviations for signal multiplicity are as follow: br = broad, s = singlet, d = doublet, t = triplet, q = quartet, m = multiplet, dd = doublet of doublet, dt = doublet of triplet, td = triplet of doublet, ddd = doublet of doublet of doublet, etc. Coupling constants (J values) were calculated directly from the spectra.

IR: IR spectra were collected on a Nicolet iS10 FT-IR spectrometer.

HRMS: Mass spectra were taken on a Thermo Finnigan LTQ-FTMS spectrometer with APCI, ESI.

HPLC and SFC: Enantiomeric excess data were obtained from either Agilent 1100 series instrument High Performance Liquid Chromatography (HPLC) or Waters ACQUITY UPC^2 Supercritical Fluid Chromatography (SFC). The HPLC system operated with HPLC grade isopropanol/n-hexane gradient and commercial ChiralPak/ChiralCel columns from Daicel Chemical Industries, notably ChiralPak AD-H (5 µm particle size, 4.6 mm vs. 250 mm), ChiralPak AS-H (5 µm particle size, 4.6 mm vs. 250 mm), and Regis (R,R) Whelk-O 1 from Regis Technologies (5 µm particle size, 4.6 mm vs. 250 mm). The SFC system operated with supercritical CO_2 and HPLC grade (50% methanol in isopropanol with 0.2% formic acid) and commercial ChiralPak/ChiralCel columns from Daicel Chemical Industries, notably ChiralCel OJ-3 (3 µm particle size, 3.0 mm vs. 150 mm).

Chiral HPLC or SFC conditions were determined by obtaining separation of the racemic products using Rh_2(*R/S*-megaBNP)$_4$ or Rh_2(*R/S*-TPPTTL)$_4$ as catalyst for C–H insertion reactions.

Optical Rotation: Optical rotations were determined by Autopol IV (Rudolph Research Analytical).

2. Substrate Synthesis and Characterization

General Procedure A:

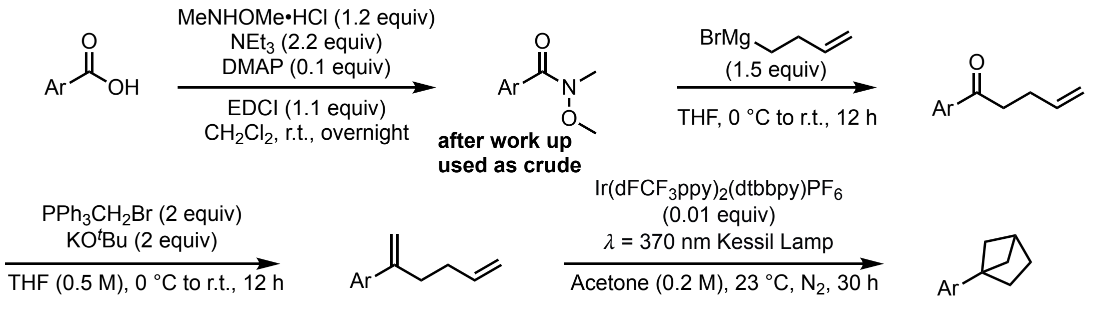

Synthesis of Amide: To a round-bottom flask was added the corresponding **benzoic acid** (1.0 equiv), EDCI (1.1 equiv), DMAP (0.1 equiv), *N*,*O*-dimethylhydroxylamine hydrochloride (1.2 equiv) and a magnetic stir bar. Dichloromethane (0.5 M) was added to the flask and the mixture was stirred at 0 °C for 5 min before the addition of triethylamine (2.2 equiv), then the mixture was stirred at room temperature overnight. After that, the stirring mixture was quenched with NH_4Cl (sat. aq.) at 0 °C and was extracted with ethyl acetate (50 mL X 3). The combined organic layer was dried over anhydrous sodium sulfate, concentrated and purified via column chromatography over silica gel to afford the **amide** and the amide was used as crude.

Synthesis of Enone: To a flame-dried flask was added the **amide** (1.0 equiv) and a magnetic stir bar. The flask was vacuumed and refilled with N_2 for three times before dry THF (0.5 M) was added in. Then the solution was stirred at 0 °C for 15 min and (but-3-en-1-yl) magnesium bromide (1.0 M, 1.5 equiv) was added in the stirring solution at 0 °C over 15 min. The mixture was gradually warmed up to room temperature over 4 h. After that, the stirring mixture was quenched with NH_4Cl (sat. aq.) at 0 °C and was extracted with diethyl ether (50 mL X 3). The combined organic layer was dried over anhydrous sodium sulfate, concentrated and purified via column chromatography over silica gel to afford the desired **enone**.

Synthesis of Diene: To an oven-dried flask was added methyl triphenylphosphonium bromide (2 equiv), potassium tert-butoxide (2 equiv) and a magnetic stir bar. The flask was vacuumed and refilled with N_2 for three times before the addition of dry THF (0.5 M) at 0 °C. The mixture was stirred at 0 °C for 1 hour and a solution of the corresponding **enone** (1 equiv) in 5 mL dry THF (from solvent purification system) was added to the stirring mixture over 3 min. Then the stirring mixture was gradually warmed up to room temperature overnight. After 12 h, the reaction was quenched with ammonium chloride (sat. aq.) at 0 °C and the mixture was extracted with diethyl ether (50 mL X 3). The combined organic layer was dried over anhydrous sodium sulfate, concentrated and purified via column chromatography over silica gel to afford the desired **diene**.

Synthesis of Bicyclo[2.1.1]hexane substrates: To a 25 mL glass tube was added the corresponding **diene** (1 equiv), Ir(dFCF$_3$ppy)$_2$(dtbbpy)PF$_6$ (0.01 equiv) and a magnetic stir bar. Acetone (0.2 M) was added to the mixture and the tube was sealed with a septa and electric tape.

While stirring at room temperature, the mixture was purged through Argon with an argon balloon for 15 min, then the septa was sealed with electric tape again. After that, a 370 nm Kessil light was set up 3 cm ~ 5 cm to the tube containing stirring reaction and the light was adjust to 75% intensity for 30 h at 23 °C. After the full consumption of starting material by TLC analysis, the crude was concentrated and purified via column chromatography over silica gel to afford the desired bicyclo compound.

General Procedure B:

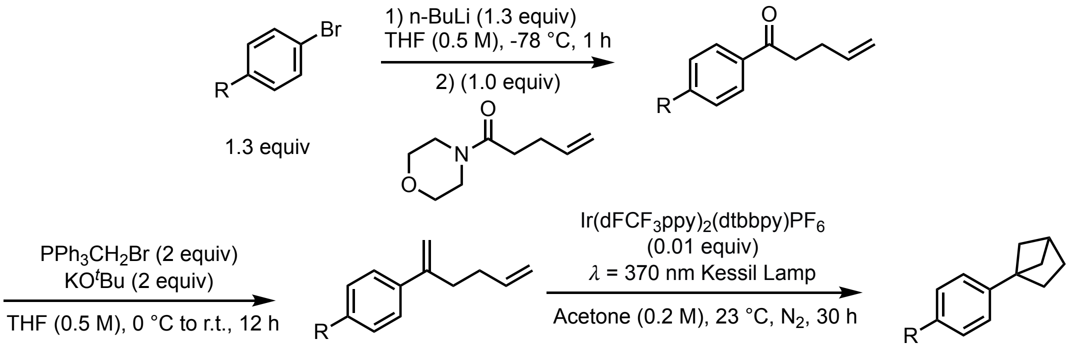

Synthesis of Enone: To a flame-dried flask was added the corresponding bromoarene (1.3 equiv) and a magnetic stir bar. The flask was vacuumed and refilled with N_2 for three times before dry THF (0.3 M) was added in. Then the solution was stirred at -78 °C for 15 min and n-BuLi (2.5 M, 1.3 equiv) was added in the stirring solution at -78 °C over 15 min. The mixture was kept stirring at -78 °C for 1 h before dropwise addition of a solution of 1-morpholinopent-4-en-1-one (1.0 equiv, prepared from reported procedure[1]) in dry THF (5 mL) at -78 °C. After that, the stirring mixture was gradually warmed up to room temperature overnight. After 12 h, the mixture was quenched with NH_4Cl (sat. aq.) at 0 °C and was extracted with diethyl ether (50 mL X 3). The combined organic layer was dried over anhydrous sodium sulfate, concentrated and purified via column chromatography over silica gel to afford the desired **enone**.

Synthesis of Diene: To an oven-dried flask was added methyl triphenylphosphonium bromide (2 equiv), potassium tert-butoxide (2 equiv) and a magnetic stir bar. The flask was vacuumed and refilled with N_2 for three times before the addition of dry THF (0.5 M) at 0 °C. The mixture was stirred at 0 °C for 1 hour and a solution of the corresponding **enone** (1 equiv) in 5 mL dry THF was added to the stirring mixture dropwise. Then the stirring mixture was gradually warmed up to room temperature overnight. After 12 h, the reaction was quenched with ammonium chloride (sat. aq.) at 0 °C and the mixture was extracted with diethyl ether (50 mL X 3). The combined organic layer was dried over anhydrous sodium sulfate, concentrated and purified via column chromatography over silica gel to afford the desired **diene**.

Synthesis of Bicyclo[2.1.1]hexane substrates: To a 25 mL glass tube was added 1-(*tert*-butyl)-4-(hexa-1,5-dien-2-yl)benzene (1 equiv), Ir(dFCF$_3$ppy)$_2$(dtbbpy)PF$_6$ (0.01 equiv) and a magnetic stir

bar. Acetone (0.2 M) was added to the mixture and the tube was sealed with a septa and electric tape. While stirring at room temperature, the mixture was purged through Argon with an argon balloon for 15 min, then the septa was sealed with electric tape again and an Argon balloon added through the septa. After that, a 370 nm Kessil light was set up 3 cm to the tube containing stirring reaction and the light was adjust to 75% intensity for 30 h at 23 °C. After the full consumption of starting material by TLC analysis, the crude was concentrated and purified via column chromatography over silica gel to afford the desired **bicyclic compound**.

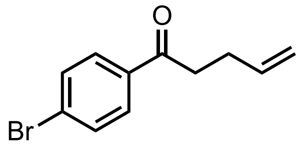

s1

Using **general procedure A**, 1-(4-bromophenyl)pent-4-en-1-one **s1** was obtained as a colorless liquid (86% yield, 5.08 g).

Characterization: The NMR spectra are in agreement with the published data[2].

^{1}H NMR (400 MHz, $CDCl_3$) δ 7.82 (d, J = 8.7 Hz, 2H), 7.60 (d, J = 8.7 Hz, 2H), 5.89 (ddt, J = 17.0, 10.2, 6.7 Hz, 1H), 5.08 (dq, *J* = 17.0, 1.7 Hz, 1H), 5.02 (ddt, *J* = 10.2, 1.7, 1.2 Hz, 1H), 3.04 (dd, *J* = 7.8, 6.7 Hz, 2H), 2.53 – 2.44 (m, 2H).

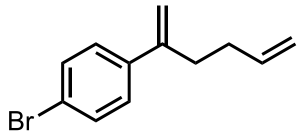

s2

Using **general procedure A**, 1-bromo-4-(hexa-1,5-dien-2-yl)benzene **s2** was obtained as a colorless liquid (82% yield, 810 mg).

Characterization: The NMR spectra are in agreement with the published data[3].

^{1}H NMR (400 MHz, $CDCl_3$) δ 7.45 (d, J = 8.6 Hz, 2H), 7.26 (d, J = 8.6 Hz, 2H), 5.82 (ddt, J = 16.9, 10.2, 6.6 Hz, 1H), 5.27 (d, J = 1.3 Hz, 1H), 5.09 (q, J = 1.3 Hz, 1H), 5.04 – 4.94 (m, 2H), 2.59 – 2.52 (m, 2H), 2.23 – 2.15 (m, 2H).

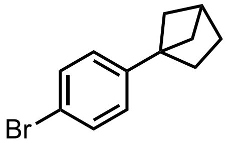

4a

Using **general procedure A**, 1-(4-bromophenyl)bicyclo[2.1.1]hexane **4a** was obtained as a white solid (81% yield, 656 mg).

Characterization:

^{1}H NMR (400 MHz, $CDCl_3$) δ 7.41 (d, J = 8.4 Hz, 2H), 7.11 (d, J = 8.4 Hz, 2H), 2.53 (t, J = 2.9 Hz, 1H), 1.83 – 1.80 (m, 4H), 1.75 – 1.71 (m, 2H), 1.47 – 1.40 (m, 2H);

^{13}C NMR (101 MHz, $CDCl_3$) δ 143.8, 131.3, 127.8, 119.6, 53.8, 43.4, 36.6, 33.4, 28.8;

IR (neat) 2963, 2911, 2868, 1494, 1474, 1455, 1440, 1393, 1332, 1282, 1216, 1205, 1173, 1160, 1101, 1069, 965, 920 cm^{-1}

HRMS (FTMS +p APCI) calcd for $C_{12}H_{13}{}^{79}Br$ $[M]^+$ 236.0195 found 236.0197.

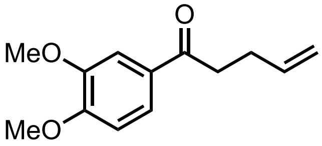

s3

Using **general procedure A**, 1-(3,4-dimethoxyphenyl)pent-4-en-1-one **s3** was obtained as a light-yellow liquid (89% yield, 2.94 g).

Characterization: The NMR spectra are in agreement with the published data[4].

^{1}H NMR (400 MHz, $CDCl_3$) δ 7.58 (dd, J = 8.4, 2.0 Hz, 1H), 7.53 (d, J = 2.0 Hz, 1H), 6.88 (d, J = 8.4 Hz, 1H), 5.89 (ddt, J = 17.0, 10.2, 6.8 Hz, 1H), 5.08 (dq, J = 17.0, 1.6 Hz, 1H), 5.00 (dq, J = 10.2, 1.6 Hz, 1H), 3.94 (s, 3H), 3.93 (s, 3H), 3.03 (dd, J = 8.0, 6.8 Hz, 2H), 2.53 – 2.42 (m, 2H).

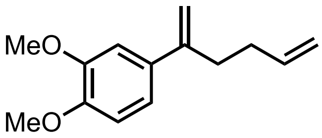

s4

Using **general procedure A**, 4-(hexa-1,5-dien-2-yl)-1,2-dimethoxybenzene **s4** was obtained as a colorless liquid (85% yield, 2.49 g).

Characterization:

^{1}H NMR (400 MHz, $CDCl_3$) δ 6.99 – 6.92 (m, 2H), 6.83 (d, J = 8.1 Hz, 1H), 5.85 (ddt, J = 16.9, 10.2, 6.6 Hz, 1H), 5.23 (d, J = 1.4 Hz, 1H), 5.06 – 4.94 (m, 3H), 3.90 (s, 3H), 3.89 (s, 3H), 2.61 – 2.52 (m, 2H), 2.28 – 2.17 (m, 2H)

^{13}C NMR (101 MHz, $CDCl_3$) δ 148.8, 148.7, 147.6, 138.4, 134.3, 118.6, 114.8, 111.3, 111.0, 109.7, 56.1, 56.0, 35.0, 32.7

IR (neat) 2998, 2933, 2836, 1722, 1673, 1641, 1593, 1514, 1464, 1414, 1326, 1263, 1172, 1145, 1092, 1056, 1025, 953, 944 cm^{-1}

HRMS (FTMS +p APCI) calcd for $C_{14}H_{19}O_2$ $[M+H]^+$ 219.1380 found 219.1381.

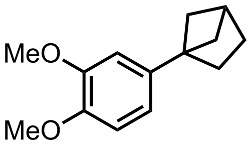

4d

Using **general procedure A**, 1-(3,4-dimethoxyphenyl)bicyclo[2.1.1]hexane **4d** was obtained as a white solid (82% yield, 890 mg).

Characterization:

^{1}H NMR (400 MHz, $CDCl_3$) δ 6.85 – 6.75 (m, 3H), 3.88 (s, 3H), 3.86 (s, 3H), 2.51 (tt, J = 2.7, 1.1 Hz, 1H), 1.87 – 1.77 (m, 4H), 1.77 – 1.69 (m, 2H), 1.47 – 1.38 (m, 2H)

^{13}C NMR (101 MHz, $CDCl_3$) δ 148.8, 147.3, 137.6, 117.9, 111.1, 109.4, 56.1, 55.9, 54.0, 43.6, 36.4, 33.3, 28.9

IR (neat) 2965, 2958, 2941, 2916, 2907, 2871, 1587, 1517, 1463, 1411, 1341, 1268, 1251, 1230, 1208, 1174, 1155, 1137, 1030 cm^{-1}

HRMS (FTMS +p APCI) calcd for $C_{14}H_{19}O_2$ $[M+H]^+$ 219.1380 found 219.1379.

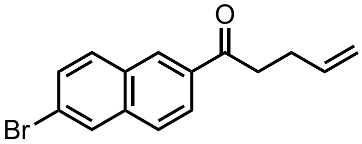

s5

Using **general procedure A**, 1-(6-bromonaphthalen-2-yl)pent-4-en-1-one **s5** was obtained as a colorless liquid (93% yield, 1.25 g).

Characterization:

^{1}H NMR (400 MHz, $CDCl_3$) δ 8.46 – 8.41 (m, 1H), 8.06 (dd, J = 9.0, 1.9 Hz, 2H), 7.83 (d, J = 9.0 Hz, 1H), 7.81 (d, J = 9.0 Hz, 1H), 7.63 (dd, J = 9.0, 1.9 Hz, 1H), 5.94 (ddt, J = 17.0, 10.2, 6.7 Hz, 1H), 5.12 (dq, J = 17.0, 1.6 Hz, 1H), 5.04 (dq, J = 10.2, 1.6 Hz, 1H), 3.20 (dd, J = 7.9, 6.7 Hz, 2H), 2.60 – 2.50 (m, 2H)

^{13}C NMR (101 MHz, $CDCl_3$) δ 199.2, 137.4, 136.6, 134.7, 131.2, 131.1, 130.5, 130.1, 129.6, 127.7, 125.2, 122.9, 115.6, 38.0, 28.4

IR (neat) 3080, 2998, 2979, 2917, 2875, 1681, 1641, 1617, 1585, 1497, 1459, 1415, 1396, 1383, 1353, 1300, 1268, 1245, 1199, 1172, 1134, 1067, 1014, 966 cm^{-1}

HRMS (FTMS +p APCI) calcd for $C_{15}H_{14}O^{79}Br$ $[M+H]^+$ 289.0223 found 289.0225.

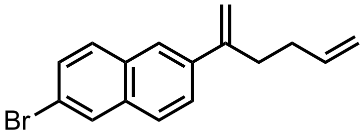

s6

Using **general procedure A**, 2-bromo-6-(hexa-1,5-dien-2-yl)naphthalene **s6** was obtained as a colorless liquid (90% yield, 980 mg).

Characterization:

^{1}H NMR (400 MHz, $CDCl_3$) δ 7.98 (d, J = 1.9 Hz, 1H), 7.78 (d, J = 1.9 Hz, 1H), 7.71 (d, J = 3.5 Hz, 1H), 7.69 (d, J = 3.5 Hz, 1H), 7.60 (dd, J = 8.7, 1.9 Hz, 1H), 7.54 (dd, J = 8.7, 1.9 Hz, 1H), 5.87 (ddt, J = 16.9, 10.2, 6.6 Hz, 1H), 5.44 (d, J = 1.3 Hz, 1H), 5.20 (q, J = 1.3 Hz, 1H), 5.06 – 4.95 (m, 2H), 2.74 – 2.65 (m, 2H), 2.31 – 2.21 (m, 2H)

^{13}C NMR (101 MHz, $CDCl_3$) δ 147.5, 139.1, 138.1, 133.9, 132.0, 129.9, 129.7, 129.6, 127.1, 125.9, 124.7, 119.8, 115.0, 113.7, 34.8, 32.6

IR (neat) 3075, 2977, 2932, 2857, 1640, 1586, 1562, 1494, 1462, 1414, 1360, 1329, 1299, 1268, 1177, 1138, 1112, 1062, 955, 909 cm^{-1}

HRMS (FTMS +p APCI) calcd for $C_{16}H_{16}{}^{79}Br$ $[M+H]^+$ 287.0430 found 287.0434.

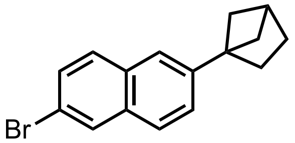

4e

Using **general procedure A**, 1-(6-bromonaphthalen-2-yl)bicyclo[2.1.1]hexane **4e** was obtained as a white solid (91% yield, 454 mg).

Characterization:

^{1}**H NMR** (400 MHz, $CDCl_3$) δ 7.96 (d, J = 2.0 Hz, 1H), 7.68 (d, J = 8.6 Hz, 1H), 7.67 – 7.64 (m, 1H), 7.62 – 7.59 (m, 1H), 7.51 (dd, J = 8.6, 2.0 Hz, 1H), 7.42 (dd, J = 8.6, 1.7 Hz, 1H), 2.60 – 2.56 (m, 1H), 1.95 – 1.89 (m, 2H), 1.89 – 1.82 (m, 4H), 1.57 – 1.50 (m, 2H)

^{13}**C NMR** (101 MHz, $CDCl_3$) δ 149.0, 147.7, 138.8, 136.7, 123.8, 51.5, 43.3, 37.1, 33.1, 28.6.

IR (neat) 2968, 2941, 2916, 2908, 2868, 1590, 1360, 1202, 1176, 1134, 1059, 903 cm^{-1}

HRMS (FTMS +p APCI) calcd for $C_{16}H_{16}{}^{79}Br$ $[M+H]^+$ 287.0433 found 287.0434.

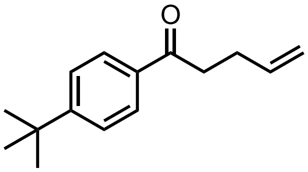

s7

Using **general procedure B**, 1-(4-(*tert*-butyl)phenyl)pent-4-en-1-one **s7** was obtained as a colorless liquid (71% yield, 2.11 g).

Characterization: The NMR spectra are in agreement with the published data[4].

^{1}**H NMR** (400 MHz, $CDCl_3$) δ 7.91 (d, J = 8.6 Hz, 2H), 7.48 (d, J = 8.6 Hz, 2H), 5.91 (ddt, J = 17.0, 10.2, 6.7 Hz, 1H), 5.09 (dq, J = 17.0, 1.6 Hz, 1H), 5.01 (dq, J = 10.2, 1.6 Hz, 1H), 3.08 (dd, J = 7.9, 6.7 Hz, 2H), 2.54 – 2.45 (m, 2H), 1.34 (s, 9H).

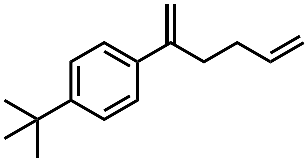

s8

Using **general procedure B**, 1-(*tert*-butyl)-4-(hexa-1,5-dien-2-yl)benzene **s8** was obtained as a colorless liquid (99% yield, 980 mg).

Characterization: The NMR spectra are in agreement with the published data[5].

^{1}**H NMR** (400 MHz, $CDCl_3$) δ 7.36 (s, 4H), 5.86 (ddt, J = 16.9, 10.2, 6.6 Hz, 1H), 5.29 (d, J = 1.5 Hz, 1H), 5.06 – 5.00 (m, 2H), 4.98 (ddt, J = 10.2, 2.2, 1.2 Hz, 1H), 2.64 – 2.55 (m, 2H), 2.28 – 2.20 (m, 2H), 1.33 (s, 9H).

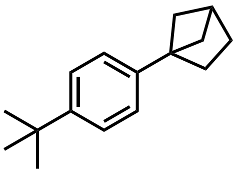

4b

Using **general procedure B**, 1-(4-(*tert*-butyl)phenyl)bicyclo[2.1.1]hexane **4b** was obtained as a white solid (81% yield, 926 mg).

Characterization:

^{1}H NMR (400 MHz, $CDCl_3$) δ 7.34 (d, J = 7.9 Hz, 2H), 7.21 (d, J = 7.9 Hz, 2H), 2.54 – 2.49 (m, 1H), 1.88 – 1.78 (m, 4H), 1.78 – 1.73 (m, 2H), 1.46 – 1.39 (m, 2H), 1.32 (s, 9H)

^{13}C NMR (101 MHz, $CDCl_3$) δ 148.7, 141.7, 125.8, 125.1, 54.0, 43.5, 36.6, 34.5, 33.1, 31.6, 28.8

IR (neat) 2961, 2904, 2869, 1522, 1475, 1460, 1392, 1362, 1336, 1269, 1201, 1112, 1017 cm^{-1}

HRMS (FTMS +p APCI) calcd for $C_{16}H_{22}$ $[M]^+$ 224.1716 found 224.1719.

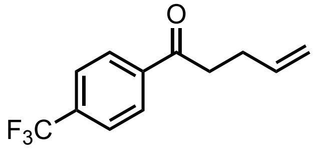

s9

Using **general procedure B**, 1-(4-(trifluoromethyl)phenyl)pent-4-en-1-one **s9** was obtained as a colorless liquid (51% yield, 2.15 g).

The NMR spectra are in agreement with the published data[6].

^{1}H NMR (400 MHz, $CDCl_3$) δ 8.07 (d, J = 8.0 Hz, 2H), 7.74 (d, J = 8.0 Hz, 2H), 5.90 (ddt, J = 17.0, 10.2, 6.5 Hz, 1H), 5.10 (dq, J = 17.0, 1.7 Hz, 1H), 5.03 (dq, J = 10.2, 1.4 Hz, 1H), 3.11 (t, J = 7.3 Hz, 2H), 2.56 – 2.47 (m, 2H)

^{19}F NMR (376 MHz, $CDCl_3$) δ -63.08.

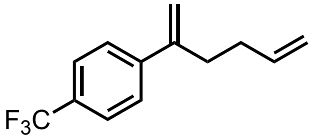

s10

Using **general procedure B**, 1-(hexa-1,5-dien-2-yl)-4-(trifluoromethyl)benzene **s10** was obtained as a light-yellow liquid (54% yield, 1.14 g).

Characterization: The NMR spectra are in agreement with the published data[5].

^{1}H NMR (400 MHz, $CDCl_3$) δ 7.58 (d, J = 8.1 Hz, 2H), 7.49 (d, J = 8.1 Hz, 2H), 5.83 (ddt, J = 16.9, 10.2, 6.6 Hz, 1H), 5.35 (d, J = 1.3 Hz, 1H), 5.18 (d, J = 1.3 Hz, 1H), 5.05 – 4.96 (m, 2H), 2.64 – 2.57 (m, 2H), 2.25 – 2.17 (m, 2H).;

^{19}F NMR (376 MHz, $CDCl_3$) δ -62.49.

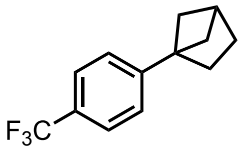

4c

Using **general procedure B**, 1-(4-(trifluoromethyl)phenyl)bicyclo[2.1.1]hexane **4c** was obtained as a light-yellow solid (92% yield, 929 mg).

Characterization:

^{1}H NMR (400 MHz, $CDCl_3$) δ 7.55 (d, J = 8.0 Hz, 2H), 7.33 (d, J = 8.0 Hz, 2H), 2.56 (t, J = 2.9 Hz, 1H), 1.88 – 1.82 (m, 4H), 1.80 – 1.73 (m, 2H), 1.52 – 1.45 (m, 2H)

^{13}C NMR (101 MHz, $CDCl_3$) δ 149.0, 128.20 (q, J = 32.0 Hz), 127.7, 126.3, 125.18 (q, J = 3.8 Hz), 124.53 (q, J = 272.9 Hz), 54.1, 43.3, 36.8, 33.7, 28.8

^{19}F NMR (376 MHz, $CDCl_3$) δ -62.26

IR (neat) 2968, 2874, 1619, 1410, 1325, 1285, 1204, 1162, 1123, 1108, 1070, 1018 cm^{-1}

HRMS (FTMS +p APCI) calcd for $C_{13}H_{13}F_3$ $[M]^+$ 226.0965 found 226.0964.

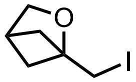

10a

Prepared using reported procedure[7], 1-(iodomethyl)-2-oxabicyclo[2.1.1]hexane **10a** was obtained as a light-yellow liquid (52% yield, 3.3 g).

Characterization: The NMR spectra are in agreement with the published data[7].

^{1}H NMR (400 MHz, $CDCl_3$) δ 3.83 (s, 2H), 3.47 (s, 2H), 2.85 (t, J = 3.2 Hz, 1H), 1.81 – 1.72 (m, 2H), 1.58 – 1.50 (m, 2H).

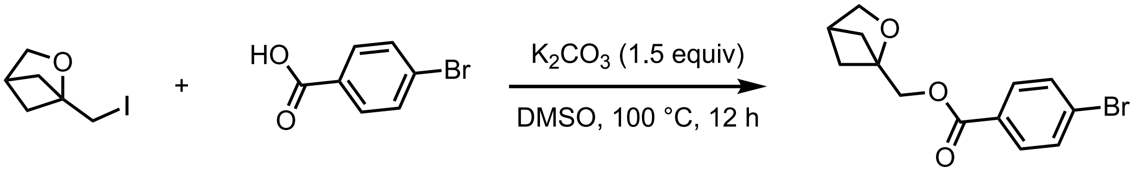

10e

To a 20 mL vial with a magnet stir bar was added 1-(iodomethyl)-2-oxabicyclo[2.1.1]hexane (150 mg, 1 equiv), 4-bromobenzoic acid (202 mg, 1.5 equiv) and 10 mL DMSO. While stirring, potassium carbonate (139 mg, 1.5 equiv) was added to the vial, then the mixture was heated to 100 °C. After 12 h, the mixture was cooled to room temperature before being diluted with 10 mL H_2O. Then the mixture was extracted with ethyl acetate (10 mL X 3), washed with brine (5 mL X 2), dried over anhydrous sodium sulfate and concentrated before purification via silica gel. Column chromatography afford (2-oxabicyclo[2.1.1]hexan-1-yl)methyl 4-bromobenzoate **10e** in 78 % yield (154 mg) as a white soild.

Characterization:

^{1}H NMR (400 MHz, $CDCl_3$) δ 7.91 (d, J = 8.5 Hz, 2H), 7.57 (d, J = 8.5 Hz, 2H), 4.57 (s, 2H), 3.83 (s, 2H), 2.95 (t, J = 3.2 Hz, 1H), 1.90 – 1.79 (m, 2H), 1.60 – 1.52 (m, 2H)

^{13}C NMR (101 MHz, $CDCl_3$) δ 165.7, 131.8, 131.4, 129.0, 128.3, 86.9, 70.0, 63.9, 40.7, 38.0

IR (neat) 2998, 2949, 2884, 1721, 1590, 1484, 1398, 1336, 1308, 1269, 1174, 1153, 1114, 1070, 1012, 968, 954, 937, 920 cm^{-1}

HRMS (FTMS +p APCI) calcd for $C_{13}H_{14}O_3{}^{79}Br$ $[M+H]^+$ 297.0121 found 297.0125.

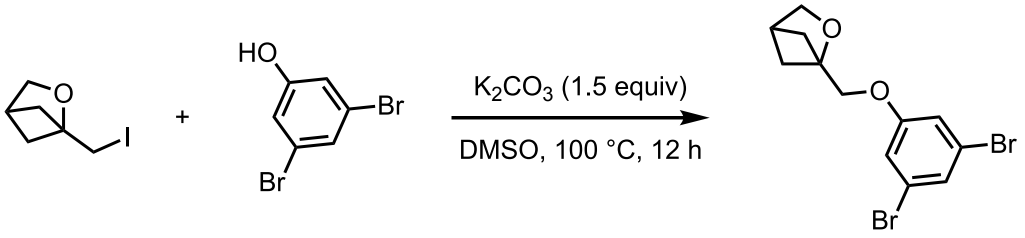

10f

To a 20 mL vial with a magnet stir bar was added 1-(iodomethyl)-2-oxabicyclo[2.1.1]hexane (150 mg, 1 equiv), 3,5-dibromophenol (253 mg, 1.5 equiv) and 10 mL DMSO. While stirring, potassium carbonate (139 mg, 1.5 equiv) was added to the vial, then the mixture was heated to 100 °C. After 12 h, the mixture was cooled to room temperature before being diluted with 10 mL H_2O. Then the mixture was extracted with ethyl acetate (10 mL X 3), washed with brine (5 mL X 2), dried over anhydrous sodium sulfate and concentrated before purification via silica gel. Column

chromatography afford 1-((3,5-dibromophenoxy)methyl)-2-oxabicyclo[2.1.1]hexane **10f** in 48 % yield (112 mg) as a colorless liquid.

Characterization:

^{1}H NMR (400 MHz, $CDCl_3$) δ 7.23 (t, J = 1.7 Hz, 1H), 7.03 (d, J = 1.7 Hz, 2H), 4.17 (s, 2H), 3.84 (s, 2H), 2.96 (t, J = 3.3 Hz, 1H), 1.90 – 1.80 (m, 2H), 1.61 – 1.53 (m, 2H)

^{13}C NMR (101 MHz, $CDCl_3$) δ 160.1, 126.7, 123.1, 117.2, 86.9, 70.0, 67.7, 40.6, 38.0

IR (neat) 2998, 2946, 2883, 1583, 1558, 1438, 1419, 1398, 1298, 1254, 1231, 1109, 1091, 1072, 1044, 1031, 987, 969, 937 cm^{-1}

HRMS (FTMS +p APCI) calcd for $C_{12}H_{13}O_2{}^{79}Br_2$ $[M+H]^+$ 346.9282 found 346.9277(Δ=1.5 ppm).

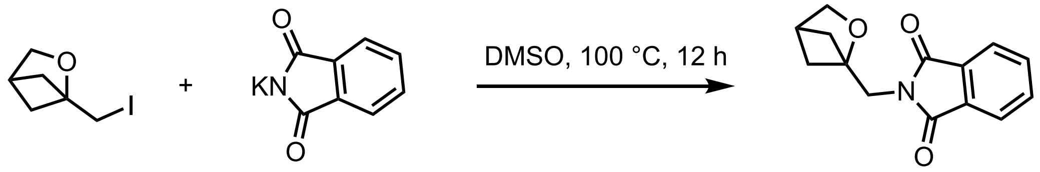

10g

To a 20 mL vial with a magnet stir bar was added 1-(iodomethyl)-2-oxabicyclo[2.1.1]hexane (150 mg, 1 equiv), potassium 1,3-dioxoisoindolin-2-ide (186 mg, 1.5 equiv) and 10 mL DMSO. While stirring, potassium carbonate (139 mg, 1.5 equiv) was added to the vial, then the mixture was heated to 100 °C. After 12 h, the mixture was cooled to room temperature before being diluted with 10 mL H_2O. Then the mixture was extracted with ethyl acetate (10 mL X 3), washed with brine (5 mL X 2), dried over anhydrous sodium sulfate and concentrated before purification via silica gel. Column chromatography afford 2-((2-oxabicyclo[2.1.1]hexan-1-yl)methyl)isoindoline-1,3-dione **10g** in 64 % yield (104 mg) as a white solid.

Characterization:

^{1}H NMR (400 MHz, $CDCl_3$) δ 7.89 – 7.82 (m, 2H), 7.74 – 7.68 (m, 2H), 4.06 (s, 2H), 3.76 (s, 2H), 2.85 (t, J = 3.2 Hz, 1H), 1.83 – 1.75 (m, 2H), 1.52 – 1.44 (m, 2H)

^{13}C NMR (101 MHz, $CDCl_3$) δ 168.3, 134.1, 132.2, 123.5, 87.5, 70.1, 41.4, 39.0, 37.5

IR (neat) 2998, 2941, 2885, 1773, 1715, 1467, 1426, 1394, 1342, 1317, 1294, 1176, 1063, 1027, 982, 968, 957 cm^{-1}

HRMS (FTMS +p APCI) calcd for $C_{14}H_{14}O_3N$ $[M+H]^+$ 244.0968 found 244.0969.

3. General Procedure for C–H functionalization (*General Procedure C*)

General Procedure C:

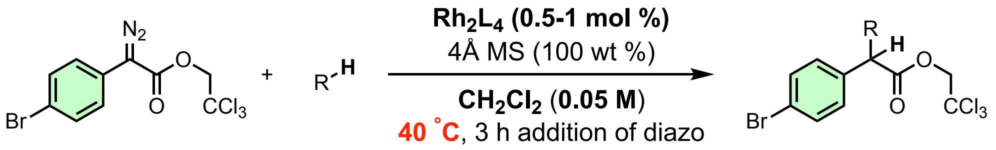

To a 4 mL vial **A** with a magnetic stir bar (bar shaped) was added 4 Å Molecular Sieves (100 wt %) then capped. Vial A and a capped empty vial **B** was flame-dried under vacuum and was refilled with nitrogen once. After both vials were cooled down to room-temperature, the corresponding catalyst (0.5-1 mol %) and alkane substrate (0.1 mmol, 1 equiv) were added to vial **A**, then the corresponding diazo compound (0.2 mmol, 2 equiv) was added to vial **B**. Both capped vials were vacuumed and refilled with nitrogen for three times before 1 mL dichloromethane (from solvent purification system) was introduced into both vials. Then, vial **A** was stirred at 40 °C with a nitrogen balloon for 5 min before the solution of diazo in vial **B** was added to vial **A** over 3 h via syringe pump with a 1 mL syringe. After the addition of diazo, the residue in vial **B** and syringe was washed with 0.5 mL dichloromethane (from solvent purification system) and was added into vial **A** dropwise.

After 30 min, the mixture was passed through a silica plug into a 20 mL vial, whose solvent was evaporated and was analyzed as crude sample to determine the regioselectivity and diastereoselectivity. Then the crude material was recovered for column chromatography with silica gel and the purified product (obtained yield) was analyzed by chiral HPLC or SFC to determine the enantioselectivity.

4. C–H functionalization of bicyclic compounds via rhodium carbene

4.1 Crude NMR Analysis for Catalyst Screening of Reactions with 3a and 4a

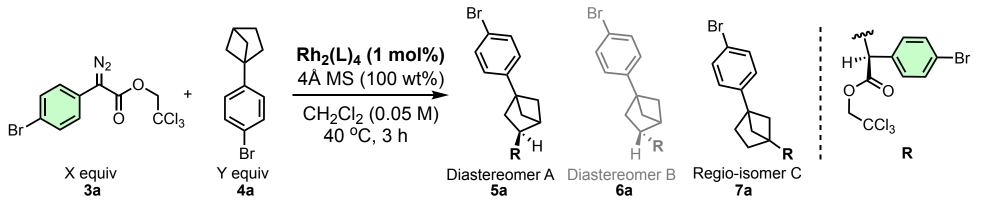

Entry	Catalysts	X:Y	Combined Yield[b] (%)	r.r. (5a+6a:7a)	d.r. (5a:6a)	ee (%)
1	Rh_2(*S*-DOSP)$_4$	1:2	< 5	-	-	-
2	Rh_2(*S*-p-PhTPCP)$_4$	1:2	< 5	-	-	-
3	Rh_2(*S*-TCPTAD)$_4$	1:2	< 5	-	-	-
4	Rh_2(*S*-TPPTTL)$_4$	1:2	< 5	-	-	-
5	Rh_2(*S*-NTTL)$_4$	1:2	< 5	-	-	-
6	Rh_2(*S*-2Cl5BrTPCP)$_4$	1:2	30	15:1	3:1	-80 (*ent*-**5a**)
7	Rh_2(*S*-BNP)$_4$	1:2	< 5	-	-	-
8[c]	Rh_2(*S*-Ph$_4$-BNP)$_4$	1:2	<5	-	-	-
9[c]	Rh_2(*S*-megaBNP)$_4$	1:2	43(40[d])	3:1	>20:1	99(**5a**), 93(**7a**)
10[c]	Rh_2(*S*-megaBNP)$_4$	1:1	47	2:1	>20:1	99(**5a**), 93(**7a**)
11[c]	Rh_2(*S*-megaBNP)$_4$	1.5:1	78	2:1	>20:1	99(**5a**), 93(**7a**)
12[c]	Rh_2(*S*-megaBNP)$_4$	**2:1**	**92(89[d])**	2:1	**>20:1**	**99(5a), 93(7a)**

a. 0.1 mmol substrate and 1 mol % catalyst were dissolved with 1 mL CH_2Cl_2 in a 4 mL vial. 0.2 mmol of diazo was dissolved in 1 mL CH_2Cl_2 and was added to the reaction vial via syringe pump over 3 h at 40 °C (0.05 M). d.r. and NMR yields were obtained from crude 1H ^{N}MR with trimethoxybenzene as internal standard
b. NMR yield, using 1,3,5-trimethoxybenzene as internal standard
c. 0.5 mol% catalyst was used
d. isolation yield

Crude NMR for Rh_2(*S*-DOSP)$_4$ and Rh_2(*S*-p-PhTPCP)$_4$ were not obtained because TLC analysis indicates the same results with entry 3-6, showing no desired product (desired product is CAM active).

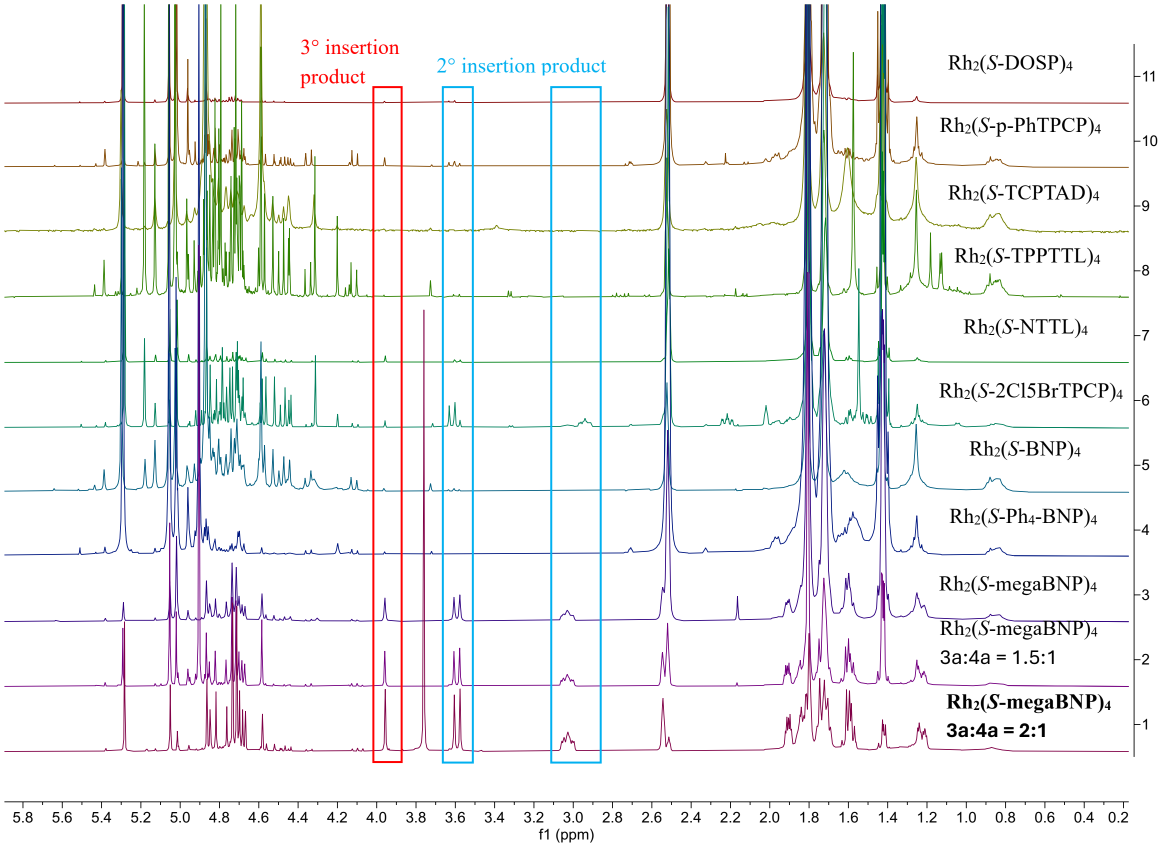

Zoomed in 5.8-0.2 ppm area of ^{1}H NMR for comparison among various catalysts

Note: All reactions achieved 100% consumption of diazo **3a**, but **a few catalysts failed (entry 1-5, 7 and 8) to give desired C–H functionalized products (major carbene dimer and other unidentified side products are formed)**. Favored major diastereomer from Rh$_2$(*S*-2Cl-5BrTPCP)$_4$ is different from the major diastereomer from Rh$_2$(*S*-megaBNP)$_4$.

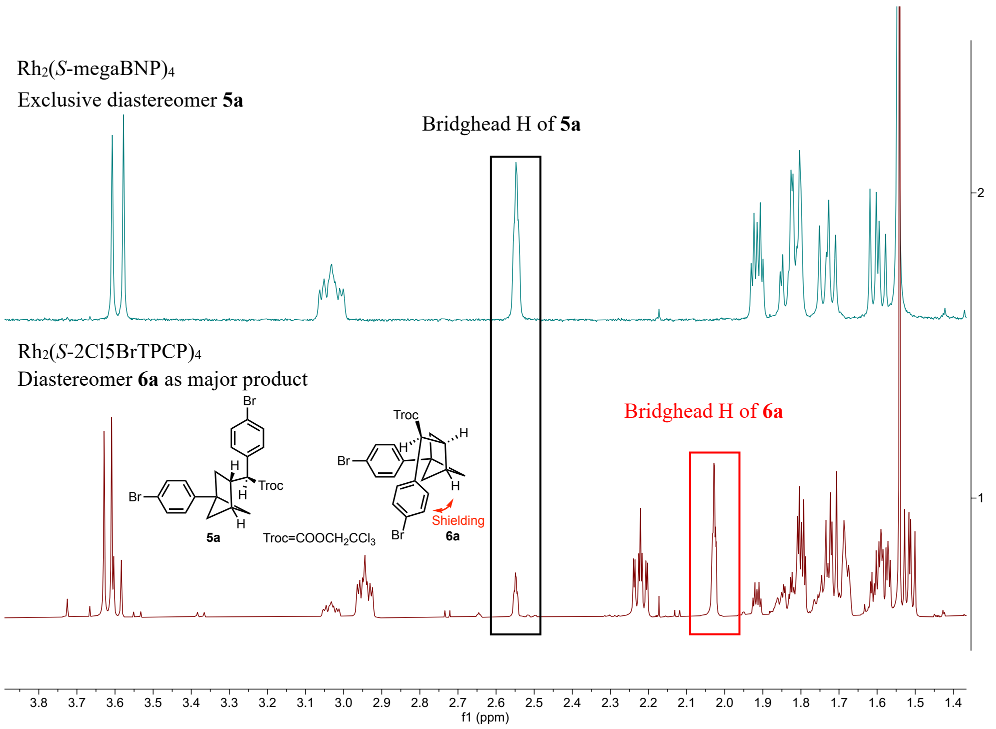

Zoomed in respective ^{1}H NMR spectra of reactions with Rh_2(*S*-megaBNP)$_4$ and Rh_2(*S*-2Cl5BrTPCP)$_4$ to study the relative chemistry between the two diastereomer **5a** and **6a**.

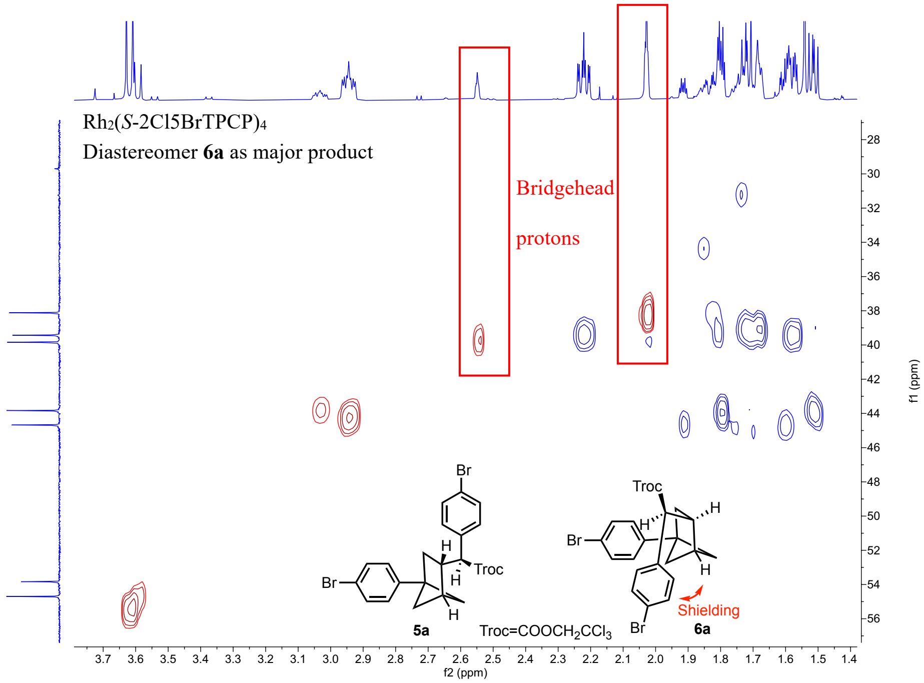

HSQC (^{1}H and DEPT) of mixed product (**5a** and **6a**) from reaction with Rh_2(*S*-2Cl5BrTPCP)$_4$.
Red color indicates the protons are attached to primary or tertiary carbons.

From NMR analysis, we determined that major diastereomer generated from reaction with Rh_2(*S*-megaBNP)$_4$ have a relative stereochemistry as shown in **5a** as the main product because we observed a significant shielding of the bridgehead proton (confirmed by HSQC) over 0.5 ppm.

4.2 Crude NMR Analysis for Catalyst Screening of Reactions with 3a and 10a

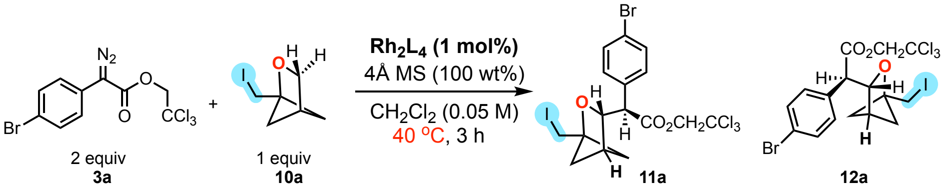

Entry[a]	Catalysts	Yield of **11a** (%)	d.r. (**11a**:**12a**)	ee (%)
1	Rh_2(*S*-DOSP)$_4$	20	1:1	-
2	Rh_2(*S*-PTAD)$_4$	23	1:1	-
3	Rh_2(*S*-TCPTAD)$_4$	11	1:1	-
4	Rh_2(*S*-2Cl5BrTPCP)$_4$	15	1:2	-
5	Rh_2(*S*-NTTL)$_4$	14	1:1	-
6	Rh_2(*S*-TPPTTL)$_4$	59	4:1	-79
7	Rh_2(*S*-BNP)$_4$	24	3:1	-
8[b]	**Rh_2(*S*-megaBNP)$_4$**	**43(40[c])**	**>20:1**	**90**

Note that the enantioselectivity for entry 1-5 and 7 is not determined because of low diastereoselectivity.

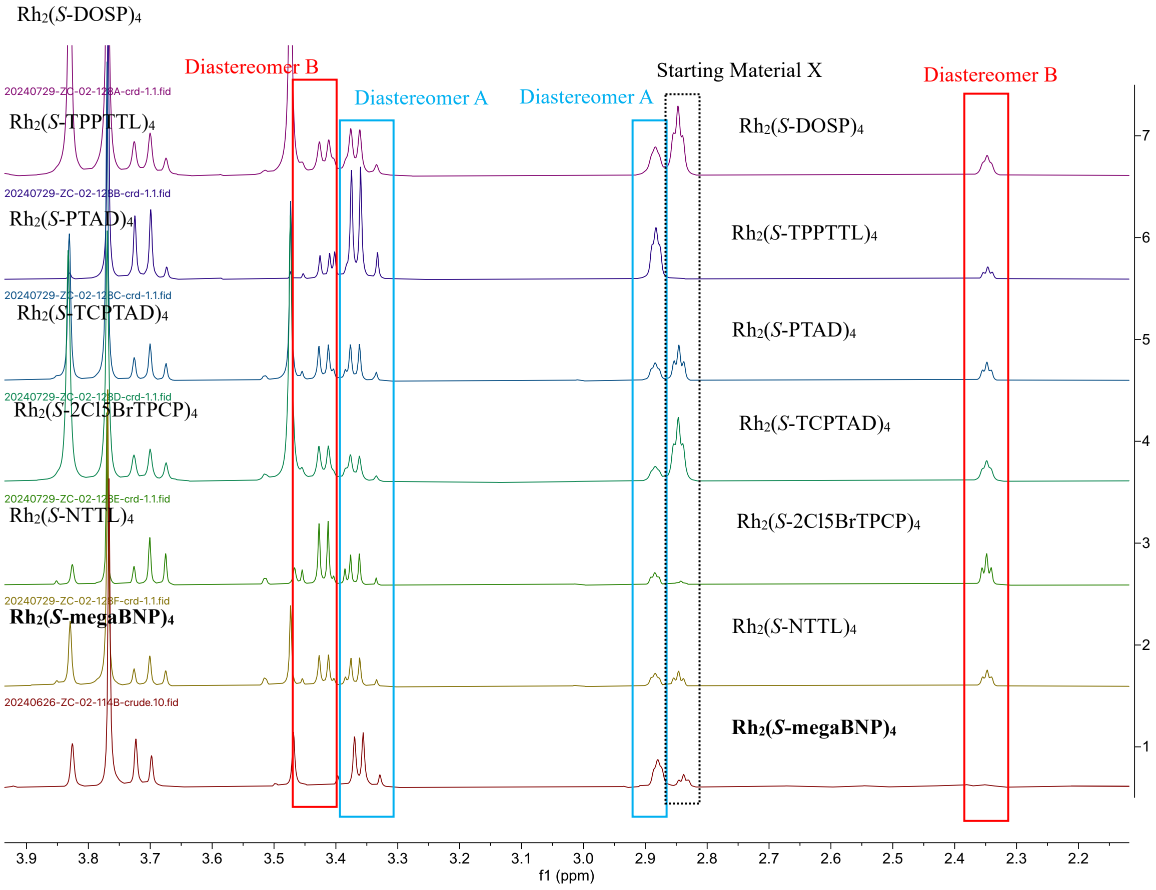

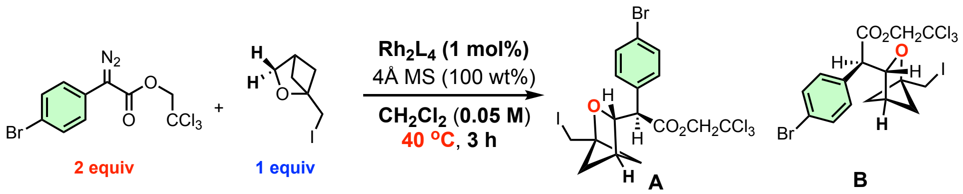

Entry	Catalysts	NMR Yield of **A** (%)	d.r. (A:B)	ee (%)
1[b]	**Rh_2(*S*-megaBNP)$_4$**	**43(40[c])**	**>20:1**	**90**
2[b]	Rh_2(*S*-megaBNP)$_4$ (**3 equiv diazo**)	50(45 iso. yield)	>20:1	90
3[c]	Rh_2(*S*-megaBNP)$_4$ (**p-Br-TFE diazo**)	43	>20:1	N.D.
4[d]	Rh_2(*S*-megaBNP)$_4$ (**p-Br-TBE diazo**)	50	>20:1	N.D
5[e]	Rh_2(*S*-megaBNP)$_4$ (**CH_2Cl_2, 0.1 M**)	40	>20:1	90
6[f]	Rh_2(*S*-megaBNP)$_4$ (**CH_2Cl_2, 0.03 M**)	40	>20:1	90
7[g]	Rh_2(*S*-megaBNP)$_4$ (**$CDCl_3$**)	37	>20:1	N.D
8[h]	Rh_2(*S*-megaBNP)$_4$ (**$ClCH_2CH_2Cl$**)	35	>20:1	N.D
9[i]	Rh_2(*S*-megaBNP)$_4$ (**CH_2Br_2**)	26	>20:1	N.D
10[j]	Rh_2(*S*-megaBNP)$_4$ (**Both slowly added in CH_2Cl_2**)	26	>20:1	N.D
11[k]	Rh_2(*S*-megaBNP)$_4$ (**Diazo/Trap=1/2**)	17	>20:1	N.D

a. 1 equiv substrate and 1 mol % catalyst were dissolved with 1 mL CH_2Cl_2 in a 4 mL vial. 2 equiv of diazo was dissolved in 1 mL CH_2Cl_2 and was added to the reaction vial via syringe pump over 3 h at 40 °C (**0.05 M**). NMR yields were obtained from crude ^{1}H NMR with trimethoxybenzene as internal standard
b. **3 equiv** of diazo was used for the reaction
c. **trifluoroethyl ester diazo** was used for the reaction
d. **tribromoethyl ester diazo** was used for the reaction
e. the reaction was performed at **0.1 M** concentration
f. the reaction was performed at **0.03 M** concentration
g. the reaction was perfomed in 2 mL **$CHCl_3$** at 40 °C (0.05 M)
h. the reaction was perfomed in 2 mL **$ClCH_2CH_2Cl$** at 40 °C (0.05 M)
i. the reaction was perfomed in 2 mL **CH_2Br_2** at 40 °C (0.05 M)
j. slow addition of **diazo in syringe A** and **trap in syringe B**
k. the reaction was perfomed with **2 equiv of trap** and **1 equiv of diazo**
N.D. (not determined)

Further optimization did not lead to significant improvement of yield of desired product. Using more diazo can slightly improve the yield but will also lead to significant more dimer formation, which is causing difficulty for product purification. Thus, we decided to use 2 equiv of diazo as the standard condition for the study.

4.3 C–H Functionalization of Bicyclo Compounds and Product Characterization

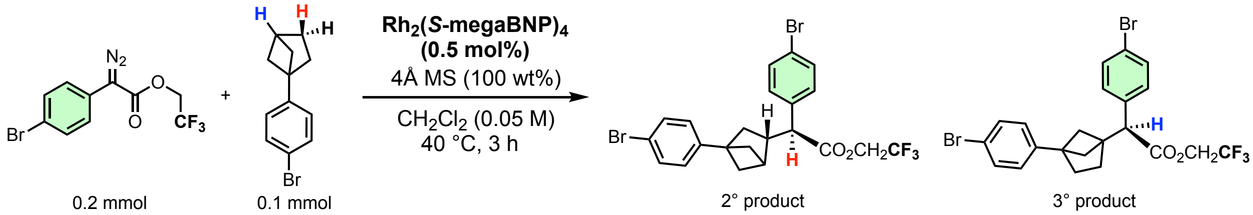

Using ***General Procedure C*** with 2,2,2-trifluoroethyl 2-(4-bromophenyl)-2-diazoacetate (**3b**, 0.2 mmol, 64.6 mg, 2 equiv) and 1-(4-bromophenyl)bicyclo[2.1.1]hexane (**4a**, 0.1 mmol, 23.7 mg, 1 equiv) and Rh_2(*S*-megaBNP)$_4$ (0.5 % mol, 1.7 mg). Combined secondary and tertiary product were obtained in 78% combined yield (41.5 mg). After preparative TLC separation (2% Diethyl ether/n-hexane), separated products were further characterized as below.

5b (**Secondary** C–H functionalization product) 2,2,2-trifluoroethyl (*S*)-2-(4-bromophenyl)-2-((*S*)-4-(4-bromophenyl)bicyclo[2.1.1]hexan-2-yl)acetate was obtained in 52% yield (27.7 mg) as a colorless liquid, >20:1 d.r., 99% ee.

$[\alpha]^{21}_D$: +7.7° (c = 0.23, $CHCl_3$, 99% ee)

^{1}H NMR (400 MHz, $CDCl_3$) δ 7.47 (d, J = 8.4 Hz, 2H), 7.37 (d, J = 8.4 Hz, 2H), 7.28 (d, J = 8.4 Hz, 2H), 7.02 (d, J = 8.4 Hz, 2H), 4.54 (dq, J = 12.7, 8.4 Hz, 1H), 4.39 (dq, J = 12.7, 8.4 Hz, 1H), 3.55 (d, J = 11.8 Hz, 1H), 3.02 – 2.93 (m, 1H), 2.46 (s, 1H), 1.92 (dt, J = 6.5, 2.9 Hz, 1H), 1.84 – 1.77 (m, 2H), 1.70 (dd, J = 9.8, 7.1 Hz, 1H), 1.59 (dd, J = 9.8, 6.5 Hz, 1H), 1.25 – 1.19 (m, 1H).

^{13}C NMR (101 MHz, $CDCl_3$) δ 171.8, 142.2, 136.0, 132.1, 131.4, 130.3, 127.7, 127.0, 122.0, 121.57 (q, J = 277.0 Hz), 120.1, 60.59 (q, J = 36.5 Hz), 54.5, 53.9, 44.8, 44.2, 39.9, 39.5, 38.2.

^{19}F NMR (376 MHz, $CDCl_3$) δ -73.71 (t, J = 8.4 Hz).

IR: 2968, 2930, 2921, 2873, 1755, 1489, 1413, 1395, 1299, 1209, 1168, 1099, 1052, 992 cm^{-1}

HRMS: (FTMS +p APCI) calcd for $C_{22}H_{19}O_2{}^{79}Br_2F_3$ $[M]^+$ 529.9698 found 529.9707(Δ=1.7 ppm).

HPLC: (ChiralPak AD-H, 1 mL/min, 1% isopropanol in hexane, λ=230 nm), retention times of 10.86 min (major) and 14.39 min (minor), 99% ee.

7b (**Tertiary** C–H functionalization product) 2,2,2-trifluoroethyl (*S*)-2-(4-bromophenyl)-2-(4-(4-bromophenyl)bicyclo[2.1.1]hexan-1-yl)acetate was obtained in 26% yield (13.8 mg) as a colorless liquid, 81% ee.

$[\alpha]^{21}_D$: -12.0° (c = 0.1, $CHCl_3$, 81% ee)

^{1}H NMR: (400 MHz, $CDCl_3$) δ 7.47 (d, J = 8.4 Hz, 2H), 7.39 (d, J = 8.4 Hz, 2H), 7.22 (d, J = 8.4 Hz, 2H), 7.04 (d, J = 8.4 Hz, 2H), 4.60 (dq, J = 12.7, 8.4 Hz, 1H), 4.40 (dq, J = 12.7, 8.4 Hz, 1H), 3.92 (s, 1H), 1.89 – 1.82 (m, 2H), 1.74 – 1.66 (m, 3H), 1.66 – 1.56 (m, 3H).

^{13}C NMR: (101 MHz, $CDCl_3$) δ 170.6, 142.5, 134.7, 131.8, 131.4, 130.5, 127.8, 123.01 (q, J = 277.9 Hz), 121.9, 120.1, 60.43 (q, J = 36.7 Hz), 54.0, 50.2, 49.6, 45.0, 44.8, 34.5, 31.2.

^{19}F NMR: (376 MHz, $CDCl_3$) δ -73.53 (t, J = 8.4 Hz).

IR: 2969, 2935, 2907, 2869, 1755, 1488, 1406, 1390, 1223, 1207, 1169, 1120, 1061, 1050, 981 cm^{-1}

HRMS: (FTMS +p APCI) calcd for $C_{22}H_{19}O_2{}^{79}Br_2F_3$ $[M]^+$ 529.9697 found 529.9707(Δ=1.9 ppm).

HPLC: (ChiralPak AD-H, 0.2 mL/min, 0.5% isopropanol in hexane, λ=230 nm), retention times of 69.81 min (major) and 74.19 min (minor), 81% ee.

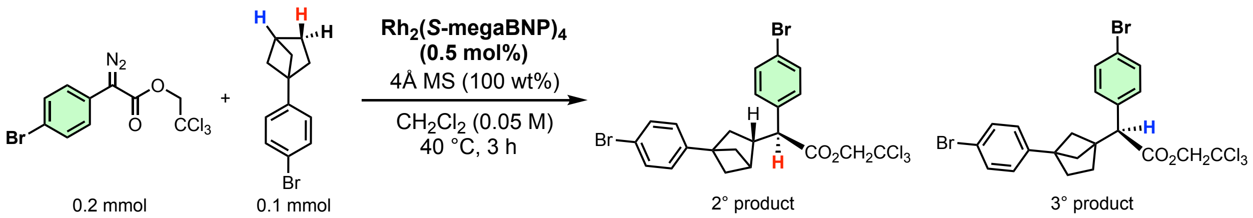

Using ***General Procedure C*** with 2,2,2-trichloroethyl 2-(4-bromophenyl)-2-diazoacetate (**3a**, 0.2 mmol, 74.8 mg, 2 equiv) and 1-(4-bromophenyl)bicyclo[2.1.1]hexane (**4a**, 0.1 mmol, 23.7 mg, 1 equiv) and Rh_2(*S*-megaBNP)$_4$ (0.5 % mol, 1.7 mg). Combined secondary and tertiary product were obtained in 89% combined yield (51.7 mg). After preparative TLC separation (2% Diethyl ether/n-hexane), separated products were further characterized as below.

5a (**Secondary** C–H functionalization product) 2,2,2-trichloroethyl (*S*)-2-(4-bromophenyl)-2-((*S*)-4-(4-bromophenyl)bicyclo[2.1.1]hexan-2-yl)acetate was obtained in 59% yield (34.3 mg) as a colorless liquid, >20:1 d.r., 99% ee.

$[\alpha]^{21}_{D}$: +37.2° (c = 2.1, $CHCl_3$, 99% ee)

^{1}H NMR (400 MHz, $CDCl_3$) δ 7.47 (d, *J* = 8.4 Hz, 2H), 7.37 (d, *J* = 8.4 Hz, 2H), 7.32 (d, *J* = 8.4 Hz, 2H), 7.03 (d, *J* = 8.4 Hz, 2H), 4.76 (d, *J* = 11.9 Hz, 1H), 4.71 (d, *J* = 11.9 Hz, 1H), 3.60 (d, J = 11.8 Hz, 1H), 3.06 – 3.00 (m, 1H), 2.57 – 2.53 (m, 1H), 1.92 (dt, J = 6.5, 2.9 Hz, 1H), 1.86 – 1.79 (m, 2H), 1.73 (dd, J = 9.7, 7.3 Hz, 1H), 1.60 (dd, J = 9.7, 6.5 Hz, 1H), 1.25 – 1.22 (m, 1H).

^{13}C NMR (151 MHz, $CDCl_3$) δ 171.6, 142.3, 136.1, 132.0, 131.4, 130.5, 127.7, 121.9, 120.1, 94.9, 74.2, 54.8, 53.9, 44.8, 43.9, 40.0, 39.6, 38.2.

IR: 2951, 2870, 1749, 1488, 1448, 1409, 1395, 1333, 1212, 1128, 1098, 1030, 988 cm^{-1}

HRMS: (FTMS +p APCI) calcd for $C_{22}H_{19}O_2{}^{79}Br_2{}^{35}Cl_3$ $[M]^+$ 577.8812 found 577.8823 (Δ=2.0 ppm).

HPLC: (ChiralPak AD-H, 1 mL/min, 1% isopropanol in hexane, λ=230 nm), retention times of 11.64 min (major) and 16.16 min (minor), 99% ee.

7a (**Tertiary** C–H functionalization product) 2,2,2-trichloroethyl (*S*)-2-(4-bromophenyl)-2-(4-(4-bromophenyl)bicyclo[2.1.1]hexan-1-yl)acetate was obtained in 30% yield (17.4 mg) as a colorless liquid, 93% ee.

$[\alpha]^{21}_{D}$: -3.7° (c = 0.1, $CHCl_3$, 93% ee)

^{1}H NMR (400 MHz, $CDCl_3$) δ 7.47 (d, *J* = 8.4 Hz, 2H), 7.39 (d, *J* = 8.4 Hz, 2H), 7.26 (d, *J* = 8.4 Hz, 2H), 7.04 (d, *J* = 8.4 Hz, 2H), 4.84 (d, *J* = 12.0 Hz, 1H), 4.69 (d, *J* = 12.0 Hz, 1H), 3.96 (s, 1H), 1.88 – 1.82 (m, 2H), 1.77 – 1.69 (m, 4H), 1.60 (t, *J* = 5.2 Hz, 2H).

^{13}C NMR (151 MHz, $CDCl_3$) δ 170.5, 142.5, 134.9, 131.7, 131.4, 130.7, 127.8, 121.8, 120.0, 94.8, 74.4, 54.4, 50.3, 49.6, 45.2, 44.9, 34.5, 31.4.

IR: 2953, 2871, 1748, 1487, 1448, 1412, 1390, 1300, 1252, 1130, 1054, 1032, 968 cm^{-1}

HRMS: (FTMS +p APCI) calcd for $C_{22}H_{19}O_2{}^{79}Br_2{}^{35}Cl_3$ $[M]^+$ 577.8812 found 577.8820 (Δ=1.4 ppm).

HPLC: (Regis (S,S) Whelk-O 1, 1 mL/min, 1% isopropanol in hexane, λ=230 nm), retention times of 14.21 min (minor) and 17.02 min (major), 93% ee.

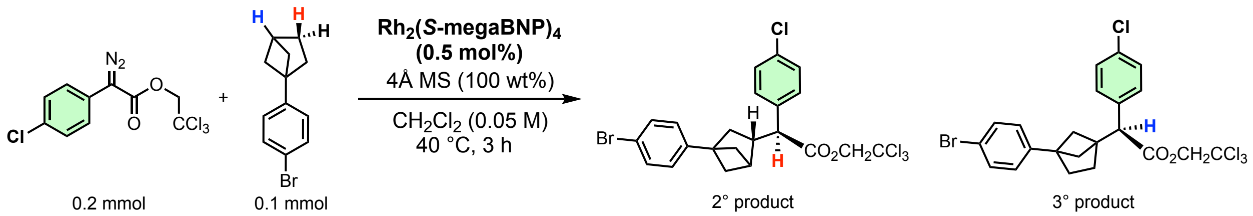

Using ***General Procedure C*** with 2,2,2-trichloroethyl 2-(4-chlorophenyl)-2-diazoacetate (**3c**, 0.2 mmol, 65.6 mg, 2 equiv) and 1-(4-bromophenyl)bicyclo[2.1.1]hexane (**4a**, 0.1 mmol, 23.7 mg, 1 equiv) and Rh_2(*S*-megaBNP)$_4$ (0.5 % mol, 1.7 mg). Combined secondary and tertiary product were obtained in 82% combined yield (44.0 mg). After preparative TLC separation (2% Diethyl ether/n-hexane), separated products were further characterized as below.

5c (**Secondary** C–H functionalization product) 2,2,2-trichloroethyl (S)-2-((S)-4-(4-bromophenyl)bicyclo[2.1.1]hexan-2-yl)-2-(4-chlorophenyl)acetate was obtained in 56% yield (30.1 mg) as a colorless liquid, 19:1 d.r., 99% ee.

$[\alpha]^{21}_{D}$: +7.6° (c = 0.26, $CHCl_3$, 99% ee)

^{1}H NMR: (400 MHz, $CDCl_3$) δ 7.41 – 7.35 (m, 4H), 7.31 (d, *J* = 8.5 Hz, 2H), 7.03 (d, *J* = 8.5 Hz, 2H), 4.75 (d, *J* = 12.0 Hz, 1H), 4.71 (d, *J* = 12.0 Hz, 1H), 3.60 (d, *J* = 11.8 Hz, 1H), 3.09 – 3.00 (m, 1H), 2.54 (td, *J* = 2.9, 1.1 Hz, 1H), 1.91 (dt, *J* = 6.5, 2.9 Hz, 1H), 1.86 – 1.79 (m, 2H), 1.73 (dd, *J* = 9.7, 7.1 Hz, 1H), 1.60 (dd, *J* = 9.7, 6.5 Hz, 1H), 1.25 – 1.20 (m, 1H).

^{13}C NMR (151 MHz, $CDCl_3$) δ 171.7, 142.3, 135.6, 133.8, 131.4, 130.1, 129.0, 127.7, 120.1, 94.9, 74.2, 54.7, 54.0, 44.8, 44.0, 40.0, 39.6, 38.2.

IR: 2965, 2955, 2874, 1750, 1491, 1373, 1287, 1266, 1161, 1091, 1058, 1029, 1010, 904 cm^{-1}

HRMS: (FTMS +p APCI) calcd for $C_{22}H_{19}O_2{}^{79}Br_2{}^{35}Cl_3F_3$ $[M+H]^+$ 534.9395 found 534.9402 (Δ=1.3 ppm).

HPLC: (ChiralPak AD-H, 1 mL/min, 1% isopropanol in hexane, λ=230 nm), retention times of 11.16 min (major) and 16.93 min (minor), 99% ee.

7c (**Tertiary** C–H functionalization product) 2,2,2-trichloroethyl (S)-2-(4-(4-bromophenyl)bicyclo[2.1.1]hexan-1-yl)-2-(4-chlorophenyl)acetate was obtained in 26% yield (13.9 mg) as a colorless liquid, 88% ee.

$[\alpha]^{21}_{D}$: -10.2° (c = 0.1, $CHCl_3$, 88% ee)

^{1}H NMR (400 MHz, $CDCl_3$) δ 7.39 (d, *J* = 8.5 Hz, 2H), 7.32 (s, 4H), 7.04 (d, *J* = 8.5 Hz, 2H), 4.84 (d, *J* = 12.0 Hz, 1H), 4.69 (d, *J* = 12.0 Hz, 1H), 3.97 (s, 1H), 1.89 – 1.83 (m, 2H), 1.77 – 1.69 (m, 4H), 1.60 (t, *J* = 5.3 Hz, 2H).

^{13}C NMR (101 MHz, $CDCl_3$) δ 170.5, 142.4, 134.2, 133.5, 131.3, 130.2, 128.6, 127.6, 119.9, 94.7, 74.3, 54.2, 50.2, 49.6, 45.0, 44.8, 34.4, 31.2.

IR: 2964, 2949, 2877, 1750, 1487, 1365, 1322, 1267, 1162, 1129, 1042, 1017, 1010, 967 cm^{-1}

HRMS: (FTMS +p APCI) calcd for $C_{22}H_{19}O_2{}^{79}Br_2{}^{35}Cl_3F_3$ $[M+H]^+$ 534.9395 found 534.9403 (Δ=1.5 ppm).

HPLC: (ChiralPak AS-H, 0.5 mL/min, 0.5% isopropanol in hexane, λ=230 nm), retention times of 15.05 min (minor) and 16.98 min (major), 88% ee.

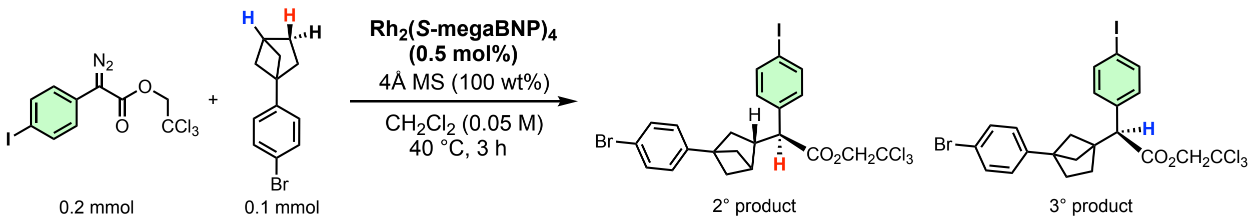

Using ***General Procedure C*** with 2,2,2-trichloroethyl 2-(4-iodophenyl)-2-diazoacetate (**3d**, 0.2 mmol, 83.9 mg, 2 equiv) and 1-(4-bromophenyl)bicyclo[2.1.1]hexane (**4a**, 0.1 mmol, 23.7 mg, 1 equiv) and Rh_2(*S*-megaBNP)$_4$ (0.5 % mol, 1.7 mg). Combined secondary and tertiary product were obtained in 99% combined yield (62.2 mg). After preparative TLC separation (2% Diethyl ether/n-hexane), separated products were further characterized as below.

5d (**Secondary** C–H functionalization product) 2,2,2-trichloroethyl (S)-2-((S)-4-(4-bromophenyl)bicyclo[2.1.1]hexan-2-yl)-2-(4-iodophenyl)acetate was obtained in 67% yield (41.7 mg) as a white solid, >20:1 d.r., 99% ee.

$[\alpha]^{21}_D$: +7.3° (c = 0.27, $CHCl_3$, 99% ee)

^{1}H NMR (400 MHz, $CDCl_3$) δ 7.67 (d, J = 8.5 Hz, 2H), 7.37 (d, J = 8.5 Hz, 2H), 7.19 (d, J = 8.4 Hz, 2H), 7.03 (d, J = 8.4 Hz, 2H), 4.76 (d, J = 12.0 Hz, 1H), 4.70 (d, J = 12.0 Hz, 1H), 3.57 (d, J = 11.8 Hz, 1H), 3.07 – 2.97 (m, 1H), 2.54 (td, J = 2.9, 1.2 Hz, 1H), 1.91 (dt, J = 6.4, 2.9 Hz, 1H), 1.86 – 1.78 (m, 2H), 1.73 (dd, J = 9.7, 7.1 Hz, 1H), 1.59 (dd, J = 9.7, 6.4 Hz, 1H), 1.27 – 1.20 (m, 1H).

^{13}C NMR (151 MHz, $CDCl_3$) δ 171.6, 142.3, 138.0, 136.8, 131.4, 130.7, 127.7, 120.1, 94.9, 93.5, 74.2, 54.9, 54.0, 44.8, 43.9, 40.0, 39.6, 38.2.

IR: 2954, 2870, 1749, 1484, 1447, 1371, 1304, 1264, 1212, 1180, 1098, 1007, 957 cm^{-1}

HRMS: (FTMS +p APCI) calcd for $C_{22}H_{20}O_2{}^{79}Br^{35}Cl_3{}^{127}I$ $[M+H]^+$ 626.8763 found 626.8752 (Δ=1.8 ppm).

HPLC: (ChiralPak AD-H, 1 mL/min, 1% isopropanol in hexane, λ=230 nm), retention times of 11.78 min (major) and 16.30 min (minor), 99% ee.

7d (**Tertiary** C–H functionalization product) 2,2,2-trichloroethyl (S)-2-(4-(4-bromophenyl)bicyclo[2.1.1]hexan-1-yl)-2-(4-iodophenyl)acetate was obtained in 32% yield (19.9 mg) as a white solid, 96% ee.

$[\alpha]^{21}_D$: -2.8° (c = 0.18, $CHCl_3$, 96% ee)

^{1}H NMR (400 MHz, $CDCl_3$) δ 7.67 (d, *J* = 8.3 Hz, 2H), 7.39 (d, *J* = 8.3 Hz, 2H), 7.13 (d, *J* = 8.3 Hz, 2H), 7.04 (d, *J* = 8.3 Hz, 2H), 4.84 (d, *J* = 12.0 Hz, 1H), 4.68 (d, *J* = 12.0 Hz, 1H), 3.94 (s, 1H), 1.89 – 1.82 (m, 2H), 1.78 – 1.68 (m, 4H), 1.64 – 1.57 (m, 2H).

^{13}C NMR (151 MHz, $CDCl_3$) δ 170.5, 142.5, 137.7, 135.6, 131.4, 130.9, 127.8, 120.0, 94.8, 93.4, 74.4, 54.5, 50.3, 49.6, 45.2, 44.9, 34.6, 31.4.

IR: 2955, 2868, 1750, 1489, 1420, 1374, 1298, 1265, 1201, 1179, 1026, 1005, 989, 904 cm^{-1}

HRMS: (FTMS +p APCI) calcd for $C_{22}H_{20}O_2{}^{79}Br^{35}Cl_3{}^{127}I$ $[M+H]^+$ 626.8763 found 626.8753 (Δ=1.6 ppm).

HPLC: (Regis (S,S) Whelk-O 1, 1 mL/min, 1% isopropanol in hexane, λ=230 nm), retention times of 16.34 min (minor) and 19.42 min (major), 96% ee.

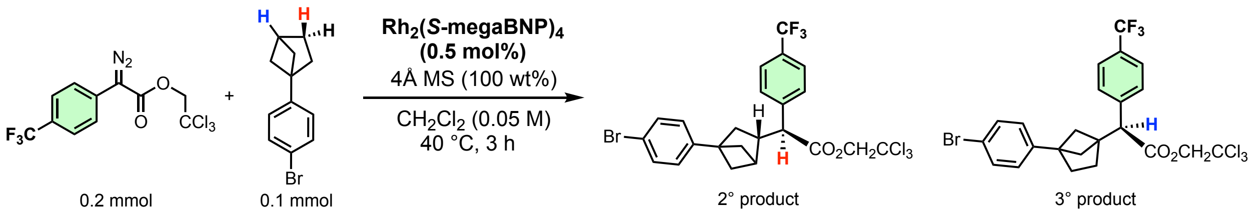

Using ***General Procedure C*** with 2,2,2-trichloroethyl 2-(4-trifluoromethyl)-2-diazoacetate (**3e**, 0.2 mmol, 73.0 mg, 2 equiv) and 1-(4-bromophenyl)bicyclo[2.1.1]hexane (**4a**, 0.1 mmol, 23.7 mg, 1 equiv) and Rh_2(*S*-megaBNP)$_4$ (0.5 % mol, 1.7 mg). Combined secondary and tertiary product were obtained in 92% combined yield (52.5 mg). After preparative TLC separation (2% Diethyl ether/n-hexane), separated products were further characterized as below.

5e (**Secondary** C–H functionalization product) 2,2,2-trichloroethyl (S)-2-((S)-4-(4-bromophenyl)bicyclo[2.1.1]hexan-2-yl)-2-(4- trifluoromethyl)acetate was obtained in 54% yield (30.6 mg) as a colorless liquid, 10:1 d.r., 99% ee.

$[\alpha]^{21}_{D}$: +2.9° (c = 0.3, $CHCl_3$, 99% ee)

^{1}H NMR (400 MHz, $CDCl_3$) δ 7.61 (d, J = 8.5 Hz, 2H), 7.57 (d, J = 8.5 Hz, 2H), 7.37 (d, J = 8.4 Hz, 1H), 7.03 (d, J = 8.4 Hz, 1H), 4.77 (d, J = 11.9 Hz, 1H), 4.71 (d, J = 11.9 Hz, 1H), 3.71 (d, J = 11.8 Hz, 1H), 3.15 – 3.04 (m, 1H), 2.62 – 2.56 (m, 1H), 1.93 (dt, J = 6.5, 2.9 Hz, 1H), 1.88 – 1.80 (m, 2H), 1.76 (dd, J = 9.7, 7.2 Hz, 1H), 1.61 (dd, J = 9.7, 6.5 Hz, 1H), 1.27 – 1.20 (m, 1H).

^{13}C NMR (151 MHz, $CDCl_3$) δ 171.4, 142.2, 141.1, 131.4, 130.2 (q, $^2J_{C\text{-}F}$ = 32.6 Hz), 129.2, 127.7, 125.8 (q, $^3J_{C\text{-}F}$ = 3.7 Hz), 124.14 (q, $^1J_{C\text{-}F}$= 272.0 Hz), 120.1, 94.8, 74.3, 55.2, 54.0, 44.8, 44.0, 40.0, 39.5, 38.3.

^{19}F NMR (565 MHz, $CDCl_3$) δ -62.59.

IR: 2955, 2873, 1750, 1619, 1494, 1421, 1395, 1323, 1208, 1163, 1068, 1011, 955 cm^{-1}

HRMS: (FTMS +p APCI) calcd for $C_{23}H_{20}O_2{}^{79}Br_2{}^{35}Cl_3F_3$ $[M+H]^+$ 568.9659 found 568.9668 (Δ=1.5 ppm).

HPLC: (ChiralPak AD-H, 1 mL/min, 1% isopropanol in hexane, λ=230 nm), retention times of 9.57 min (major) and 12.85 min (minor), 99% ee.

7e (**Tertiary** C–H functionalization product) 2,2,2-trichloroethyl (S)-2-(4-(4-bromophenyl)bicyclo[2.1.1]hexan-1-yl)-2-(4- trifluoromethyl)acetate was obtained in 38% yield (21.9 mg) as a colorless liquid, 90% ee.

$[\alpha]^{21}_{D}$: -7.8° (c = 0.1, $CHCl_3$, 90% ee)

^{1}H NMR (400 MHz, $CDCl_3$) δ 7.61 (d, J = 8.3 Hz, 2H), 7.52 (d, J = 8.3 Hz, 2H), 7.39 (d, J = 8.4 Hz, 2H), 7.04 (d, J = 8.4 Hz, 2H), 4.86 (d, J = 12.0 Hz, 1H), 4.70 (d, J = 12.0 Hz, 1H), 4.08 (s, 1H), 1.91 – 1.83 (m, 2H), 1.80 – 1.71 (m, 4H), 1.67 – 1.59 (m, 2H).

^{13}C NMR (151 MHz, CDCl3) δ 170.3, 142.4, 139.8, 131.4, 130.03 (q, $^{2}J_{C\text{-}F}$ = 32.5 Hz), 129.4, 127.8, 125.5 (q, $^{3}J_{C\text{-}F}$ = 3.7 Hz), 124.2 (q, $^{1}J_{C\text{-}F}$ = 272.0 Hz), 120.1, 94.8, 74.5, 54.8, 50.3, 49.6, 45.2, 45.0, 34.5, 31.4.

^{19}F NMR (565 MHz, $CDCl_3$) δ -62.55.

IR: 2956, 2869, 1751, 1623, 1488, 1426, 1373, 1273, 1209, 1124, 1072, 1029, 1019, 987 cm^{-1}

HRMS: (FTMS +p APCI) calcd for $C_{23}H_{20}O_2{}^{79}Br_2{}^{35}Cl_3F_3$ $[M+H]^+$ 568.9659 found 568.9667 (Δ=1.4 ppm).

HPLC: (ChiralPak AS-H, 0.25 mL/min, 0.5% isopropanol in hexane, λ=230 nm), retention times of 23.94 min (minor) and 27.38 min (major), 90% ee.

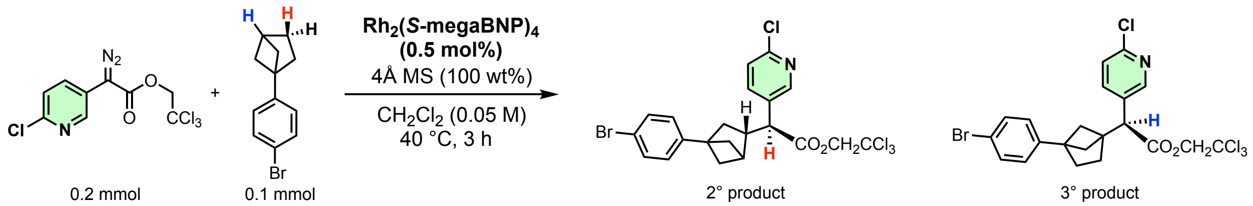

Using ***General Procedure C*** with 2,2,2-trichloroethyl 2-(6-chloropyridin-3-yl)-2-diazoacetate (**3f**, 0.2 mmol, 65.8 mg, 2 equiv) and 1-(4-bromophenyl)bicyclo[2.1.1]hexane (**4a**, 0.1 mmol, 23.7 mg, 1 equiv) and Rh_2(*S*-megaBNP)$_4$ (0.5 % mol, 1.7 mg). Combined secondary and tertiary product were obtained in 40% combined yield (21.5 mg).

Note that the separation of regioisomers for this reaction was unsuccessful, thus the NMR, IR and HRMS were characterized as a mixture of **5f** (secondary product) and **7f** (tertiary product). Specifically, HSQC and HMBC were used to fully assign NMR signals.

^{1}H NMR (**5f** and **7f** mixture):

5f (600 MHz, $CDCl_3$) δ 8.43 (d, *J* = 2.6 Hz, 1H), 7.80 (dd, *J* = 8.4, 2.6 Hz, 1H), 7.38 (d, *J* = 8.6 Hz, 2H), 7.34 (d, *J* = 8.4 Hz, 1H), 7.02 (d, *J* = 8.6 Hz, 2H), 4.76 (d, *J* = 12.0 Hz, 1H), 4.74 (d, *J* = 12.0 Hz, 1H), 3.65 (d, *J* = 11.8 Hz, 1H), 3.05 – 2.98 (m, 1H), 2.59 (td, *J* = 2.9, 1.2 Hz, 1H), 1.94 (dt, *J* = 6.7, 2.9 Hz, 1H), 1.87 – 1.83 (m, 2H), 1.77 – 1.72 (m, 1H), 1.62 – 1.60 (m, 1H), 1.22 (ddd, *J* = 11.4, 4.1, 2.9 Hz, 1H).

7f (600 MHz, $CDCl_3$) δ 8.36 (d, *J* = 2.5 Hz, 1H), 7.80 (dd, *J* = 8.3, 2.5 Hz, 1H), 7.40 (d, *J* = 8.5 Hz, 2H), 7.33 (d, *J* = 8.3 Hz, 1H), 7.04 (d, *J* = 8.5 Hz, 2H), 4.84 (d, *J* = 12.0 Hz, 1H), 4.73 (d, *J* = 12.0 Hz, 1H), 4.03 (s, 1H), 1.89 – 1.86 (m, 2H), 1.79 – 1.70 (m, 4H), 1.65 – 1.61 (m, 2H).

^{13}C NMR (**5f** and **7f** mixture):

5f (151 MHz, $CDCl_3$) δ 170.9, 151.2, 150.0, 141.9, 138.8, 131.9, 131.4, 127.7, 124.6, 120.2, 94.7, 74.4, 54.0, 52.2, 44.7, 44.1, 40.0, 39.5, 38.4.

7f (151 MHz, $CDCl_3$) δ 169.9, 151.1, 150.0, 142.1, 139.0, 131.5, 130.6, 127.7, 124.2, 120.2, 94.6, 74.6, 51.8, 50.3, 49.6, 45.2, 45.0, 34.4, 31.1.

IR: 2965, 2873, 1750, 1584, 1564, 1492, 1461, 1394, 1324, 1288, 1265, 1212, 1139, 1022, 989, 932 cm^{-1}

HRMS: (FTMS +p APCI) calcd for $C_{21}H_{19}O_2N^{79}Br^{35}Cl_4$ $[M+H]^+$ 535.9348 found 535.9352 (Δ=0.8 ppm).

Due to the difficulty of separating all four stereoisomers, partially separated chiral regioisomers were tested for HPLC and optical rotation separately.

5f (**Secondary** C–H functionalization product) 2,2,2-trichloroethyl (*S*)-2-((*S*)-4-(4-bromophenyl)bicyclo[2.1.1]hexan-2-yl)-2-(6-chloropyridin-3-yl)acetate was obtained as a colorless liquid, 14:1 d.r., 99% ee.

$[\alpha]^{21}_{D}$: +17.2° (c = 0.61, $CHCl_3$, 99% ee)

HPLC: (ChiralPak AS-H, 1 mL/min, 5% isopropanol in hexane, λ=230 nm), retention times of 10.96 min (minor) and 14.72 min (major), 99% ee.

7f (**Tertiary** C–H functionalization product) 2,2,2-trichloroethyl (*S*)-2-(4-(4-bromophenyl)bicyclo[2.1.1]hexan-1-yl)-2-(6-chloropyridin-3-yl)acetate was obtained as a colorless liquid, 87% ee.

$[\alpha]^{21}_{D}$: -21.2° (c = 0.1, $CHCl_3$, 87% ee)

HPLC: (ChiralPak AS-H, 1 mL/min, 5% isopropanol in hexane, λ=230 nm), retention times of 7.99 min (minor) and 11.18 min (major), 87% ee.

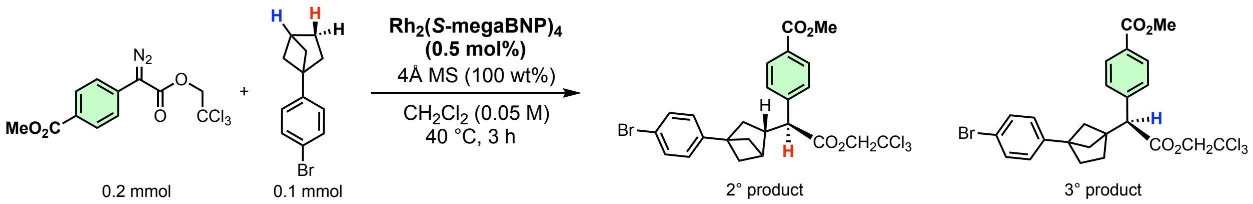

Using ***General Procedure C*** with methyl 4-(1-diazo-2-oxo-2-(2,2,2-trichloroethoxy)ethyl)benzoate (**3g**, 0.2 mmol, 70.3 mg, 2 equiv) and 1-(4-bromophenyl)bicyclo[2.1.1]hexane (**4a**, 0.1 mmol, 23.7 mg, 1 equiv) and Rh_2(*S*-megaBNP)$_4$ (0.5 % mol, 1.7 mg). Combined secondary product and tertiary product were obtained in combined 58% combined yield (32.5 mg) as a colorless liquid.

Note that the separation of regioisomers for this reaction failed in our attempt so that the NMR, IR, HRMS and Chiral SFC were characterized as a mixture. Learning from the diazo scope, secondary product is more likely to have higher enantioselectivity than tertiary product. Thus, we tentatively assign the secondary product with the higher enantioselectivity result and the tertiary product with the relatively lower enantioselectivity from this specific chiral SFC analysis.

Racemic SFC: (ChiralCel OJ-3, 2.5 mL/min, 10% methanol in isopropanol with 0.2% formic acid, λ=230 nm), retention times of 4.89 min and 5.23 min for tertiary functionalization product **5g**; 5.48 min and 6.99 min for secondary functionalization product **7g**.

Chiral SFC: (ChiralCel OJ-3, 2.5 mL/min, 10% methanol in isopropanol with 0.2% formic acid, λ=230 nm), retention times of 4.76 min (major), 5.18 min (minor), 90% ee (**5g**); 5.48 min (major) and the minor enantiomer is not detected, 99% ee (**7g**).

5g (**Secondary** C–H functionalization product) methyl 4-((*S*)-1-((*S*)-4-(4-bromophenyl)bicyclo[2.1.1]hexan-2-yl)-2-oxo-2-(2,2,2-trichloroethoxy)ethyl)benzoate was obtained in 9:1 d.r. (from crude ^{1}H NMR), 99% ee.

Chiral SFC: (ChiralCel OJ-3, 2.5 mL/min, 10% (50% methanol in isopropanol with 0.2% Formic Acid), λ=230 nm), retention times of 5.48 min (major) and minor enantiomer is not detected, >99% ee.

7g (**Tertiary** C–H functionalization product) 2,2,2-trichloroethyl methyl (*S*)-4-(1-(4-(4-bromophenyl)bicyclo[2.1.1]hexan-1-yl)-2-oxo-2-(2,2,2-trichloroethoxy)ethyl)benzoate was obtained in 90% ee.

Chiral SFC: (ChiralCel OJ-3, 2.5 mL/min, 10% (50% methanol in isopropanol with 0.2% Formic Acid), λ=230 nm), retention times of 4.76 min (major) and 5.18 min (minor), 90% ee.

^{1}H NMR (secondary product **5g** and tertiary product **7g** as a mixture):

5g (600 MHz, $CDCl_3$) δ 8.02 (d, *J* = 8.3 Hz, 2H), 7.52 (d, *J* = 8.3 Hz, 2H), 7.37 (d, *J* = 8.6 Hz, 2H), 7.02 (d, *J* = 8.6 Hz, 2H), 4.75 (d, *J* = 12.0 Hz, 1H), 4.72 (d, *J* = 12.0 Hz, 1H), 3.91 (s, 3H), 3.70 (d, *J* = 11.8 Hz, 1H), 3.13 – 3.06 (m, 1H), 2.58 (td, *J* = 2.8, 1.2 Hz, 1H), 1.94 – 1.91 (m, 1H), 1.84 – 1.79 (m, 2H), 1.77 – 1.73 (m, 1H), 1.63 – 1.60 (m, 1H), 1.23 (ddd, *J* = 11.3, 4.2, 3.0 Hz, 1H).

7g (600 MHz, $CDCl_3$) δ 8.02 (d, *J* = 8.3 Hz, 2H), 7.46 (d, *J* = 8.3 Hz, 2H), 7.38 (d, *J* = 8.0 Hz, 2H), 7.04 (d, *J* = 8z.0 Hz, 2H), 4.84 (d, *J* = 12.0 Hz, 1H), 4.71 (d, *J* = 12.0 Hz, 1H), 4.07 (s, 1H), 3.92 (s, 3H), 1.88 – 1.83 (m, 2H), 1.78 – 1.72 (m, 4H), 1.63 – 1.59 (m, 2H).

^{13}C NMR (secondary product **5g** and tertiary product **7g** as a mixture):

5g (151 MHz, $CDCl_3$) δ 171.4, 166.8, 142.23, 142.18, 131.36, 130.1, 129.78, 128.9, 127.7, 120.1, 94.9, 74.2, 55.4, 54.0, 52.3 (overlap), 44.7, 43.9, 40.0, 39.6, 38.2.

7g (151 MHz, $CDCl_3$) δ 170.4, 166.9, 142.5, 141.0, 131.37, 129.84, 129.6, 129.0, 127.8, 120.0, 94.8, 74.4, 55.0, 52.3 (overlap), 50.3, 49.6, 45.2, 45.0, 34.5, 31.4.

IR (**5g** and **7g**): 2952, 2872, 1750, 1720, 1611, 1492, 1418, 1373, 1310, 1208, 1130, 1073, 1009, 988 cm^{-1}

HRMS (**5g** and **7g**): (FTMS +p APCI) calcd for $C_{24}H_{23}O_4{}^{79}Br^{35}Cl_3$ $[M+H]^+$ 558.9840 found 558.9843 (Δ=0.6 ppm).

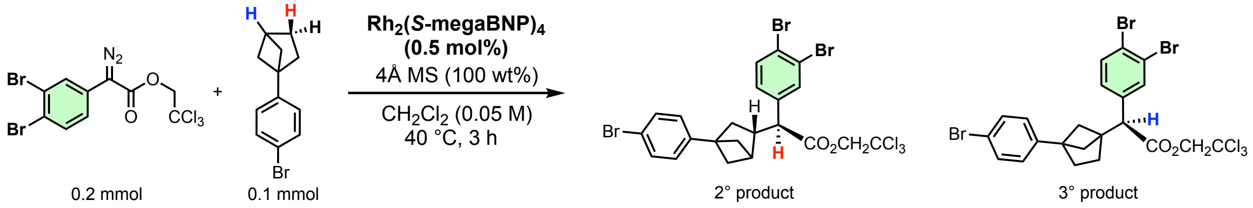

Using ***General Procedure C*** with 2,2,2-trichloroethyl 2-diazo-2-(3,4-dibromophenyl)acetate (**3h**, 0.2 mmol, 90.3 mg, 2 equiv) and 1-(4-bromophenyl)bicyclo[2.1.1]hexane (**4a**, 0.1 mmol, 23.7 mg, 1 equiv) and Rh_2(*S*-megaBNP)$_4$ (0.5 % mol, 1.7 mg). Combined secondary and tertiary product were obtained in 75% combined yield (45.3 mg). After preparative TLC separation (2% Diethyl ether/n-hexane), separated products were further characterized as below.

5h (**Secondary** C–H functionalization product) 2,2,2-trichloroethyl (*S*)-2-((*S*)-4-(4-bromophenyl)bicyclo[2.1.1]hexan-2-yl)-2-(3,4-dibromophenyl)acetate was obtained in 42% yield (25.6 mg) as a colorless liquid, 10:1 d.r., 98% ee.

$[\alpha]^{21}_{D}$: +28.3° (c = 0.97, $CHCl_3$, 98% ee)

^{1}H NMR (400 MHz, $CDCl_3$) δ 7.73 (d, *J* = 2.1 Hz, 1H), 7.59 (d, *J* = 8.4 Hz, 1H), 7.38 (d, *J* = 8.4 Hz, 2H), 7.24 (d, *J* = 2.1 Hz, 1H), 7.03 (d, *J* = 8.4 Hz, 2H), 4.77 (d, *J* = 11.9 Hz, 1H), 4.71 (d, *J* = 11.9 Hz, 1H), 3.57 (d, *J* = 11.8 Hz, 1H), 3.06 – 2.96 (m, 1H), 2.55 (td, *J* = 2.9, 1.2 Hz, 1H), 1.92 (dt, *J* = 6.5, 2.9 Hz, 1H), 1.90 – 1.80 (m, 2H), 1.72 (dd, *J* = 9.8, 7.2 Hz, 1H), 1.60 (dd, *J* = 9.8, 6.5 Hz, 1H), 1.23 (ddd, *J* = 11.1, 4.2, 2.9 Hz, 1H).

^{13}C NMR (151 MHz, CDCl3) δ 171.1, 142.1, 138.0, 134.0, 133.9, 131.4, 129.0, 127.7, 125.3, 124.3, 120.2, 94.8, 74.3, 54.5, 54.0, 44.8, 44.0, 40.0, 39.5, 38.3.

IR: 2953, 2872, 1750, 1555, 1491, 1462, 1395, 1335, 1263, 1185, 1113, 1072, 1029, 934 cm^{-1}

HRMS (FTMS +p APCI) calcd for $C_{22}H_{19}O_2{}^{79}Br_3{}^{35}Cl_3$ $[M+H]^+$ 656.7995 found 656.8006 (Δ=1.6 ppm).

HPLC: (Regis (S,S) Whelk O - 1, 1 mL/min, 1% isopropanol in hexane, λ=230 nm), retention times of 15.02 min (major) and 18.98 min (minor), 98% ee.

7h (**Tertiary** C–H functionalization product) 2,2,2-trichloroethyl (*S*)-2-(4-(4-bromophenyl)bicyclo[2.1.1]hexan-1-yl)-2-(3,4-dibromophenyl)acetate was obtained in 33% yield (16.7 mg) as a colorless liquid, 90% ee.

$[\alpha]^{21}_{D}$: -9.2° (c = 0.73, $CHCl_3$, 90% ee)

^{1}H NMR (400 MHz, $CDCl_3$) δ 7.67 (d, *J* = 2.2 Hz, 1H), 7.59 (d, *J* = 8.3 Hz, 1H), 7.40 (d, *J* = 8.5 Hz, 2H), 7.20 (dd, *J* = 8.3, 2.2 Hz, 1H), 7.04 (d, *J* = 8.5 Hz, 2H), 4.85 (d, *J* = 12.0 Hz, 1H), 4.70 (d, *J* = 12.0 Hz, 1H), 3.94 (s, 1H), 1.91 – 1.83 (m, 2H), 1.78 – 1.68 (m, 4H), 1.66 – 1.58 (m, 2H).

^{13}C NMR (151 MHz, $CDCl_3$) δ 170.1, 142.3, 136.7, 134.1, 133.7, 131.4, 129.2, 127.8, 125.0, 124.2, 120.1, 94.7, 74.5, 54.1, 50.3, 49.5, 45.2, 44.9, 34.5, 31.3.

IR: 2955, 2871, 1751, 1554, 1491, 1439, 1390, 1324, 1261, 1133, 1113, 1072, 1031 cm^{-1}

HRMS (FTMS +p APCI) calcd for $C_{22}H_{19}O_2{}^{79}Br_3{}^{35}Cl_3$ $[M+H]^+$ 656.7995 found 656.8005 (Δ=1.5 ppm).

HPLC: (Regis (S,S) Whelk O - 1, 1 mL/min, 1% isopropanol in hexane, λ=230 nm), retention times of 17.06 min (minor) and 22.69 min (major), 90% ee.

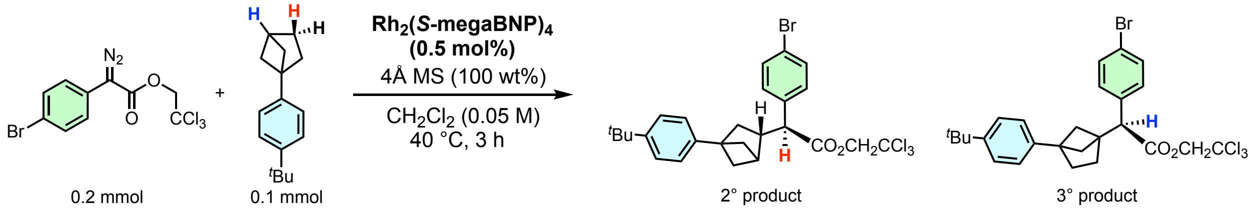

Using ***General Procedure C*** with 2,2,2-trichloroethyl 2-diazo-2-(4-dibromophenyl)acetate (**3a**, 0.2 mmol, 74.5 mg, 2 equiv) and 1-(4-(*tert*-butyl)phenyl)bicyclo[2.1.1]hexane (**4b**, 0.1 mmol, 21.4 mg, 1 equiv) and Rh_2(*S*-megaBNP)$_4$ (0.5 % mol, 1.7 mg). Combined secondary and tertiary product were obtained in 80% yield (44.7 mg). After preparative TLC separation (1% Diethyl ether/n-hexane), separated products were further characterized as below.

5i (**Secondary** C–H functionalization product) 2,2,2-trichloroethyl (*S*)-2-(4-bromophenyl)-2-((*S*)-4-(4-(*tert*-butyl)phenyl)bicyclo[2.1.1]hexan-2-yl)acetate was obtained in 44% yield (24.4 mg) as a colorless liquid, >20:1 d.r., 99% ee.

$[\alpha]^{21}_{D}$: +3.4° (c = 0.27, $CHCl_3$, 99% ee)

^{1}H NMR (400 MHz, $CDCl_3$) δ 7.46 (d, *J* = 8.6 Hz, 2H), 7.34 – 7.25 (m, 4H), 7.11 (d, *J* = 8.6 Hz, 2H), 4.76 (d, *J* = 12.0 Hz, 1H), 4.71 (d, *J* = 12.0 Hz, 1H), 3.60 (d, *J* = 11.7 Hz, 1H), 3.08 – 2.95 (m, 1H), 2.57 – 2.49 (m, 1H), 1.94 (dt, *J* = 6.7, 3.0 Hz, 1H), 1.84 (ddt, *J* = 9.9, 7.4, 4.1 Hz, 2H), 1.72 (dd, *J* = 9.9, 6.7 Hz, 1H), 1.62 – 1.57 (m, 1H), 1.29 (s, 9H).

^{13}C NMR (151 MHz, $CDCl_3$) δ 171.8, 149.2, 140.2, 136.3, 131.9, 130.5, 125.7, 125.2, 121.8, 94.9, 74.2, 54.9, 54.2, 45.0, 44.0, 40.0, 39.6, 38.2, 31.5.

IR: 2960, 2904, 2869, 1750, 1488, 1409, 1364, 1302, 1228, 1179, 1104, 1048, 991 cm^{-1}

HRMS: (FTMS +p APCI) calcd for $C_{26}H_{28}O_2{}^{79}Br^{35}Cl_3$ $[M]^+$ 556.0333 found 556.0342 (Δ=1.7 ppm).

HPLC: (ChiralPak AD-H, 1 mL/min, 1% isopropanol in hexane, λ=230 nm), retention times of 5.51 min (major) and 6.79 min (minor), 99% ee.

7i, (**Tertiary** C–H functionalization product) 2,2,2-trichloroethyl (*S*)-2-(4-bromophenyl)-2-(4-(4-(*tert*-butyl)phenyl)bicyclo[2.1.1]hexan-1-yl)acetate was obtained in 36% yield (20.3 mg) as a colorless liquid, 95% ee.

$[\alpha]^{21}_{D}$: -2.4° (c = 0.1, $CHCl_3$, 95% ee)

^{1}H NMR (400 MHz, $CDCl_3$) δ 7.47 (d, *J* = 8.4 Hz, 2H), 7.32 (d, *J* = 8.4 Hz, 2H), 7.28 (d, *J* = 8.4 Hz, 2H), 7.13 (d, *J* = 8.4 Hz, 2H), 4.84 (d, *J* = 12.0 Hz, 1H), 4.69 (d, *J* = 12.0 Hz, 1H), 3.97 (s, 1H), 1.88 (td, *J* = 5.3, 2.5 Hz, 2H), 1.79 – 1.70 (m, 4H), 1.65 – 1.57 (m, 2H), 1.30 (s, 9H).

^{13}C NMR (151 MHz, $CDCl_3$) δ 170.7, 149.2, 140.5, 135.1, 131.6, 130.7, 125.7, 125.2, 121.7, 94.9, 74.4, 54.6, 50.4, 49.6, 45.3, 45.1, 34.6, 34.4, 31.5.

IR: 2962, 2911, 2868, 1750, 1461, 1411, 1360, 1337, 1268, 1206, 1103, 1051, 1001, 935 cm^{-1}

HRMS: (FTMS +p APCI) calcd for $C_{26}H_{28}O_2{}^{79}Br^{35}Cl_3$ $[M]^+$ 556.0333 found 556.0341 (Δ=1.4 ppm).

HPLC: (ChiralPak AD-H, 1 mL/min, 1% isopropanol in hexane, λ=230 nm), retention times of 15.96 min (major) and 18.11 min (minor), 93% ee.

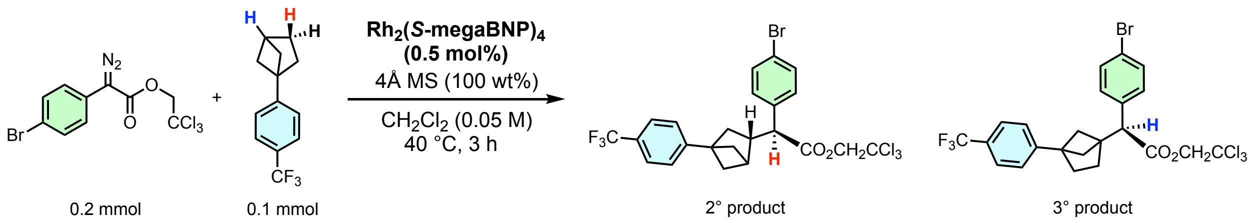

Using ***General Procedure C*** with 2,2,2-trichloroethyl 2-(4-bromophenyl)-2-diazoacetate (**3a**, 0.2 mmol, 74.5 mg, 2 equiv) and 1-(4-(trifluoromethyl)phenyl)bicyclo[2.1.1]hexane (**4c**, 0.1 mmol, 22.6 mg, 1 equiv) and Rh_2(*S*-megaBNP)$_4$ (0.5 % mol, 1.7 mg). Combined secondary and tertiary product were obtained in 88% yield (50.2 mg). After preparative TLC separation (2% Diethyl ether/n-hexane), separated products were further characterized as below.

5j (**Secondary** C–H functionalization product) 2,2,2-trichloroethyl (*S*)-2-(4-bromophenyl)-2-((*S*)-4-(4-(trifluoromethyl)phenyl)bicyclo[2.1.1]hexan-2-yl)acetate was obtained in 62% yield (35.5 mg) as a colorless liquid, >20:1 d.r., 99% ee.

$[\alpha]^{21}_D$: +27.1° (c = 1.25, $CHCl_3$, 99% ee)

^{1}H NMR (400 MHz, $CDCl_3$) δ 7.54 – 7.50 (m, 2H), 7.48 (d, *J* = 8.5 Hz, 2H), 7.33 (d, *J* = 8.5 Hz, 2H), 7.28 – 7.23 (m, 2H), 4.76 (d, *J* = 12.0 Hz, 1H), 4.71 (d, *J* = 12.0 Hz, 1H), 3.61 (d, *J* = 11.8 Hz, 1H), 3.13 – 3.02 (m, 1H), 2.59 (td, *J* = 2.9, 1.2 Hz, 1H), 1.96 (dt, *J* = 6.4, 2.9 Hz, 1H), 1.92 – 1.84 (m, 2H), 1.79 (dd, *J* = 9.6, 7.2 Hz, 1H), 1.66 (dd, *J* = 9.6, 6.4 Hz, 1H), 1.27 (ddd, *J* = 11.7, 4.3, 2.9 Hz, 1H).

^{13}C NMR (151 MHz, $CDCl_3$) δ 171.6, 147.3, 136.1, 132.0, 130.5, 128.63 (q, *J* = 32.3 Hz), 126.2, 125.28 (q, *J* = 3.8 Hz), 124.38 (q, *J* = 272.7 Hz), 122.0, 94.9, 74.2, 54.8, 54.2, 44.8, 43.9, 40.1, 39.5, 38.5.

^{19}F NMR: (565 MHz, $CDCl_3$) δ -62.38.

IR: 2955, 2874, 1750, 1488, 1445, 1373, 1323, 1299, 1266, 1209, 1161, 1121, 1031, 1011 cm^{-1}

HRMS: (FTMS +p APCI) calcd for $C_{22}H_{20}O_2{}^{79}Br^{35}Cl_3F_3$ $[M+H]^+$ 568.9659 found 568.9667 (Δ=1.5 ppm).

HPLC: (ChiralPak AD-H, 1 mL/min, 1% isopropanol in hexane, λ=230 nm), retention times of 9.97 min (major) and 16.26 min (minor), 99% ee.

7j (**Tertiary** C–H functionalization product) 2,2,2-trichloroethyl (*S*)-2-(4-bromophenyl)-2-(4-(4-(trifluoromethyl)phenyl)bicyclo[2.1.1]hexan-1-yl)acetate was obtained in 26% yield (14.8 mg) as a colorless liquid, 90% ee.

$[\alpha]^{21}_D$: -4.0° (c = 0.79, $CHCl_3$, 90% ee)

^{1}H NMR (400 MHz, $CDCl_3$) δ 7.53 (d, *J* = 8.3 Hz, 2H), 7.48 (d, *J* = 8.3 Hz, 2H), 7.29 – 7.26 (m, 4H), 4.85 (d, *J* = 12.0 Hz, 1H), 4.70 (d, *J* = 12.0 Hz, 1H), 3.98 (s, 1H), 1.94 – 1.86 (m, 2H), 1.83 – 1.73 (m, 4H), 1.71 – 1.62 (m, 2H).

^{13}C NMR (151 MHz, $CDCl_3$) δ 170.5, 147.6, 134.8, 131.8, 130.7, 128.60 (q, J = 64.7 Hz), 126.3, 125.30 (q, J = 3.8 Hz), 124.45 (q, J = 241.2 Hz), 121.9, 94.8, 77.4, 77.2, 76.9, 74.4, 54.4, 50.6, 49.8, 45.1, 44.9, 34.7, 31.3.

^{19}F NMR (376 MHz, $CDCl_3$) δ -62.37.

IR: 2956, 2869, 1750, 1489, 1409, 1360, 1333, 1279, 1260, 1121, 1087, 1002, 954 cm^{-1}

HRMS: (FTMS +p APCI) calcd for $C_{22}H_{20}O_2{}^{79}Br^{35}Cl_3F_3$ $[M+H]^+$ 568.9659 found 568.9664 (Δ=0.8 ppm).

HPLC: (ChiralPak AS-H, 0.25 mL/min, 0.5% isopropanol in hexane, λ=230 nm), retention times of 24.70 min (minor) and 27.56 min (major), 90% ee.

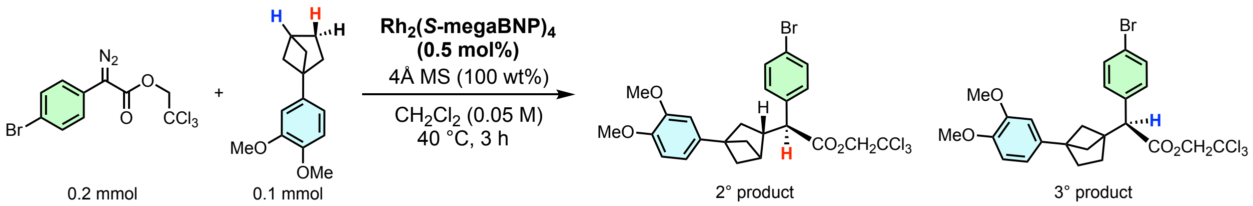

Using ***General Procedure C*** with 2,2,2-trichloroethyl 2-(4-bromophenyl)-2-diazoacetate (**3a**, 0.2 mmol, 74.5 mg, 2 equiv) and 1-(3,4-dimethoxyphenyl)bicyclo[2.1.1]hexane (**4d**, 0.1 mmol, 21.8 mg, 1 equiv) and Rh_2(*S*-megaBNP)$_4$ (0.5 % mol, 1.7 mg). Combined secondary and tertiary product were obtained in combined 79% yield (44.5 mg) as a colorless liquid.

Note that the separation of regioisomers for this reaction failed in our attempts so that the NMR, IR, HRMS and Chiral HPLC were characterized as a mixture. Learning from the diazo scope, secondary product is more likely to have higher enantioselectivity than tertiary product. Thus, we tentatively assign the secondary product with the higher enantioselectivity result and the tertiary product with the relatively lower enantioselectivity from this specific chiral HPLC analysis.

IR: 2950, 2872, 1751, 1587, 1518, 1488, 1411, 1372, 1342, 1233, 1138, 1030, 1011 cm^{-1}

HRMS: (FTMS +p APCI) calcd for $C_{24}H_{25}O_4{}^{79}Br^{35}Cl_3$ $[M+H]^+$ 561.0010 found 561.0001 (Δ=1.5 ppm).

5k, (**Secondary** C–H functionalization product) 2,2,2-trichloroethyl (S)-2-((S)-4-(4-bromophenyl)bicyclo[2.1.1]hexan-2-yl)-2-(4-iodophenyl)acetate was obtained in >20:1 d.r.(determined from crude ^{1}H NMR), 99% ee.

HPLC: (ChiralPak AD-H, 1 mL/min, 2% isopropanol in hexane, λ=230 nm), retention times of 24.11 min (minor) and 38.26 min (minor), 99% ee.

7k, (**Tertiary** C–H functionalization product) 2,2,2-trichloroethyl (S)-2-(4-(4-bromophenyl)bicyclo[2.1.1]hexan-1-yl)-2-(4-iodophenyl)acetate was obtained in 97% ee.

HPLC: (ChiralPak AD-H, 1 mL/min, 2% isopropanol in hexane, λ=230 nm), retention times of 23.19 min (minor) and 32.70 min (major), 97% ee.

^{1}H NMR (**5k** and **7k** as a mixture):

5k (600 MHz, $CDCl_3$) δ 7.47 (d, *J* = 8.5 Hz, 2H), 7.33 (d, *J* = 8.5 Hz, 2H), 6.78 (d, *J* = 8.2 Hz, 1H), 6.72 (dd, *J* = 8.2, 2.0 Hz, 1H), 6.68 (d, *J* = 2.0 Hz, 1H), 4.76 (d, *J* = 12.0 Hz, 1H), 4.71 (d, *J* = 12.0 Hz, 1H), 3.85 (s, 3H), 3.84 (s, 3H), 3.61 (d, *J* = 11.9 Hz, 1H), 3.03 (ddd, *J* = 11.9, 6.7, 3.0 Hz, 1H), 2.53 (td, *J* = 2.9, 1.2 Hz, 1H), 1.91 (dt, *J* = 6.3, 2.9 Hz, 1H), 1.87 – 1.81 (m, 2H), 1.73 (td, *J* = 7.2, 2.9 Hz, 1H), 1.62 – 1.59 (m, 1H), 1.24 (ddd, *J* = 11.1, 4.2, 2.9 Hz, 1H).

7k (600 MHz, $CDCl_3$) δ 7.47 (d, *J* = 8.6 Hz, 2H), 7.28 (d, *J* = 8.5 Hz, 2H), 6.79 (d, *J* = 8.2 Hz, 1H), 6.71 – 6.69 (m, 2H), 4.84 (d, *J* = 12.0 Hz, 1H), 4.70 (d, *J* = 12.0 Hz, 1H), 3.97 (s, 1H), 3.86 (s, 3H), 3.85 (s, 3H), 1.88 – 1.85 (m, 2H), 1.78 (d, *J* = 5.5 Hz, 1H), 1.75 – 1.70 (m, 3H), 1.62 – 1.58 (m, 2H).

^{13}C NMR (**5k** and **7k** as a mixture):

5k (151 MHz, $CDCl_3$) δ 171.7, 148.87, 147.62, 136.27, 136.1, 132.0, 130.5, 121.8, 117.99, 111.16, 109.29, 94.90, 74.2, 56.08, 56.0(overlap with **7k**), 54.8, 54.2, 45.15, 44.0, 39.7, 39.6, 38.2.

7k (151 MHz, $CDCl_3$) δ 170.6, 148.90, 147.61, 136.33, 135.0, 131.7, 130.7, 121.7, 117.97, 111.21, 109.33, 94.85, 74.4, 56.09, 56.0 (overlap with **5k**), 54.5, 50.4, 49.4, 45.4, 45.12, 34.6, 31.4.

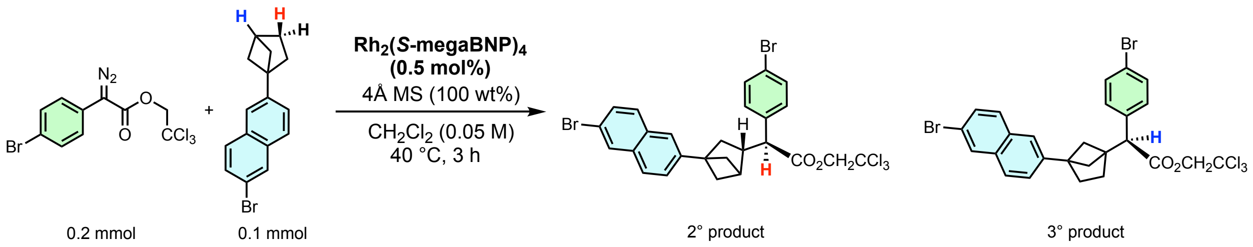

Using ***General Procedure C*** with 2,2,2-trichloroethyl 2-(4-bromophenyl)-2-diazoacetate (**3a**, 0.2 mmol, 74.5 mg, 2 equiv) and 1-(6-bromonaphthalen-2-yl)bicyclo[2.1.1]hexane (**4e**, 0.1 mmol, 28.7 mg, 1 equiv) and Rh_2(*S*-megaBNP)$_4$ (0.5 % mol, 1.7 mg). Combined secondary and tertiary product were obtained in 62% yield (39.2 mg). After preparative TLC separation (2% Diethyl ether/n-hexane), separated products were further characterized as below.

5l (**Secondary** C–H functionalization product) 2,2,2-trichloroethyl (*S*)-2-((*S*)-4-(6-bromonaphthalen-2-yl)bicyclo[2.1.1]hexan-2-yl)-2-(4-bromophenyl)acetate was obtained in 37% yield (23.5 mg) as a colorless liquid, 10:1 d.r., 99% ee.

$[\alpha]^{21}_{D}$: +5.3° (c = 1.72, $CHCl_3$, 99% ee)

^{1}H NMR (400 MHz, $CDCl_3$) δ 7.93 (d, *J* = 1.8 Hz, 1H), 7.65 (d, *J* = 8.5 Hz, 1H), 7.62 (d, *J* = 8.8 Hz, 1H), 7.52 (d, J = 1.8 Hz, 1H), 7.52 – 7.45 (m, 3H), 7.38 – 7.32 (m, 3H), 4.77 (d, *J* = 12.0 Hz, 1H), 4.72 (d, *J* = 12.0 Hz, 1H), 3.65 (d, *J* = 11.8 Hz, 1H), 3.15 – 3.04 (m, 1H), 2.60 (dt, *J* = 2.9, 1.8 Hz, 1H), 2.03 (dt, *J* = 6.4, 2.9 Hz, 1H), 1.98 – 1.90 (m, 2H), 1.83 (dd, *J* = 9.7, 7.0 Hz, 1H), 1.70 (dd, *J* = 9.7, 6.4 Hz, 1H), 1.34 (dt, *J* = 11.8, 3.4 Hz, 1H).

^{13}C NMR (151 MHz, $CDCl_3$) δ 171.7, 141.4, 136.2, 133.3, 132.0, 131.9, 130.5, 129.8, 129.5, 129.4, 127.0, 125.6, 124.1, 121.9, 119.3, 94.9, 74.2, 54.9, 54.6, 44.9, 44.0, 40.1, 39.6, 38.3.

IR: 2951, 2923, 2872, 1750, 1592, 1488, 1373, 1323, 1265, 1206, 1135, 1073, 1032, 998 cm^{-1}

HRMS: (FTMS +p APCI) calcd for $C_{26}H_{21}O_2{}^{79}Br_2{}^{35}Cl_3$ $[M]^+$ 627.8968 found 627.8980 (Δ=1.9 ppm).

HPLC: (ChiralPak AD-H, 1 mL/min, 5% isopropanol in hexane, λ=230 nm), retention times of 7.74 min (major) and 9.11 min (minor), 99% ee.

7l, (**Tertiary** C–H functionalization product) 2,2,2-trichloroethyl (*S*)-2-(4-(6-bromonaphthalen-2-yl)bicyclo[2.1.1]hexan-1-yl)-2-(4-bromophenyl)acetate was obtained in 25% yield (15.7 mg) as a colorless liquid, 94% ee.

$[\alpha]^{21}_{D}$: -11.8° (c = 1.4, $CHCl_3$, 94% ee)

^{1}H NMR (400 MHz, $CDCl_3$) δ 7.95 (d, *J* = 1.9 Hz, 1H), 7.66 (d, *J* = 8.5 Hz, 1H), 7.63 (d, *J* = 8.5 Hz, 1H), 7.54 (d, J = 1.7 Hz, 1H), 7.52 – 7.47 (m, 3H), 7.35 (dd, *J* = 8.5, 1.7 Hz, 1H), 7.30 (d, *J* = 8.5 Hz, 2H), 4.86 (d, *J* = 12.0 Hz, 1H), 4.71 (d, *J* = 12.0 Hz, 1H), 4.01 (s, 1H), 2.00 – 1.93 (m, 2H), 1.87 (d, *J* = 5.6 Hz, 1H), 1.84 – 1.77 (m, 3H), 1.75 – 1.67 (m, 2H).

^{13}C NMR (151 MHz, $CDCl_3$) δ 170.6, 141.7, 135.0, 133.3, 131.9, 131.7, 130.7, 129.8, 129.5, 129.4, 127.0, 125.7, 124.1, 121.8, 119.3, 94.8, 74.4, 54.5, 50.9, 49.8, 45.2, 45.0, 34.6, 31.5.

IR: 2950, 2924, 2870, 1750, 1586, 1483, 1368, 1309, 1246, 1212, 1134, 1089, 1057, 1011, 934 cm^{-1}

HRMS: (FTMS +p APCI) calcd for $C_{26}H_{21}O_2{}^{79}Br_2{}^{35}Cl_3$ $[M]^+$ 627.8968 found 627.8979 (Δ=1.8 ppm).

HPLC: (Regis (S,S) Whelk-O 1, 1 mL/min, 2% isopropanol in hexane, λ=230 nm), retention times of 20.58 min (minor) and 23.44 min (major), 94% ee.

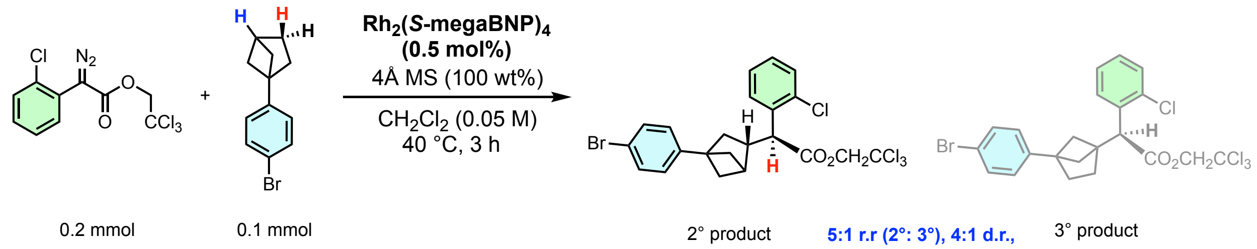

Using ***General Procedure C*** with 2,2,2-trichloroethyl 2-(2-chlorophenyl)-2-diazoacetate (**3i**, 0.2 mmol, 65.6 mg, 2 equiv) and 1-(4-bromophenyl)bicyclo[2.1.1]hexane (**4a**, 0.1 mmol, 23.7 mg, 1 equiv) and Rh_2(*S*-megaBNP)$_4$ (0.5 % mol, 1.7 mg). Combined secondary and tertiary product were obtained in 62% combined yield (33.3 mg). After preparative TLC separation (1% Diethyl ether/n-hexane), separated secondary product is further characterized as below.

Note that tertiary product is a minor product and difficult to decently purify for full characterization. As a result, we only show the full characterization of purified major secondary product.

8a, (**Secondary** C–H functionalization product) 2,2,2-trichloroethyl (*S*)-2-((*S*)-4-(4-bromophenyl)bicyclo[2.1.1]hexan-2-yl)-2-(2-chlorophenyl)acetate was obtained in 52% yield (27.8 mg) as a white solid, 4:1 d.r., 90% ee.

$[\alpha]^{21}_{D}$: -0.8° (c = 1.64, $CHCl_3$, 90% ee)

^{1}H NMR: (400 MHz, $CDCl_3$) δ 7.57 (dd, J = 7.8, 1.8 Hz, 1H), 7.40 (dd, J = 7.8, 1.6 Hz, 1H), 7.37 (d, J = 8.5 Hz, 2H), 7.30 – 7.26 (m, 1H), 7.24 – 7.19 (m, 1H), 7.04 (d, J = 8.5 Hz, 2H), 4.76 (d, J = 11.9 Hz, 1H), 4.72 (d, J = 11.9 Hz, 1H), 4.39 (d, J = 11.7 Hz, 1H), 3.13 – 3.02 (m, 1H), 2.63 (s, 1H), 1.93 (dt, J = 6.7, 2.9 Hz, 1H), 1.85 – 1.76 (m, 3H), 1.64 – 1.57 (m, 1H), 1.25 (ddd, J = 11.2, 4.2, 2.9 Hz, 1H).

^{13}C NMR: (101 MHz, $CDCl_3$) δ 171.6, 142.4, 135.2, 134.7, 131.3, 129.8, 129.5, 128.9, 127.7, 127.3, 120.0, 94.9, 74.2, 53.8, 50.2, 44.5, 44.1, 40.1, 39.8, 37.9.

IR: 2953, 2872, 1751, 1492, 1474, 1443, 1395, 1337, 1208, 1144, 1072, 1036, 1009 cm^{-1}

HRMS: (FTMS +p APCI) calcd for $C_{22}H_{20}O_2{}^{79}Br^{35}Cl_4$ $[M+H]^+$ 534.9395 found 534.9402(Δ=1.28 ppm).

HPLC: (ChiralPak AD-H, 1 mL/min, 1% isopropanol in hexane, λ=230 nm), retention times of 8.97 min (major) and 12.34 min (minor), 90% ee.

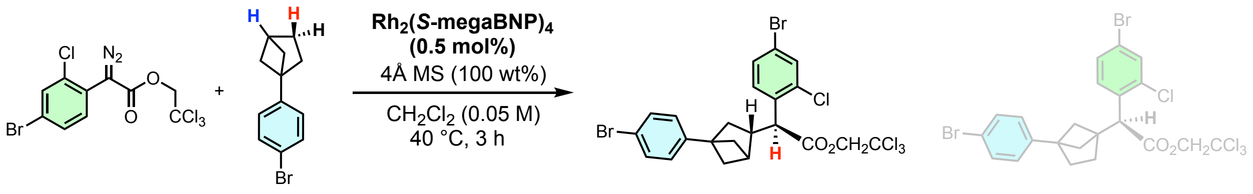

0.2 mmol 0.1 mmol 2° product **11:1 r.r (2°: 3°), 12:1 d.r.,** 3° product

Using ***General Procedure C*** with 2,2,2-trichloroethyl 2-(4-bromo-2-chlorophenyl)-2-diazoacetate (**3j**, 0.2 mmol, 81.3 mg, 2 equiv) and 1-(4-bromophenyl)bicyclo[2.1.1]hexane (**4a**, 0.1 mmol, 23.7 mg, 1 equiv) and Rh_2(*S*-megaBNP)$_4$ (0.5 % mol, 1.7 mg). Combined secondary and tertiary product were obtained in 76% combined yield (46.8 mg).

8b, (**Secondary** C–H functionalization product) 2,2,2-trichloroethyl (*S*)-2-(4-bromo-2-chlorophenyl)-2-((*S*)-4-(4-bromophenyl)bicyclo[2.1.1]hexan-2-yl)acetate was obtained in 70% yield (27.8 mg) as a colorless liquid, 12:1 d.r., 98% ee.

$[\alpha]^{21}_{D}$: +14.9° (c = 1.94, $CHCl_3$, 98% ee)

^{1}H NMR: (400 MHz, $CDCl_3$) δ 7.58 (d, J = 1.9 Hz, 1H), 7.45 (d, J = 8.4 Hz, 1H), 7.43 – 7.40 (m, 1H), 7.38 (d, J = 8.4 Hz, 2H), 7.03 (d, J = 8.4 Hz, 2H), 4.76 (d, J = 12.0 Hz, 1H), 4.72 (d, J = 12.0 Hz, 1H), 4.31 (d, J = 11.7 Hz, 1H), 3.07 – 2.98 (m, 1H), 2.63 (td, J = 2.9, 1.2 Hz, 1H), 1.93 (dt, J = 6.5, 2.9 Hz, 1H), 1.87 – 1.76 (m, 3H), 1.59 (dd, J = 9.3, 6.5 Hz, 1H), 1.21 (ddd, J = 11.2, 4.2, 2.8 Hz, 1H).

^{13}C NMR: (101 MHz, $CDCl_3$) δ 171.1, 142.2, 135.6, 134.3, 132.4, 131.4, 130.7, 130.6, 127.7, 121.8, 120.1, 94.8, 74.3, 53.9, 49.9, 44.5, 44.1, 40.1, 39.8, 37.9.

IR: 2954, 2873, 1751, 1582, 1558, 1492, 1471, 1444, 1378, 1286, 1207, 1183, 1132, 1073, 1030, 971, 926 cm^{-1}

HRMS: (FTMS +p APCI) calcd for $C_{22}H_{19}O_2{}^{79}Br_2{}^{35}Cl_4$ $[M+H]^+$ 612.8500 found 612.8510(Δ=1.5 ppm).

HPLC: (ChiralPak AD-H, 1 mL/min, 1% isopropanol in hexane, λ=230 nm), retention times of 9.57 min (major) and 13.79 min (minor), 98% ee.

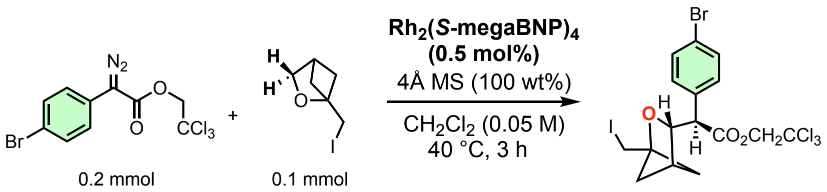

Using ***General Procedure C*** with 2,2,2-trichloroethyl 2-(4-bromophenyl)-2-diazoacetate (**3a**, 0.2 mmol, 74.5 mg, 2 equiv) and 1-(iodomethyl)-2-oxabicyclo[2.1.1]hexane (**10a**, 0.1 mmol, 22.4 mg, 1 equiv) and Rh_2(*S*-megaBNP)$_4$ (0.5 % mol, 1.7 mg). 2,2,2-trichloroethyl (*R*)-2-(4-bromophenyl)-2-((*S*)-1-(iodomethyl)-2-oxabicyclo[2.1.1]hexan-3-yl)acetate **11a** was obtained in 40% yield (22.7 mg) as a colorless liquid, >20:1 d.r., 90% ee.

$[\alpha]^{21}_{D}$: +48.2° (c = 2.49, $CHCl_3$, 90% ee)

^{1}H NMR (400 MHz, $CDCl_3$) δ 7.49 (d, *J* = 8.5 Hz, 2H), 7.33 (d, *J* = 8.5 Hz, 2H), 4.80 (d, *J* = 12.0 Hz, 1H), 4.69 (d, *J* = 12.0 Hz, 1H), 4.52 (d, *J* = 10.2 Hz, 1H), 3.71 (d, *J* = 10.2 Hz, 1H), 3.39 (d, *J* = 10.9 Hz, 1H), 3.35 (d, *J* = 10.9 Hz, 1H), 2.88 (t, *J* = 3.0 Hz, 1H), 1.96 (dd, *J* = 7.2, 3.0 Hz, 1H), 1.81 – 1.74 (m, 2H), 1.73 – 1.66 (m, 1H).

^{13}C NMR (101 MHz, $CDCl_3$) δ 169.8, 134.3, 132.0, 130.5, 122.2, 94.7, 88.1, 80.9, 74.2, 54.7, 43.7, 39.3, 38.8, 4.5.

IR: 3000, 2952, 2922, 1748, 1488, 1439, 1410, 1371, 1317, 1275, 1193, 1140, 1093, 1059, 1011, 978, 949 cm^{-1}

HRMS: (FTMS +p APCI) calcd for $C_{16}H_{16}O_3{}^{79}Br^{35}Cl_3{}^{127}I$ $[M+H]^+$ 566.8388 found 566.8392 (Δ=0.8 ppm).

HPLC: (ChiralPak AD-H, 1 mL/min, 5% isopropanol in hexane, λ=230 nm), retention times of 6.48 min (major) and 9.90 min (minor), 90% ee.

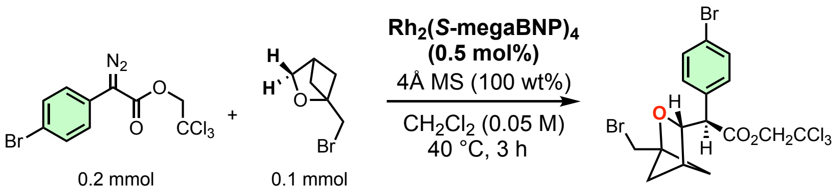

Using ***General Procedure C*** with 2,2,2-trichloroethyl 2-(4-bromophenyl)-2-diazoacetate (**3a**, 0.2 mmol, 74.5 mg, 2 equiv) and 1-(bromomethyl)-2-oxabicyclo[2.1.1]hexane (**10b**, 0.1 mmol, 17.7 mg, 1 equiv) and Rh_2(*S*-megaBNP)$_4$ (0.5 % mol, 1.7 mg). 2,2,2-trichloroethyl (*R*)-2-(4-bromophenyl)-2-((*S*)-1-(bromomethyl)-2-oxabicyclo[2.1.1]hexan-3-yl)acetate **11b** was obtained in 58% yield (30.2 mg) as a colorless liquid, >20:1 d.r., 92% ee.

$[\alpha]^{21}_D$: +44.8° (c = 1.03, $CHCl_3$, 92% ee)

^{1}H NMR (400 MHz, $CDCl_3$) δ 7.49 (d, *J* = 8.5 Hz, 2H), 7.32 (d, *J* = 8.5 Hz, 2H), 4.79 (d, *J* = 12.0 Hz, 1H), 4.69 (d, *J* = 12.0 Hz, 1H), 4.56 (d, *J* = 10.3 Hz, 1H), 3.71 (d, *J* = 10.3 Hz, 1H), 3.54 (d, *J* = 11.2 Hz, 1H), 3.50 (d, *J* = 11.2 Hz, 1H), 2.94 (t, *J* = 3.2 Hz, 1H), 1.98 (dd, *J* = 7.3, 3.2 Hz, 1H), 1.87 – 1.77 (m, 2H), 1.71 (ddd, *J* = 10.0, 7.3, 1.4 Hz, 1H).

^{13}C NMR (101 MHz, $CDCl_3$) δ 169.8, 134.2, 132.0, 130.4, 122.2, 94.6, 88.2, 80.6, 74.1, 54.7, 43.5, 40.0, 37.7, 31.0.

IR: 3004, 2957, 2923, 1750, 1489, 1427, 1410, 1370, 1323, 1276, 1212, 1178, 1097, 1060, 1012, 963, 921 cm^{-1}

HRMS: (FTMS +p APCI) calcd for $C_{16}H_{16}O_3{}^{79}Br_2{}^{35}Cl_3$ $[M+H]^+$ 518.8526 found 518.8534 (Δ=1.4 ppm).

HPLC: (ChiralPak AD-H, 1 mL/min, 5% isopropanol in hexane, λ=230 nm), retention times of 6.43 min (major) and 10.13 min (minor), 92% ee.

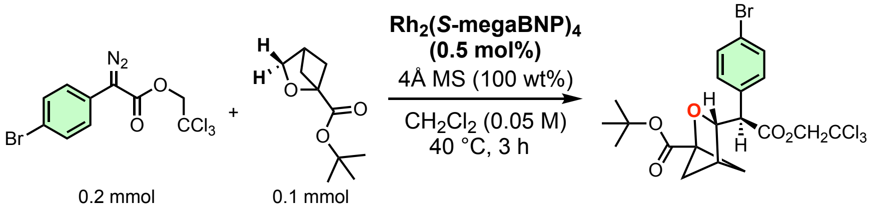

Using ***General Procedure C*** with 2,2,2-trichloroethyl 2-(4-bromophenyl)-2-diazoacetate (**3a**, 0.2 mmol, 74.5 mg, 2 equiv) and *tert*-butyl 2-oxabicyclo[2.1.1]hexane-1-carboxylate (**10c**, 0.1 mmol, 18.4 mg, 1 equiv) and Rh_2(*S*-megaBNP)$_4$ (0.5 % mol, 1.7 mg). *tert*-butyl (*S*)-3-((*R*)-1-(4-bromophenyl)-2-oxo-2-(2,2,2-trichloroethoxy)ethyl)-2-oxabicyclo[2.1.1]hexane-1-carboxylate **11c** was obtained in 50% yield (26.5 mg) as a colorless liquid, 10:1 d.r., 86% ee.

$[\alpha]^{21}_{D}$: +37.9° (c = 1.49, $CHCl_3$, 86% ee)

^{1}H NMR (400 MHz, $CDCl_3$) δ 7.47 (d, *J* = 8.5 Hz, 2H), 7.33 (d, *J* = 8.5 Hz, 2H), 4.78 (d, *J* = 12.0 Hz, 1H), 4.68 (d, *J* = 12.0 Hz, 1H), 4.60 (d, *J* = 10.2 Hz, 1H), 3.74 (d, *J* = 10.2 Hz, 1H), 2.95 (t, *J* = 3.1 Hz, 1H), 2.24 (dd, *J* = 6.9, 3.1 Hz, 1H), 2.12 (dd, *J* = 7.4, 3.1 Hz, 1H), 1.99 – 1.86 (m, 2H), 1.44 (s, 9H).

^{13}C NMR (101 MHz, $CDCl_3$) δ 169.7, 166.7, 134.0, 131.9, 130.5, 122.2, 94.6, 86.6, 82.1, 80.0, 74.1, 54.7, 44.6, 40.8, 39.0, 28.1.

IR: 2959, 2923, 2852, 1747, 1489, 1457, 1410, 1368, 1260, 1219, 1134, 1073, 1012, 978 cm^{-1}

HRMS: (FTMS -p APCI) calcd for $C_{20}H_{21}O_5{}^{79}Br^{35}Cl_3$ [M-H]$^-$ 524.9643 found 524.9653 (Δ=1.7 ppm).

HPLC: (ChiralPak AS-H, 1 mL/min, 1% isopropanol in hexane, λ=230 nm), retention times of 7.14 min (minor) and 8.63 min (major), 86% ee.

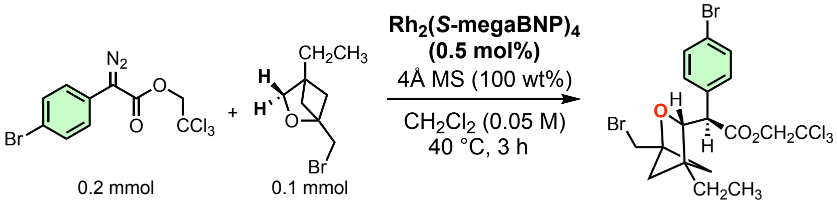

Using ***General Procedure C*** with 2,2,2-trichloroethyl 2-(4-bromophenyl)-2-diazoacetate (**3a**, 0.2 mmol, 74.5 mg, 2 equiv) and 1-(bromomethyl)-4-ethyl-2-oxabicyclo[2.1.1]hexane (**10d**, 0.1 mmol, 20.5 mg, 1 equiv) and Rh_2(*S*-megaBNP)$_4$ (0.5 % mol, 1.7 mg). 2,2,2-trichloroethyl (*R*)-2-((*R*)-1-(bromomethyl)-4-ethyl-2-oxabicyclo[2.1.1]hexan-3-yl)-2-(4-bromophenyl)acetate **11d** was obtained in 46% yield (25.3 mg) as a colorless liquid, 10:1 d.r., 84% ee.

$[\alpha]^{21}_D$: +37.9° (c = 1.49, $CHCl_3$, 84% ee)

^{1}H NMR (400 MHz, $CDCl_3$) δ 7.46 (d, *J* = 8.5 Hz, 2H), 7.29 (d, *J* = 8.5 Hz, 2H), 4.76 (d, *J* = 12.0 Hz, 1H), 4.64 (d, *J* = 12.0 Hz, 1H), 4.55 (d, *J* = 10.0 Hz, 1H), 3.63 (d, *J* = 10.0 Hz, 1H), 3.49 (d, *J* = 11.1 Hz, 1H), 3.44 (d, *J* = 11.1 Hz, 1H), 1.80 (d, *J* = 6.3 Hz, 1H), 1.73 – 1.56 (m, 5H), 0.97 (t, *J* = 7.4 Hz, 3H).

^{13}C NMR (101 MHz, $CDCl_3$) δ 170.1, 134.2, 131.9, 130.8, 122.1, 94.6, 84.5, 80.9, 74.5, 53.7, 53.6, 44.5, 39.4, 31.5, 21.7, 10.4.

IR: 2960, 2921, 2879, 2850, 1749, 1488, 1430, 1408, 1380, 1253, 1136, 1073, 1012, 975 cm^{-1}

HRMS: (FTMS +p APCI) calcd for $C_{18}H_{20}O_3{}^{79}Br_2{}^{35}Cl_3$ $[M+H]^+$ 546.8839 found 546.8843 (Δ=0.7 ppm).

HPLC: (ChiralPak AD-H, 2 mL/min, 2% isopropanol in hexane, λ=230 nm), retention times of 5.31 min (major) and 9.48 min (minor), 84% ee.

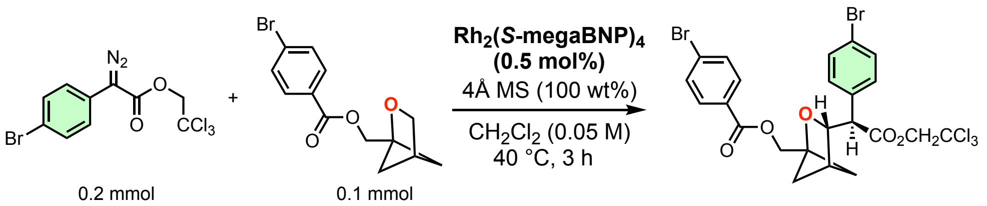

Using ***General Procedure C*** with 2,2,2-trichloroethyl 2-(4-bromophenyl)-2-diazoacetate (**3a**, 0.2 mmol, 74.5 mg, 2 equiv) and (2-oxabicyclo[2.1.1]hexan-1-yl)methyl 4-bromobenzoate (**10e**, 0.1 mmol, 29.7 mg, 1 equiv) and Rh_2(*S*-megaBNP)$_4$ (0.5 % mol, 1.7 mg). ((*S*)-3-((*R*)-1-(4-bromophenyl)-2-oxo-2-(2,2,2-trichloroethoxy)ethyl)-2-oxabicyclo[2.1.1]hexan-1-yl)methyl 4-bromobenzoate **11e** was obtained in 40% yield (25.7 mg) as a colorless liquid, >20:1 d.r., 86% ee.

$[\alpha]^{21}_D$: +7.0° (c = 0.35, $CHCl_3$, 86% ee)

^{1}H NMR (400 MHz, $CDCl_3$) δ 7.87 (d, *J* = 8.5 Hz, 2H), 7.59 (d, *J* = 8.5 Hz, 2H), 7.47 (d, *J* = 8.5 Hz, 2H), 7.30 (d, *J* = 8.5 Hz, 2H), 4.79 (d, *J* = 12.0 Hz, 1H), 4.69 (d, *J* = 12.0 Hz, 1H), 4.59 (d, *J* = 10.2 Hz, 1H), 4.49 (d, *J* = 12.2 Hz, 1H), 4.45 (d, *J* = 12.2 Hz, 1H), 3.70 (d, *J* = 10.2 Hz, 1H), 2.99 (t, *J* = 3.0 Hz, 1H), 2.00 (dd, *J* = 6.9, 3.0 Hz, 1H), 1.86 (dd, *J* = 6.9, 3.0 Hz, 1H), 1.83 – 1.71 (m, 2H).

^{13}C NMR (101 MHz, $CDCl_3$) δ 169.8, 165.6, 134.3, 132.0, 131.9, 131.3, 130.4, 128.9, 128.4, 122.2, 94.7, 87.7, 79.8, 74.2, 63.4, 54.9, 42.8, 40.9, 37.2.

IR: 2999, 2956, 2923, 1749, 1720, 1590, 1488, 1441, 1398, 1316, 1266, 1173, 1115, 1012 cm^{-1}

HRMS: (FTMS +p APCI) calcd for $C_{23}H_{20}O_5{}^{79}Br_2{}^{35}Cl_3$ $[M+H]^+$ 638.8738 found 638.8750 (Δ=2.0 ppm).

HPLC: (Regis (S,S) Whelk-O 1, 1 mL/min, 5% isopropanol in hexane, λ=230 nm), retention times of 23.72 min (major) and 27.27 min (minor), 86% ee.

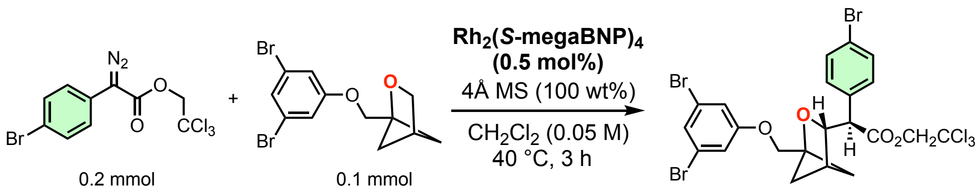

Using ***General Procedure C*** with 2,2,2-trichloroethyl 2-(4-bromophenyl)-2-diazoacetate (**3a**, 0.2 mmol, 74.5 mg, 2 equiv) and 1-((3,5-dibromophenoxy)methyl)-2-oxabicyclo[2.1.1]hexane (**10f**, 0.1 mmol, 34.8 mg, 1 equiv) and Rh_2(*S*-megaBNP)$_4$ (0.5 % mol, 1.7 mg). 2,2,2-trichloroethyl (*R*)-2-(4-bromophenyl)-2-((*S*)-1-((3,5-dibromophenoxy)methyl)-2-oxabicyclo[2.1.1]hexan-3-yl)acetate **11f** was obtained in 49% yield (33.9 mg) as a colorless liquid, >20:1 d.r., 85% ee.

$[\alpha]^{21}_{D}$: +10.7° (c = 0.26, $CHCl_3$, 85% ee)

^{1}H NMR (400 MHz, $CDCl_3$) δ 7.48 (d, *J* = 8.5 Hz, 2H), 7.30 (d, *J* = 8.5 Hz, 2H), 7.23 (t, *J* = 1.6 Hz, 1H), 6.97 (d, *J* = 1.6 Hz, 2H), 4.79 (d, *J* = 12.0 Hz, 1H), 4.70 (d, *J* = 12.0 Hz, 1H), 4.60 (d, *J* = 10.3 Hz, 1H), 4.09 (s, 2H), 3.70 (d, *J* = 10.3 Hz, 1H), 3.00 (t, *J* = 3.1 Hz, 1H), 2.02 (dd, *J* = 6.9, 3.1 Hz, 1H), 1.90 (dd, *J* = 6.9, 3.1 Hz, 1H), 1.81 – 1.70 (m, 2H).

^{13}C NMR (151 MHz, $CDCl_3$) δ 169.8, 160.0, 134.2, 132.1, 130.4, 126.9, 123.2, 122.3, 117.3, 94.7, 88.0, 79.9, 74.2, 67.4, 54.9, 42.8, 40.9, 37.0.

IR: 3000, 2953, 2923, 2852, 1750, 1583, 1558, 1438, 1254, 1229, 1139, 1073, 1012, 958 cm^{-1}

HRMS: (FTMS +p APCI) calcd for $C_{22}H_{19}O_4{}^{79}Br_3{}^{35}Cl_3$ $[M+H]^+$ 688.7894 found 688.7900 (Δ=0.9 ppm).

HPLC: (ChiralPak AD-H, 1 mL/min, 1% isopropanol in hexane, λ=230 nm), retention times of 11.75 min (major) and 13.94 min (minor), 85% ee.

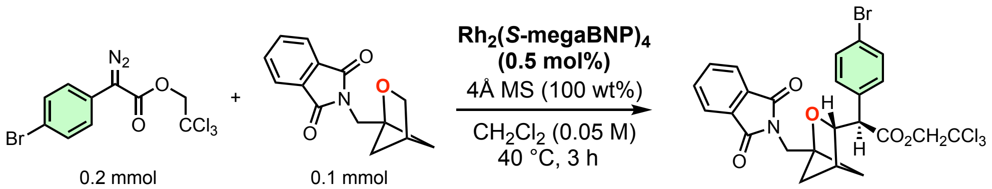

Using ***General Procedure C*** with 2,2,2-trichloroethyl 2-(4-bromophenyl)-2-diazoacetate (**3a**, 0.2 mmol, 74.5 mg, 2 equiv) and 2-((2-oxabicyclo[2.1.1]hexan-1-yl)methyl)isoindoline-1,3-dione (**10g**, 0.1 mmol, 24.3 mg, 1 equiv) and Rh_2(*S*-megaBNP)$_4$ (0.5 % mol, 1.7 mg). 2,2,2-trichloroethyl (*R*)-2-(4-bromophenyl)-2-((*S*)-1-((1,3-dioxoisoindolin-2-yl)methyl)-2-oxabicyclo[2.1.1]hexan-3-yl)acetate **11g** was obtained in 42% yield (24.7 mg) as a white solid, >20:1 d.r., 85% ee.

$[\alpha]^{21}_{D}$: +47.7° (c = 0.8, $CHCl_3$, 85% ee)

^{1}H NMR (400 MHz, $CDCl_3$) δ 7.86 (dd, *J* = 5.5, 3.1 Hz, 2H), 7.75 (dd, *J* = 5.5, 3.1 Hz, 2H), 7.39 (d, *J* = 8.5 Hz, 2H), 7.20 (d, *J* = 8.5 Hz, 2H), 4.77 (d, *J* = 12.0 Hz, 1H), 4.65 (d, *J* = 12.0 Hz, 1H), 4.52 (d, *J* = 10.1 Hz, 1H), 3.98 (d, *J* = 14.4 Hz, 1H), 3.93 (d, *J* = 14.4 Hz, 1H), 3.64 (d, *J* = 10.1 Hz, 1H), 2.88 (t, *J* = 3.0 Hz, 1H), 1.93 – 1.86 (m, 1H), 1.77 – 1.65 (m, 3H).

^{13}C NMR (101 MHz, $CDCl_3$) δ 169.8, 168.0, 134.3, 134.2, 132.1, 131.8, 130.4, 123.5, 122.0, 94.7, 88.2, 79.8, 74.1, 54.8, 43.5, 40.5, 38.6, 37.9.

IR: 2958, 2920, 2851, 1774, 1750, 1715, 1489, 1393, 1190, 1139, 1073, 1012, 979, 954 cm^{-1}

HRMS: (FTMS +p APCI) calcd for $C_{24}H_{20}O_5N^{79}Br^{35}Cl_3$ $[M+H]^+$ 585.9585 found 585.9590 (Δ=0.8 ppm).

HPLC: (ChiralPak AD-H, 1 mL/min, 10% isopropanol in hexane, *λ*=230 nm), retention times of 13.17 min (minor) and 15.55 min (minor), 84% ee.

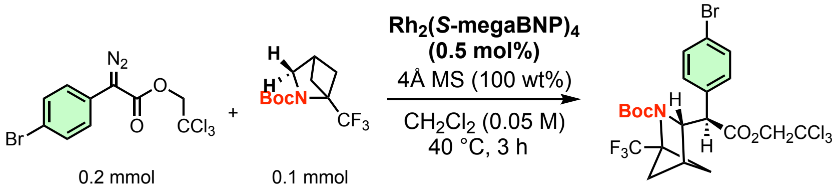

Using ***General Procedure C*** with 2,2,2-trichloroethyl 2-(4-bromophenyl)-2-diazoacetate (**3a**, 0.2 mmol, 74.5 mg, 2 equiv) and *tert*-butyl 1-(trifluoromethyl)-2-azabicyclo[2.1.1]hexane-2-carboxylate (**13a**, 0.1 mmol, 25.1 mg, 1 equiv) and Rh_2(*S*-megaBNP)$_4$ (0.5 % mol, 1.7 mg). *tert*-butyl (*S*)-3-((*R*)-1-(4-bromophenyl)-2-oxo-2-(2,2,2-trichloroethoxy)ethyl)-1-(trifluoromethyl)-2-azabicyclo[2.1.1]hexane-2-carboxylate **14a** was obtained in 32% yield (19.1 mg) as a colorless liquid, >20:1 d.r., 98% ee.

$[\alpha]^{21}_{D}$: +9.1° (c = 0.55, $CHCl_3$, 98% ee)

^{1}H NMR (400 MHz, $CDCl_3$) δ 7.45 (d, *J* = 8.5 Hz, 2H), 7.31 (d, *J* = 8.5 Hz, 2H), 4.84 (d, *J* = 12.0 Hz, 1H), 4.67 (d, *J* = 12.0 Hz, 1H), 4.45 (d, *J* = 8.3 Hz, 1H), 3.95 (d, *J* = 8.3 Hz, 1H), 2.88 (t, *J* = 3.3 Hz, 1H), 2.26 (dd, *J* = 7.6, 3.3 Hz, 1H), 2.16 (dd, *J* = 8.2, 3.3 Hz, 1H), 2.03 (dd, *J* = 10.3, 8.2 Hz, 1H), 1.68 (dd, *J* = 10.3, 7.6 Hz, 1H), 1.24 (s, 9H).

^{13}C NMR (151 MHz, $CDCl_3$) δ 170.3, 156.2, 134.3, 131.6, 131.0, 122.54 (q, *J* = 275.0 Hz), 122.0, 94.6, 81.5, 74.5, 71.30 (q, *J* = 36.8 Hz), 65.0, 54.7, 41.7, 38.5, 37.8, 27.9.

^{19}F NMR (376 MHz, $CDCl_3$) δ -67.66.

IR: 2985, 2958, 2931, 1752, 1726, 1712, 1489, 1368, 1332, 1247, 1205, 1177, 1128, 1011, 954 cm^{-1}

HRMS: (FTMS -p APCI) calcd for $C_{25}H_{30}O_6N^{79}Br^{35}Cl_3$ $[M-H]^-$ 624.0328 found 624.0324 (Δ=0.6 ppm).

HPLC: (ChiralPak AD-H, 1 mL/min, 1% isopropanol in hexane, λ=230 nm), retention times of 7.72 min (minor) and 11.21 min (major), 98% ee.

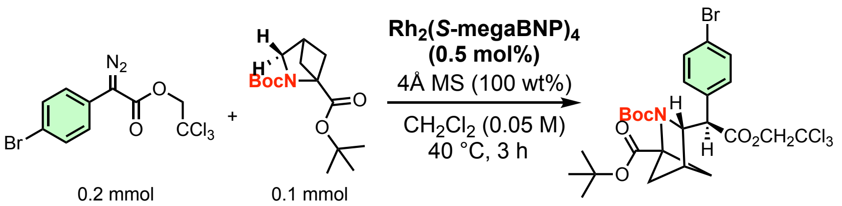

Using ***General Procedure C*** with 2,2,2-trichloroethyl 2-(4-bromophenyl)-2-diazoacetate (**3a**, 0.2 mmol, 74.5 mg, 2 equiv) and di-*tert*-butyl 2-azabicyclo[2.1.1]hexane-1,2-dicarboxylate (**13b**, 0.1 mmol, 28.3 mg, 1 equiv) and Rh_2(*S*-megaBNP)$_4$ (0.5 % mol, 1.7 mg). di-*tert*-butyl (*S*)-3-((*R*)-1-(4-bromophenyl)-2-oxo-2-(2,2,2-trichloroethoxy)ethyl)-2-azabicyclo[2.1.1]hexane-1,2-dicarboxylate **14b** was obtained in 48% yield (30.1 mg) as a white solid, >10:1 d.r., 95% ee.

$[\alpha]^{21}_D$: +23.3° (c = 0.86, $CHCl_3$, 94% ee)

^{1}H NMR (400 MHz, $CDCl_3$) δ 7.45 (d, *J* = 8.5 Hz, 2H), 7.31 (d, *J* = 8.5 Hz, 2H), 4.87 (d, *J* = 12.0 Hz, 1H), 4.65 (d, *J* = 12.0 Hz, 1H), 4.15 (d, *J* = 9.1 Hz, 1H), 3.92 (d, *J* = 9.1 Hz, 1H), 2.76 (t, *J* = 3.1 Hz, 1H), 2.21 (dd, *J* = 7.8, 3.1 Hz, 1H), 2.09 (dd, *J* = 10.4, 8.1 Hz, 1H), 1.96 (dd, *J* = 8.1, 3.1 Hz, 1H), 1.50 – 1.47 (m, 10H), 1.12 (s, 9H).

^{13}C NMR (101 MHz, $CDCl_3$) δ 170.6, 167.6, 158.7, 135.0, 131.6, 131.1, 121.8, 94.7, 81.1, 77.4, 74.3, 72.1, 65.6, 55.4, 42.3, 40.6, 39.4, 28.1, 28.0.

IR: 2977, 2932, 1736, 1706, 1489, 1456, 1367, 1334, 1139, 1098, 1012, 936 cm^{-1}

HRMS: (FTMS -p APCI) calcd for $C_{21}H_{21}O_4N^{79}Br^{35}Cl_3F_3$ [M-H]$^-$ 591.9677 found 591.9673 (Δ=0.7 ppm).

HPLC: (ChiralPak AD-H, 0.5 mL/min, 1% isopropanol in hexane, λ=230 nm), retention times of 17.73 min (minor) and 19.20 min (major), 94% ee.

4.4 Failed reactions with different aryl diazo acetate substrates

Reaction with methyl p-bromophenyldiazoacetate. Under the optimized reaction condition, the reaction with methyl ester diazo failed to generate desired C–H functionalized products (missing distinctive signals of desired C–H functionalized products in the ^{1}H NMR of the crude reaction mixture).

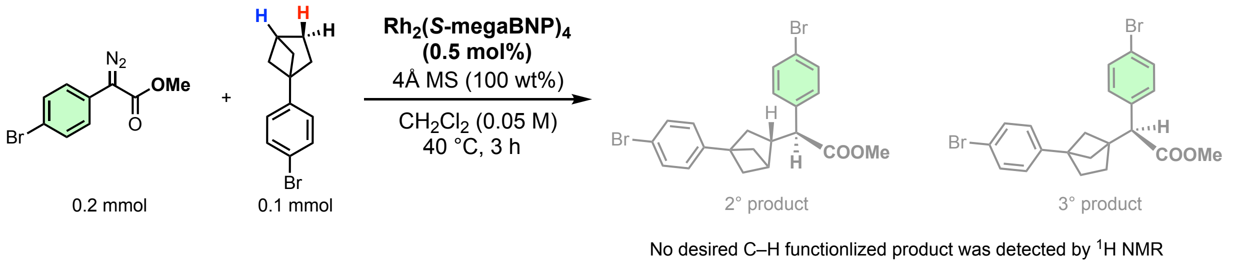

Reaction with trichloroethyl phenyldiazoacetate.

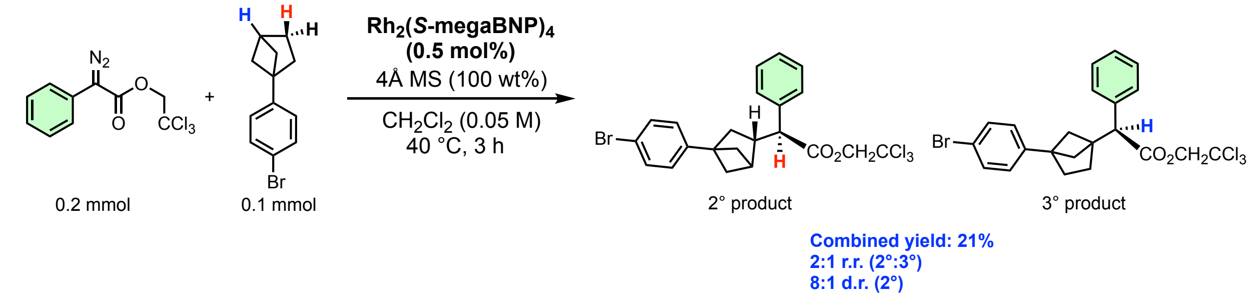

The reaction with unsubstituted trichloroethyl phenyldiazoacetate under the standard conditions gave a low yield of C-H functionalization products (21%). Analysis of thhe ^{1}H NMR of the crude reaction mixture revealed that the diazo compound is fully consumed but the reactions give with carbene dimerization products (5.07 ppm, s) and a rich mixture of unidentified side products (ass seen by the multiple peaks for the trichloroethyl group between 4.4 - 5 ppm). It was possible to isolate d C–H functionalized products but with low yield (21% combined isolation yield), 2:1 r.r (2°:3°) and 8:1 d.r. for secondary C–H functionalized product..

Spectral data of crude reaction mixture

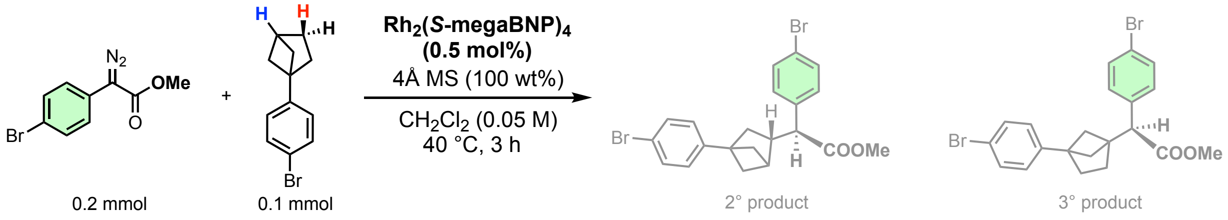

No desired C–H functionlized product was detected by ^{1}H NMR

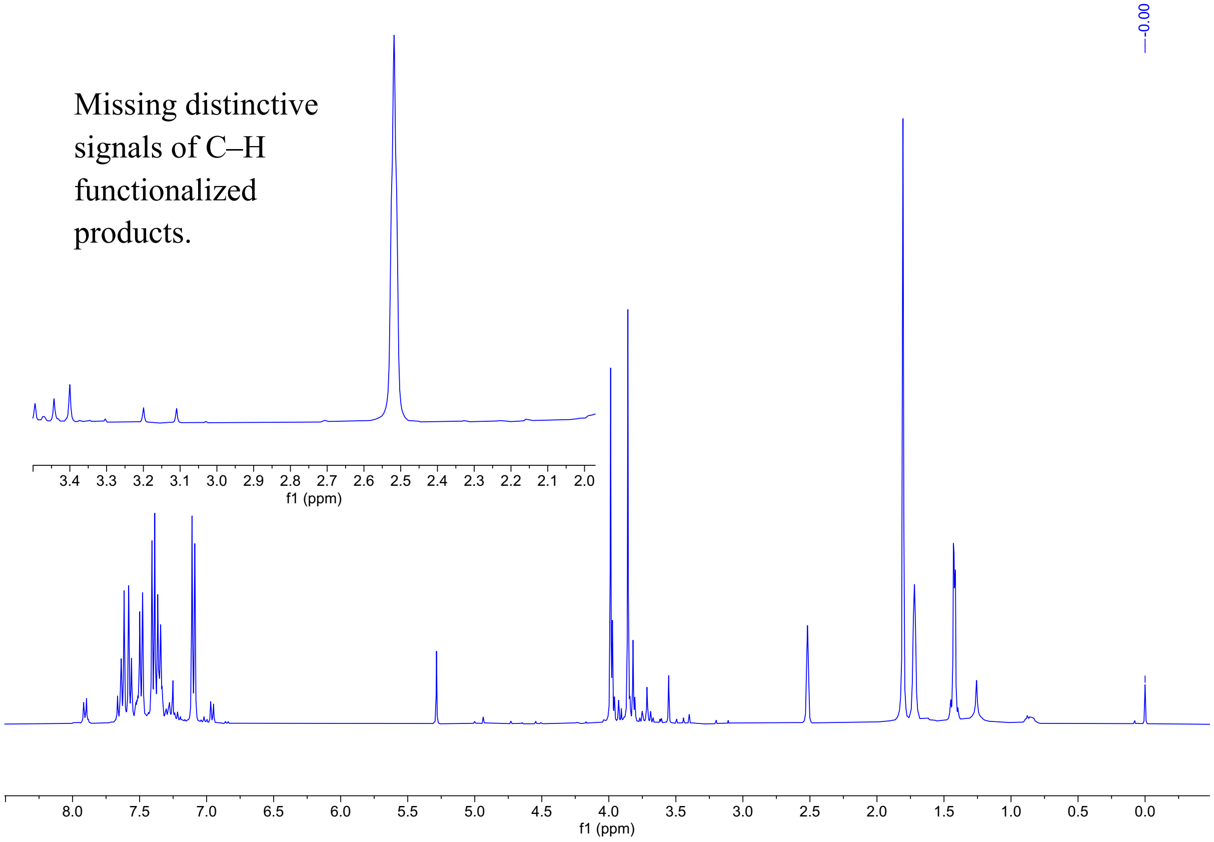

Spectral data of crude reaction mixture

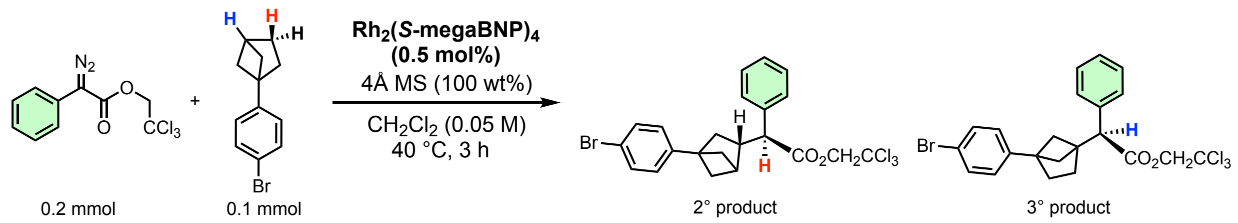

Combined yield: 21%
2:1 r.r. (2°:3°)
8:1 d.r. (2°)

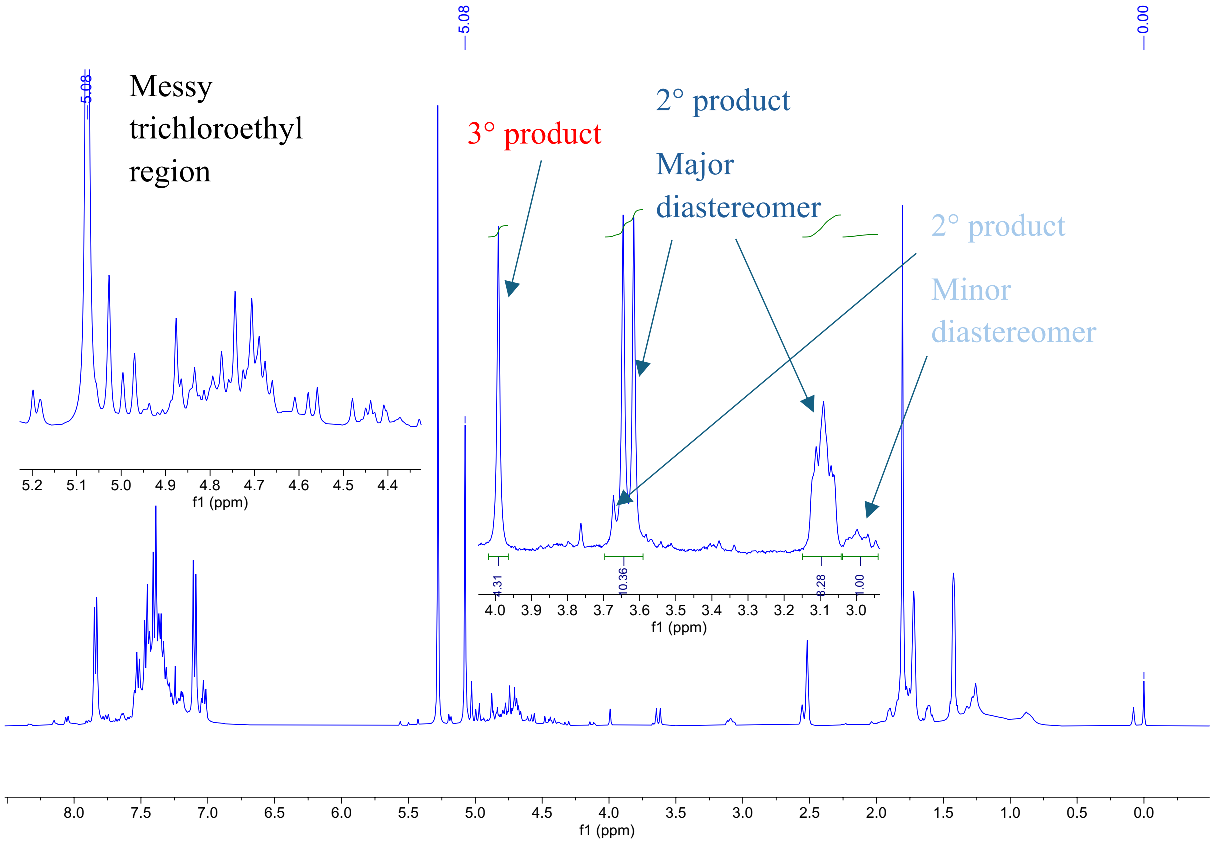

Spectral data of purified products

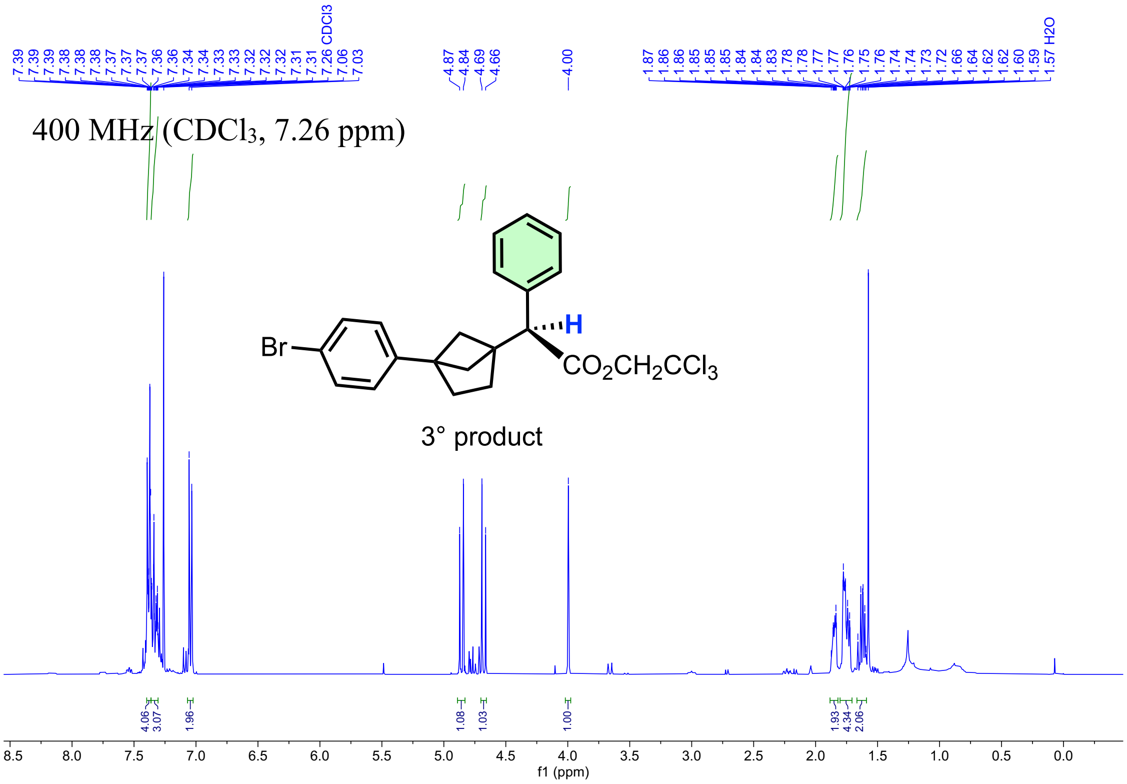

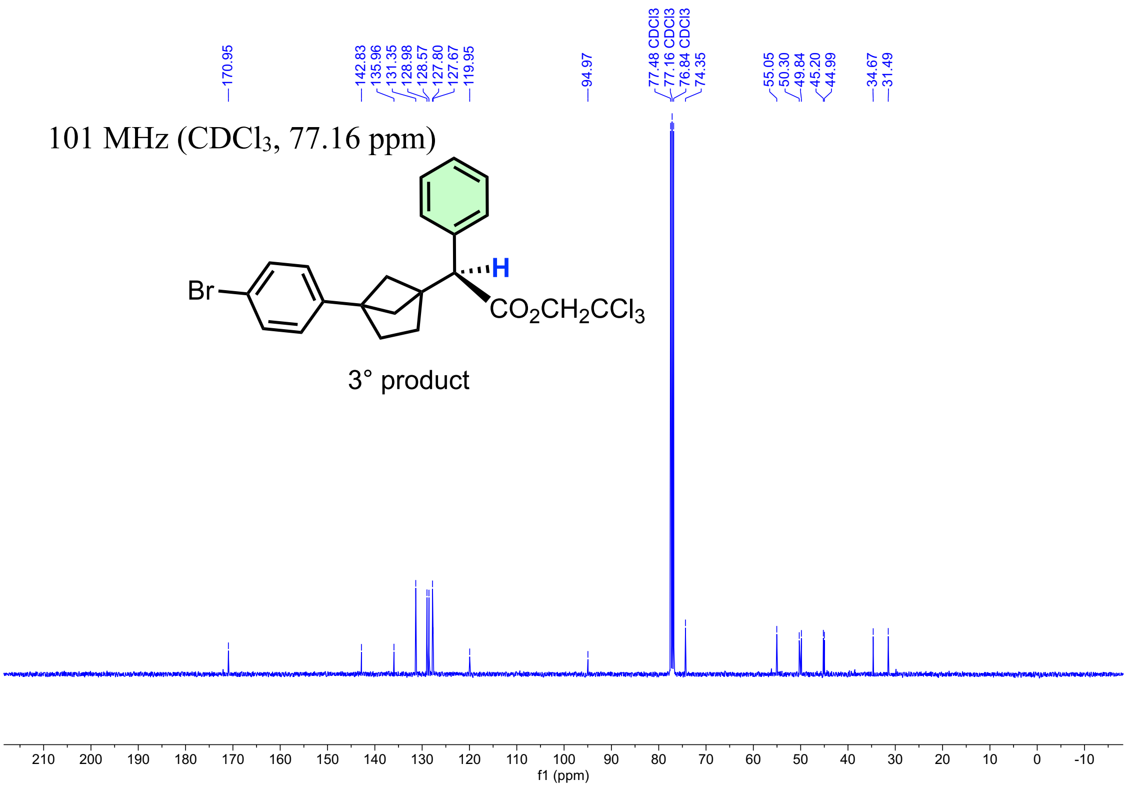

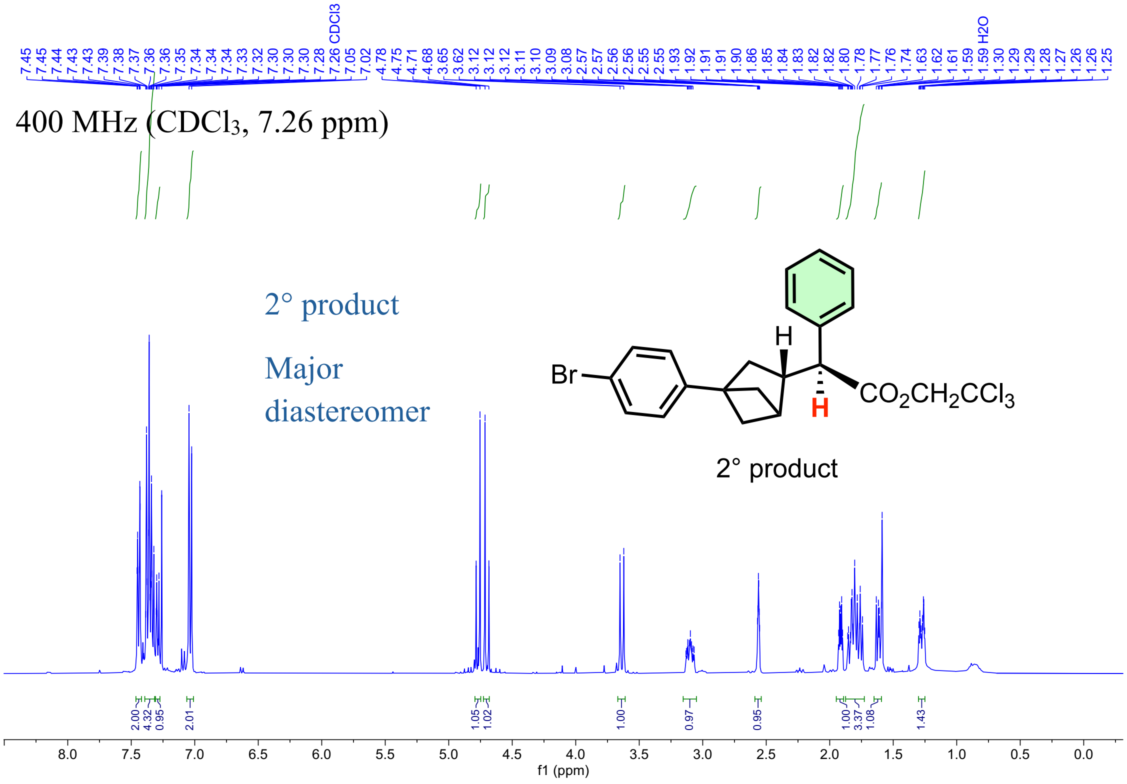
7.45 7.45 7.44 7.43 7.43 7.39 7.38 7.37 7.36 7.36 7.35 7.34 7.34 7.34 7.33 7.32 7.30 7.30 7.30 7.28 7.26 CDCl3 7.05 7.02 4.78 4.75 4.71 4.68 3.65 3.62 3.12 3.12 3.12 3.11 3.10 3.09 3.08 2.57 2.57 2.56 2.56 2.55 2.55 1.93 1.92 1.91 1.91 1.90 1.86 1.85 1.84 1.83 1.82 1.82 1.80 1.78 1.77 1.76 1.74 1.63 1.62 1.61 1.59 1.59 H2O 1.30 1.29 1.29 1.28 1.27 1.26 1.25
400 MHz (CDCl3, 7.26 ppm)
2° product
Major
diastereomer
Br
H
H
CO2CH2CCl3
2° product
2.00 4.32 0.95 2.01 1.05 1.02 1.00 0.97 0.95 1.00 3.37 1.08 1.43
f1 (ppm)

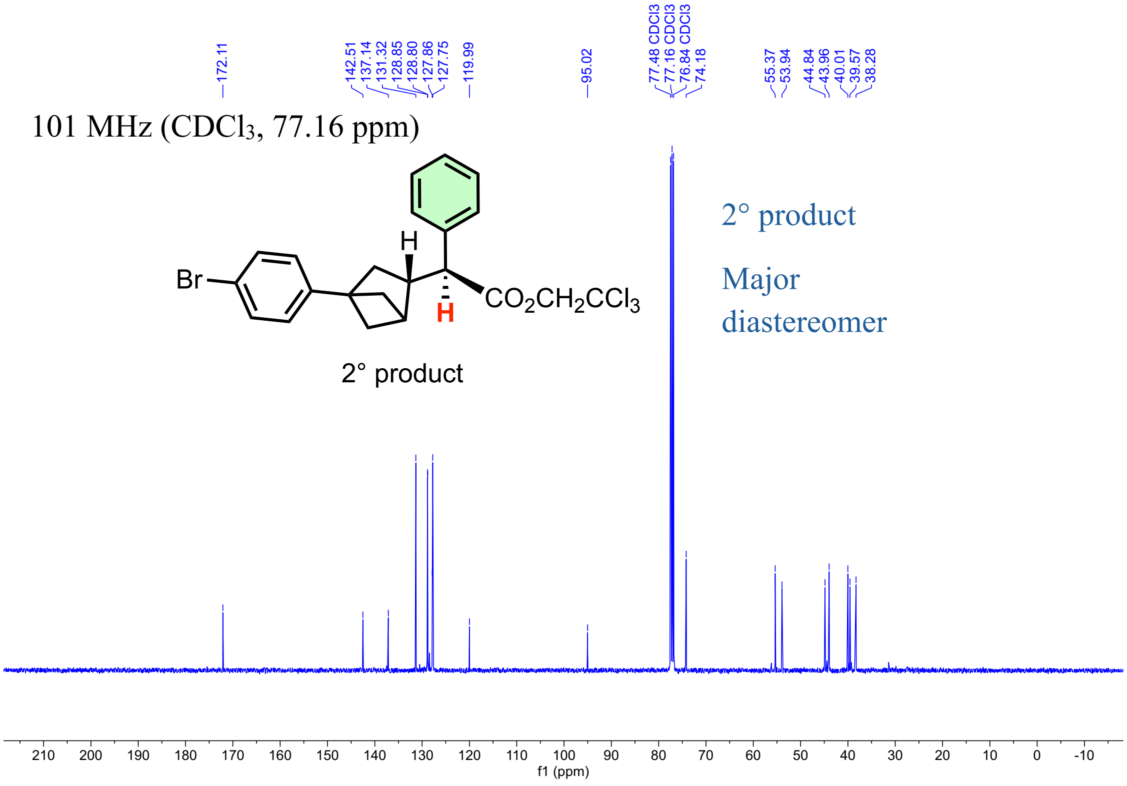
172.11 142.51 137.14 131.32 128.85 128.80 127.86 127.75 119.99 95.02 77.48 CDCl3 77.16 CDCl3 76.84 CDCl3 74.18 55.37 53.94 44.84 43.96 40.01 39.57 38.28
101 MHz (CDCl3, 77.16 ppm)
Br
H
H
CO2CH2CCl3
2° product
2° product
Major
diastereomer
f1 (ppm)

5. NMR Spectra

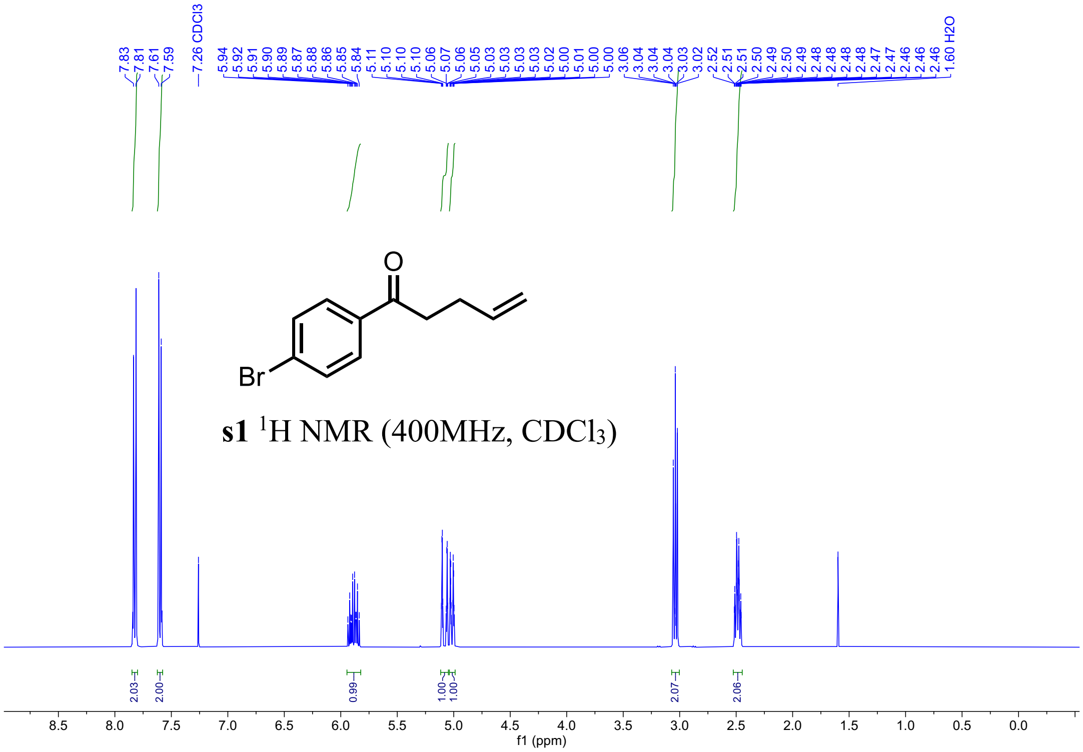

s1 ^{1}H NMR (400MHz, $CDCl_3$)

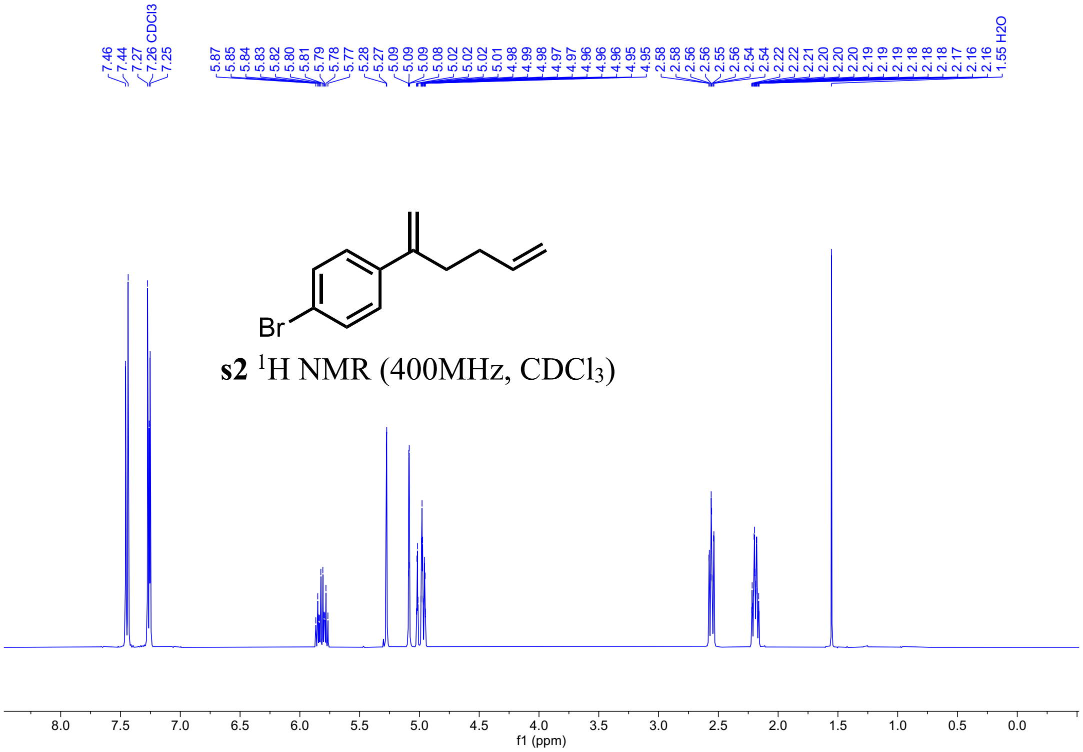

s2 ^{1}H NMR (400MHz, $CDCl_3$)

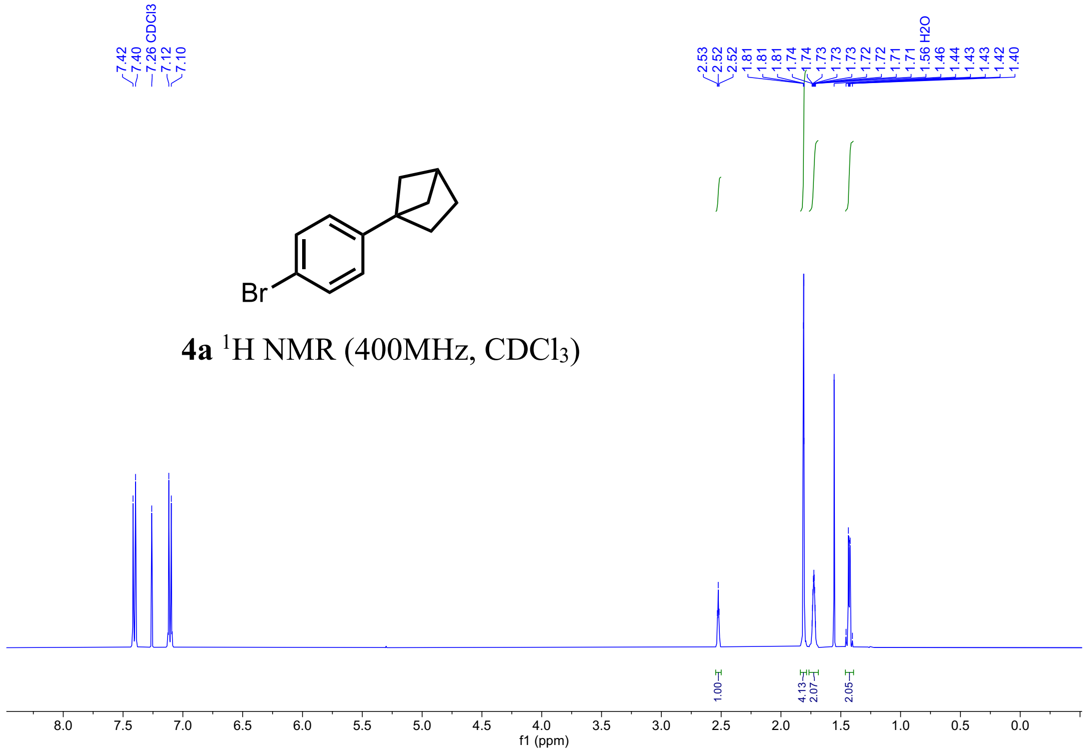
4a ^{1}H NMR (400MHz, $CDCl_3$)
Br
f1 (ppm)

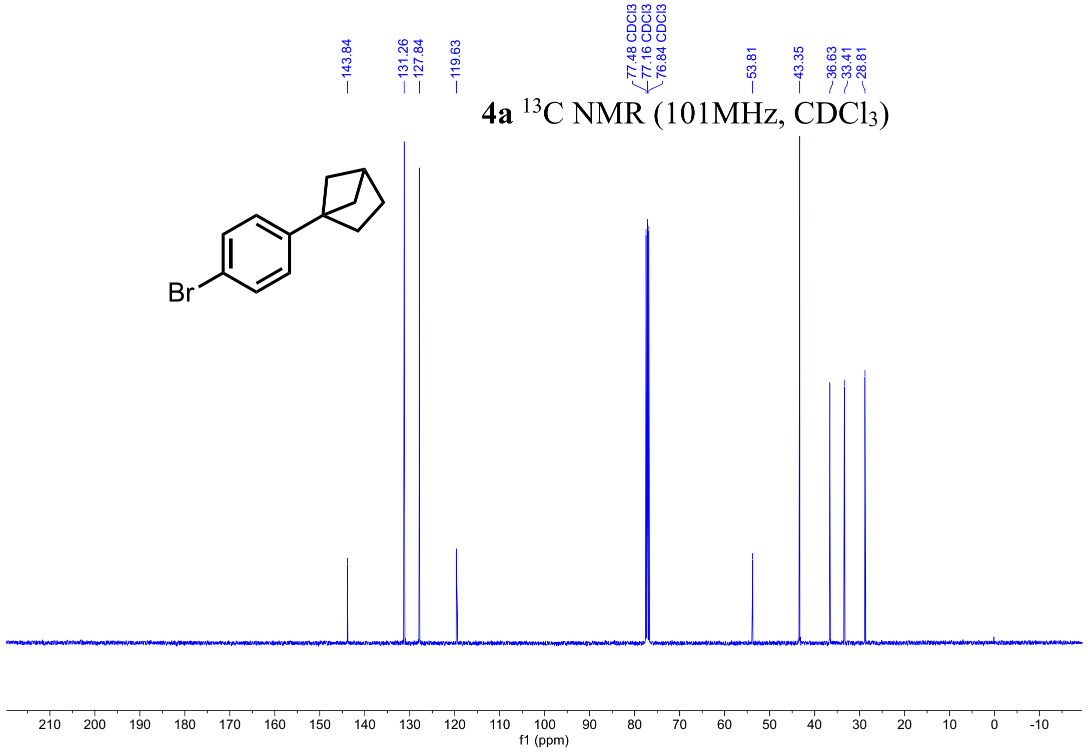
4a ^{13}C NMR (101MHz, $CDCl_3$)
Br
f1 (ppm)

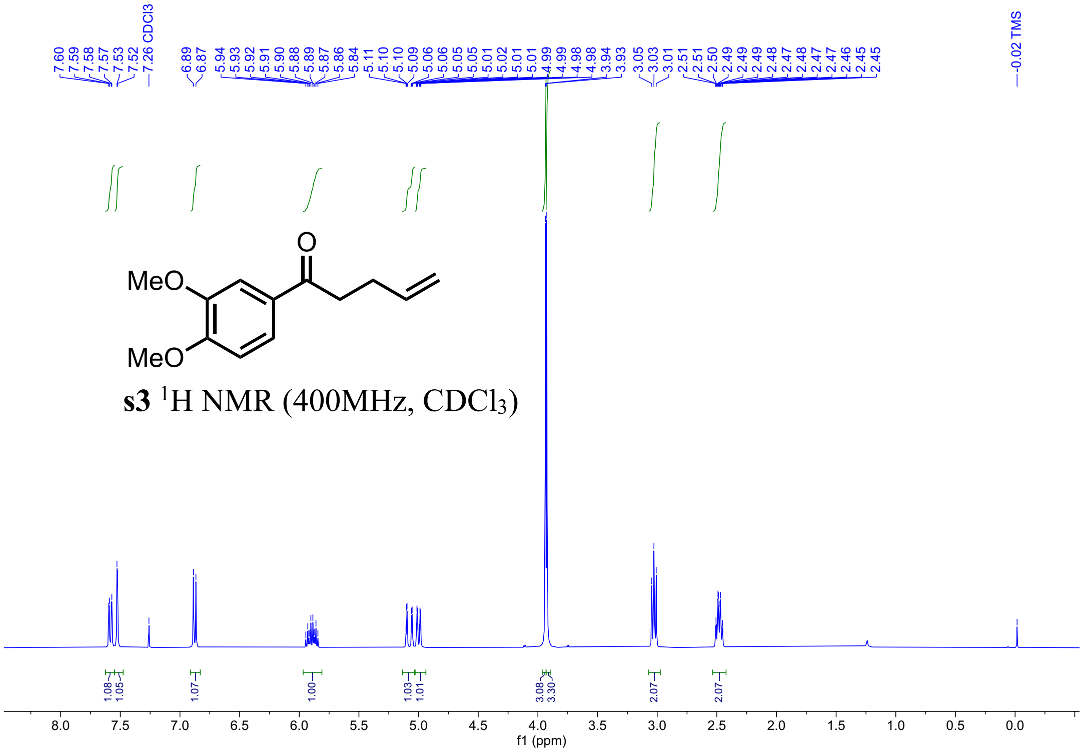

s3 ^{1}H NMR (400MHz, $CDCl_3$)

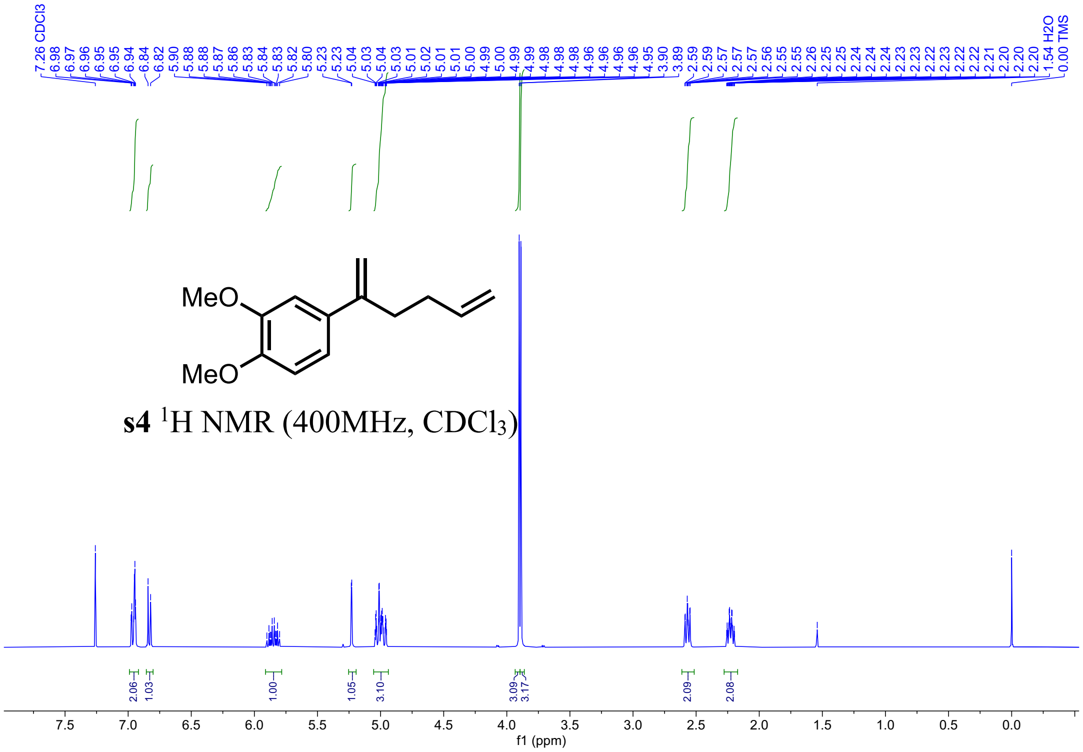

s4 ^{1}H NMR (400MHz, $CDCl_3$)

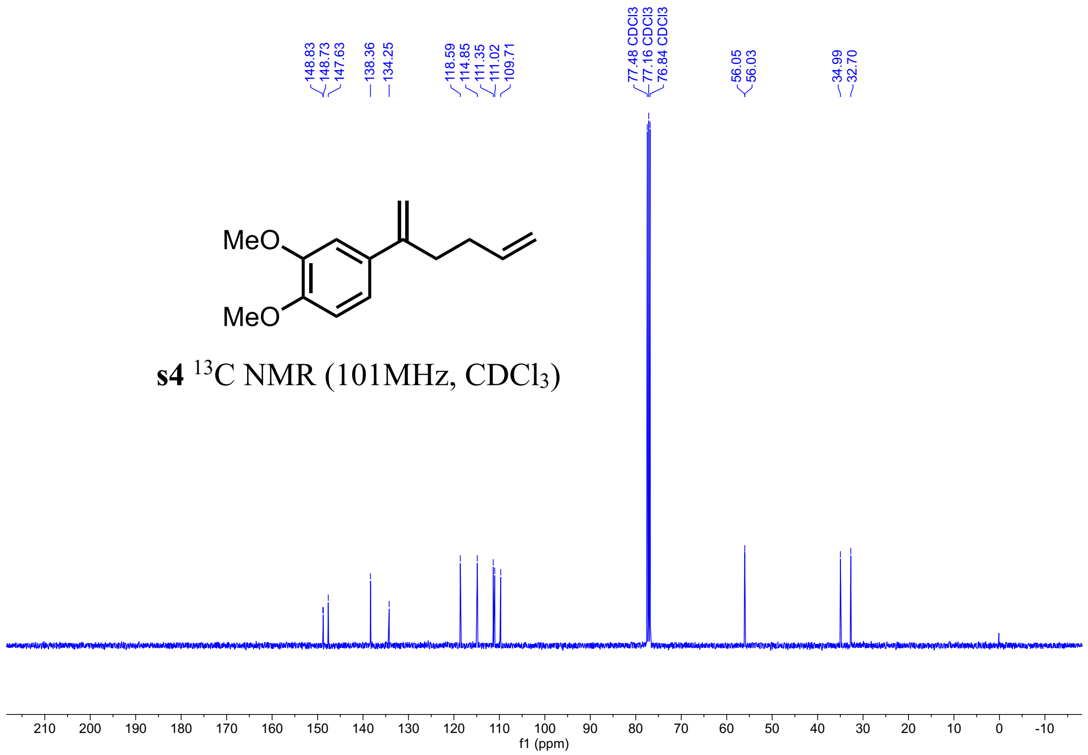
148.83
148.73
147.63
138.36
134.25
118.59
114.85
111.35
111.02
109.71
77.48 CDCl3
77.16 CDCl3
76.84 CDCl3
56.05
56.03
34.99
32.70
MeO
MeO
s4 13C NMR (101MHz, CDCl3)
f1 (ppm)

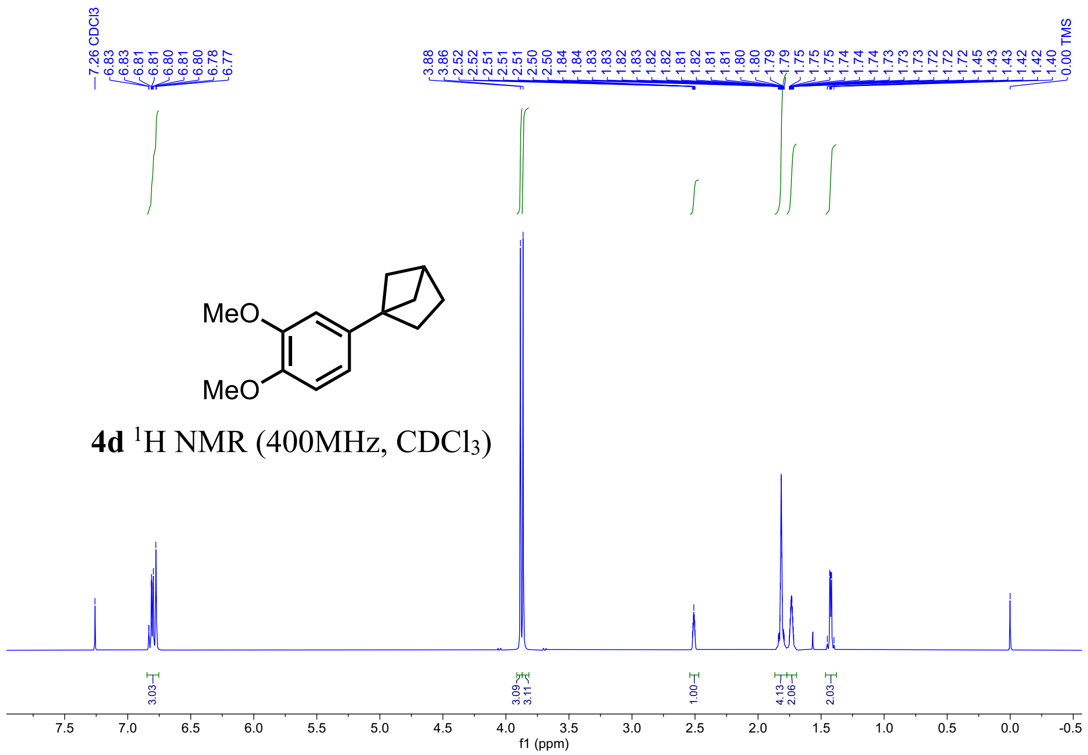
7.26 CDCl3
6.83
6.83
6.81
6.81
6.80
6.81
6.80
6.78
6.77
3.88
3.86
2.52
2.52
2.51
2.51
2.50
2.50
1.84
1.84
1.83
1.83
1.82
1.83
1.82
1.82
1.81
1.82
1.81
1.81
1.80
1.80
1.79
1.75
1.75
1.75
1.74
1.74
1.74
1.73
1.73
1.73
1.72
1.72
1.72
1.45
1.43
1.42
1.42
1.40
0.00 TMS
MeO
MeO
4d 1H NMR (400MHz, CDCl3)
3.03
3.09
3.11
1.00
4.13
2.06
2.03
f1 (ppm)

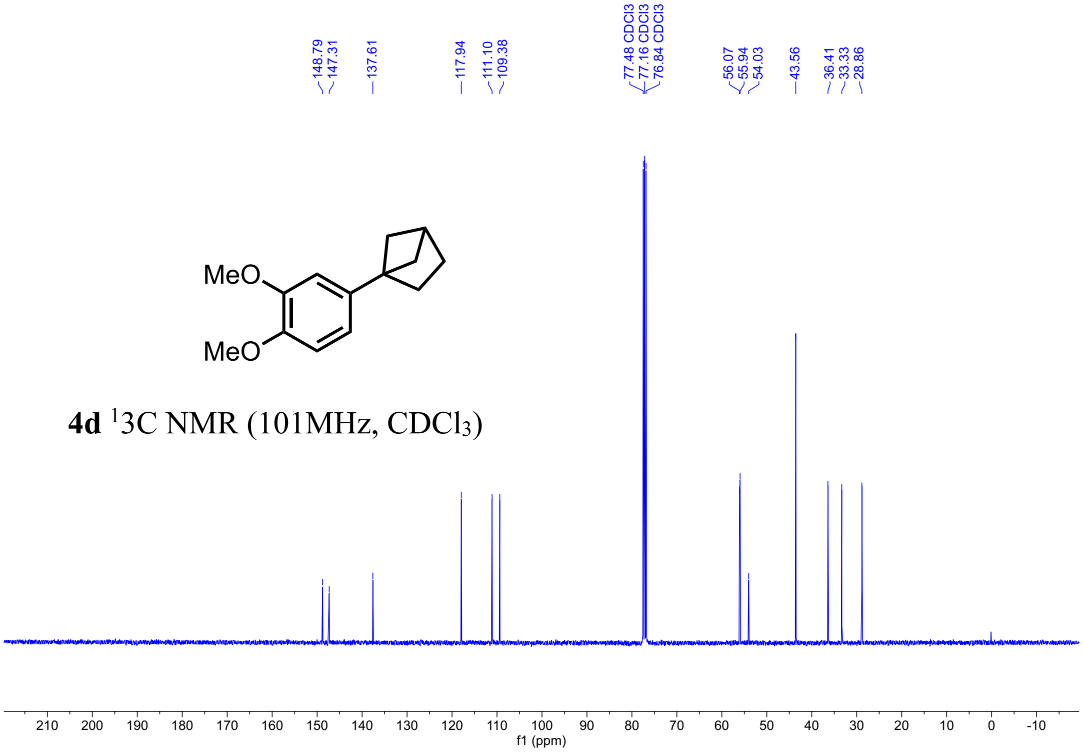

4d 13C NMR (101MHz, $CDCl_3$)

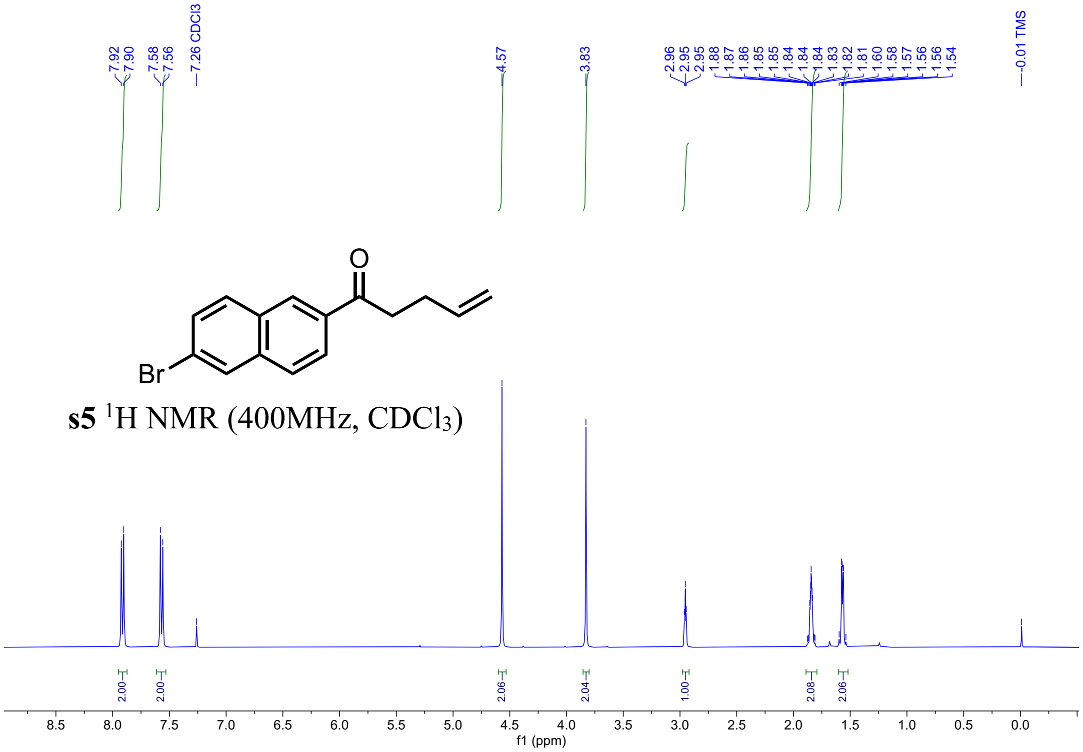

s5 ^{1}H NMR (400MHz, $CDCl_3$)

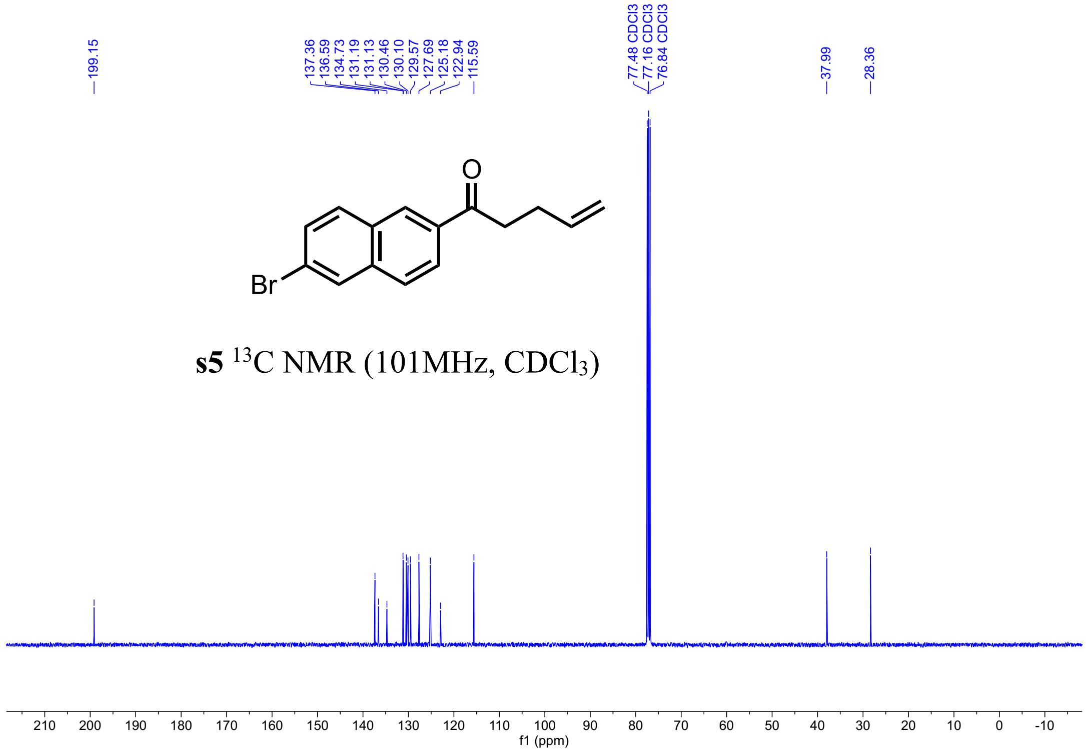
199.15
137.36
136.59
134.73
131.19
131.13
130.46
130.10
129.57
127.69
125.18
122.94
115.59
77.48 CDCl3
77.16 CDCl3
76.84 CDCl3
37.99
28.36
O
Br
s5 ^{13}C NMR (101MHz, CDCl$_3$)
f1 (ppm)

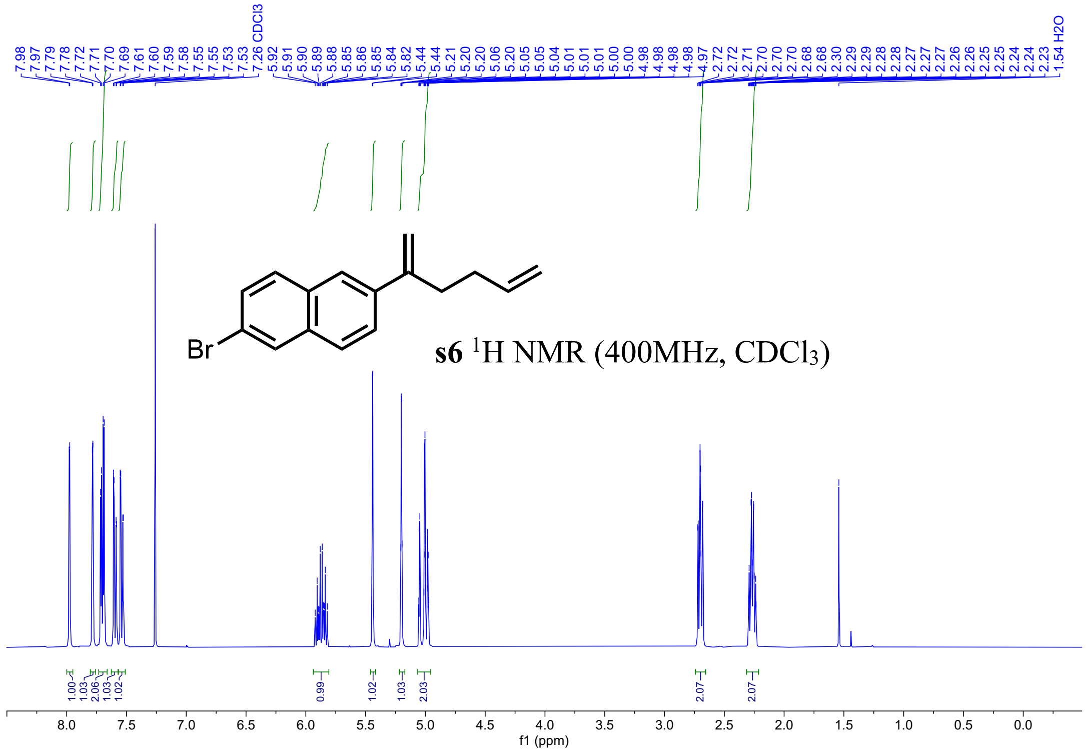
7.98 7.97 7.79 7.78 7.72 7.71 7.70 7.69 7.61 7.60 7.59 7.58 7.55 7.55 7.53 7.53 7.26 CDCl3
5.92 5.91 5.90 5.89 5.88 5.86 5.85 5.85 5.84 5.82 5.44 5.44 5.21 5.20 5.20 5.20 5.06 5.05 5.04 5.01 5.01 5.00 4.98 4.98 4.98 4.97
2.72 2.72 2.71 2.70 2.70 2.70 2.68 2.68 2.30 2.29 2.29 2.28 2.28 2.27 2.27 2.26 2.26 2.25 2.25 2.24 2.24 2.23 1.54 H2O
Br
s6 ^{1}H NMR (400MHz, CDCl$_3$)
1.00 1.03 2.06 1.03 1.02 0.99 1.02 1.03 2.03 2.07 2.07
f1 (ppm)

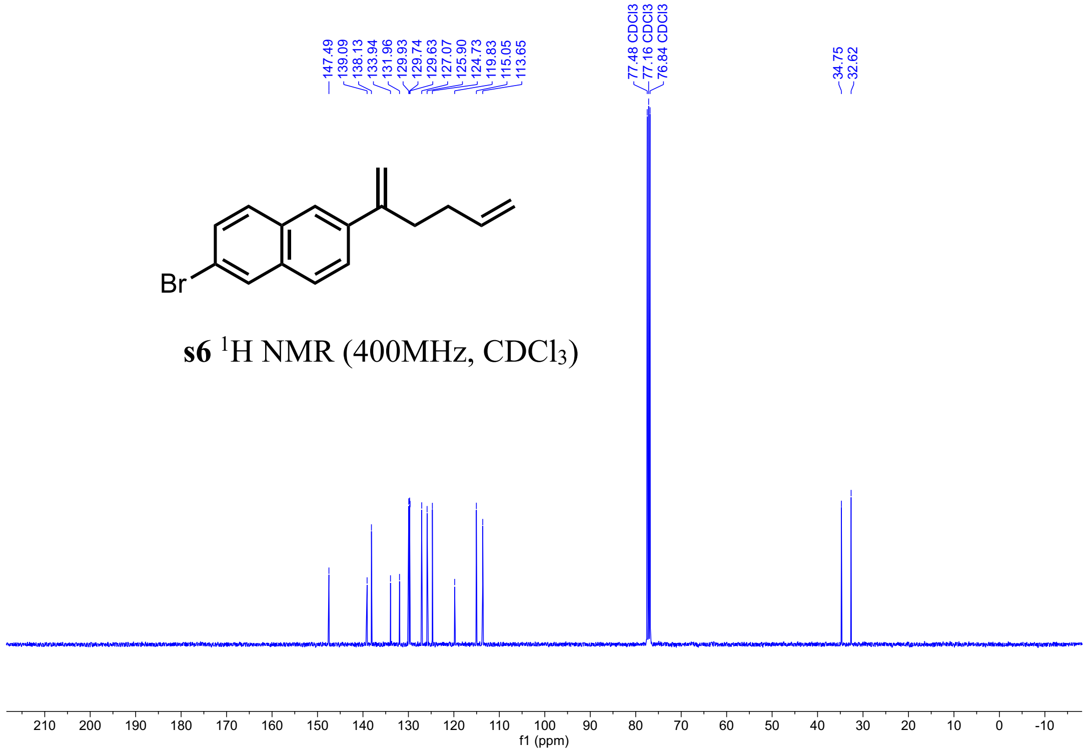

s6 ^{1}H NMR (400MHz, $CDCl_3$)

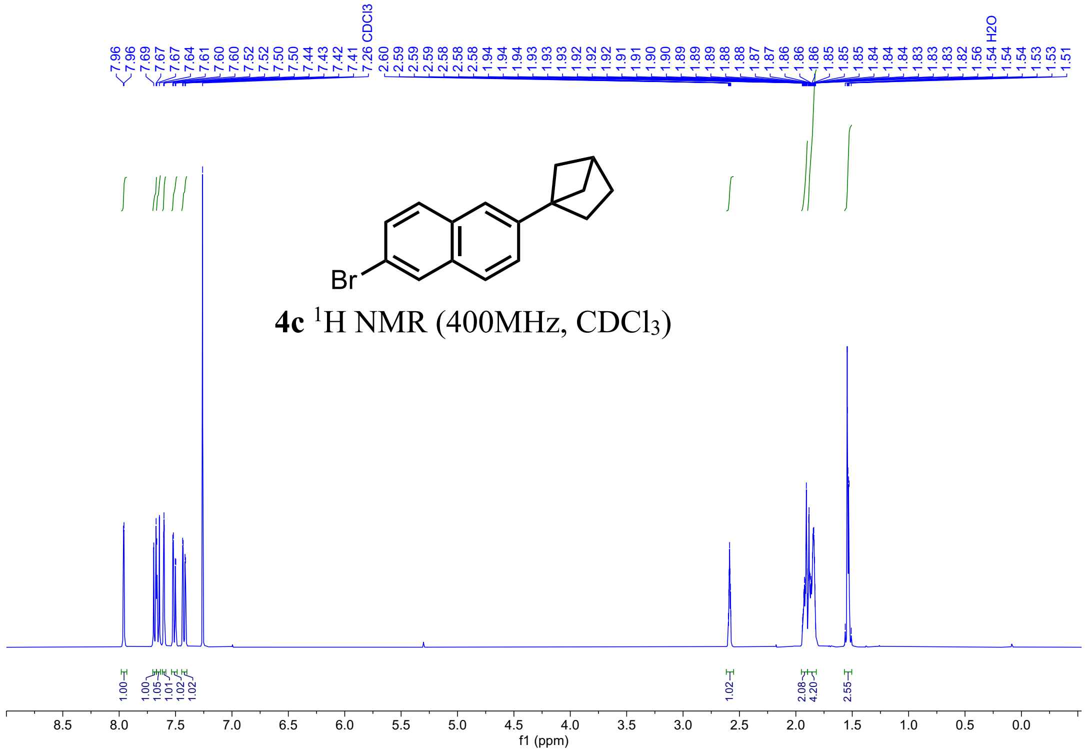

4c ^{1}H NMR (400MHz, $CDCl_3$)

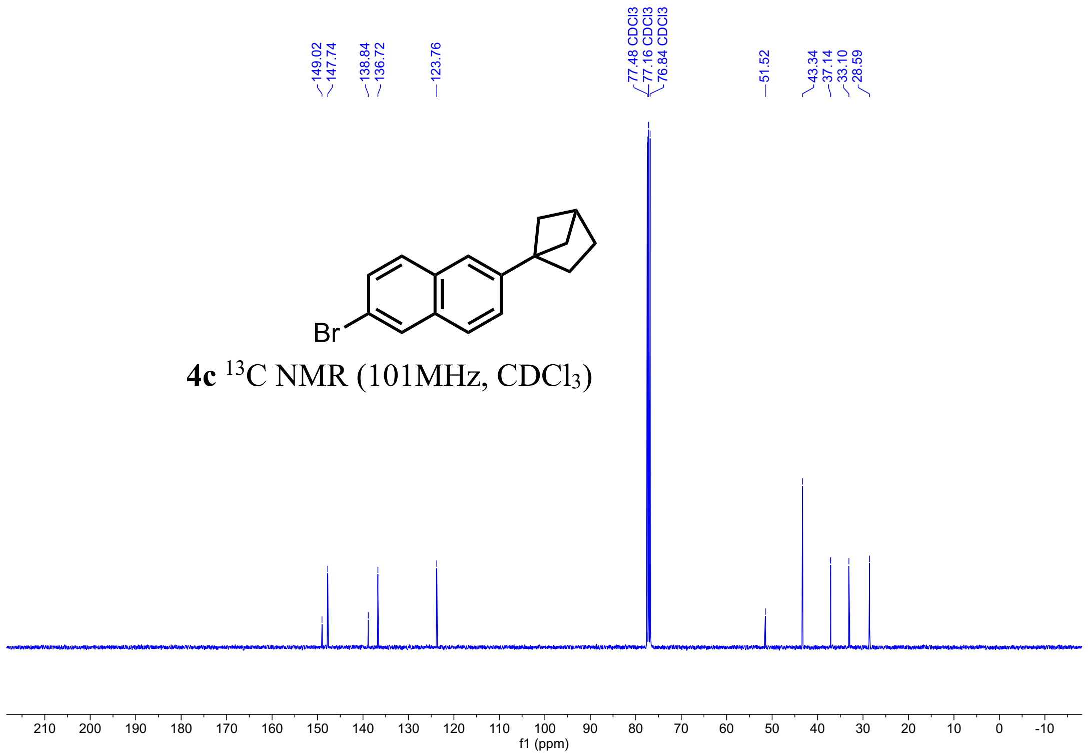
149.02
147.74
138.84
136.72
123.76
77.48 CDCl3
77.16 CDCl3
76.84 CDCl3
51.52
43.34
37.14
33.10
28.59
Br
4c 13C NMR (101MHz, CDCl3)
f1 (ppm)

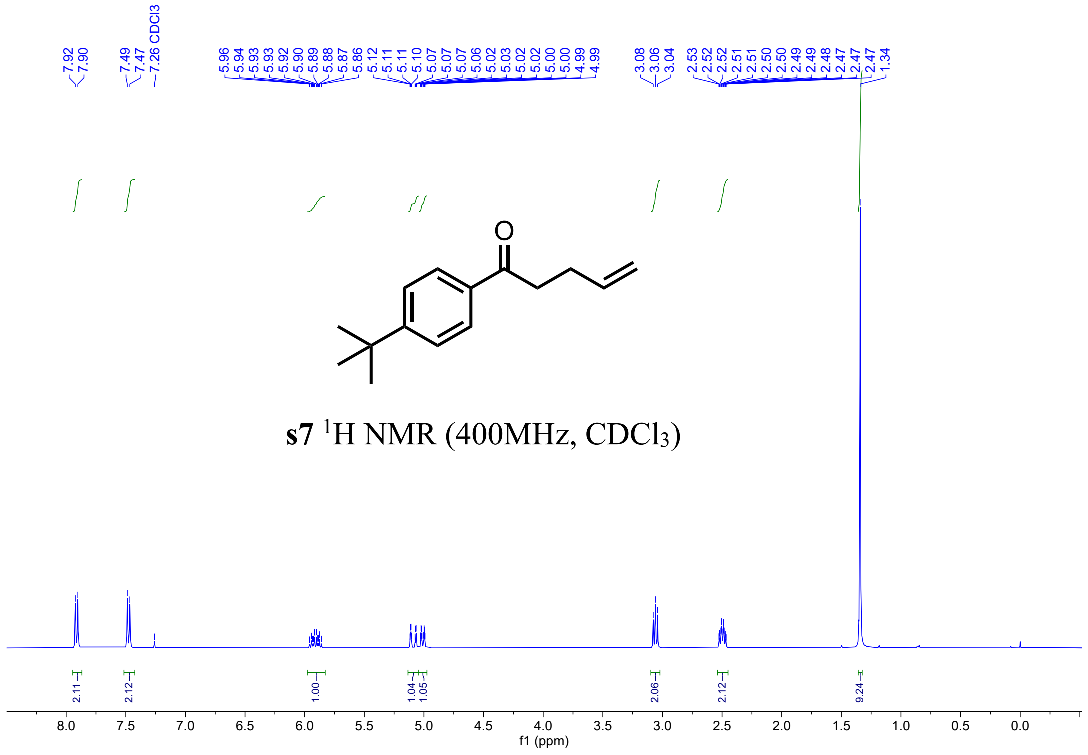
7.92
7.90
7.49
7.47
7.26 CDCl3
1.34
O
s7 1H NMR (400MHz, CDCl3)
2.11
2.12
1.00
1.04
1.05
2.06
2.12
9.24
f1 (ppm)

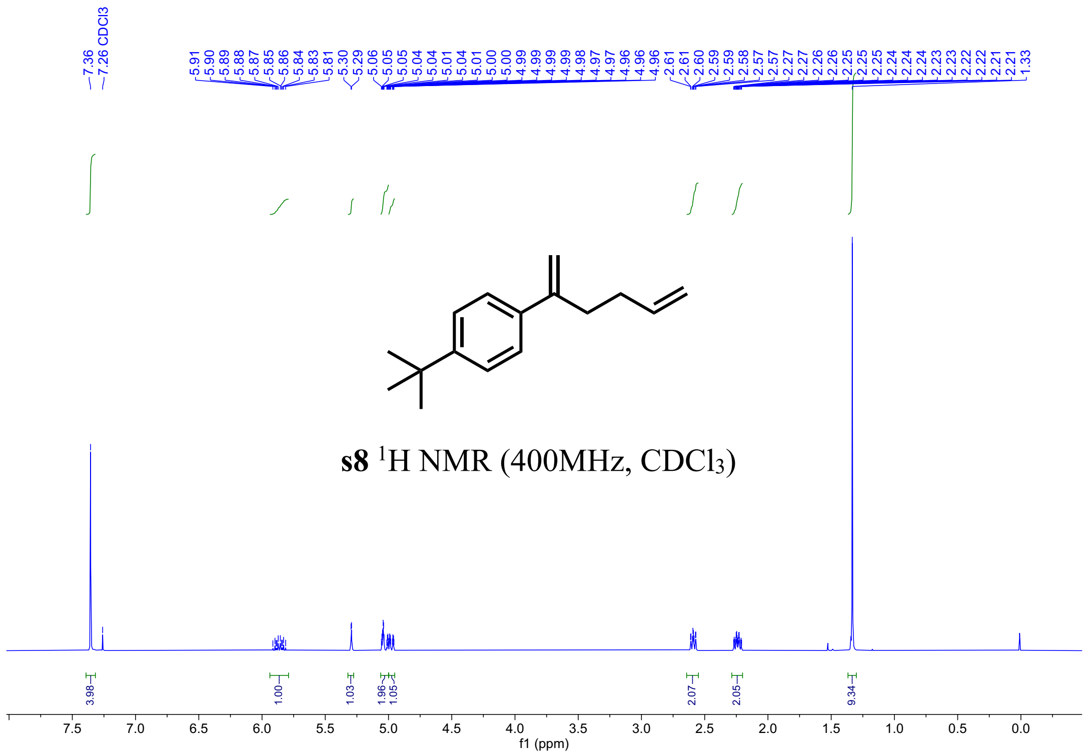

s8 ^{1}H NMR (400MHz, $CDCl_3$)

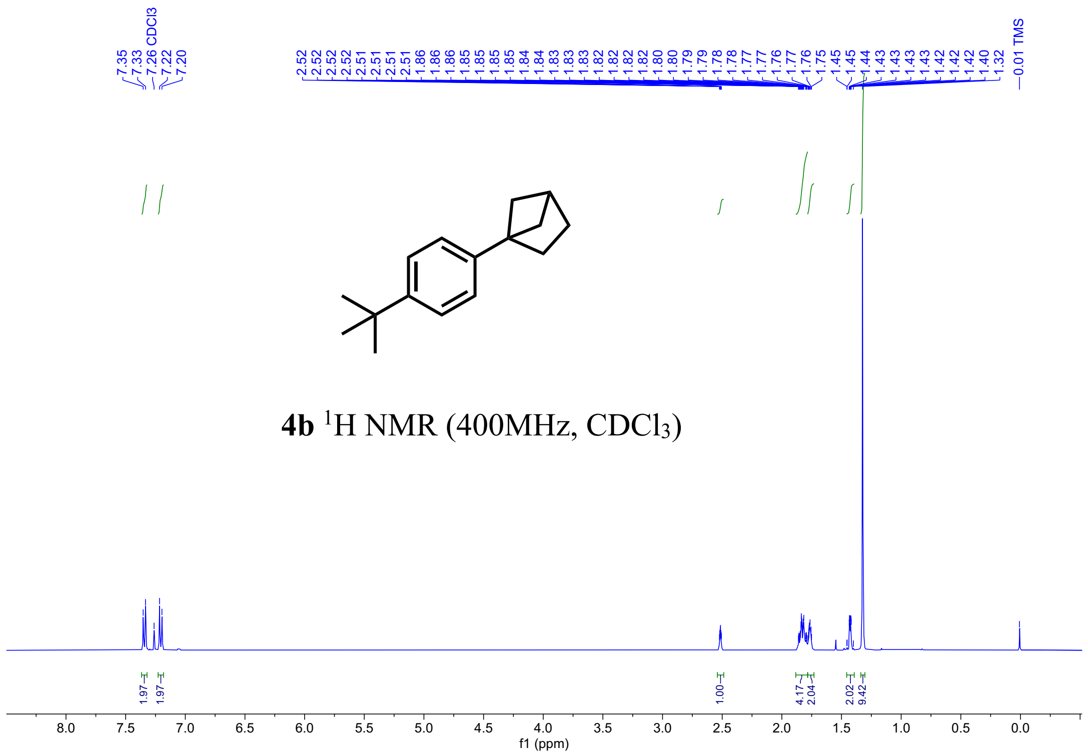

4b ^{1}H NMR (400MHz, $CDCl_3$)

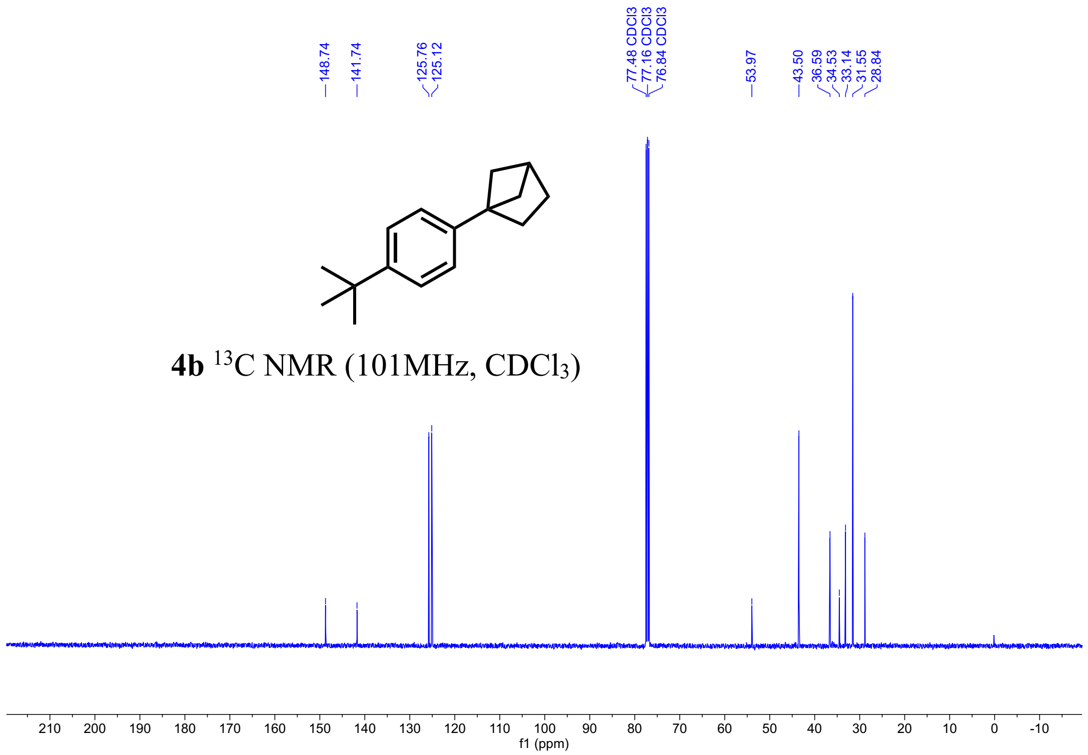

4b ^{13}C NMR (101MHz, $CDCl_3$)

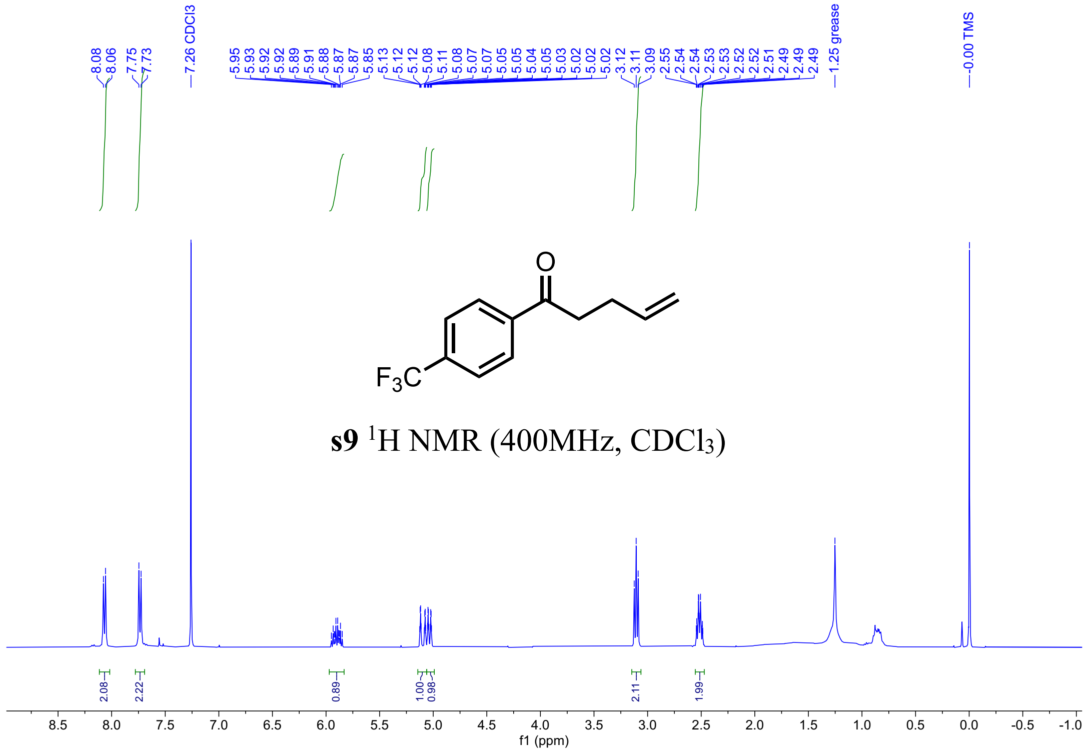

s9 ^{1}H NMR (400MHz, $CDCl_3$)

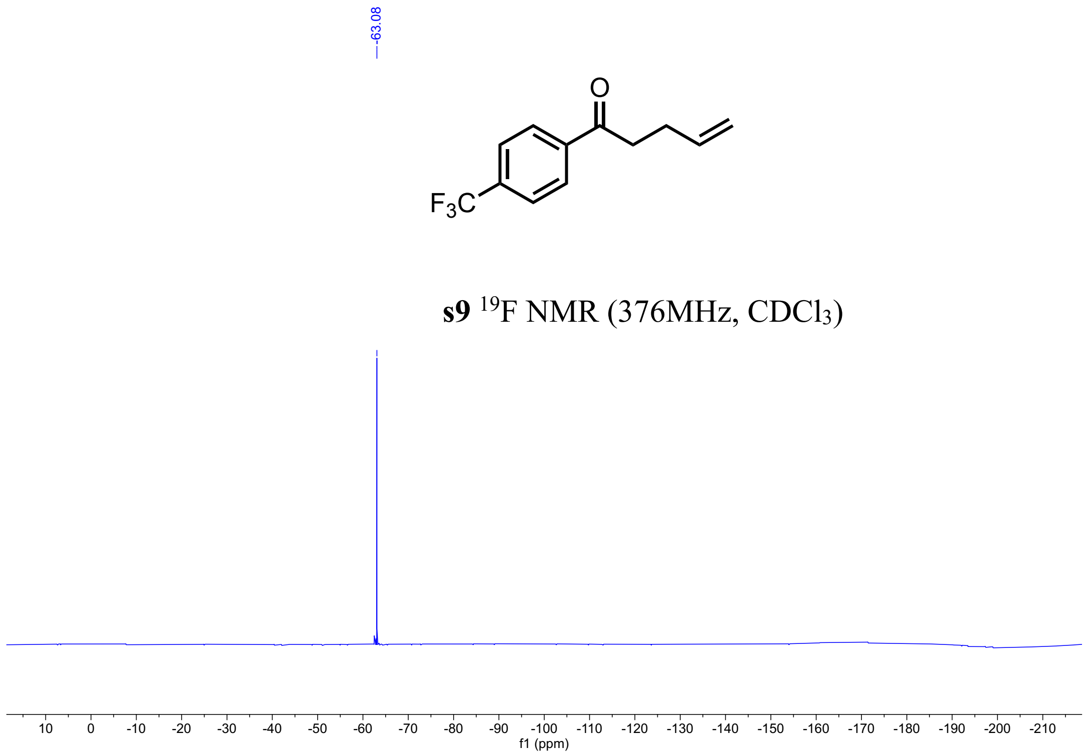

s9 ^{19}F NMR (376MHz, $CDCl_3$)

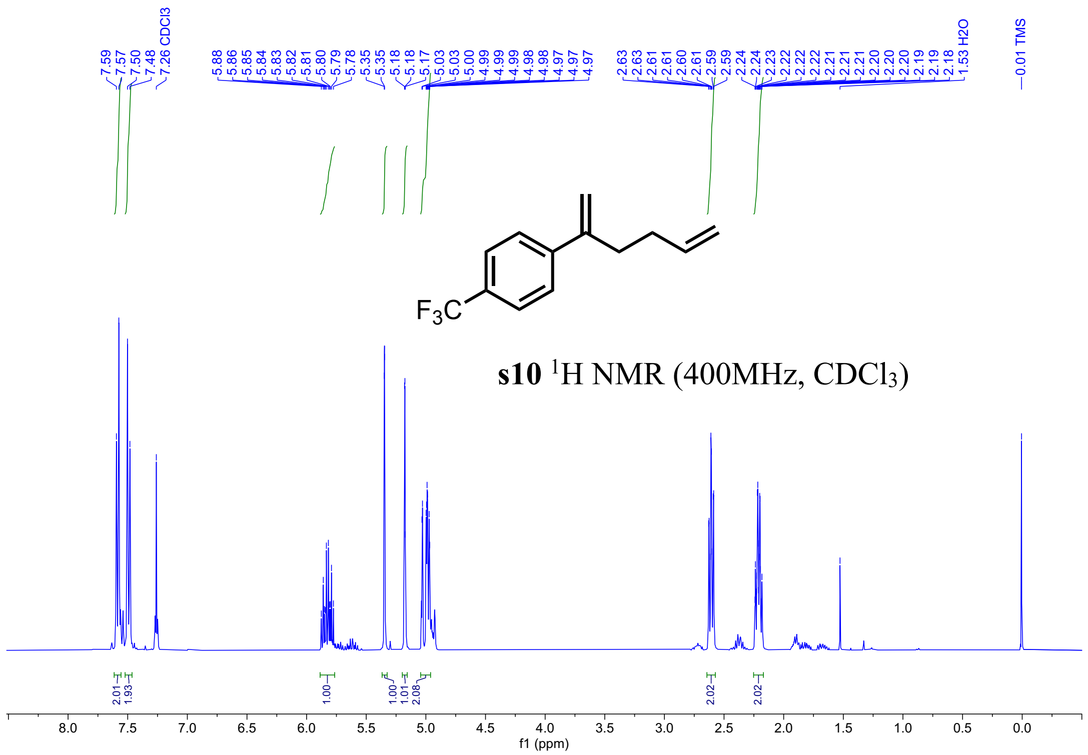

s10 ^{1}H NMR (400MHz, $CDCl_3$)

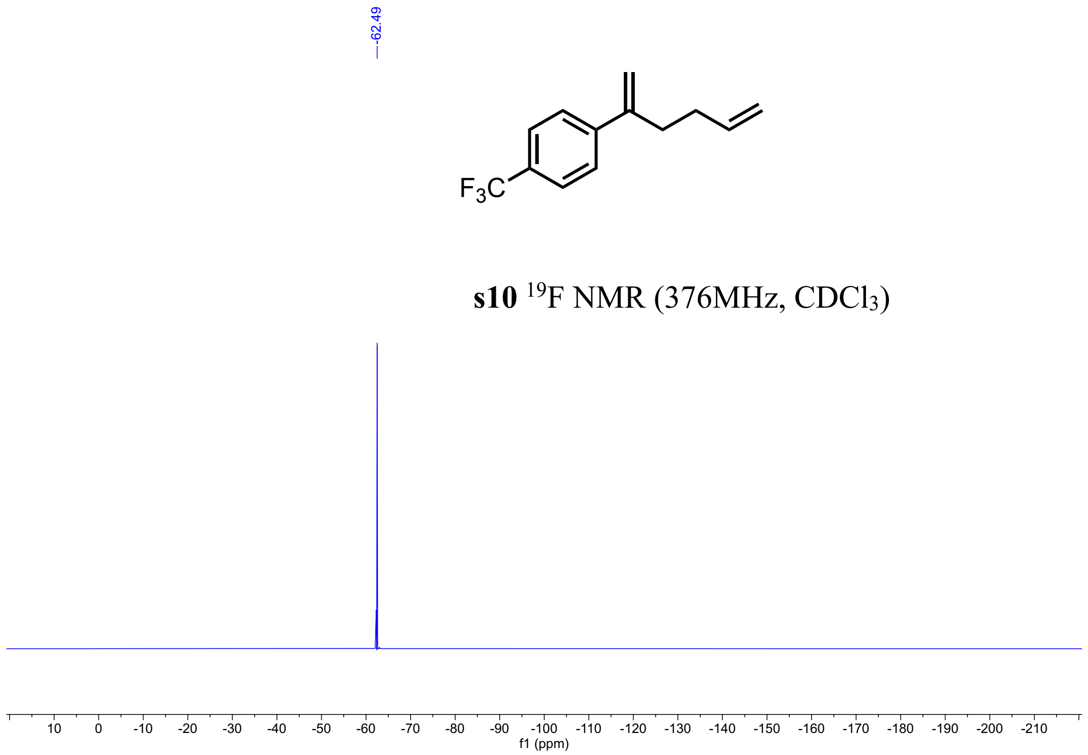

s10 ^{19}F NMR (376MHz, $CDCl_3$)

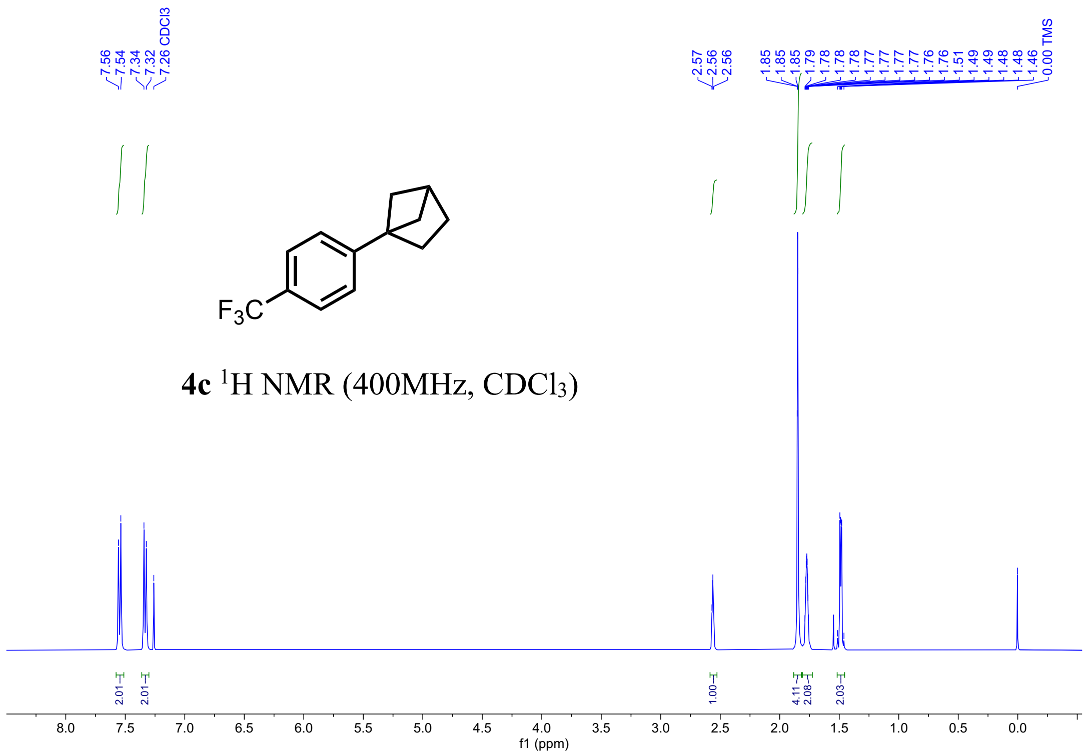

4c ^{1}H NMR (400MHz, $CDCl_3$)

4c ^{13}C NMR (101MHz, $CDCl_3$)

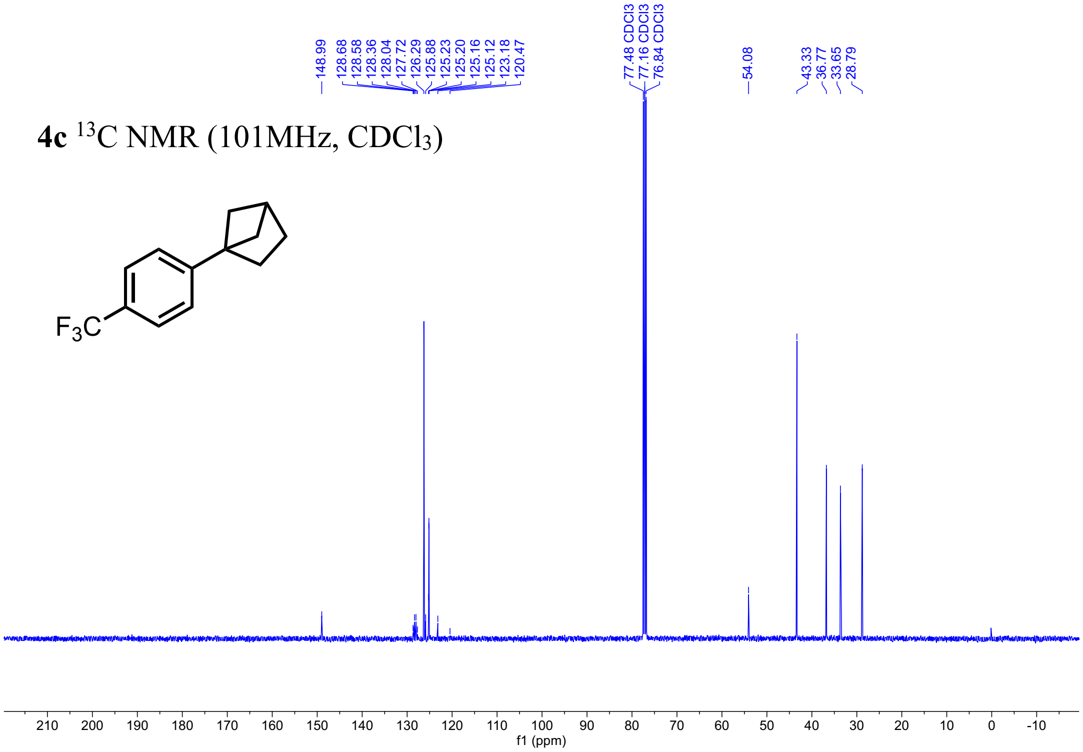

4c ^{19}F NMR (376MHz, $CDCl_3$)

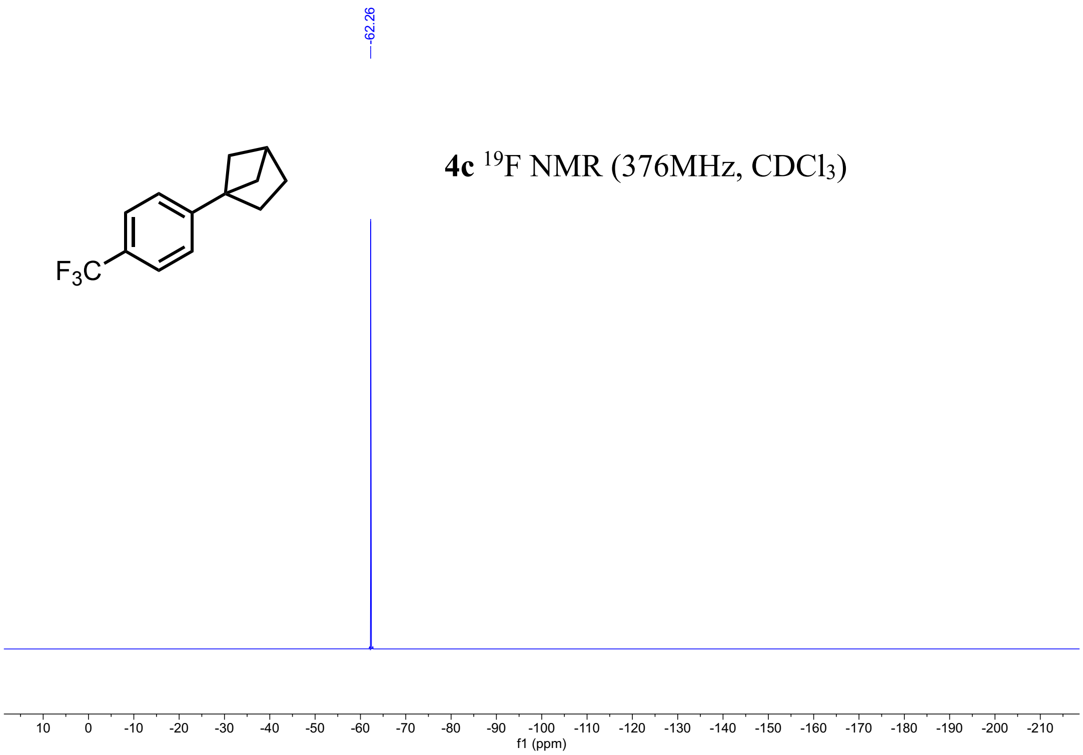

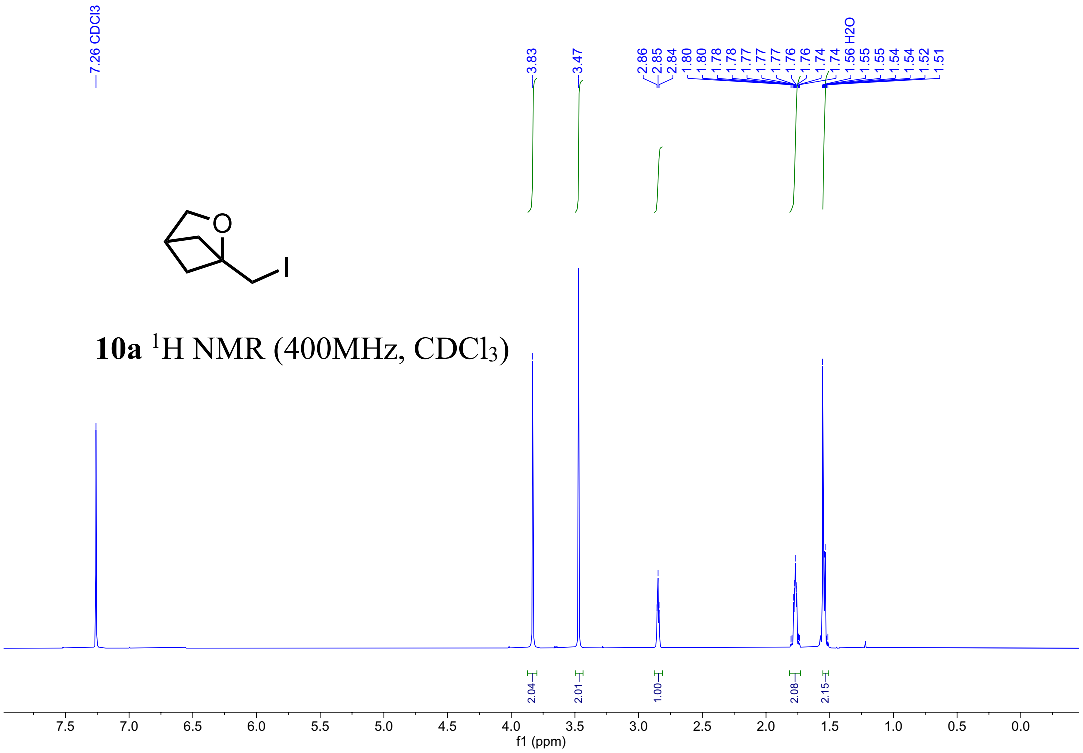

10a ^{1}H NMR (400MHz, $CDCl_3$)

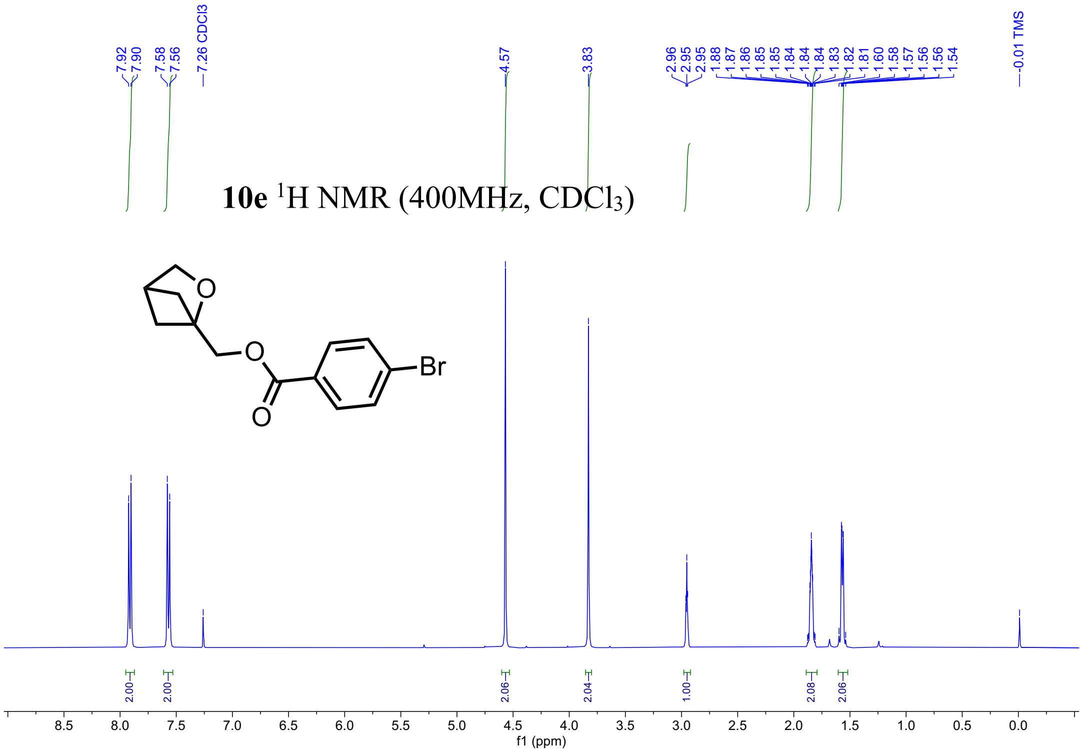

10e ^{1}H NMR (400MHz, $CDCl_3$)

10e ^{13}C NMR (101MHz, $CDCl_3$)

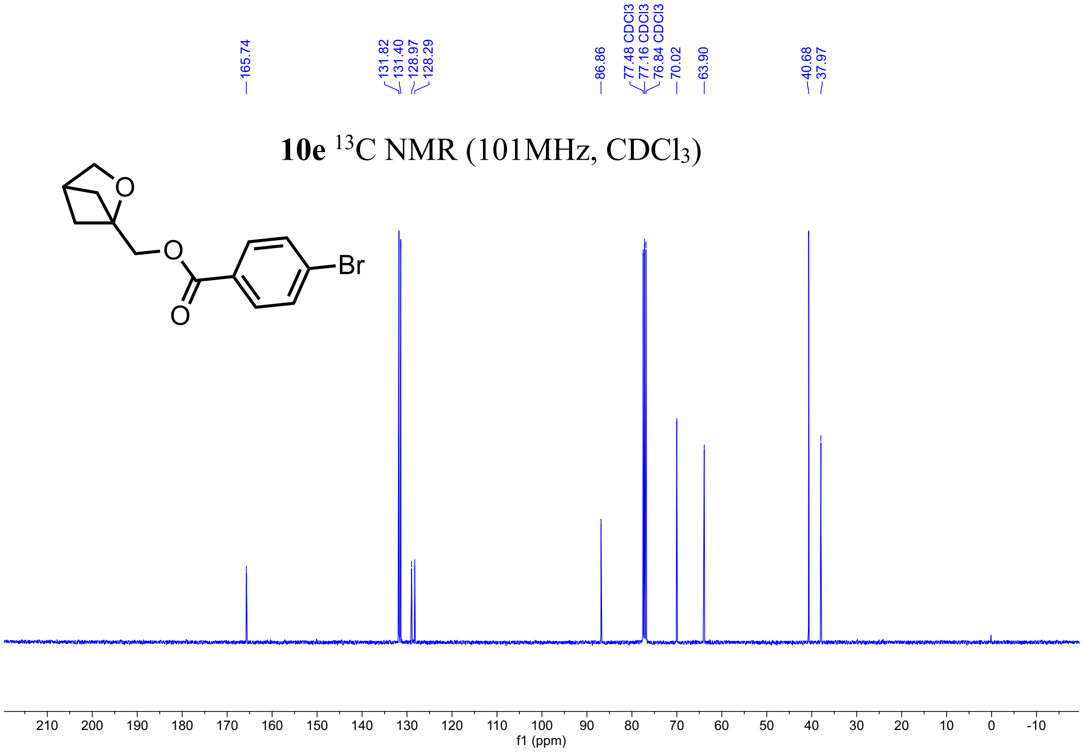

10f ^{1}H NMR (400MHz, $CDCl_3$)

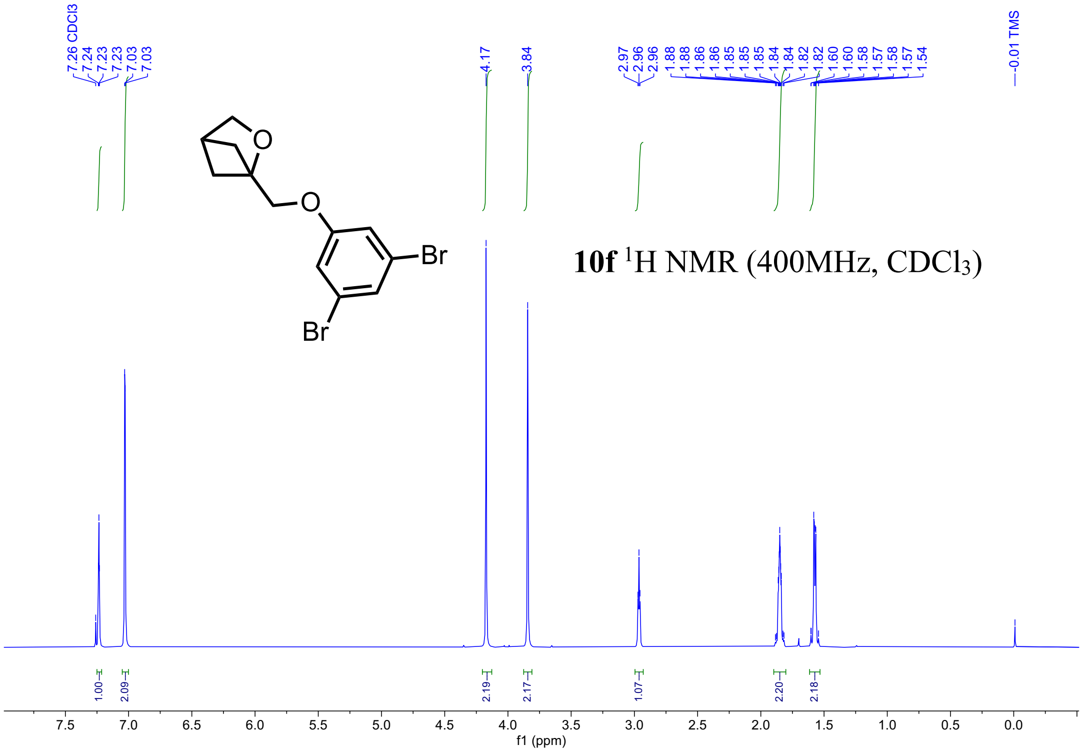

10f ^{13}C NMR (101MHz, CDCl$_3$)

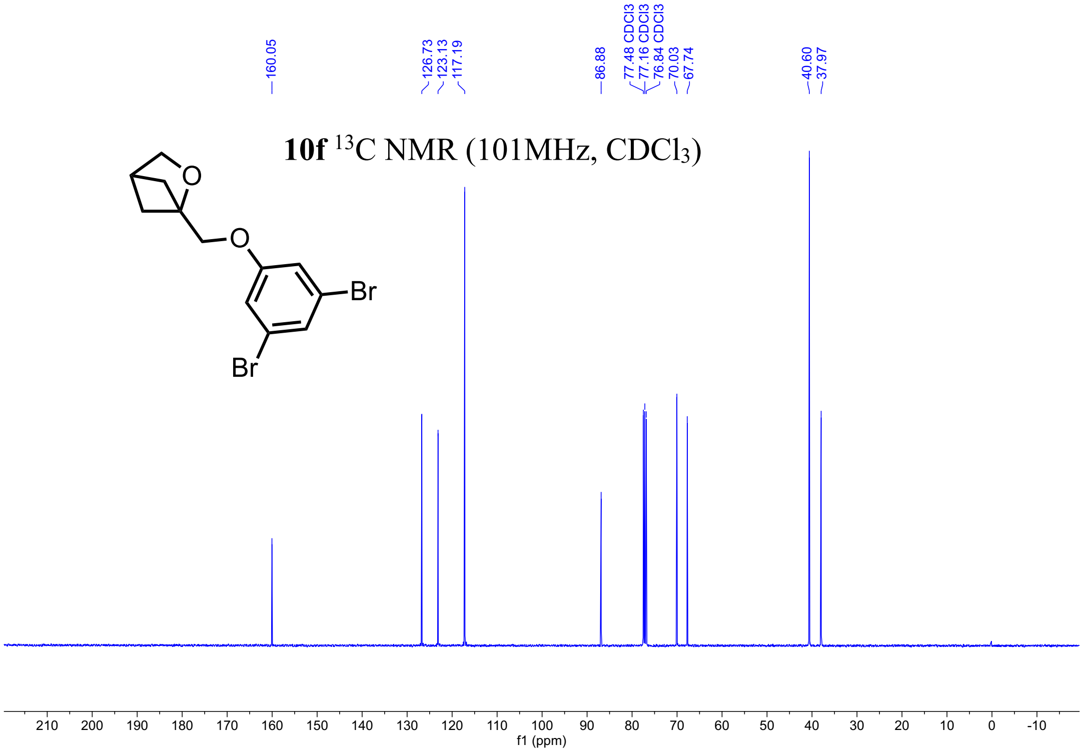

10g ^{1}H NMR (400MHz, CDCl$_3$)

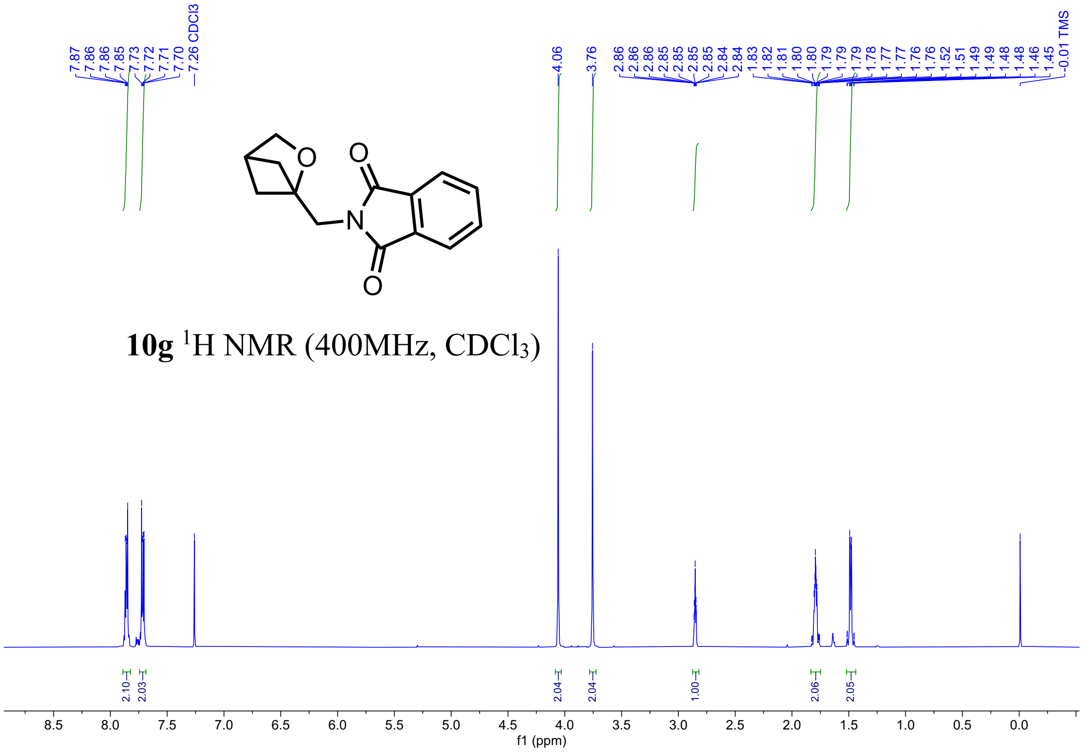

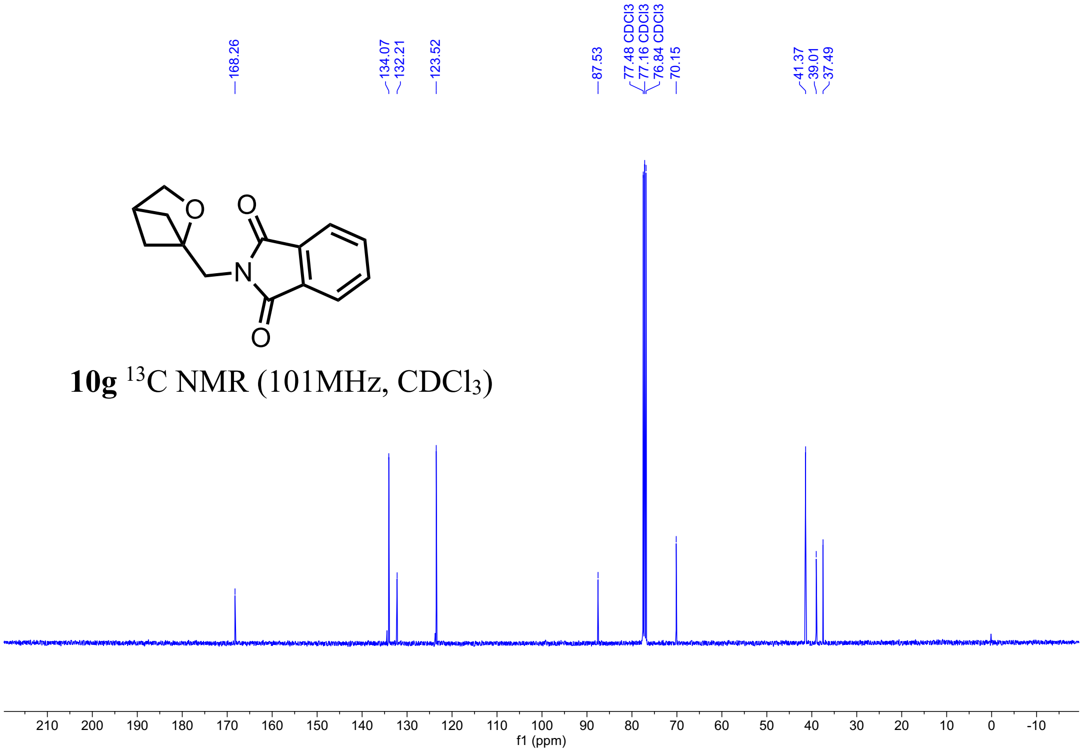
10g ^{13}C NMR (101MHz, $CDCl_3$)
f1 (ppm)

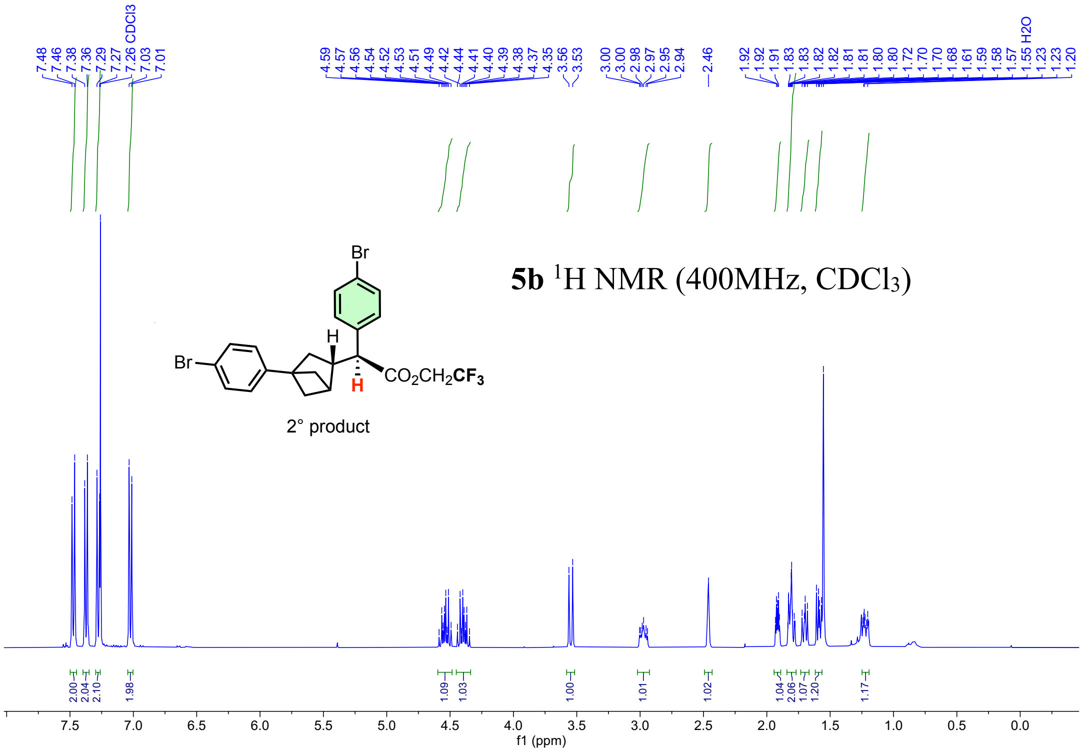
5b ^{1}H NMR (400MHz, $CDCl_3$)
2° product
f1 (ppm)

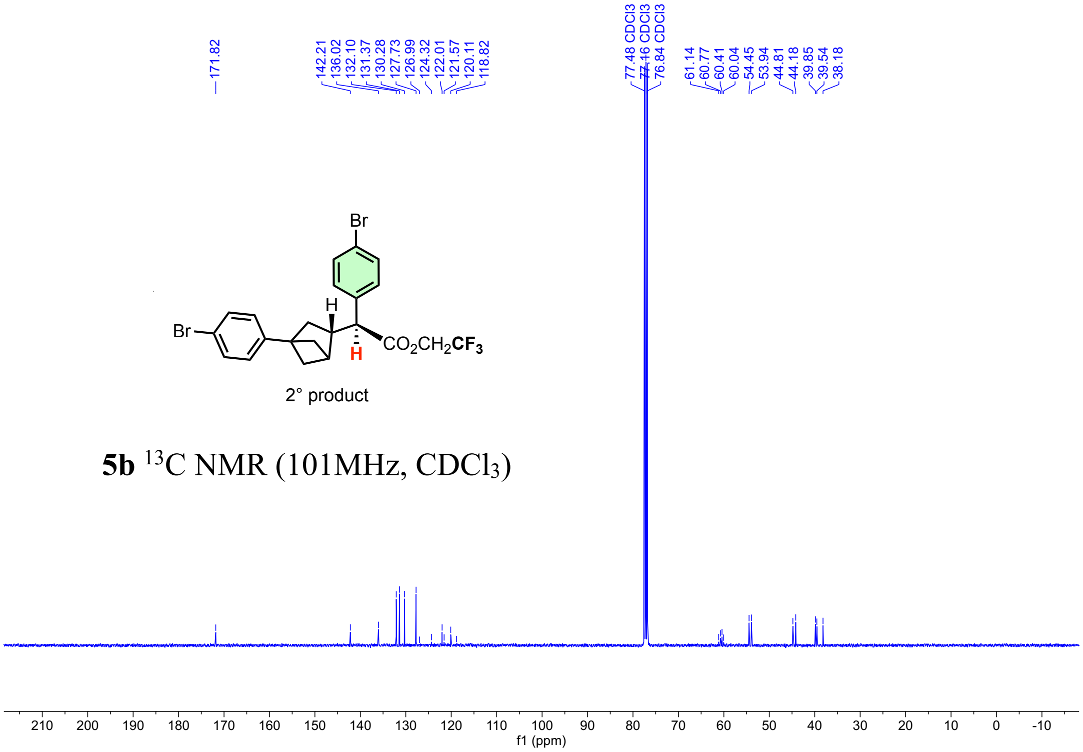

5b ^{13}C NMR (101MHz, $CDCl_3$)

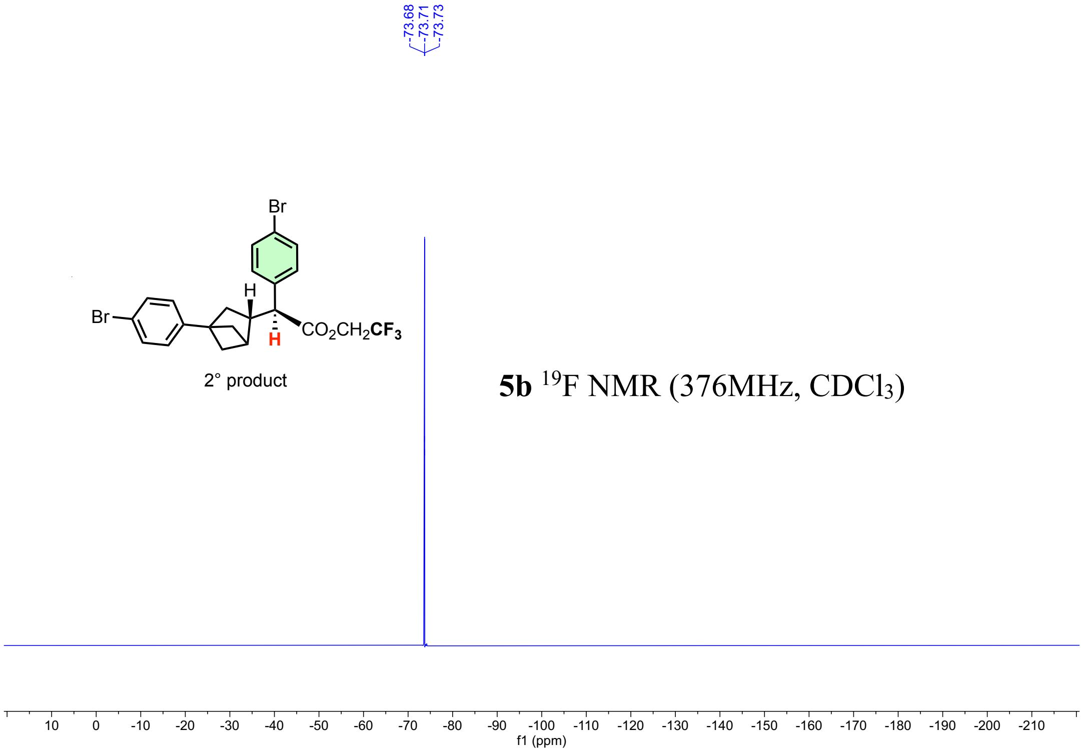

5b ^{19}F NMR (376MHz, $CDCl_3$)

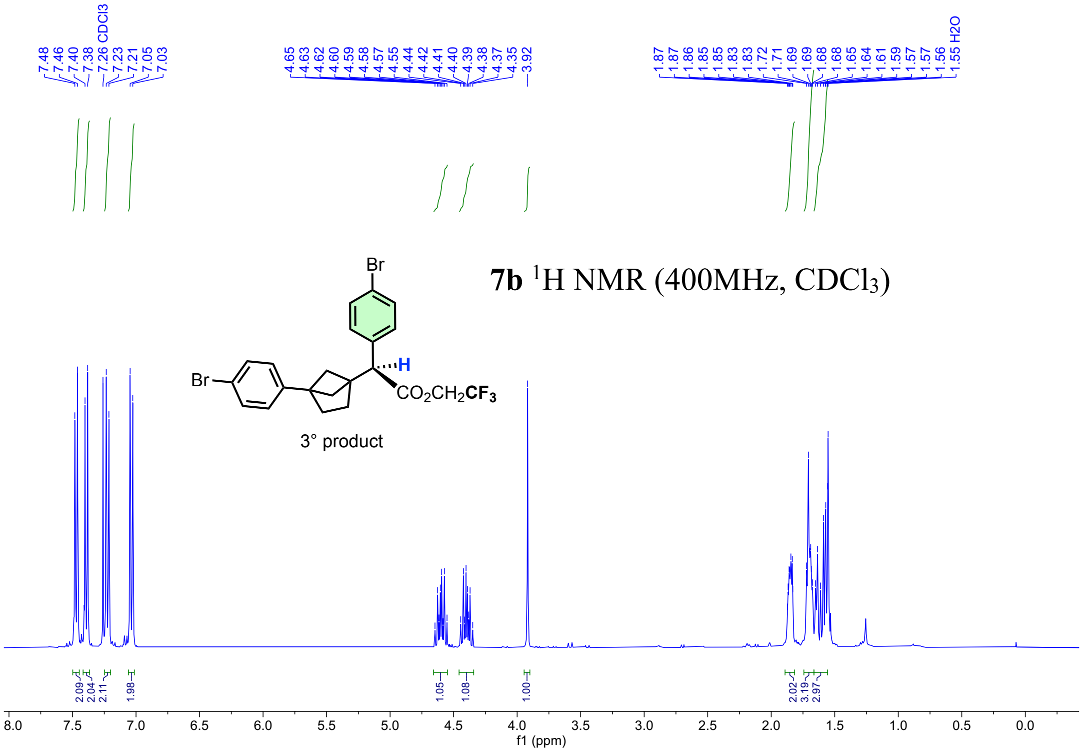

7b ^{1}H NMR (400MHz, $CDCl_3$)

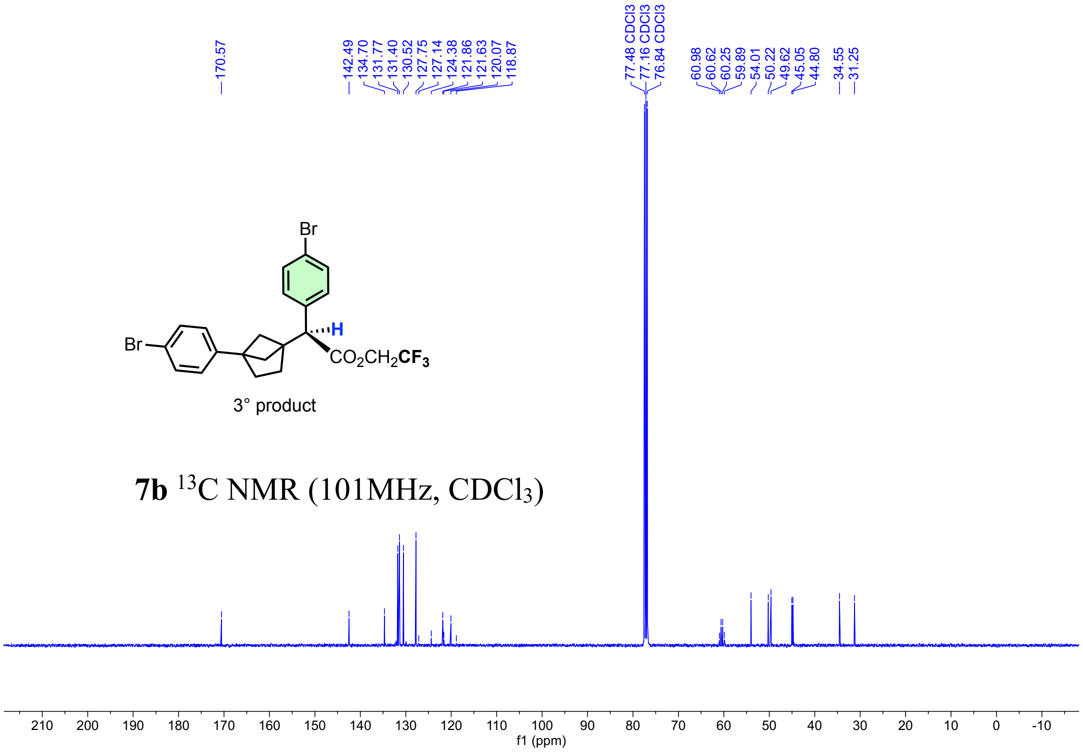

7b ^{13}C NMR (101MHz, $CDCl_3$)

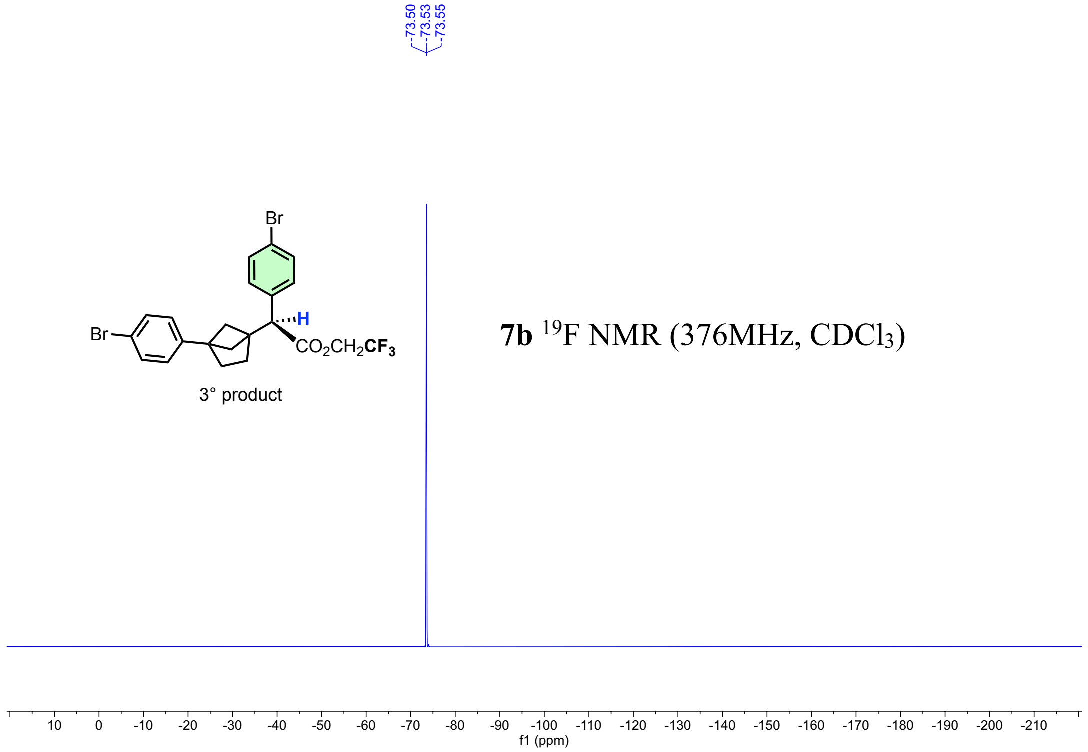

7b ^{19}F NMR (376MHz, $CDCl_3$)

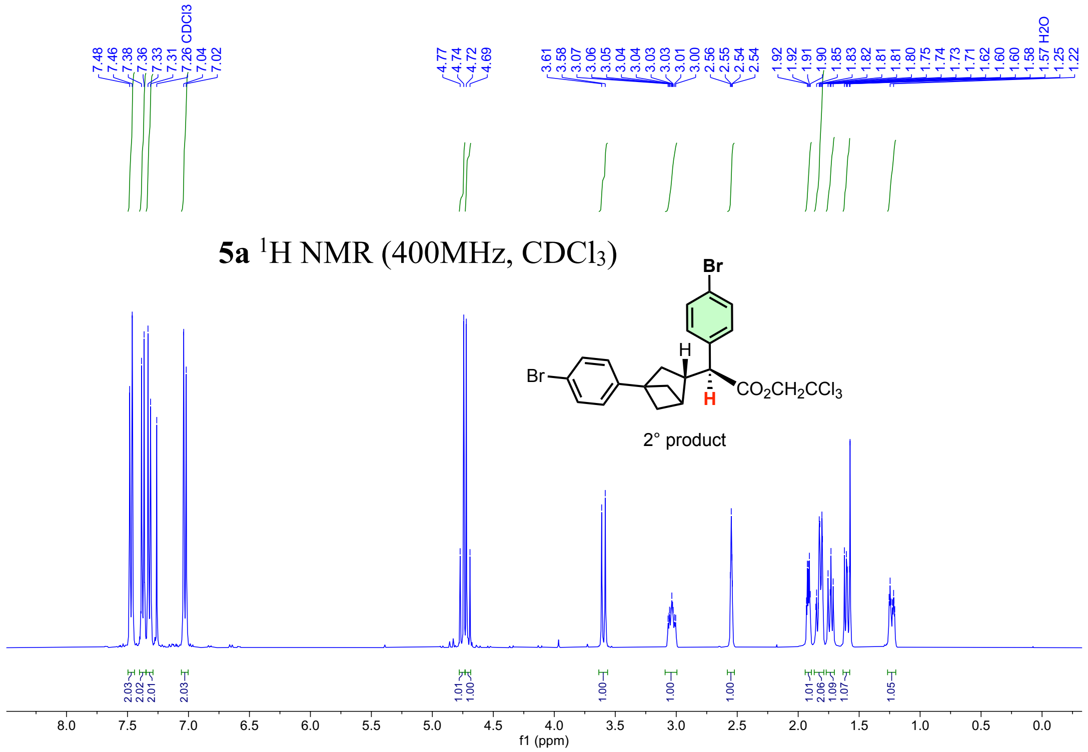

5a ^{1}H NMR (400MHz, $CDCl_3$)

5a ^{13}C NMR (151MHz, $CDCl_3$)

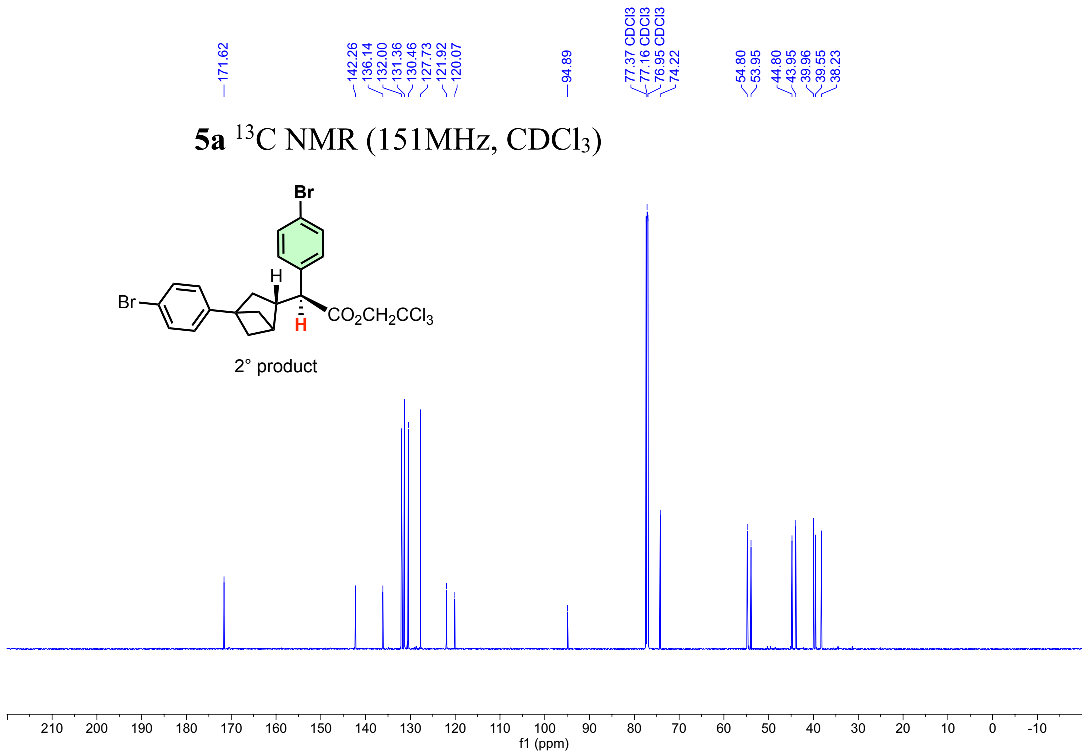

7a ^{1}H NMR (400MHz, $CDCl_3$)

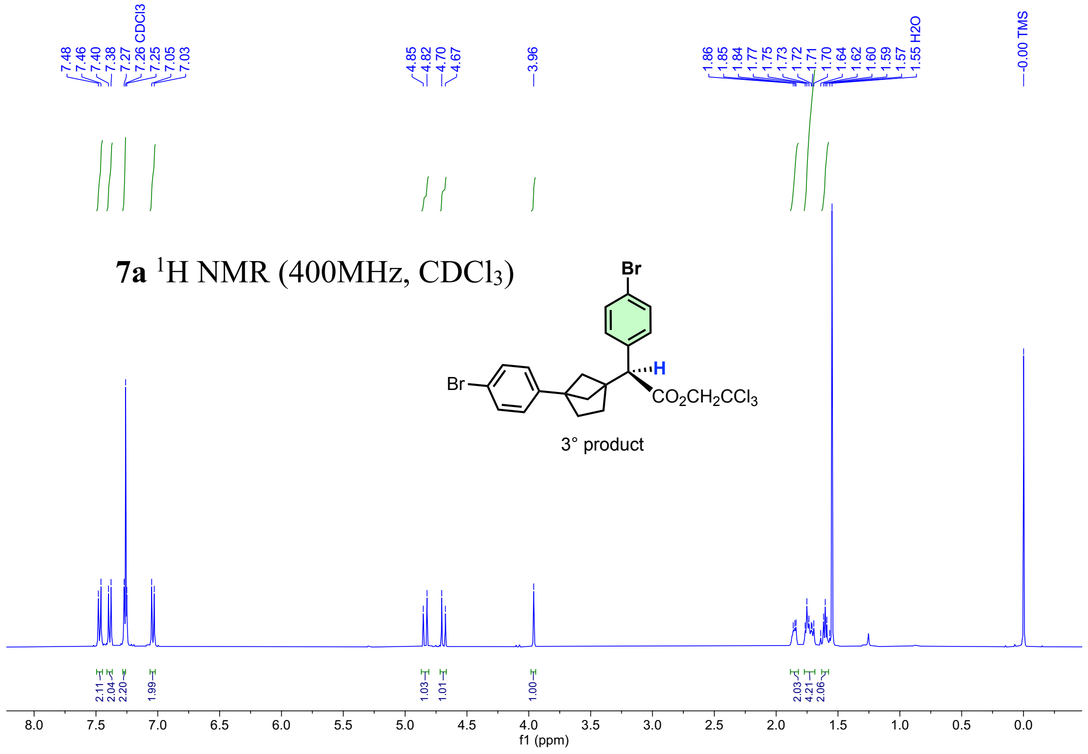

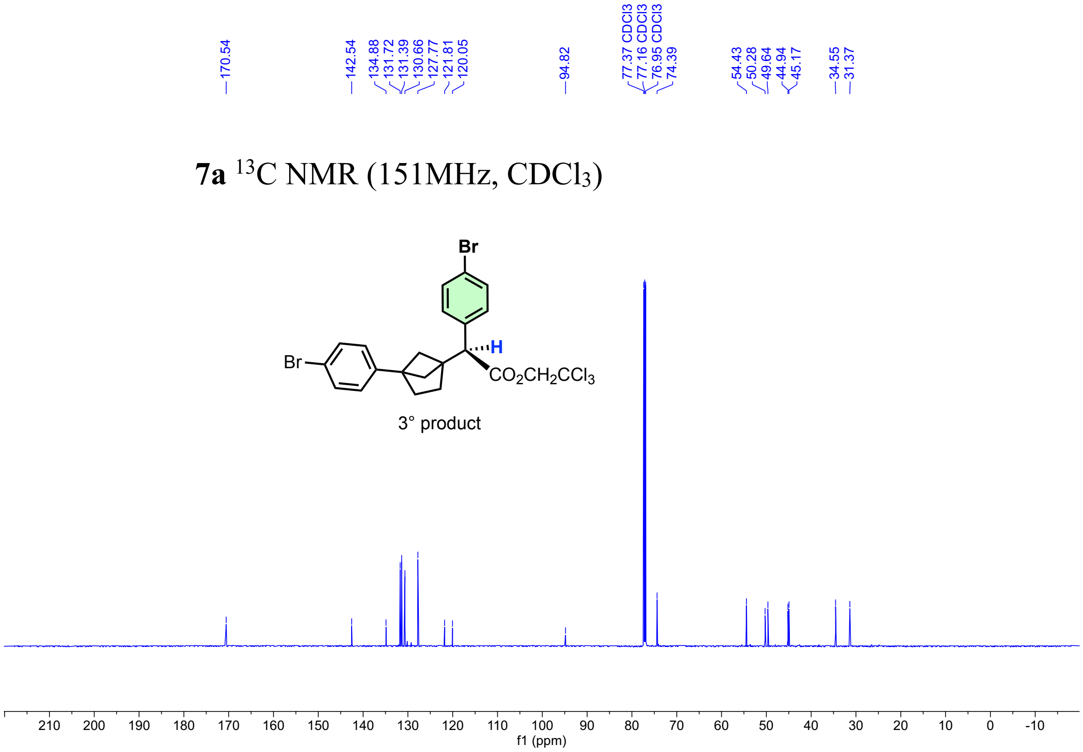

7a ^{13}C NMR (151MHz, $CDCl_3$)

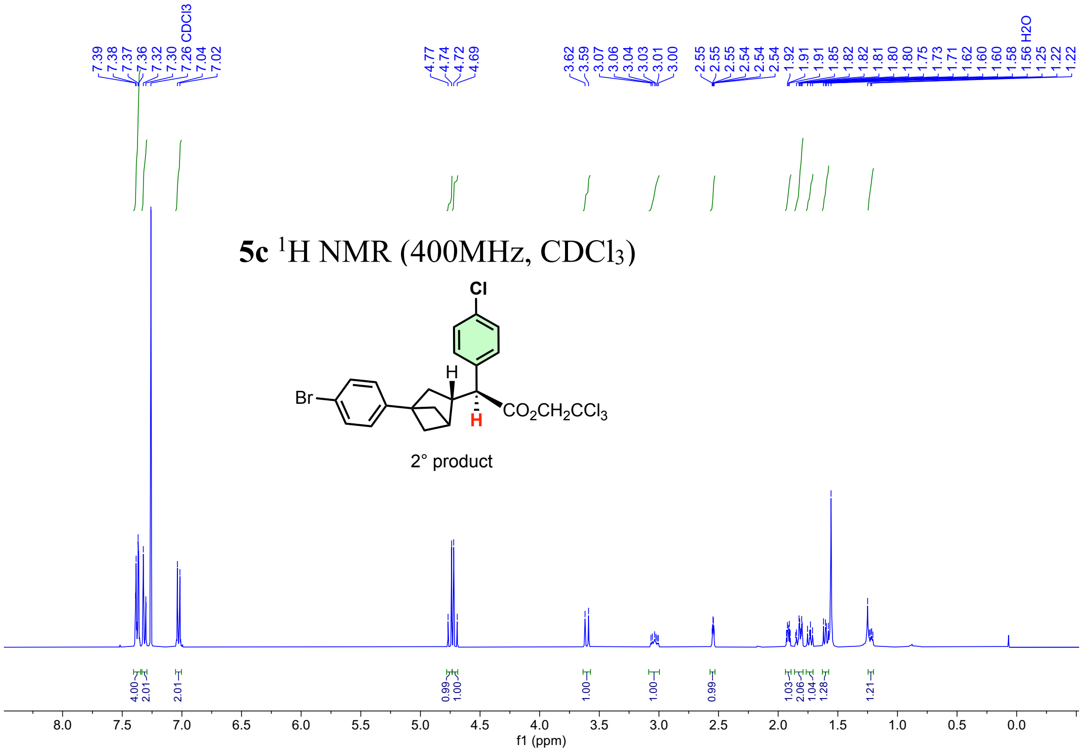

5c ^{1}H NMR (400MHz, $CDCl_3$)

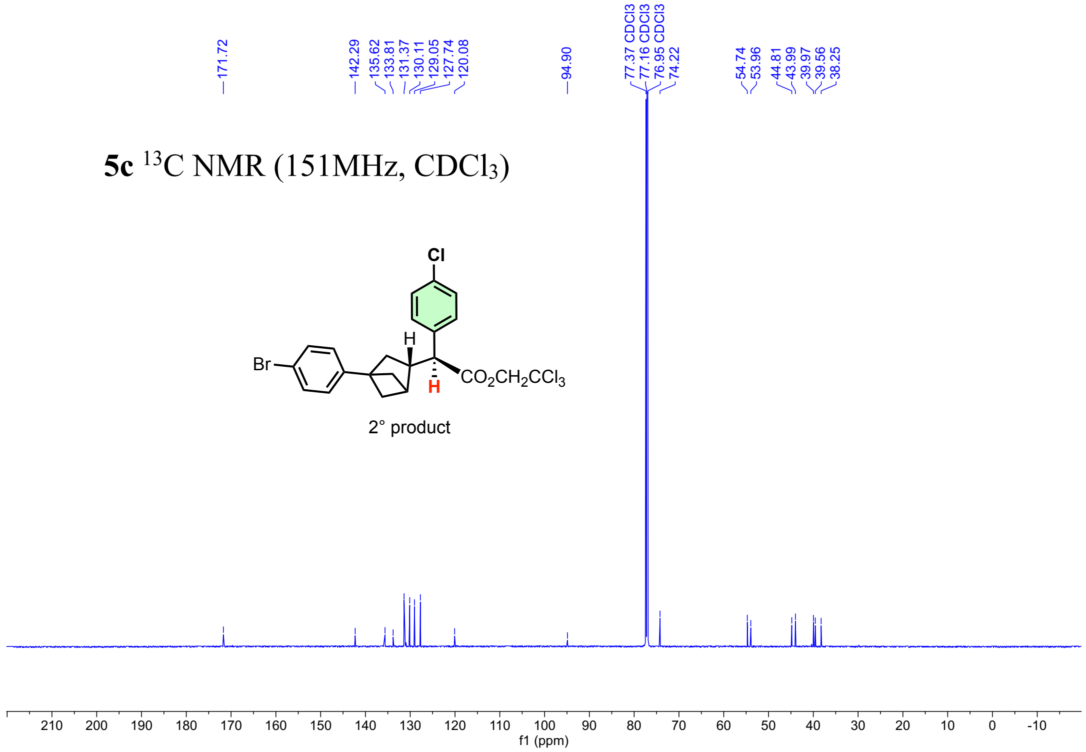
171.72
142.29
135.62
133.81
131.37
130.11
129.05
127.74
120.08
94.90
77.37 CDCl3
77.16 CDCl3
76.95 CDCl3
74.22
54.74
53.96
44.81
43.99
39.97
39.56
38.25
5c 13C NMR (151MHz, CDCl3)
Cl
H
Br
H
CO2CH2CCl3
2° product
f1 (ppm)

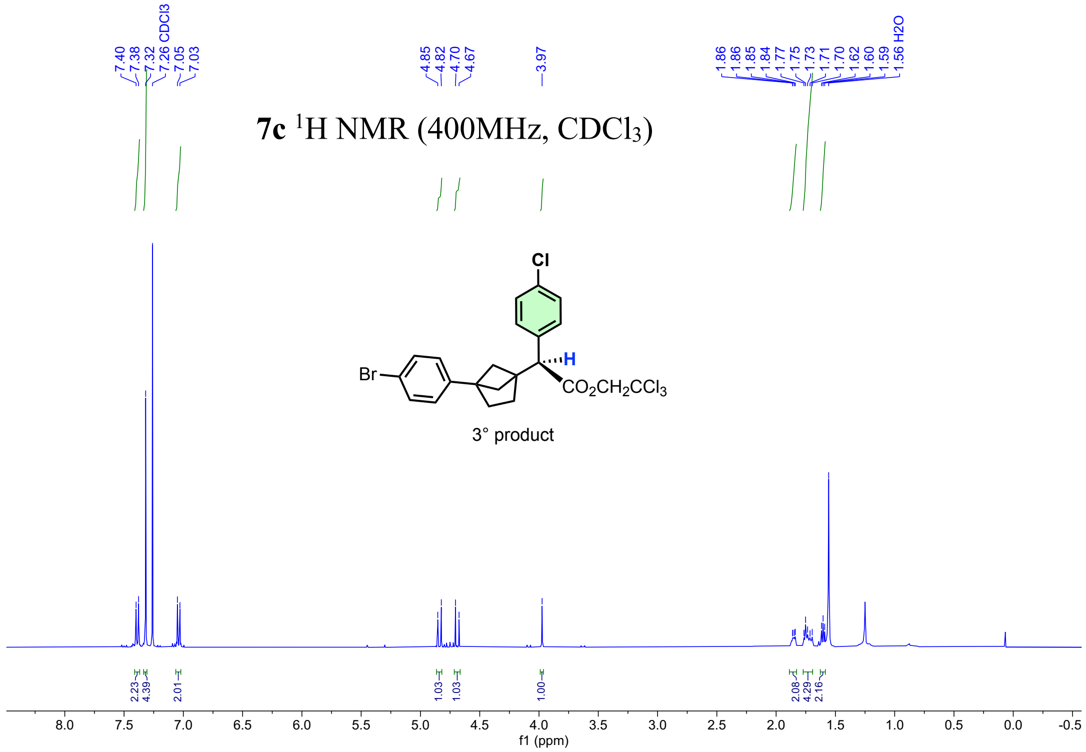
7.40
7.38
7.32
7.26 CDCl3
7.05
7.03
4.85
4.82
4.70
4.67
3.97
1.86
1.86
1.85
1.84
1.77
1.75
1.73
1.71
1.70
1.62
1.60
1.59
1.56 H2O
7c 1H NMR (400MHz, CDCl3)
Cl
H
Br
CO2CH2CCl3
3° product
2.23
4.39
2.01
1.03
1.03
1.00
2.08
4.29
2.16
f1 (ppm)

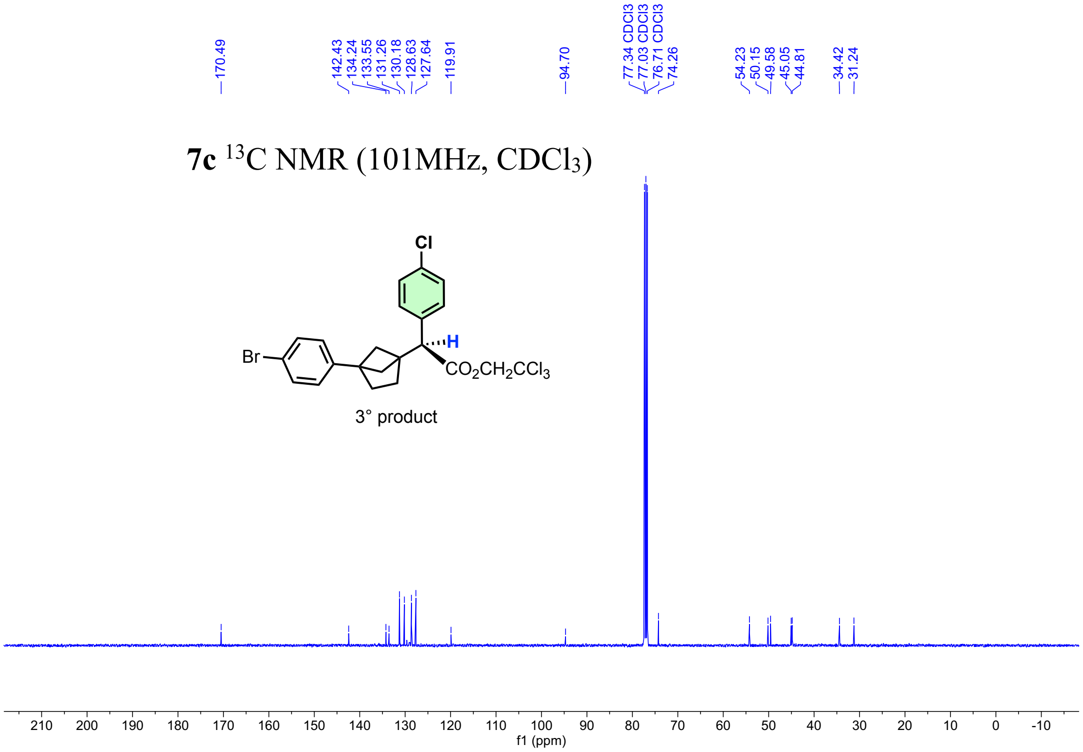
170.49
142.43
134.24
133.55
131.26
130.18
128.63
127.64
119.91
94.70
77.34 CDCl3
77.03 CDCl3
76.71 CDCl3
74.26
54.23
50.15
49.58
45.05
44.81
34.42
31.24
7c 13C NMR (101MHz, CDCl3)
Cl
H
Br
CO2CH2CCl3
3° product
f1 (ppm)

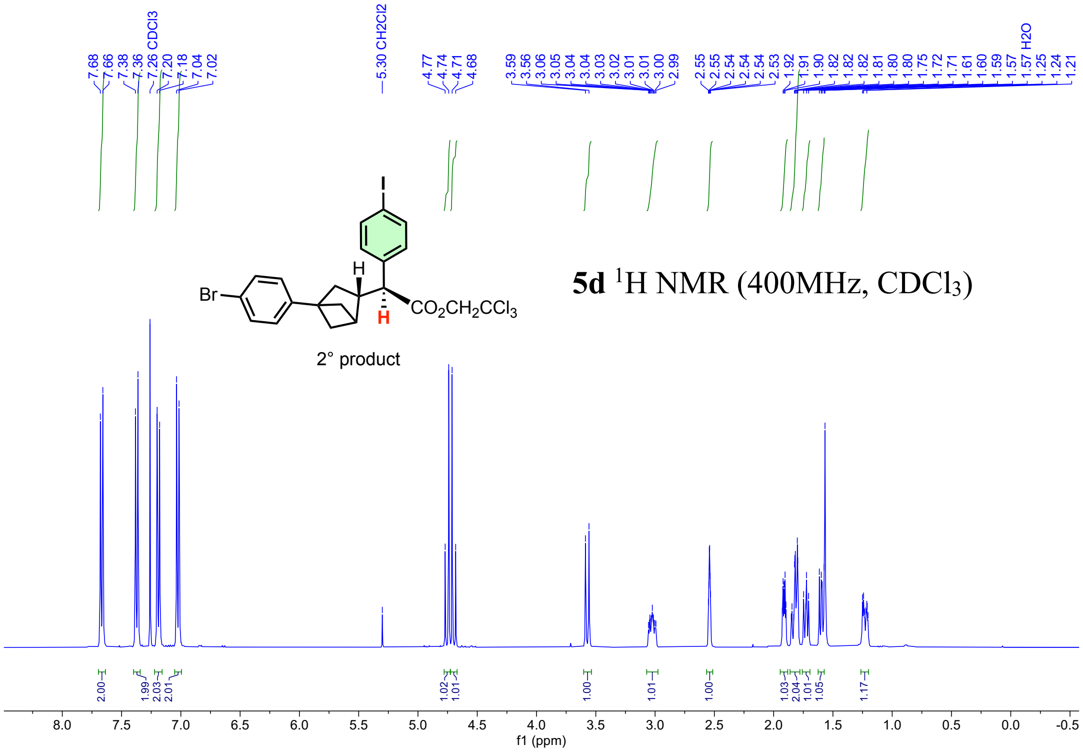
7.68
7.66
7.38
7.36
7.26 CDCl3
7.20
7.18
7.04
7.02
5.30 CH2Cl2
4.77
4.74
4.71
4.68
3.59
3.56
3.06
3.05
3.04
3.03
3.02
3.01
3.01
3.00
2.99
2.55
2.55
2.54
2.54
2.54
2.53
1.92
1.91
1.90
1.82
1.82
1.82
1.81
1.80
1.75
1.72
1.71
1.61
1.60
1.59
1.57
1.57 H2O
1.25
1.24
1.21
I
H
Br
H
CO2CH2CCl3
2° product
5d 1H NMR (400MHz, CDCl3)
2.00
1.99
2.03
2.01
1.02
1.01
1.00
1.01
1.00
1.03
2.04
1.01
1.05
1.17
f1 (ppm)

5d ^{13}C NMR (151MHz, $CDCl_3$)

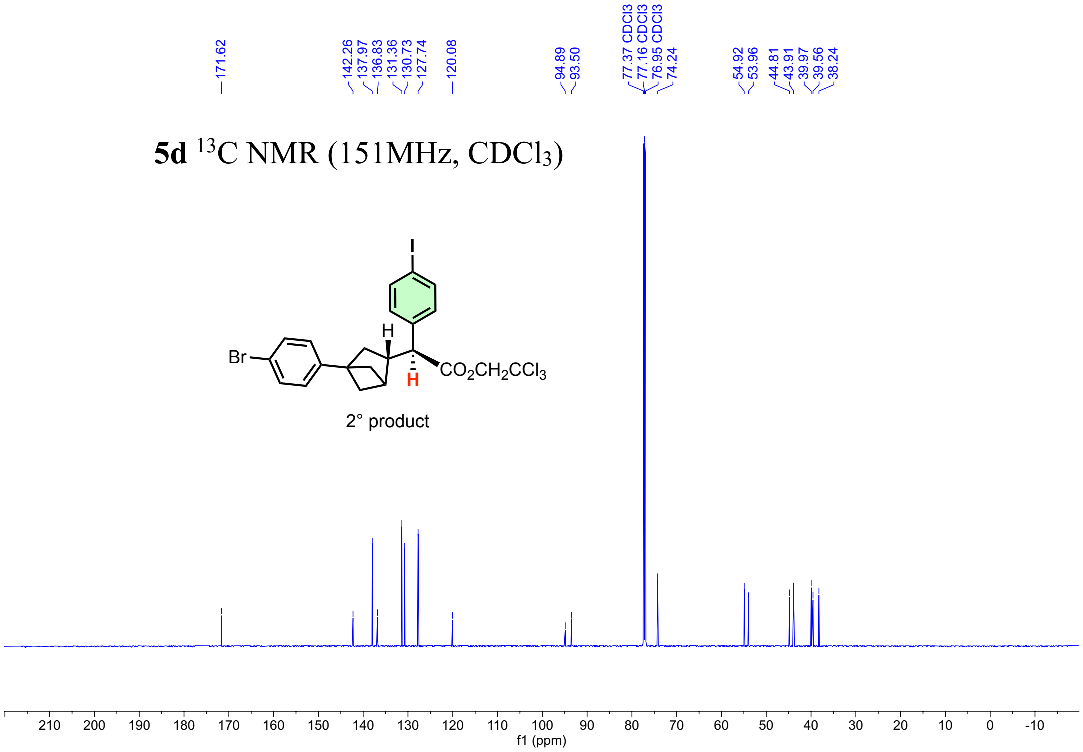

7d ^{1}H NMR (400MHz, $CDCl_3$)

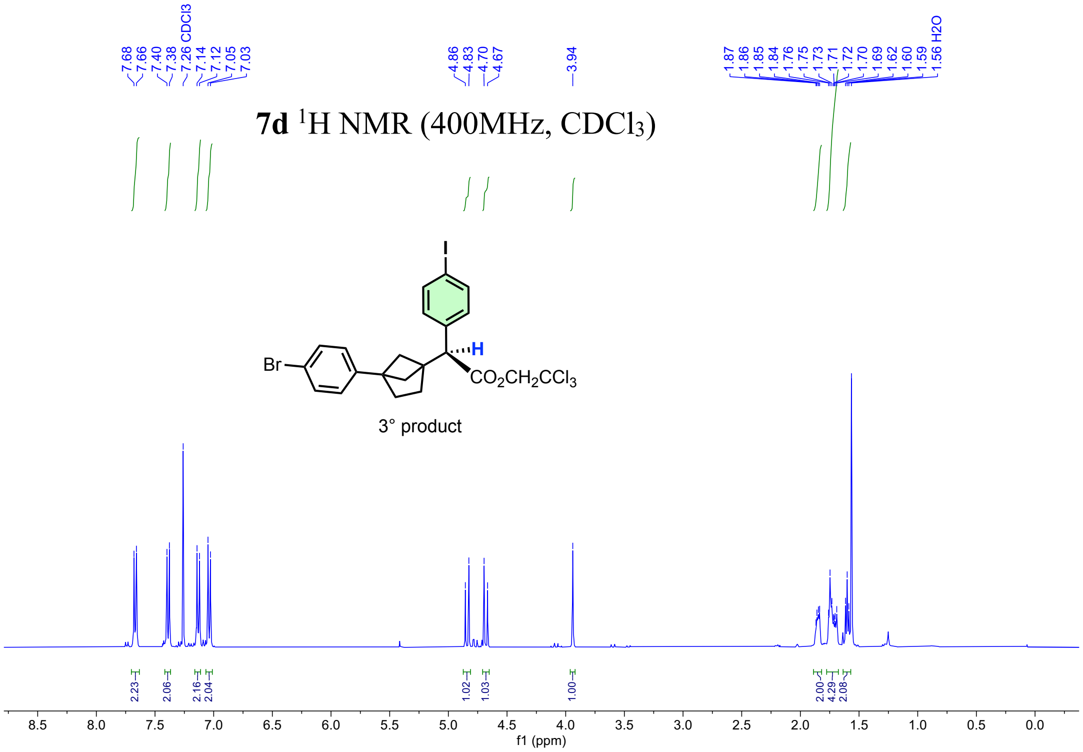

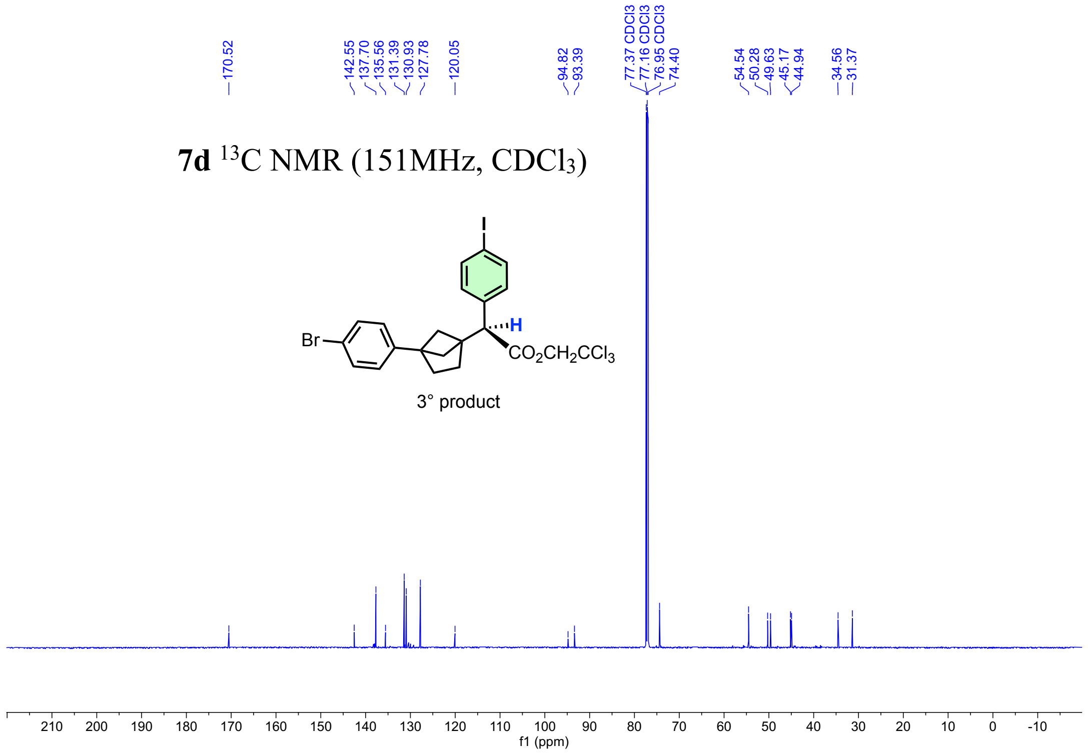
170.52
142.55
137.70
135.56
131.39
130.93
127.78
120.05
94.82
93.39
77.37 CDCl3
77.16 CDCl3
76.95 CDCl3
74.40
54.54
50.28
49.63
45.17
44.94
34.56
31.37
7d 13C NMR (151MHz, CDCl3)
I
H
Br
CO2CH2CCl3
3° product
210 200 190 180 170 160 150 140 130 120 110 100 90 80 70 60 50 40 30 20 10 0 -10
f1 (ppm)

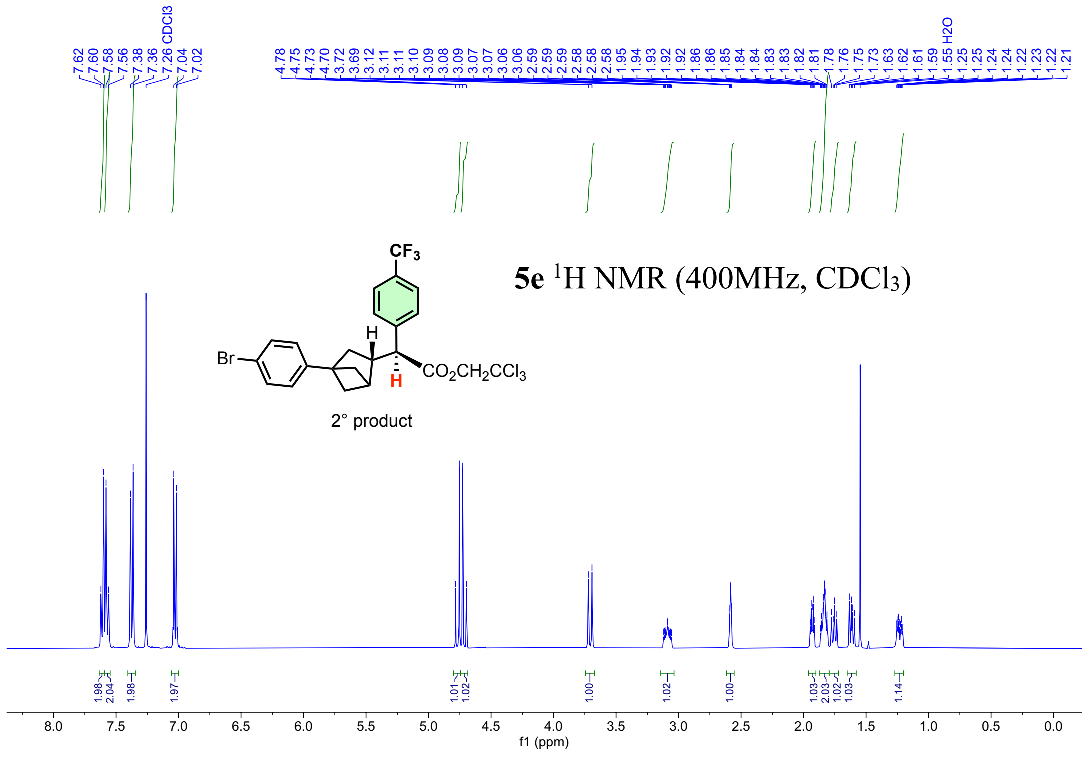
7.62
7.60
7.58
7.56
7.38
7.36
7.26 CDCl3
7.04
7.02
4.78
4.75
4.73
4.70
3.72
3.69
3.12
3.11
3.11
3.10
3.09
3.08
3.09
3.07
3.06
3.06
2.59
2.59
2.58
2.58
1.95
1.94
1.93
1.92
1.92
1.86
1.85
1.84
1.84
1.83
1.83
1.82
1.81
1.78
1.76
1.75
1.73
1.63
1.62
1.61
1.59
1.55 H2O
1.25
1.25
1.24
1.24
1.22
1.23
1.22
1.21
5e 1H NMR (400MHz, CDCl3)
CF3
H
Br
H
CO2CH2CCl3
2° product
1.98
2.04
1.98
1.97
1.01
1.02
1.00
1.02
1.00
1.03
2.03
1.02
1.03
1.14
8.0 7.5 7.0 6.5 6.0 5.5 5.0 4.5 4.0 3.5 3.0 2.5 2.0 1.5 1.0 0.5 0.0
f1 (ppm)

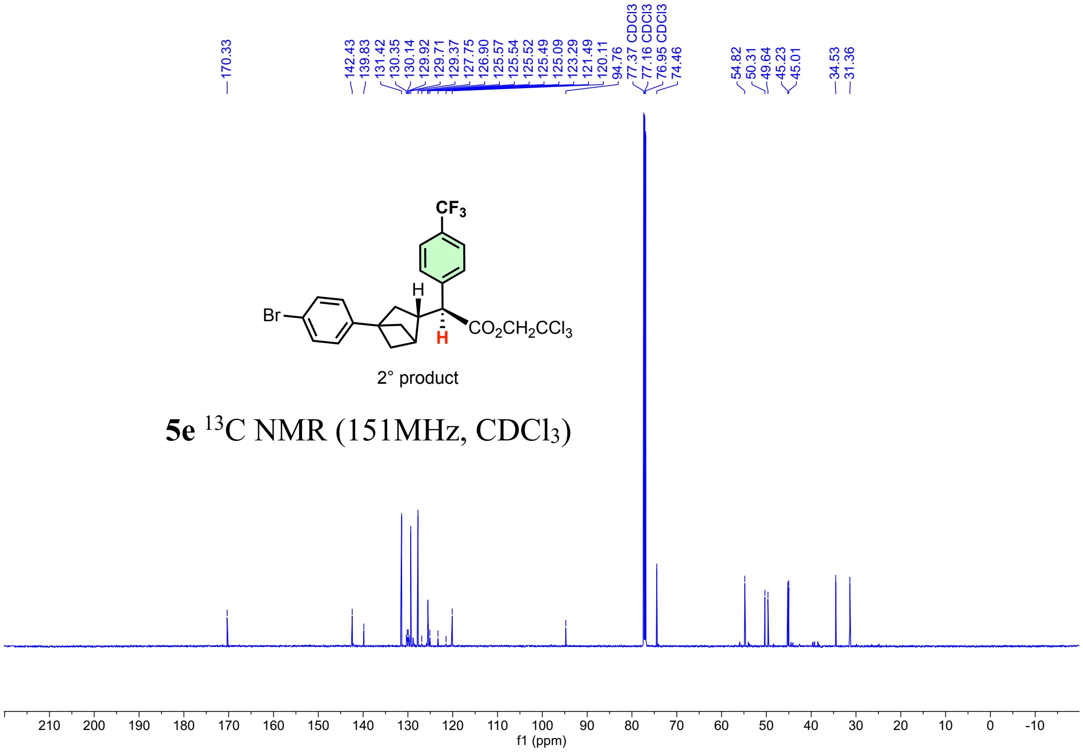

5e ^{13}C NMR (151MHz, $CDCl_3$)

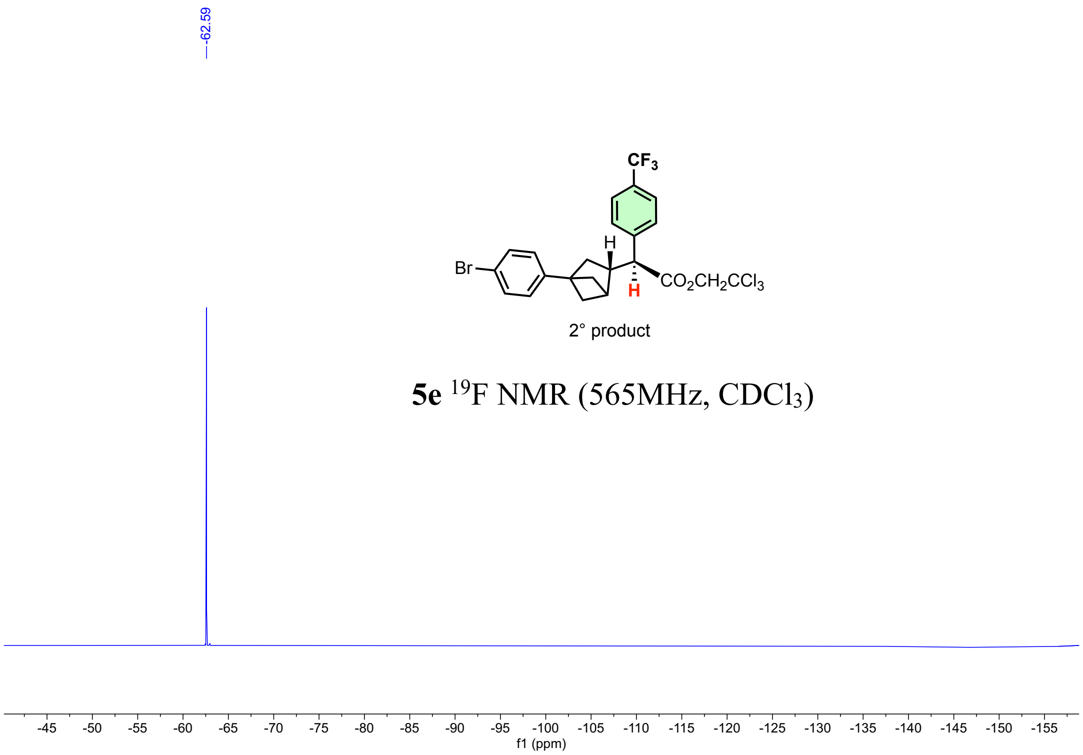

5e ^{19}F NMR (565MHz, $CDCl_3$)

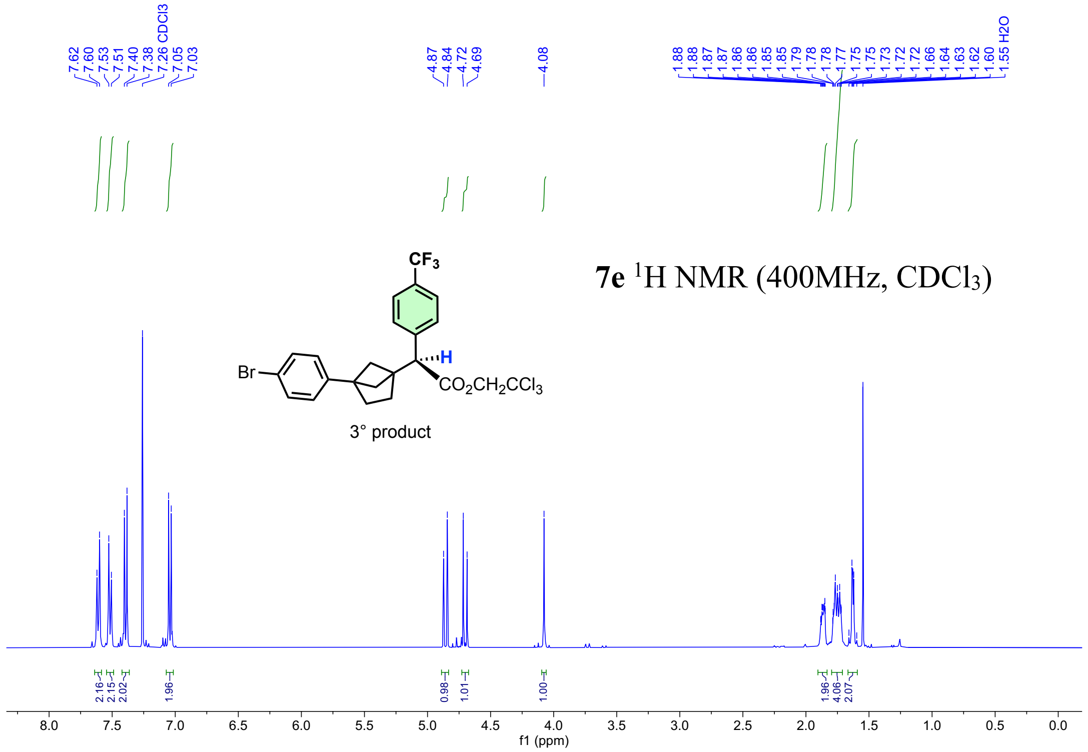
7.62
7.60
7.53
7.51
7.40
7.38
7.26 CDCl3
7.05
7.03
4.87
4.84
4.72
4.69
4.08
1.55 H2O
CF3
H
Br
CO2CH2CCl3
3° product
7e 1H NMR (400MHz, CDCl3)
f1 (ppm)

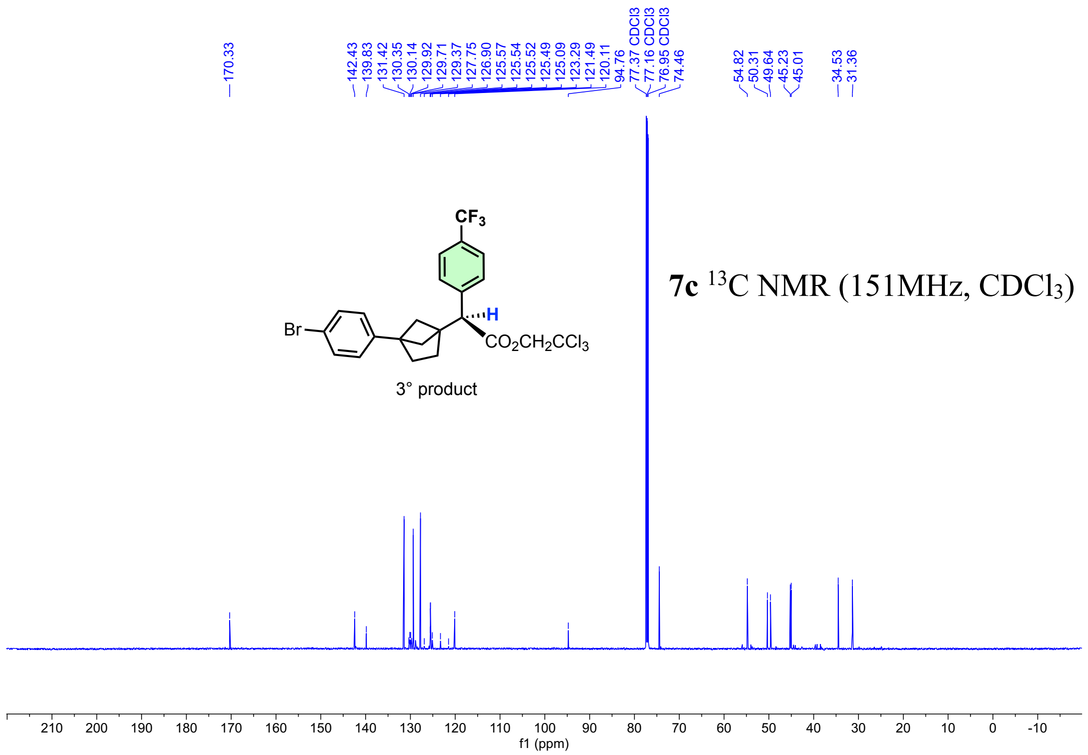
170.33
142.43
139.83
131.42
130.35
130.14
129.92
129.71
129.37
127.75
126.90
125.57
125.54
125.52
125.49
125.09
123.29
121.49
120.11
94.76
77.37 CDCl3
77.16 CDCl3
76.95 CDCl3
74.46
54.82
50.31
49.64
45.23
45.01
34.53
31.36
CF3
H
Br
CO2CH2CCl3
3° product
7c 13C NMR (151MHz, CDCl3)
f1 (ppm)

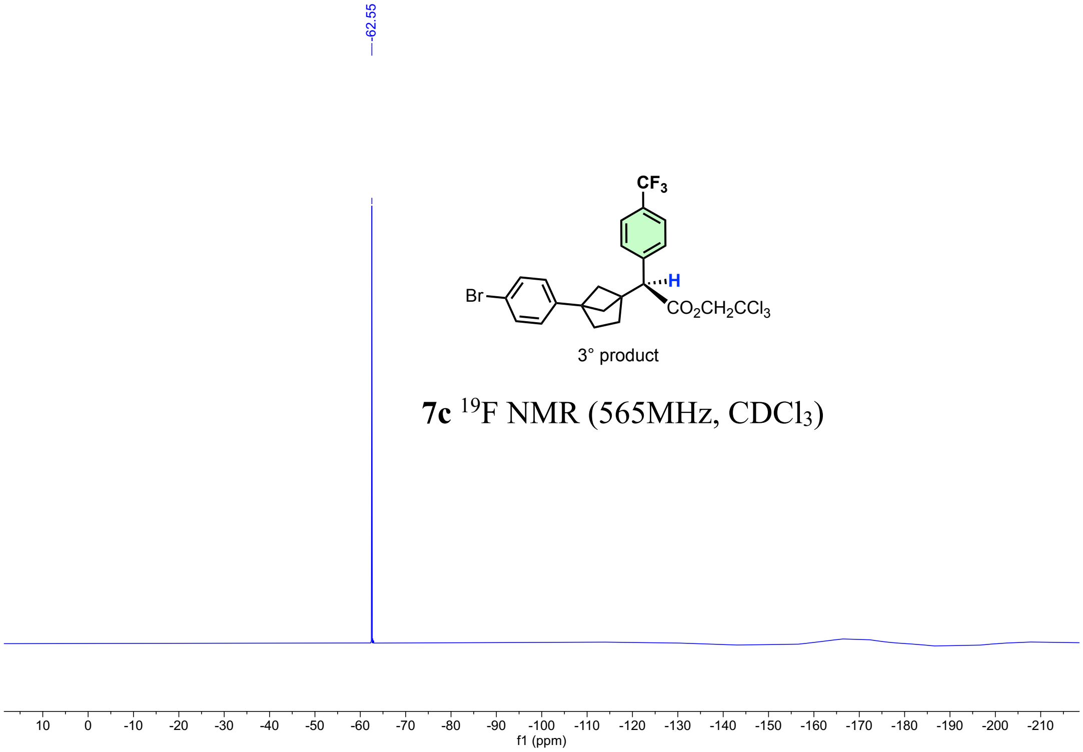

7c ^{19}F NMR (565MHz, $CDCl_3$)

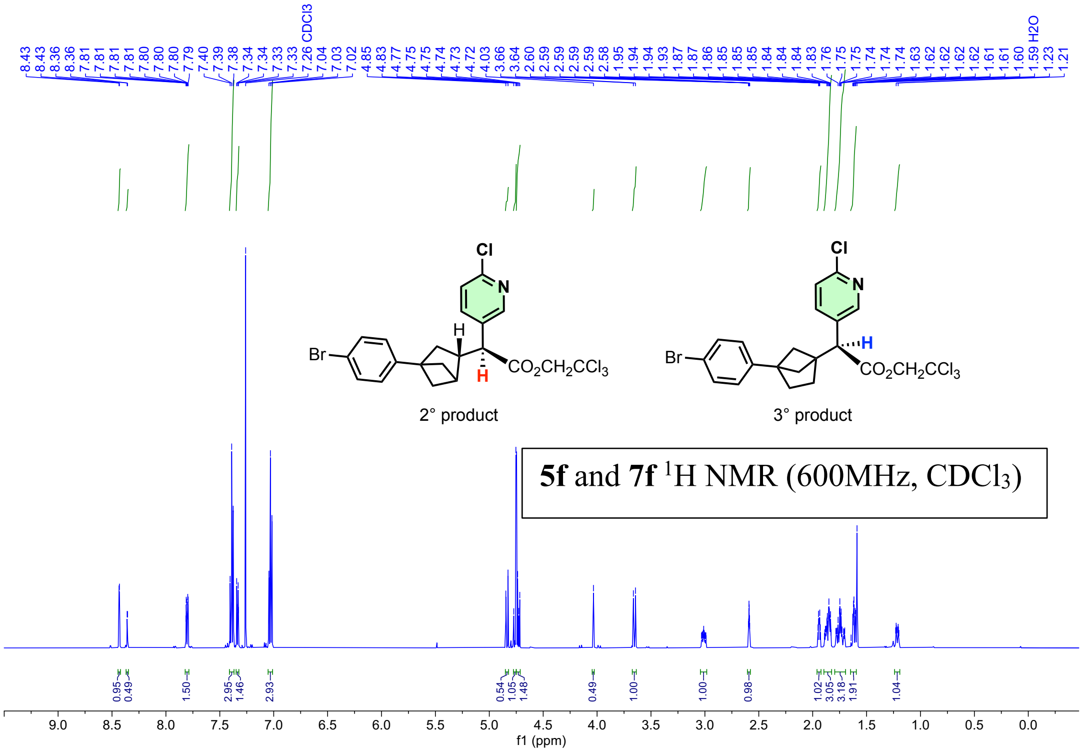

5f and **7f** ^{1}H NMR (600MHz, $CDCl_3$)

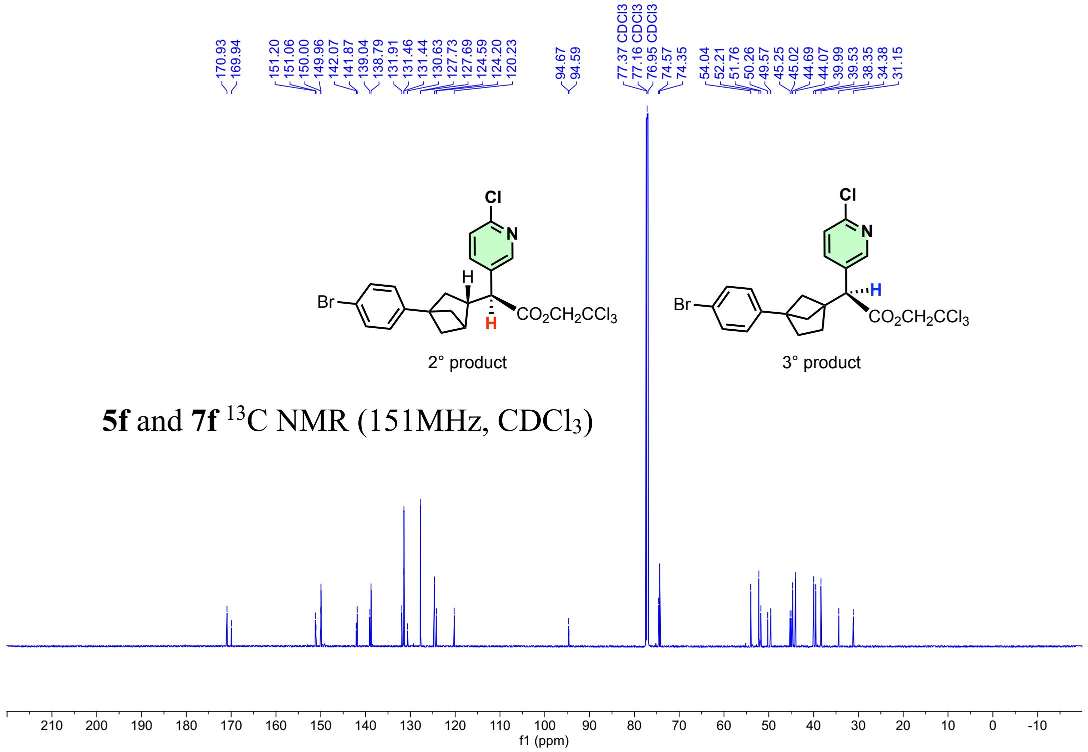
170.93
169.94
151.20
151.06
150.00
149.96
142.07
141.87
139.04
138.79
131.91
131.46
131.44
130.63
127.73
127.69
124.59
124.20
120.23
94.67
94.59
77.37 CDCl3
77.16 CDCl3
76.95 CDCl3
74.57
74.35
54.04
52.21
51.76
50.26
49.57
45.25
45.02
44.69
44.07
39.99
39.53
38.35
34.38
31.15
2° product
3° product
5f and **7f** ^{13}C NMR (151MHz, $CDCl_3$)
f1 (ppm)

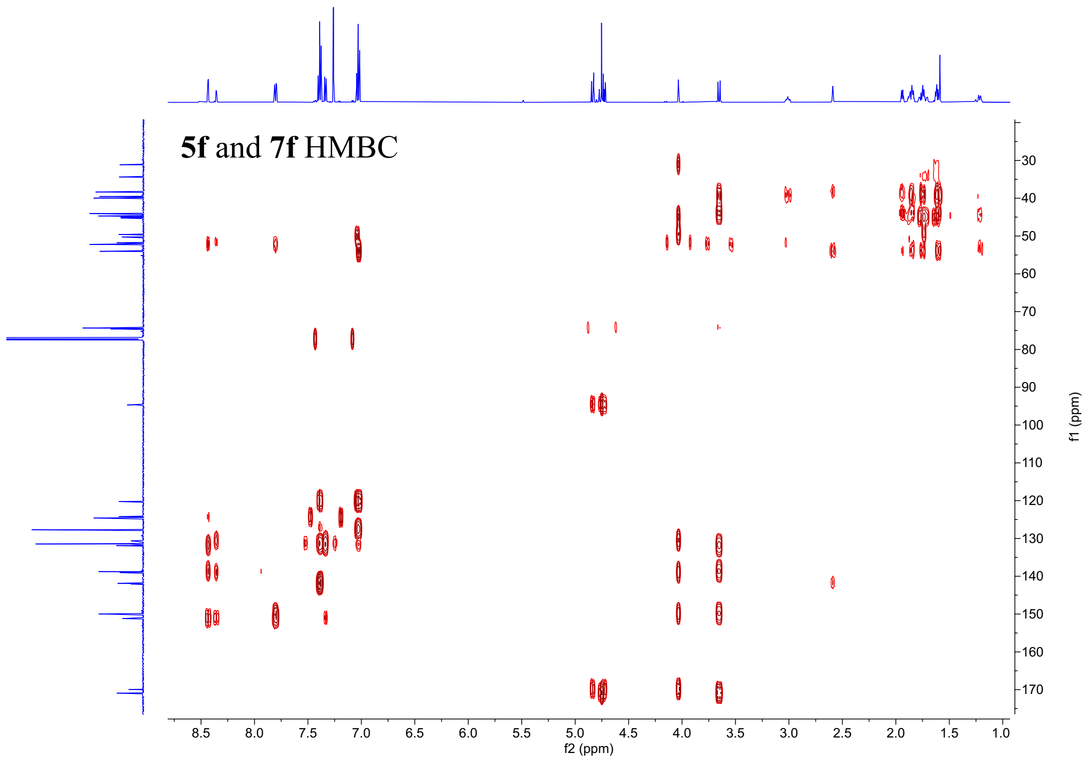
5f and **7f** HMBC
f2 (ppm)
f1 (ppm)

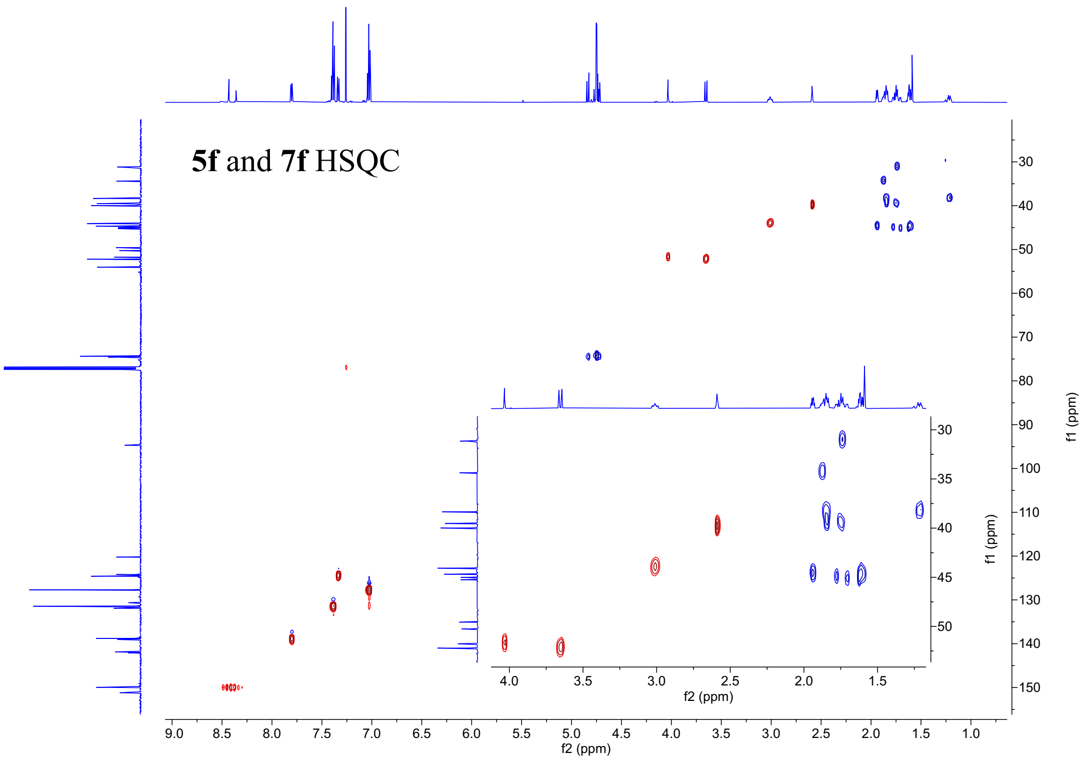

5f and **7f** HSQC
f2 (ppm)
f1 (ppm)

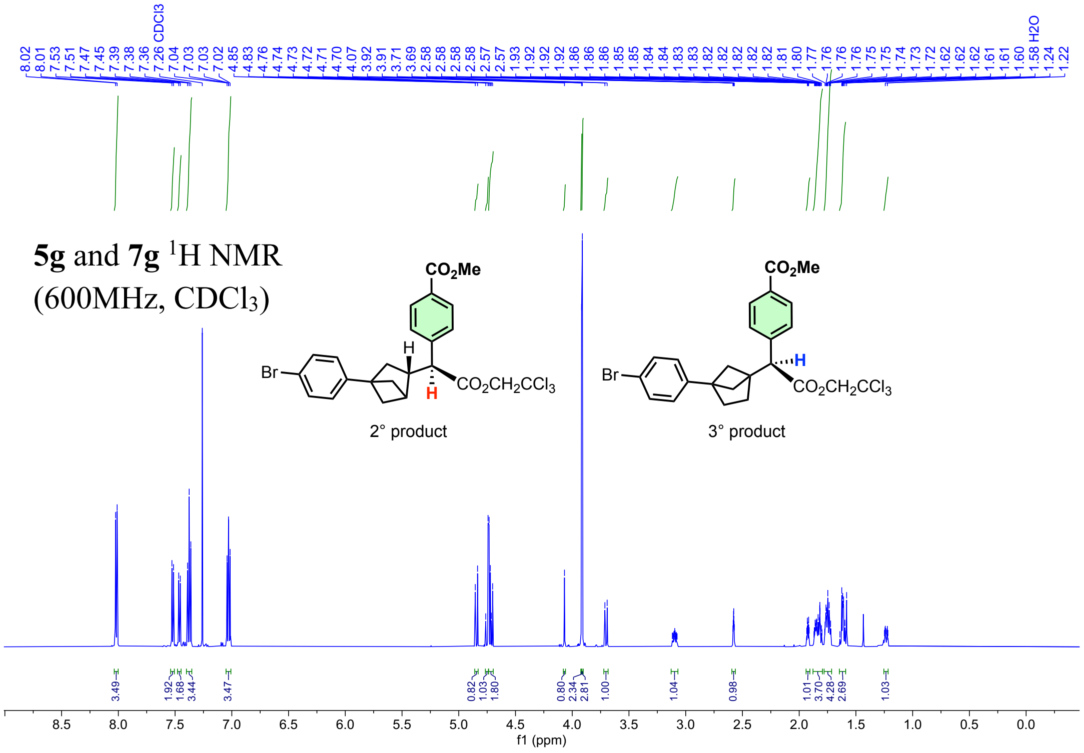

8.02 8.01 7.53 7.51 7.47 7.45 7.39 7.38 7.36 7.26 CDCl3 7.04 7.03 7.03 7.02 4.85 4.83 4.76 4.74 4.73 4.72 4.71 4.70 4.07 3.92 3.91 3.71 3.69 2.58 2.58 2.58 2.58 2.57 2.57 1.93 1.92 1.92 1.92 1.86 1.86 1.86 1.85 1.85 1.84 1.84 1.83 1.83 1.82 1.82 1.82 1.82 1.82 1.81 1.80 1.77 1.76 1.76 1.76 1.75 1.75 1.74 1.73 1.72 1.62 1.62 1.62 1.61 1.61 1.60 1.58 H2O 1.24 1.22
5g and **7g** ^{1}H NMR
(600MHz, $CDCl_3$)
CO_2Me
Br
H
H
$CO_2CH_2CCl_3$
2° product
CO_2Me
Br
H
$CO_2CH_2CCl_3$
3° product
3.49 1.92 1.68 3.44 3.47 0.82 1.03 1.80 0.80 2.34 2.81 1.00 1.04 0.98 1.01 3.70 4.28 2.69 1.03
f1 (ppm)

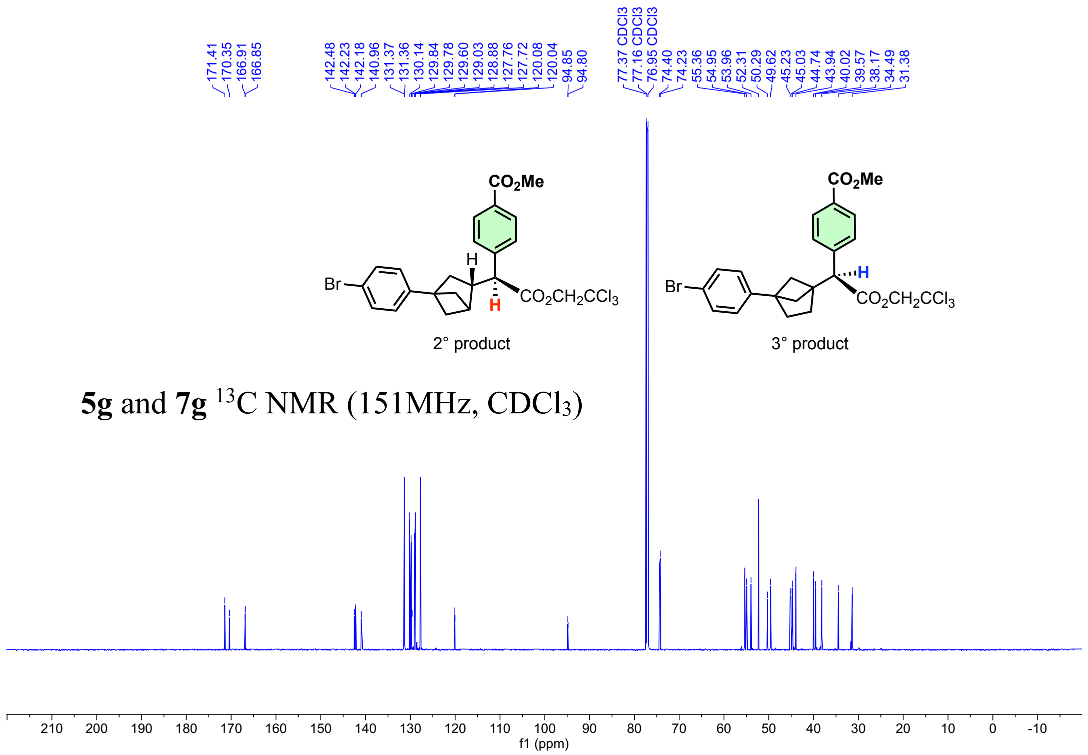
171.41
170.35
166.91
166.85
142.48
142.23
142.18
140.96
131.37
131.36
130.14
129.84
129.78
129.60
129.03
128.88
127.76
127.72
120.08
120.04
94.85
94.80
77.37 CDCl3
77.16 CDCl3
76.95 CDCl3
74.40
74.23
55.36
54.95
53.96
52.31
50.29
49.62
45.23
45.03
44.74
43.94
40.02
39.57
38.17
34.49
31.38
CO2Me
Br
H
H
CO2CH2CCl3
2° product
3° product
5g and 7g 13C NMR (151MHz, CDCl3)
f1 (ppm)

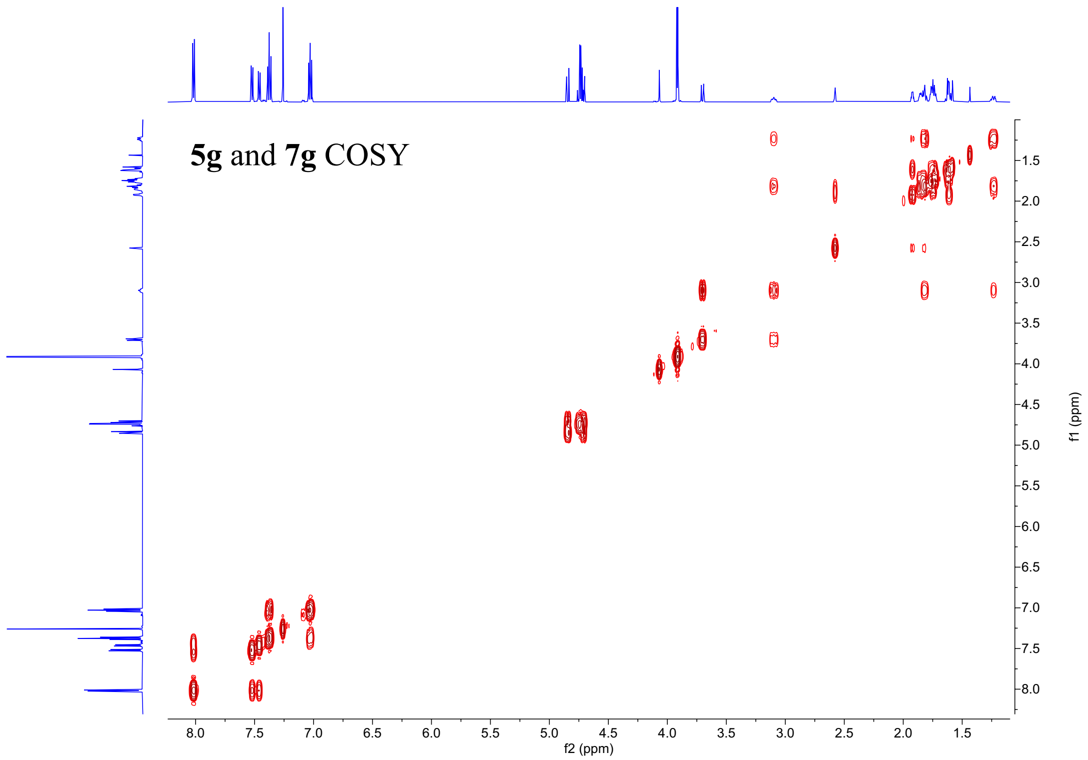
5g and 7g COSY
f2 (ppm)
f1 (ppm)

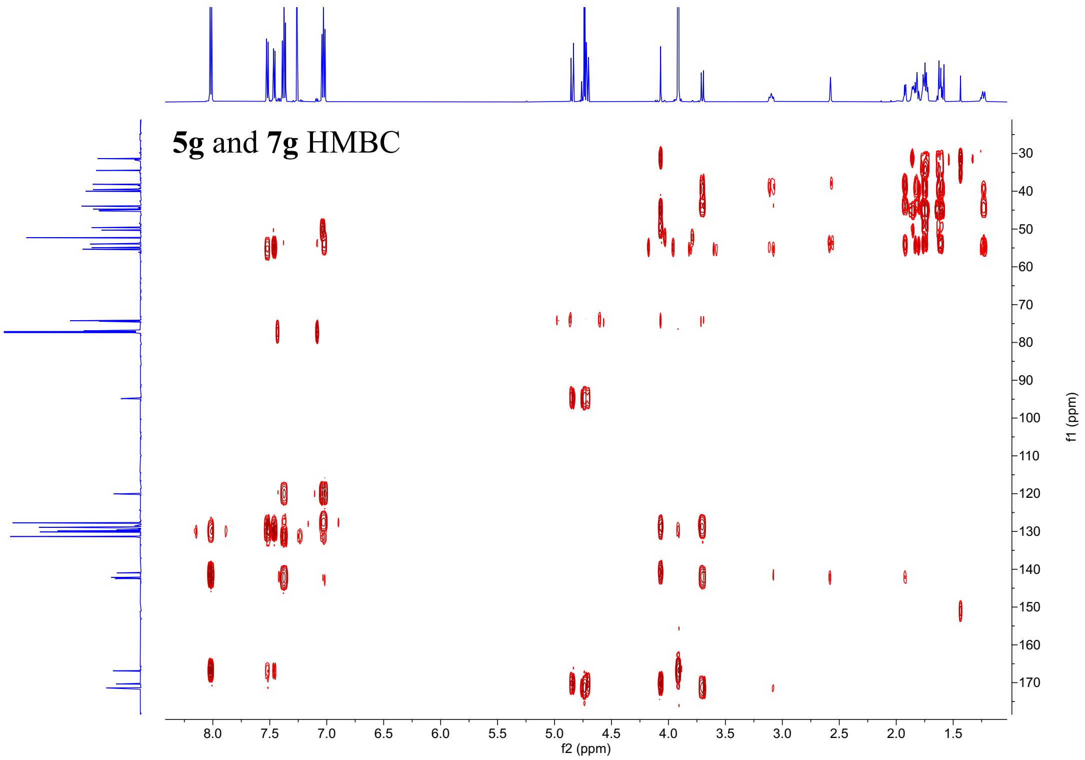
5g and 7g HMBC
f2 (ppm)
f1 (ppm)

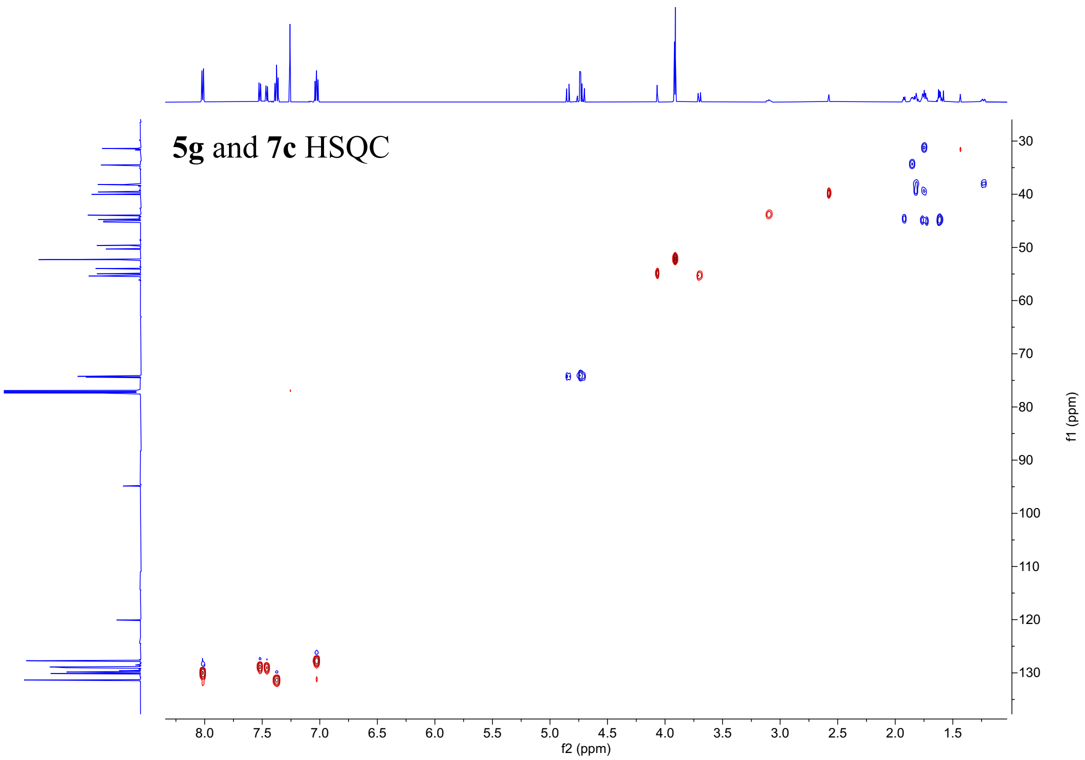
5g and 7c HSQC
f2 (ppm)
f1 (ppm)

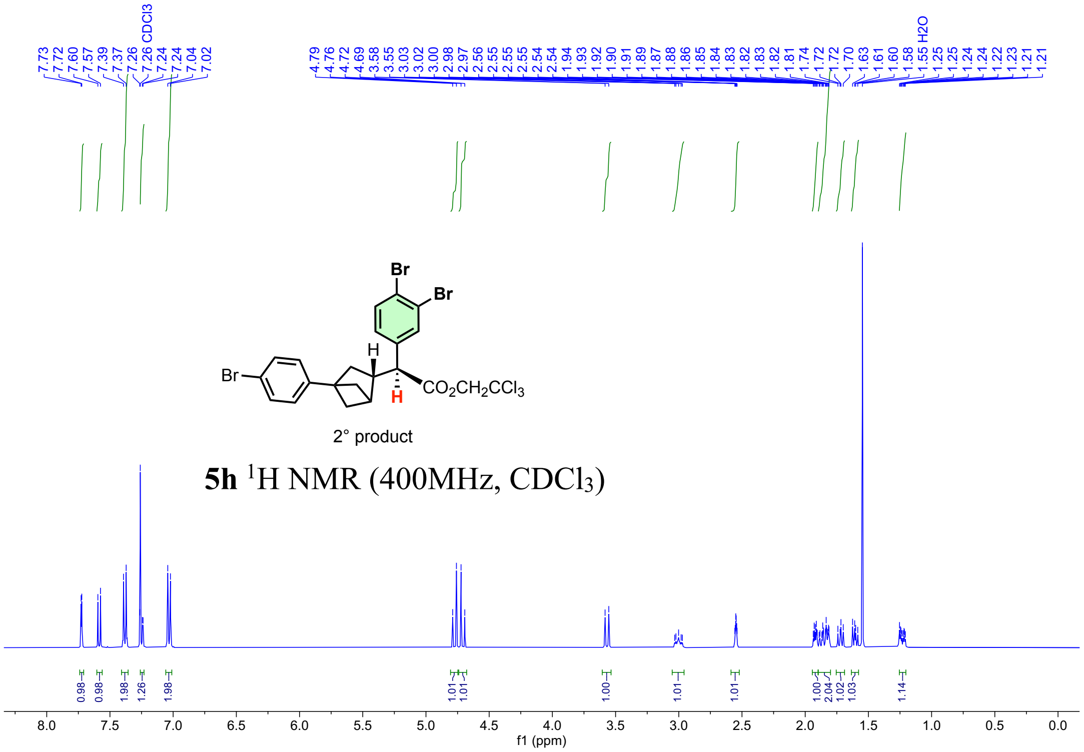

5h ^{1}H NMR (400MHz, $CDCl_3$)

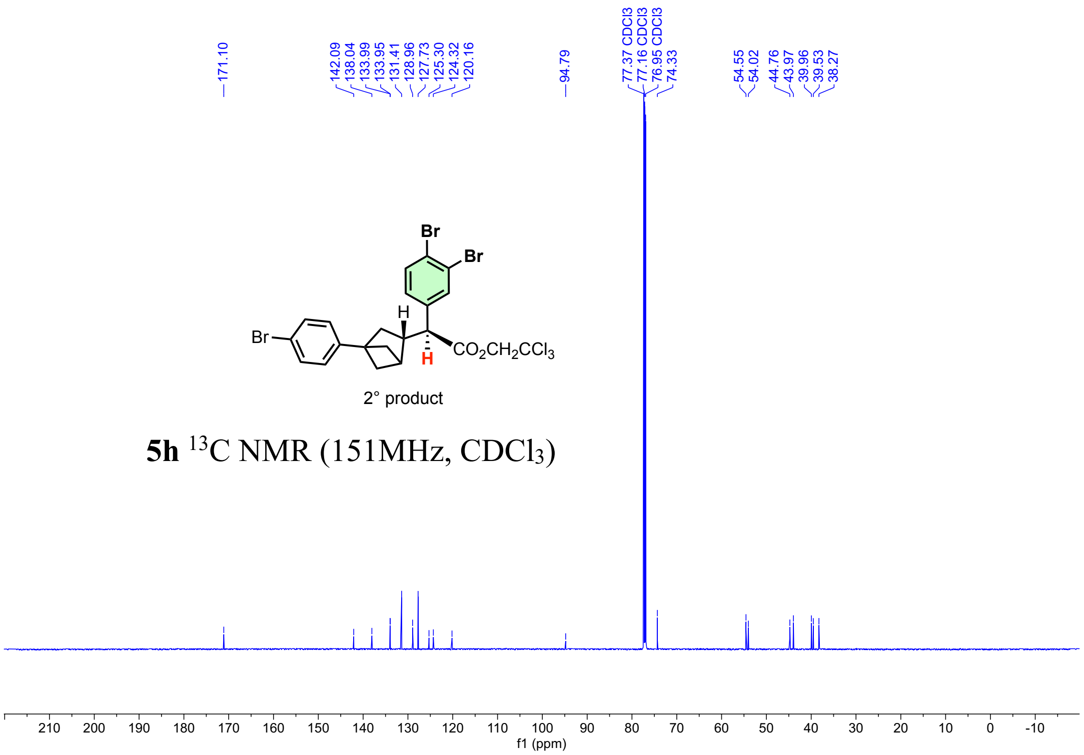

5h ^{13}C NMR (151MHz, $CDCl_3$)

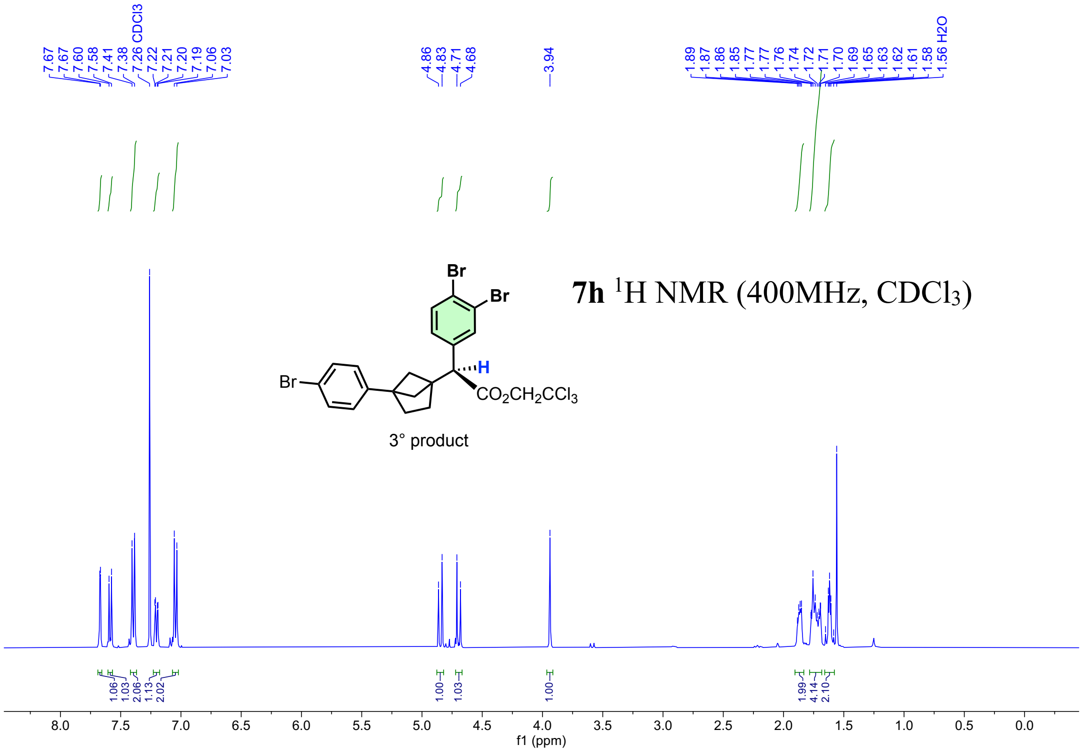

7h ^{1}H NMR (400MHz, $CDCl_3$)

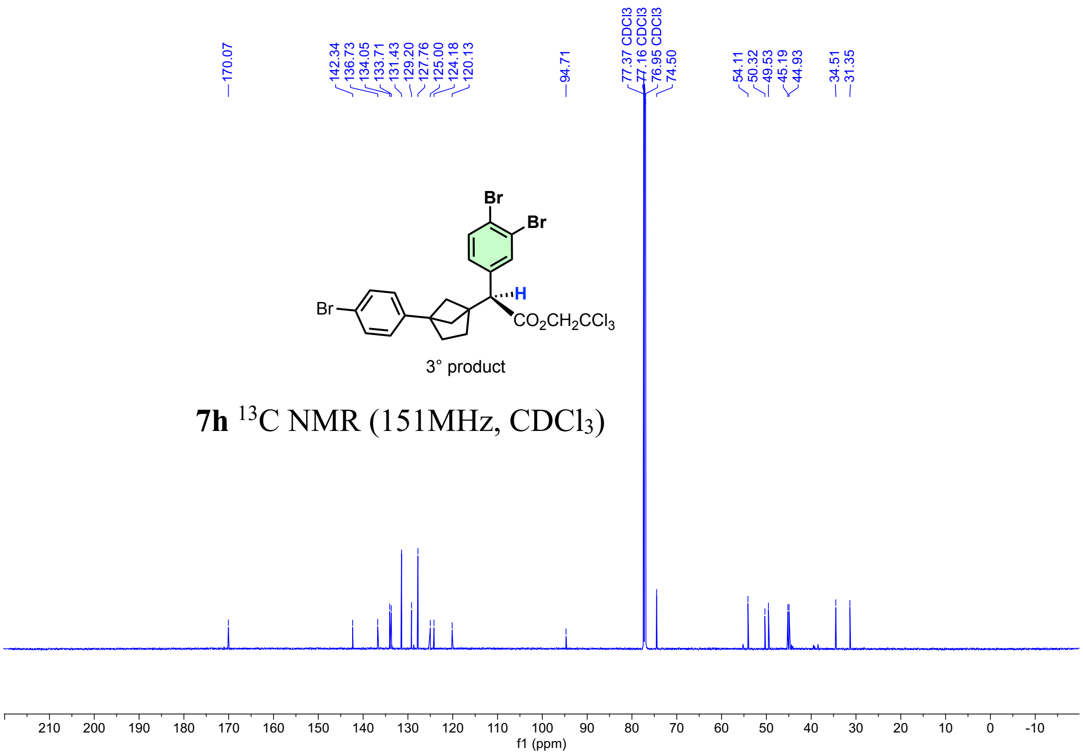

7h ^{13}C NMR (151MHz, $CDCl_3$)

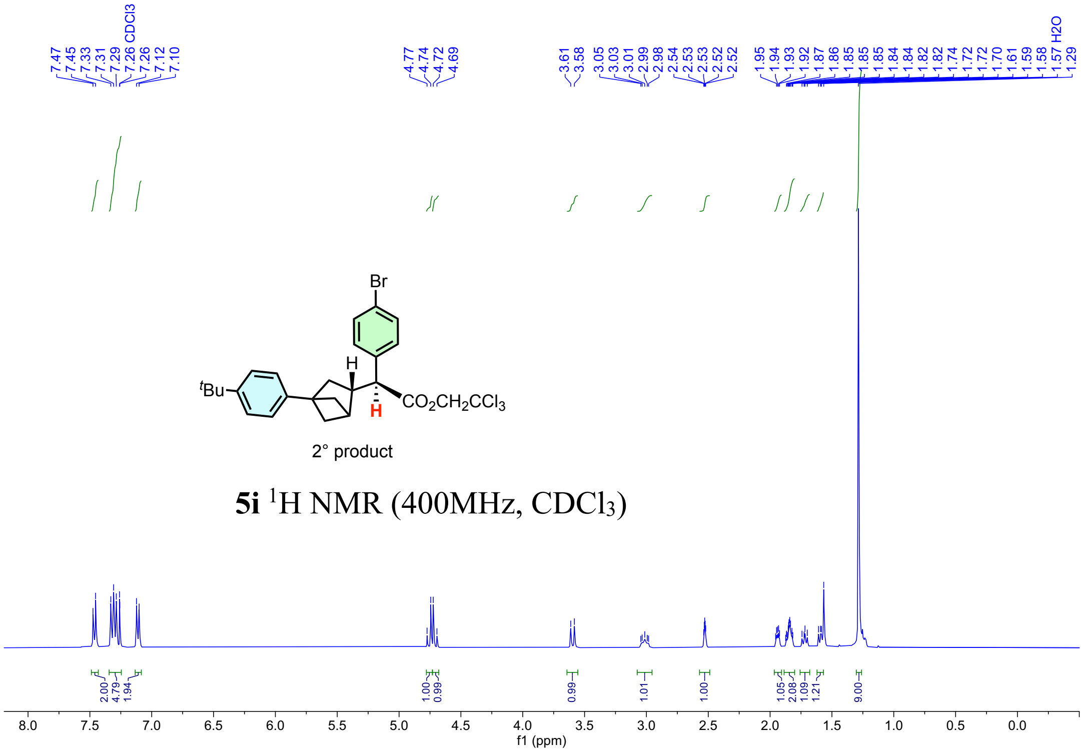
7.47
7.45
7.33
7.31
7.29
7.26 CDCl3
7.26
7.12
7.10
4.77
4.74
4.72
4.69
3.61
3.58
3.05
3.03
3.01
2.99
2.98
2.54
2.53
2.53
2.52
2.52
1.95
1.94
1.93
1.92
1.87
1.86
1.85
1.85
1.85
1.84
1.84
1.82
1.82
1.74
1.72
1.72
1.70
1.61
1.59
1.58
1.57 H2O
1.29
Br
tBu
H
H
CO2CH2CCl3
2° product
5i 1H NMR (400MHz, CDCl3)
2.00
4.79
1.94
1.00
0.99
0.99
1.01
1.00
1.05
2.08
1.09
1.21
9.00
8.0
7.5
7.0
6.5
6.0
5.5
5.0
4.5
4.0
3.5
3.0
2.5
2.0
1.5
1.0
0.5
0.0
f1 (ppm)

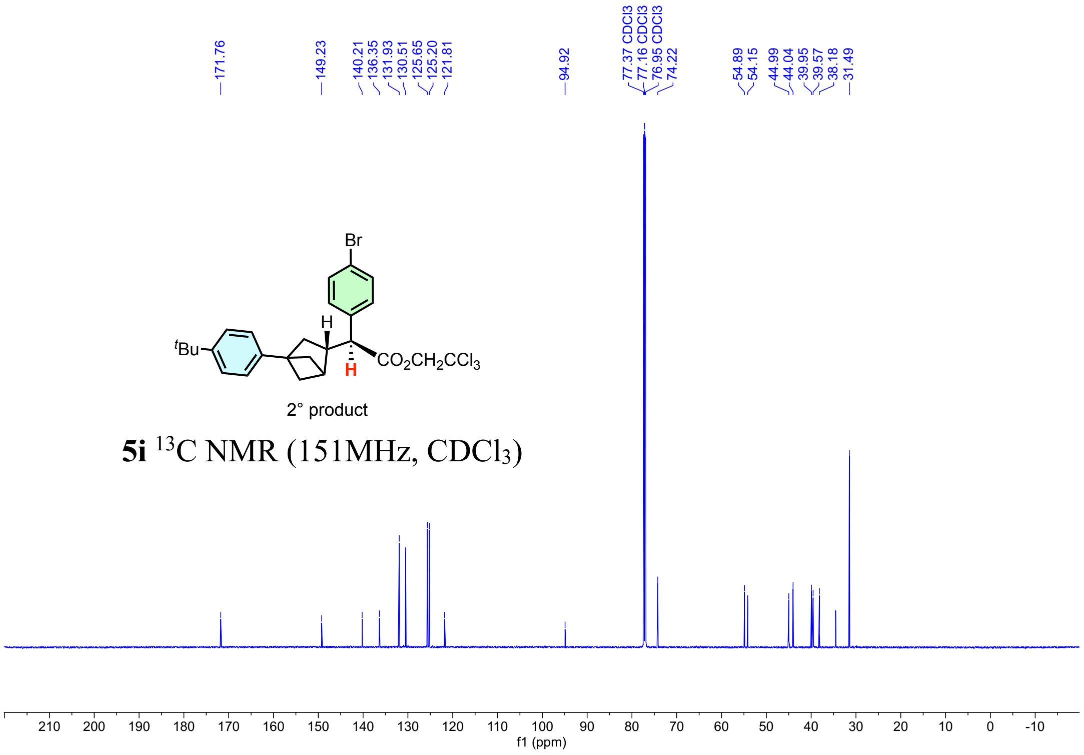
171.76
149.23
140.21
136.35
131.93
130.51
125.65
125.20
121.81
94.92
77.37 CDCl3
77.16 CDCl3
76.95 CDCl3
74.22
54.89
54.15
44.99
44.04
39.95
39.57
38.18
31.49
Br
tBu
H
H
CO2CH2CCl3
2° product
5i 13C NMR (151MHz, CDCl3)
210
200
190
180
170
160
150
140
130
120
110
100
90
80
70
60
50
40
30
20
10
0
-10
f1 (ppm)

7i ^{1}H NMR (400MHz, $CDCl_3$)

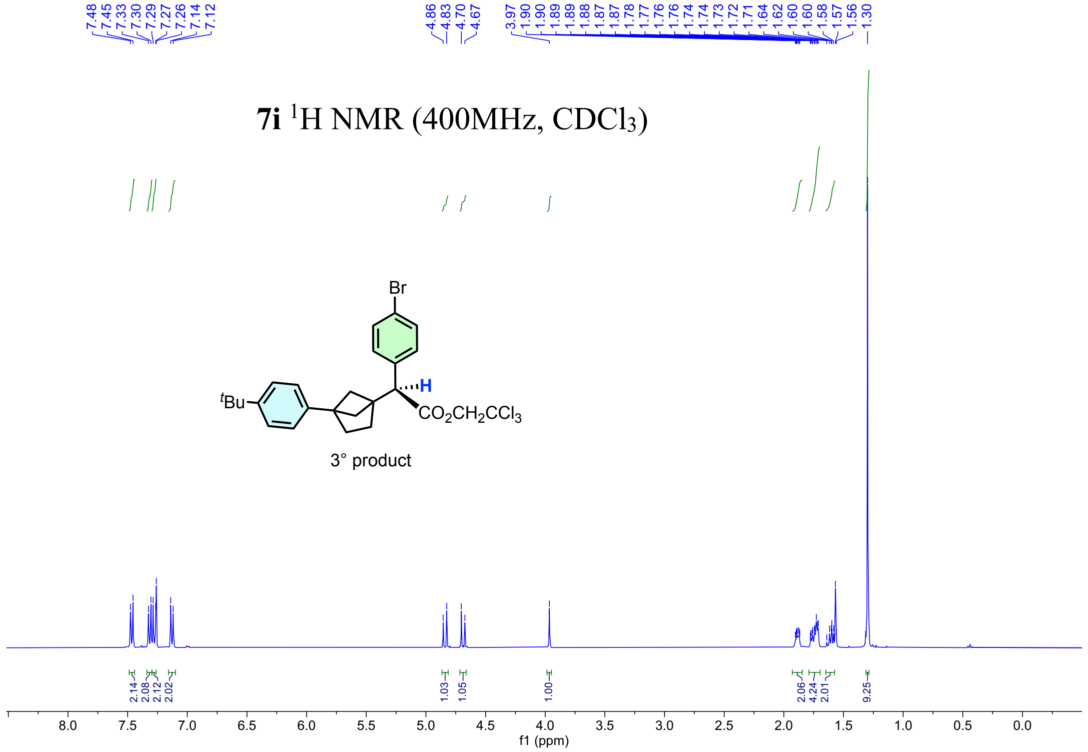

7i ^{13}C NMR (151MHz, $CDCl_3$)

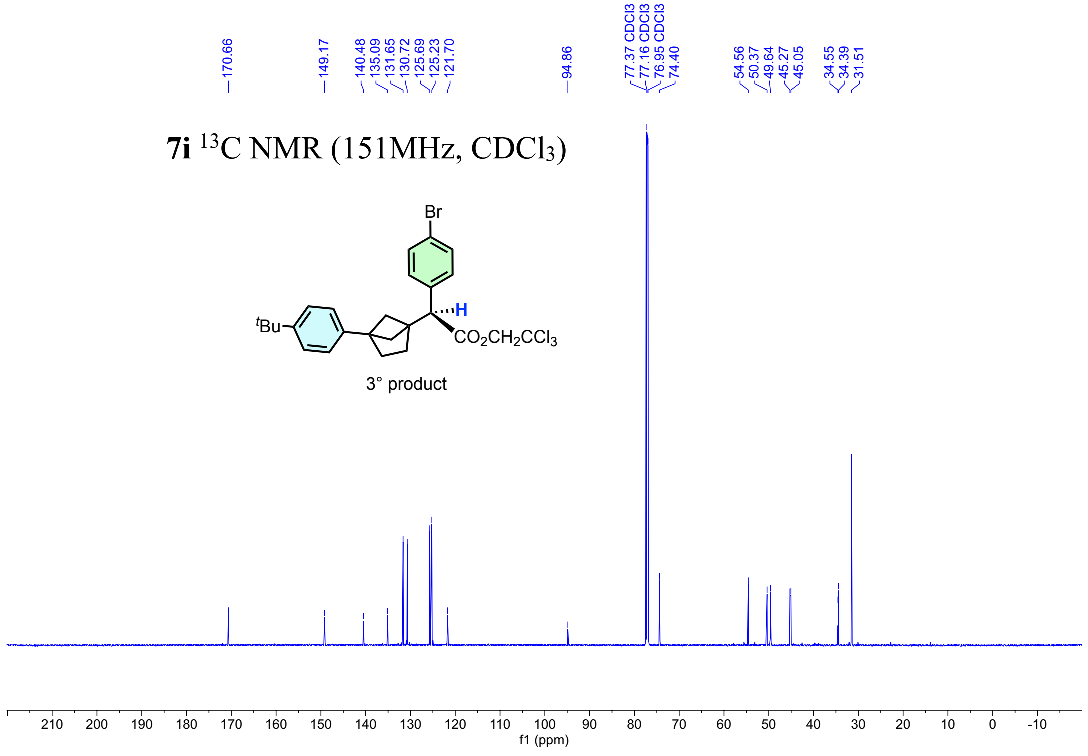

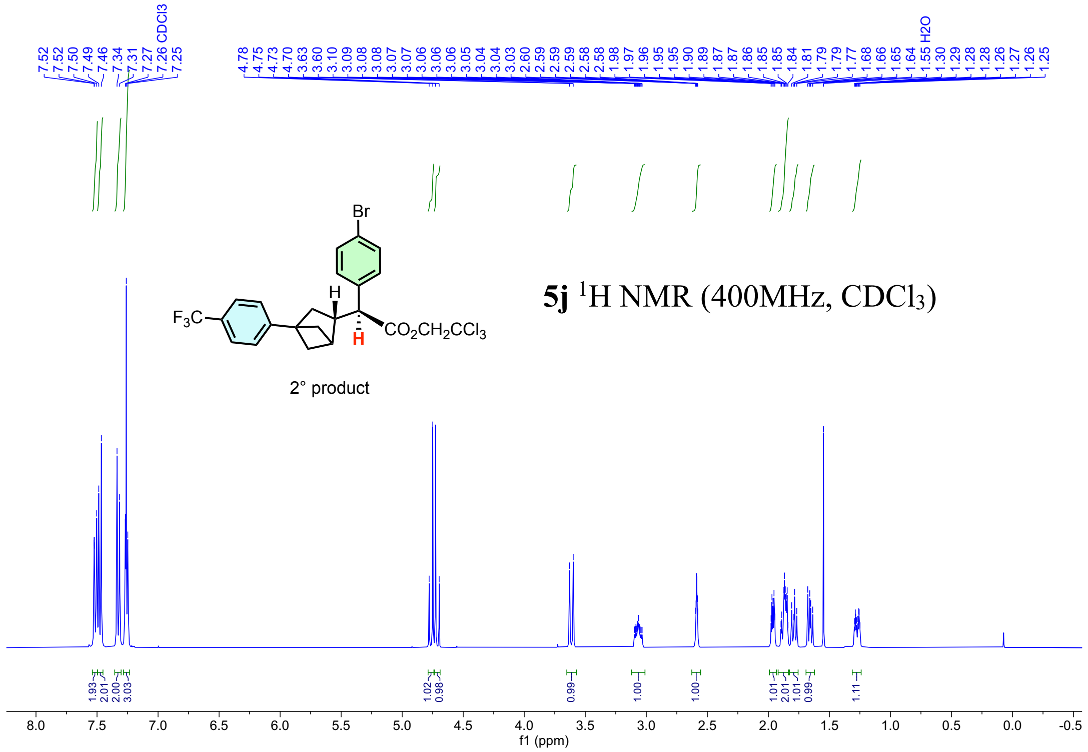
5j ¹H NMR (400MHz, CDCl3)
Br
H
F3C
H
CO2CH2CCl3
2° product
f1 (ppm)

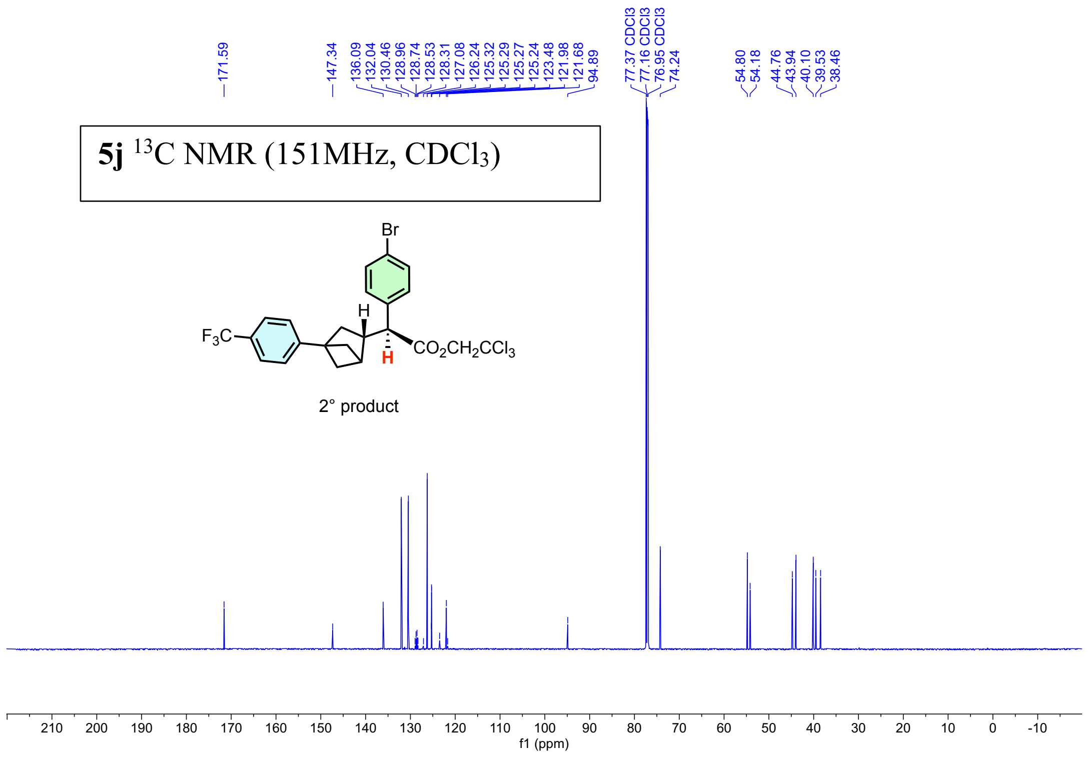
5j ¹³C NMR (151MHz, CDCl3)
Br
H
F3C
H
CO2CH2CCl3
2° product
f1 (ppm)

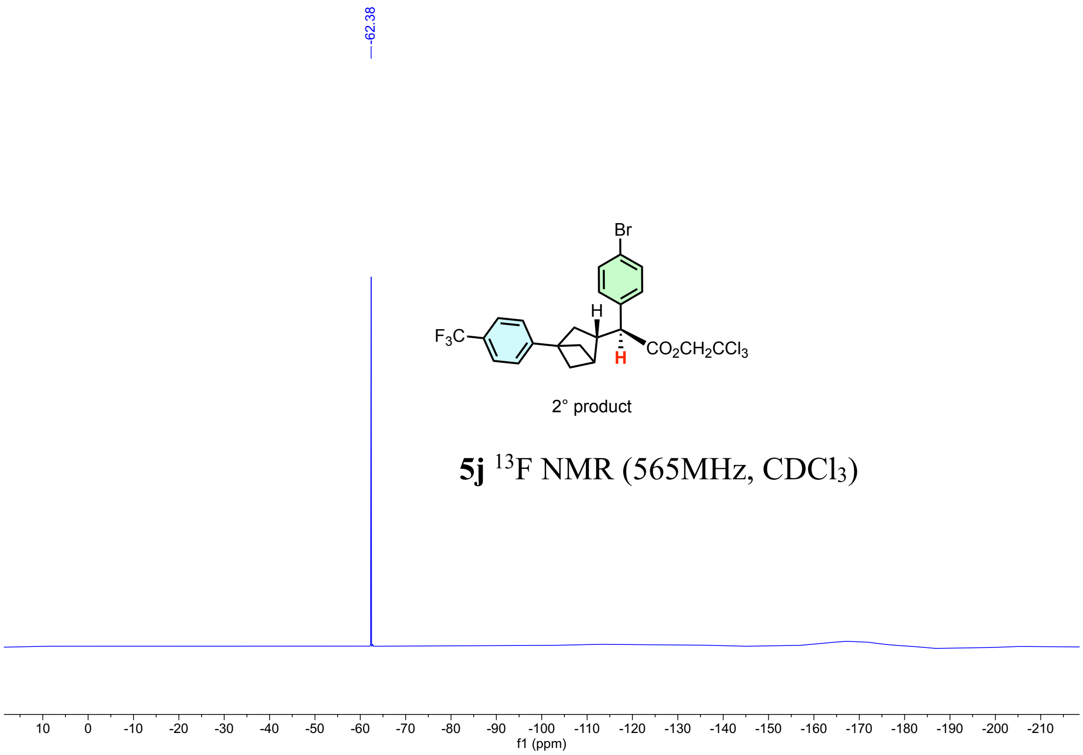

5j [13]F NMR (565MHz, CDCl3)

7j ^{1}H NMR (400MHz, CDCl3)

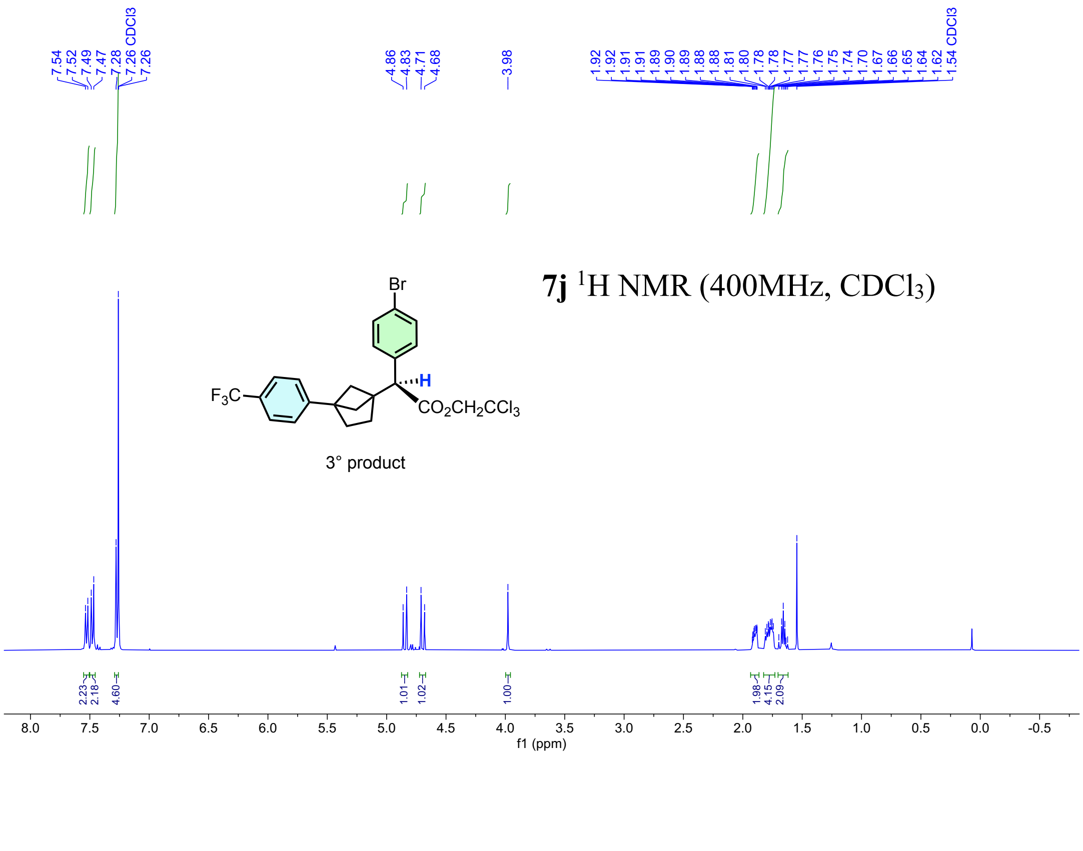

7j ^{13}C NMR (151MHz, $CDCl_3$)

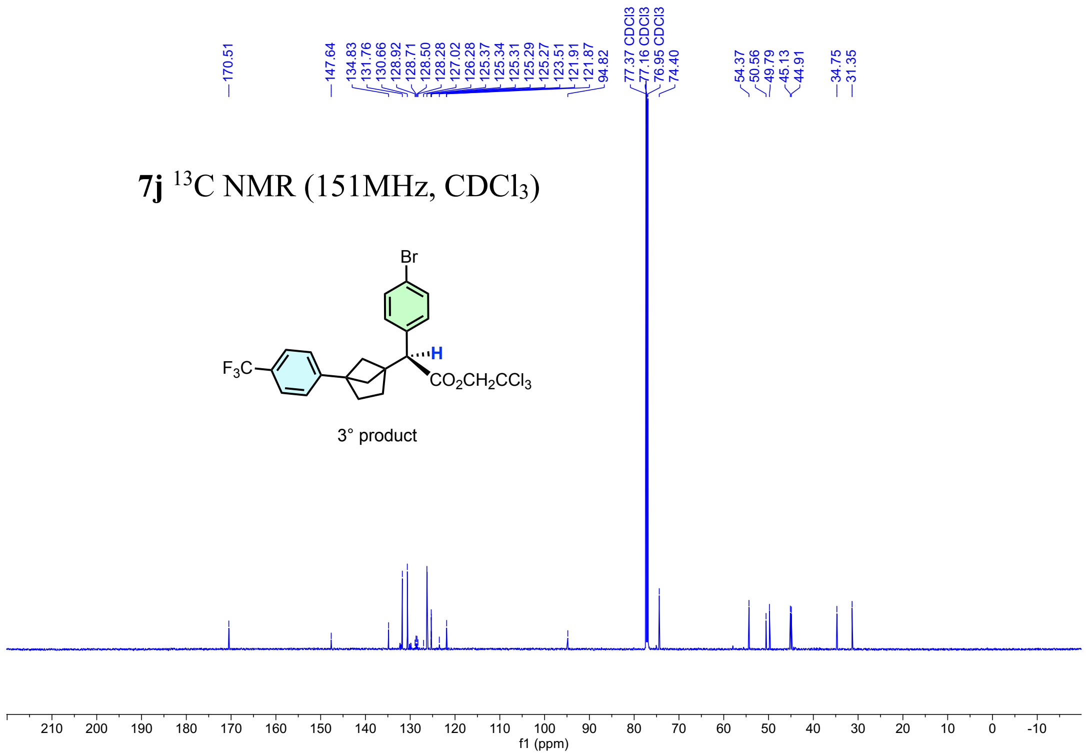

7j ^{19}F NMR (565MHz, $CDCl_3$)

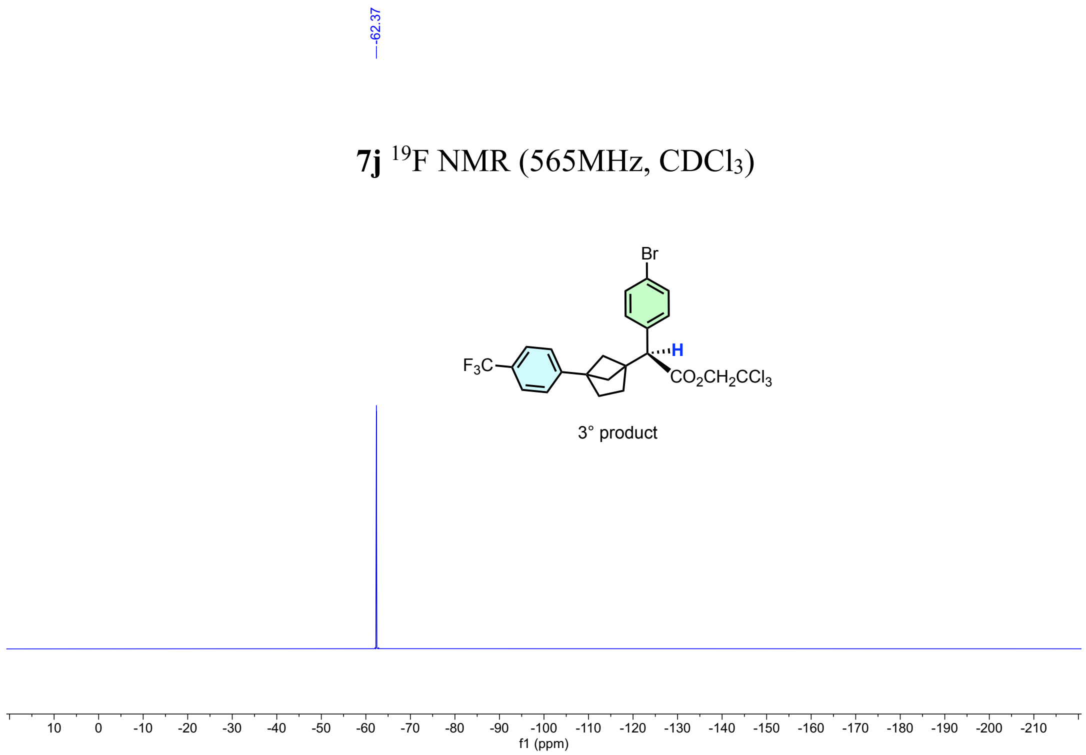

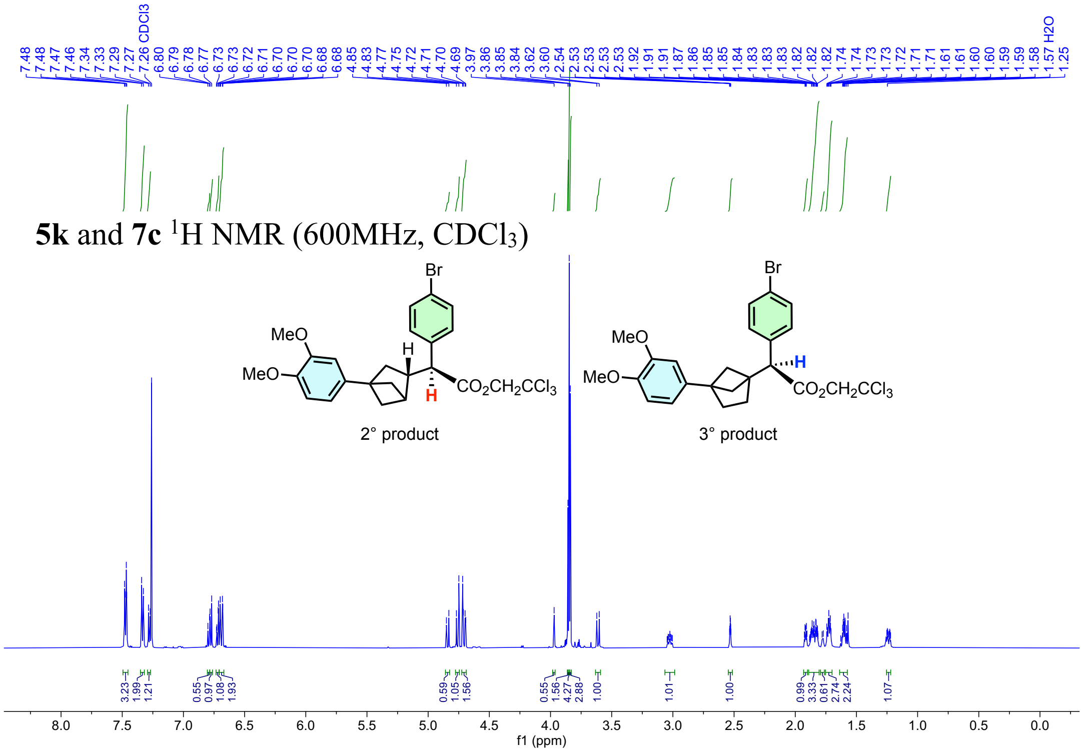
5k and 7c ¹H NMR (600MHz, CDCl₃)
Br
MeO
MeO
H
H
CO2CH2CCl3
2° product
3° product
f1 (ppm)

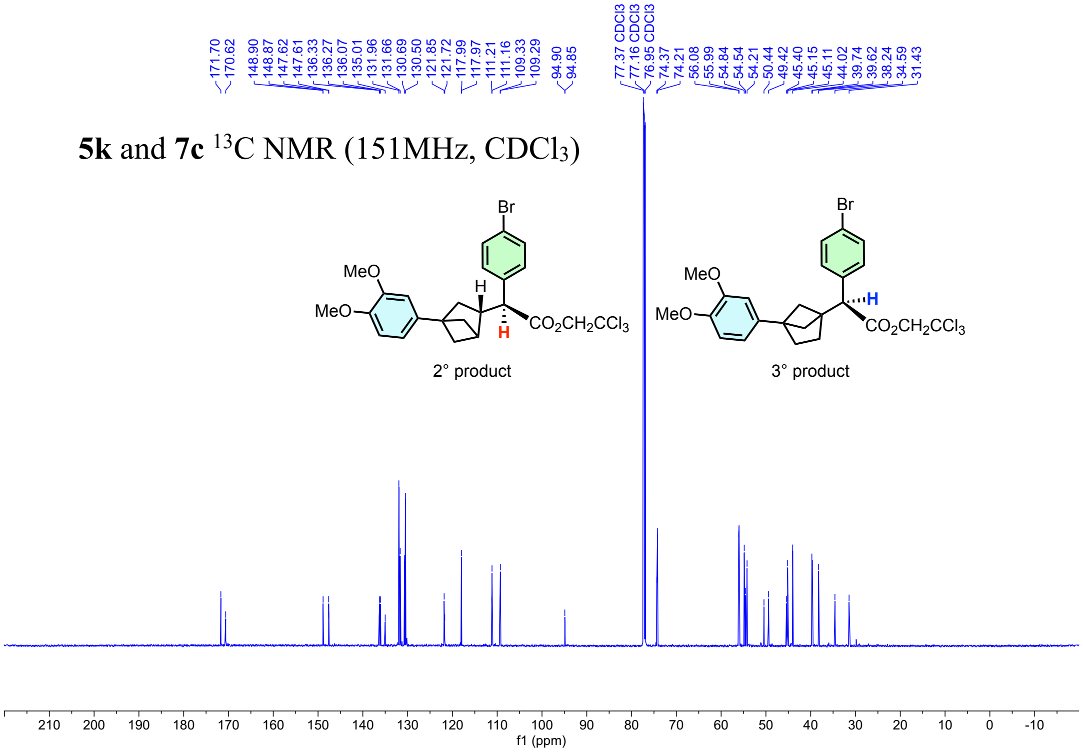
5k and 7c ¹³C NMR (151MHz, CDCl₃)
Br
MeO
MeO
H
H
CO2CH2CCl3
2° product
3° product
f1 (ppm)

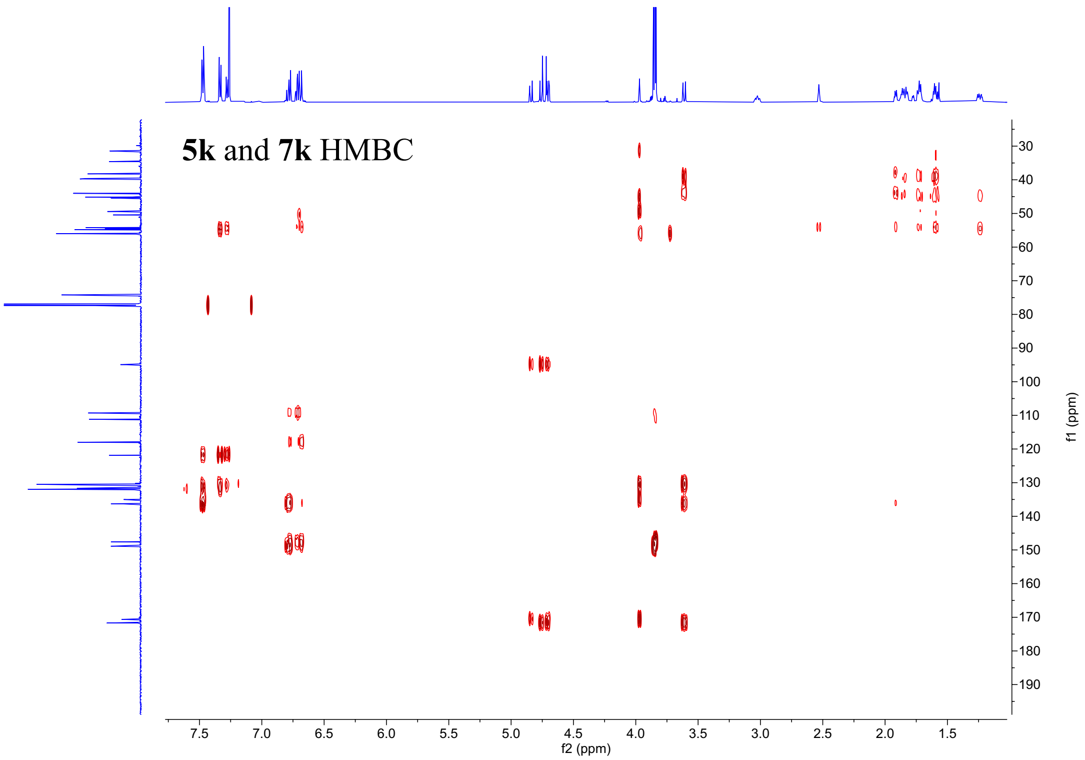
5k and **7k** HMBC
f1 (ppm)
f2 (ppm)

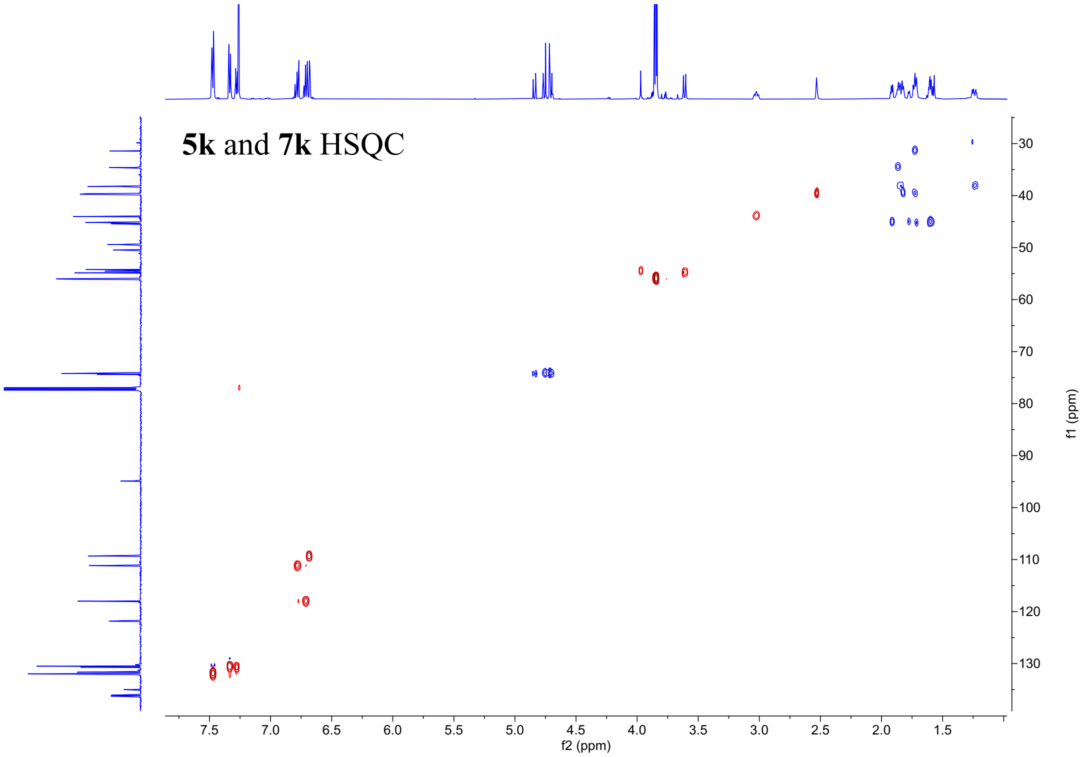
5k and **7k** HSQC
f1 (ppm)
f2 (ppm)

5l ^{1}H NMR (400MHz, $CDCl_3$)

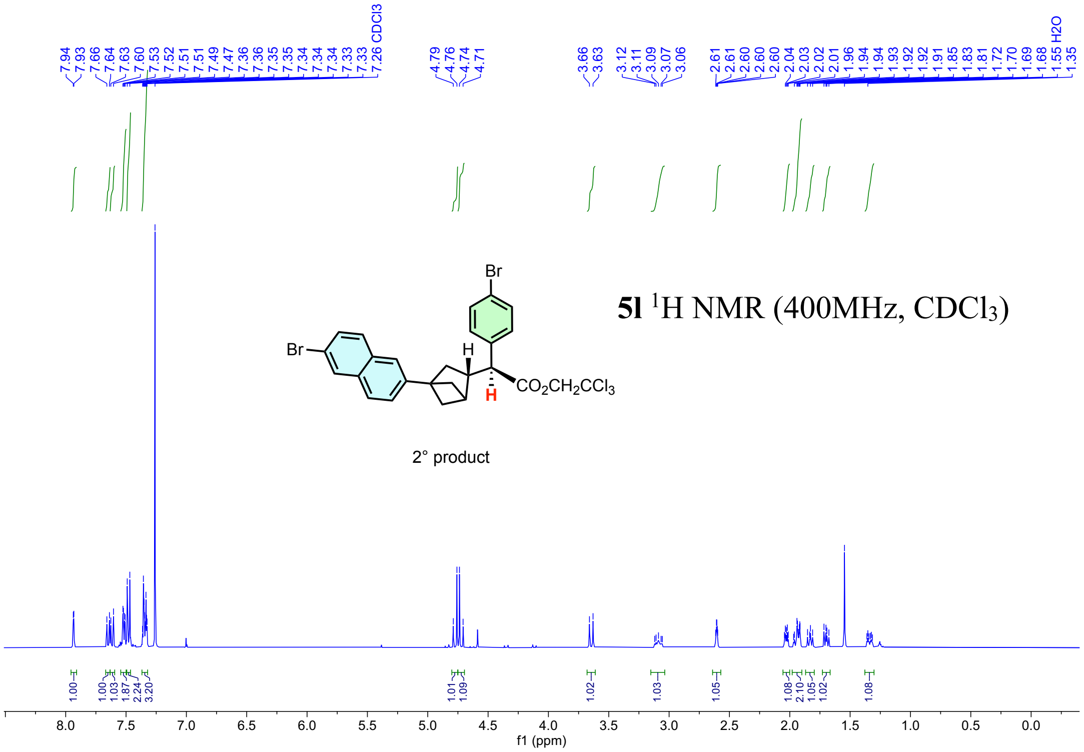

5l ^{13}C NMR (151MHz, $CDCl_3$)

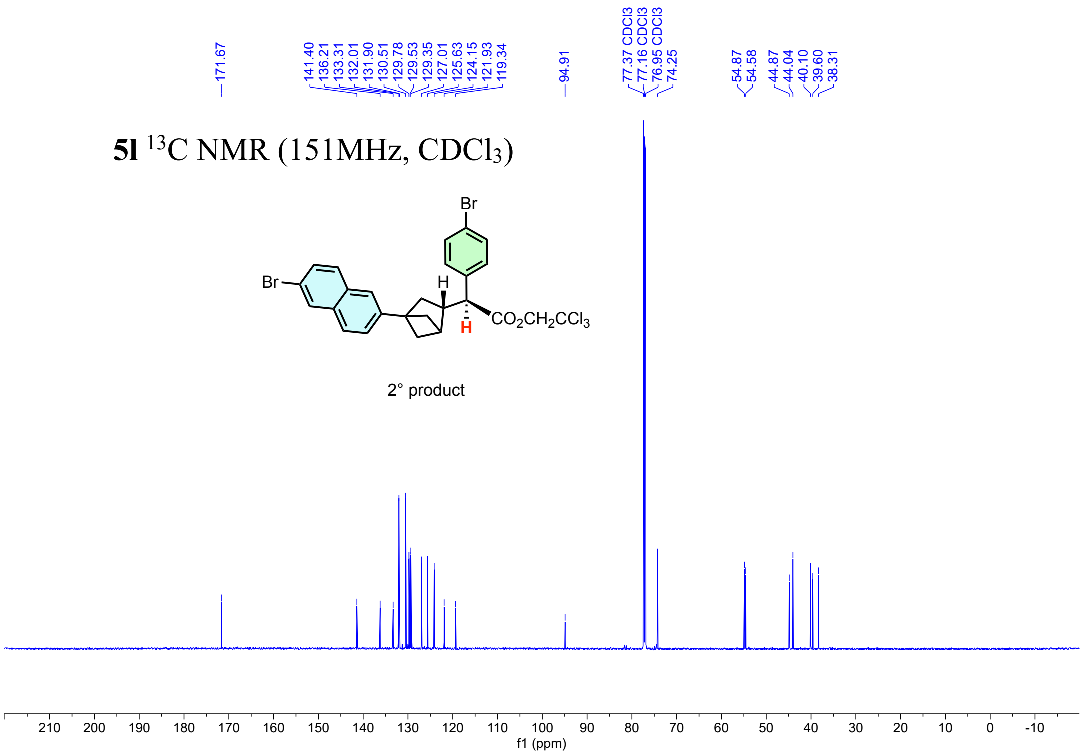

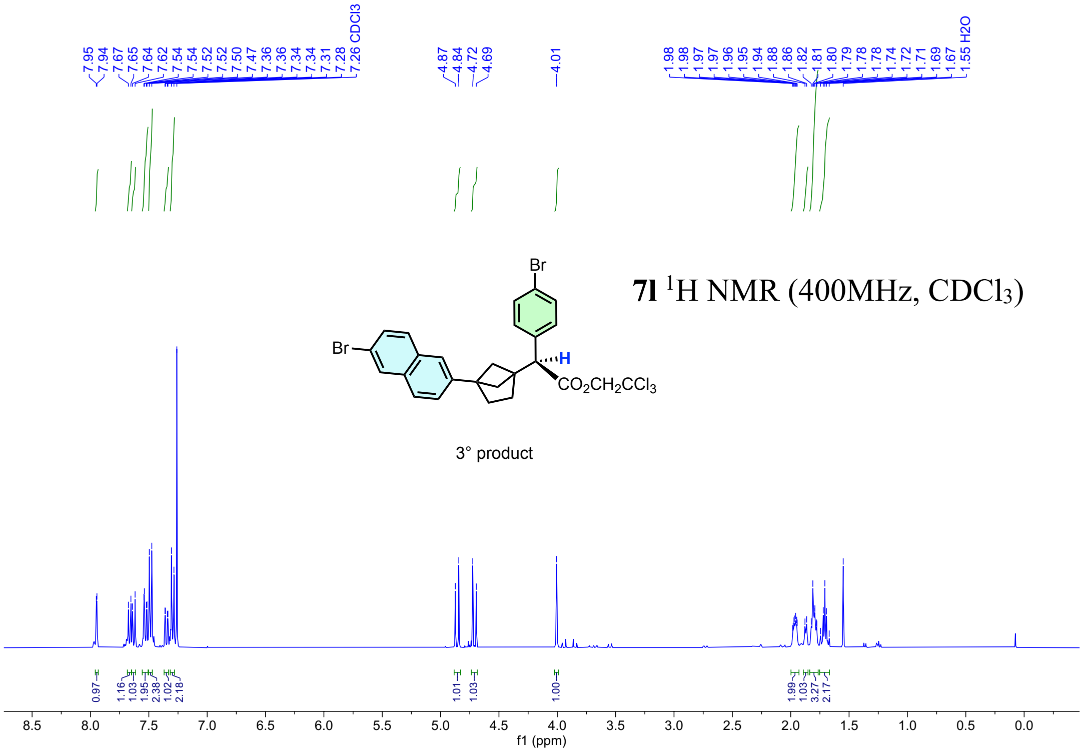

7l ^{1}H NMR (400MHz, $CDCl_3$)

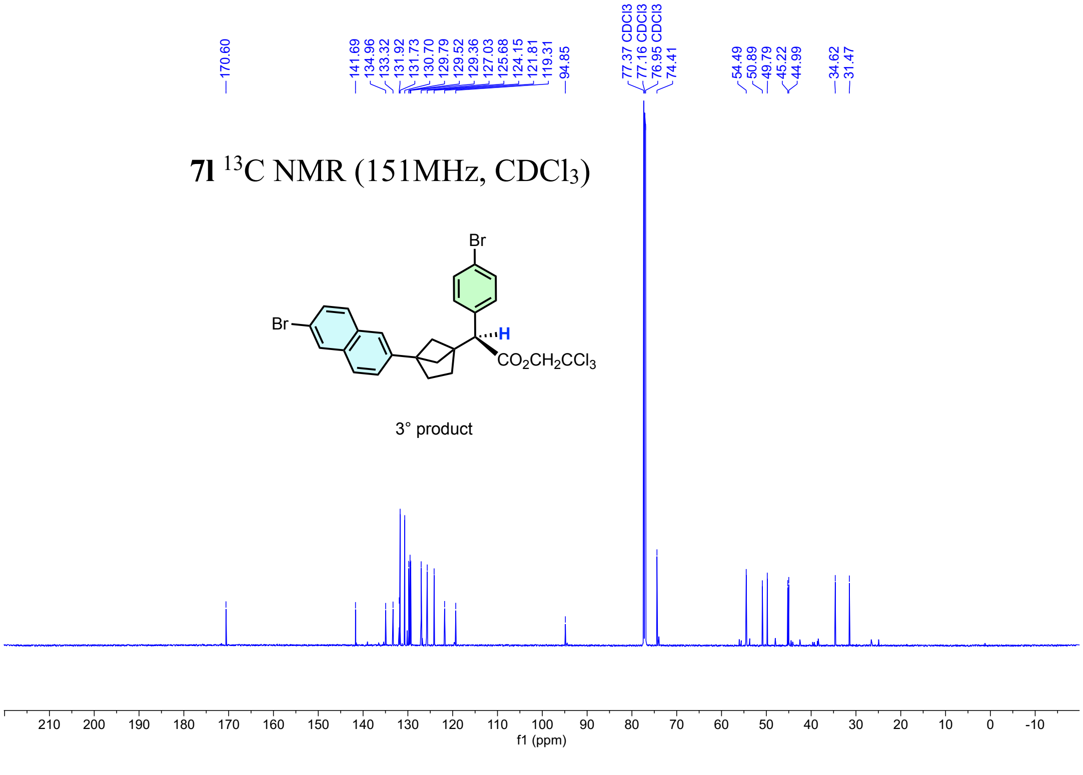

7l ^{13}C NMR (151MHz, $CDCl_3$)

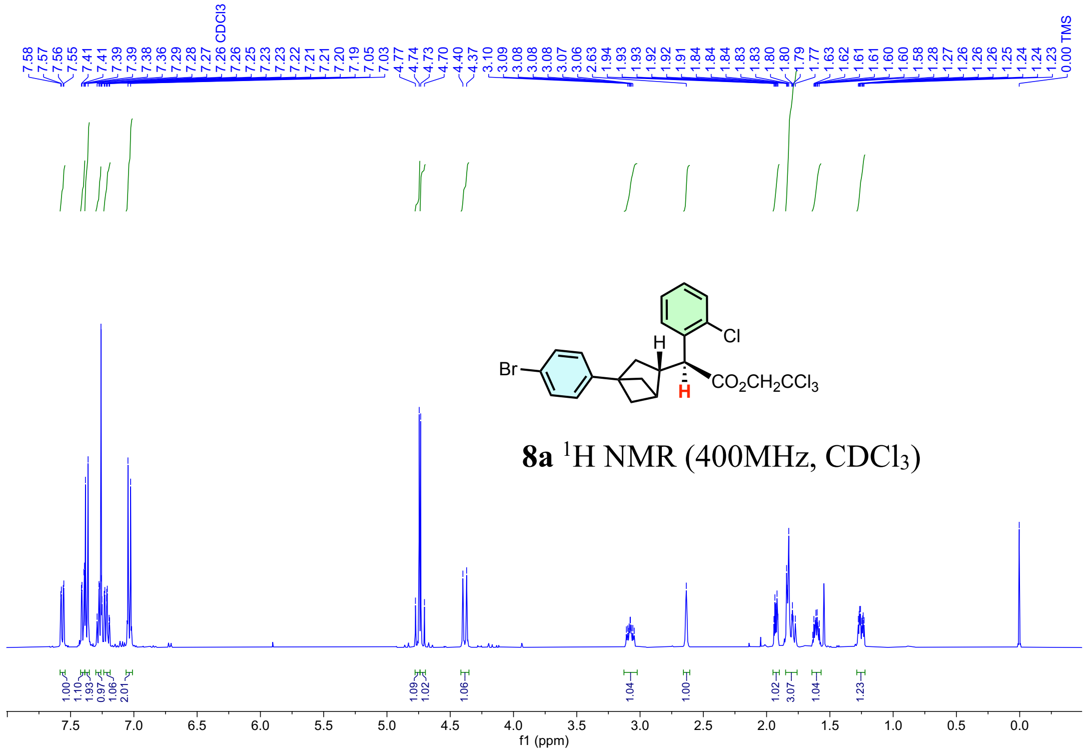
Br
H
Cl
H
CO2CH2CCl3
8a 1H NMR (400MHz, CDCl3)
f1 (ppm)

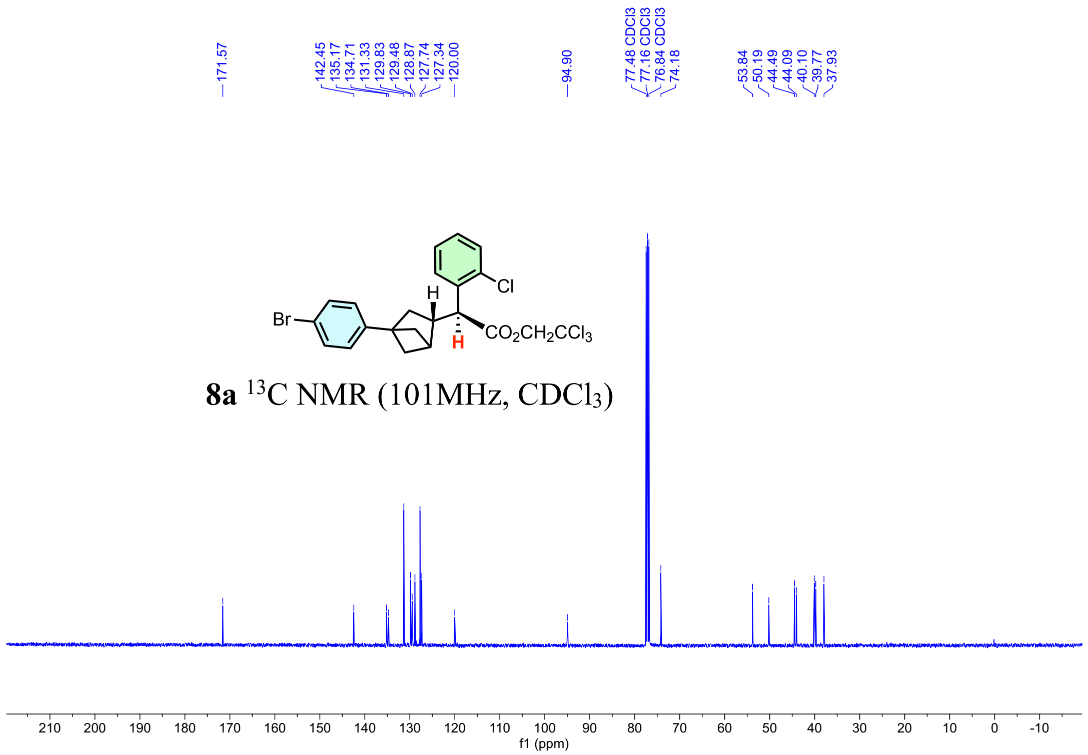
Br
H
Cl
H
CO2CH2CCl3
8a 13C NMR (101MHz, CDCl3)
f1 (ppm)

8b ^{1}H NMR (400MHz, $CDCl_3$)

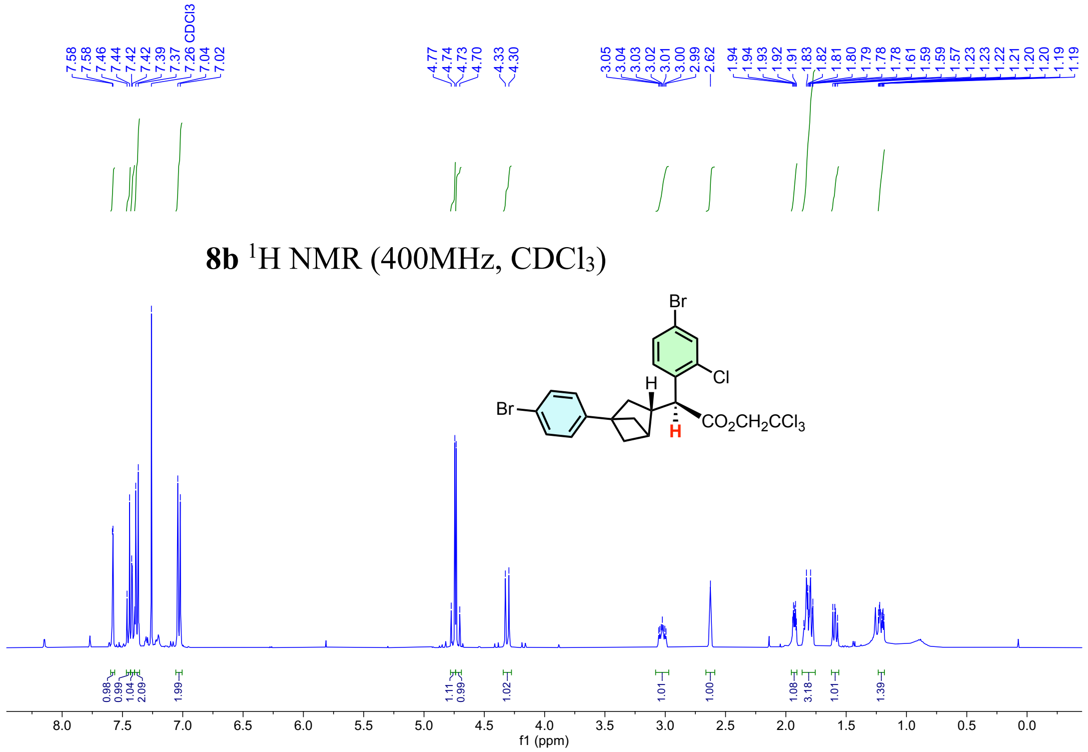

8b ^{13}C NMR (101MHz, $CDCl_3$)

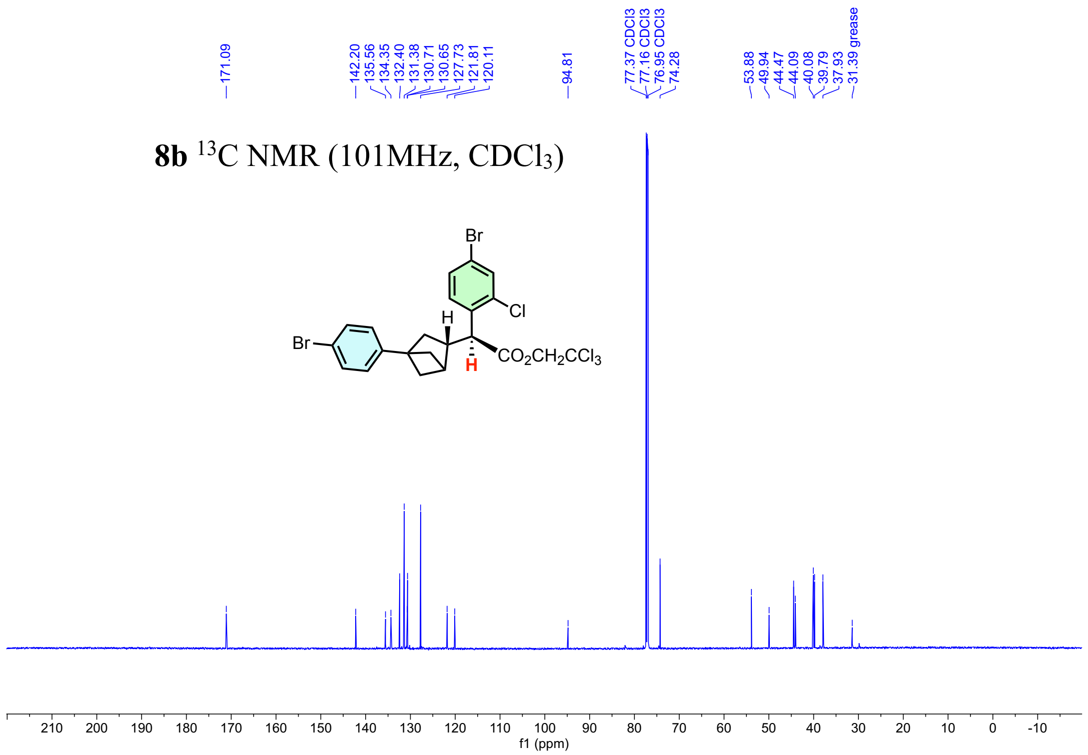

11a ^{1}H NMR (400MHz, $CDCl_3$)

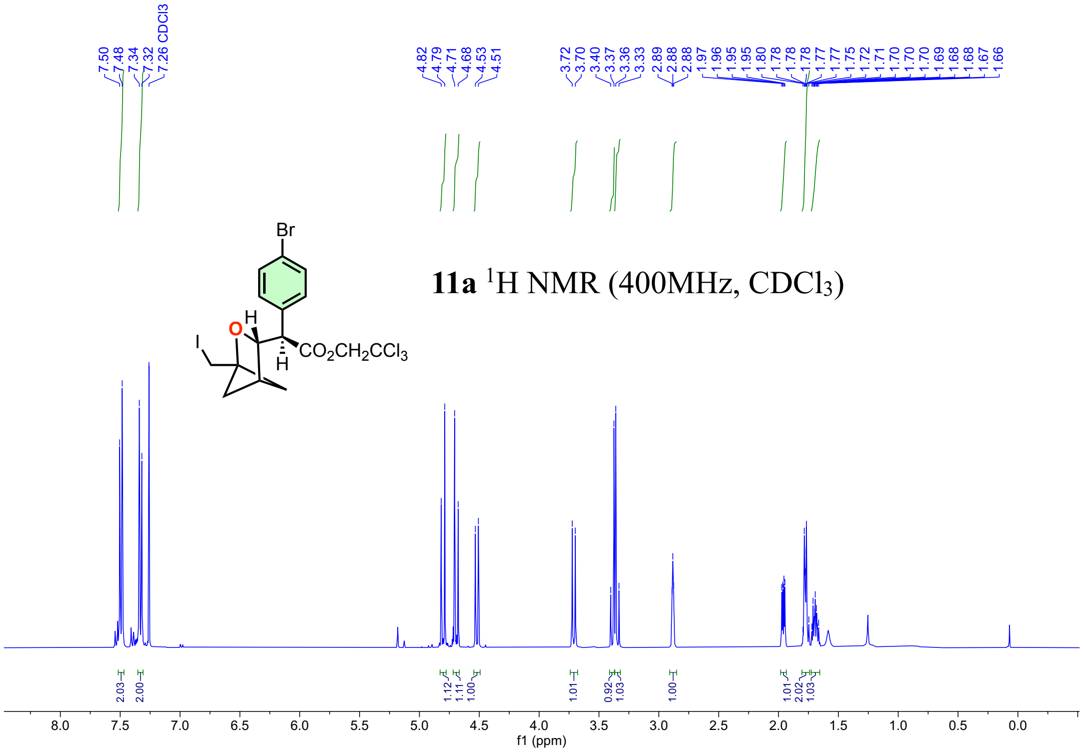

11a ^{13}C NMR (101MHz, $CDCl_3$)

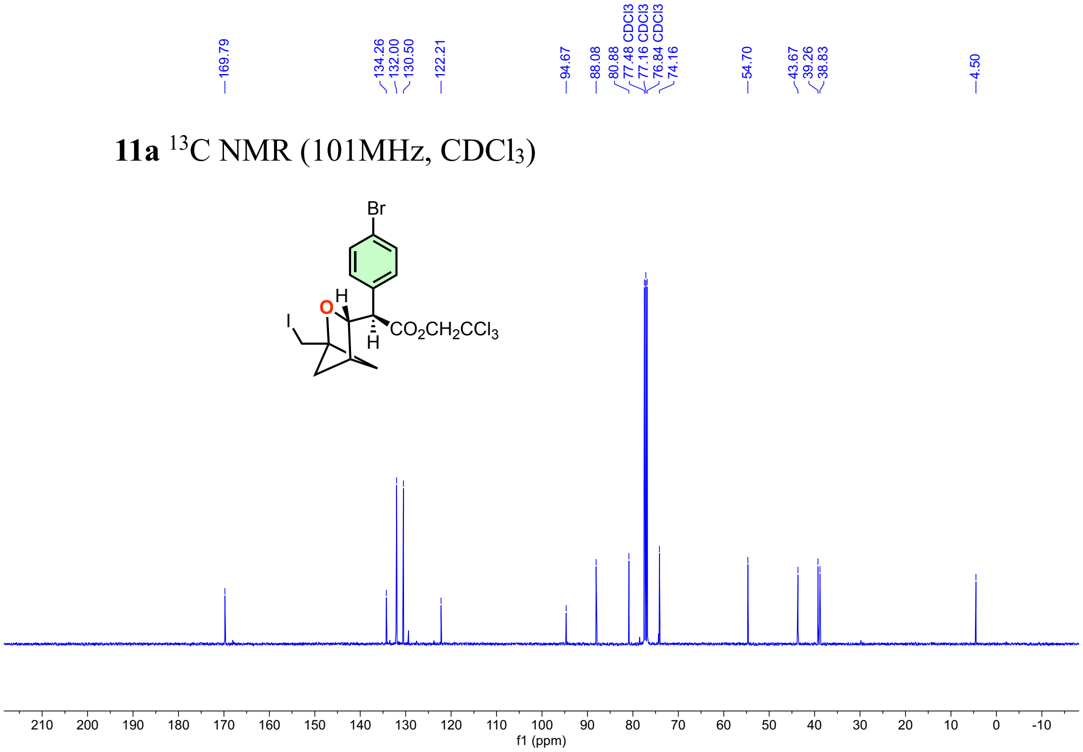

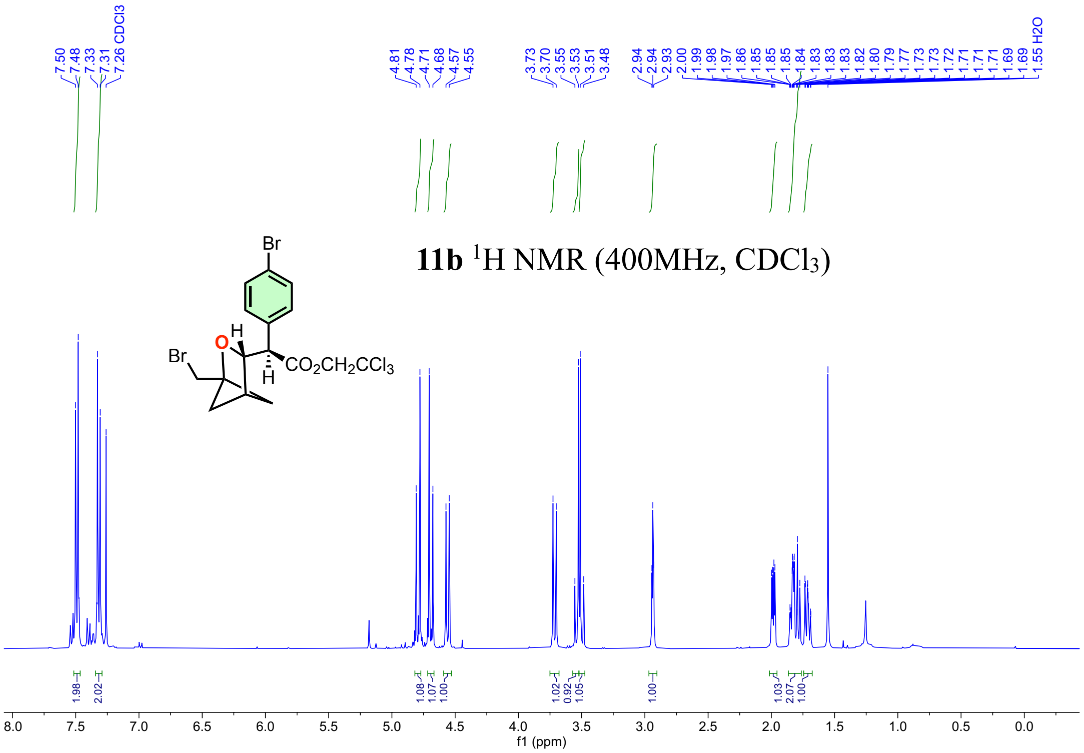
7.50
7.48
7.33
7.31
7.26 CDCl3
4.81
4.78
4.71
4.68
4.57
4.55
3.73
3.70
3.55
3.53
3.51
3.48
2.94
2.94
2.93
2.00
1.99
1.98
1.97
1.86
1.85
1.85
1.85
1.84
1.83
1.83
1.83
1.82
1.80
1.79
1.77
1.73
1.73
1.72
1.71
1.71
1.69
1.69
1.55 H2O
11b ^{1}H NMR (400MHz, $CDCl_3$)
Br
O
H
H
$CO_2CH_2CCl_3$
1.98
2.02
1.08
1.07
1.00
1.02
0.92
1.05
1.00
1.03
2.07
1.00
8.0 7.5 7.0 6.5 6.0 5.5 5.0 4.5 4.0 3.5 3.0 2.5 2.0 1.5 1.0 0.5 0.0
f1 (ppm)

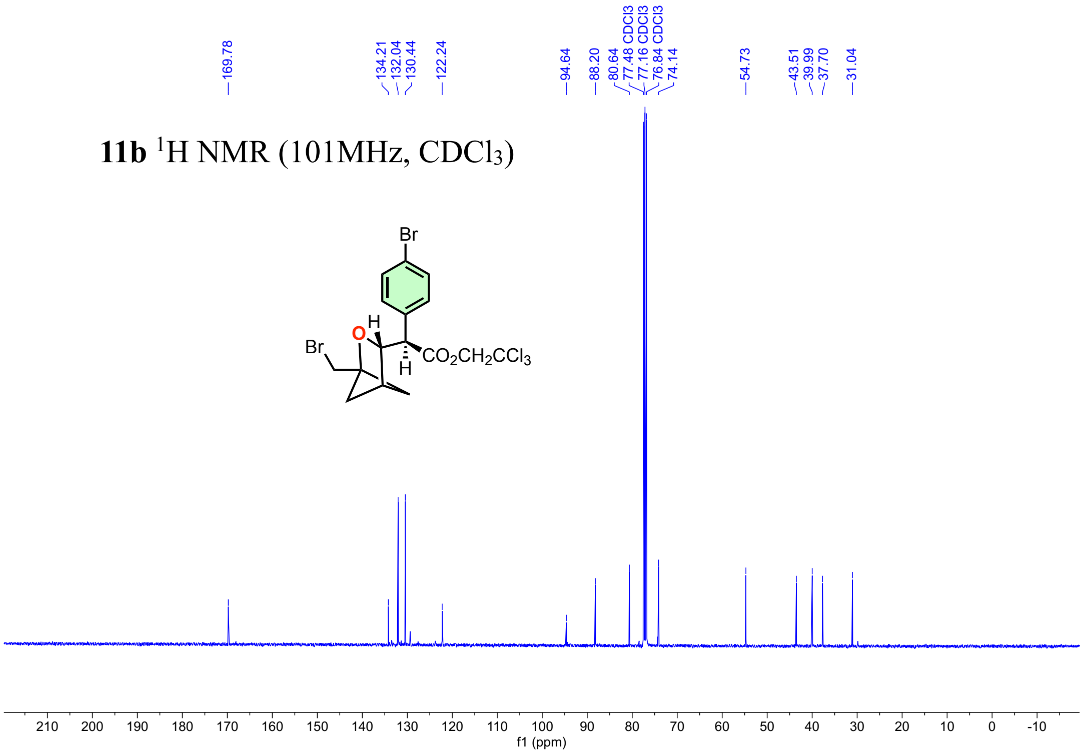
169.78
134.21
132.04
130.44
122.24
94.64
88.20
80.64
77.48 CDCl3
77.16 CDCl3
76.84 CDCl3
74.14
54.73
43.51
39.99
37.70
31.04
11b ^{1}H NMR (101MHz, $CDCl_3$)
Br
O
H
H
$CO_2CH_2CCl_3$
210 200 190 180 170 160 150 140 130 120 110 100 90 80 70 60 50 40 30 20 10 0 -10
f1 (ppm)

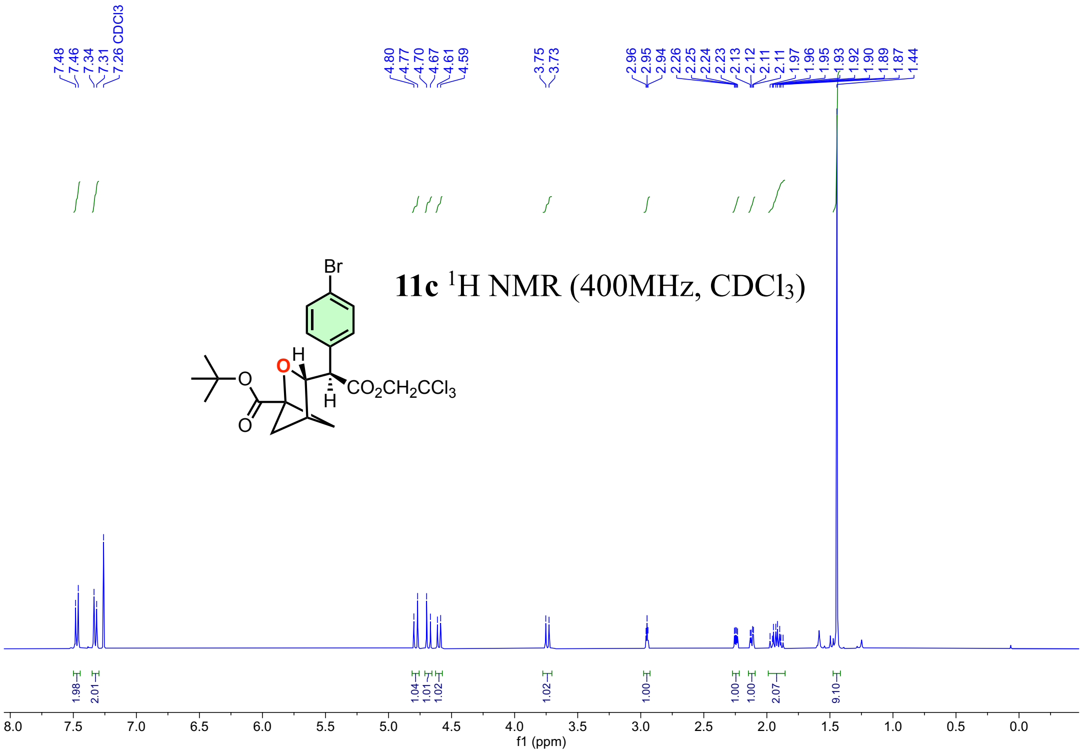

7.48
7.46
7.34
7.31
7.26 CDCl3
4.80
4.77
4.70
4.67
4.61
4.59
3.75
3.73
2.96
2.95
2.94
2.26
2.25
2.24
2.23
2.13
2.12
2.11
2.11
1.97
1.96
1.95
1.93
1.92
1.90
1.89
1.87
1.44
11c ^{1}H NMR (400MHz, $CDCl_3$)
Br
O
H
H
$CO_2CH_2CCl_3$
O
O
1.98
2.01
1.04
1.01
1.02
1.02
1.00
1.00
1.00
2.07
9.10
f1 (ppm)

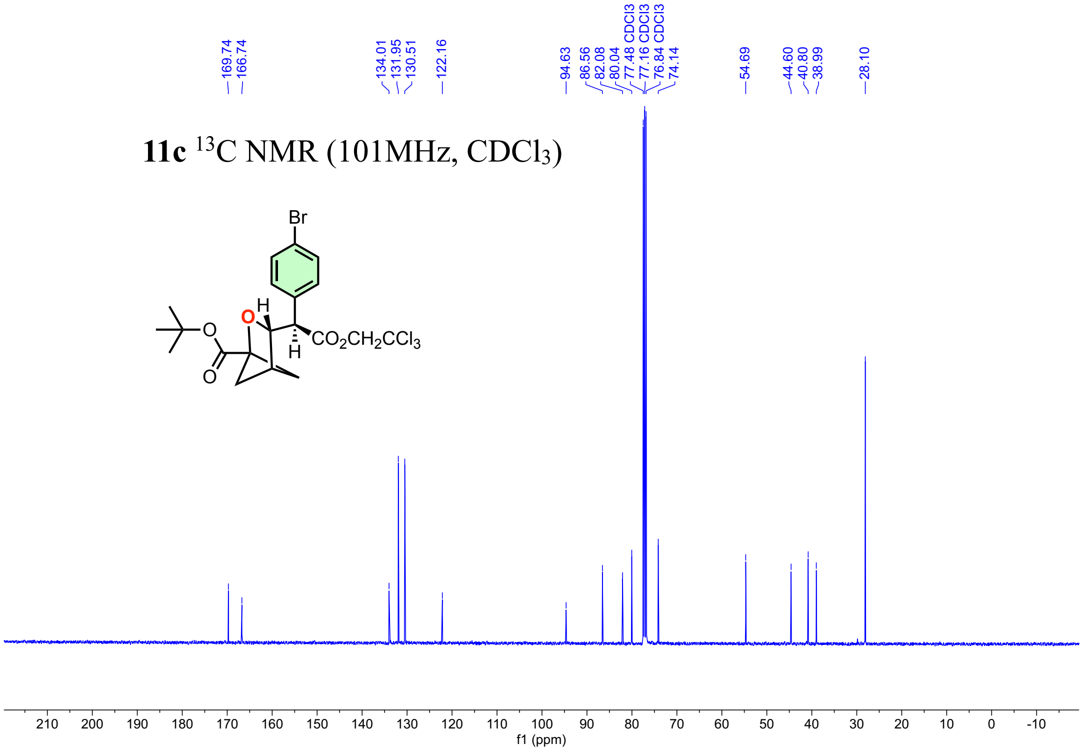

169.74
166.74
134.01
131.95
130.51
122.16
94.63
86.56
82.08
80.04
77.48 CDCl3
77.16 CDCl3
76.84 CDCl3
74.14
54.69
44.60
40.80
38.99
28.10
11c ^{13}C NMR (101MHz, $CDCl_3$)
Br
O
H
H
$CO_2CH_2CCl_3$
O
O
f1 (ppm)

11d ^{1}H NMR (400MHz, $CDCl_3$)

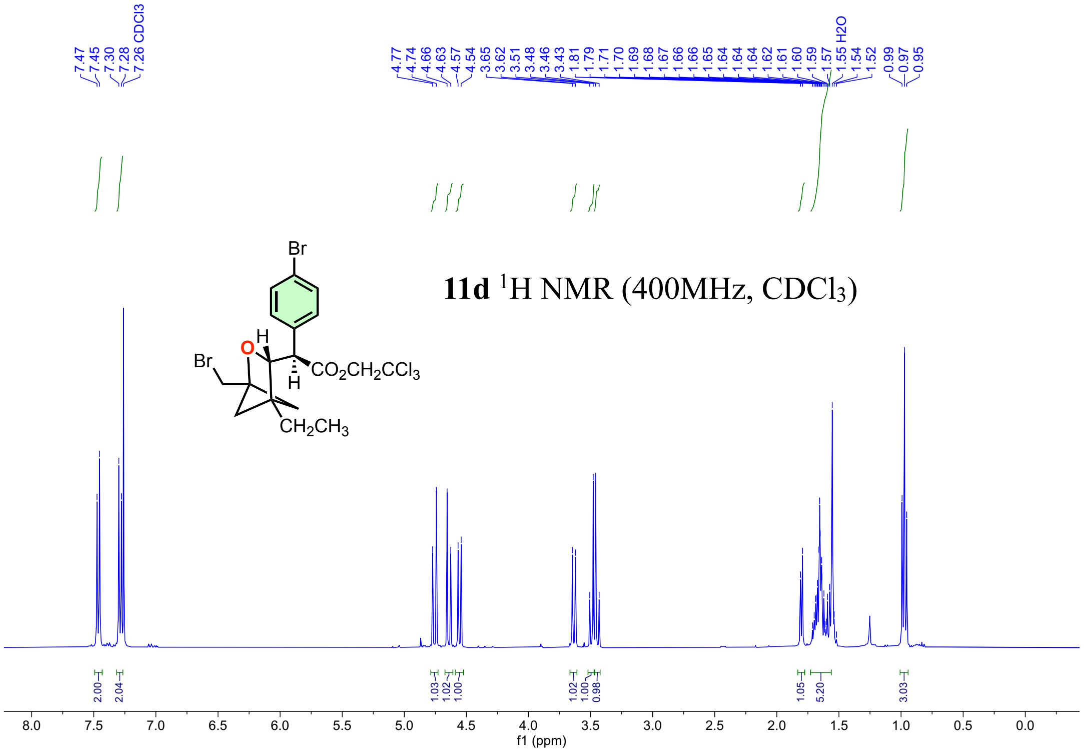

11d ^{13}C NMR (101MHz, $CDCl_3$)

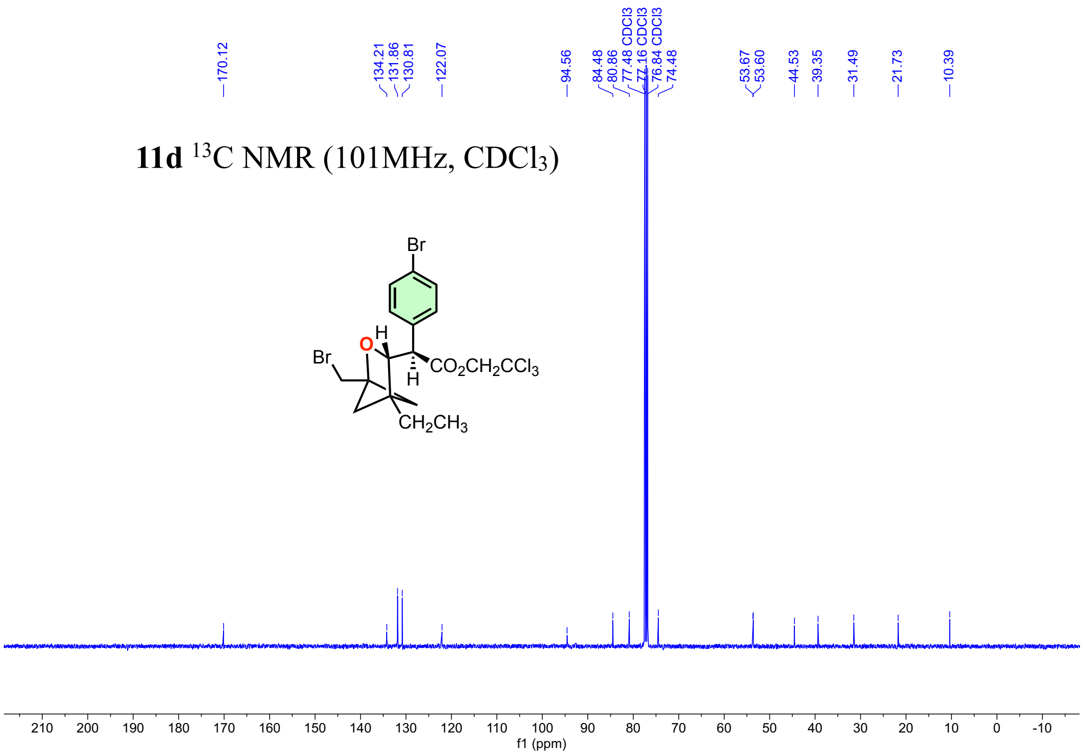

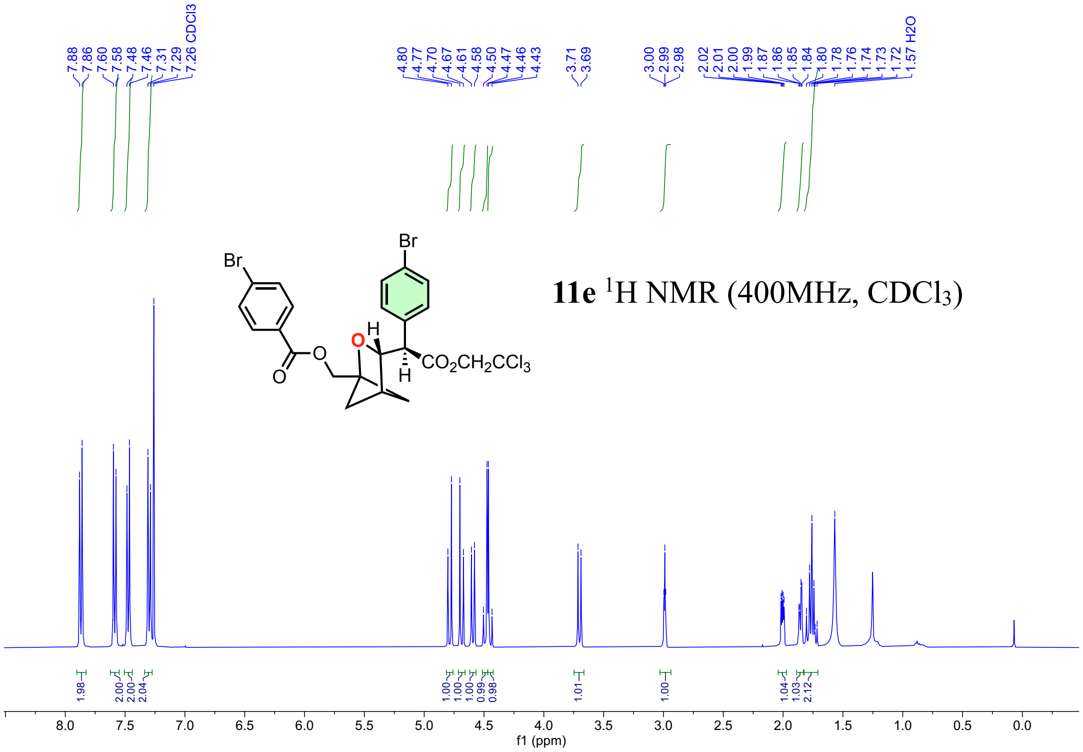

11e ^{1}H NMR (400MHz, $CDCl_3$)

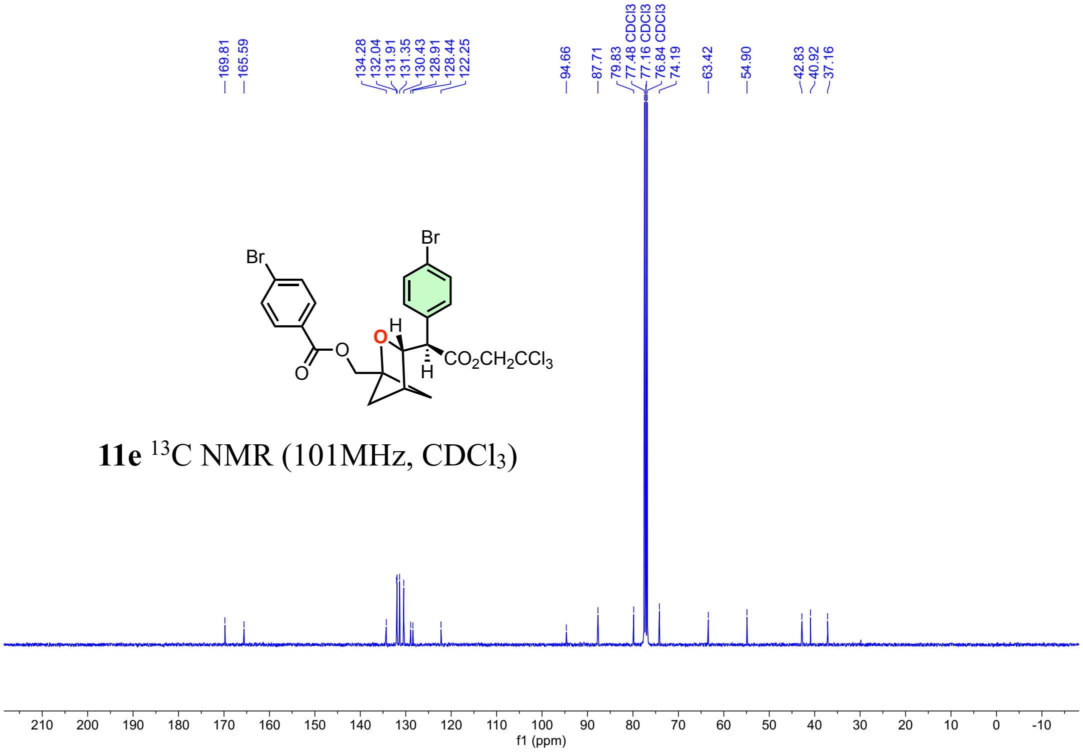

11e ^{13}C NMR (101MHz, $CDCl_3$)

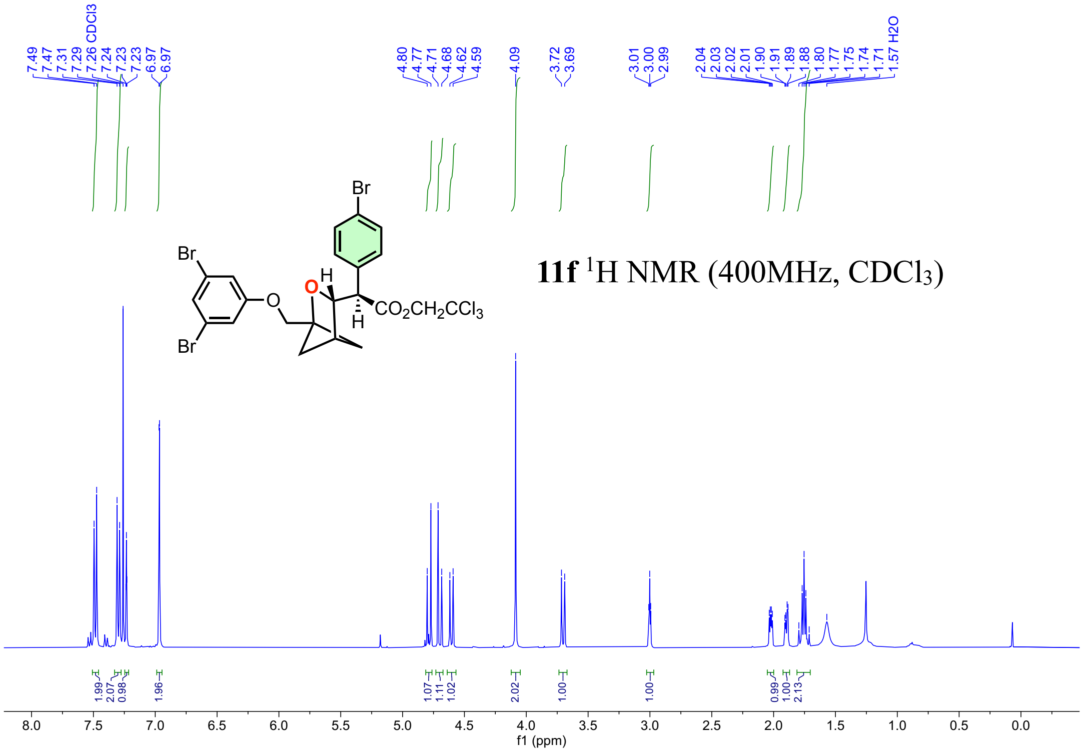
7.49
7.47
7.31
7.29
7.26 CDCl3
7.24
7.23
7.23
6.97
6.97
4.80
4.77
4.71
4.68
4.62
4.59
4.09
3.72
3.69
3.01
3.00
2.99
2.04
2.03
2.02
2.01
1.90
1.91
1.89
1.88
1.80
1.77
1.75
1.74
1.71
1.57 H2O
11f 1H NMR (400MHz, CDCl3)
Br
Br
Br
O
O
H
H
CO2CH2CCl3
1.99
2.07
0.98
1.96
1.07
1.11
1.02
2.02
1.00
1.00
0.99
1.00
2.13
f1 (ppm)

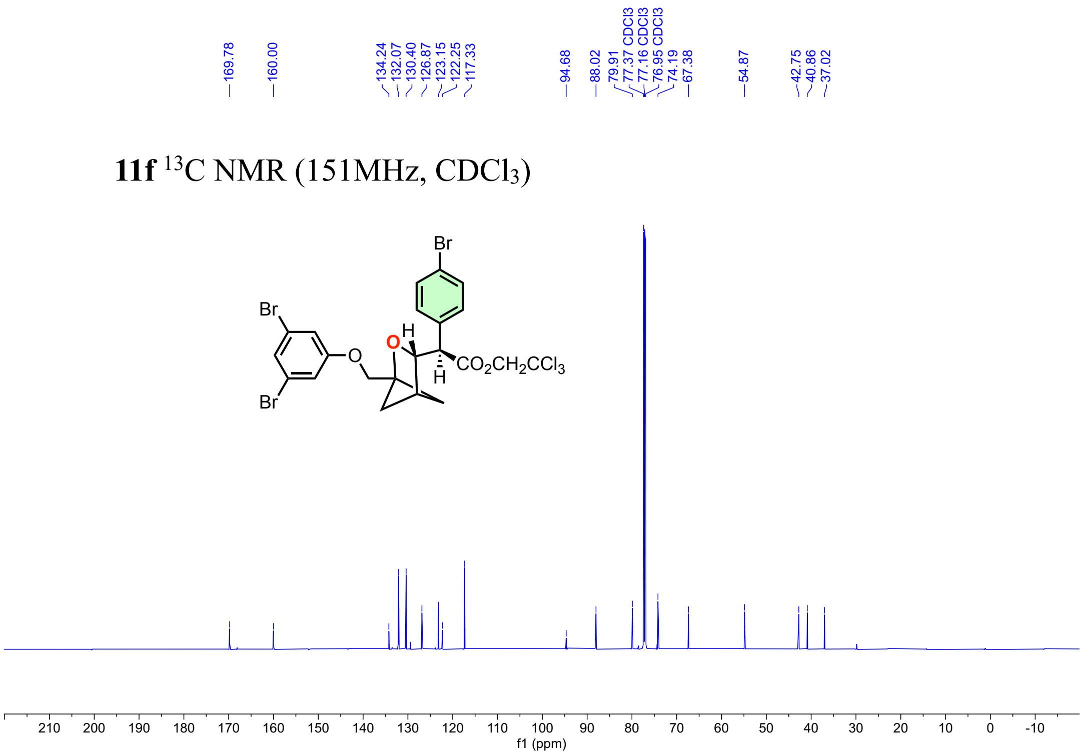
169.78
160.00
134.24
132.07
130.40
126.87
123.15
122.25
117.33
94.68
88.02
79.91
77.37 CDCl3
77.16 CDCl3
76.95 CDCl3
74.19
67.38
54.87
42.75
40.86
37.02
11f 13C NMR (151MHz, CDCl3)
Br
Br
Br
O
O
H
H
CO2CH2CCl3
f1 (ppm)

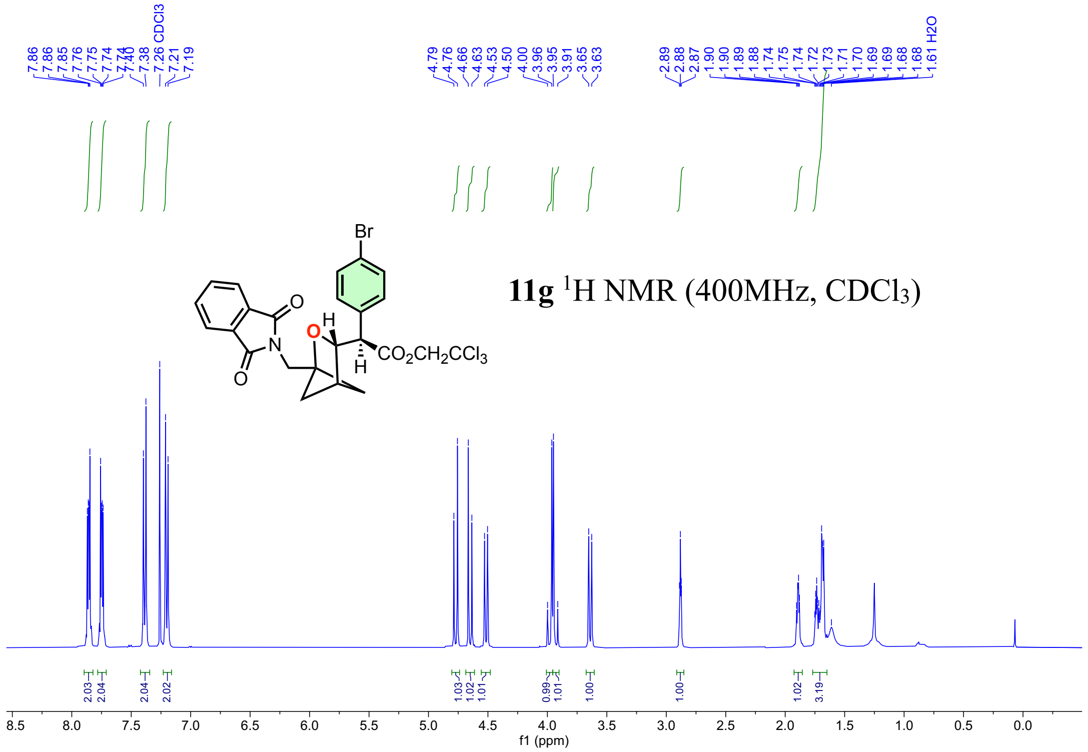

11g ^{1}H NMR (400MHz, $CDCl_3$)

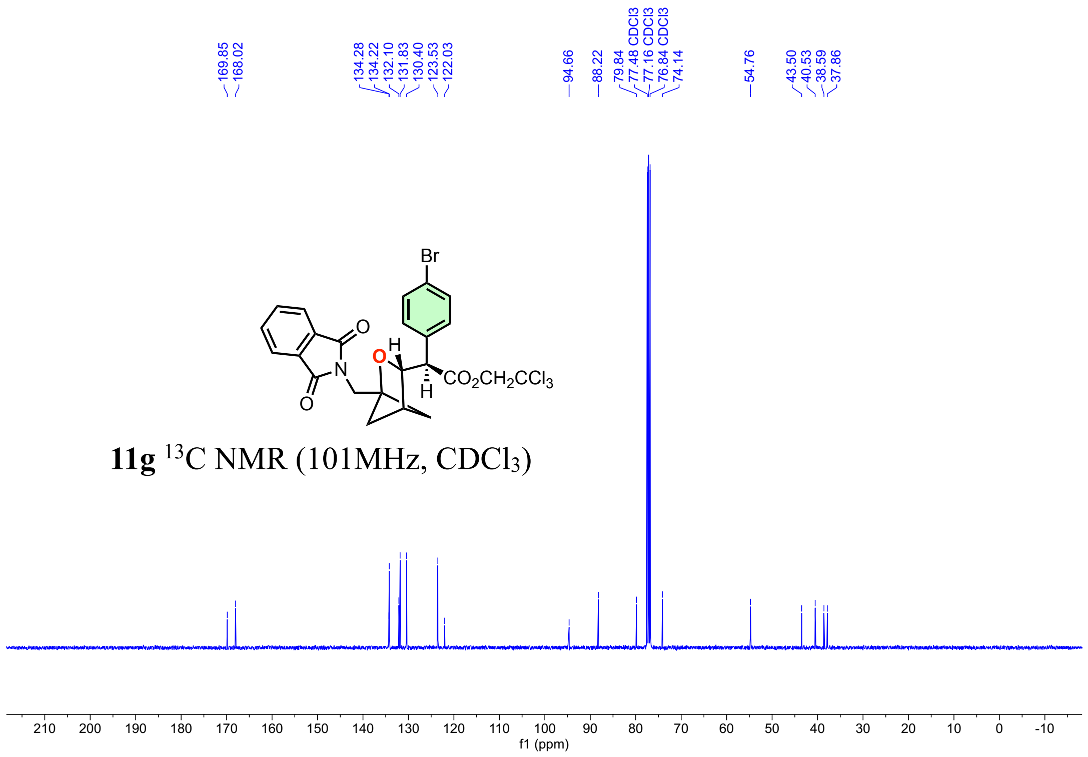

11g ^{13}C NMR (101MHz, $CDCl_3$)

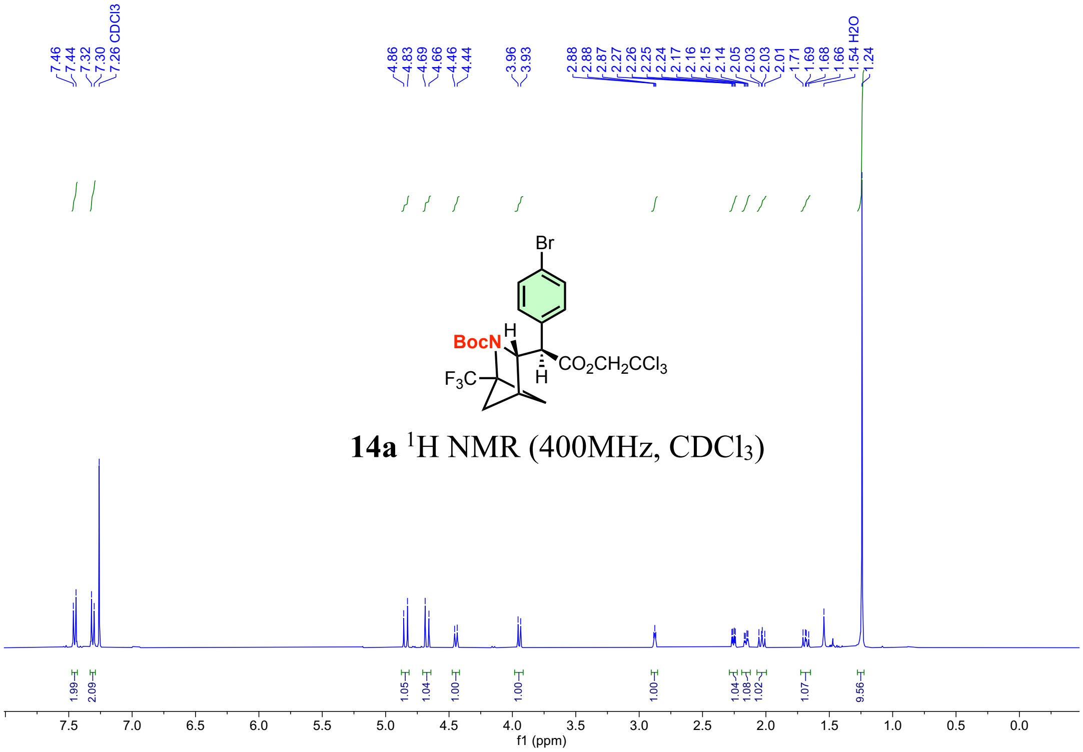

7.46
7.44
7.32
7.30
7.26 CDCl3
4.86
4.83
4.69
4.66
4.46
4.44
3.96
3.93
2.88
2.88
2.87
2.27
2.26
2.25
2.24
2.17
2.16
2.15
2.14
2.05
2.03
2.03
2.01
1.71
1.69
1.68
1.66
1.54 H2O
1.24
Br
BocN
H
CO2CH2CCl3
F3C
H
14a 1H NMR (400MHz, CDCl3)
1.99
2.09
1.05
1.04
1.00
1.00
1.00
1.04
1.08
1.02
1.07
9.56
f1 (ppm)

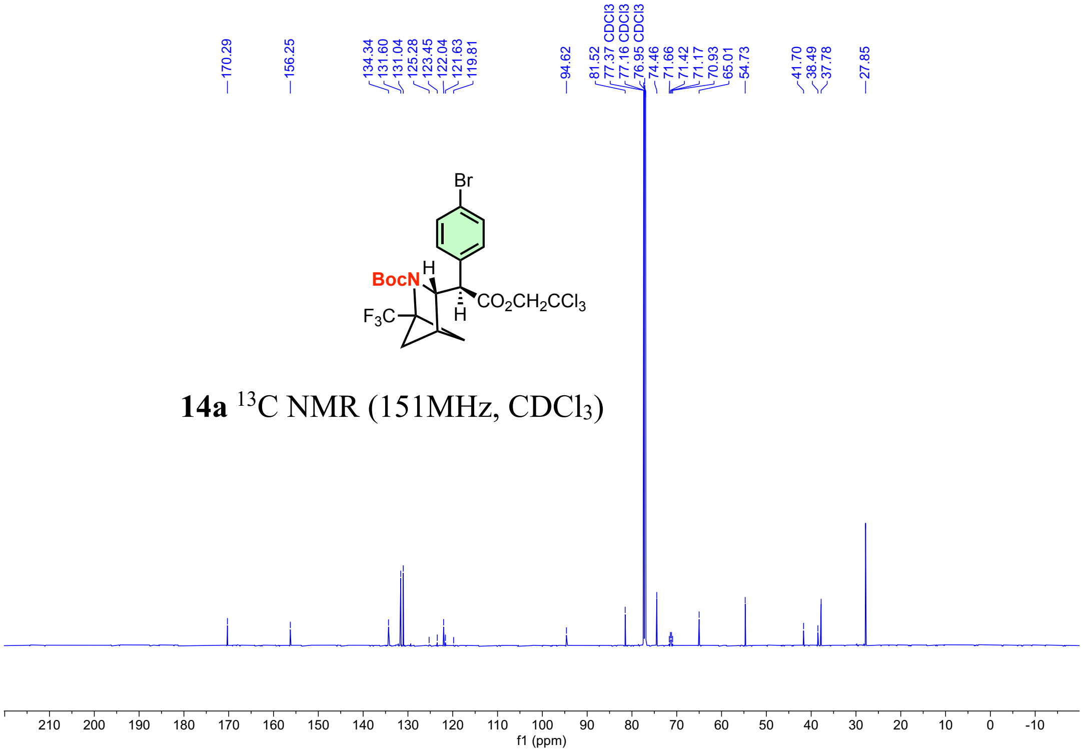

170.29
156.25
134.34
131.60
131.04
125.28
123.45
122.04
121.63
119.81
94.62
81.52
77.37 CDCl3
77.16 CDCl3
76.95 CDCl3
74.46
71.66
71.42
71.17
70.93
65.01
54.73
41.70
38.49
37.78
27.85
Br
BocN
H
CO2CH2CCl3
F3C
H
14a 13C NMR (151MHz, CDCl3)
f1 (ppm)

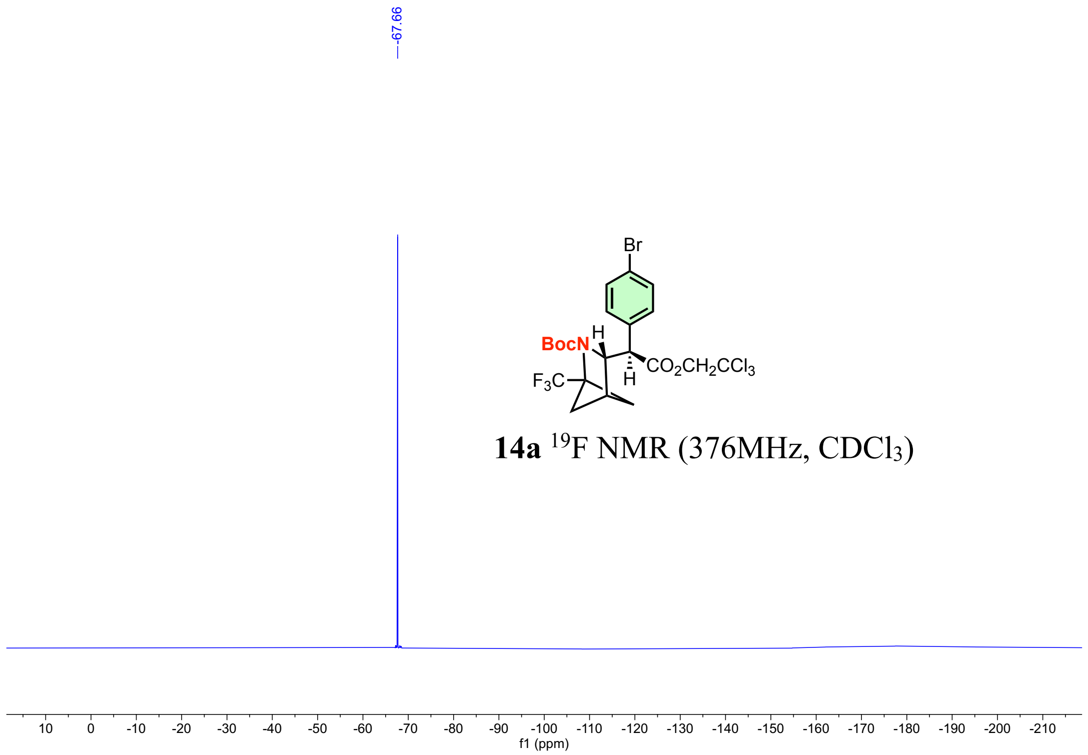
-67.66
Br
BocN
H
F3C
H
CO2CH2CCl3
14a 19F NMR (376MHz, CDCl3)
10 0 -10 -20 -30 -40 -50 -60 -70 -80 -90 -100 -110 -120 -130 -140 -150 -160 -170 -180 -190 -200 -210
f1 (ppm)

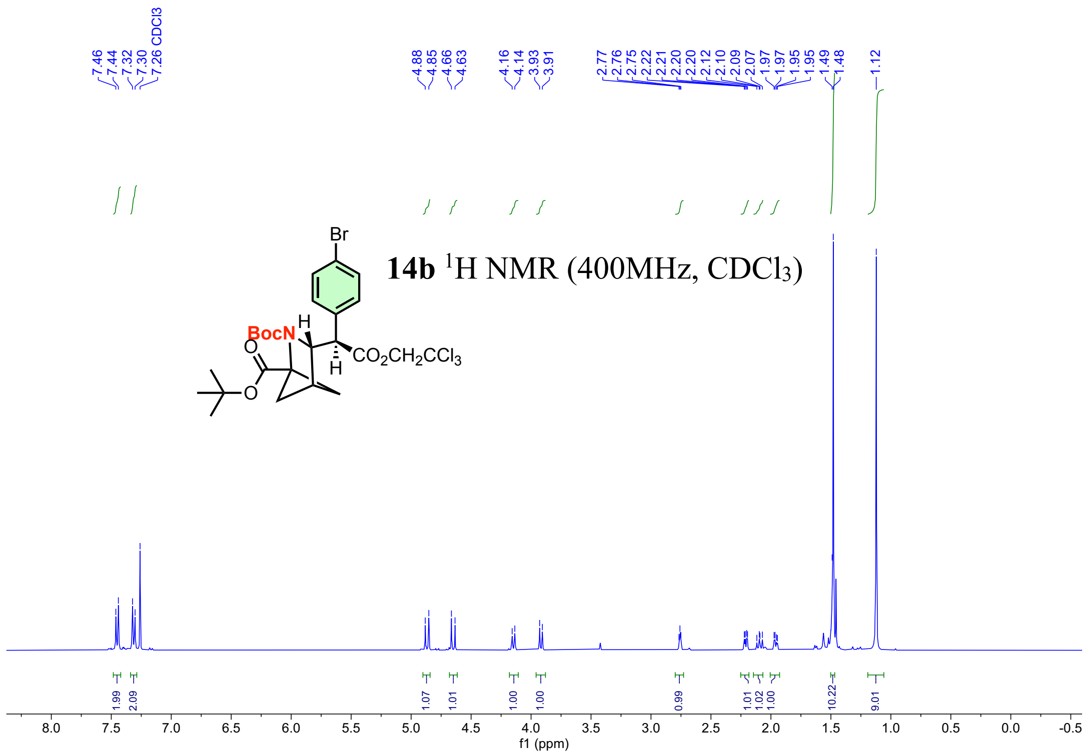
7.46 7.44 7.32 7.30 7.26 CDCl3
4.88 4.85 4.66 4.63
4.16 4.14 3.93 3.91
2.77 2.76 2.75 2.22 2.21 2.20 2.20 2.12 2.10 2.09 2.07 1.97 1.97 1.95 1.95 1.49 1.48
1.12
14b 1H NMR (400MHz, CDCl3)
Br
BocN
H
H
CO2CH2CCl3
O
O
1.99 2.09 1.07 1.01 1.00 1.00 0.99 1.01 1.02 1.00 10.22 9.01
8.0 7.5 7.0 6.5 6.0 5.5 5.0 4.5 4.0 3.5 3.0 2.5 2.0 1.5 1.0 0.5 0.0 -0.5
f1 (ppm)

14b ^{13}C NMR (101MHz, $CDCl_3$)

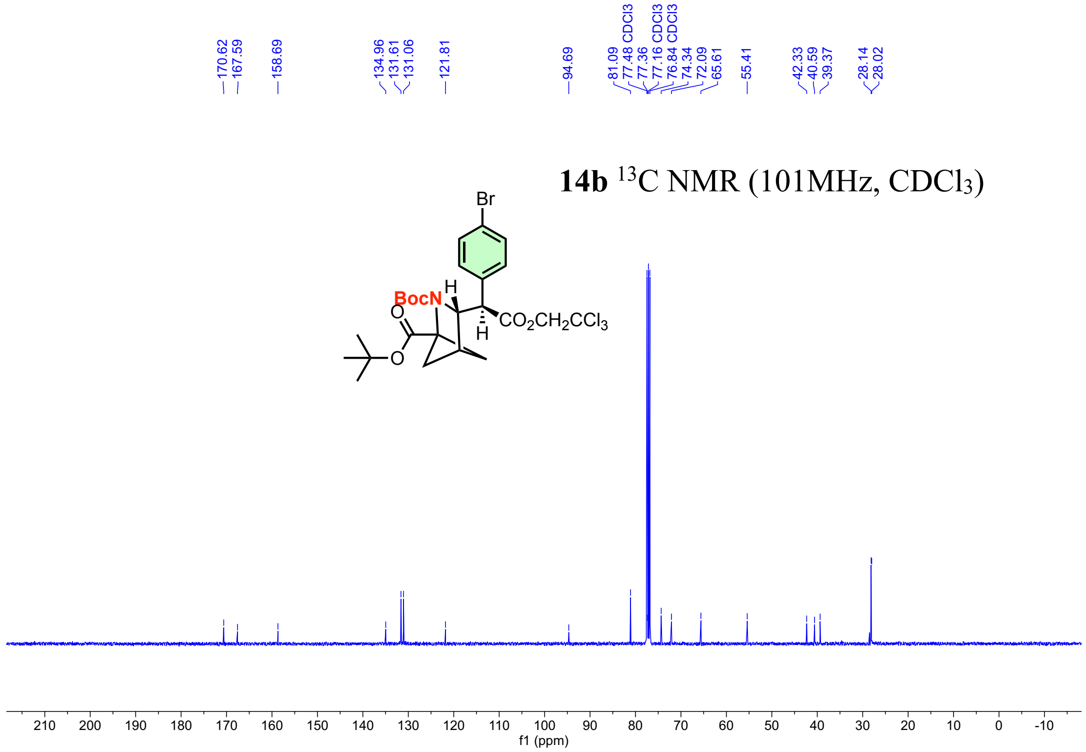

6. HPLC and SFC Chromatograms

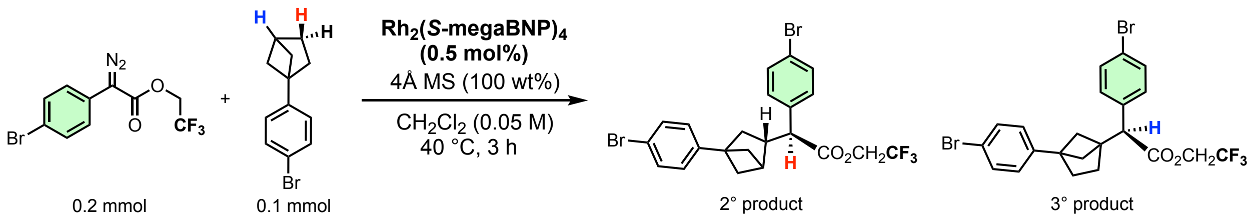

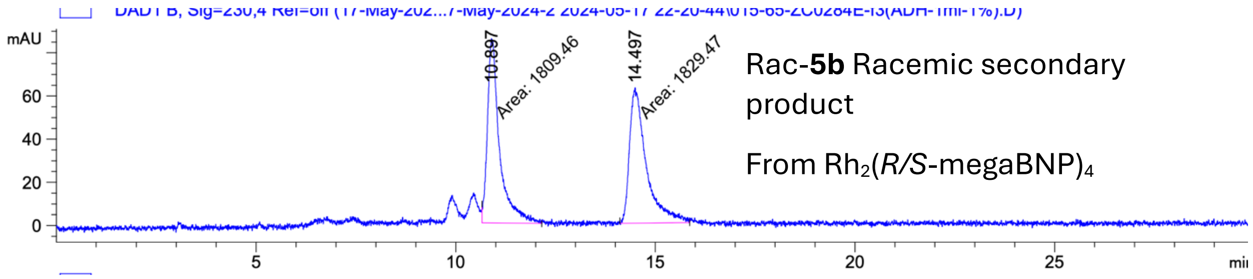

Signal 2: DAD1 B, Sig=230,4 Ref=off

Peak #	RetTime [min]	Type	Width [min]	Area [mAU*s]	Height [mAU]	Area %
1	10.897	MM	0.3532	1809.46191	85.37358	49.7251
2	14.497	MM	0.4857	1829.46838	62.77546	50.2749
Totals :				3638.93030	148.14904	

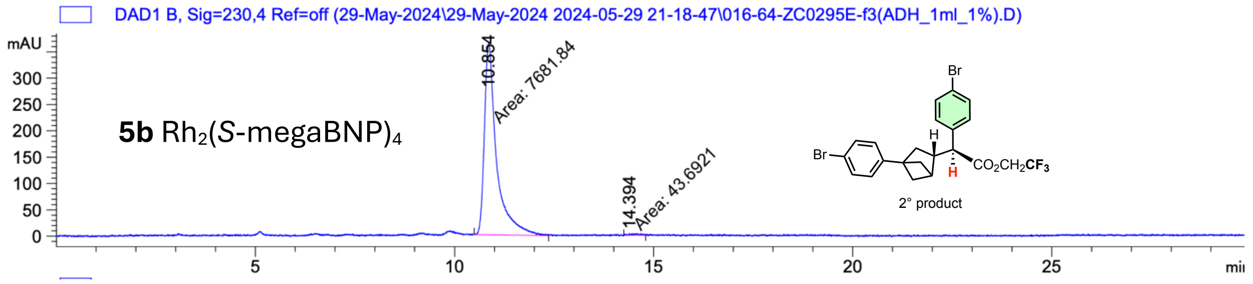

Signal 2: DAD1 B, Sig=230,4 Ref=off

Peak #	RetTime [min]	Type	Width [min]	Area [mAU*s]	Height [mAU]	Area %
1	10.854	MM	0.3522	7681.83643	363.47470	99.4344
2	14.394	MM	0.2310	43.69215	3.15232	0.5656
Totals :				7725.52857	366.62703	

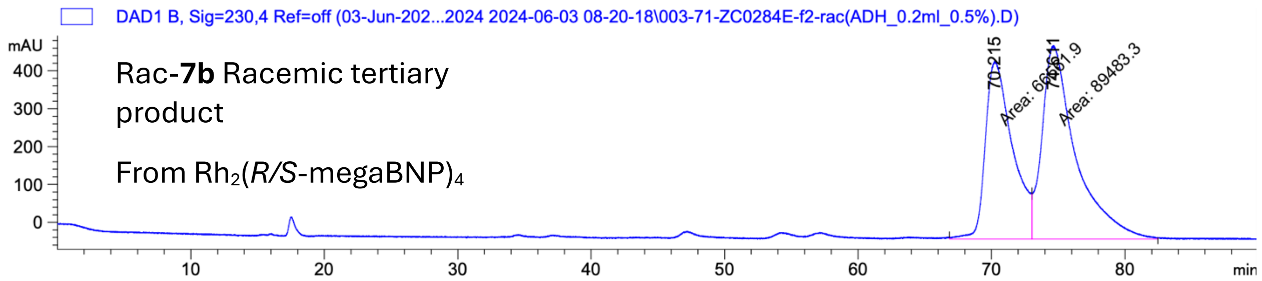

Signal 2: DAD1 B, Sig=230,4 Ref=off

Peak #	RetTime [min]	Type	Width [min]	Area [mAU*s]	Height [mAU]	Area %
1	70.215	MF	2.3612	6.65619e4	469.83527	42.6555
2	74.611	FM	2.9198	8.94833e4	510.79019	57.3445
Totals :				1.56045e5	980.62546	

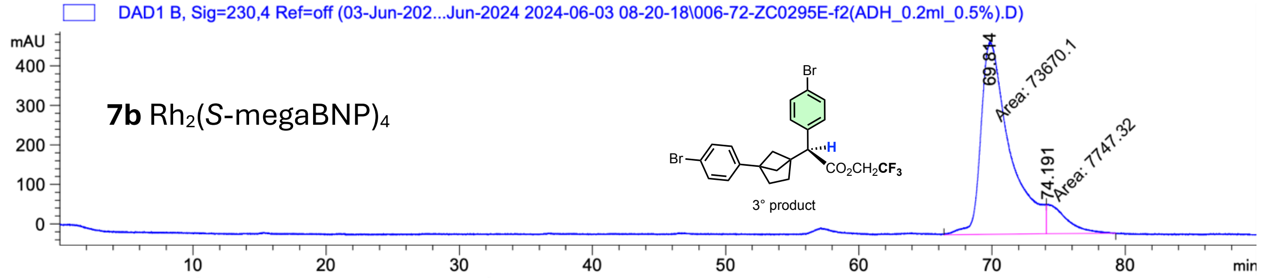

Signal 2: DAD1 B, Sig=230,4 Ref=off

Peak #	RetTime [min]	Type	Width [min]	Area [mAU*s]	Height [mAU]	Area %
1	69.814	MF	2.5134	7.36701e4	488.51749	90.4844
2	74.191	FM	1.7247	7747.31934	74.86497	9.5156
Totals :				8.14174e4	563.38245	

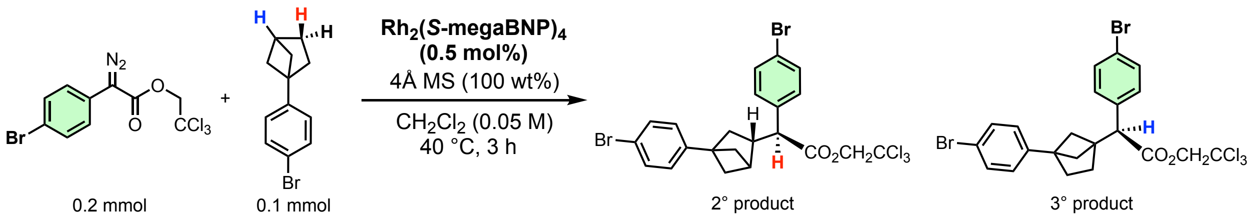

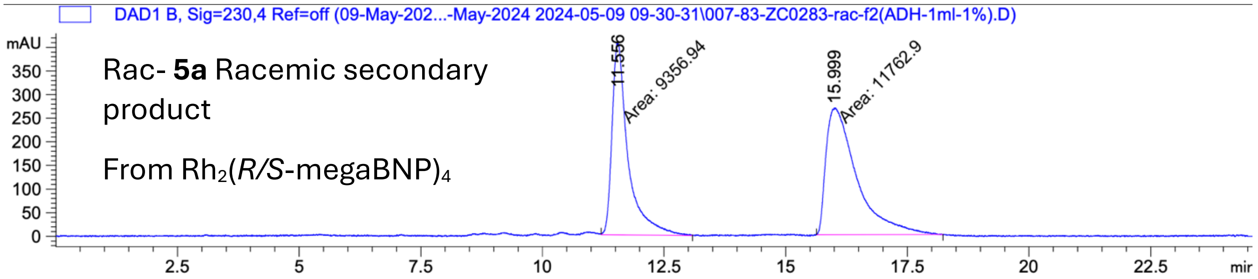

Signal 2: DAD1 B, Sig=230,4 Ref=off

Peak #	RetTime [min]	Type	Width [min]	Area [mAU*s]	Height [mAU]	Area %
1	11.556	MM	0.3842	9356.93652	405.95023	44.3041
2	15.999	MM	0.7298	1.17629e4	268.63205	55.6959

Totals : 2.11198e4 674.58228

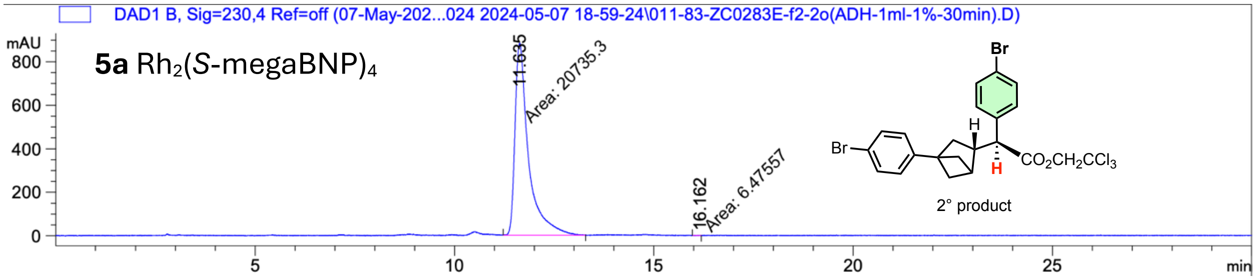

Signal 2: DAD1 B, Sig=230,4 Ref=off

Peak #	RetTime [min]	Type	Width [min]	Area [mAU*s]	Height [mAU]	Area %
1	11.635	MM	0.3912	2.07353e4	883.30011	99.9688
2	16.162	MM	0.0610	6.47557	1.76850	0.0312

Totals : 2.07418e4 885.06861

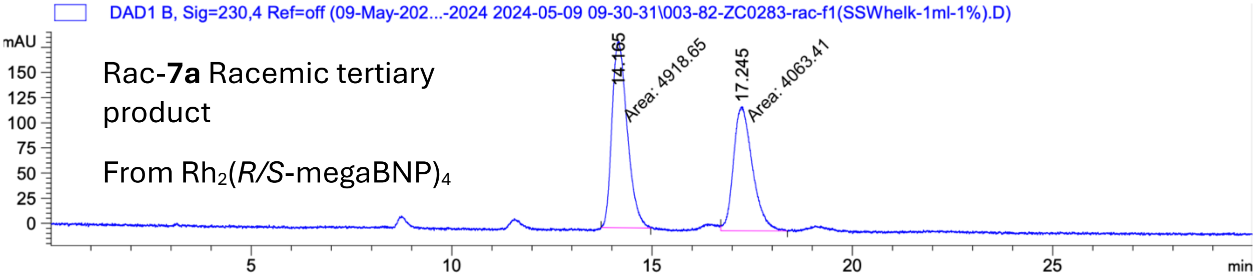

Signal 2: DAD1 B, Sig=230,4 Ref=off

Peak #	RetTime [min]	Type	Width [min]	Area [mAU*s]	Height [mAU]	Area %
1	14.165	MM	0.4429	4918.65039	185.10672	54.7608
2	17.245	MM	0.5485	4063.40674	123.47163	45.2392
Totals :				8982.05713	308.57835	

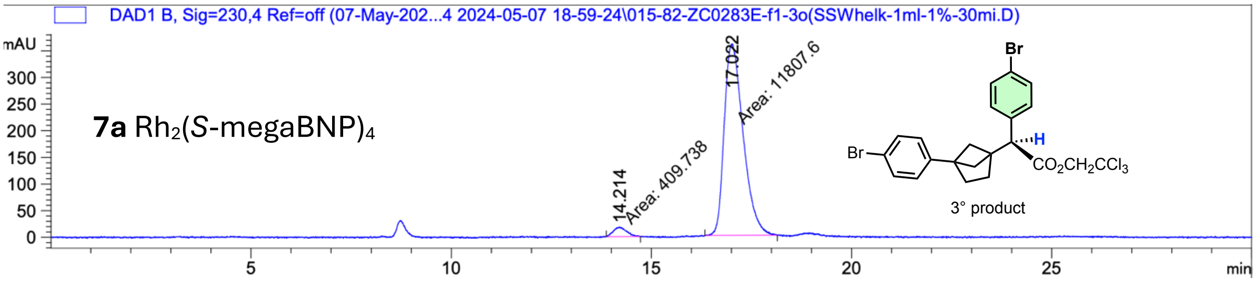

Signal 2: DAD1 B, Sig=230,4 Ref=off

Peak #	RetTime [min]	Type	Width [min]	Area [mAU*s]	Height [mAU]	Area %
1	14.214	MM	0.3805	409.73819	17.94862	3.3537
2	17.022	MM	0.5472	1.18076e4	359.62250	96.6463
Totals :				1.22173e4	377.57111	

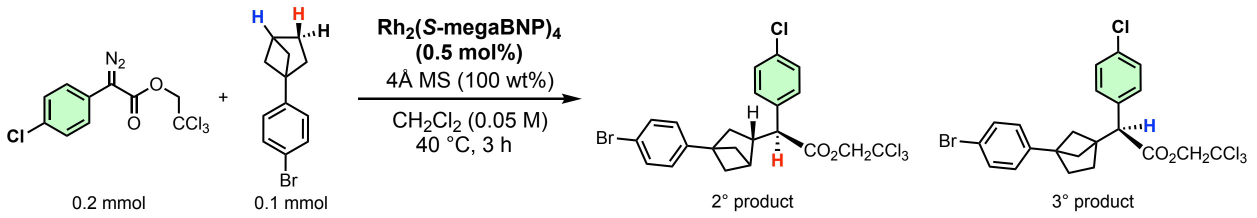

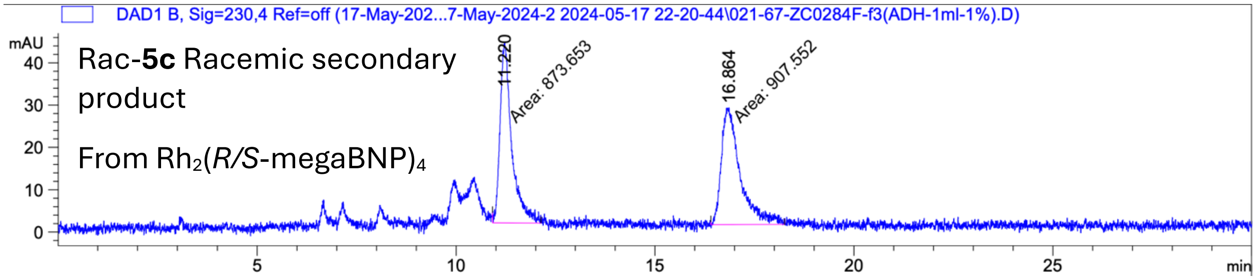

Signal 2: DAD1 B, Sig=230,4 Ref=off

Peak #	RetTime [min]	Type	Width [min]	Area [mAU*s]	Height [mAU]	Area %
1	11.220	MM	0.3426	873.65314	42.50075	49.0484
2	16.864	MM	0.5470	907.55200	27.65072	50.9516

Totals : 1781.20514 70.15147

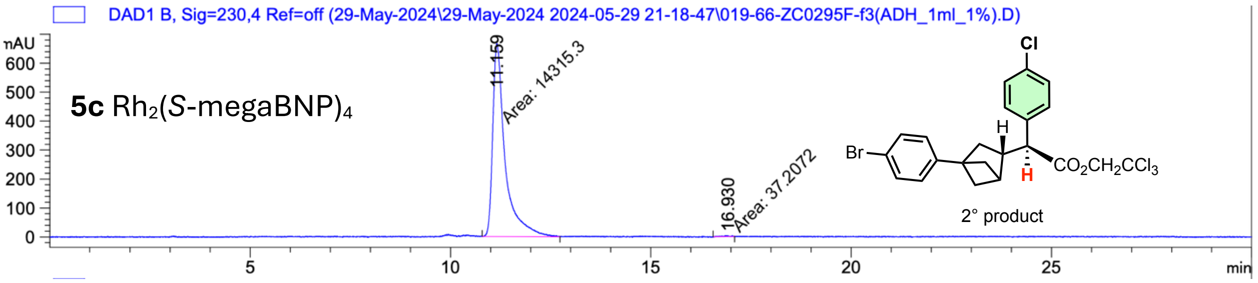

Signal 2: DAD1 B, Sig=230,4 Ref=off

Peak #	RetTime [min]	Type	Width [min]	Area [mAU*s]	Height [mAU]	Area %
1	11.159	MM	0.3578	1.43153e4	666.82635	99.7408
2	16.930	MM	0.1846	37.20716	3.35992	0.2592

Totals : 1.43525e4 670.18627

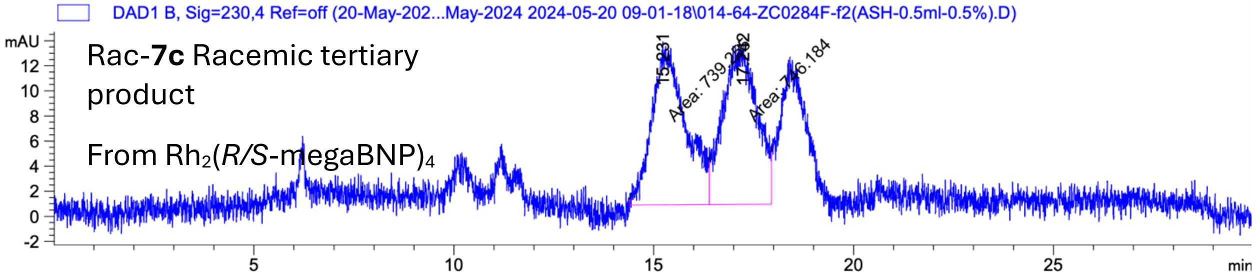

Signal 2: DAD1 B, Sig=230,4 Ref=off

Peak #	RetTime [min]	Type	Width [min]	Area [mAU*s]	Height [mAU]	Area %
1	15.231	MF	0.9445	739.25470	13.04508	49.7667
2	17.242	FM	0.9651	746.18439	12.88614	50.2333
Totals :				1485.43909	25.93122	

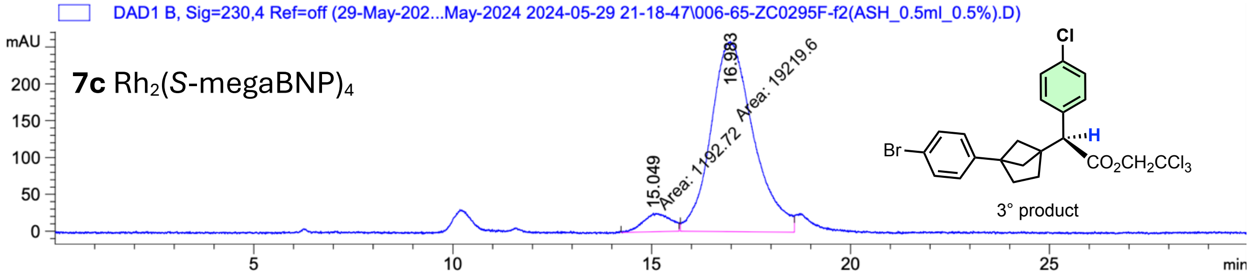

Signal 2: DAD1 B, Sig=230,4 Ref=off

Peak #	RetTime [min]	Type	Width [min]	Area [mAU*s]	Height [mAU]	Area %
1	15.049	MM	0.7763	1192.71606	25.60528	5.8431
2	16.983	MM	1.2398	1.92196e4	258.37076	94.1569
Totals :				2.04123e4	283.97604	

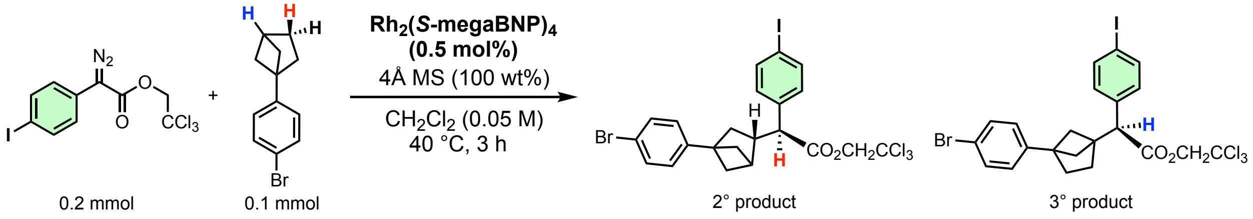

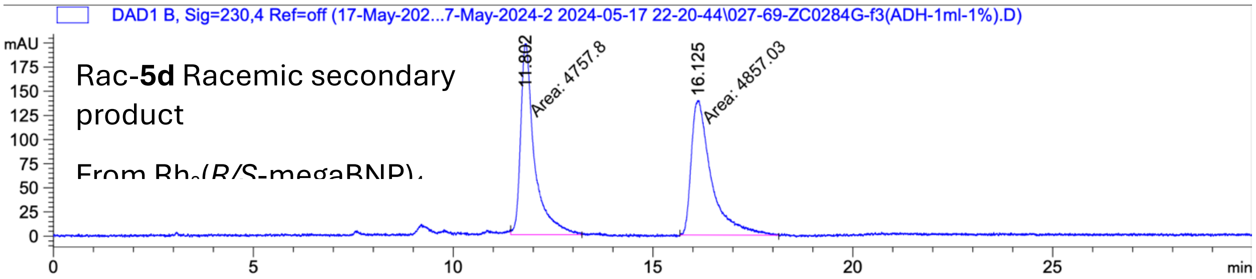

Signal 2: DAD1 B, Sig=230,4 Ref=off

Peak #	RetTime [min]	Type	Width [min]	Area [mAU*s]	Height [mAU]	Area %
1	11.802	MM	0.4013	4757.80225	197.60693	49.4840
2	16.125	MM	0.5814	4857.02637	139.22923	50.5160
Totals :				9614.82861	336.83617	

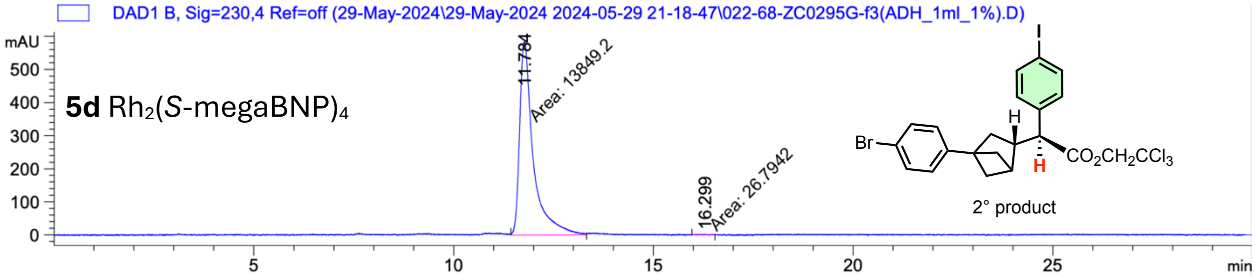

Signal 2: DAD1 B, Sig=230,4 Ref=off

Peak #	RetTime [min]	Type	Width [min]	Area [mAU*s]	Height [mAU]	Area %
1	11.784	MM	0.3954	1.38492e4	583.80969	99.8069
2	16.299	MM	0.1899	26.79424	2.35190	0.1931
Totals :				1.38760e4	586.16159	

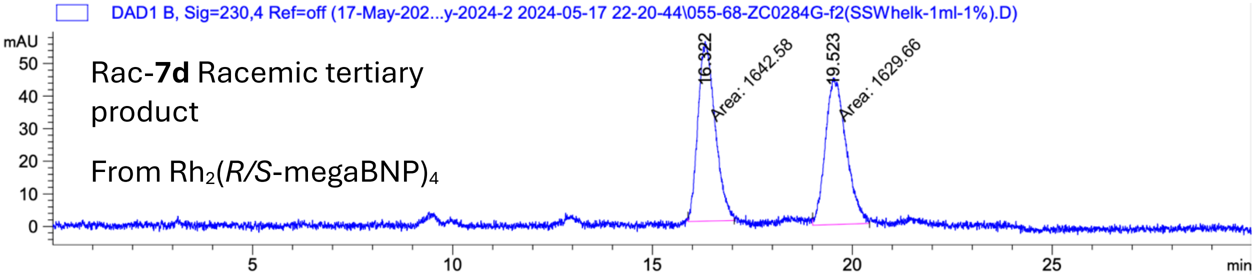


```
Signal 2: DAD1 B, Sig=230,4 Ref=off

Peak RetTime Type  Width     Area       Height      Area
  #   [min]        [min]   [mAU*s]      [mAU]        %
----|-------|----|-------|----------|----------|--------|
   1  16.322 MM     0.4964 1642.58179   55.15130  50.1974
   2  19.523 MM     0.6034 1629.65991   45.01616  49.8026

Totals :                   3272.24170  100.16746
```

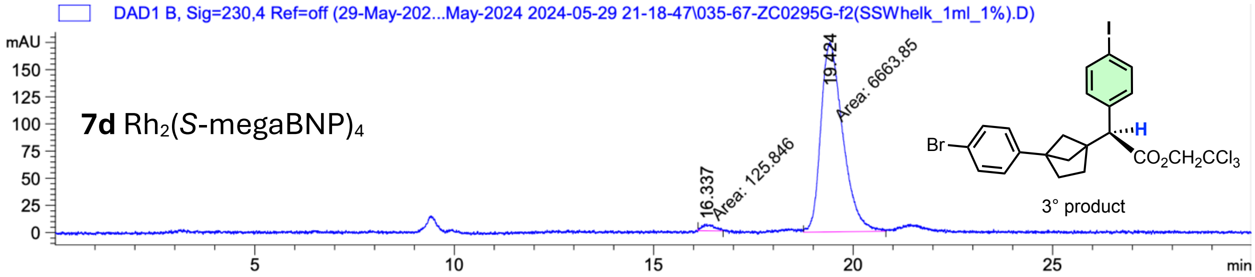


```
Signal 2: DAD1 B, Sig=230,4 Ref=off

Peak RetTime Type  Width     Area       Height      Area
  #   [min]        [min]   [mAU*s]      [mAU]        %
----|-------|----|-------|----------|----------|--------|
   1  16.337 MM     0.3311  125.84606    6.33453   1.8535
   2  19.424 MM     0.6349 6663.84863  174.92798  98.1465

Totals :                   6789.69469  181.26251
```

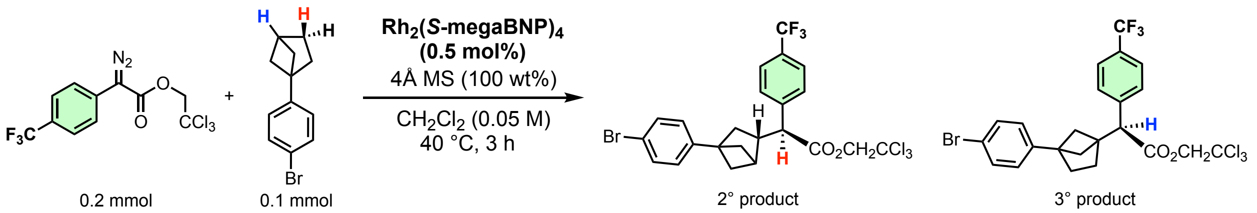

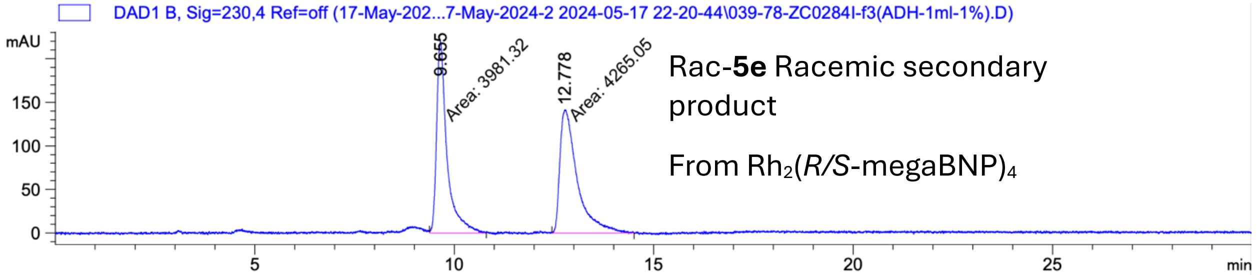

Signal 2: DAD1 B, Sig=230,4 Ref=off

Peak #	RetTime [min]	Type	Width [min]	Area [mAU*s]	Height [mAU]	Area %
1	9.655	MM	0.3021	3981.31812	219.66425	48.2796
2	12.778	MM	0.5025	4265.05322	141.46004	51.7204
Totals :				8246.37134	361.12428	

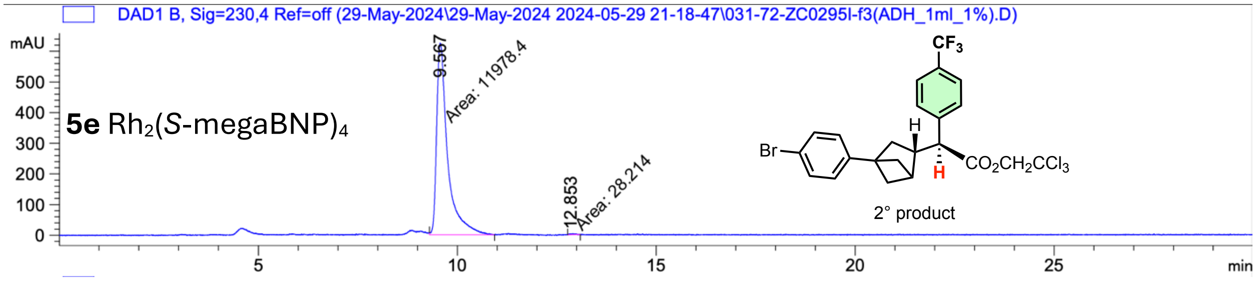

Signal 2: DAD1 B, Sig=230,4 Ref=off

Peak #	RetTime [min]	Type	Width [min]	Area [mAU*s]	Height [mAU]	Area %
1	9.567	MM	0.3190	1.19784e4	625.79089	99.7650
2	12.853	MM	0.1439	28.21398	3.26864	0.2350
Totals :				1.20066e4	629.05954	

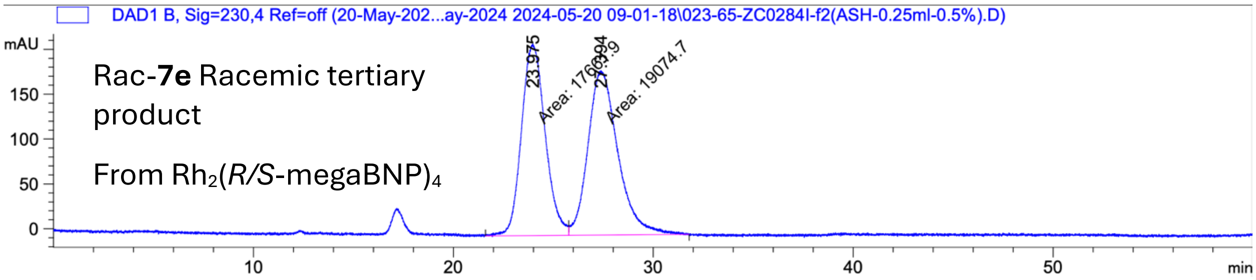

Signal 2: DAD1 B, Sig=230,4 Ref=off

Peak #	RetTime [min]	Type	Width [min]	Area [mAU*s]	Height [mAU]	Area %
1	23.975	MF	1.3779	1.76619e4	213.62686	48.0771
2	27.394	FM	1.7350	1.90747e4	183.22955	51.9229
Totals :				3.67367e4	396.85641	

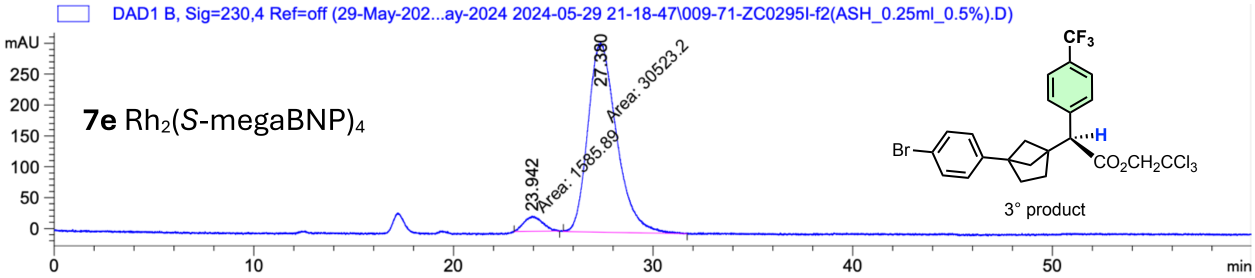

Signal 2: DAD1 B, Sig=230,4 Ref=off

Peak #	RetTime [min]	Type	Width [min]	Area [mAU*s]	Height [mAU]	Area %
1	23.942	MM	1.0579	1585.89404	24.98541	4.9391
2	27.380	MM	1.6567	3.05232e4	307.06601	95.0609
Totals :				3.21091e4	332.05142	

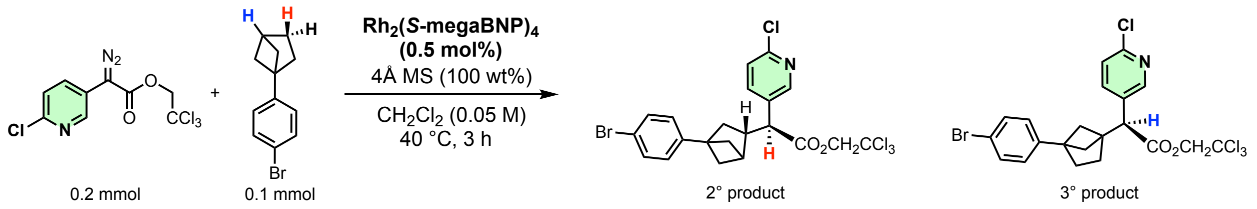

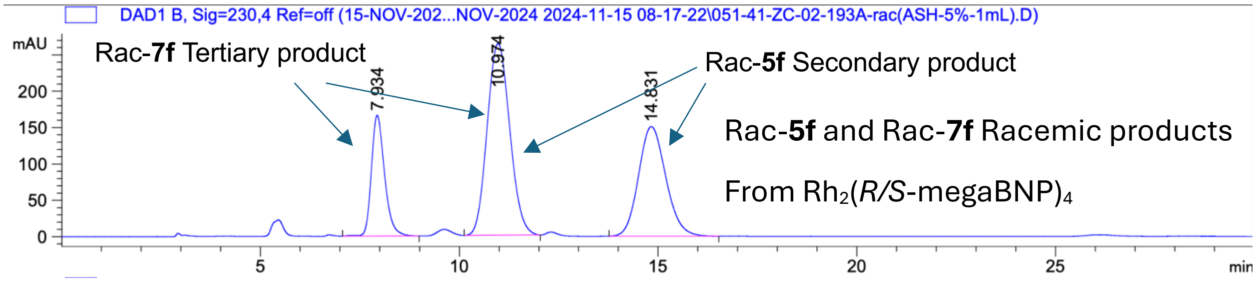

Signal 2: DAD1 B, Sig=230,4 Ref=off

Peak #	RetTime [min]	Type	Width [min]	Area [mAU*s]	Height [mAU]	Area %
1	7.934	BB	0.3419	3826.72925	166.31200	17.8662
2	10.974	BB	0.5551	1.04037e4	263.53311	48.5728
3	14.831	BB	0.5605	7188.34619	150.70573	33.5610

Totals : 2.14188e4 580.55084

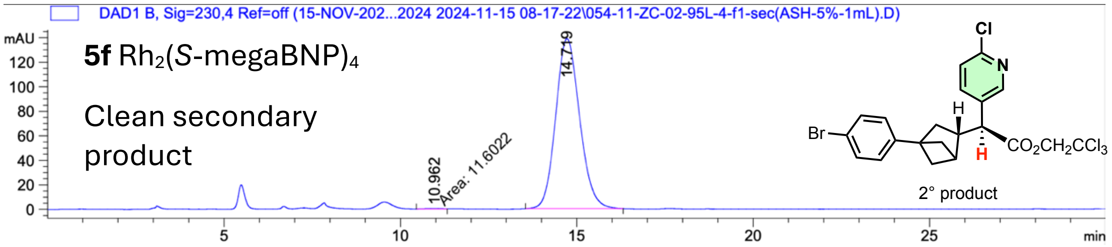

Signal 2: DAD1 B, Sig=230,4 Ref=off

Peak #	RetTime [min]	Type	Width [min]	Area [mAU*s]	Height [mAU]	Area %
1	10.962	MM	0.5423	11.60215	3.56578e-1	0.1774
2	14.719	BB	0.5538	6526.78955	138.63266	99.8226

Totals : 6538.39171 138.98924

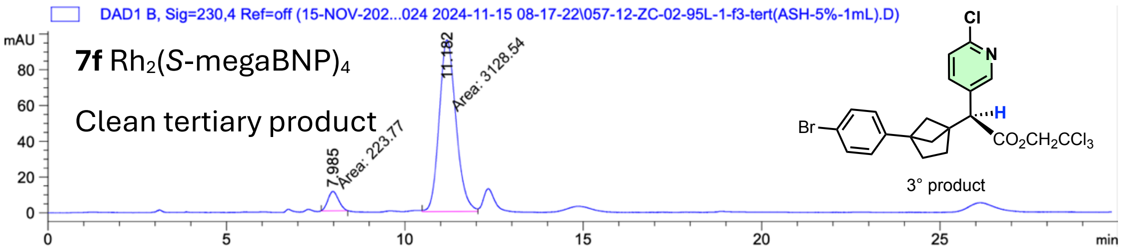

Signal 2: DAD1 B, Sig=230,4 Ref=off

Peak #	RetTime [min]	Type	Width [min]	Area [mAU*s]	Height [mAU]	Area %
1	7.985	MM	0.3455	223.77039	10.79486	6.6751
2	11.182	MM	0.5430	3128.54102	96.02675	93.3249

Totals : 3352.31140 106.82161

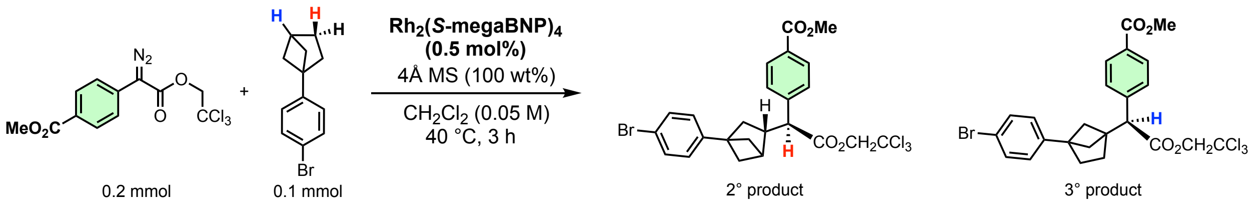
0.2 mmol
0.1 mmol
Rh2(S-megaBNP)4
(0.5 mol%)
4Å MS (100 wt%)
CH2Cl2 (0.05 M)
40 °C, 3 h
2° product
3° product

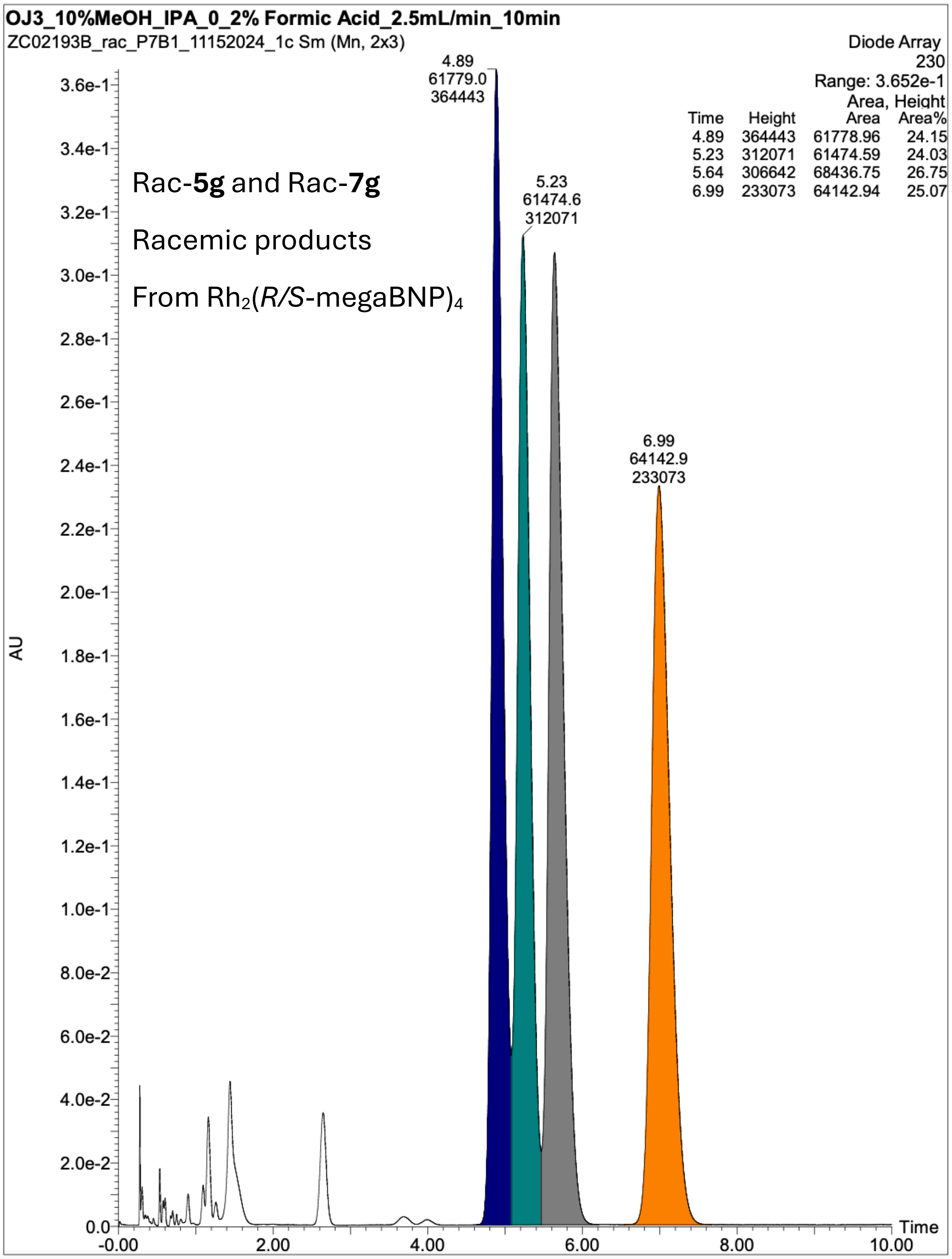
OJ3_10%MeOH_IPA_0_2% Formic Acid_2.5mL/min_10min
ZC02193B_rac_P7B1_11152024_1c Sm (Mn, 2x3)
Diode Array
230
Range: 3.652e-1
Area, Height
Time Height Area Area%
4.89 364443 61778.96 24.15
5.23 312071 61474.59 24.03
5.64 306642 68436.75 26.75
6.99 233073 64142.94 25.07
Rac-5g and Rac-7g
Racemic products
From Rh2(R/S-megaBNP)4
AU
Time

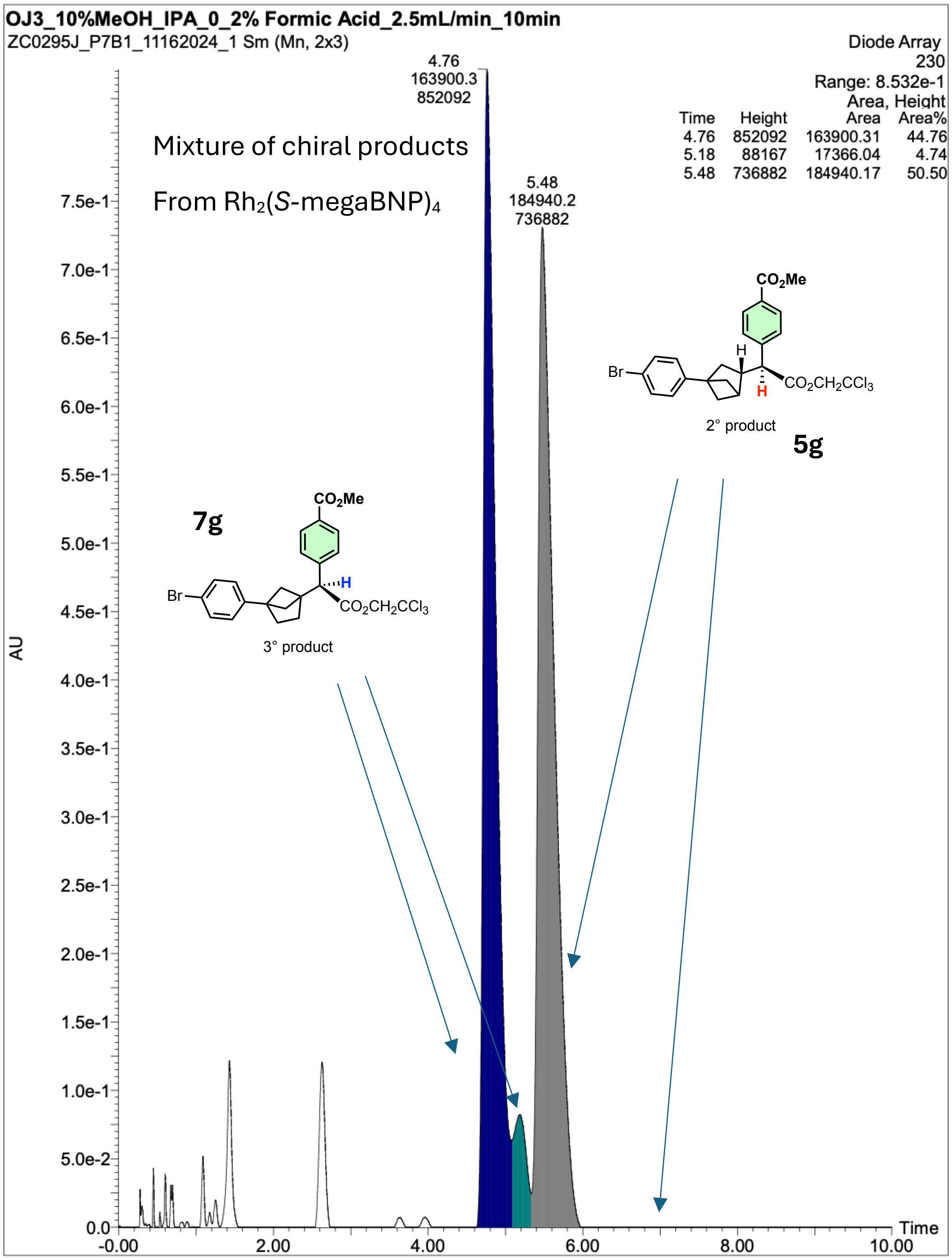
OJ3_10%MeOH_IPA_0_2% Formic Acid_2.5mL/min_10min
ZC0295J_P7B1_11162024_1 Sm (Mn, 2x3)
Diode Array
230
Range: 8.532e-1
Area, Height
Time Height Area Area%
4.76 852092 163900.31 44.76
5.18 88167 17366.04 4.74
5.48 736882 184940.17 50.50
4.76
163900.3
852092
5.48
184940.2
736882
Mixture of chiral products
From Rh2(S-megaBNP)4
7g
3° product
5g
2° product
AU
Time

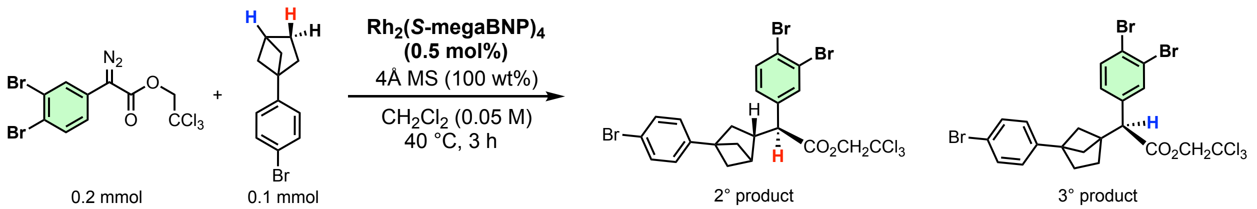

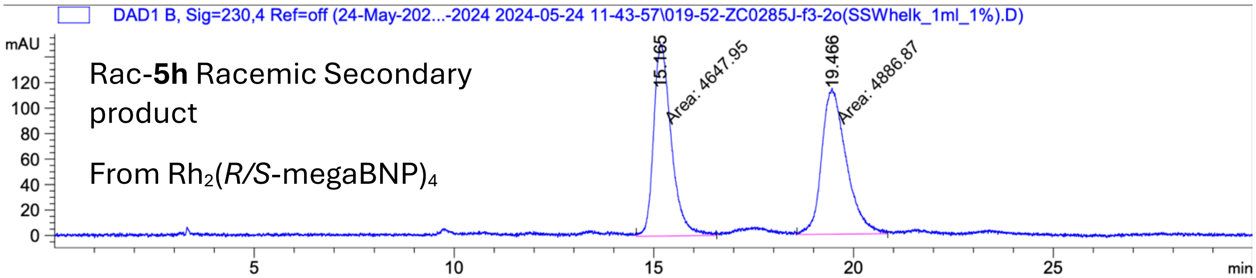

Signal 2: DAD1 B, Sig=230,4 Ref=off

Peak #	RetTime [min]	Type	Width [min]	Area [mAU*s]	Height [mAU]	Area %
1	15.165	MM	0.5139	4647.95313	150.75381	48.7472
2	19.466	MM	0.7116	4886.86670	114.46020	51.2528
Totals :				9534.81982	265.21401	

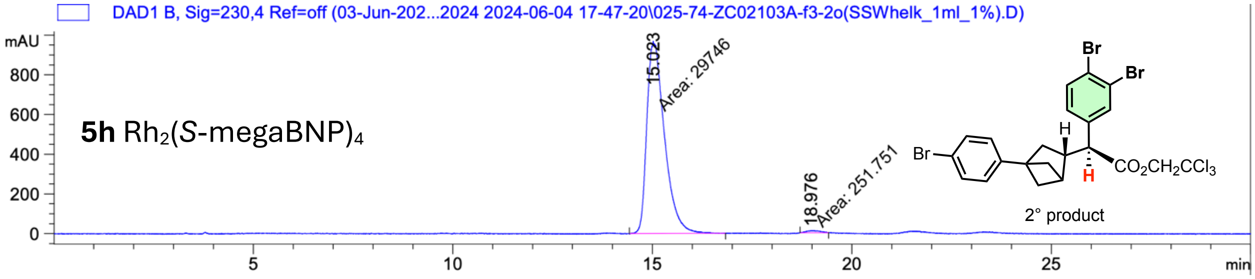

Signal 2: DAD1 B, Sig=230,4 Ref=off

Peak #	RetTime [min]	Type	Width [min]	Area [mAU*s]	Height [mAU]	Area %
1	15.023	MM	0.5117	2.97460e4	968.94055	99.1608
2	18.976	MM	0.3786	251.75082	11.08251	0.8392
Totals :				2.99977e4	980.02306	

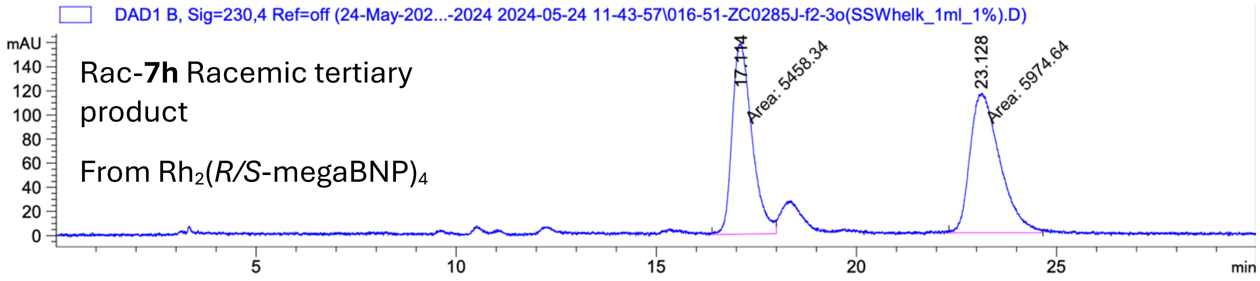

Signal 2: DAD1 B, Sig=230,4 Ref=off

Peak #	RetTime [min]	Type	Width [min]	Area [mAU*s]	Height [mAU]	Area %
1	17.114	MM	0.5756	5458.34180	158.05334	47.7421
2	23.128	MM	0.8609	5974.63672	115.67055	52.2579

Totals : 1.14330e4 273.72389

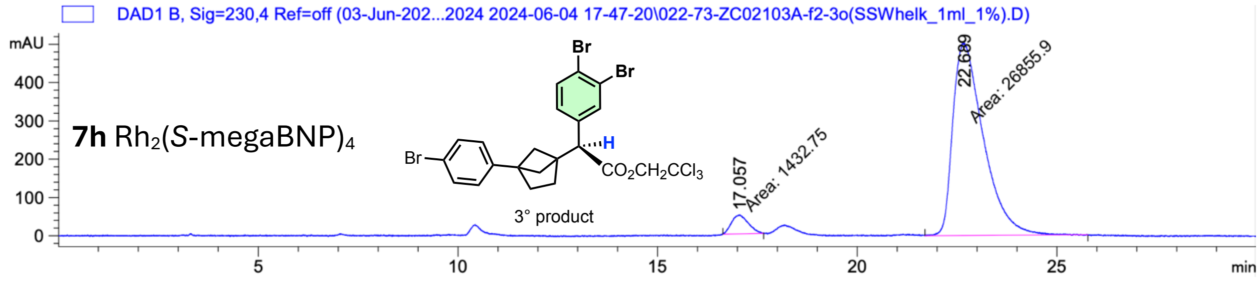

Signal 2: DAD1 B, Sig=230,4 Ref=off

Peak #	RetTime [min]	Type	Width [min]	Area [mAU*s]	Height [mAU]	Area %
1	17.057	MM	0.4802	1432.74939	49.72739	5.0648
2	22.689	MM	0.8905	2.68559e4	502.62122	94.9352

Totals : 2.82886e4 552.34861

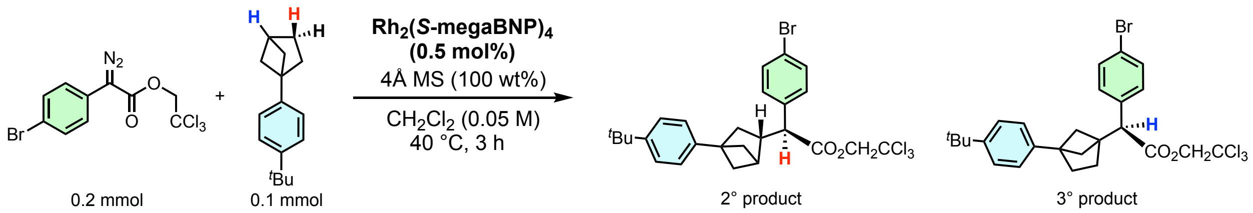

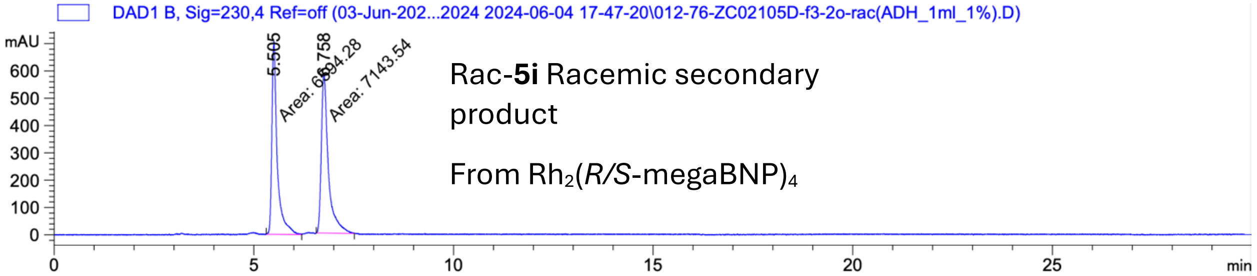

Signal 2: DAD1 B, Sig=230,4 Ref=off

Peak #	RetTime [min]	Type	Width [min]	Area [mAU*s]	Height [mAU]	Area %
1	5.505	MM	0.1557	6594.28467	705.83667	48.0009
2	6.758	MM	0.2035	7143.54199	584.97241	51.9991

Totals : 1.37378e4 1290.80908

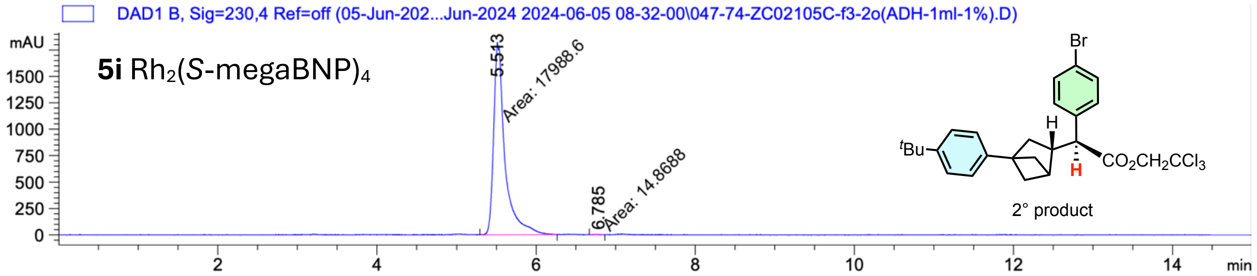

Signal 2: DAD1 B, Sig=230,4 Ref=off

Peak #	RetTime [min]	Type	Width [min]	Area [mAU*s]	Height [mAU]	Area %
1	5.513	MM	0.1645	1.79886e4	1823.09546	99.9174
2	6.785	MM	0.0804	14.86882	3.08144	0.0826

Totals : 1.80034e4 1826.17690

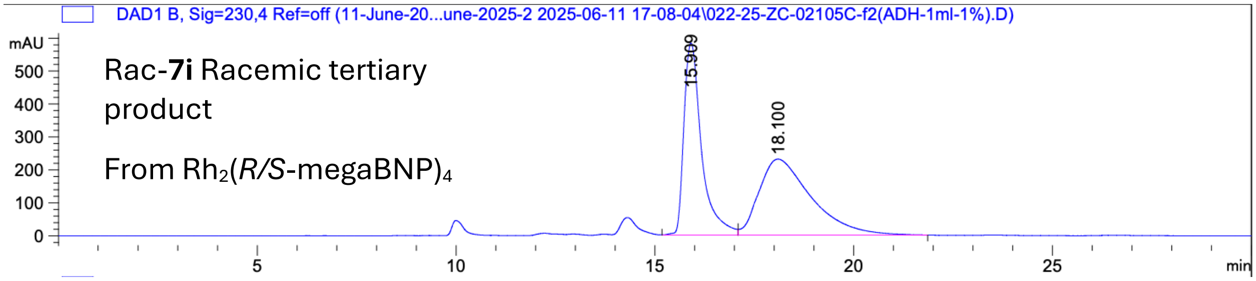

Signal 2: DAD1 B, Sig=230,4 Ref=off

Peak #	RetTime [min]	Type	Width [min]	Area [mAU*s]	Height [mAU]	Area %
1	15.909	BV	0.4594	1.79535e4	582.75940	46.1314
2	18.100	VV R	1.0656	2.09647e4	230.49498	53.8686

Totals : 3.89182e4 813.25438

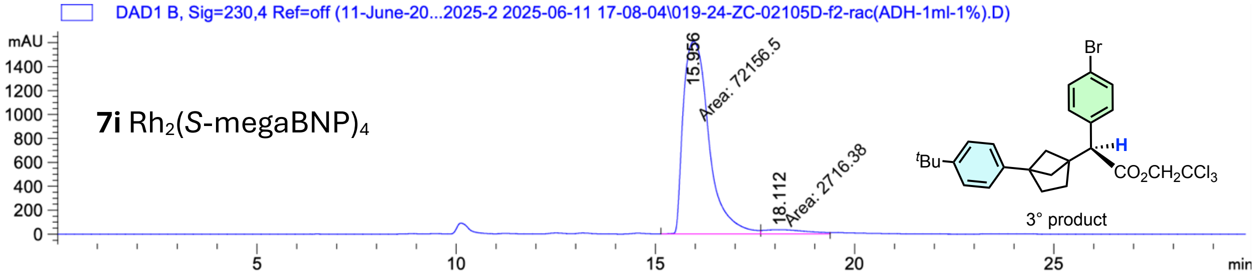

Signal 2: DAD1 B, Sig=230,4 Ref=off

Peak #	RetTime [min]	Type	Width [min]	Area [mAU*s]	Height [mAU]	Area %
1	15.956	MF	0.7469	7.21565e4	1610.16895	96.3720
2	18.112	MF	1.2469	2716.37646	36.30723	3.6280

Totals : 7.48728e4 1646.47617

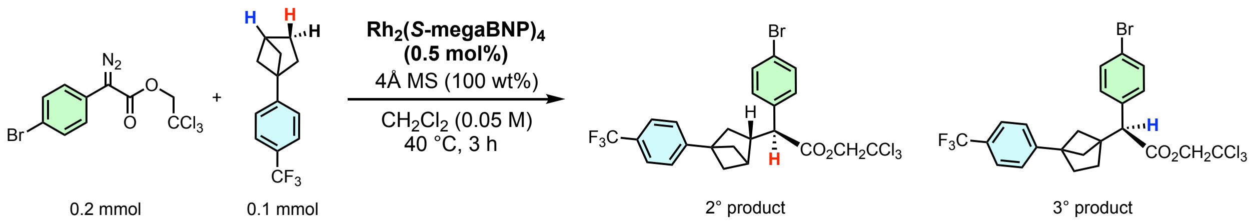

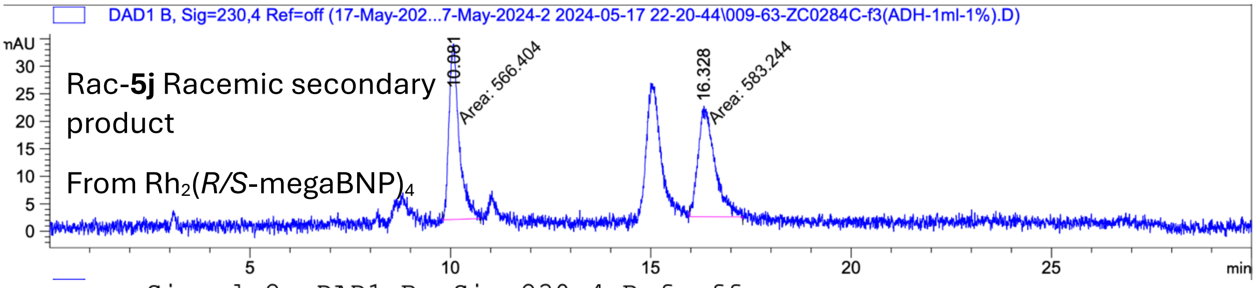

Signal 2: DAD1 B, Sig=230,4 Ref=off

Peak #	RetTime [min]	Type	Width [min]	Area [mAU*s]	Height [mAU]	Area %
1	10.081	MM	0.2957	566.40411	31.92060	49.2676
2	16.328	MM	0.4830	583.24390	20.12586	50.7324
Totals :				1149.64801	52.04646	

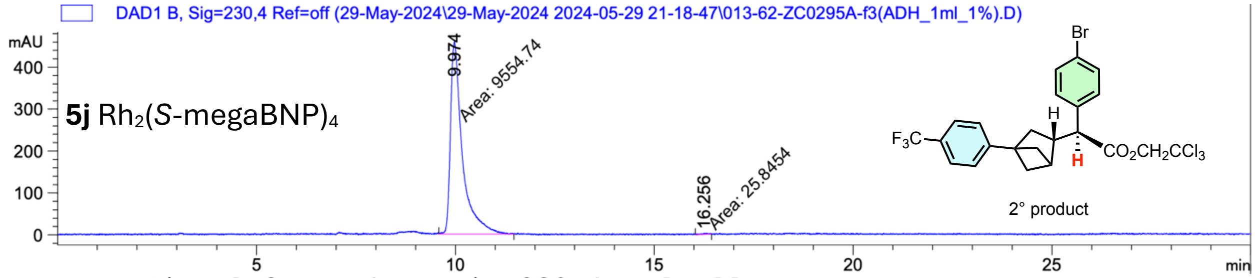

Signal 2: DAD1 B, Sig=230,4 Ref=off

Peak #	RetTime [min]	Type	Width [min]	Area [mAU*s]	Height [mAU]	Area %
1	9.974	MM	0.3472	9554.73926	458.71909	99.7302
2	16.256	MM	0.1561	25.84543	2.76019	0.2698
Totals :				9580.58469	461.47928	

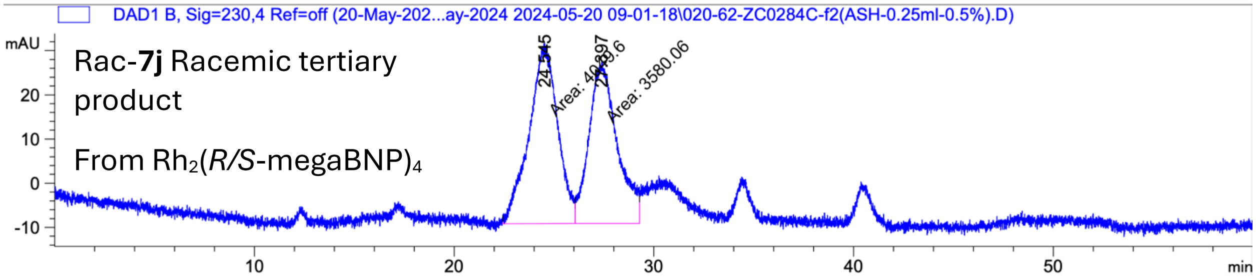

Signal 2: DAD1 B, Sig=230,4 Ref=off

Peak #	RetTime [min]	Type	Width [min]	Area [mAU*s]	Height [mAU]	Area %
1	24.545	MF	1.6550	4049.59839	40.78022	53.0770
2	27.397	MF	1.6294	3580.06226	36.61910	46.9230
Totals :				7629.66064	77.39933	

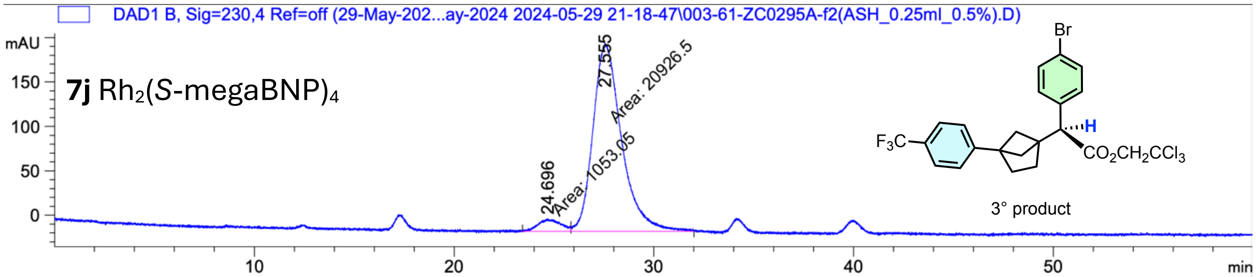

Signal 2: DAD1 B, Sig=230,4 Ref=off

Peak #	RetTime [min]	Type	Width [min]	Area [mAU*s]	Height [mAU]	Area %
1	24.696	MF	1.2615	1053.05493	13.91238	4.7911
2	27.555	FM	1.6478	2.09265e4	211.65862	95.2089
Totals :				2.19796e4	225.57099	

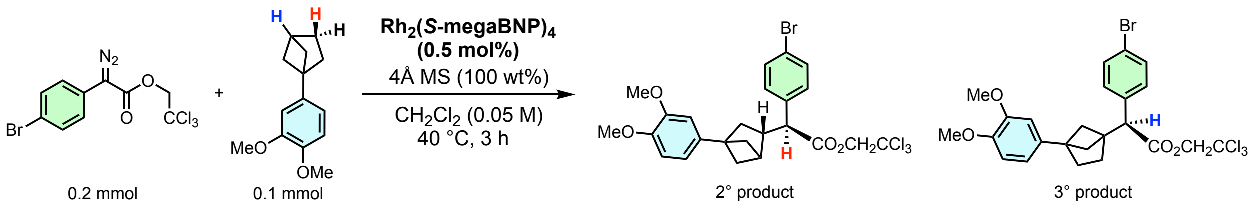

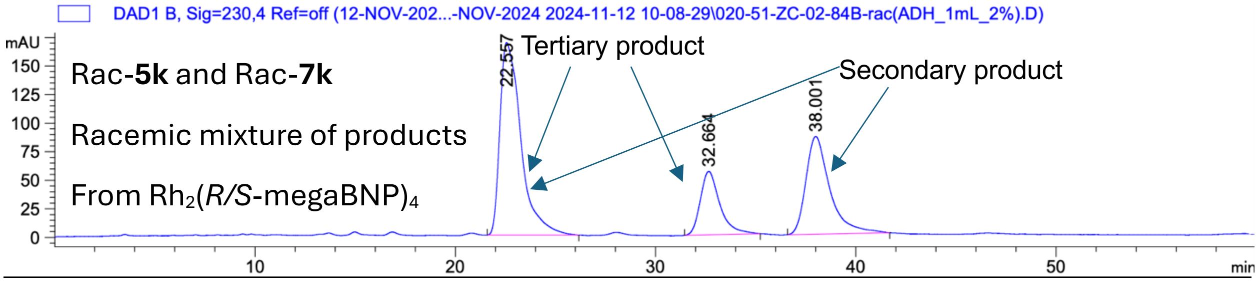

Signal 2: DAD1 B, Sig=230,4 Ref=off

Peak #	RetTime [min]	Type	Width [min]	Area [mAU*s]	Height [mAU]	Area %
1	22.557	BB	0.8564	1.22607e4	168.40852	53.0592
2	32.664	BB	0.7921	3752.59619	55.51903	16.2397
3	38.001	BB	0.9690	7094.31055	85.54368	30.7012

Totals : 2.31076e4 309.47123

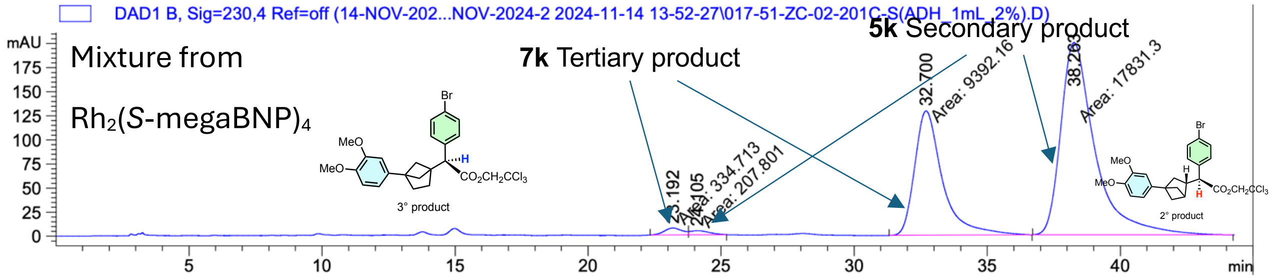

Signal 2: DAD1 B, Sig=230,4 Ref=off

Peak #	RetTime [min]	Type	Width [min]	Area [mAU*s]	Height [mAU]	Area %
1	23.192	MF	0.7791	334.71271	7.16015	1.2055
2	24.105	FM	0.8223	207.80136	4.21192	0.7484
3	32.700	MM	1.2122	9392.16016	129.13120	33.8262
4	38.263	MM	1.4885	1.78313e4	199.65520	64.2199

Totals : 2.77659e4 340.15846

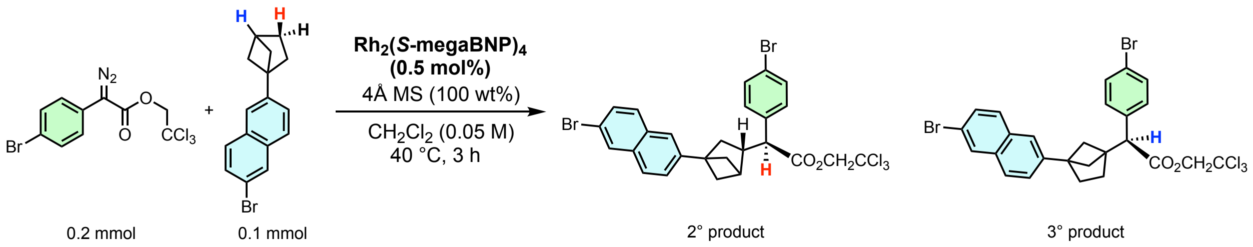

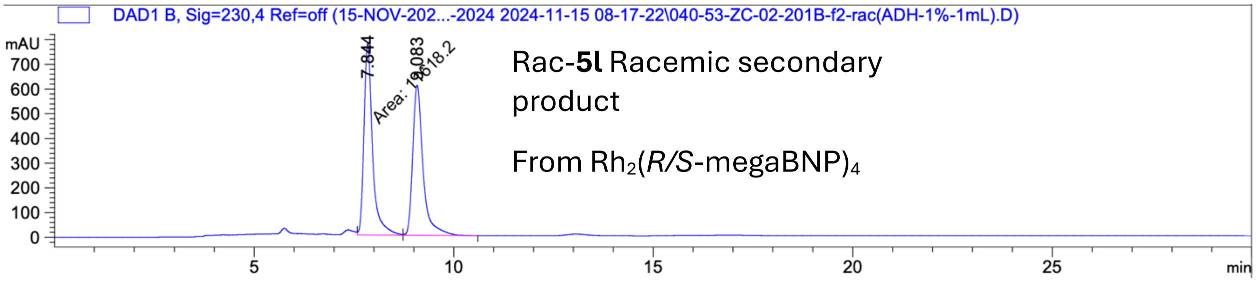

Signal 2: DAD1 B, Sig=230,4 Ref=off

Peak #	RetTime [min]	Type	Width [min]	Area [mAU*s]	Height [mAU]	Area %
1	7.844	FM	0.2504	1.16182e4	773.37592	51.3642
2	9.083	VB	0.2708	1.10011e4	607.34393	48.6358

Totals : 2.26193e4 1380.71985

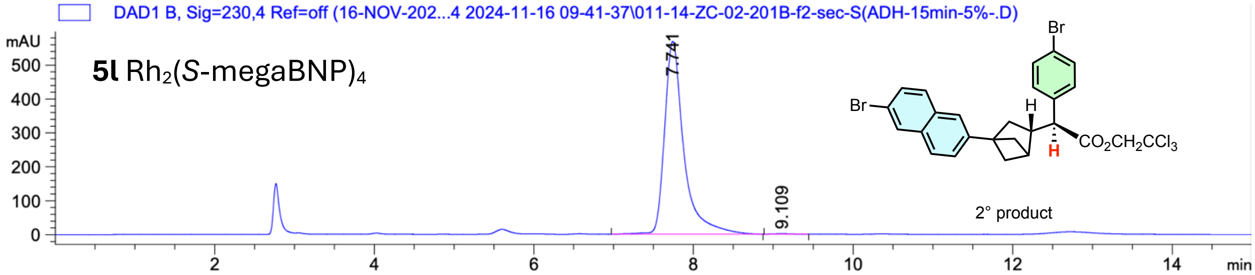

Signal 2: DAD1 B, Sig=230,4 Ref=off

Peak #	RetTime [min]	Type	Width [min]	Area [mAU*s]	Height [mAU]	Area %
1	7.741	BB	0.2514	9570.64746	568.60938	99.7902
2	9.109	BB	0.1705	20.12417	1.40164	0.2098

Totals : 9590.77163 570.01102

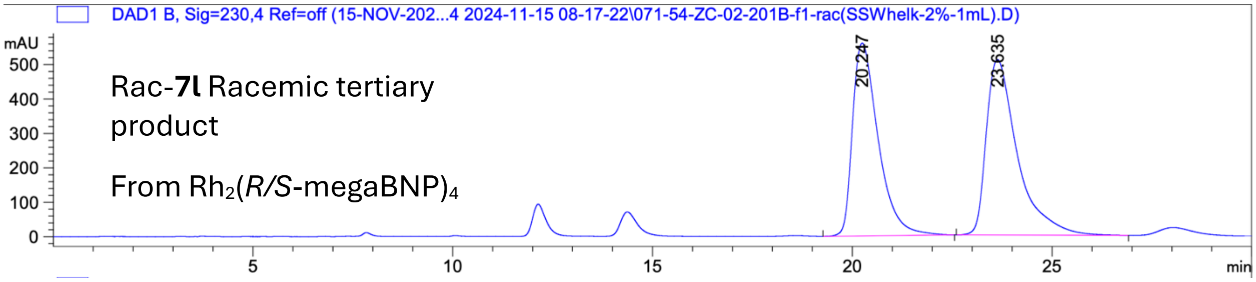

Signal 2: DAD1 B, Sig=230,4 Ref=off

Peak #	RetTime [min]	Type	Width [min]	Area [mAU*s]	Height [mAU]	Area %
1	20.247	BB	0.5767	2.50635e4	560.49200	47.5285
2	23.635	BB	0.6560	2.76701e4	510.34781	52.4715
Totals :				5.27336e4	1070.83981	

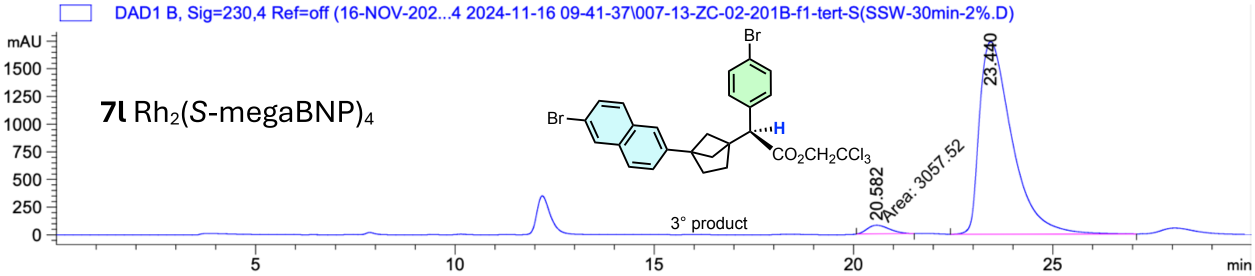

Signal 2: DAD1 B, Sig=230,4 Ref=off

Peak #	RetTime [min]	Type	Width [min]	Area [mAU*s]	Height [mAU]	Area %
1	20.582	MM	0.6423	3057.52319	79.33748	3.0064
2	23.440	BB	0.6675	9.86438e4	1738.51245	96.9936
Totals :				1.01701e5	1817.84993	

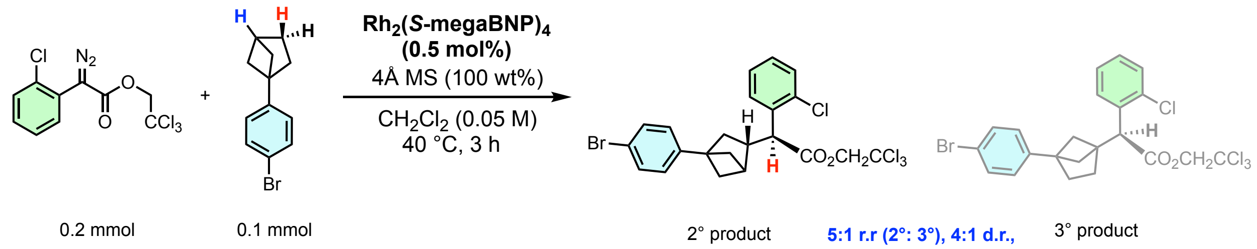

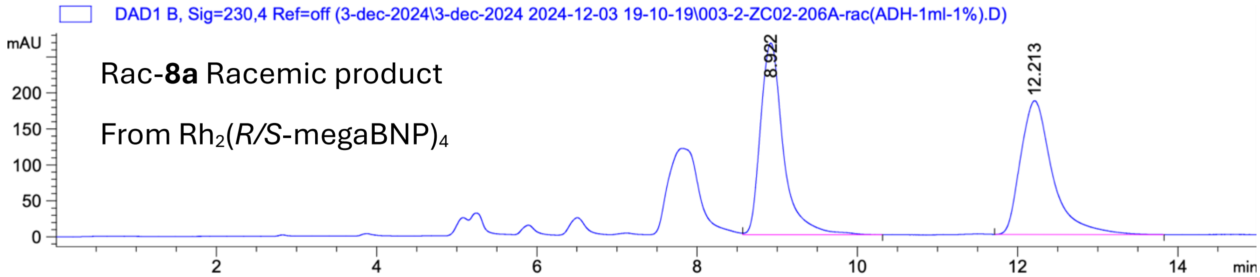

Signal 2: DAD1 B, Sig=230,4 Ref=off

Peak #	RetTime [min]	Type	Width [min]	Area [mAU*s]	Height [mAU]	Area %
1	8.922	VB	0.3048	5294.91895	266.50494	50.6963
2	12.213	BB	0.3929	5149.47314	185.78651	49.3037

Totals : 1.04444e4 452.29146

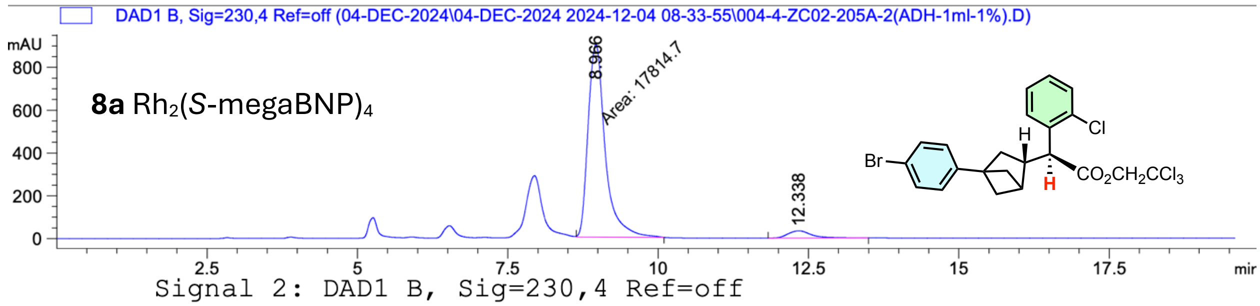

Signal 2: DAD1 B, Sig=230,4 Ref=off

Peak #	RetTime [min]	Type	Width [min]	Area [mAU*s]	Height [mAU]	Area %
1	8.966	MM	0.3298	1.78147e4	900.33276	95.1653
2	12.338	BB	0.3297	905.03510	33.54793	4.8347

Totals : 1.87197e4 933.88070

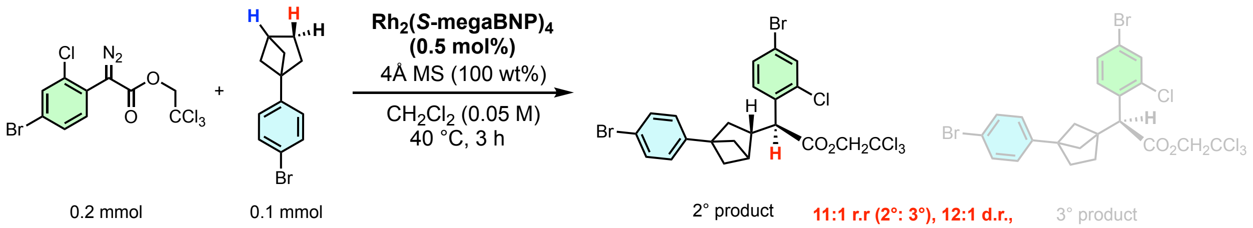

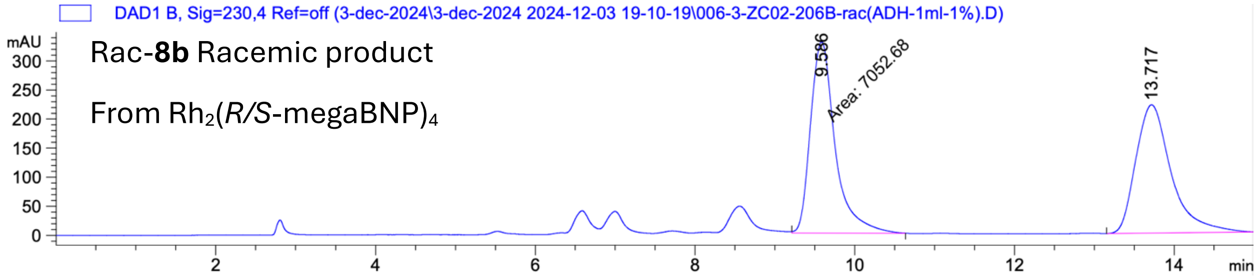

Signal 2: DAD1 B, Sig=230,4 Ref=off

Peak #	RetTime [min]	Type	Width [min]	Area [mAU*s]	Height [mAU]	Area %
1	9.586	MM	0.3576	7052.67627	328.70258	50.9500
2	13.717	BBA	0.4516	6789.67285	220.56035	49.0500
Totals :				1.38423e4	549.26292	

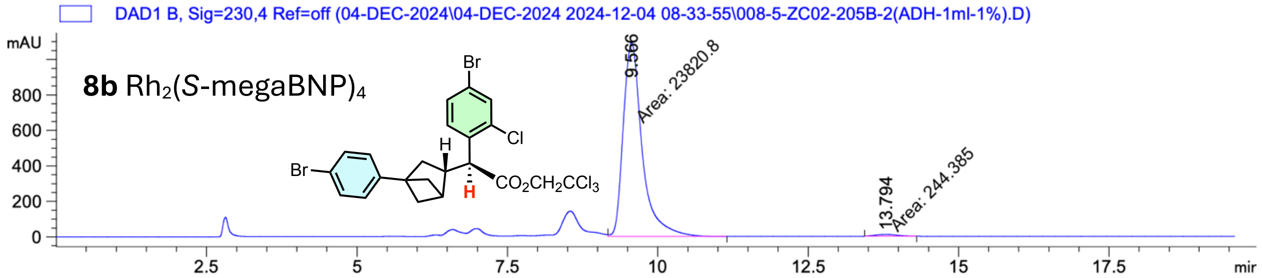

Signal 2: DAD1 B, Sig=230,4 Ref=off

Peak #	RetTime [min]	Type	Width [min]	Area [mAU*s]	Height [mAU]	Area %
1	9.566	MM	0.3637	2.38208e4	1091.67737	98.9845
2	13.794	MM	0.4264	244.38458	9.55212	1.0155
Totals :				2.40652e4	1101.22949	

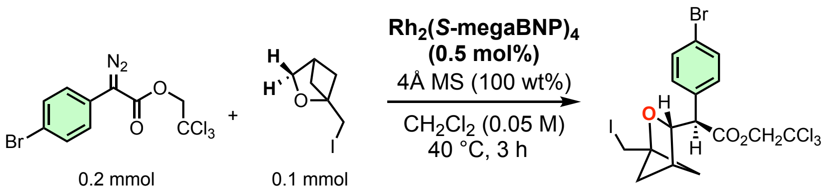

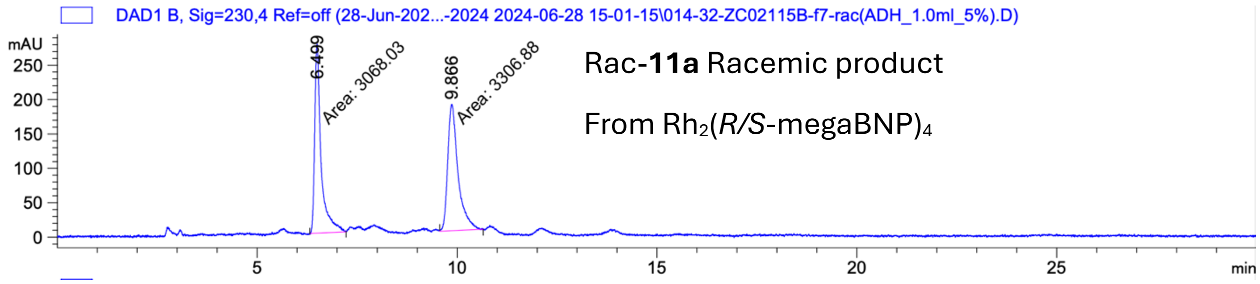

Signal 2: DAD1 B, Sig=230,4 Ref=off

Peak #	RetTime [min]	Type	Width [min]	Area [mAU*s]	Height [mAU]	Area %
1	6.499	MM	0.1860	3068.03491	274.85892	48.1267
2	9.866	MM	0.2997	3306.87817	183.90901	51.8733
Totals :				6374.91309	458.76793	

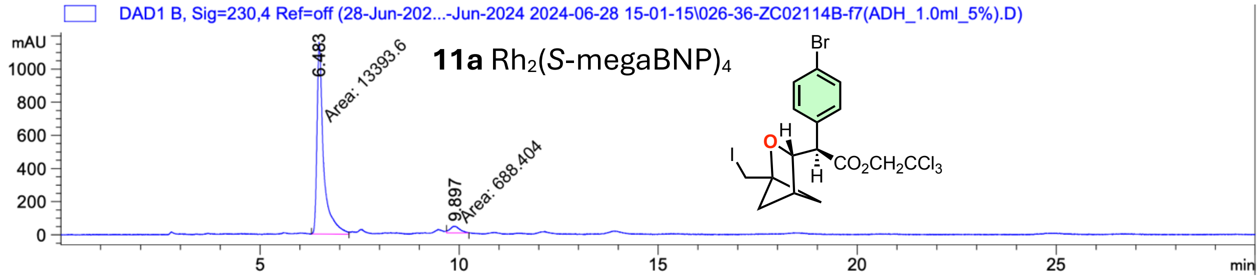

Signal 2: DAD1 B, Sig=230,4 Ref=off

Peak #	RetTime [min]	Type	Width [min]	Area [mAU*s]	Height [mAU]	Area %
1	6.483	MM	0.1928	1.33936e4	1157.52698	95.1115
2	9.897	MM	0.2806	688.40356	40.88302	4.8885
Totals :				1.40820e4	1198.41000	

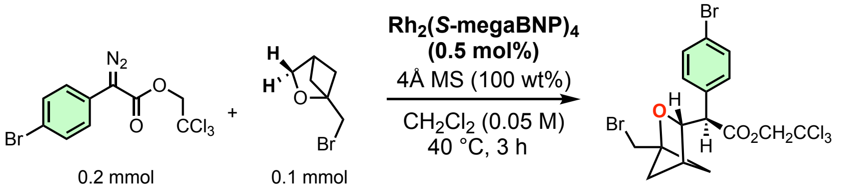

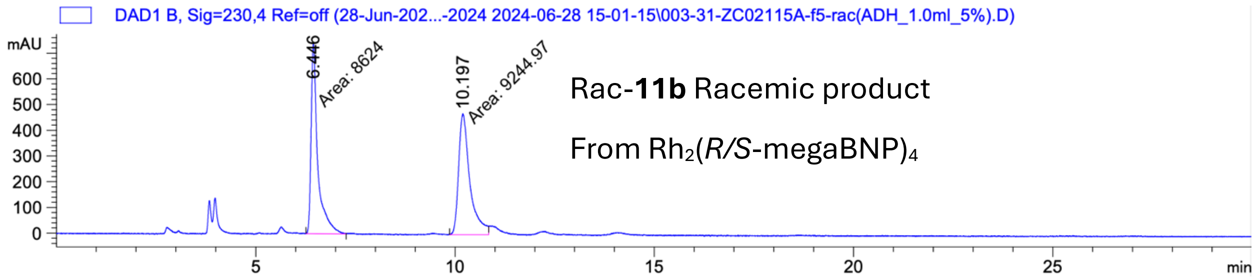

Signal 2: DAD1 B, Sig=230,4 Ref=off

Peak #	RetTime [min]	Type	Width [min]	Area [mAU*s]	Height [mAU]	Area %
1	6.446	MM	0.1947	8623.99512	738.25275	48.2624
2	10.197	MF	0.3274	9244.96777	470.58411	51.7376

Totals : 1.78690e4 1208.83685

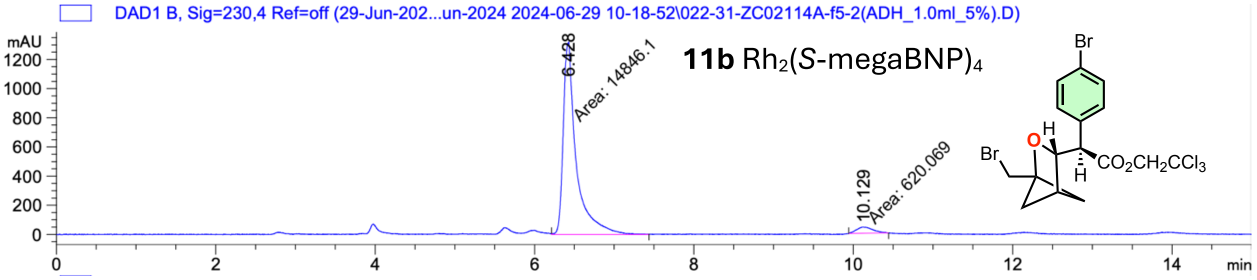

Signal 2: DAD1 B, Sig=230,4 Ref=off

Peak #	RetTime [min]	Type	Width [min]	Area [mAU*s]	Height [mAU]	Area %
1	6.428	MM	0.1873	1.48461e4	1321.26465	95.9908
2	10.129	MM	0.2383	620.06860	43.37490	4.0092

Totals : 1.54661e4 1364.63955

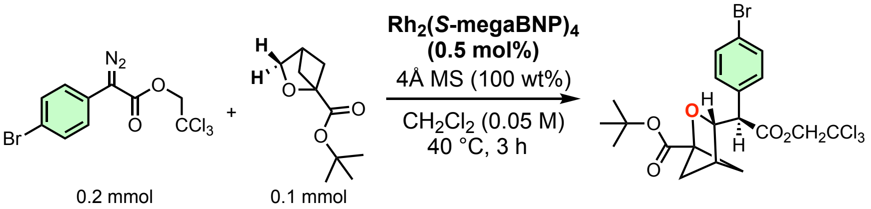

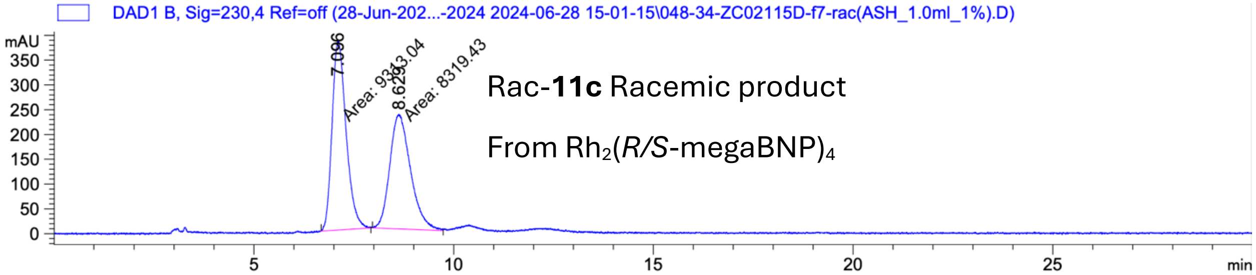

Signal 2: DAD1 B, Sig=230,4 Ref=off

Peak #	RetTime [min]	Type	Width [min]	Area [mAU*s]	Height [mAU]	Area %
1	7.096	MM	0.4082	9313.04297	380.21887	52.8176
2	8.629	MM	0.6002	8319.42969	231.00748	47.1824

Totals : 1.76325e4 611.22635

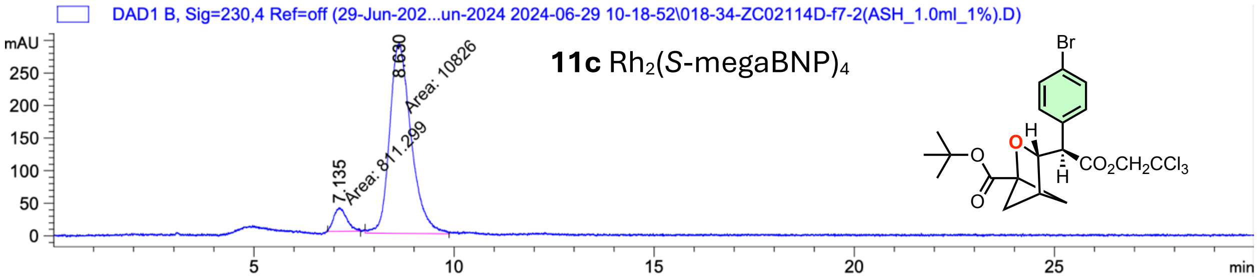

Signal 2: DAD1 B, Sig=230,4 Ref=off

Peak #	RetTime [min]	Type	Width [min]	Area [mAU*s]	Height [mAU]	Area %
1	7.135	MM	0.3731	811.29944	36.23883	6.9715
2	8.630	MM	0.6183	1.08260e4	291.83151	93.0285

Totals : 1.16373e4 328.07034

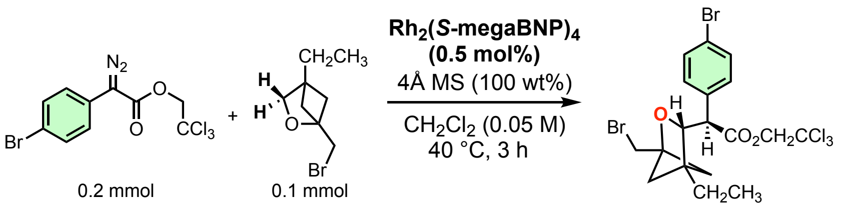

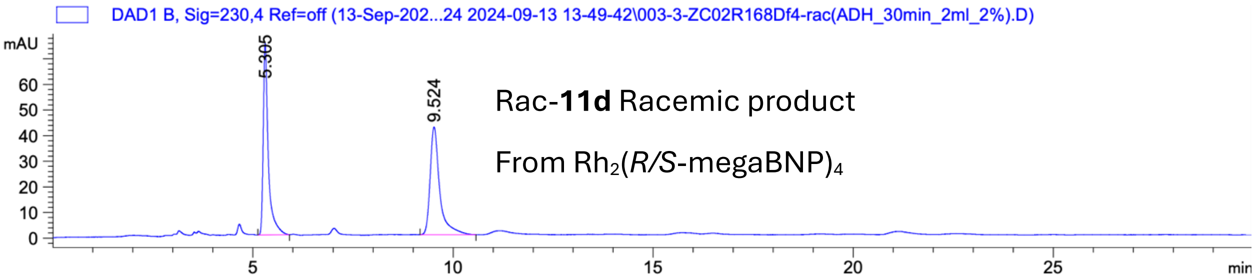


```
Signal 2: DAD1 B, Sig=230,4 Ref=off

Peak RetTime Type  Width     Area      Height     Area
  #   [min]        [min]   [mAU*s]     [mAU]       %
----|-------|----|-------|----------|----------|--------|
   1   5.305 BB    0.1284  659.10632   74.71120  48.5131
   2   9.524 BB    0.2436  699.50903   42.05354  51.4869

Totals :                  1358.61536  116.76474
```

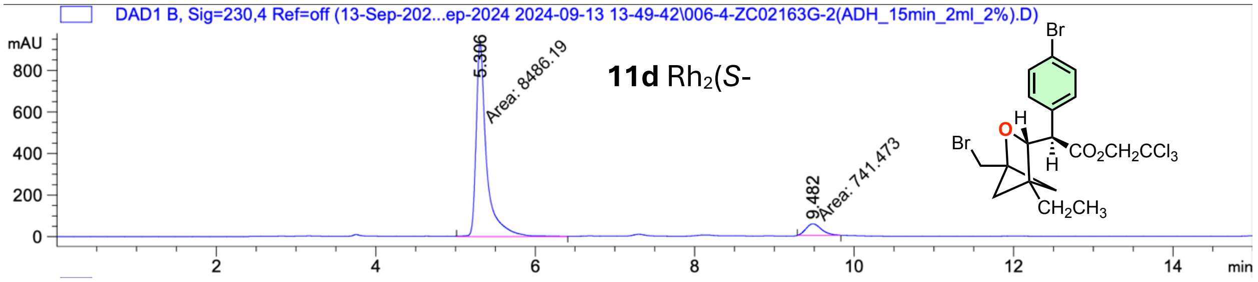


```
Signal 2: DAD1 B, Sig=230,4 Ref=off

Peak RetTime Type  Width     Area      Height     Area
  #   [min]        [min]   [mAU*s]     [mAU]       %
----|-------|----|-------|----------|----------|--------|
   1   5.306 MM    0.1520 8486.18848  930.69873  91.9647
   2   9.482 MM    0.2235  741.47345   55.30072   8.0353

Totals :                  9227.66193  985.99945
```

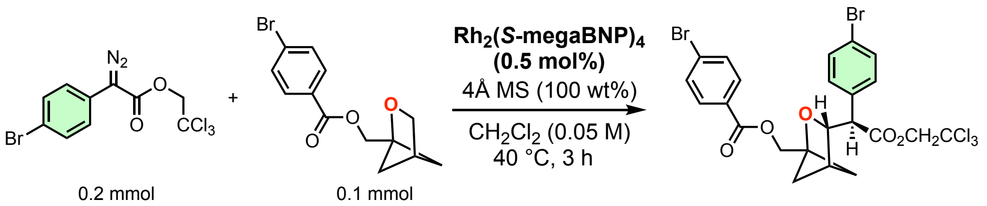

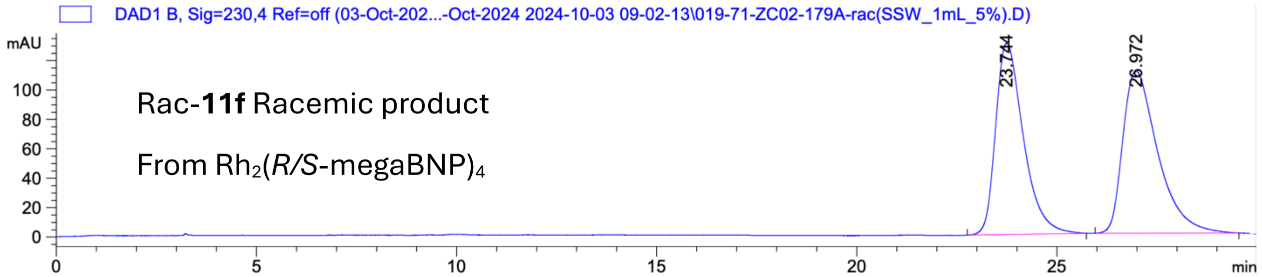

Signal 2: DAD1 B, Sig=230,4 Ref=off

Peak #	RetTime [min]	Type	Width [min]	Area [mAU*s]	Height [mAU]	Area %
1	23.744	BB	0.5657	6256.42871	130.17728	48.2912
2	26.972	BB	0.7141	6699.19287	110.45767	51.7088

Totals : 1.29556e4 240.63495

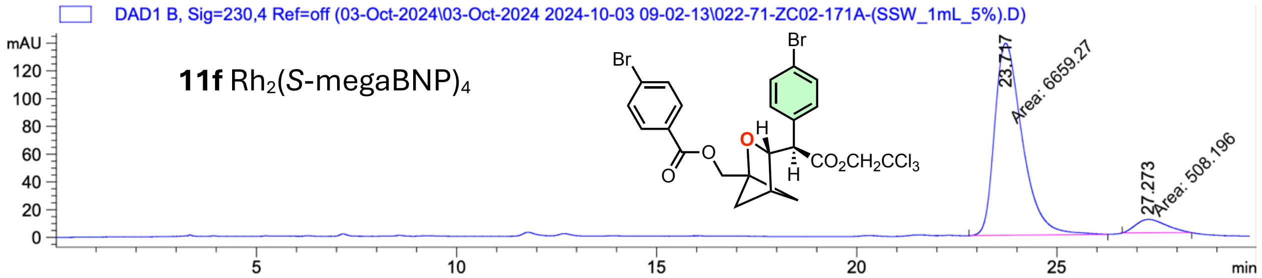

Signal 2: DAD1 B, Sig=230,4 Ref=off

Peak #	RetTime [min]	Type	Width [min]	Area [mAU*s]	Height [mAU]	Area %
1	23.717	MM	0.8015	6659.27393	138.47496	92.9097
2	27.273	MM	0.8799	508.19577	9.62600	7.0903

Totals : 7167.46970 148.10096

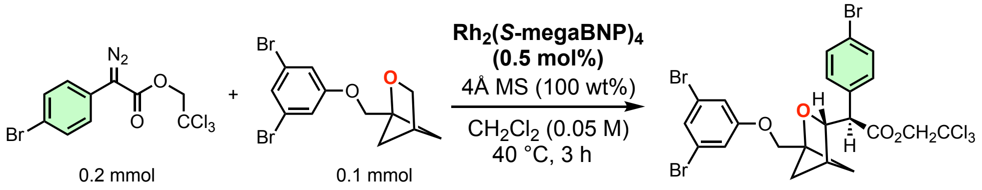

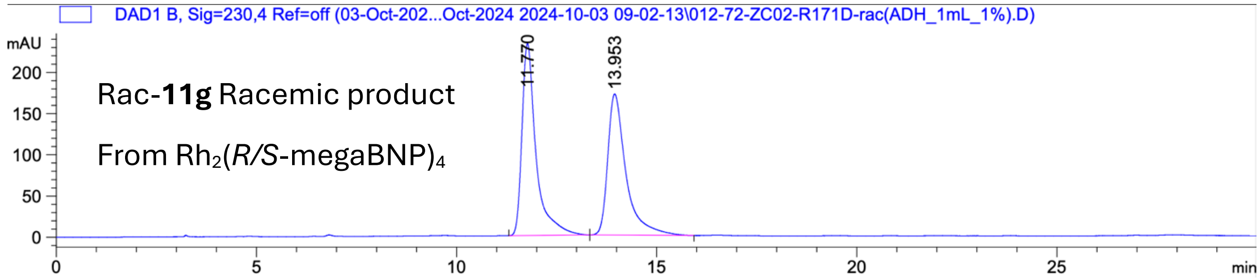

Signal 2: DAD1 B, Sig=230,4 Ref=off

Peak #	RetTime [min]	Type	Width [min]	Area [mAU*s]	Height [mAU]	Area %
1	11.770	BB	0.3547	5708.79346	233.16066	51.3020
2	13.953	BB	0.4540	5419.01807	171.11154	48.6980

Totals : 1.11278e4 404.27220

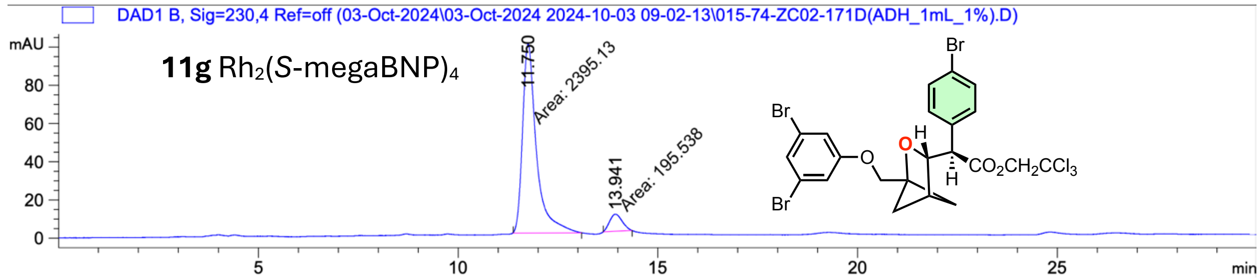

Signal 2: DAD1 B, Sig=230,4 Ref=off

Peak #	RetTime [min]	Type	Width [min]	Area [mAU*s]	Height [mAU]	Area %
1	11.750	MM	0.4034	2395.12988	98.95222	92.4522
2	13.941	MM	0.3638	195.53796	8.95854	7.5478

Totals : 2590.66785 107.91076

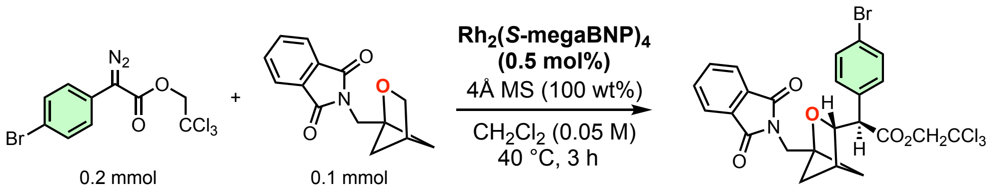

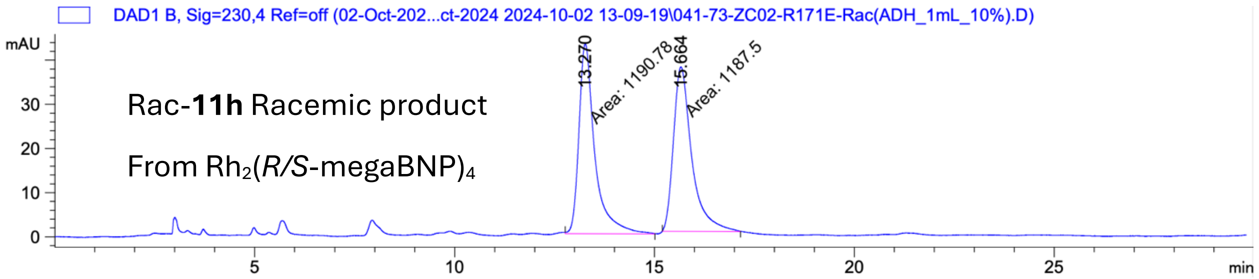

Signal 2: DAD1 B, Sig=230,4 Ref=off

Peak #	RetTime [min]	Type	Width [min]	Area [mAU*s]	Height [mAU]	Area %
1	13.270	MM	0.4615	1190.77551	43.00379	50.0688
2	15.664	MM	0.5317	1187.50464	37.22644	49.9312
Totals :				2378.28015	80.23023	

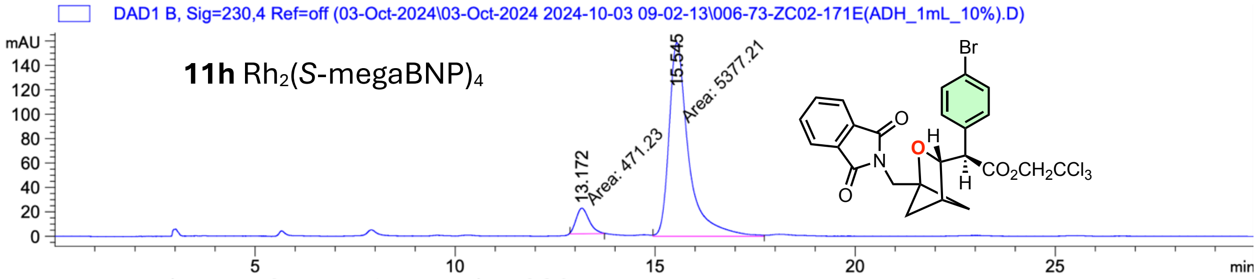

Signal 2: DAD1 B, Sig=230,4 Ref=off

Peak #	RetTime [min]	Type	Width [min]	Area [mAU*s]	Height [mAU]	Area %
1	13.172	MM	0.3755	471.22964	20.91766	8.0574
2	15.545	MM	0.5650	5377.21143	158.62215	91.9426
Totals :				5848.44107	179.53981	

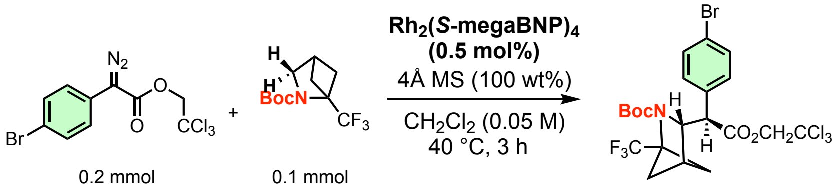

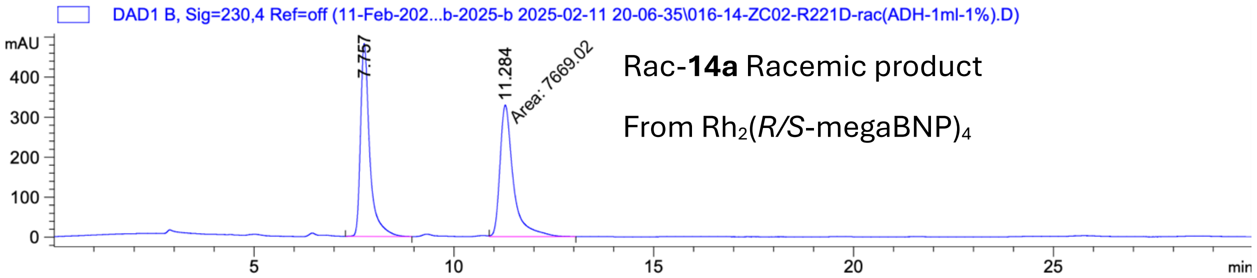

Signal 2: DAD1 B, Sig=230,4 Ref=off

Peak #	RetTime [min]	Type	Width [min]	Area [mAU*s]	Height [mAU]	Area %
1	7.757	BB	0.2380	7726.07178	481.97269	50.1853
2	11.284	FM	0.3892	7669.02441	328.44196	49.8147
Totals :				1.53951e4	810.41464	

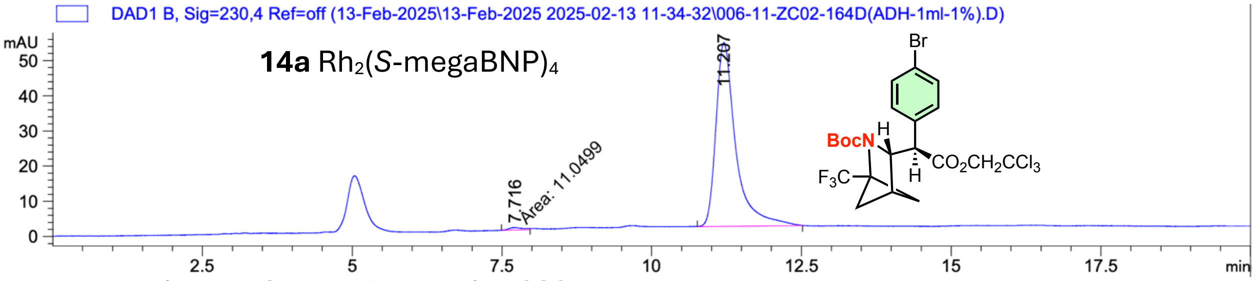

Signal 2: DAD1 B, Sig=230,4 Ref=off

Peak #	RetTime [min]	Type	Width [min]	Area [mAU*s]	Height [mAU]	Area %
1	7.716	MF	0.2657	11.04991	6.93062e-1	0.9099
2	11.207	BB	0.3119	1203.34644	52.21214	99.0901
Totals :				1214.39635	52.90521	

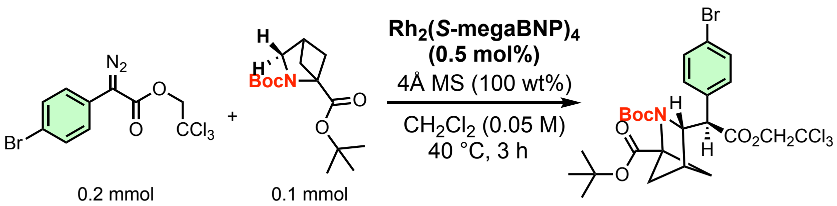

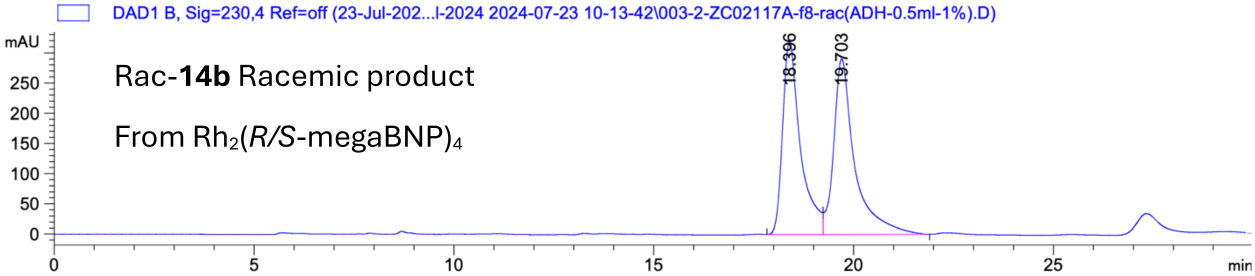


```
Signal 2: DAD1 B, Sig=230,4 Ref=off

Peak RetTime Type  Width     Area       Height      Area
  #   [min]        [min]   [mAU*s]      [mAU]        %
----|-------|----|-------|----------|----------|--------|
   1  18.396 BV    0.4507 9931.39453  319.05734  48.2802
   2  19.703 VB    0.5065 1.06389e4   291.01846  51.7198

Totals :                  2.05703e4   610.07581
```

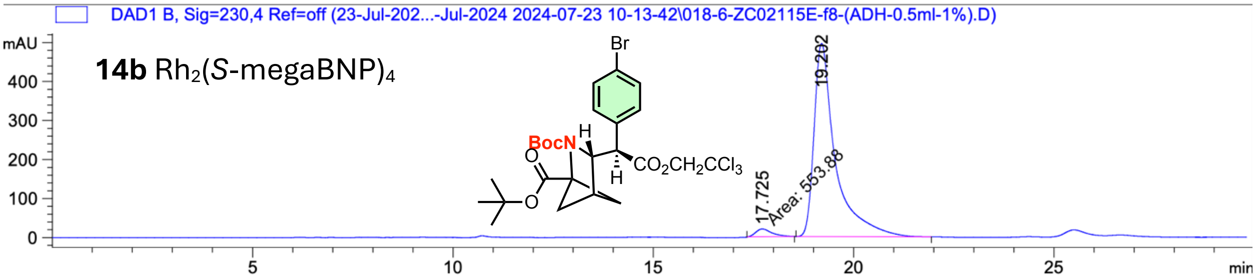


```
Signal 2: DAD1 B, Sig=230,4 Ref=off

Peak RetTime Type  Width     Area       Height      Area
  #   [min]        [min]   [mAU*s]      [mAU]        %
----|-------|----|-------|----------|----------|--------|
   1  17.725 MM    0.4643  553.88019   19.88435   2.9276
   2  19.202 BB    0.5310 1.83656e4   494.89087  97.0724

Totals :                  1.89195e4   514.77522
```

7. Computational Calculation

7.1 General information about computational study

All calculations were performed using Gaussian-16 suite of programs.[8] 3D images of the presented structures were rendered using VMD[9] and Vesta[10]. Geometry and vibrational frequencies of the Rh_2(*S*-megaBNP)$_4$ systems with approximately over 450 atoms and their carbene complexes were calculated by using the ONIOM[11, 12] approach via partitioning of the complex Rh_2(*S*-megaBNP)$_4$ into the two layers. (**Figure S1**) The highlighted catalyst structure, Rh-coordinated carbene fragment, and substrates were treated at the M06[13] level of theory in conjunction with in conjunction with Lanl2dz[14, 15] basis sets and associated effective core potentials (ECP) for rhodium (Rh) and bromine (Br) and 6-31G(d,p)[16, 17] all-electron basis sets for other atoms. The real system including all the atoms was calculated by using the molecular mechanics UFF[18] approach. The resulting approach is called the ONIOM(M06:UFF) approach. Frequency analyses and Gibbs free energy and zero-point energy corrections were calculated at 298.15K and 1 atm.

The steric plots were generated with by SambVca 2.1 program[19]. ETS-NOCV were performed with Multiwfn 3.8(dev)[20] and visualized by VMD[9] using the default parameters and a grid resolution of 0.15 Bohr with the isovalue of 0.0035. The input for ETS-NOCV analysis[21] is obtained from the computational output (.fchk) from Gaussian 16. sobEDAw[22] calculation was also performed with Multiwfn 3.8(dev)[20].

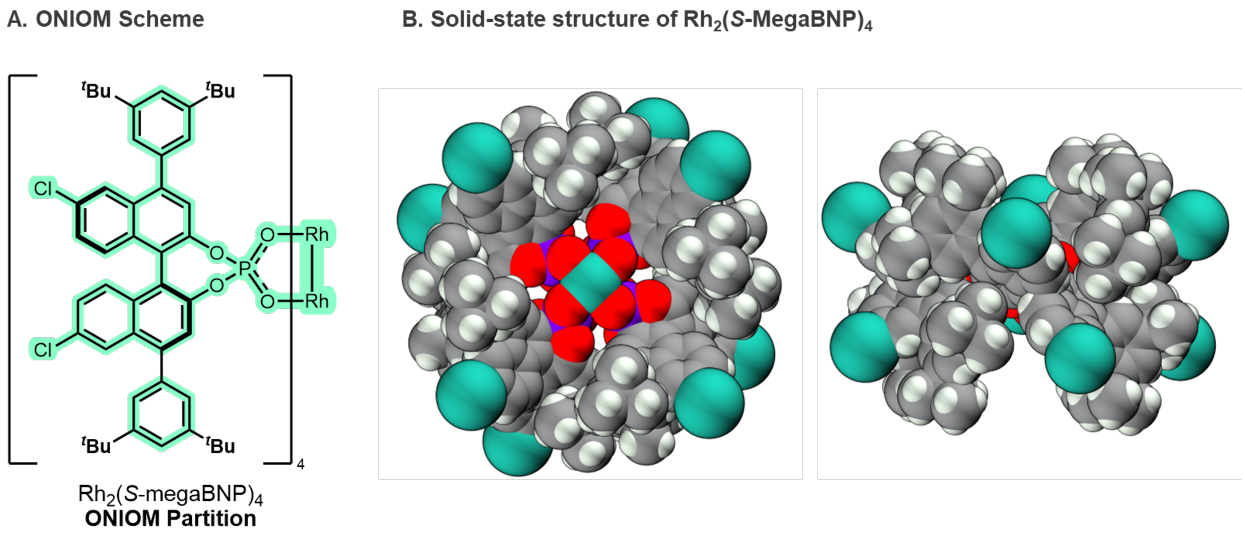

Figure S1. (A) ONIOM Partitioning scheme for the rest of study. The green-highlighted atoms (QM layer) were treated at the M06 level, the rest (all sixteen *t*-butyl groups, the second layer) were treated with UFF force fields. (B) The solid-state Structure of studied catalyst (top view-left, side view-right).

7.2 Validation of the ONIOM partitioning scheme for Rh_2(*S*-megaBNP)$_4$

In general, the two-layer ONIOM approach divides the targeted system into the “model” and “real” layers and treats them at the different levels (quantum mechanical (QM) and lower, respectively) of theory. In our

study we validated two different two-layer ONIOM partitioning schemes and used different levels of theory to treat the second layer. In the first approach all eight 3,5-butylphenyl groups were treated at the lower-level methods (such as UFF and PM6), while the rest of the molecule (i.e. "model" system) was treated at the QM (i.e. M06) level. In the second approach only sixteen *t*-butyl groups were included the second layer and treated at the lower-level methods. We compared structural parameters of the catalyst calculated at the different level of ONIOM approach by their values obtained from the X-ray study.

In general, the ONIOM approach appropriately describes the D_4 symmetrical structure of Rh_2(*S*-megaBNP)$_4$. A second partitioning scheme which includes only sixteen *t*-butyl groups in second layer showed a better alignment (smaller RMSD) than the first partitioning scheme. Calculations showed (see **Figure S2**) that increasing levels of theory to treat the second layer from UFF to PM6 improves the structure alignment. Balancing between the cost of computation and the improvement in structure alignment, in our further calculations we use the UFF approach for a second layer.

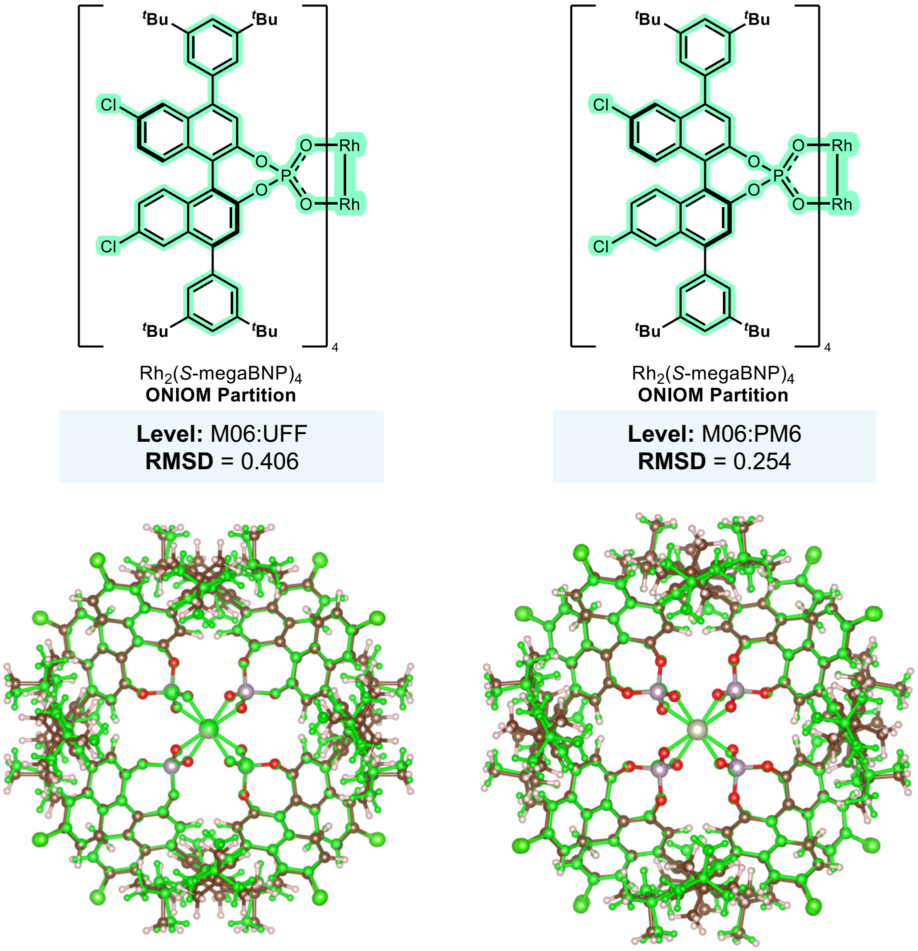

Figure S2. Comparison of the ONIOM calculated and X-ray structures. An overlay between the ONIOM calculated (colorful) and X-ray (green) structures is shown at the bottom. RMSD was calculated using Pymol program with align function.

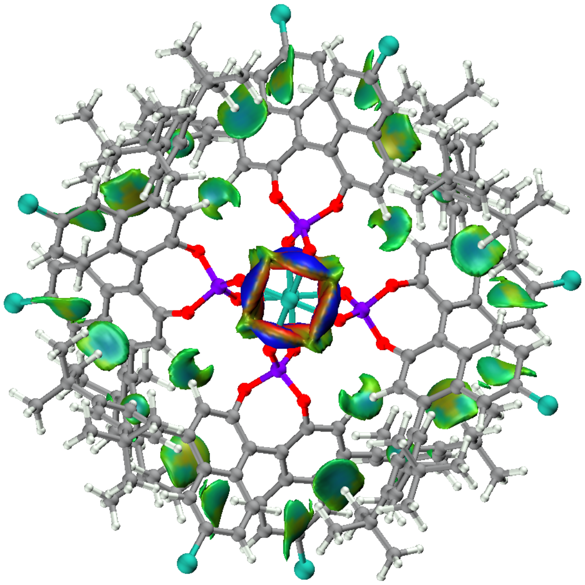

Figure S3. Inter-ligand noncovalent interaction in Rh_2(*S*-megaBNP)$_4$

7.3 Analysis of rhodium-carbene complex of Rh_2(*S*-megaBNP)$_4$

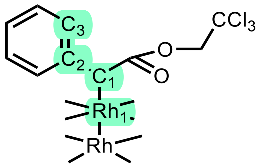

Figure S4. Highlighted important characteristics of donor-acceptor carbene.

In general, for donor-acceptor carbene rhodium complex, the aryl (donor, acceptor) group is in-plane with rhodium-carbene group (φ(Rh1-C1-C2-C3) = 180°, see **Figure S4.**) and ester group is orthogonal to the acceptor group. However, the aryl group in the rhodium-carbene complexes of Rh_2(*S*-megaBNP)$_4$ is significantly tilting (out-of-plane). (**Table S1**) This sets the metal-carbene complex into a pre-transition state position, presumably explaining the high reactivity of Rh_2(megaBNP)$_4$-carbene complex toward unactivated C-H bonds of the bicyclic[2.1.1]hexane system.

Table S1. The calculated dihedral angle φ(Rh1-C1-C2-C3) of the donor group

	**ONION(M06:UFF)**
**Structure I**	-154.8
**Structure II**	-153.2

7.4 Analysis of the C-H functionalization transition states

The amazing diastereoselectivity of Rh_2(*S*-megaBNP)$_4$ prompted us to analyze deeper the transition state of C-H functionalization of the 2° C-H bond. We performed distortion-interaction analysis by Houk-

Bickelhaupt protocol.[23] The partitioning scheme are shown in **Figure S5**. The single-point energy calculations of each transition state and their separated fragments obtained at the ONIOM(M06:UFF) level were performed at the M06/[6-31G(d,p) + Lanl2dz (for Rh and Br)] level of theory in the gas phase. The results are shown in **Table S2**. The **TS1** is more favorable than **TS2** because of better interaction between the two fragments. Energy decomposition analyses were used to understand the interaction between the fragments by sobEDAw method by utilizing Multiwfn_3.8 program. In sobEDAw studies, the transition state was divided into the same two fragments as those in the distortion-interaction analyses. The sobEDAw method decomposed interaction into four components: ΔE_{int} = ΔE_{els}(electrostatic) + ΔE_{xrep} (exchange-repulsion) + ΔE_{orb} (orbital) + ΔE_{disp} (dispersion). The results of sobEDAw analysis showed that the orbital interaction is the most important factor that stabilizes the **TS1** over **TS2**. (**Table S3**) The orbital interaction was further studied by extended Transition State-Natural Orbital for Chemical Valance-method (ETS-NOCV)[21] method. The pair of orbitals dominantly contributing to the ΔE_{orb} component are the σ (C−H) orbital of the substrate and π* orbital of the three-center Rh-Rh-C superelectrophile,[24, 25] in both transition state. The stronger interaction of this orbital pair in **TS1** could be explained as the ligand environment guides the substrate for better orbital overlap. (**Figure S6**)

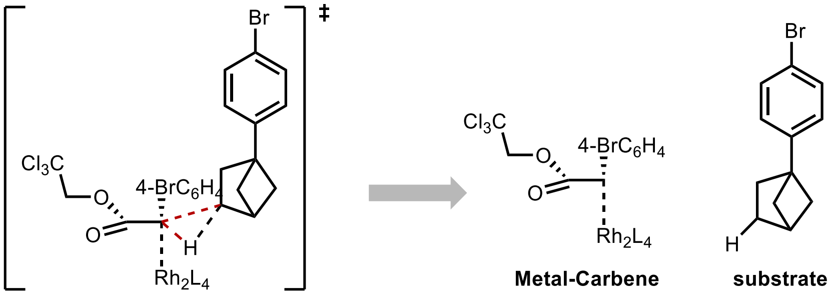

Figure S5. Partitioning scheme for activation strain model (ASM)

Table S2. Distortion-interaction *analysis*. ΔE_{dis}, ΔE_{cat}, and ΔE_{sub} are differences (relative to that in TS1) in total distortion energy, distortion energy of metal-carbene fragment, and distortion energy of substrate fragment, respectively. The total distortion energy is defined as $\Delta E_{dis} = \Delta E_{sub} + \Delta E_{cat}$. $\Delta E_{int} = \Delta E_{TS} - \Delta E_{dis}$, and is difference in interaction energies. (All presented energies are in kcal/mol).

**Structure**	E_{TS} (hartree)	E_{cat} (hartree)	E_{sub}(hartree)	ΔE_{TS}	ΔE_{cat}	ΔE_{sub}	ΔE_{dis}	ΔE_{int}
**TS1**	-18395.79804	-16315.91586	-3036.490851	0.0	0.0	0.0	0.0	0.0
**TS2**	-18395.79872	-16315.92422	-3036.486304	5.2	0.4	-2.9	-2.4	7.7

Table S3. Result of the energy-decomposition analysis at the sobEDAw level for TS1, TS2. These analyses were performed by Multiwfn 3.8 program. (All presented energies are in kcal/mol).

	**TS1**	**TS2**
ΔE_{int} interaction	-82.1	-74.1
ΔE_{els} electrostatic	-98.2	-97.4
ΔE_{xrep} exchange-repulsion	279.0	275.5
ΔE_{orb} orbital	**-195.1**	-187.5

ΔE_{disp} dispersion	-67.8	-64.6

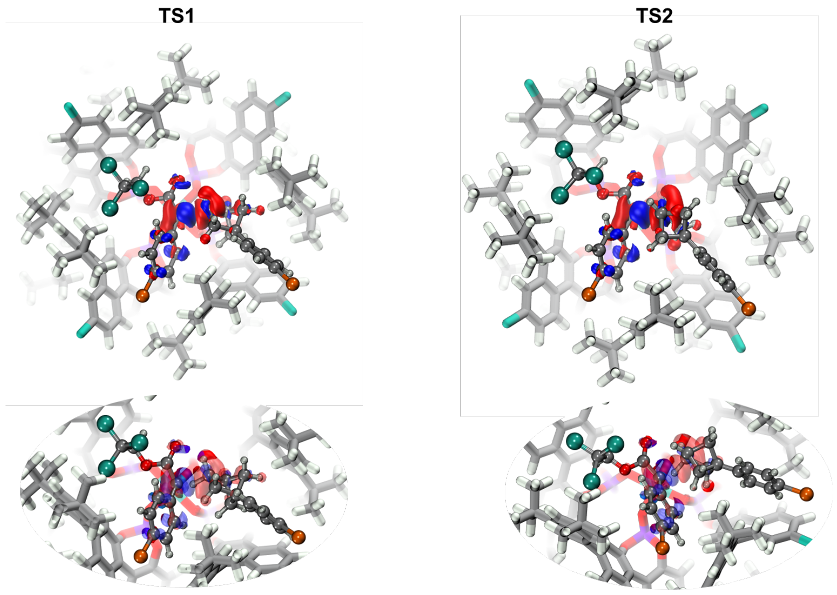

$\Delta E_{orb(1)}$ = -138.17 (kcal/mol)

$\Delta E_{orb(1)}$ = -133.98 (kcal/mol)

Figure S6. ETS-NOCV analysis. The sign of orbital is color coded of the charge flow is red → blue. $\Delta E_{Orb(1)}$ is the energy contributed by the orbital pair with largest eigenvalue. (isovalue = 0.0035)

Table S4. Zero-point energy correction (ZPE), thermal correction to enthalpy (TCH), thermal correction to Gibbs free energy (TCG), electronic energies (E), enthalpies (H), and Gibbs free energies (G) (in hartree) of the studied structures calculated at the ONIOM[M06:UFF] level of theory followed **Figure S1**.

**Structure**	**ZPE**	**TCH**	**TCG**	**E**	**H**	**G**	**Imaginary Frequency (in cm^{-1})**
**Rh_2(S-*megaBNP)$_4$***	3.551786	3.747862	3.307886	-11386.401569	-11382.653707	-11383.093683	-
**Structure I**	3.694577	3.906510	3.435430	-13314.795111	-13310.888601	-13311.359681	-
**Structure II**	3.693680	3.906631	3.433640	-13314.791372	-13310.884740	-13311.357731	-
**TS1**	3.911764	4.135405	3.643695	-13792.671688	-13788.536284	-13789.027993	-212.66
**TS2**	3.910898	4.134677	3.642867	-13792.666035	-13788.531357	-13789.023168	-297.28

Coordination of the computed structures

Rh$_2$(*S*-megaBNP)$_4$

O	-9.28100000	-1.74320000	-30.46260000
O	-8.64750000	-1.10690000	-32.92090000
Rh	-10.39140000	-0.00260000	-30.44560000
P	-8.41720000	-1.97240000	-31.69250000
C	-7.98460000	-4.46360000	-31.31540000
C	-6.61550000	-4.64060000	-31.27630000
C	-6.11820000	-5.62870000	-30.36800000
C	-7.02660000	-6.44830000	-29.62560000
C	-6.50050000	-7.50790000	-28.84400000
H	-7.17330000	-8.19440000	-28.33960000
C	-5.14760000	-7.67790000	-28.72500000
C	-4.24040000	-6.81050000	-29.35930000
H	-3.17230000	-6.94350000	-29.21240000
C	-4.72730000	-5.81690000	-30.16650000
H	-4.03060000	-5.15440000	-30.67140000
C	-8.43570000	-6.19610000	-29.69560000
C	-8.88370000	-5.19850000	-30.53250000
H	-9.93920000	-4.92960000	-30.57630000
Cl	-4.51980000	-8.99180000	-27.75960000
O	-8.53030000	-3.50930000	-32.16240000
C	-5.92690000	-2.40640000	-32.07340000
C	-5.75070000	-3.77560000	-32.11400000
C	-4.76390000	-4.27260000	-33.02390000
C	-3.94460000	-3.36390000	-33.76630000
C	-4.19600000	-1.95470000	-33.69470000
C	-5.19240000	-1.50700000	-32.85630000
H	-5.46070000	-0.45140000	-32.81130000
C	-2.88610000	-3.88990000	-34.54940000
H	-2.19980000	-3.21700000	-35.05390000
C	-2.71700000	-5.24270000	-34.67000000
C	-3.58440000	-6.15010000	-34.03570000
H	-3.45210000	-7.21810000	-34.18370000
C	-4.57670000	-5.66340000	-33.22690000
H	-5.23920000	-6.36020000	-32.72200000
Cl	-1.40460000	-5.87040000	-35.63740000
O	-6.87970000	-1.86090000	-31.22450000
C	-9.42100000	-6.95330000	-28.89350000
C	-10.56520000	-7.45940000	-29.50940000
H	-10.63780000	-7.26690000	-30.57850000
C	-11.52570000	-8.17390000	-28.78470000
C	-11.27530000	-8.37750000	-27.41490000
H	-12.00400000	-8.96320000	-26.86480000
C	-10.15170000	-7.86010000	-26.74940000
C	-9.24240000	-7.13110000	-27.52310000
H	-8.36290000	-6.66480000	-27.08340000
C	-12.81360000	-8.73810000	-29.42310000
C	-12.95830000	-8.35290000	-30.91410000
H	-12.97490000	-7.24910000	-31.02460000
H	-13.90790000	-8.74690000	-31.33770000
H	-12.12530000	-8.77110000	-31.51960000
C	-14.04960000	-8.18430000	-28.67930000
H	-14.09310000	-8.53840000	-27.62880000
H	-14.98830000	-8.51530000	-29.17400000
H	-14.02860000	-7.07310000	-28.67160000
C	-12.80330000	-10.27820000	-29.33110000
H	-11.90170000	-10.68860000	-29.83530000
H	-13.70410000	-10.70720000	-29.82140000
H	-12.79990000	-10.62380000	-28.27630000
C	-9.88080000	-8.05560000	-25.24290000
C	-8.52910000	-8.77910000	-25.05380000
H	-8.53520000	-9.75420000	-25.58740000
H	-8.33150000	-8.97080000	-23.97680000
H	-7.68350000	-8.17470000	-25.44410000
C	-10.96900000	-8.89890000	-24.53660000
H	-11.96240000	-8.40540000	-24.60970000
H	-10.73550000	-9.01850000	-23.45620000
H	-11.03020000	-9.91690000	-24.97900000
C	-9.82750000	-6.67850000	-24.54630000
H	-8.99180000	-6.05780000	-24.93160000
H	-9.67670000	-6.79620000	-23.45120000
H	-10.77760000	-6.12560000	-24.71110000
C	-3.43910000	-0.96900000	-34.49670000
C	-2.93200000	0.17430000	-33.88010000
H	-3.12350000	0.24600000	-32.81070000
C	-2.21770000	1.13530000	-34.60450000
C	-2.01540000	0.88610000	-35.97470000
H	-1.42970000	1.61500000	-36.52450000
C	-2.53380000	-0.23660000	-36.64070000
C	-3.26260000	-1.14640000	-35.86740000
H	-3.72970000	-2.02520000	-36.30750000
C	-1.65260000	2.42240000	-33.96530000
C	-2.03680000	2.56600000	-32.47390000
H	-1.64230000	3.51510000	-32.04980000
H	-3.14050000	2.58280000	-32.36270000
H	-1.61850000	1.73240000	-31.86940000
C	-2.20630000	3.65930000	-34.70770000
H	-1.85270000	3.70360000	-35.75840000
H	-1.87470000	4.59740000	-34.21230000
H	-3.31750000	3.63860000	-34.71490000
C	-0.11260000	2.41150000	-34.05820000
H	0.31710000	3.31170000	-33.56750000
H	0.29780000	1.50930000	-33.55500000
H	0.23240000	2.40880000	-35.11330000
C	-2.33970000	-0.50630000	-38.14770000
C	-1.61690000	-1.85800000	-38.33870000
H	-1.42630000	-2.05470000	-39.41610000
H	-0.64140000	-1.85300000	-37.80590000
H	-2.22140000	-2.70370000	-37.94870000
C	-1.49640000	0.58230000	-38.85360000
H	-1.37780000	0.34970000	-39.93440000
H	-1.98940000	1.57580000	-38.77920000
H	-0.47810000	0.64260000	-38.41210000
C	-3.71730000	-0.55820000	-38.84320000
H	-4.33820000	-1.39400000	-38.45820000
H	-3.60070000	-0.70800000	-39.93850000
H	-4.26970000	0.39200000	-38.67710000
O	-11.50160000	1.73810000	-30.45820000
O	-12.13040000	1.11110000	-32.92010000
P	-12.36300000	1.97200000	-31.68880000
C	-12.79650000	4.46150000	-31.30270000
C	-14.16560000	4.63840000	-31.26560000
C	-14.66480000	5.62270000	-30.35420000

C	-13.75790000	6.43910000	-29.60660000
C	-14.28570000	7.49540000	-28.82160000
H	-13.61390000	8.17970000	-28.31290000
C	-15.63880000	7.66480000	-28.70450000
C	-16.54470000	6.80010000	-29.34430000
H	-17.61310000	6.93250000	-29.19910000
C	-16.05610000	5.81000000	-30.15480000
H	-16.75180000	5.14960000	-30.66390000
C	-12.34860000	6.18720000	-29.67470000
C	-11.89890000	5.19320000	-30.51500000
H	-10.84330000	4.92460000	-30.55780000
Cl	-16.26860000	8.97430000	-27.73450000
O	-12.24910000	3.51070000	-32.15260000
C	-14.85260000	2.40740000	-32.07290000
C	-15.02860000	3.77670000	-32.10850000
C	-16.01340000	4.27730000	-33.01850000
C	-16.83110000	3.37170000	-33.76640000
C	-16.57990000	1.96220000	-33.69980000
C	-15.58550000	1.51100000	-32.86090000
H	-15.31740000	0.45520000	-32.81930000
C	-17.88790000	3.90080000	-34.54980000
H	-18.57300000	3.22990000	-35.05850000
C	-18.05670000	5.25410000	-34.66520000
C	-17.19080000	6.15890000	-34.02540000
H	-17.32280000	7.22750000	-34.16930000
C	-16.20020000	5.66900000	-33.21640000
H	-15.53890000	6.36370000	-32.70720000
Cl	-19.36690000	5.88570000	-35.63310000
O	-13.90150000	1.85860000	-31.22420000
C	-11.36510000	6.94120000	-28.86740000
C	-10.21990000	7.45030000	-29.47870000
H	-10.14500000	7.26250000	-30.54860000
C	-9.26120000	8.16230000	-28.74900000
C	-9.51460000	8.36000000	-27.37890000
H	-8.78740000	8.94380000	-26.82480000
C	-10.63930000	7.83930000	-26.71800000
C	-11.54670000	7.11310000	-27.49660000
H	-12.42680000	6.64460000	-27.06070000
C	-7.97220000	8.72970000	-29.38230000
C	-7.82400000	8.35050000	-30.87450000
H	-7.80680000	7.24720000	-30.98930000
H	-6.87370000	8.74660000	-31.29440000
H	-8.65590000	8.77080000	-31.48020000
C	-6.73750000	8.17360000	-28.63800000
H	-6.69640000	8.52380000	-27.58610000
H	-5.79800000	8.50690000	-29.12950000
H	-6.75810000	7.06240000	-28.63450000
C	-7.98360000	10.26940000	-29.28420000
H	-8.88430000	10.68130000	-29.78880000
H	-7.08190000	10.70090000	-29.77070000
H	-7.98960000	10.61080000	-28.22800000
C	-10.91350000	8.02830000	-25.21120000
C	-12.26590000	8.75040000	-25.02190000
H	-12.25920000	9.72770000	-25.55150000
H	-12.46590000	8.93750000	-23.94450000
H	-13.11040000	8.14730000	-25.41660000
C	-9.82720000	8.86920000	-24.49910000
H	-8.83340000	8.37650000	-24.57230000
H	-10.06300000	8.98420000	-23.41870000
H	-9.76550000	9.88900000	-24.93710000
C	-10.96760000	6.64830000	-24.52050000
H	-11.80220000	6.02880000	-24.91020000
H	-11.12080000	6.76130000	-23.42530000
H	-10.01690000	6.09650000	-24.68560000
C	-17.33510000	0.97990000	-34.50760000
C	-17.84400000	-0.16580000	-33.89690000
H	-17.65520000	-0.24190000	-32.82730000
C	-18.55690000	-1.12360000	-34.62690000
C	-18.75600000	-0.86860000	-35.99660000
H	-19.34060000	-1.59500000	-36.55080000
C	-18.23560000	0.25670000	-36.65680000
C	-17.50830000	1.16290000	-35.87790000
H	-17.03980000	2.04330000	-36.31340000
C	-19.12400000	-2.41310000	-33.99450000
C	-18.74310000	-2.56320000	-32.50290000
H	-19.13890000	-3.51390000	-32.08360000
H	-17.63960000	-2.58070000	-32.38940000
H	-19.16250000	-1.73200000	-31.89570000
C	-18.56920000	-3.64710000	-34.74080000
H	-18.92070000	-3.68700000	-35.79240000
H	-18.90210000	-4.58720000	-34.25000000
H	-17.45800000	-3.62680000	-34.74580000
C	-20.66380000	-2.40120000	-34.09070000
H	-21.09500000	-3.30330000	-33.60480000
H	-21.07490000	-1.50090000	-33.58460000
H	-21.00650000	-2.39380000	-35.14650000
C	-18.42610000	0.53260000	-38.16310000
C	-19.14790000	1.88540000	-38.35020000
H	-19.33600000	2.08660000	-39.42710000
H	-20.12470000	1.87850000	-37.81960000
H	-18.54400000	2.72920000	-37.95520000
C	-19.26810000	-0.55270000	-38.87540000
H	-19.38410000	-0.31570000	-39.95550000
H	-18.77560000	-1.54670000	-38.80390000
H	-20.28750000	-0.61450000	-38.43650000
C	-17.04680000	0.58680000	-38.85510000
H	-16.42660000	1.42080000	-38.46520000
H	-17.16080000	0.74130000	-39.95010000
H	-16.49520000	-0.36430000	-38.69160000
O	-9.27980000	1.74350000	-32.91600000
O	-8.65070000	1.10760000	-30.45640000
Rh	-10.38890000	0.00220000	-32.93530000
P	-8.41790000	1.97270000	-31.68480000
C	-7.98300000	4.46350000	-32.06160000
C	-6.61380000	4.63970000	-32.09800000
C	-6.11400000	5.62740000	-33.00540000
C	-7.02040000	6.44740000	-33.74970000
C	-6.49210000	7.50670000	-34.53030000
H	-7.16350000	8.19350000	-35.03610000
C	-5.13890000	7.67590000	-34.64650000
C	-4.23350000	6.80800000	-34.01020000
H	-3.16500000	6.94040000	-34.15480000
C	-4.72260000	5.81480000	-33.20390000
H	-4.02740000	5.15190000	-32.69750000
C	-8.42980000	6.19600000	-33.68260000
C	-8.88010000	5.19890000	-32.84640000
H	-9.93590000	4.93060000	-32.80470000
Cl	-4.50830000	8.98940000	-35.61060000
O	-8.53100000	3.50970000	-31.21550000
C	-5.92810000	2.40520000	-31.29930000
C	-5.75120000	3.77420000	-31.25840000
C	-4.76610000	4.27070000	-30.34650000
C	-3.94900000	3.36170000	-29.60220000
C	-4.20100000	1.95260000	-29.67420000

C	-5.19580000	1.50540000	-30.51480000
H	-5.46460000	0.44990000	-30.56030000
C	-2.89200000	3.88710000	-28.81660000
H	-2.20730000	3.21380000	-28.31050000
C	-2.72250000	5.23980000	-28.69570000
C	-3.58770000	6.14760000	-29.33210000
H	-3.45520000	7.21560000	-29.18390000
C	-4.57850000	5.66150000	-30.14320000
H	-5.23940000	6.35860000	-30.64970000
Cl	-1.41210000	5.86680000	-27.72500000
O	-6.87960000	1.86030000	-32.15010000
C	-9.41310000	6.95370000	-34.48680000
C	-10.55820000	7.46040000	-33.87330000
H	-10.63320000	7.26800000	-32.80430000
C	-11.51690000	8.17540000	-34.60000000
C	-11.26350000	8.37900000	-35.96930000
H	-11.99070000	8.96510000	-36.52080000
C	-10.13870000	7.86110000	-36.63230000
C	-9.23140000	7.13150000	-35.85680000
H	-8.35130000	6.66470000	-36.29470000
C	-12.80590000	8.74010000	-33.96430000
C	-12.95420000	8.35440000	-32.47370000
H	-12.97140000	7.25060000	-32.36370000
H	-13.90460000	8.74860000	-32.05220000
H	-12.12240000	8.77210000	-31.86620000
C	-14.04060000	8.18720000	-34.71110000
H	-14.08170000	8.54180000	-35.76150000
H	-14.98020000	8.51840000	-34.21820000
H	-14.02000000	7.07600000	-34.71930000
C	-12.79460000	10.28010000	-34.05570000
H	-11.89390000	10.68990000	-33.54940000
H	-13.69630000	10.70950000	-33.56740000
H	-12.78860000	10.62610000	-35.11050000
C	-9.86450000	8.05650000	-38.13830000
C	-8.51200000	8.77930000	-38.32450000
H	-8.51860000	9.75430000	-37.79080000
H	-8.31200000	8.97090000	-39.40100000
H	-7.66760000	8.17440000	-37.93240000
C	-10.95080000	8.90050000	-38.84680000
H	-11.94450000	8.40760000	-38.77580000
H	-10.71490000	9.02010000	-39.92670000
H	-11.01230000	9.91840000	-38.40450000
C	-9.81050000	6.67950000	-38.83490000
H	-8.97600000	6.05820000	-38.44790000
H	-9.65730000	6.79710000	-39.92960000
H	-10.76130000	6.12700000	-38.67220000
C	-3.44640000	0.96670000	-28.87040000
C	-2.93800000	-0.17680000	-29.48570000
H	-3.12690000	-0.24850000	-30.55560000
C	-2.22570000	-1.13790000	-28.75950000
C	-2.02670000	-0.88870000	-27.38880000
H	-1.44260000	-1.61770000	-26.83740000
C	-2.54660000	0.23420000	-26.72410000
C	-3.27320000	1.14410000	-27.49930000
H	-3.74120000	2.02310000	-27.06040000
C	-1.65910000	-2.42510000	-29.39710000
C	-2.03940000	-2.56860000	-30.88950000
H	-1.64380000	-3.51770000	-31.31260000
H	-3.14280000	-2.58530000	-31.00360000
H	-1.61940000	-1.73500000	-31.49300000
C	-2.21490000	-3.66190000	-28.65620000
H	-1.86410000	-3.70620000	-27.60460000
H	-1.88210000	-4.60000000	-29.15070000
H	-3.32620000	-3.64110000	-28.65190000
C	-0.11930000	-2.41440000	-29.30020000
H	0.31160000	-3.31470000	-29.78980000
H	0.29250000	-1.51220000	-29.80230000
H	0.22290000	-2.41180000	-28.24430000
C	-2.35620000	0.50390000	-25.21660000
C	-1.63360000	1.85550000	-25.02390000
H	-1.44560000	2.05220000	-23.94610000
H	-0.65670000	1.85020000	-25.55430000
H	-2.23690000	2.70130000	-25.41550000
C	-1.51500000	-0.58480000	-24.50850000
H	-1.39900000	-0.35230000	-23.42750000
H	-2.00800000	-1.57820000	-24.58420000
H	-0.49550000	-0.64540000	-24.94750000
C	-3.73560000	0.55610000	-24.52460000
H	-4.35520000	1.39210000	-24.91120000
H	-3.62160000	0.70600000	-23.42900000
H	-4.28770000	-0.39400000	-24.69200000
O	-11.49790000	-1.73920000	-32.92490000
O	-12.13210000	-1.11280000	-30.46420000
P	-12.36260000	-1.97280000	-31.69650000
C	-12.79720000	-4.46170000	-32.08500000
C	-14.16630000	-4.63760000	-32.12550000
C	-14.66390000	-5.62130000	-33.03840000
C	-13.75570000	-6.43800000	-33.78420000
C	-14.28230000	-7.49370000	-34.57080000
H	-13.60960000	-8.17830000	-35.07810000
C	-15.63510000	-7.66230000	-34.69110000
C	-16.54210000	-6.79730000	-34.05320000
H	-17.61020000	-6.92900000	-34.20100000
C	-16.05490000	-5.80780000	-33.24120000
H	-16.75130000	-5.14710000	-32.73350000
C	-12.34650000	-6.18690000	-33.71270000
C	-11.89820000	-5.19350000	-32.87100000
H	-10.84260000	-4.92530000	-32.82580000
Cl	-16.26340000	-8.97110000	-35.66310000
O	-12.25120000	-3.51180000	-31.23320000
C	-14.85370000	-2.40640000	-31.31910000
C	-15.03070000	-3.77560000	-31.28440000
C	-16.01820000	-4.27580000	-30.37710000
C	-16.83720000	-3.36990000	-29.63110000
C	-16.58500000	-1.96050000	-29.69660000
C	-15.58810000	-1.50980000	-30.53280000
H	-15.31940000	-0.45410000	-30.57350000
C	-17.89630000	-3.89860000	-28.85050000
H	-18.58240000	-3.22740000	-28.34340000
C	-18.06630000	-5.25180000	-28.73590000
C	-17.19930000	-6.15700000	-29.37380000
H	-17.33230000	-7.22550000	-29.23040000
C	-16.20630000	-5.66740000	-30.18010000
H	-15.54410000	-6.36240000	-30.68770000
Cl	-19.37950000	-5.88290000	-27.77180000
O	-13.89980000	-1.85790000	-32.16490000
C	-11.36140000	-6.94110000	-34.51800000
C	-10.21780000	-7.45070000	-33.90390000
H	-10.14540000	-7.26300000	-32.83390000
C	-9.25750000	-8.16270000	-34.63150000
C	-9.50770000	-8.36020000	-36.00220000
H	-8.77940000	-8.94420000	-36.55460000
C	-10.63080000	-7.83920000	-36.66570000
C	-11.53980000	-7.11280000	-35.88920000

H	-12.41880000	-6.64390000	-36.32710000
C	-7.97020000	-8.73040000	-33.99520000
C	-7.82530000	-8.35130000	-32.50260000
H	-7.80810000	-7.24800000	-32.38770000
H	-6.87600000	-8.74750000	-32.08060000
H	-8.65860000	-8.77140000	-31.89890000
C	-6.73360000	-8.17460000	-34.73670000
H	-6.69010000	-8.52470000	-35.78850000
H	-5.79520000	-8.50810000	-34.24300000
H	-6.75400000	-7.06340000	-34.74020000
C	-7.98160000	-10.27010000	-34.09330000
H	-8.88360000	-10.68190000	-33.59090000
H	-7.08120000	-10.70180000	-33.60480000
H	-7.98530000	-10.61150000	-35.14960000
C	-10.90150000	-8.02800000	-38.17320000
C	-12.25370000	-8.74970000	-38.36570000
H	-12.24850000	-9.72710000	-37.83620000
H	-12.45120000	-8.93660000	-39.44350000
H	-13.09890000	-8.14630000	-37.97290000
C	-9.81370000	-8.86910000	-38.88270000
H	-8.82000000	-8.37670000	-38.80730000
H	-10.04710000	-8.98400000	-39.96370000
H	-9.75340000	-9.88900000	-38.44480000
C	-10.95360000	-6.64790000	-38.86380000
H	-11.78890000	-6.02820000	-38.47610000
H	-11.10420000	-6.76080000	-39.95940000
H	-10.00310000	-6.09640000	-38.69650000
C	-17.34170000	-0.97800000	-28.89060000
C	-17.84850000	0.16810000	-29.50230000
H	-17.65690000	0.24440000	-30.57130000
C	-18.56260000	1.12610000	-28.77370000
C	-18.76500000	0.87110000	-27.40460000
H	-19.35050000	1.59770000	-26.85160000
C	-18.24690000	-0.25470000	-26.74340000
C	-17.51830000	-1.16120000	-27.52070000
H	-17.05140000	-2.04200000	-27.08430000
C	-19.12760000	2.41600000	-29.40730000
C	-18.74340000	2.56600000	-30.89810000
H	-19.13790000	3.51690000	-31.31810000
H	-17.63970000	2.58310000	-31.00920000
H	-19.16190000	1.73500000	-31.50610000
C	-18.57360000	3.64960000	-28.65970000
H	-18.92720000	3.68960000	-27.60880000
H	-18.90500000	4.58990000	-29.15100000
H	-17.46240000	3.62880000	-28.65250000
C	-20.66760000	2.40500000	-29.31440000
H	-21.09720000	3.30740000	-29.80120000
H	-21.07810000	1.50500000	-29.82140000
H	-21.01250000	2.39790000	-28.25930000
C	-18.44110000	-0.53070000	-25.23760000
C	-19.16410000	-1.88310000	-25.05240000
H	-19.35490000	-2.08440000	-23.97590000
H	-20.13970000	-1.87560000	-25.58520000
H	-18.55990000	-2.72730000	-25.44610000
C	-19.28410000	0.55500000	-24.52700000
H	-19.40280000	0.31780000	-23.44730000
H	-18.79090000	1.54870000	-24.59720000
H	-20.30240000	0.61740000	-24.96830000
C	-17.06340000	-0.58600000	-24.54230000
H	-16.44280000	-1.42020000	-24.93090000
H	-17.18010000	-0.74050000	-23.44760000
H	-16.51080000	0.36480000	-24.70430000

Structure I

O	-9.45750000	-1.83180000	-30.71530000
O	-8.71670000	-1.10790000	-33.13570000
Rh	-10.57220000	-0.09220000	-30.59240000
P	-8.53720000	-1.98760000	-31.92520000
C	-8.14610000	-4.44580000	-31.47060000
C	-6.78850000	-4.65080000	-31.33350000
C	-6.37040000	-5.58470000	-30.33210000
C	-7.34340000	-6.28420000	-29.54900000
C	-6.90340000	-7.27820000	-28.63950000
H	-7.63000000	-7.86730000	-28.08790000
C	-5.56620000	-7.50830000	-28.45850000
C	-4.59450000	-6.76380000	-29.15060000
H	-3.54050000	-6.94420000	-28.95930000
C	-4.99920000	-5.82800000	-30.06550000
H	-4.24980000	-5.25900000	-30.60720000
C	-8.73390000	-5.97880000	-29.70410000
C	-9.10630000	-5.07560000	-30.67100000
H	-10.14780000	-4.78310000	-30.80350000
Cl	-5.04030000	-8.74320000	-27.34080000
O	-8.61300000	-3.53060000	-32.39920000
C	-6.02620000	-2.45150000	-32.14010000
C	-5.86380000	-3.82410000	-32.14490000
C	-4.83740000	-4.35510000	-32.98580000
C	-3.96380000	-3.47520000	-33.70000000
C	-4.19060000	-2.06030000	-33.66010000
C	-5.22640000	-1.57990000	-32.88930000
H	-5.47660000	-0.51920000	-32.87480000
C	-2.87640000	-4.03660000	-34.41660000
H	-2.15180000	-3.38710000	-34.89790000
C	-2.72710000	-5.39470000	-34.50100000
C	-3.64120000	-6.27430000	-33.89290000
H	-3.52030000	-7.34730000	-34.01140000
C	-4.66470000	-5.75330000	-33.14690000
H	-5.36480000	-6.42570000	-32.65920000
Cl	-1.37850000	-6.06620000	-35.38610000
O	-7.02450000	-1.87920000	-31.36630000
C	-9.78310000	-6.56270000	-28.84330000
C	-10.93230000	-7.10000000	-29.42420000
H	-10.95930000	-7.07440000	-30.51250000
C	-11.94650000	-7.65560000	-28.63850000
C	-11.76300000	-7.64600000	-27.24490000
H	-12.53080000	-8.12000000	-26.64180000
C	-10.65010000	-7.06290000	-26.62120000
C	-9.66510000	-6.52610000	-27.45460000
H	-8.77200000	-6.05000000	-27.05380000
C	-13.21220000	-8.29180000	-29.24300000
C	-13.29220000	-8.13010000	-30.78070000
H	-13.27700000	-7.05590000	-31.05720000
H	-14.23420000	-8.56650000	-31.17850000
H	-12.44880000	-8.65030000	-31.28410000
C	-14.46980000	-7.63060000	-28.64100000
H	-14.55450000	-7.81020000	-27.55000000
H	-15.38750000	-8.04860000	-29.10560000
H	-14.44690000	-6.53330000	-28.81470000
C	-13.22620000	-9.80160000	-28.92550000
H	-12.31090000	-10.28890000	-29.32610000
H	-14.11230000	-10.29200000	-29.38390000
H	-13.26870000	-9.98740000	-27.83170000

C	-10.46320000	-7.00080000	-25.09380000
C	-9.18160000	-7.77080000	-24.70570000
H	-9.24220000	-8.82380000	-25.05680000
H	-9.04400000	-7.77750000	-23.60260000
H	-8.27590000	-7.30810000	-25.15110000
C	-11.64160000	-7.62580000	-24.30920000
H	-12.59320000	-7.09960000	-24.53600000
H	-11.47080000	-7.54780000	-23.21340000
H	-11.75170000	-8.70500000	-24.55110000
C	-10.32820000	-5.52850000	-24.64500000
H	-9.43190000	-5.04510000	-25.08640000
H	-10.23010000	-5.46070000	-23.53990000
H	-11.22190000	-4.94590000	-24.95010000
C	-3.36500000	-1.09830000	-34.42230000
C	-2.87230000	0.03920000	-33.78160000
H	-3.12720000	0.12620000	-32.72640000
C	-2.09600000	0.97760000	-34.46640000
C	-1.81440000	0.72000000	-35.81800000
H	-1.17990000	1.43330000	-36.33460000
C	-2.31540000	-0.39360000	-36.50670000
C	-3.10710000	-1.28650000	-35.77960000
H	-3.56340000	-2.15660000	-36.24780000
C	-1.54810000	2.25380000	-33.79870000
C	-2.02400000	2.41670000	-32.33590000
H	-1.64180000	3.36310000	-31.89480000
H	-3.13250000	2.45100000	-32.29560000
H	-1.65820000	1.58240000	-31.69900000
C	-2.02540000	3.49790000	-34.58170000
H	-1.59650000	3.53300000	-35.60430000
H	-1.71420000	4.43210000	-34.06620000
H	-3.13340000	3.49630000	-34.66920000
C	-0.00580000	2.21040000	-33.79090000
H	0.41000000	3.10510000	-33.27840000
H	0.35200000	1.30360000	-33.25680000
H	0.40730000	2.19300000	-34.82110000
C	-2.03700000	-0.66930000	-37.99630000
C	-1.33160000	-2.03600000	-38.14250000
H	-1.08030000	-2.23870000	-39.20630000
H	-0.38990000	-2.04970000	-37.55200000
H	-1.97510000	-2.86900000	-37.78950000
C	-1.13100000	0.39900000	-38.65490000
H	-0.95420000	0.15970000	-39.72620000
H	-1.60660000	1.40280000	-38.61290000
H	-0.13880000	0.43970000	-38.15530000
C	-3.37130000	-0.69520000	-38.77340000
H	-4.03100000	-1.51770000	-38.42640000
H	-3.19220000	-0.84960000	-39.85970000
H	-3.91350000	0.26620000	-38.64170000
O	-11.55940000	1.72500000	-30.68610000
O	-12.24820000	1.16530000	-33.15740000
P	-12.44520000	1.97570000	-31.90270000
C	-12.82130000	4.43890000	-31.34110000
C	-14.18310000	4.64830000	-31.22750000
C	-14.61290000	5.58720000	-30.23360000
C	-13.64910000	6.31740000	-29.46740000
C	-14.10200000	7.32840000	-28.58310000
H	-13.38160000	7.93810000	-28.04590000
C	-15.44230000	7.53740000	-28.39870000
C	-16.40430000	6.75540000	-29.06380000
H	-17.46030000	6.91570000	-28.86610000
C	-15.98740000	5.80800000	-29.96080000
H	-16.72890000	5.21030000	-30.48220000
C	-12.25550000	6.01380000	-29.59970000
C	-11.87150000	5.07630000	-30.53140000
H	-10.82990000	4.77010000	-30.62340000
Cl	-15.98530000	8.78590000	-27.30450000
O	-12.34010000	3.54200000	-32.27960000
C	-14.95220000	2.47810000	-32.11980000
C	-15.10520000	3.85100000	-32.07180000
C	-16.12780000	4.41690000	-32.89800000
C	-17.00820000	3.56760000	-33.64080000
C	-16.78190000	2.15250000	-33.66440000
C	-15.74790000	1.64150000	-32.91230000
H	-15.49410000	0.58270000	-32.95000000
C	-18.09350000	4.15830000	-34.33650000
H	-18.82220000	3.52860000	-34.83740000
C	-18.23270000	5.51940000	-34.37980000
C	-17.30790000	6.37210000	-33.75040000
H	-17.41690000	7.44920000	-33.83990000
C	-16.28810000	5.82060000	-33.02130000
H	-15.58010000	6.47420000	-32.52040000
Cl	-19.57710000	6.22800000	-35.24170000
O	-13.96760000	1.86740000	-31.35940000
C	-11.22110000	6.63350000	-28.74270000
C	-10.07930000	7.18590000	-29.32210000
H	-10.03960000	7.14170000	-30.40960000
C	-9.08100000	7.76700000	-28.53580000
C	-9.27900000	7.78260000	-27.14500000
H	-8.52050000	8.27260000	-26.54300000
C	-10.38940000	7.19640000	-26.52200000
C	-11.35080000	6.61840000	-27.35430000
H	-12.23130000	6.12200000	-26.94870000
C	-7.80280000	8.38540000	-29.13380000
C	-7.69160000	8.17430000	-30.66260000
H	-7.70070000	7.09110000	-30.90010000
H	-6.74140000	8.59700000	-31.05580000
H	-8.52450000	8.67670000	-31.20040000
C	-6.55700000	7.74020000	-28.48570000
H	-6.47140000	7.99060000	-27.40850000
H	-5.62700000	8.10250000	-28.97420000
H	-6.60310000	6.63420000	-28.58280000
C	-7.79370000	9.90400000	-28.86320000
H	-8.69660000	10.38000000	-29.30370000
H	-6.89390000	10.37800000	-29.31210000
H	-7.78160000	10.12320000	-27.77480000
C	-10.58900000	7.15180000	-24.99600000
C	-11.90590000	7.87110000	-24.62850000
H	-11.88920000	8.91950000	-24.99770000
H	-12.05040000	7.89050000	-23.52650000
H	-12.78820000	7.36130000	-25.06950000
C	-9.44370000	7.83660000	-24.21250000
H	-8.47150000	7.33680000	-24.41420000
H	-9.62650000	7.77960000	-23.11730000
H	-9.36790000	8.91250000	-24.48130000
C	-10.66210000	5.68050000	-24.53030000
H	-11.53600000	5.15530000	-24.96870000
H	-10.76050000	5.62080000	-23.42470000
H	-9.74150000	5.13410000	-24.82970000
C	-17.58810000	1.22430000	-34.48790000
C	-18.06340000	0.03850000	-33.92560000
H	-17.82550000	-0.10570000	-32.87300000
C	-18.80150000	-0.87720000	-34.68040000
C	-19.06640000	-0.54560000	-36.01930000
H	-19.66880000	-1.24440000	-36.59090000

C	-18.58690000	0.62190000	-36.62940000
C	-17.83040000	1.48810000	-35.83570000
H	-17.38800000	2.39490000	-36.24360000
C	-19.32250000	-2.20870000	-34.10430000
C	-18.88440000	-2.44120000	-32.63930000
H	-19.25410000	-3.41990000	-32.26270000
H	-17.77780000	-2.45290000	-32.56950000
H	-19.28820000	-1.65220000	-31.96850000
C	-18.77640000	-3.38760000	-34.94180000
H	-19.16270000	-3.36980000	-35.98170000
H	-19.07850000	-4.36100000	-34.49790000
H	-17.66610000	-3.35020000	-34.98200000
C	-20.86520000	-2.22030000	-34.14170000
H	-21.26250000	-3.15540000	-33.69040000
H	-21.27130000	-1.35690000	-33.57140000
H	-21.24970000	-2.16270000	-35.18130000
C	-18.85010000	0.98210000	-38.10350000
C	-19.58800000	2.33750000	-38.17730000
H	-19.82830000	2.59940000	-39.23060000
H	-20.53850000	2.29150000	-37.60270000
H	-18.97130000	3.16270000	-37.76320000
C	-19.71890000	-0.06530000	-38.84100000
H	-19.88760000	0.23620000	-39.89780000
H	-19.21760000	-1.05740000	-38.85500000
H	-20.71610000	-0.16270000	-38.35960000
C	-17.50560000	1.08880000	-38.85580000
H	-16.87240000	1.90450000	-38.44860000
H	-17.67260000	1.30500000	-39.93340000
H	-16.94080000	0.13480000	-38.77420000
O	-9.33300000	1.75410000	-32.98070000
O	-8.80540000	0.96470000	-30.52390000
Rh	-10.47480000	0.02330000	-33.19920000
P	-8.51130000	1.87010000	-31.71720000
C	-7.98360000	4.34300000	-32.00490000
C	-6.60990000	4.47670000	-32.05460000
C	-6.08970000	5.46120000	-32.95530000
C	-6.97660000	6.32970000	-33.66770000
C	-6.42230000	7.38380000	-34.43730000
H	-7.07620000	8.10520000	-34.91750000
C	-5.06590000	7.50220000	-34.57740000
C	-4.18360000	6.58550000	-33.97830000
H	-3.11400000	6.67730000	-34.14390000
C	-4.69620000	5.59670000	-33.18110000
H	-4.01840000	4.89630000	-32.70260000
C	-8.39240000	6.12380000	-33.58650000
C	-8.86180000	5.12070000	-32.76950000
H	-9.92210000	4.87590000	-32.73290000
Cl	-4.40170000	8.80970000	-35.52810000
O	-8.55400000	3.39070000	-31.17340000
C	-5.99760000	2.21570000	-31.26430000
C	-5.76990000	3.57850000	-31.22710000
C	-4.75910000	4.04280000	-30.32480000
C	-3.97620000	3.10830000	-29.57560000
C	-4.28320000	1.71000000	-29.63900000
C	-5.29220000	1.29490000	-30.47800000
H	-5.59470000	0.24880000	-30.52310000
C	-2.89230000	3.59450000	-28.80110000
H	-2.23730000	2.89670000	-28.28880000
C	-2.65790000	4.93890000	-28.70130000
C	-3.48380000	5.87650000	-29.34610000
H	-3.29850000	6.93900000	-29.21690000
C	-4.50450000	5.42570000	-30.14050000
H	-5.13590000	6.14750000	-30.64950000
Cl	-1.31140000	5.51840000	-27.74950000
O	-6.95550000	1.69590000	-32.11850000
C	-9.36620000	6.92350000	-34.36200000
C	-10.49450000	7.44090000	-33.72360000
H	-10.56290000	7.23100000	-32.65720000
C	-11.44780000	8.18400000	-34.42490000
C	-11.21470000	8.40710000	-35.79190000
H	-11.94130000	9.01280000	-36.32400000
C	-10.10970000	7.88300000	-36.47680000
C	-9.20170000	7.12560000	-35.73190000
H	-8.33830000	6.65220000	-36.19530000
C	-12.71590000	8.75620000	-33.76130000
C	-12.84500000	8.36150000	-32.27130000
H	-12.88220000	7.25800000	-32.17090000
H	-13.78050000	8.76910000	-31.83000000
H	-11.99490000	8.75830000	-31.67500000
C	-13.97060000	8.22390000	-34.49020000
H	-14.02200000	8.58030000	-35.53950000
H	-14.89740000	8.56920000	-33.98290000
H	-13.96830000	7.11230000	-34.49940000
C	-12.68950000	10.29730000	-33.83910000
H	-11.77360000	10.69260000	-33.34860000
H	-13.57550000	10.73230000	-33.32760000
H	-12.70320000	10.65420000	-34.89010000
C	-9.85910000	8.10000000	-37.98090000
C	-8.49750000	8.80290000	-38.17870000
H	-8.47790000	9.76850000	-37.62840000
H	-8.31290000	9.00990000	-39.25520000
H	-7.65660000	8.17720000	-37.81260000
C	-10.94120000	8.97460000	-38.65920000
H	-10.71910000	9.10850000	-39.74030000
H	-11.94220000	8.49740000	-38.58260000
H	-10.97860000	9.98600000	-38.19960000
C	-9.84070000	6.73450000	-38.70220000
H	-9.01010000	6.09240000	-38.34170000
H	-9.70500000	6.86870000	-39.79740000
H	-10.79770000	6.19520000	-38.53210000
C	-3.55670000	0.70530000	-28.83100000
C	-3.04730000	-0.43540000	-29.45150000
H	-3.22300000	-0.50140000	-30.52440000
C	-2.34670000	-1.40130000	-28.72370000
C	-2.16620000	-1.16140000	-27.35440000
H	-1.59500000	-1.88430000	-26.77710000
C	-2.68810000	-0.04410000	-26.68590000
C	-3.40220000	0.87810000	-27.45370000
H	-3.87950000	1.76180000	-27.03700000
C	-1.77270000	-2.67970000	-29.36100000
C	-2.15860000	-2.82970000	-30.85030000
H	-1.76610000	-3.78120000	-31.27090000
H	-3.26270000	-2.84330000	-30.95710000
H	-1.73970000	-2.00010000	-31.46000000
C	-2.31030000	-3.92100000	-28.61400000
H	-1.93080000	-3.97390000	-27.57270000
H	-1.99270000	-4.85670000	-29.12290000
H	-3.42050000	-3.89640000	-28.57750000
C	-0.23320000	-2.65170000	-29.26980000
H	0.20540000	-3.55300000	-29.75060000
H	0.16770000	-1.75090000	-29.78320000
H	0.11100000	-2.63190000	-28.21430000
C	-2.45880000	0.12360000	-25.17260000
C	-0.94310000	0.19490000	-24.88420000

H	-0.75550000	0.36760000	-23.80220000
H	-0.42730000	-0.74760000	-25.16250000
H	-0.48020000	1.02800000	-25.45640000
C	-3.07060000	-1.07830000	-24.42020000
H	-2.95940000	-0.95310000	-23.32120000
H	-4.15360000	-1.16930000	-24.65370000
H	-2.57350000	-2.03140000	-24.69700000
C	-3.10420000	1.41160000	-24.60570000
H	-2.67990000	2.31540000	-25.09470000
H	-2.91310000	1.50140000	-23.51410000
H	-4.20620000	1.39940000	-24.75080000
O	-11.62380000	-1.74690000	-33.29490000
O	-12.33580000	-1.14250000	-30.84080000
P	-12.50560000	-1.98870000	-32.09860000
C	-12.93090000	-4.50360000	-32.42530000
C	-14.30140000	-4.66770000	-32.50960000
C	-14.77650000	-5.65720000	-33.42590000
C	-13.85770000	-6.52830000	-34.09280000
C	-14.37990000	-7.58750000	-34.87810000
H	-13.70770000	-8.31450000	-35.32310000
C	-15.72910000	-7.70240000	-35.08010000
C	-16.63700000	-6.77980000	-34.52850000
H	-17.69780000	-6.86760000	-34.74580000
C	-16.15920000	-5.79010000	-33.71160000
H	-16.85340000	-5.08340000	-33.26540000
C	-12.44750000	-6.31770000	-33.94880000
C	-12.01770000	-5.28960000	-33.13880000
H	-10.95880000	-5.04360000	-33.06030000
Cl	-16.35160000	-9.01340000	-36.05270000
O	-12.39890000	-3.52930000	-31.59510000
C	-14.97160000	-2.44690000	-31.67280000
C	-15.17670000	-3.81260000	-31.67230000
C	-16.16320000	-4.32120000	-30.76520000
C	-16.89540000	-3.42280000	-29.92550000
C	-16.56920000	-2.02750000	-29.91580000
C	-15.62120000	-1.56500000	-30.79910000
H	-15.31190000	-0.51990000	-30.79910000
C	-17.95400000	-3.93460000	-29.13340000
H	-18.56350000	-3.25720000	-28.54320000
C	-18.22530000	-5.27580000	-29.11940000
C	-17.45570000	-6.18390000	-29.86630000
H	-17.67120000	-7.24720000	-29.81050000
C	-16.44750000	-5.70680000	-30.66220000
H	-15.85540000	-6.41230000	-31.23630000
Cl	-19.54520000	-5.88730000	-28.15220000
O	-14.04990000	-1.89860000	-32.55180000
C	-11.43240000	-7.13820000	-34.64570000
C	-10.33730000	-7.62170000	-33.92740000
H	-10.33260000	-7.37630000	-32.86670000
C	-9.33780000	-8.37550000	-34.54700000
C	-9.49450000	-8.65400000	-35.91480000
H	-8.73330000	-9.26990000	-36.38300000
C	-10.56590000	-8.16960000	-36.67800000
C	-11.51880000	-7.39170000	-36.01410000
H	-12.35560000	-6.94060000	-36.54380000
C	-8.09810000	-8.89770000	-33.79480000
C	-8.04500000	-8.42430000	-32.32230000
H	-8.03230000	-7.31590000	-32.27570000
H	-7.12450000	-8.79140000	-31.81810000
H	-8.91440000	-8.80710000	-31.74510000
C	-6.81450000	-8.38910000	-34.48960000
H	-6.70030000	-8.81150000	-35.50910000
H	-5.91050000	-8.68270000	-33.91330000
H	-6.83500000	-7.28100000	-34.57240000
C	-8.10700000	-10.44060000	-33.79490000
H	-9.03920000	-10.82100000	-33.32390000
H	-7.23950000	-10.83880000	-33.22490000
H	-8.04610000	-10.84900000	-34.82530000
C	-10.73280000	-8.44830000	-38.18360000
C	-12.07840000	-9.16770000	-38.42690000
H	-12.12390000	-10.11050000	-37.83980000
H	-12.20260000	-9.41920000	-39.50260000
H	-12.94100000	-8.53350000	-38.13330000
C	-9.61140000	-9.34230000	-38.76600000
H	-8.61840000	-8.85470000	-38.65680000
H	-9.77480000	-9.52300000	-39.85080000
H	-9.59320000	-10.33320000	-38.26260000
C	-10.71790000	-7.11300000	-38.95930000
H	-11.56990000	-6.46260000	-38.67080000
H	-10.79280000	-7.29190000	-40.05390000
H	-9.77400000	-6.56090000	-38.75920000
C	-17.19750000	-1.07970000	-28.97020000
C	-17.71660000	0.12740000	-29.43770000
H	-17.64240000	0.28260000	-30.51290000
C	-18.30240000	1.04740000	-28.56350000
C	-18.35750000	0.69270000	-27.20650000
H	-18.82980000	1.38410000	-26.51300000
C	-17.83110000	-0.50340000	-26.69300000
C	-17.23390000	-1.37490000	-27.60610000
H	-16.76210000	-2.31180000	-27.31960000
C	-18.88680000	2.39310000	-29.03180000
C	-18.69170000	2.63770000	-30.54770000
H	-19.10130000	3.62690000	-30.84730000
H	-17.61200000	2.63250000	-30.80370000
H	-19.21520000	1.86370000	-31.14960000
C	-18.19380000	3.55340000	-28.28360000
H	-18.36680000	3.50330000	-27.18900000
H	-18.58410000	4.53350000	-28.63290000
H	-17.09820000	3.52950000	-28.46530000
C	-20.40150000	2.42530000	-28.73910000
H	-20.85400000	3.37080000	-29.10940000
H	-20.90990000	1.57420000	-29.24190000
H	-20.60760000	2.36040000	-27.65020000
C	-17.94160000	-0.81570000	-25.18880000
C	-19.42980000	-0.83840000	-24.77590000
H	-19.53850000	-1.12230000	-23.70660000
H	-19.90580000	0.15600000	-24.90310000
H	-19.98980000	-1.57570000	-25.39120000
C	-17.19880000	0.26410000	-24.37270000
H	-17.27790000	0.05600000	-23.28370000
H	-16.12110000	0.27900000	-24.64180000
H	-17.61930000	1.27570000	-24.55050000
C	-17.33070000	-2.18850000	-24.81340000
H	-16.24570000	-2.21890000	-25.05290000
H	-17.84740000	-3.01260000	-25.35190000
H	-17.43580000	-2.38250000	-23.72380000
C	-11.00370000	-0.19280000	-28.64280000
C	-11.50030000	-1.32620000	-27.94810000
C	-11.19820000	1.15380000	-28.05470000
C	-11.41830000	-2.62610000	-28.51340000
C	-12.25270000	-1.16000000	-26.75330000
O	-10.32380000	1.85270000	-27.61700000
O	-12.51050000	1.47210000	-28.12930000
C	-12.12550000	-3.68510000	-27.97350000

H	-10.80490000	-2.77730000	-29.39690000
C	-12.96850000	-2.20740000	-26.21510000
H	-12.31420000	-0.18490000	-26.27520000
C	-12.88860000	2.80920000	-27.89290000
C	-12.92070000	-3.44660000	-26.85510000
H	-12.08300000	-4.67470000	-28.42740000
H	-13.57830000	-2.06780000	-25.32760000
H	-12.01130000	3.43440000	-27.68050000
H	-13.41540000	3.17660000	-28.78490000
C	-13.84700000	2.84590000	-26.71130000
Br	-14.03530000	-4.86780000	-26.15850000
Cl	-12.99410000	2.34760000	-25.21410000
Cl	-15.22480000	1.75840000	-26.99840000
Cl	-14.44100000	4.52030000	-26.52650000

Structure II

O	-9.44340000	-1.73880000	-30.70070000
O	-8.68650000	-1.03820000	-33.12630000
Rh	-10.62560000	-0.03850000	-30.64030000
P	-8.52970000	-1.91740000	-31.91210000
C	-8.17610000	-4.37480000	-31.43960000
C	-6.82120000	-4.60070000	-31.30180000
C	-6.41690000	-5.52320000	-30.28450000
C	-7.40010000	-6.17980000	-29.47770000
C	-6.97770000	-7.16390000	-28.54960000
H	-7.71480000	-7.72230000	-27.98010000
C	-5.64550000	-7.42430000	-28.37400000
C	-4.66140000	-6.71890000	-29.08890000
H	-3.61060000	-6.92050000	-28.90130000
C	-5.05010000	-5.79320000	-30.02110000
H	-4.29060000	-5.25410000	-30.57890000
C	-8.78360000	-5.84170000	-29.62520000
C	-9.14520000	-4.96300000	-30.61850000
H	-10.18030000	-4.64500000	-30.74420000
Cl	-5.14210000	-8.64900000	-27.23490000
O	-8.62610000	-3.46180000	-32.37910000
C	-6.03080000	-2.42080000	-32.13020000
C	-5.88570000	-3.79540000	-32.12320000
C	-4.86530000	-4.34450000	-32.95940000
C	-3.98710000	-3.48130000	-33.68860000
C	-4.20290000	-2.06460000	-33.66900000
C	-5.22860000	-1.56610000	-32.89610000
H	-5.46980000	-0.50320000	-32.89340000
C	-2.90560000	-4.06020000	-34.39970000
H	-2.17820000	-3.42240000	-34.89220000
C	-2.76450000	-5.42040000	-34.46310000
C	-3.68180000	-6.28470000	-33.83820000
H	-3.56670000	-7.36010000	-33.93900000
C	-4.70070000	-5.74570000	-33.09860000
H	-5.40300000	-6.40520000	-32.59690000
Cl	-1.42210000	-6.11370000	-35.34060000
O	-7.01490000	-1.82810000	-31.35430000
C	-9.82950000	-6.34400000	-28.71100000
C	-11.02070000	-6.85240000	-29.22670000
H	-11.08000000	-6.88040000	-30.31350000
C	-12.04010000	-7.30770000	-28.38070000
C	-11.80980000	-7.22430000	-26.99310000
H	-12.59400000	-7.59390000	-26.34110000
C	-10.63530000	-6.69210000	-26.43450000
C	-9.65850000	-6.24800000	-27.33160000
H	-8.72810000	-5.79880000	-26.98960000
C	-13.35790000	-7.91410000	-28.90750000
C	-13.44670000	-7.90330000	-30.45290000
H	-13.38630000	-6.86510000	-30.83940000
H	-14.41170000	-8.33420000	-30.79780000
H	-12.63170000	-8.50920000	-30.90470000
C	-14.55590000	-7.10640000	-28.36490000
H	-14.61580000	-7.14880000	-27.25830000
H	-15.51000000	-7.51840000	-28.75540000
H	-14.47640000	-6.04070000	-28.67160000
C	-13.47300000	-9.38090000	-28.44070000
H	-12.59430000	-9.96650000	-28.78790000
H	-14.39200000	-9.85360000	-28.85050000
H	-13.52620000	-9.45520000	-27.33450000
C	-10.37120000	-6.59220000	-24.91740000
C	-9.10240000	-7.39940000	-24.56370000
H	-9.21730000	-8.45840000	-24.88130000
H	-8.91570000	-7.38110000	-23.46800000
H	-8.20080000	-6.98360000	-25.06060000
C	-11.53420000	-7.14870000	-24.06230000
H	-12.47110000	-6.58230000	-24.24840000
H	-11.30310000	-7.06130000	-22.97840000
H	-11.70660000	-8.22480000	-24.28100000
C	-10.16180000	-5.11300000	-24.52590000
H	-9.27260000	-4.67880000	-25.02870000
H	-10.00630000	-5.01380000	-23.42980000
H	-11.04910000	-4.50800000	-24.80750000
C	-3.38150000	-1.11810000	-34.45440000
C	-2.89740000	0.03720000	-33.84150000
H	-3.14390000	0.13760000	-32.78610000
C	-2.14030000	0.97560000	-34.55090000
C	-1.86170000	0.68680000	-35.89980000
H	-1.24310000	1.39850000	-36.43600000
C	-2.34750000	-0.45300000	-36.56180000
C	-3.12840000	-1.33520000	-35.80710000
H	-3.58070000	-2.22000000	-36.25050000
C	-1.61400000	2.28200000	-33.91790000
C	-2.08390000	2.46720000	-32.45580000
H	-1.71790000	3.43060000	-32.03840000
H	-3.19290000	2.48000000	-32.40760000
H	-1.69740000	1.65430000	-31.80370000
C	-2.12560000	3.49580000	-34.72600000
H	-1.71240000	3.51040000	-35.75560000
H	-1.82430000	4.44880000	-34.23970000
H	-3.23470000	3.47250000	-34.79620000
C	-0.07130000	2.27270000	-33.92190000
H	0.32850000	3.18780000	-33.43340000
H	0.31050000	1.38640000	-33.37020000
H	0.33360000	2.24060000	-34.95490000
C	-2.06780000	-0.76740000	-38.04650000
C	-1.34910000	-2.13040000	-38.15620000
H	-1.09740000	-2.35970000	-39.21440000
H	-0.40650000	-2.11840000	-37.56700000
H	-1.98420000	-2.95920000	-37.77890000
C	-1.17290000	0.29290000	-38.73160000
H	-0.99110000	0.02700000	-39.79570000
H	-1.66000000	1.29190000	-38.71690000
H	-0.18280000	0.35870000	-38.23050000
C	-3.40240000	-0.82730000	-38.82060000
H	-4.05340000	-1.64650000	-38.44990000
H	-3.22290000	-1.00920000	-39.90250000
H	-3.95390000	0.13190000	-38.71410000
O	-11.74990000	1.67800000	-30.77090000

O	-12.31080000	1.05750000	-33.26400000
P	-12.57270000	1.89710000	-32.03860000
C	-12.95060000	4.33670000	-31.49660000
C	-14.30900000	4.57350000	-31.42770000
C	-14.75570000	5.48150000	-30.41320000
C	-13.80730000	6.11040000	-29.54490000
C	-14.26260000	7.08450000	-28.62190000
H	-13.54710000	7.61760000	-28.00280000
C	-15.59750000	7.36520000	-28.51260000
C	-16.55260000	6.68920000	-29.29160000
H	-17.60850000	6.90630000	-29.15800000
C	-16.12950000	5.77040000	-30.21610000
H	-16.86850000	5.25170000	-30.81880000
C	-12.42240000	5.75680000	-29.62460000
C	-12.01860000	4.88920000	-30.61000000
H	-10.98430000	4.55120000	-30.67440000
Cl	-16.14230000	8.57770000	-27.37840000
O	-12.45840000	3.45320000	-32.44520000
C	-15.06930000	2.42250000	-32.36810000
C	-15.20960000	3.79740000	-32.31510000
C	-16.20340000	4.37560000	-33.16610000
C	-17.07370000	3.54110000	-33.93800000
C	-16.86610000	2.12370000	-33.95870000
C	-15.85530000	1.60020000	-33.18390000
H	-15.61380000	0.53850000	-33.21420000
C	-18.13270000	4.14910000	-34.65850000
H	-18.85580000	3.53120000	-35.18210000
C	-18.25550000	5.51190000	-34.69490000
C	-17.34080000	6.35010000	-34.03180000
H	-17.43780000	7.42890000	-34.11330000
C	-16.34730000	5.78150000	-33.28000000
H	-15.64870000	6.42080000	-32.74840000
Cl	-19.56840000	6.24190000	-35.58770000
O	-14.12120000	1.78580000	-31.58520000
C	-11.42020000	6.24620000	-28.65630000
C	-10.22020000	6.78920000	-29.11130000
H	-10.11570000	6.83800000	-30.19430000
C	-9.25010000	7.25250000	-28.21450000
C	-9.53780000	7.13770000	-26.84110000
H	-8.79440000	7.52040000	-26.14960000
C	-10.71970000	6.56360000	-26.34240000
C	-11.64830000	6.11630000	-27.28800000
H	-12.58170000	5.63820000	-26.99690000
C	-7.92390000	7.89670000	-28.67140000
C	-7.76620000	7.91440000	-30.21110000
H	-7.78790000	6.88220000	-30.61570000
H	-6.79540000	8.36910000	-30.50580000
H	-8.57290000	8.51120000	-30.68910000
C	-6.73410000	7.10460000	-28.08860000
H	-5.77150000	7.53540000	-28.43670000
H	-6.72010000	7.13550000	-26.97990000
H	-6.78450000	6.04100000	-28.40840000
C	-7.86160000	9.35730000	-28.17620000
H	-8.73660000	9.93000000	-28.55300000
H	-6.93590000	9.85610000	-28.53700000
H	-7.85910000	9.41410000	-27.06770000
C	-11.04220000	6.42580000	-24.83990000
C	-12.33010000	7.21500000	-24.51830000
H	-12.20930000	8.28310000	-24.80140000
H	-12.56270000	7.16480000	-23.43250000
H	-13.20590000	6.80760000	-25.06600000
C	-9.91610000	6.96680000	-23.92730000

H	-8.96700000	6.41490000	-24.09970000
H	-10.18400000	6.84310000	-22.85550000
H	-9.74840000	8.05120000	-24.10500000
C	-11.25430000	4.93690000	-24.49200000
H	-12.12160000	4.50710000	-25.03510000
H	-11.44550000	4.80830000	-23.40450000
H	-10.35160000	4.35030000	-24.75910000
C	-17.66610000	1.19840000	-34.79060000
C	-18.13890000	0.01280000	-34.22770000
H	-17.91190000	-0.11820000	-33.17140000
C	-18.85650000	-0.91920000	-34.98460000
C	-19.11260000	-0.59130000	-36.32910000
H	-19.69870000	-1.30060000	-36.90380000
C	-18.64280000	0.58300000	-36.94040000
C	-17.89800000	1.45640000	-36.14000000
H	-17.45520000	2.36400000	-36.54510000
C	-19.35980000	-2.26110000	-34.40890000
C	-18.92750000	-2.47970000	-32.93980000
H	-19.27990000	-3.46460000	-32.56270000
H	-17.82070000	-2.46700000	-32.85960000
H	-19.35290000	-1.69690000	-32.27510000
C	-18.78620000	-3.43160000	-35.23900000
H	-19.16150000	-3.41990000	-36.28300000
H	-19.07780000	-4.40900000	-34.79710000
H	-17.67620000	-3.37700000	-35.26700000
C	-20.90140000	-2.29950000	-34.45890000
H	-21.28590000	-3.24070000	-34.00940000
H	-21.32700000	-1.44240000	-33.89330000
H	-21.27730000	-2.24950000	-35.50200000
C	-18.89990000	0.94220000	-38.41910000
C	-19.64650000	2.29230000	-38.49740000
H	-19.88190000	2.55250000	-39.55220000
H	-20.60020000	2.23970000	-37.92870000
H	-19.03780000	3.12130000	-38.07900000
C	-19.75730000	-0.11280000	-39.15810000
H	-19.92580000	0.18670000	-40.21550000
H	-19.24750000	-1.10050000	-39.16960000
H	-20.75450000	-0.21880000	-38.67850000
C	-17.55180000	1.05750000	-39.16310000
H	-16.92690000	1.87750000	-38.75180000
H	-17.71400000	1.27230000	-40.24170000
H	-16.98130000	0.10740000	-39.07770000
O	-9.41130000	1.81140000	-33.06760000
O	-8.89600000	1.09080000	-30.58880000
Rh	-10.48870000	0.01470000	-33.24730000
P	-8.59410000	1.95570000	-31.80740000
C	-8.07580000	4.44220000	-32.09270000
C	-6.70150000	4.56850000	-32.15200000
C	-6.18420000	5.55500000	-33.05200000
C	-7.07010000	6.45220000	-33.72780000
C	-6.51210000	7.51140000	-34.48810000
H	-7.16160000	8.25660000	-34.93660000
C	-5.15740000	7.60360000	-34.66050000
C	-4.28040000	6.65540000	-34.10380000
H	-3.21390000	6.72560000	-34.29770000
C	-4.79420000	5.66420000	-33.31050000
H	-4.12130000	4.93940000	-32.86240000
C	-8.48690000	6.26610000	-33.62320000
C	-8.95590000	5.24550000	-32.82720000
H	-10.01860000	5.01460000	-32.77770000
Cl	-4.48930000	8.91590000	-35.60100000
O	-8.64770000	3.48350000	-31.26880000

C	-6.07960000	2.31730000	-31.33220000
C	-5.85520000	3.68150000	-31.32050000
C	-4.82870000	4.16390000	-30.44480000
C	-4.02550000	3.24420000	-29.70000000
C	-4.33730000	1.84570000	-29.72270000
C	-5.36400000	1.41360000	-30.53350000
H	-5.67040000	0.36800000	-30.55220000
C	-2.91260000	3.74400000	-28.97640000
H	-2.23760000	3.05590000	-28.47780000
C	-2.67050000	5.08910000	-28.91340000
C	-3.51960000	6.01470000	-29.54440000
H	-3.33160000	7.07980000	-29.44210000
C	-4.56820000	5.54980000	-30.29260000
H	-5.21550000	6.26450000	-30.79030000
Cl	-1.28660000	5.68340000	-28.02820000
O	-7.03290000	1.78130000	-32.18210000
C	-9.46490000	7.10220000	-34.35190000
C	-10.58090000	7.59290000	-33.67400000
H	-10.62700000	7.33890000	-32.61650000
C	-11.54730000	8.36590000	-34.32650000
C	-11.32390000	8.65880000	-35.68490000
H	-12.05230000	9.29560000	-36.17540000
C	-10.22860000	8.16380000	-36.41170000
C	-9.31910000	7.36260000	-35.71290000
H	-8.46770000	6.90050000	-36.20830000
C	-12.81660000	8.89370000	-33.62310000
C	-12.94430000	8.38980000	-32.16560000
H	-12.96740000	7.28060000	-32.14330000
H	-13.88470000	8.75510000	-31.69830000
H	-12.10020000	8.75190000	-31.53960000
C	-14.07200000	8.41860000	-34.38910000
H	-14.13300000	8.86700000	-35.40210000
H	-14.99860000	8.70980000	-33.84860000
H	-14.05990000	7.31270000	-34.49870000
C	-12.78480000	10.43550000	-33.59180000
H	-11.87170000	10.79200000	-33.06760000
H	-13.67380000	10.83600000	-33.05780000
H	-12.78730000	10.86370000	-34.61600000
C	-9.99040000	8.45370000	-37.90860000
C	-8.62390000	9.15210000	-38.08540000
H	-8.58850000	10.08850000	-37.48750000
H	-8.44830000	9.41110000	-39.15210000
H	-7.78620000	8.50020000	-37.75990000
C	-11.07160000	9.37200000	-38.52680000
H	-10.86080000	9.56040000	-39.60200000
H	-12.07610000	8.90030000	-38.46250000
H	-11.09420000	10.35780000	-38.01370000
C	-9.99400000	7.12620000	-38.69720000
H	-9.16660000	6.45880000	-38.37750000
H	-9.86800000	7.31390000	-39.78560000
H	-10.95490000	6.58880000	-38.54390000
C	-3.60530000	0.86650000	-28.88800000
C	-3.14480000	-0.32180000	-29.45370000
H	-3.35350000	-0.44250000	-30.51560000
C	-2.44870000	-1.26590000	-28.68860000
C	-2.24310000	-0.95870000	-27.33360000
H	-1.69070000	-1.67020000	-26.72550000
C	-2.71250000	0.21240000	-26.71630000
C	-3.40090000	1.11720000	-27.52910000
H	-3.82120000	2.04950000	-27.16180000
C	-1.90290000	-2.58770000	-29.26690000
C	-2.27520000	-2.78400000	-30.75410000
H	-1.89290000	-3.75540000	-31.13670000
H	-3.37780000	-2.78610000	-30.87390000
H	-1.83860000	-1.98080000	-31.38640000
C	-2.48660000	-3.78060000	-28.47820000
H	-2.14350000	-3.78530000	-27.42300000
H	-2.16790000	-4.74470000	-28.93000000
H	-3.59720000	-3.73840000	-28.48240000
C	-0.36380000	-2.60160000	-29.15850000
H	0.05340000	-3.53010000	-29.60540000
H	0.06870000	-1.72970000	-29.69550000
H	-0.02960000	-2.55950000	-28.10070000
C	-2.46100000	0.45050000	-25.21320000
C	-0.94140000	0.46640000	-24.93870000
H	-0.73650000	0.68980000	-23.86910000
H	-0.47270000	-0.51330000	-25.16710000
H	-0.44550000	1.24400000	-25.55930000
C	-3.12180000	-0.67970000	-24.39510000
H	-2.99190000	-0.50470000	-23.30510000
H	-4.21070000	-0.72850000	-24.61420000
H	-2.67490000	-1.66850000	-24.62900000
C	-3.04200000	1.79600000	-24.71430000
H	-2.58350000	2.65090000	-25.25750000
H	-2.83500000	1.93780000	-23.63130000
H	-4.14460000	1.82620000	-24.84850000
O	-11.55850000	-1.80150000	-33.27160000
O	-12.34820000	-1.15860000	-30.84790000
P	-12.49570000	-2.00590000	-32.10960000
C	-12.96140000	-4.51110000	-32.43630000
C	-14.33000000	-4.64620000	-32.57900000
C	-14.78760000	-5.63870000	-33.50070000
C	-13.86050000	-6.53770000	-34.11630000
C	-14.37230000	-7.59970000	-34.90470000
H	-13.69810000	-8.34790000	-35.30970000
C	-15.71420000	-7.69150000	-35.15970000
C	-16.62460000	-6.74160000	-34.66090000
H	-17.67720000	-6.81210000	-34.92020000
C	-16.16010000	-5.74830000	-33.84090000
H	-16.85720000	-5.02070000	-33.43500000
C	-12.45330000	-6.34870000	-33.92270000
C	-12.03590000	-5.32090000	-33.10570000
H	-10.97680000	-5.09210000	-32.98980000
Cl	-16.32320000	-9.00660000	-36.13560000
O	-12.44460000	-3.54270000	-31.58790000
C	-14.99680000	-2.40100000	-31.80550000
C	-15.22490000	-3.76290000	-31.79500000
C	-16.26080000	-4.24290000	-30.92880000
C	-17.01990000	-3.32030000	-30.14250000
C	-16.67930000	-1.92950000	-30.14620000
C	-15.68450000	-1.49270000	-30.98970000
H	-15.36260000	-0.45120000	-30.99490000
C	-18.11380000	-3.80390000	-29.38170000
H	-18.73830000	-3.10700000	-28.83100000
C	-18.39730000	-5.14200000	-29.34780000
C	-17.60740000	-6.07320000	-30.04360000
H	-17.83200000	-7.13370000	-29.97110000
C	-16.56380000	-5.62300000	-30.80870000
H	-15.95770000	-6.34560000	-31.34500000
Cl	-19.75880000	-5.72050000	-28.41840000
O	-14.01410000	-1.88210000	-32.63390000
C	-11.42880000	-7.18690000	-34.58260000
C	-10.35080000	-7.66050000	-33.83450000
H	-10.36880000	-7.39400000	-32.77960000

C -9.34090000 -8.43200000 -34.41890000
C -9.48120000 -8.74350000 -35.78420000
H -8.72030000 -9.38010000 -36.22280000
C -10.53530000 -8.26600000 -36.58050000
C -11.49040000 -7.46360000 -35.94680000
H -12.31260000 -7.01250000 -36.49840000
C -8.11020000 -8.93780000 -33.63530000
C -8.07190000 -8.41250000 -32.18000000
H -8.06090000 -7.30270000 -32.17160000
H -7.15680000 -8.76180000 -31.65390000
H -8.94720000 -8.77500000 -31.59870000
C -6.81640000 -8.46140000 -34.33360000
H -6.69160000 -8.92460000 -35.33400000
H -5.92050000 -8.73450000 -33.73490000
H -6.83250000 -7.35760000 -34.46120000
C -8.13030000 -10.47930000 -33.58390000
H -9.06900000 -10.83720000 -33.10820000
H -7.27010000 -10.86390000 -32.99390000
H -8.06490000 -10.92130000 -34.60010000
C -10.68180000 -8.57500000 -38.08530000
C -12.03090000 -9.28480000 -38.33510000
H -12.09620000 -10.21430000 -37.72900000
H -12.14070000 -9.55760000 -39.40720000
H -12.89060000 -8.63490000 -38.06830000
C -9.55990000 -9.49300000 -38.62670000
H -8.56400000 -9.01350000 -38.50890000
H -9.70520000 -9.69590000 -39.71020000
H -9.56150000 -10.47230000 -38.10090000
C -10.63920000 -7.25680000 -38.88830000
H -11.48830000 -6.59140000 -38.62690000
H -10.69860000 -7.45810000 -39.98000000
H -9.69260000 -6.71100000 -38.68440000
C -17.33330000 -0.96550000 -29.23740000
C -17.85630000 0.22630000 -29.73280000
H -17.75670000 0.36460000 -30.80830000
C -18.47710000 1.15050000 -28.88360000
C -18.54270000 0.82500000 -27.51570000
H -19.05290000 1.52860000 -26.86670000
C -17.98580000 -0.34470000 -26.97180000
C -17.38210000 -1.22830000 -27.87050000
H -16.91720000 -2.15550000 -27.54040000
C -19.08780000 2.47520000 -29.38850000
C -18.84210000 2.70840000 -30.89850000
H -19.26440000 3.68350000 -31.22540000
H -17.75360000 2.72570000 -31.11300000
H -19.32380000 1.91570000 -31.51100000
C -18.45940000 3.66280000 -28.62650000
H -18.72290000 3.65060000 -27.54900000
H -18.82250000 4.62920000 -29.03760000
H -17.35200000 3.63280000 -28.71400000
C -20.61220000 2.46120000 -29.15290000
H -20.85650000 2.38920000 -28.07230000
H -21.07510000 1.59460000 -29.67290000
H -21.07860000 3.39240000 -29.54160000
C -18.00370000 -0.68960000 -25.46820000
C -18.76070000 -2.01870000 -25.25540000
H -18.82270000 -2.26820000 -24.17390000
H -19.79440000 -1.94300000 -25.65710000
H -18.25160000 -2.86460000 -25.76350000
C -18.69760000 0.39330000 -24.60840000
H -18.68280000 0.11170000 -23.53290000
H -18.17560000 1.36990000 -24.70540000
H -19.76180000 0.51370000 -24.90590000
C -16.55330000 -0.83840000 -24.95710000
H -16.03010000 -1.68160000 -25.45400000
H -16.53820000 -1.03790000 -23.86370000
H -15.97700000 0.09210000 -25.15120000
C -10.88190000 -0.04140000 -28.66820000
C -11.46800000 -1.09090000 -27.91850000
C -10.65420000 1.28520000 -28.04530000
C -11.52200000 -2.41340000 -28.43300000
C -12.11210000 -0.81510000 -26.68350000
O -11.53710000 2.05590000 -27.75470000
O -9.34040000 1.50230000 -27.91430000
C -12.23010000 -3.40310000 -27.77680000
H -10.98610000 -2.64510000 -29.34830000
C -12.85210000 -1.78800000 -26.04540000
H -12.07760000 0.19150000 -26.27420000
C -8.91160000 2.78920000 -27.52170000
C -12.91720000 -3.06070000 -26.61470000
H -12.27100000 -4.41670000 -28.17370000
H -13.38570000 -1.57110000 -25.12510000
H -8.35280000 3.23430000 -28.35700000
H -9.76050000 3.43180000 -27.25280000
C -7.98420000 2.63540000 -26.32770000
Br -14.03490000 -4.38470000 -25.75050000
Cl -6.57340000 1.64020000 -26.76550000
Cl -8.86060000 1.85950000 -24.97320000
Cl -7.44350000 4.26460000 -25.84510000

Structure TS1

O -9.54560000 -1.79070000 -30.79200000
O -8.81580000 -1.10510000 -33.22590000
Rh -10.62890000 -0.01550000 -30.69890000
P -8.61650000 -1.94470000 -31.99150000
C -8.19540000 -4.35960000 -31.43040000
C -6.84330000 -4.54540000 -31.22290000
C -6.46480000 -5.41860000 -30.15070000
C -7.47150000 -6.04570000 -29.34970000
C -7.08450000 -6.97600000 -28.35410000
H -7.84370000 -7.48940000 -27.77090000
C -5.76000000 -7.24060000 -28.13340000
C -4.75300000 -6.57280000 -28.85280000
H -3.70910000 -6.78000000 -28.63370000
C -5.10800000 -5.67890000 -29.83140000
H -4.32790000 -5.17280000 -30.39170000
C -8.85140000 -5.74390000 -29.57440000
C -9.19090000 -4.92840000 -30.62530000
H -10.22890000 -4.64970000 -30.81090000
Cl -5.29480000 -8.40690000 -26.91980000
O -8.61790000 -3.49940000 -32.43000000
C -6.06860000 -2.37260000 -32.09300000
C -5.89000000 -3.74300000 -32.02690000
C -4.80960000 -4.29310000 -32.78370000
C -3.89540000 -3.43200000 -33.46950000
C -4.13440000 -2.01950000 -33.49790000
C -5.22880000 -1.52350000 -32.82440000
H -5.48930000 -0.46610000 -32.87210000
C -2.75900000 -4.00860000 -34.09090000
H -2.00780000 -3.36980000 -34.54490000
C -2.59670000 -5.36770000 -34.11800000
C -3.54670000 -6.23290000 -33.54470000
H -3.41440000 -7.30840000 -33.62110000

C -4.62060000 -5.69450000 -32.88700000
H -5.35140000 -6.35470000 -32.42830000
Cl -1.18760000 -6.05790000 -34.88510000
O -7.11110000 -1.77300000 -31.40540000
C -9.91710000 -6.26270000 -28.69610000
C -10.96800000 -6.99050000 -29.24680000
H -10.91840000 -7.12860000 -30.32540000
C -11.97290000 -7.53000000 -28.43580000
C -11.88180000 -7.28320000 -27.05190000
H -12.63850000 -7.73540000 -26.42090000
C -10.86870000 -6.50220000 -26.46860000
C -9.88440000 -6.00300000 -27.32920000
H -9.05150000 -5.39460000 -26.97140000
C -13.10610000 -8.41490000 -28.99540000
C -13.14420000 -8.42510000 -30.54460000
H -13.24920000 -7.39130000 -30.93900000
H -14.00670000 -9.01900000 -30.91760000
H -12.22490000 -8.88540000 -30.96660000
C -14.47260000 -7.89580000 -28.50240000
H -14.60760000 -8.04170000 -27.41120000
H -15.30540000 -8.43470000 -29.00290000
H -14.56160000 -6.81340000 -28.72300000
C -12.90450000 -9.86320000 -28.50840000
H -11.91920000 -10.25090000 -28.84680000
H -13.69990000 -10.52740000 -28.91100000
H -12.93870000 -9.92340000 -27.39980000
C -10.78030000 -6.19590000 -24.95950000
C -9.45670000 -6.76140000 -24.40050000
H -9.40480000 -7.85910000 -24.56610000
H -9.37270000 -6.56660000 -23.30930000
H -8.57700000 -6.29750000 -24.89140000
C -11.94090000 -6.81480000 -24.14580000
H -12.92160000 -6.42760000 -24.49800000
H -11.84650000 -6.55730000 -23.06830000
H -11.93380000 -7.92340000 -24.22480000
C -10.81880000 -4.66840000 -24.73970000
H -9.98010000 -4.16270000 -25.25630000
H -10.74290000 -4.42240000 -23.65840000
H -11.76890000 -4.24520000 -25.12670000
C -3.26400000 -1.07310000 -34.22860000
C -2.83690000 0.09090000 -33.59030000
H -3.17220000 0.20180000 -32.56030000
C -2.03010000 1.02690000 -34.24620000
C -1.65260000 0.73180000 -35.56960000
H -1.00350000 1.44590000 -36.06470000
C -2.07960000 -0.41620000 -36.25740000
C -2.90590000 -1.30070000 -35.55550000
H -3.31450000 -2.19420000 -36.02330000
C -1.55200000 2.33660000 -33.58210000
C -2.10920000 2.51750000 -32.15050000
H -1.76910000 3.47940000 -31.70850000
H -3.21850000 2.53150000 -32.16940000
H -1.76380000 1.70140000 -31.47950000
C -2.02280000 3.54640000 -34.41990000
H -1.55600000 3.55780000 -35.42630000
H -1.74960000 4.50140000 -33.92100000
H -3.12660000 3.52070000 -34.54800000
C -0.01160000 2.33870000 -33.49230000
H 0.35080000 3.25440000 -32.97650000
H 0.34270000 1.45270000 -32.92210000
H 0.45640000 2.31530000 -34.49840000
C -1.68960000 -0.73750000 -37.71550000
C -0.95180000 -2.09370000 -37.76280000
H -0.62170000 -2.32740000 -38.79820000
H -0.05470000 -2.06830000 -37.10680000
H -1.60450000 -2.92650000 -37.42650000
C -0.75770000 0.32740000 -38.34140000
H -0.49680000 0.05660000 -39.38770000
H -1.25410000 1.32160000 -38.36900000
H 0.19290000 0.40620000 -37.77060000
C -2.96390000 -0.81600000 -38.58410000
H -3.63220000 -1.63910000 -38.25560000
H -2.70430000 -1.00330000 -39.64870000
H -3.53070000 0.13840000 -38.52500000
O -11.58250000 1.82360000 -30.77360000
O -12.29300000 1.24290000 -33.23410000
P -12.48170000 2.06010000 -31.97810000
C -12.88890000 4.52470000 -31.46250000
C -14.25220000 4.73980000 -31.38320000
C -14.70070000 5.70040000 -30.41740000
C -13.75230000 6.41190000 -29.61670000
C -14.21480000 7.42900000 -28.74440000
H -13.50090000 8.02070000 -28.17920000
C -15.55570000 7.66790000 -28.60790000
C -16.50800000 6.91550000 -29.31870000
H -17.56750000 7.10290000 -29.16910000
C -16.07730000 5.96390000 -30.20460000
H -16.81120000 5.40480000 -30.77250000
C -12.36110000 6.08720000 -29.70170000
C -11.95580000 5.15270000 -30.62630000
H -10.91580000 4.83260000 -30.68470000
Cl -16.11040000 8.92550000 -27.52990000
O -12.39130000 3.62000000 -32.38480000
C -14.99590000 2.53010000 -32.19780000
C -15.15710000 3.90380000 -32.20960000
C -16.18060000 4.42470000 -33.06230000
C -17.06210000 3.53900000 -33.75940000
C -16.83630000 2.12480000 -33.71050000
C -15.79610000 1.65380000 -32.94130000
H -15.54220000 0.59450000 -32.92440000
C -18.14540000 4.09460000 -34.48630000
H -18.87500000 3.44080000 -34.95440000
C -18.28050000 5.45190000 -34.60250000
C -17.35390000 6.33500000 -34.01900000
H -17.45940000 7.40600000 -34.16690000
C -16.33660000 5.82030000 -33.26000000
H -15.62650000 6.49630000 -32.79250000
Cl -19.62260000 6.11660000 -35.50260000
O -14.00080000 1.95440000 -31.42350000
C -11.35550000 6.69320000 -28.80450000
C -10.20230000 7.26160000 -29.34010000
H -10.13300000 7.22910000 -30.42600000
C -9.23370000 7.84480000 -28.51590000
C -9.48090000 7.83850000 -27.13000000
H -8.74930000 8.33040000 -26.49790000
C -10.60700000 7.23200000 -26.54880000
C -11.53340000 6.65660000 -27.42370000
H -12.42550000 6.14650000 -27.06140000
C -7.94370000 8.48890000 -29.06650000
C -7.78400000 8.29390000 -30.59420000
H -7.77450000 7.21290000 -30.84720000
H -6.82760000 8.73210000 -30.95390000
H -8.60620000 8.79320000 -31.15110000
C -6.71120000 7.85280000 -28.38640000

H -6.66420000 8.09190000 -27.30420000
H -5.77150000 8.23250000 -28.84170000
H -6.73960000 6.74760000 -28.49720000
C -7.96480000 10.00400000 -28.77890000
H -8.85950000 10.47250000 -29.24330000
H -7.05750000 10.49510000 -29.19290000
H -7.99150000 10.20980000 -27.68810000
C -10.86140000 7.16570000 -25.02870000
C -12.19540000 7.87130000 -24.69950000
H -12.17260000 8.92470000 -25.05370000
H -12.37970000 7.87440000 -23.60330000
H -13.05700000 7.36170000 -25.18000000
C -9.74810000 7.84850000 -24.19930000
H -8.76600000 7.35880000 -24.37630000
H -9.96620000 7.77550000 -23.11160000
H -9.67190000 8.92820000 -24.45200000
C -10.94010000 5.68830000 -24.58510000
H -11.79470000 5.16470000 -25.06140000
H -11.07670000 5.61310000 -23.48450000
H -10.00610000 5.15170000 -24.85960000
C -17.64490000 1.15580000 -34.48280000
C -18.12320000 0.00340000 -33.85920000
H -17.88500000 -0.08120000 -32.80030000
C -18.85990000 -0.95430000 -34.56580000
C -19.11480000 -0.69250000 -35.92490000
H -19.70890000 -1.42410000 -36.46200000
C -18.63470000 0.44370000 -36.59660000
C -17.88150000 1.34950000 -35.84220000
H -17.43570000 2.23470000 -36.29130000
C -19.38700000 -2.25580000 -33.92240000
C -18.97080000 -2.40040000 -32.44030000
H -19.34570000 -3.35540000 -32.01170000
H -17.86560000 -2.40680000 -32.35280000
H -19.38590000 -1.57310000 -31.82530000
C -18.82280000 -3.47680000 -34.68320000
H -19.19090000 -3.51670000 -35.72920000
H -19.12880000 -4.42540000 -34.19090000
H -17.71210000 -3.43660000 -34.70620000
C -20.92890000 -2.27590000 -33.98240000
H -21.33000000 -3.18470000 -33.48320000
H -21.34630000 -1.38230000 -33.46970000
H -21.29760000 -2.28090000 -35.02920000
C -18.89140000 0.73010000 -38.09110000
C -19.62860000 2.07990000 -38.23610000
H -19.86380000 2.28840000 -39.30230000
H -20.58170000 2.06260000 -37.66430000
H -19.01340000 2.92440000 -37.86060000
C -19.75710000 -0.35470000 -38.77510000
H -19.92390000 -0.10830000 -39.84630000
H -19.25490000 -1.34560000 -38.73610000
H -20.75490000 -0.42840000 -38.29060000
C -17.54360000 0.79840000 -38.84140000
H -16.91210000 1.63330000 -38.47210000
H -17.70570000 0.96030000 -39.92920000
H -16.97970000 -0.15040000 -38.70930000
O -9.34120000 1.77110000 -33.09620000
O -8.82090000 1.01390000 -30.63030000
Rh -10.55510000 0.07250000 -33.27400000
P -8.54290000 1.92190000 -31.82210000
C -8.04140000 4.41480000 -32.07320000
C -6.66850000 4.56760000 -32.06670000
C -6.12410000 5.56850000 -32.93200000
C -6.99240000 6.43690000 -33.66660000
C -6.41960000 7.50290000 -34.40550000
H -7.06210000 8.22260000 -34.90340000
C -5.05990000 7.63430000 -34.49420000
C -4.19180000 6.72020000 -33.87020000
H -3.11760000 6.82320000 -33.99500000
C -4.72440000 5.71910000 -33.10210000
H -4.05890000 5.01960000 -32.60480000
C -8.40830000 6.21900000 -33.63760000
C -8.89890000 5.19900000 -32.85410000
H -9.95820000 4.94650000 -32.85560000
Cl -4.37260000 8.95570000 -35.40770000
O -8.63020000 3.44260000 -31.28180000
C -6.06420000 2.30490000 -31.28730000
C -5.85540000 3.66930000 -31.21290000
C -4.91050000 4.13340000 -30.24170000
C -4.19370000 3.19940000 -29.42930000
C -4.48690000 1.80180000 -29.52520000
C -5.40420000 1.38210000 -30.46220000
H -5.66740000 0.32770000 -30.55780000
C -3.18240000 3.68110000 -28.56020000
H -2.58050000 2.98120000 -27.98860000
C -2.94760000 5.02440000 -28.44750000
C -3.70600000 5.96230000 -29.16970000
H -3.52100000 7.02400000 -29.03380000
C -4.66180000 5.51540000 -30.04340000
H -5.24380000 6.24000000 -30.60420000
Cl -1.68950000 5.60150000 -27.38210000
O -6.97120000 1.79480000 -32.19970000
C -9.36120000 7.02150000 -34.43550000
C -10.51290000 7.52160000 -33.82810000
H -10.60910000 7.29880000 -32.76680000
C -11.45490000 8.26100000 -34.55220000
C -11.17900000 8.49350000 -35.91250000
H -11.89740000 9.09120000 -36.46330000
C -10.04460000 7.98820000 -36.56870000
C -9.15320000 7.23580000 -35.79630000
H -8.26930000 6.77370000 -36.23140000
C -12.75180000 8.81740000 -33.92460000
C -12.91500000 8.41860000 -32.43910000
H -12.94090000 7.31450000 -32.34030000
H -13.86660000 8.81430000 -32.02190000
H -12.08480000 8.82390000 -31.82100000
C -13.97700000 8.26850000 -34.68950000
H -13.99990000 8.62250000 -35.74070000
H -14.92290000 8.60300000 -34.21090000
H -13.96080000 7.15700000 -34.69640000
C -12.74360000 10.35860000 -34.00070000
H -11.84690000 10.76550000 -33.48500000
H -13.64910000 10.78130000 -33.51360000
H -12.73300000 10.71590000 -35.05150000
C -9.74560000 8.21720000 -38.06530000
C -8.38640000 8.93610000 -38.21320000
H -8.39620000 9.89860000 -37.65720000
H -8.16820000 9.15140000 -39.28170000
H -7.55170000 8.31740000 -37.82200000
C -10.81580000 9.08410000 -38.77060000
H -10.56100000 9.22940000 -39.84310000
H -11.81290000 8.59460000 -38.72900000
H -10.88020000 10.09090000 -38.30400000
C -9.68830000 6.85660000 -38.79310000
H -8.86340000 6.22150000 -38.40780000

H -9.51730000 6.99900000 -39.88220000
H -10.64460000 6.30610000 -38.65830000
C -3.85210000 0.80290000 -28.63710000
C -3.27100000 -0.33710000 -29.19010000
H -3.31220000 -0.39810000 -30.27620000
C -2.65640000 -1.30080000 -28.38160000
C -2.65980000 -1.06400000 -26.99700000
H -2.15510000 -1.77950000 -26.35360000
C -3.28420000 0.04070000 -26.39380000
C -3.87580000 0.97480000 -27.25150000
H -4.39580000 1.86460000 -26.90360000
C -1.97940000 -2.56610000 -28.94430000
C -2.20510000 -2.73170000 -30.46580000
H -1.75240000 -3.67720000 -30.83610000
H -3.29180000 -2.76560000 -30.69000000
H -1.74000000 -1.89780000 -31.03480000
C -2.55620000 -3.81830000 -28.24820000
H -2.27060000 -3.86810000 -27.17730000
H -2.17840000 -4.74700000 -28.72730000
H -3.66400000 -3.80940000 -28.30980000
C -0.46100000 -2.48990000 -28.68930000
H 0.05230000 -3.38360000 -29.10620000
H -0.03350000 -1.58360000 -29.17060000
H -0.23560000 -2.44510000 -27.60280000
C -3.30220000 0.16980000 -24.85670000
C -1.85410000 0.27270000 -24.33230000
H -1.84380000 0.41820000 -23.23020000
H -1.27360000 -0.64760000 -24.55120000
H -1.33320000 1.13430000 -24.80350000
C -3.98750000 -1.06900000 -24.23580000
H -4.03850000 -0.97710000 -23.12930000
H -5.02310000 -1.17530000 -24.62110000
H -3.43550000 -2.00410000 -24.46480000
C -4.07020000 1.42330000 -24.37340000
H -3.59440000 2.35180000 -24.75690000
H -4.07040000 1.48200000 -23.26320000
H -5.13000000 1.39070000 -24.70710000
O -11.76520000 -1.64840000 -33.32930000
O -12.42530000 -1.00900000 -30.87200000
P -12.59350000 -1.90350000 -32.09170000
C -12.91680000 -4.45780000 -32.33930000
C -14.28260000 -4.67910000 -32.36690000
C -14.74970000 -5.71740000 -33.23270000
C -13.82340000 -6.59100000 -33.88430000
C -14.33260000 -7.69510000 -34.61430000
H -13.65050000 -8.42220000 -35.04400000
C -15.68190000 -7.85150000 -34.78350000
C -16.60220000 -6.92970000 -34.25100000
H -17.66440000 -7.05180000 -34.44320000
C -16.13400000 -5.89660000 -33.48390000
H -16.83730000 -5.19080000 -33.05110000
C -12.41810000 -6.33480000 -33.78250000
C -12.00080000 -5.25160000 -33.04160000
H -10.94920000 -4.96860000 -33.00890000
Cl -16.28950000 -9.21640000 -35.69020000
O -12.38670000 -3.43190000 -31.57530000
C -15.01240000 -2.46810000 -31.55710000
C -15.16440000 -3.83980000 -31.52270000
C -16.11190000 -4.36690000 -30.58460000
C -16.86020000 -3.47930000 -29.74950000
C -16.58730000 -2.07490000 -29.77610000
C -15.67410000 -1.59310000 -30.68620000
H -15.41660000 -0.53490000 -30.72460000
C -17.88490000 -4.00950000 -28.92640000
H -18.50370000 -3.34190000 -28.33510000
C -18.11270000 -5.35730000 -28.88420000
C -17.33150000 -6.25270000 -29.63340000
H -17.51380000 -7.32090000 -29.55780000
C -16.35360000 -5.75790000 -30.45580000
H -15.75090000 -6.45530000 -31.02830000
Cl -19.38800000 -5.99490000 -27.87350000
O -14.15340000 -1.90500000 -32.49100000
C -11.39580000 -7.16890000 -34.45130000
C -10.28850000 -7.59890000 -33.72030000
H -10.28030000 -7.29290000 -32.67550000
C -9.28350000 -8.37460000 -34.30790000
C -9.45110000 -8.72120000 -35.66170000
H -8.68650000 -9.35010000 -36.10510000
C -10.53710000 -8.28930000 -36.44100000
C -11.49400000 -7.49220000 -35.80270000
H -12.34600000 -7.08100000 -36.34040000
C -8.03250000 -8.85270000 -33.53890000
C -7.98620000 -8.32350000 -32.08560000
H -7.98520000 -7.21450000 -32.08030000
H -7.06250000 -8.66170000 -31.56720000
H -8.85340000 -8.69250000 -31.49630000
C -6.75890000 -8.35210000 -34.25630000
H -6.64950000 -8.80160000 -35.26480000
H -5.84870000 -8.62150000 -33.67790000
H -6.78970000 -7.24680000 -34.36940000
C -8.01970000 -10.39440000 -33.48060000
H -8.94770000 -10.76980000 -32.99720000
H -7.14810000 -10.75840000 -32.89440000
H -7.95020000 -10.84090000 -34.49450000
C -10.71780000 -8.64400000 -37.93210000
C -12.05380000 -9.39550000 -38.12320000
H -12.07680000 -10.30920000 -37.49060000
H -12.18670000 -9.70070000 -39.18380000
H -12.92280000 -8.76110000 -37.84900000
C -9.58740000 -9.54700000 -38.48090000
H -8.60170000 -9.03900000 -38.40390000
H -9.75720000 -9.78280000 -39.55400000
H -9.54850000 -10.51160000 -37.92980000
C -10.73350000 -7.34810000 -38.77150000
H -11.59230000 -6.69790000 -38.50330000
H -10.81880000 -7.58120000 -39.85510000
H -9.79640000 -6.77240000 -38.61040000
C -17.28750000 -1.12750000 -28.88530000
C -17.89850000 -0.00710000 -29.43800000
H -17.82300000 0.09810000 -30.51980000
C -18.58050000 0.92580000 -28.65010000
C -18.62760000 0.67140000 -27.26960000
H -19.14330000 1.36380000 -26.61300000
C -18.02570000 -0.45170000 -26.66690000
C -17.33550000 -1.33470000 -27.50590000
H -16.80290000 -2.21600000 -27.15030000
C -19.24430000 2.14340000 -29.32470000
C -18.17070000 2.97210000 -30.05840000
H -18.62670000 3.86760000 -30.53280000
H -17.38410000 3.30550000 -29.34700000
H -17.68490000 2.38470000 -30.86240000
C -19.95480000 3.07960000 -28.31910000
H -19.23230000 3.48430000 -27.57730000
H -20.76890000 2.54300000 -27.78560000

H -20.41390000 3.94570000 -28.84380000
C -20.29700000 1.65680000 -30.34520000
H -20.82380000 2.51850000 -30.80950000
H -19.83110000 1.07280000 -31.16670000
H -21.05220000 1.01340000 -29.84410000
C -18.15620000 -0.67150000 -25.14390000
C -19.64990000 -0.72840000 -24.75200000
H -19.76590000 -0.95810000 -23.67050000
H -20.16250000 0.23820000 -24.93660000
H -20.17160000 -1.51850000 -25.33430000
C -17.46970000 0.48880000 -24.39270000
H -17.54440000 0.34320000 -23.29320000
H -16.39300000 0.54100000 -24.66260000
H -17.93760000 1.46590000 -24.63410000
C -17.50400000 -1.99360000 -24.67150000
H -16.41230000 -1.99050000 -24.86890000
H -17.96520000 -2.86680000 -25.18220000
H -17.64000000 -2.13230000 -23.57690000
C -10.81370000 -0.21230000 -28.45780000
C -11.66620000 -1.31150000 -27.96970000
C -11.12250000 1.19760000 -28.11350000
H -9.69260000 -0.45070000 -28.59940000
C -11.62170000 -2.59160000 -28.54930000
C -12.58290000 -1.09730000 -26.92850000
O -10.33680000 2.08120000 -27.85270000
O -12.45980000 1.39130000 -28.21270000
C -8.80700000 -0.33340000 -27.45220000
C -12.51270000 -3.59110000 -28.17460000
H -10.90910000 -2.79600000 -29.34540000
C -13.49310000 -2.07390000 -26.55710000
H -12.62110000 -0.13950000 -26.41770000
C -12.94640000 2.70640000 -28.11190000
C -7.61520000 -0.77130000 -28.25800000
C -8.88010000 -1.39230000 -26.37640000
H -8.91350000 0.72640000 -27.22100000
C -13.46370000 -3.30020000 -27.20460000
H -12.47460000 -4.57120000 -28.65270000
H -14.21630000 -1.87370000 -25.77280000
H -12.13200000 3.41860000 -27.92520000
H -13.46410000 2.95850000 -29.04740000
C -13.95640000 2.77020000 -26.97600000
C -6.63870000 -0.93470000 -27.05350000
C -7.71080000 -2.31350000 -28.23330000
H -7.37040000 -0.19560000 -29.15100000
C -7.56290000 -2.13330000 -26.70440000
H -9.76200000 -2.04390000 -26.47340000
H -8.90790000 -0.93340000 -25.38070000
Br -14.80510000 -4.62840000 -26.70960000
Cl -13.14860000 2.41430000 -25.41260000
Cl -15.27440000 1.60740000 -27.24420000
Cl -14.62600000 4.42700000 -26.92550000
H -6.55590000 -0.11490000 -26.32860000
H -5.64280000 -1.24790000 -27.38910000
H -8.62280000 -2.79080000 -28.61650000
H -6.82900000 -2.76100000 -28.70760000
C -7.07760000 -3.20860000 -25.79060000
C -7.43640000 -3.24070000 -24.44040000
C -6.17080000 -4.16530000 -26.25710000
C -6.91490000 -4.19690000 -23.57540000
H -8.14030000 -2.51050000 -24.04630000
C -5.63130000 -5.12230000 -25.40690000
H -5.87480000 -4.16970000 -27.30600000
C -6.01490000 -5.12640000 -24.07420000
H -7.20930000 -4.22050000 -22.53000000
H -4.92570000 -5.86120000 -25.77710000
Br -5.28900000 -6.49260000 -22.88620000

Structure TS2

O	-9.56380000	-1.87320000	-30.71200000
O	-8.76550000	-1.16140000	-33.11700000
Rh	-10.66010000	-0.09560000	-30.67720000
P	-8.62450000	-2.04170000	-31.90220000
C	-8.22830000	-4.50460000	-31.47410000
C	-6.87160000	-4.74700000	-31.38140000
C	-6.45720000	-5.76250000	-30.45710000
C	-7.43350000	-6.44710000	-29.66470000
C	-7.03620000	-7.55740000	-28.87900000
H	-7.78450000	-8.11740000	-28.32460000
C	-5.72490000	-7.95220000	-28.85330000
C	-4.73230000	-7.21510000	-29.52690000
H	-3.69020000	-7.51020000	-29.43850000
C	-5.09890000	-6.13970000	-30.29650000
H	-4.33020000	-5.58290000	-30.82370000
C	-8.80410000	-6.04380000	-29.71790000
C	-9.18320000	-5.10600000	-30.64640000
H	-10.21820000	-4.77200000	-30.72700000
Cl	-5.26080000	-9.38590000	-27.97840000
O	-8.69520000	-3.57800000	-32.38880000
C	-6.11290000	-2.51140000	-32.09190000
C	-5.95440000	-3.88480000	-32.16470000
C	-4.93630000	-4.37170000	-33.04260000
C	-4.04820000	-3.46100000	-33.69760000
C	-4.26360000	-2.04970000	-33.57900000
C	-5.30710000	-1.60770000	-32.79560000
H	-5.55570000	-0.54860000	-32.73630000
C	-2.96540000	-3.99060000	-34.44460000
H	-2.22730000	-3.32210000	-34.87650000
C	-2.84100000	-5.34180000	-34.62490000
C	-3.77880000	-6.24380000	-34.08970000
H	-3.68340000	-7.30700000	-34.29100000
C	-4.79360000	-5.75710000	-33.30960000
H	-5.51320000	-6.44720000	-32.87910000
Cl	-1.49930000	-5.97410000	-35.54740000
O	-7.11770000	-1.96470000	-31.30110000
C	-9.82580000	-6.57820000	-28.79790000
C	-10.99310000	-7.13550000	-29.31310000
H	-11.04430000	-7.17640000	-30.39990000
C	-11.99490000	-7.62140000	-28.46550000
C	-11.77750000	-7.49650000	-27.07900000
H	-12.54780000	-7.88840000	-26.42380000
C	-10.63370000	-6.89560000	-26.52440000
C	-9.65700000	-6.44990000	-27.42230000
H	-8.73730000	-5.96700000	-27.08840000
C	-13.27570000	-8.30140000	-28.99310000
C	-13.37820000	-8.26790000	-30.53860000
H	-13.36600000	-7.22050000	-30.90930000
H	-14.32630000	-8.73490000	-30.88370000
H	-12.54230000	-8.83070000	-31.00730000
C	-14.52060000	-7.58430000	-28.43120000
H	-14.59280000	-7.67990000	-27.32870000
H	-15.44810000	-8.02640000	-28.85260000
H	-14.49050000	-6.50440000	-28.69080000

C	-13.29000000	-9.77780000	-28.54670000
H	-12.38510000	-10.30320000	-28.92170000
H	-14.18760000	-10.30010000	-28.94340000
H	-13.31030000	-9.86710000	-27.44010000
C	-10.41720000	-6.69070000	-25.01080000
C	-9.11300000	-7.39490000	-24.57490000
H	-9.15630000	-8.47690000	-24.82520000
H	-8.95760000	-7.29450000	-23.47880000
H	-8.22410000	-6.95790000	-25.07510000
C	-11.57020000	-7.25940000	-24.15100000
H	-12.53130000	-6.75750000	-24.39350000
H	-11.37290000	-7.09400000	-23.06940000
H	-11.67740000	-8.35440000	-24.30830000
C	-10.30750000	-5.18070000	-24.71180000
H	-9.44760000	-4.72820000	-25.24380000
H	-10.16190000	-5.00070000	-23.62470000
H	-11.22980000	-4.65210000	-25.03430000
C	-3.41910000	-1.05210000	-34.27090000
C	-2.92900000	0.03980000	-33.55510000
H	-3.20620000	0.06330000	-32.50250000
C	-2.12820000	1.01080000	-34.16610000
C	-1.81980000	0.82370000	-35.52630000
H	-1.16660000	1.55750000	-35.98600000
C	-2.31820000	-0.24320000	-36.29200000
C	-3.13750000	-1.16460000	-35.63080000
H	-3.59670000	-2.00040000	-36.15500000
C	-1.58210000	2.24440000	-33.41520000
C	-2.09460000	2.33080000	-31.95820000
H	-1.71330000	3.24570000	-31.45440000
H	-3.20310000	2.37730000	-31.94400000
H	-1.75560000	1.45690000	-31.36070000
C	-2.02660000	3.53450000	-34.14050000
H	-1.56980000	3.62080000	-35.14790000
H	-1.72110000	4.43570000	-33.56610000
H	-3.13170000	3.54870000	-34.25760000
C	-0.04130000	2.18160000	-33.37250000
H	0.37140000	3.04280000	-32.80330000
H	0.29290000	1.24340000	-32.87880000
H	0.39620000	2.21310000	-34.39220000
C	-2.01040000	-0.43970000	-37.79140000
C	-1.31980000	-1.80570000	-37.99900000
H	-1.04820000	-1.95210000	-39.06700000
H	-0.39160000	-1.86560000	-37.39050000
H	-1.98210000	-2.64740000	-37.70630000
C	-1.07630000	0.65210000	-38.36590000
H	-0.87720000	0.47260000	-39.44490000
H	-1.54030000	1.65820000	-38.27620000
H	-0.09610000	0.65070000	-37.84170000
C	-3.32740000	-0.40260000	-38.59640000
H	-4.00520000	-1.23350000	-38.30930000
H	-3.12660000	-0.49870000	-39.68560000
H	-3.85940000	0.55770000	-38.42230000
O	-11.61400000	1.73660000	-30.78580000
O	-12.26090000	1.15270000	-33.26360000
P	-12.47670000	1.97580000	-32.01650000
C	-12.87370000	4.44670000	-31.50330000
C	-14.23870000	4.65800000	-31.44640000
C	-14.70830000	5.61730000	-30.49150000
C	-13.77530000	6.35850000	-29.69990000
C	-14.26050000	7.39270000	-28.86060000
H	-13.56150000	8.01270000	-28.30740000
C	-15.60590000	7.61380000	-28.74100000
C	-16.54230000	6.82150000	-29.42960000
H	-17.60500000	6.99280000	-29.28380000
C	-16.09200000	5.85180000	-30.28600000
H	-16.81410000	5.24650000	-30.82510000
C	-12.37880000	6.04610000	-29.76360000
C	-11.95550000	5.09390000	-30.66350000
H	-10.91080000	4.78470000	-30.70520000
Cl	-16.18810000	8.89390000	-27.70470000
O	-12.36170000	3.53710000	-32.41340000
C	-14.97990000	2.46370000	-32.30280000
C	-15.13060000	3.83760000	-32.30040000
C	-16.13000000	4.37940000	-33.16900000
C	-17.00100000	3.50980000	-33.89890000
C	-16.78640000	2.09340000	-33.86420000
C	-15.76710000	1.60380000	-33.07840000
H	-15.52010000	0.54290000	-33.07090000
C	-18.06480000	4.08300000	-34.64080000
H	-18.78760000	3.44080000	-35.13480000
C	-18.19010000	5.44260000	-34.73800000
C	-17.27210000	6.31090000	-34.11980000
H	-17.36920000	7.38480000	-34.25230000
C	-16.27430000	5.77880000	-33.34710000
H	-15.57120000	6.44420000	-32.85450000
Cl	-19.50800000	6.12970000	-35.65670000
O	-14.01140000	1.87350000	-31.50640000
C	-11.39200000	6.69000000	-28.86940000
C	-10.23240000	7.25170000	-29.39960000
H	-10.13400000	7.17610000	-30.48140000
C	-9.29280000	7.88370000	-28.57630000
C	-9.57870000	7.94030000	-27.19940000
H	-8.87230000	8.47370000	-26.57240000
C	-10.71090000	7.34400000	-26.62080000
C	-11.60140000	6.70940000	-27.49190000
H	-12.49160000	6.19880000	-27.12750000
C	-7.99250000	8.51560000	-29.11700000
C	-7.78230000	8.24440000	-30.62550000
H	-7.75690000	7.15180000	-30.81700000
H	-6.81830000	8.67180000	-30.97770000
H	-8.59010000	8.70560000	-31.23390000
C	-6.77540000	7.92930000	-28.36630000
H	-6.75640000	8.23930000	-27.30130000
H	-5.82340000	8.27860000	-28.82140000
H	-6.80020000	6.81920000	-28.40370000
C	-8.03880000	10.04290000	-28.90890000
H	-8.92180000	10.47640000	-29.42650000
H	-7.12290000	10.52280000	-29.31710000
H	-8.10490000	10.30400000	-27.83180000
C	-11.01160000	7.35600000	-25.10780000
C	-12.36670000	8.05440000	-24.85900000
H	-12.35000000	9.08710000	-25.26980000
H	-12.58570000	8.11390000	-23.77080000
H	-13.20440000	7.50400000	-25.33690000
C	-9.93640000	8.10320000	-24.28370000
H	-8.94140000	7.62210000	-24.40260000
H	-10.18790000	8.08640000	-23.20080000
H	-9.86960000	9.16860000	-24.59340000
C	-11.08000000	5.90430000	-24.58590000
H	-11.91310000	5.34280000	-25.05610000
H	-11.24680000	5.88690000	-23.48710000
H	-10.13070000	5.36900000	-24.80500000
C	-17.58860000	1.13930000	-34.66130000
C	-18.08410000	-0.01520000	-34.05530000

H	-17.86450000	-0.10980000	-32.99340000
C	-18.81720000	-0.96100000	-34.78100000
C	-19.05490000	-0.68270000	-36.13990000
H	-19.65070000	-1.40250000	-36.69080000
C	-18.55660000	0.45580000	-36.79430000
C	-17.80480000	1.34770000	-36.02200000
H	-17.34540000	2.23300000	-36.45710000
C	-19.35930000	-2.26580000	-34.15730000
C	-18.94780000	-2.43500000	-32.67620000
H	-19.33110000	-3.39310000	-32.26230000
H	-17.84260000	-2.45070000	-32.58670000
H	-19.35640000	-1.61330000	-32.04900000
C	-18.80640000	-3.48250000	-34.93330000
H	-19.17320000	-3.50590000	-35.98010000
H	-19.12380000	-4.43410000	-34.45410000
H	-17.69530000	-3.45380000	-34.95410000
C	-20.90090000	-2.26890000	-34.22230000
H	-21.31290000	-3.18150000	-33.73940000
H	-21.31080000	-1.37950000	-33.69660000
H	-21.26540000	-2.25260000	-35.27060000
C	-18.79250000	0.75830000	-38.28910000
C	-19.51610000	2.11580000	-38.43030000
H	-19.73620000	2.33640000	-39.49740000
H	-20.47630000	2.10150000	-37.87060000
H	-18.89830000	2.95120000	-38.03910000
C	-19.65910000	-0.31240000	-38.99410000
H	-19.81000000	-0.05470000	-40.06500000
H	-19.16620000	-1.30810000	-38.95800000
H	-20.66360000	-0.38160000	-38.52310000
C	-17.43500000	0.82170000	-39.02220000
H	-16.80090000	1.64750000	-38.63720000
H	-17.58230000	0.99530000	-40.11030000
H	-16.88110000	-0.13320000	-38.89220000
O	-9.33360000	1.69400000	-33.03210000
O	-8.84510000	0.90810000	-30.56490000
Rh	-10.51770000	-0.01720000	-33.24580000
P	-8.55310000	1.82850000	-31.74560000
C	-8.03190000	4.31860000	-31.95170000
C	-6.65960000	4.46810000	-31.89290000
C	-6.08260000	5.48010000	-32.72390000
C	-6.92190000	6.34660000	-33.49460000
C	-6.32360000	7.42130000	-34.19950000
H	-6.94790000	8.13810000	-34.72410000
C	-4.96280000	7.56700000	-34.21910000
C	-4.11800000	6.65900000	-33.55570000
H	-3.04010000	6.77520000	-33.62340000
C	-4.67810000	5.64700000	-32.82210000
H	-4.03100000	4.95210000	-32.29520000
C	-8.33690000	6.12320000	-33.52850000
C	-8.85860000	5.10370000	-32.76470000
H	-9.91750000	4.85160000	-32.81020000
Cl	-4.24410000	8.90040000	-35.09000000
O	-8.65050000	3.34220000	-31.18790000
C	-6.09310000	2.18380000	-31.15410000
C	-5.87830000	3.54380000	-31.03590000
C	-4.95730000	3.96930000	-30.02590000
C	-4.25100000	3.00520000	-29.23860000
C	-4.54200000	1.61180000	-29.39220000
C	-5.46130000	1.23120000	-30.34320000
H	-5.74490000	0.18540000	-30.46700000
C	-3.25910000	3.45780000	-28.33250000
H	-2.66080000	2.73880000	-27.78080000
C	-3.04170000	4.79740000	-28.15500000
C	-3.79460000	5.76050000	-28.85010000
H	-3.62540000	6.81680000	-28.66190000
C	-4.72440000	5.34330000	-29.76530000
H	-5.29830000	6.08430000	-30.31330000
Cl	-1.81090000	5.33810000	-27.03930000
O	-6.97960000	1.70530000	-32.10280000
C	-9.25610000	6.92440000	-34.36560000
C	-10.43420000	7.42180000	-33.80840000
H	-10.57670000	7.19670000	-32.75260000
C	-11.34460000	8.16110000	-34.57200000
C	-11.01060000	8.39460000	-35.91910000
H	-11.70640000	8.98970000	-36.50080000
C	-9.84650000	7.89370000	-36.52510000
C	-8.98840000	7.14210000	-35.71530000
H	-8.08450000	6.68350000	-36.11130000
C	-12.66680000	8.71790000	-33.99980000
C	-12.88850000	8.32710000	-32.52000000
H	-12.92010000	7.22360000	-32.41670000
H	-13.85520000	8.72590000	-32.14240000
H	-12.08250000	8.73460000	-31.87190000
C	-13.85910000	8.16160000	-34.80980000
H	-13.83650000	8.50390000	-35.86500000
H	-14.82380000	8.50230000	-34.37510000
H	-13.84500000	7.05000000	-34.80330000
C	-12.65910000	10.25880000	-34.08360000
H	-11.78350000	10.67040000	-33.53640000
H	-13.58330000	10.68180000	-33.63340000
H	-12.60990000	10.61100000	-35.13500000
C	-9.48030000	8.12810000	-38.00580000
C	-8.11800000	8.85170000	-38.08950000
H	-8.15620000	9.81240000	-37.53180000
H	-7.85200000	9.07090000	-39.14640000
H	-7.29990000	8.23430000	-37.66250000
C	-10.51990000	8.99380000	-38.75660000
H	-10.21700000	9.14330000	-39.81580000
H	-11.51630000	8.50100000	-38.76190000
H	-10.60890000	9.99890000	-38.29020000
C	-9.38540000	6.76980000	-38.73420000
H	-8.57670000	6.13630000	-38.31380000
H	-9.16550000	6.91610000	-39.81400000
H	-10.34490000	6.21590000	-38.64460000
C	-3.90090000	0.56460000	-28.56670000
C	-3.33420000	-0.54180000	-29.19820000
H	-3.38400000	-0.52900000	-30.28570000
C	-2.73570000	-1.56980000	-28.46200000
C	-2.73290000	-1.42860000	-27.06450000
H	-2.26400000	-2.21030000	-26.47290000
C	-3.30300000	-0.33810000	-26.38590000
C	-3.89840000	0.65340000	-27.17380000
H	-4.39240000	1.53150000	-26.76340000
C	-2.09620000	-2.81310000	-29.11310000
C	-2.22200000	-2.81030000	-30.65470000
H	-1.76890000	-3.72610000	-31.09310000
H	-3.29060000	-2.79190000	-30.95200000
H	-1.70220000	-1.93320000	-31.09740000
C	-2.79980000	-4.08280000	-28.59250000
H	-2.66220000	-4.21490000	-27.49930000
H	-2.39680000	-4.99150000	-29.08970000
H	-3.88780000	-4.01830000	-28.80250000
C	-0.59540000	-2.87110000	-28.76000000
H	-0.10760000	-3.73750000	-29.25720000

H	-0.08440000	-1.94240000	-29.09470000
H	-0.43700000	-2.98170000	-27.66690000
C	-3.24250000	-0.27100000	-24.84580000
C	-1.76900000	-0.28600000	-24.38390000
H	-1.70100000	-0.18020000	-23.27940000
H	-1.26320000	-1.23570000	-24.65600000
H	-1.20970000	0.55390000	-24.85030000
C	-3.98260000	-1.48470000	-24.24660000
H	-3.97360000	-1.44400000	-23.13590000
H	-5.03830000	-1.48830000	-24.58540000
H	-3.51550000	-2.44480000	-24.55040000
C	-3.90010000	1.00970000	-24.27770000
H	-3.38930000	1.91980000	-24.66080000
H	-3.83250000	1.02870000	-23.16810000
H	-4.97760000	1.05510000	-24.54590000
O	-11.70670000	-1.75250000	-33.31470000
O	-12.44410000	-1.10690000	-30.87810000
P	-12.59110000	-1.98610000	-32.11230000
C	-12.99570000	-4.51870000	-32.39460000
C	-14.36710000	-4.69320000	-32.45660000
C	-14.85250000	-5.70560000	-33.34170000
C	-13.94110000	-6.58550000	-34.00430000
C	-14.46970000	-7.66440000	-34.75840000
H	-13.80100000	-8.39760000	-35.19840000
C	-15.82090000	-7.79020000	-34.93680000
C	-16.72470000	-6.85980000	-34.39150000
H	-17.78820000	-6.95660000	-34.59050000
C	-16.23890000	-5.85090000	-33.60350000
H	-16.92980000	-5.13830000	-33.16230000
C	-12.53060000	-6.36140000	-33.89110000
C	-12.09060000	-5.31860000	-33.10540000
H	-11.03140000	-5.06670000	-33.04920000
Cl	-16.45150000	-9.12560000	-35.87120000
O	-12.45470000	-3.52210000	-31.59730000
C	-15.04770000	-2.45700000	-31.67850000
C	-15.24180000	-3.82290000	-31.63680000
C	-16.22580000	-4.31360000	-30.71830000
C	-16.97150000	-3.39640000	-29.91300000
C	-16.64840000	-2.00100000	-29.93510000
C	-15.70150000	-1.55500000	-30.82900000
H	-15.39770000	-0.50830000	-30.85800000
C	-18.03650000	-3.89230000	-29.11940000
H	-18.66230000	-3.20260000	-28.56180000
C	-18.29370000	-5.23470000	-29.06290000
C	-17.50310000	-6.15950000	-29.76580000
H	-17.70590000	-7.22270000	-29.67350000
C	-16.49460000	-5.69810000	-30.57010000
H	-15.88870000	-6.41520000	-31.11450000
Cl	-19.62210000	-5.82680000	-28.09450000
O	-14.13290000	-1.93170000	-32.57950000
C	-11.53480000	-7.18390000	-34.61220000
C	-10.42150000	-7.67110000	-33.92860000
H	-10.38360000	-7.41760000	-32.87070000
C	-9.44890000	-8.44120000	-34.57630000
C	-9.66070000	-8.73010000	-35.93720000
H	-8.92630000	-9.36240000	-36.42450000
C	-10.75310000	-8.23730000	-36.67000000
C	-11.67040000	-7.44210000	-35.97490000
H	-12.52290000	-6.98550000	-36.47350000
C	-8.18570000	-8.97210000	-33.86460000
C	-8.07440000	-8.47830000	-32.40290000
H	-8.05680000	-7.36920000	-32.37390000
H	-7.13720000	-8.84320000	-31.92870000
H	-8.92350000	-8.84740000	-31.78790000
C	-6.92370000	-8.48940000	-34.61480000
H	-6.85040000	-8.93400000	-35.62870000
H	-6.00170000	-8.77830000	-34.06510000
H	-6.93860000	-7.38320000	-34.72110000
C	-8.21470000	-10.51420000	-33.84340000
H	-9.13280000	-10.87580000	-33.33140000
H	-7.33070000	-10.91620000	-33.30220000
H	-8.20000000	-10.93600000	-34.87010000
C	-10.98180000	-8.52650000	-38.16850000
C	-12.34300000	-9.23310000	-38.35330000
H	-12.37570000	-10.17050000	-37.75680000
H	-12.51150000	-9.49180000	-39.42120000
H	-13.18640000	-8.58680000	-38.03130000
C	-9.89170000	-9.43710000	-38.78230000
H	-8.89030000	-8.95990000	-38.71110000
H	-10.09520000	-9.62450000	-39.85920000
H	-9.86630000	-10.42400000	-38.27130000
C	-10.98230000	-7.19790000	-38.95520000
H	-11.81530000	-6.53600000	-38.63870000
H	-11.10170000	-7.38480000	-40.04450000
H	-10.02550000	-6.65490000	-38.79630000
C	-17.28670000	-1.03490000	-29.01750000
C	-17.78500000	0.16600000	-29.51900000
H	-17.68750000	0.29380000	-30.59550000
C	-18.37060000	1.11580000	-28.67560000
C	-18.46190000	0.78150000	-27.31290000
H	-18.93400000	1.49410000	-26.64160000
C	-17.97030000	-0.41710000	-26.76440000
C	-17.35450000	-1.30730000	-27.65040000
H	-16.89200000	-2.24150000	-27.34040000
C	-18.90490000	2.47310000	-29.17780000
C	-18.68710000	2.67650000	-30.69680000
H	-19.06490000	3.67000000	-31.02280000
H	-17.60580000	2.63370000	-30.94340000
H	-19.22720000	1.90360000	-31.28540000
C	-18.17580000	3.62050000	-28.44590000
H	-18.34810000	3.58790000	-27.35040000
H	-18.53800000	4.60660000	-28.80730000
H	-17.08150000	3.56020000	-28.62930000
C	-20.41990000	2.56550000	-28.90050000
H	-20.83560000	3.51670000	-29.29840000
H	-20.95340000	1.72040000	-29.38700000
H	-20.63810000	2.53580000	-27.81250000
C	-18.14120000	-0.71150000	-25.25890000
C	-19.64260000	-0.68970000	-24.89710000
H	-19.79550000	-0.96160000	-23.83010000
H	-20.08590000	0.31660000	-25.04750000
H	-20.20180000	-1.41600000	-25.52590000
C	-17.39710000	0.35580000	-24.42880000
H	-17.52130000	0.16320000	-23.34110000
H	-16.31050000	0.33660000	-24.65890000
H	-17.78160000	1.37650000	-24.63440000
C	-17.58250000	-2.09750000	-24.85420000
H	-16.49060000	-2.15800000	-25.05070000
H	-18.09860000	-2.91000000	-25.41050000
H	-17.73500000	-2.28300000	-23.76870000
C	-10.88310000	-0.18150000	-28.45820000
C	-11.69430000	-1.27780000	-27.90250000
C	-11.18560000	1.23250000	-28.12990000
H	-9.75710000	-0.40820000	-28.61090000

C	-11.62310000	-2.57520000	-28.43880000
C	-12.54410000	-1.06020000	-26.80700000
O	-10.39700000	2.11620000	-27.88860000
O	-12.52600000	1.41610000	-28.21070000
C	-8.86890000	-0.23970000	-27.54020000
C	-12.38290000	-3.61540000	-27.91680000
H	-10.97230000	-2.77020000	-29.28780000
C	-13.32390000	-2.08190000	-26.28730000
H	-12.61130000	-0.07650000	-26.35040000
C	-13.01360000	2.73210000	-28.13010000
C	-9.13570000	-0.32080000	-26.07130000
C	-8.03670000	-1.49320000	-27.78960000
H	-8.54550000	0.72230000	-27.94270000
C	-13.23450000	-3.34600000	-26.85250000
H	-12.31960000	-4.61590000	-28.34330000
H	-13.98860000	-1.89410000	-25.44880000
H	-12.19200000	3.45380000	-28.03330000
H	-13.59740000	2.93880000	-29.03760000
C	-13.93670000	2.84490000	-26.92600000
C	-7.65090000	-0.56210000	-25.63430000
C	-9.32360000	-1.83050000	-25.80020000
H	-9.75000000	0.46160000	-25.62160000
C	-7.87150000	-1.93700000	-26.32200000
H	-8.55100000	-2.24610000	-28.40680000
H	-7.09320000	-1.24270000	-28.29550000
Br	-14.35490000	-4.76260000	-26.11640000
Cl	-13.01050000	2.56360000	-25.41360000
Cl	-15.26070000	1.66580000	-27.03980000
Cl	-14.61780000	4.49820000	-26.90290000
H	-6.86960000	0.09290000	-26.03940000
H	-7.57010000	-0.62750000	-24.54390000
H	-10.09880000	-2.38900000	-26.33100000
H	-9.38630000	-2.03110000	-24.72570000
C	-7.05040000	-3.14730000	-26.03730000
C	-6.45700000	-3.89080000	-27.05790000
C	-6.89790000	-3.58580000	-24.71740000
C	-5.72840000	-5.04170000	-26.77160000
H	-6.55950000	-3.58240000	-28.09900000
C	-6.17130000	-4.72770000	-24.41230000
H	-7.35860000	-3.02320000	-23.90640000
C	-5.59340000	-5.44180000	-25.45320000
H	-5.27060000	-5.62010000	-27.56850000
H	-6.05860000	-5.06110000	-23.38460000
Br	-4.56900000	-7.05250000	-25.05740000

8. X-ray Crystallographic Data

8.1 X-ray Crystallographic Data of 5l

R_1=7.11%

Submitted by: **Ziyi Chen, Davies Lab**

Data collected by: **John Bacsa**

Crystal Data and Experimental

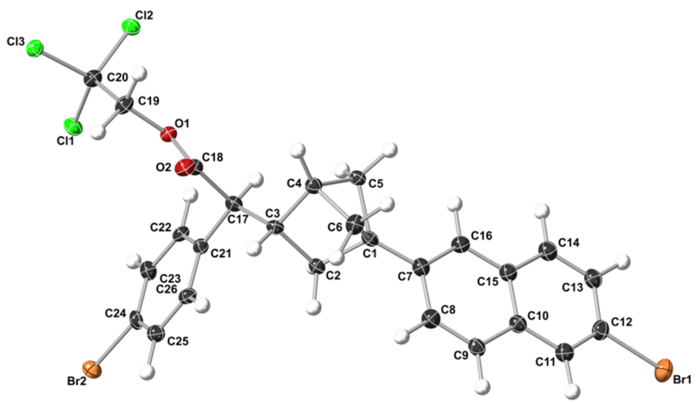

Experimental. Single colorless needle-shaped crystals of ZC-02-201B were chosen from the sample as supplied. A suitable crystal with dimensions 0.27 × 0.07 × 0.04 mm^3 was selected and mounted on a loop with IVH oil on a XtaLAB Synergy-S diffractometer. The crystal was kept at a steady T = 100.0(1) K during data collection. The structure was solved with the ShelXT (Sheldrick, 2015) solution program and by using Olex2 1.5-alpha (Dolomanov et al., 2009). The model was refined with ShelXL 2018/3 (Sheldrick, 2015) using full matrix least squares minimisation on $\boldsymbol{F}^2$. Crystallographic data of these structures, including cif, res, fcf, and hkl files, have been deposited with the Cambridge Crystallographic Data Centre with deposition numbers **2432903**. Copies of these data can be requested, free of charge, from the CCDC website at https://www.ccdc.cam.ac.uk/structures/.

Crystal Data. Identification code: 2432903, $C_{26}H_{21}Br_2Cl_3O_2$, M_r = 631.60, triclinic, P1 (No. 1), a = 5.66690(8) Å, b = 9.36289(15) Å, c = 23.3182(5) Å, α = 92.2317(15)°, β = 91.4516(14)°, γ = 90.1423(12)°, V = 1235.89(4) Å^3, T = 100.00(10) K, Z = 2, Z' = 2, μ(Cu K$_\alpha$) = 7.327, 12102 reflections measured, 12102 unique (R_{int} = NA) which were used in all calculations. The final wR_2 was 0.2071 (all data) and R_1 was 0.0711 (I≥2 σ(I)).

**Compound**	ZC-02-201B
Formula	$C_{26}H_{21}Br_2Cl_3O_2$
$D_{calc.}$/ g cm^{-3}	1.697
μ/mm^{-1}	7.327
Formula Weight	631.60
Color	colorless
Shape	needle-shaped
Size/mm^3	0.27×0.07×0.04
T/K	100.00(10)
Crystal System	triclinic
Flack Parameter	0.04(2)
Hooft Parameter	0.011(4)
Space Group	P1
a/Å	5.66690(8)
b/Å	9.36289(15)
c/Å	23.3182(5)
α/°	92.2317(15)
β/°	91.4516(14)
γ/°	90.1423(12)
V/Å^3	1235.89(4)
Z	2
Z'	2
Wavelength/Å	1.54184
Radiation type	Cu K$_\alpha$
Θ_{min}/°	3.795
Θ_{max}/°	77.051
Measured Refl's.	12102
Indep't Refl's	12102
Refl's I≥2 σ(I)	11906
R_{int}	NA
Parameters	596
Restraints	636
Largest Peak	1.669
Deepest Hole	-1.278
GooF	1.128
wR_2 (all data)	0.2071
wR_2	0.2046
R_1 (all data)	0.0720
R_1	0.0711

Structure Quality Indicators

**Reflections:**	d min (CuKα) 2Θ=154.1° 0.79	I/σ(I) 20.1	Full 135.4° 95% to 154.1° 97.9		
**Refinement:**	Shift 0.000	Max Peak 1.7	Min Peak -1.3	GooF 1.128	Hooft .011(4)

A colorless needle-shaped-shaped twinned crystal with dimensions 0.27 × 0.07 × 0.04 mm^3 was mounted on a loop with IVH oil. Data were collected using a XtaLAB Synergy, Dualflex, HyPix diffractometer operating at T = 100.00(10) K.

Data were measured using ω scans with Cu K_α radiation. The twinned diffraction pattern was indexed and the total number of runs and images was based on the strategy calculation from the program CrysAlisPro system (CCD 44.57a 64-bit (release 20-06-2024)).

The twinned crystal consisted of two overlapping domains. The angles between the unit cell edges are close to 90°. Owing to a two-fold rotation about the b-axis [010], the reflections from the two domains are nearly but not completely overlapping. In reciprocal space, this two-fold axis is the [0, 1, -0.1] direction. Consequently, the diffraction patterns are almost collinear, with a slight offset of -0.1 along the b-axis. The rotational angles between the two diffraction patterns is close to 5° along the a* direction.

The maximum resolution that was achieved was Θ = 77.051° (0.79 Å). The unit cell was refined using CrysAlisPro 1.171.44.57a (Rigaku OD, 2024) on 29516 reflections, 244% of the observed reflections.

Data reduction, scaling and absorption corrections were performed using CrysAlisPro 1.171.44.57a (Rigaku OD, 2024). The final completeness is 97.90 % out to 77.051° in Θ. A numerical absorption correction based on gaussian integration over a multifaceted crystal model was performed using CrysAlisPro 1.171.42.74a (Rigaku Oxford Diffraction, 2022). An empirical absorption correction using spherical harmonics, implemented in SCALE3 ABSPACK scaling algorithm was also applied.The absorption coefficient μ of this material is 7.327 mm^{-1} at this wavelength (λ = 1.54184Å) and the minimum and maximum transmissions are 0.289 and 0.777.

The structure was solved and the space group $P1$ (# 1) determined by the ShelXT (Sheldrick, 2015) structure solution program using using dual methods and refined by full matrix least squares minimisation on $\boldsymbol{F}^2$ using version 2018/3 of ShelXL 2018/3 (Sheldrick, 2015). All non-hydrogen atoms were refined anisotropically. Hydrogen atom positions were calculated geometrically and refined using the riding model.

The value of Z' is 2. The moiety formula is C26 H21 Br2 Cl3 O2.

The Flack parameter was refined to 0.04(2). Determination of absolute structure using Bayesian statistics on Bijvoet differences using the Olex2 results in 0.011(4). The chiral atoms in this structure are: C3(S), C3B(S), C17(S), C17B(S). Note: The Flack parameter is used to determine chirality of the crystal studied, the value should be near 0, a value of 1 means that the stereochemistry is wrong and the model should be inverted. A value of 0.5 means that the crystal consists of a racemic mixture of the two enantiomers.

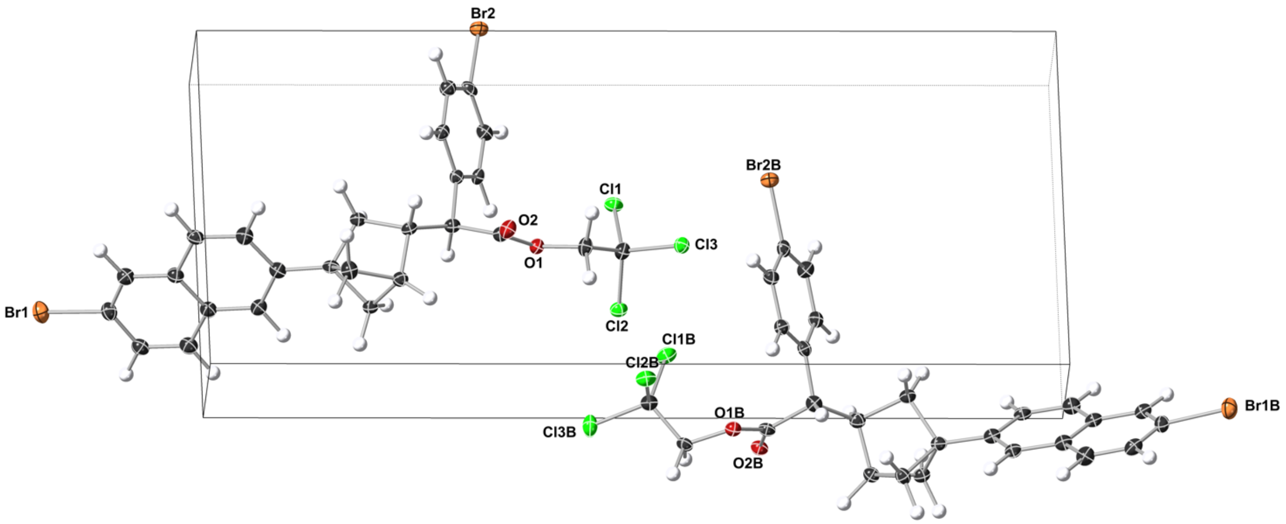

Figure 5 Thermal ellipsoidal (50% probability) representation of the asymmetric unit. There are two independent molecules in the asymmetric unit. The structure is chiral, space group P1.

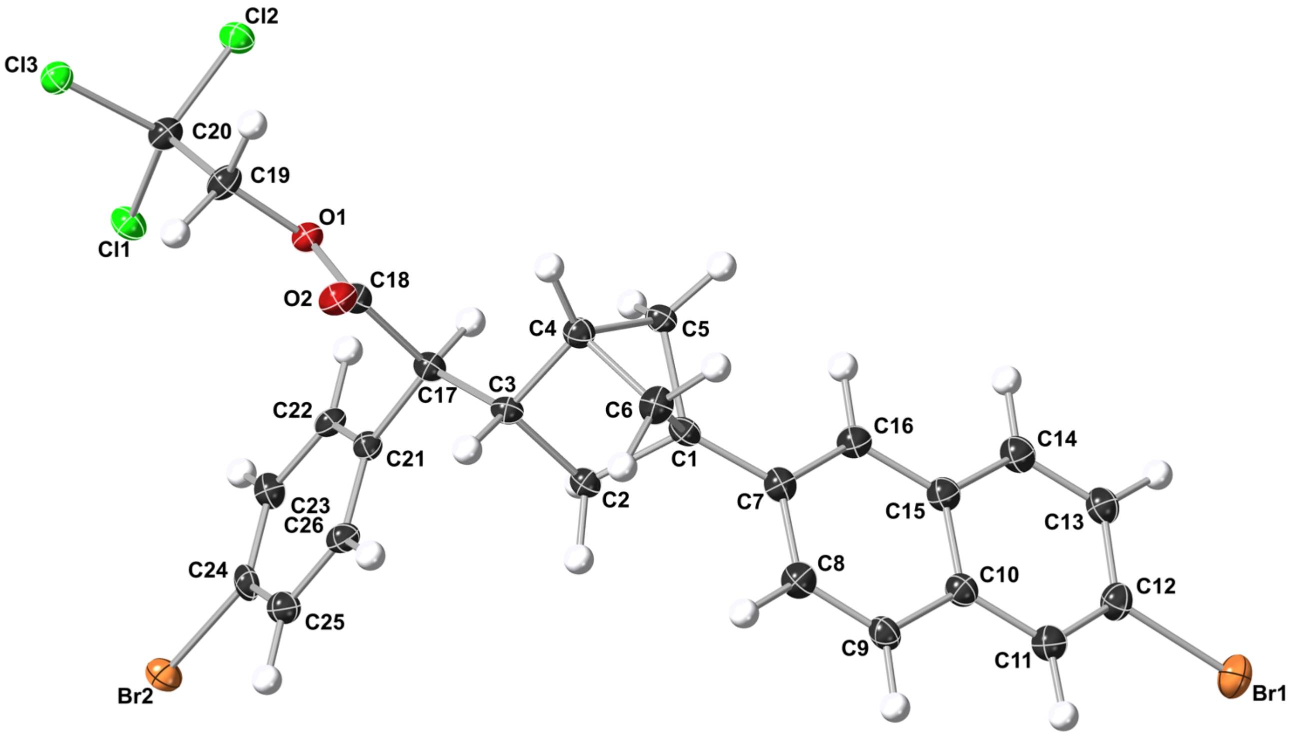

Figure 6 Thermal ellipsoidal (50% probability, non-hydrogen atoms) representation of the molecular structure (and one of the two independent molecules in the crystal structure). The chiral atoms in this structure are: C3(S), and C17(S).

_*twin_special_details*: Component 2 rotated by 179.9833° around [-0.00 1.00 -0.10] (reciprocal) or [0.00 1.00 -

0.00] (direct)

Data Plots: Diffraction Data

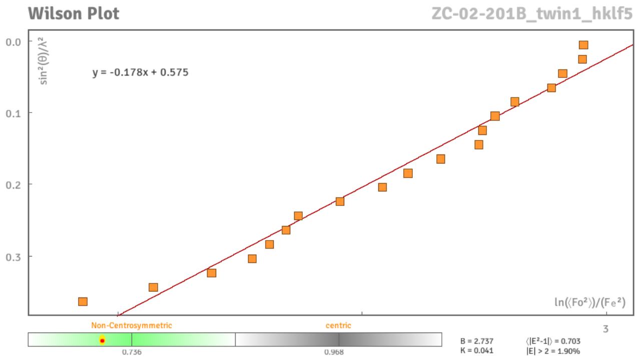

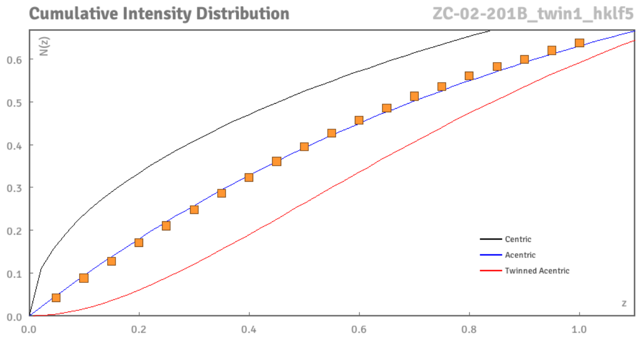

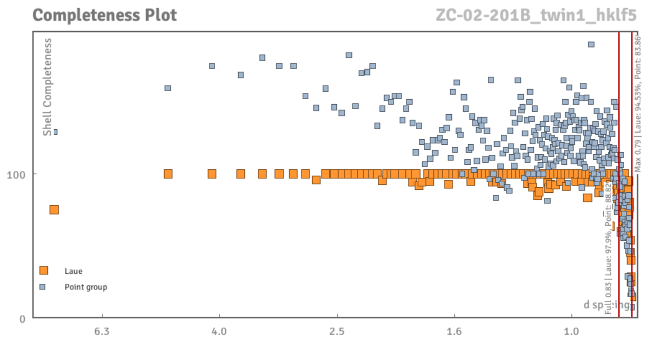

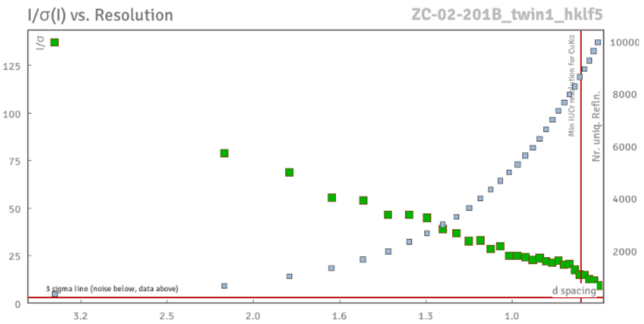

Data Plots: Refinement and Data

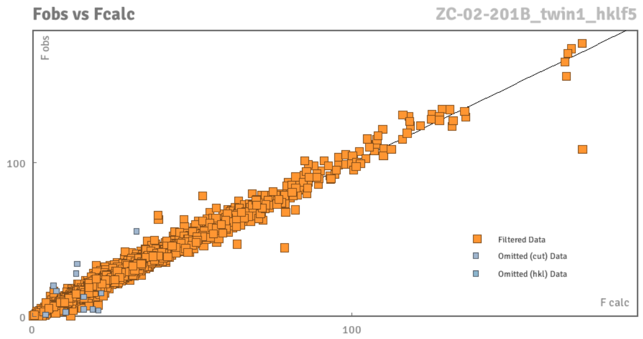

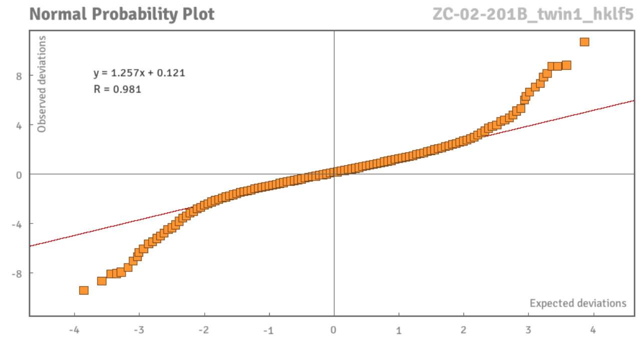

Reflection Statistics

Total reflections (after filtering)	18447	Unique reflections	8767
Completeness	0.839	Mean I/σ	27.75
hkl_{max} collected	(7, 11, 29)	hkl_{min} collected	(-7, -11, -29)
hkl_{max} used	(7, 11, 29)	hkl_{min} used	(-7, -11, -29)
Lim d_{max} collected	100.0	Lim d_{min} collected	0.77
d_{max} used	11.65	d_{min} used	0.79
Friedel pairs	3834	Friedel pairs merged	0
Inconsistent equivalents	518	R_{int}	0.3032
R_{sigma}	0.0497	Intensity transformed	0
Omitted reflections	0	Omitted by user (OMIT hkl)	28
Multiplicity	(5902, 2425, 456)	Maximum multiplicity	4
Removed systematic absences	0	Filtered off (Shel/OMIT)	0

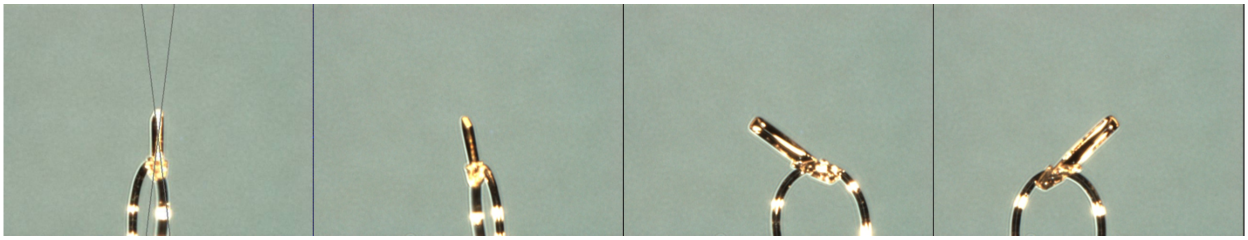

Table 1: Fractional Atomic Coordinates (×10⁴) and Equivalent Isotropic Displacement Parameters (Å²×10³) for ZC-02-201B_twin1_hklf5. U_{eq} is defined as 1/3 of the trace of the orthogonalised U_{ij}.

Atom	x	y	z	U_{eq}
Br1	10252(6)	3205(4)	-1833.9(12)	44.2(5)
Br2	8691(6)	11471(3)	3385.1(12)	41.0(4)
Cl1	8860(8)	6223(4)	4868.5(17)	30.7(7)
Cl2	9472(7)	3176(5)	4883.4(16)	30.5(7)
Cl3	6012(7)	4542(5)	5609.2(14)	29.0(7)
O1	6392(13)	4570(11)	3926(3)	23.8(19)
O2	2674(14)	4471(14)	3560(3)	32(2)
C1	5077(17)	3734(11)	1506(3)	24(2)
C2	5286(19)	5187(11)	1844(4)	27(3)
C3	4268(19)	4760(11)	2426(4)	25(2)
C4	3865(19)	3166(11)	2295(4)	28(3)
C5	6070(20)	2697(12)	1959(4)	28(3)
C6	2576(18)	3229(13)	1692(4)	32(3)
C7	5713(10)	3731(7)	893(3)	29(3)
C8	4394(15)	4540(11)	508(3)	32(3)
C9	4900(20)	4591(16)	-58(4)	32(3)
C10	6830(20)	3761(16)	-287(4)	32(3)
C11	7410(20)	3809(16)	-863(4)	33(3)
C12	9310(20)	3075(16)	-1049(4)	34(3)
C13	10720(20)	2237(16)	-688(4)	33(3)
C14	10130(20)	2182(17)	-131(4)	34(3)
C15	8190(20)	2948(15)	94(4)	30(3)
C16	7580(16)	2916(12)	676(4)	29(3)
C17	5913(17)	5111(11)	2948(4)	25(2)
C18	4735(18)	4657(13)	3494(4)	26(2)
C19	5482(18)	4408(12)	4482(4)	31(3)
C20	7386(19)	4592(11)	4932(4)	28(3)
C21	6577(8)	6683(8)	3030(3)	24(2)
C22	8718(13)	7042(7)	3305(4)	25(2)
C23	9338(17)	8466(6)	3407(5)	29(2)
C24	7800(15)	9530(7)	3237(5)	29(3)
C25	5662(16)	9194(7)	2960(5)	32(3)
C26	5052(15)	7766(7)	2866(5)	29(2)
Br1B	-285(6)	-1368(4)	11831.4(12)	44.0(4)
Br2B	1214(6)	5723(3)	6611.1(12)	33.4(4)
Cl1B	4840(8)	1045(5)	5364.0(17)	34.6(7)
Cl2B	446(7)	-371(5)	5066.1(17)	31.4(7)
Cl3B	4627(8)	-1113(6)	4438.6(15)	42.7(10)
O1B	3400(14)	-1392(9)	6088(3)	26.5(19)
O2B	7162(14)	-1356(12)	6427(3)	28(2)
C1B	4916(17)	-1584(12)	8493(4)	28(3)
C2B	4693(19)	-209(11)	8149(4)	26(2)
C3B	5682(19)	-766(12)	7565(4)	30(3)
C4B	6100(20)	-2326(12)	7705(4)	30(3)
C5B	3910(20)	-2726(12)	8047(4)	29(3)
C6B	7409(18)	-2123(13)	8304(4)	30(3)
C7B	4293(16)	-1457(10)	9107(4)	27(3)

Atom	x	y	z	U_{eq}
C8B	5620(20)	-567(14)	9489(4)	28(3)
C9B	5130(20)	-409(16)	10056(4)	32(3)
C10B	3160(20)	-1157(15)	10285(4)	28(3)
C11B	2580(20)	-987(16)	10861(4)	33(3)
C12B	670(20)	-1680(16)	11049(4)	33(3)
C13B	-730(20)	-2605(16)	10692(4)	36(3)
C14B	-140(20)	-2780(16)	10136(4)	34(3)
C15B	1810(20)	-2067(15)	9909(4)	28(3)
C16B	2400(20)	-2209(14)	9326(4)	26(3)
C17B	3997(18)	-542(11)	7049(4)	28(2)
C18B	5114(18)	-1135(12)	6500(4)	25(2)
C19B	4202(19)	-1734(10)	5527(4)	27(3)
C20B	3565(19)	-585(11)	5126(4)	24(3)
C21B	3349(15)	1010(8)	6956(4)	26(2)
C22B	1209(17)	1308(8)	6680(5)	29(2)
C23B	598(15)	2709(6)	6573(5)	31(3)
C24B	2122(8)	3812(7)	6750(3)	29(3)
C25B	4274(11)	3537(8)	7020(5)	31(3)
C26B	4875(17)	2130(7)	7120(5)	27(2)

Table 2: Anisotropic Displacement Parameters (×10[4]) for ZC-02-201B_twin1_hklf5. The anisotropic displacement factor exponent takes the form: $-2\pi^2[h^2a^{*2} \times U_{11} + ... + 2hka^* \times b^* \times U_{12}]$

Atom	U_{11}	U_{22}	U_{33}	U_{23}	U_{13}	U_{12}
Br1	45.6(10)	54.2(12)	33.0(7)	-2.0(7)	10.2(6)	-1.3(9)
Br2	59.3(12)	23.1(8)	40.4(7)	0.3(6)	-3.0(7)	-2.2(8)
Cl1	27.5(17)	22.6(17)	42.0(15)	-0.3(12)	4.0(12)	-4.2(14)
Cl2	27.3(17)	27.8(17)	36.7(14)	3.8(12)	4.1(11)	3.2(14)
Cl3	27.8(16)	31.6(18)	27.9(13)	2.3(11)	3.1(10)	-0.3(14)
O1	20(4)	26(5)	26(3)	3(3)	2(3)	-4(4)
O2	23(4)	42(7)	33(4)	8(4)	-1(3)	0(4)
C1	17(5)	22(6)	33(4)	0(4)	-2(4)	4(5)
C2	28(7)	22(6)	30(5)	1(4)	1(4)	0(5)
C3	23(6)	23(6)	30(4)	4(4)	0(4)	2(5)
C4	28(6)	26(6)	29(5)	2(4)	2(4)	0(5)
C5	32(7)	25(7)	29(5)	3(4)	3(4)	8(5)
C6	24(6)	39(9)	33(5)	0(5)	3(4)	-6(6)
C7	24(6)	29(7)	33(4)	-2(4)	1(4)	-2(5)
C8	21(6)	39(9)	36(5)	2(5)	3(4)	1(6)
C9	30(7)	30(8)	35(5)	-3(5)	1(4)	9(6)
C10	31(7)	31(8)	33(5)	0(4)	2(4)	6(5)
C11	25(7)	36(8)	36(5)	2(5)	3(4)	1(6)
C12	32(7)	41(9)	30(5)	-1(5)	7(4)	2(6)
C13	31(7)	31(8)	37(5)	-4(5)	3(4)	-1(6)
C14	27(7)	36(9)	39(5)	-2(5)	-1(4)	4(6)
C15	26(7)	32(8)	33(5)	0(4)	0(4)	2(5)
C16	22(6)	33(8)	32(5)	3(5)	-2(4)	1(5)
C17	21(6)	23(5)	31(5)	2(4)	2(4)	-2(5)
C18	24(4)	26(7)	28(4)	1(4)	0(3)	0(5)
C19	26(7)	44(9)	25(5)	6(5)	2(4)	-1(6)
C20	26(6)	31(7)	28(5)	4(5)	-2(4)	-10(6)
C21	26(6)	24(5)	23(5)	2(4)	4(4)	-2(4)
C22	26(6)	29(5)	21(5)	6(4)	1(4)	-3(5)
C23	21(6)	31(6)	33(6)	-3(4)	5(4)	-2(4)
C24	32(6)	28(6)	29(5)	-7(4)	13(4)	2(5)
C25	31(7)	33(6)	32(6)	4(5)	7(4)	6(5)

Atom	*U11*	*U22*	*U33*	*U23*	*U13*	*U12*
C26	28(6)	33(6)	26(5)	7(4)	3(4)	3(5)
Br1B	45.5(10)	55.9(12)	31.1(6)	4.5(7)	10.0(6)	2.6(9)
Br2B	39.0(9)	23.0(8)	38.5(7)	2.6(5)	1.0(6)	0.7(7)
Cl1B	27.4(17)	28.8(19)	48.1(17)	9.6(13)	-0.3(12)	-7.5(15)
Cl2B	16.6(15)	30.0(19)	47.9(16)	4.2(13)	1.0(11)	0.8(14)
Cl3B	32.9(19)	70(3)	25.5(13)	4.2(15)	1.8(11)	16(2)
O1B	24(5)	25(5)	31(4)	1(3)	2(3)	-3(4)
O2B	22(4)	26(6)	35(4)	-2(4)	2(3)	1(4)
C1B	29(6)	22(7)	31(4)	1(4)	-1(4)	2(5)
C2B	21(6)	25(6)	32(5)	3(4)	1(4)	1(5)
C3B	29(7)	29(6)	32(5)	2(4)	2(4)	3(5)
C4B	29(6)	30(6)	30(5)	-1(4)	4(4)	4(5)
C5B	29(6)	30(7)	28(5)	-3(4)	3(4)	-4(5)
C6B	28(6)	27(8)	35(5)	4(5)	1(4)	3(5)
C7B	22(6)	26(7)	32(4)	4(4)	-1(4)	-1(5)
C8B	27(7)	17(6)	38(5)	5(4)	1(4)	-7(5)
C9B	27(7)	32(8)	38(5)	2(5)	1(4)	-6(6)
C10B	25(6)	23(6)	35(5)	6(4)	-1(4)	1(5)
C11B	39(7)	28(8)	34(5)	7(5)	1(4)	2(6)
C12B	38(7)	36(8)	26(5)	4(4)	7(4)	4(6)
C13B	34(7)	36(8)	39(5)	11(5)	2(4)	1(6)
C14B	22(6)	42(9)	39(5)	4(5)	-3(4)	-4(6)
C15B	17(6)	32(7)	35(5)	3(4)	-3(4)	2(5)
C16B	21(6)	25(7)	33(5)	4(4)	-3(4)	1(5)
C17B	19(6)	32(6)	32(4)	-1(4)	2(4)	-2(5)
C18B	22(4)	20(6)	34(4)	-3(4)	1(3)	-1(4)
C19B	31(7)	20(7)	30(5)	-5(4)	-1(4)	-2(5)
C20B	11(5)	27(7)	34(5)	2(4)	3(4)	-3(5)
C21B	25(5)	31(5)	23(5)	-1(4)	7(4)	1(4)
C22B	29(6)	29(6)	27(5)	1(4)	3(4)	1(5)
C23B	32(7)	32(6)	30(6)	-1(5)	1(5)	8(5)
C24B	29(6)	37(7)	22(5)	2(5)	7(4)	2(5)
C25B	27(6)	32(6)	34(6)	4(5)	0(4)	-4(5)
C26B	22(6)	33(5)	27(5)	1(4)	6(4)	1(4)

Table 3: Bond Lengths in Å for ZC-02-201B_twin1_hklf5.

Atom	Atom	Length/Å
Br1	C12	1.927(8)
Br2	C24	1.901(7)
Cl1	C20	1.752(11)
Cl2	C20	1.782(11)
Cl3	C20	1.779(9)
O1	C18	1.365(11)
O1	C19	1.419(10)
O2	C18	1.195(12)
C1	C2	1.549(12)
C1	C5	1.557(12)
C1	C6	1.570(13)
C1	C7	1.483(11)
C2	H2A	1.0930
C2	H2B	1.0930
C2	C3	1.554(12)
C3	H3	1.0930
C3	C4	1.528(14)
C3	C17	1.539(12)

Atom	Atom	Length/Å
C4	H4	1.0930
C4	C5	1.545(13)
C4	C6	1.570(12)
C5	H5A	1.0930
C5	H5B	1.0930
C6	H6A	1.0930
C6	H6B	1.0930
C7	C8	1.401(7)
C7	C16	1.401(7)
C8	H8	1.0780
C8	C9	1.359(12)
C9	H9	1.0780
C9	C10	1.441(13)
C10	C11	1.395(12)
C10	C15	1.409(13)
C11	H11	1.0780
C11	C12	1.351(15)
C12	C13	1.407(15)

Atom	Atom	Length/Å
C13	H13	1.0780
C13	C14	1.352(13)
C14	H14	1.0780
C14	C15	1.414(14)
C15	C16	1.409(12)
C16	H16	1.0780
C17	H17	1.0930
C17	C18	1.527(11)
C17	C21	1.522(12)
C19	H19A	1.0930
C19	H19B	1.0930
C19	C20	1.492(12)
C21	C22	1.392(7)
C21	C26	1.392(7)
C22	H22	1.0780
C22	C23	1.3879(15)
C23	H23	1.0780
C23	C24	1.3881(15)
C24	C25	1.3880(15)
C25	H25	1.0780
C25	C26	1.3882(15)
C26	H26	1.0780
Br1B	C12B	1.927(8)
Br2B	C24B	1.901(7)
Cl1B	C20B	1.751(11)
Cl2B	C20B	1.782(10)
Cl3B	C20B	1.779(9)
O1B	C18B	1.363(11)
O1B	C19B	1.419(10)
O2B	C18B	1.194(12)
C1B	C2B	1.548(12)
C1B	C5B	1.559(12)
C1B	C6B	1.569(13)
C1B	C7B	1.483(11)
C2B	H2BA	1.0930
C2B	H2BB	1.0930
C2B	C3B	1.556(12)
C3B	H3B	1.0930
C3B	C4B	1.527(14)
C3B	C17B	1.539(12)
C4B	H4B	1.0930
C4B	C5B	1.544(13)
C4B	C6B	1.569(12)
C5B	H5BA	1.0930
C5B	H5BB	1.0930
C6B	H6BA	1.0930
C6B	H6BB	1.0930
C7B	C8B	1.401(8)
C7B	C16B	1.401(8)
C8B	H8B	1.0780
C8B	C9B	1.360(12)
C9B	H9B	1.0780
C9B	C10B	1.441(14)
C10B	C11B	1.395(12)
C10B	C15B	1.408(13)
C11B	H11B	1.0780
C11B	C12B	1.351(15)
C12B	C13B	1.407(15)
C13B	H13B	1.0780
C13B	C14B	1.352(13)
C14B	H14B	1.0780
C14B	C15B	1.415(14)
C15B	C16B	1.409(12)
C16B	H16B	1.0780
C17B	H17B	1.0930
C17B	C18B	1.528(11)
C17B	C21B	1.522(12)
C19B	H19C	1.0930
C19B	H19D	1.0930
C19B	C20B	1.492(12)
C21B	C22B	1.392(7)
C21B	C26B	1.392(7)
C22B	H22B	1.0780
C22B	C23B	1.388(3)
C23B	H23B	1.0780
C23B	C24B	1.3880(10)
C24B	C25B	1.3880(10)
C25B	H25B	1.0780
C25B	C26B	1.388(3)
C26B	H26B	1.0780

Table 4: Bond Angles in ° for ZC-02-201B_twin1_hklf5.

Atom	Atom	Atom	Angle/°
C18	O1	C19	115.2(7)
C2	C1	C5	101.1(7)
C2	C1	C6	100.6(7)
C5	C1	C6	85.6(7)
C7	C1	C2	116.2(8)
C7	C1	C5	125.7(7)
C7	C1	C6	121.4(7)
C1	C2	H2A	111.8
C1	C2	H2B	111.8
C1	C2	C3	99.7(7)
H2A	C2	H2B	109.6
C3	C2	H2A	111.8
C3	C2	H2B	111.8
C2	C3	H3	109.4
C4	C3	C2	99.4(7)
C4	C3	H3	109.4
C4	C3	C17	114.8(8)
C17	C3	C2	114.0(8)
C17	C3	H3	109.4
C3	C4	H4	120.2
C3	C4	C5	103.9(8)
C3	C4	C6	99.9(8)
C5	C4	H4	120.2
C5	C4	C6	86.0(6)

Atom	Atom	Atom	Angle/˚
C6	C4	H4	120.2
C1	C5	H5A	114.7
C1	C5	H5B	114.7
C4	C5	C1	83.6(7)
C4	C5	H5A	114.7
C4	C5	H5B	114.7
H5A	C5	H5B	111.8
C1	C6	H6A	114.9
C1	C6	H6B	114.9
C4	C6	C1	82.4(7)
C4	C6	H6A	114.9
C4	C6	H6B	114.9
H6A	C6	H6B	112.0
C8	C7	C1	119.9(6)
C16	C7	C1	122.3(6)
C16	C7	C8	117.8(7)
C7	C8	H8	118.7
C9	C8	C7	122.7(8)
C9	C8	H8	118.7
C8	C9	H9	119.9
C8	C9	C10	120.1(8)
C10	C9	H9	119.9
C11	C10	C9	121.4(9)
C11	C10	C15	120.4(9)
C15	C10	C9	118.2(8)
C10	C11	H11	120.5
C12	C11	C10	119.1(9)
C12	C11	H11	120.5
C11	C12	Br1	119.8(8)
C11	C12	C13	122.8(8)
C13	C12	Br1	117.4(7)
C12	C13	H13	121.1
C14	C13	C12	117.8(9)
C14	C13	H13	121.1
C13	C14	H14	118.9
C13	C14	C15	122.3(10)
C15	C14	H14	118.9
C10	C15	C14	117.6(8)
C10	C15	C16	119.7(8)
C16	C15	C14	122.7(8)
C7	C16	C15	121.5(7)
C7	C16	H16	119.3
C15	C16	H16	119.3
C3	C17	H17	108.3
C18	C17	C3	109.5(8)
C18	C17	H17	108.3
C21	C17	C3	114.6(7)
C21	C17	H17	108.3
C21	C17	C18	107.8(7)
O1	C18	C17	109.6(8)
O2	C18	O1	123.2(8)
O2	C18	C17	127.0(8)
O1	C19	H19A	109.5
O1	C19	H19B	109.5
O1	C19	C20	110.9(7)
H19A	C19	H19B	108.0
C20	C19	H19A	109.5
C20	C19	H19B	109.5

Atom	Atom	Atom	Angle/˚
Cl1	C20	Cl2	109.0(6)
Cl1	C20	Cl3	110.4(5)
Cl3	C20	Cl2	108.6(5)
C19	C20	Cl1	110.7(7)
C19	C20	Cl2	111.1(7)
C19	C20	Cl3	107.1(7)
C22	C21	C17	118.7(6)
C26	C21	C17	121.8(6)
C26	C21	C22	119.3(7)
C21	C22	H22	119.9
C23	C22	C21	120.2(7)
C23	C22	H22	119.9
C22	C23	H23	120.2
C22	C23	C24	119.6(8)
C24	C23	H23	120.2
C23	C24	Br2	118.6(5)
C25	C24	Br2	120.3(5)
C25	C24	C23	121.1(7)
C24	C25	H25	120.6
C24	C25	C26	118.8(7)
C26	C25	H25	120.6
C21	C26	H26	119.5
C25	C26	C21	121.0(7)
C25	C26	H26	119.5
C18B	O1B	C19B	115.9(7)
C2B	C1B	C5B	101.1(7)
C2B	C1B	C6B	100.5(7)
C5B	C1B	C6B	85.6(7)
C7B	C1B	C2B	116.4(8)
C7B	C1B	C5B	125.4(7)
C7B	C1B	C6B	121.5(7)
C1B	C2B	H2BA	111.8
C1B	C2B	H2BB	111.8
C1B	C2B	C3B	99.7(7)
H2BA	C2B	H2BB	109.5
C3B	C2B	H2BA	111.8
C3B	C2B	H2BB	111.8
C2B	C3B	H3B	109.5
C4B	C3B	C2B	99.3(7)
C4B	C3B	H3B	109.5
C4B	C3B	C17B	114.8(8)
C17B	C3B	C2B	113.7(8)
C17B	C3B	H3B	109.5
C3B	C4B	H4B	120.1
C3B	C4B	C5B	103.9(8)
C3B	C4B	C6B	100.0(8)
C5B	C4B	H4B	120.1
C5B	C4B	C6B	86.1(6)
C6B	C4B	H4B	120.1
C1B	C5B	H5BA	114.7
C1B	C5B	H5BB	114.7
C4B	C5B	C1B	83.6(7)
C4B	C5B	H5BA	114.7
C4B	C5B	H5BB	114.7
H5BA	C5B	H5BB	111.8
C1B	C6B	C4B	82.4(7)
C1B	C6B	H6BA	114.9
C1B	C6B	H6BB	114.9

Atom	Atom	Atom	Angle/°
C4B	C6B	H6BA	114.9
C4B	C6B	H6BB	114.9
H6BA	C6B	H6BB	112.0
C8B	C7B	C1B	120.1(6)
C8B	C7B	C16B	117.7(7)
C16B	C7B	C1B	122.2(7)
C7B	C8B	H8B	118.7
C9B	C8B	C7B	122.7(8)
C9B	C8B	H8B	118.7
C8B	C9B	H9B	119.9
C8B	C9B	C10B	120.1(9)
C10B	C9B	H9B	119.9
C11B	C10B	C9B	121.4(9)
C11B	C10B	C15B	120.4(9)
C15B	C10B	C9B	118.2(8)
C10B	C11B	H11B	120.5
C12B	C11B	C10B	119.0(9)
C12B	C11B	H11B	120.5
C11B	C12B	Br1B	119.8(8)
C11B	C12B	C13B	122.8(8)
C13B	C12B	Br1B	117.3(7)
C12B	C13B	H13B	121.1
C14B	C13B	C12B	117.8(10)
C14B	C13B	H13B	121.1
C13B	C14B	H14B	118.9
C13B	C14B	C15B	122.3(10)
C15B	C14B	H14B	118.9
C10B	C15B	C14B	117.6(8)
C10B	C15B	C16B	119.7(8)
C16B	C15B	C14B	122.6(8)
C7B	C16B	C15B	121.5(8)
C7B	C16B	H16B	119.2
C15B	C16B	H16B	119.2
C3B	C17B	H17B	108.3
C18B	C17B	C3B	109.7(8)
C18B	C17B	H17B	108.3

Atom	Atom	Atom	Angle/°
C21B	C17B	C3B	114.5(7)
C21B	C17B	H17B	108.3
C21B	C17B	C18B	107.7(7)
O1B	C18B	C17B	109.6(8)
O2B	C18B	O1B	123.5(8)
O2B	C18B	C17B	126.9(8)
O1B	C19B	H19C	109.5
O1B	C19B	H19D	109.5
O1B	C19B	C20B	110.9(7)
H19C	C19B	H19D	108.1
C20B	C19B	H19C	109.5
C20B	C19B	H19D	109.5
Cl1B	C20B	Cl2B	108.9(6)
Cl1B	C20B	Cl3B	110.3(5)
Cl3B	C20B	Cl2B	108.4(5)
C19B	C20B	Cl1B	110.5(7)
C19B	C20B	Cl2B	111.1(7)
C19B	C20B	Cl3B	107.5(7)
C22B	C21B	C17B	118.8(6)
C22B	C21B	C26B	119.4(7)
C26B	C21B	C17B	121.8(6)
C21B	C22B	H22B	119.9
C23B	C22B	C21B	120.2(7)
C23B	C22B	H22B	119.9
C22B	C23B	H23B	120.2
C24B	C23B	C22B	119.5(8)
C24B	C23B	H23B	120.2
C23B	C24B	Br2B	118.6(5)
C25B	C24B	Br2B	120.2(5)
C25B	C24B	C23B	121.1(7)
C24B	C25B	H25B	120.6
C24B	C25B	C26B	118.8(7)
C26B	C25B	H25B	120.6
C21B	C26B	H26B	119.5
C25B	C26B	C21B	120.9(7)
C25B	C26B	H26B	119.5

Table 5: Torsion Angles in ° for ZC-02-201B_twin1_hklf5.

Atom	Atom	Atom	Atom	Angle/°
Br1	C12	C13	C14	177.6(12)
Br2	C24	C25	C26	-179.1(9)
O1	C19	C20	Cl1	54.1(11)
O1	C19	C20	Cl2	-67.1(10)
O1	C19	C20	Cl3	174.5(8)
C1	C2	C3	C4	3.2(9)
C1	C2	C3	C17	125.7(9)
C1	C7	C8	C9	179.4(12)
C1	C7	C16	C15	-178.7(11)
C2	C1	C5	C4	65.0(8)
C2	C1	C6	C4	-66.1(7)
C2	C1	C7	C8	-63.4(11)
C2	C1	C7	C16	118.1(10)
C2	C3	C4	C5	40.7(9)
C2	C3	C4	C6	-47.6(9)
C2	C3	C17	C18	179.6(9)

Atom	Atom	Atom	Atom	Angle/°
C2	C3	C17	C21	58.4(11)
C3	C4	C5	C1	-64.3(8)
C3	C4	C6	C1	68.8(8)
C3	C17	C18	O1	162.3(9)
C3	C17	C18	O2	-21.4(16)
C3	C17	C21	C22	-152.5(9)
C3	C17	C21	C26	31.0(12)
C4	C3	C17	C18	-66.7(11)
C4	C3	C17	C21	172.1(7)
C5	C1	C2	C3	-45.5(9)
C5	C1	C6	C4	34.4(7)
C5	C1	C7	C8	168.4(10)
C5	C1	C7	C16	-10.1(14)
C5	C4	C6	C1	-34.7(7)
C6	C1	C2	C3	42.0(8)
C6	C1	C5	C4	-34.9(7)
C6	C1	C7	C8	59.3(13)
C6	C1	C7	C16	-119.3(11)
C6	C4	C5	C1	34.9(7)
C7	C1	C2	C3	175.0(7)
C7	C1	C5	C4	-160.9(9)
C7	C1	C6	C4	164.1(8)
C7	C8	C9	C10	2(2)
C8	C7	C16	C15	2.6(16)
C8	C9	C10	C11	-179.0(15)
C8	C9	C10	C15	-2(2)
C9	C10	C11	C12	176.2(16)
C9	C10	C15	C14	-177.6(15)
C9	C10	C15	C16	3(2)
C10	C11	C12	Br1	-176.8(12)
C10	C11	C12	C13	1(3)
C10	C15	C16	C7	-3(2)
C11	C10	C15	C14	-1(2)
C11	C10	C15	C16	179.7(14)
C11	C12	C13	C14	0(3)
C12	C13	C14	C15	-1(2)
C13	C14	C15	C10	2(2)
C13	C14	C15	C16	-178.9(14)
C14	C15	C16	C7	177.3(13)
C15	C10	C11	C12	0(2)
C16	C7	C8	C9	-2.0(17)
C17	C3	C4	C5	-81.3(9)
C17	C3	C4	C6	-169.6(8)
C17	C21	C22	C23	-177.5(9)
C17	C21	C26	C25	178.1(10)
C18	O1	C19	C20	-169.5(10)
C18	C17	C21	C22	85.4(9)
C18	C17	C21	C26	-91.1(10)
C19	O1	C18	O2	-7.2(18)
C19	O1	C18	C17	169.2(9)
C21	C17	C18	O1	-72.4(10)
C21	C17	C18	O2	103.9(14)
C21	C22	C23	C24	0.6(16)
C22	C21	C26	C25	1.7(15)
C22	C23	C24	Br2	179.7(8)
C22	C23	C24	C25	-0.8(19)
C23	C24	C25	C26	1.5(19)
C24	C25	C26	C21	-1.9(17)

Atom	Atom	Atom	Atom	Angle/°
C26	C21	C22	C23	-1.0(14)
Br1B	C12B	C13B	C14B	177.0(12)
Br2B	C24B	C25B	C26B	179.5(8)
O1B	C19B	C20B	Cl1B	59.8(9)
O1B	C19B	C20B	Cl2B	-61.3(9)
O1B	C19B	C20B	Cl3B	-179.7(6)
C1B	C2B	C3B	C4B	2.9(9)
C1B	C2B	C3B	C17B	125.3(9)
C1B	C7B	C8B	C9B	180.0(13)
C1B	C7B	C16B	C15B	-179.4(11)
C2B	C1B	C5B	C4B	65.0(8)
C2B	C1B	C6B	C4B	-66.1(7)
C2B	C1B	C7B	C8B	-63.8(13)
C2B	C1B	C7B	C16B	116.5(12)
C2B	C3B	C4B	C5B	41.0(9)
C2B	C3B	C4B	C6B	-47.4(9)
C2B	C3B	C17B	C18B	-179.4(9)
C2B	C3B	C17B	C21B	59.4(11)
C3B	C4B	C5B	C1B	-64.5(8)
C3B	C4B	C6B	C1B	68.7(8)
C3B	C17B	C18B	O1B	159.3(9)
C3B	C17B	C18B	O2B	-20.5(15)
C3B	C17B	C21B	C22B	-153.6(10)
C3B	C17B	C21B	C26B	29.0(13)
C4B	C3B	C17B	C18B	-66.0(11)
C4B	C3B	C17B	C21B	172.8(8)
C5B	C1B	C2B	C3B	-45.3(9)
C5B	C1B	C6B	C4B	34.4(7)
C5B	C1B	C7B	C8B	168.1(11)
C5B	C1B	C7B	C16B	-11.6(16)
C5B	C4B	C6B	C1B	-34.7(7)
C6B	C1B	C2B	C3B	42.2(8)
C6B	C1B	C5B	C4B	-34.9(7)
C6B	C1B	C7B	C8B	59.1(15)
C6B	C1B	C7B	C16B	-120.6(13)
C6B	C4B	C5B	C1B	34.9(7)
C7B	C1B	C2B	C3B	175.5(7)
C7B	C1B	C5B	C4B	-160.9(9)
C7B	C1B	C6B	C4B	163.8(9)
C7B	C8B	C9B	C10B	-1(2)
C8B	C7B	C16B	C15B	1(2)
C8B	C9B	C10B	C11B	-178.3(15)
C8B	C9B	C10B	C15B	1(2)
C9B	C10B	C11B	C12B	178.0(15)
C9B	C10B	C15B	C14B	-179.1(15)
C9B	C10B	C15B	C16B	-1(2)
C10B	C11B	C12B	Br1B	-176.1(11)
C10B	C11B	C12B	C13B	2(3)
C10B	C15B	C16B	C7B	0(2)
C11B	C10B	C15B	C14B	0(2)
C11B	C10B	C15B	C16B	178.9(14)
C11B	C12B	C13B	C14B	-1(3)
C12B	C13B	C14B	C15B	0(2)
C13B	C14B	C15B	C10B	0(2)
C13B	C14B	C15B	C16B	-178.0(15)
C14B	C15B	C16B	C7B	178.0(14)
C15B	C10B	C11B	C12B	-2(2)
C16B	C7B	C8B	C9B	0(2)

Atom	Atom	Atom	Atom	Angle/°
C17B	C3B	C4B	C5B	-80.6(9)
C17B	C3B	C4B	C6B	-169.0(8)
C17B	C21B	C22B	C23B	-178.0(10)
C17B	C21B	C26B	C25B	178.6(10)
C18B	O1B	C19B	C20B	-113.3(10)
C18B	C17B	C21B	C22B	84.1(11)
C18B	C17B	C21B	C26B	-93.3(11)
C19B	O1B	C18B	O2B	-9.7(15)
C19B	O1B	C18B	C17B	170.5(7)
C21B	C17B	C18B	O1B	-75.5(9)
C21B	C17B	C18B	O2B	104.7(13)
C21B	C22B	C23B	C24B	-0.8(17)
C22B	C21B	C26B	C25B	1.1(17)
C22B	C23B	C24B	Br2B	-178.8(9)
C22B	C23B	C24B	C25B	1.4(16)
C23B	C24B	C25B	C26B	-0.8(16)
C24B	C25B	C26B	C21B	-0.5(17)
C26B	C21B	C22B	C23B	-0.5(17)

Table 6: Hydrogen Fractional Atomic Coordinates (×10[4]) and Equivalent Isotropic Displacement Parameters (Å[2]×10[3]) for ZC-02-201B_twin1_hklf5. U_{eq} is defined as 1/3 of the trace of the orthogonalised U_{ij}.

Atom	x	y	z	U_{eq}
H2A	4226.26	6013.91	1643.72	32
H2B	7119.26	5547.56	1892.04	32
H3	2569.17	5286.35	2488.02	30
H4	3170.21	2487.97	2622.74	33
H5A	6075.75	1571.61	1816.34	34
H5B	7749.8	3026.64	2162.45	34
H6A	1189.87	4035.49	1665	38
H6B	1999.57	2194.8	1504.62	38
H8	2915.45	5146.61	665.39	38
H9	3870.08	5254.34	-335.38	38
H11	6364.6	4423.69	-1155.67	39
H13	12221.33	1659	-849.58	40
H14	11164.62	1531.79	150.32	41
H16	8575.03	2248	959.68	35
H17	7534.74	4494.41	2897.55	30
H19A	4692.6	3345.96	4505.16	37
H19B	4101.7	5200.08	4558.98	37
H22	9897.99	6210.97	3440.12	30
H23	11002.92	8743.96	3616.55	34
H25	4496.03	10027.12	2820.44	38
H26	3373.17	7490.51	2663.59	35
H2BA	5763.86	663.98	8342.01	31
H2BB	2858.78	137.43	8104.38	31
H3B	7373.14	-245.91	7493.69	36
H4B	6784.71	-3075.32	7379.15	36
H5BA	3908.2	-3819.44	8195.16	35
H5BB	2220.23	-2448.15	7845.44	35
H6BA	7993.2	-3112.31	8495.55	36
H6BB	8793.63	-1306.28	8324.18	36
H8B	7090.42	15.66	9328.04	33
H9B	6221.17	277.81	10335.02	39
H11B	3637.25	-311.97	11151.82	40
H13B	-2223.24	-3156.48	10857.1	43

Atom	x	y	z	U_{eq}
H14B	-1185.15	-3486.62	9856.67	41
H16B	1362.4	-2912.34	9041.46	32
H17B	2372.16	-1138.87	7113.43	33
H19C	3408.28	-2744.54	5369.62	33
H19D	6117.37	-1868.43	5542.98	33
H22B	23.49	447.16	6549.16	34
H23B	-1048.8	2939.06	6355.06	38
H25B	5456.55	4399.17	7151.14	37
H26B	6547.1	1900.02	7326.19	33

8.2 X-ray Crystallographic Data of 8a

R_1=7.63%

Submitted by: **Ziyi Chen, Davies Lab**

Data collected by: **John Bacsa**

Crystal Data and Experimental

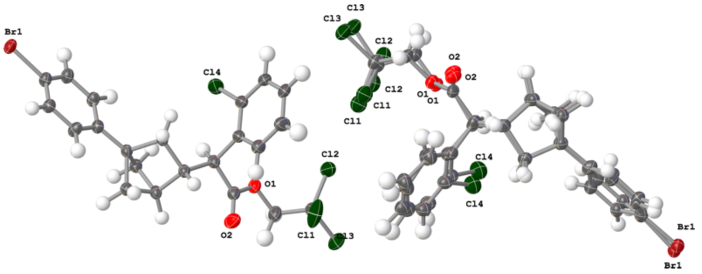

Experimental. Single colorless needle-shaped crystals of zc-02-217a were chosen from the sample as supplied. A suitable crystal with dimensions 0.20 × 0.08 × 0.02 mm³ was selected and mounted on a loop with paratone on a XtaLAB Synergy, Dualflex, HyPix diffractometer. The crystal was kept at a steady *T* = 100.00(11) K during data collection. The structure was solved with the ShelXT (Sheldrick, 2015) solution program and Olex2 1.5-alpha (Dolomanov et al., 2009). The model was refined with ShelXL 2018/3 (Sheldrick, 2015) using full matrix least squares minimisation on F^2. Crystallographic data of these structures, including cif, res, fcf, and hkl files, have been deposited with the Cambridge Crystallographic Data Centre with deposition numbers **2441163**. Copies of these data can be requested, free of charge, from the CCDC website at https://www.ccdc.cam.ac.uk/structures/.

Crystal Data. Identification code: 2441163, $C_{22}H_{19}BrCl_4O_2$, M_r = 537.08, triclinic, *P*1 (No. 1), a = 5.8872(2) Å, b = 10.6766(5) Å, c = 19.1046(8) Å, α = 100.426(4)°, β = 98.761(3)°, γ = 104.307(4)°, *V* = 1119.64(8) Å³, *T* = 100.00(11) K, *Z* = 2, *Z'* = 2, μ(Cu K$_\alpha$) = 7.031, 17910 reflections measured, 6589 unique (R_{int} = 0.0519) which were used in all calculations. The final wR_2 was 0.2029 (all data) and R_1 was 0.0763 (I≥2 σ(I)).

**Compound**	zc-02-217a
Formula	$C_{22}H_{19}BrCl_4O_2$
$D_{calc.}$/ g cm^{-3}	1.593
μ/mm^{-1}	7.031
Formula Weight	537.08
Color	colorless
Shape	needle-shaped
Size/mm³	0.20×0.08×0.02
T/K	100.00(11)
Crystal System	triclinic
Flack Parameter	-0.01(3)
Hooft Parameter	-0.038(16)
Space Group	*P*1
a/Å	5.8872(2)
b/Å	10.6766(5)
c/Å	19.1046(8)
α/°	100.426(4)
β/°	98.761(3)
γ/°	104.307(4)
V/Å³	1119.64(8)
Z	2
Z'	2
Wavelength/Å	1.54184
Radiation type	Cu K$_\alpha$
Θ_{min}/°	4.498
Θ_{max}/°	76.892
Measured Refl's.	17910
Indep't Refl's	6589
Refl's I≥2 σ(I)	5944
R_{int}	0.0519
Parameters	575
Restraints	1008
Largest Peak	2.343
Deepest Hole	-1.155
GooF	1.043
wR_2 (all data)	0.2029
wR_2	0.1974
R_1 (all data)	0.0825
R_1	0.0763

Structure Quality Indicators

**Reflections:**	d min (CuKα) 2Θ=153.8° 0.79	I/σ(I) 19.0	Rint m=2.72 5.19%	Full 135.4° 94% to 153.8° 98.5
**Refinement:**	Shift 0.000	Max Peak 2.3	Min Peak -1.2	GooF 1.043	Hooft -.01(3)

A colourless needle-shaped-shaped crystal with dimensions 0.20 × 0.08 × 0.02 mm^3 was mounted on a loop with paratone. Data were collected using a XtaLAB Synergy, Dualflex, HyPix diffractometer equipped with an Oxford Cryosystems low-temperature device operating at T = 100.00(11) K.

Data were measured using ω scans with Cu K_α radiation. The diffraction pattern was indexed and the total number of runs and images was based on the strategy calculation from the program CrysAlisPro system (CCD 44.57a 64-bit (release 20-06-2024)). The maximum resolution that was achieved was Θ = 76.892° (0.83 Å).

The unit cell was refined using CrysAlisPro 1.171.44.57a (Rigaku OD, 2024) on 5931 reflections, 33% of the observed reflections.

Data reduction, scaling and absorption corrections were performed using CrysAlisPro 1.171.44.57a (Rigaku OD, 2024). The final completeness is 98.50 % out to 76.892° in Θ. A numerical absorption correction based on gaussian integration over a multifaceted crystal model was performed using CrysAlisPro 1.171.42.74a (Rigaku Oxford Diffraction, 2022). An empirical absorption correction using spherical harmonics, implemented in SCALE3 ABSPACK scaling algorithm was also applied. The absorption coefficient μ of this material is 7.031 mm^{-1} at this wavelength (λ = 1.54184Å) and the minimum and maximum transmissions are 0.297 and 1.000.

The structure was solved and the space group P1 (# 1) determined by the ShelXT (Sheldrick, 2015) structure solution program and refined by full matrix least squares minimisation on $\boldsymbol{F^2}$ using version 2018/3 of ShelXL 2018/3 (Sheldrick, 2015). All non-hydrogen atoms were refined anisotropically. Hydrogen atom positions were calculated geometrically and refined using the riding model.

The value of Z' is 2. This means that there are two independent molecules in the asymmetric unit. The moiety formula is C22 H19 Br Cl4 O2.

The Flack parameter was refined to -0.01(3). Determination of absolute structure using Bayesian statistics on Bijvoet differences using the Olex2 results in -0.038(16). The chiral atoms in this structure are: C3_1(S), C3_2(S), C3_3(S), C13_1(S), C13_2(S), C13_3(S). Note: The Flack parameter is used to determine chirality of the crystal studied, the value should be near 0, a value of 1 means that the stereochemistry is wrong and the model should be inverted. A value of 0.5 means that the crystal consists of a racemic mixture of the two enantiomers.

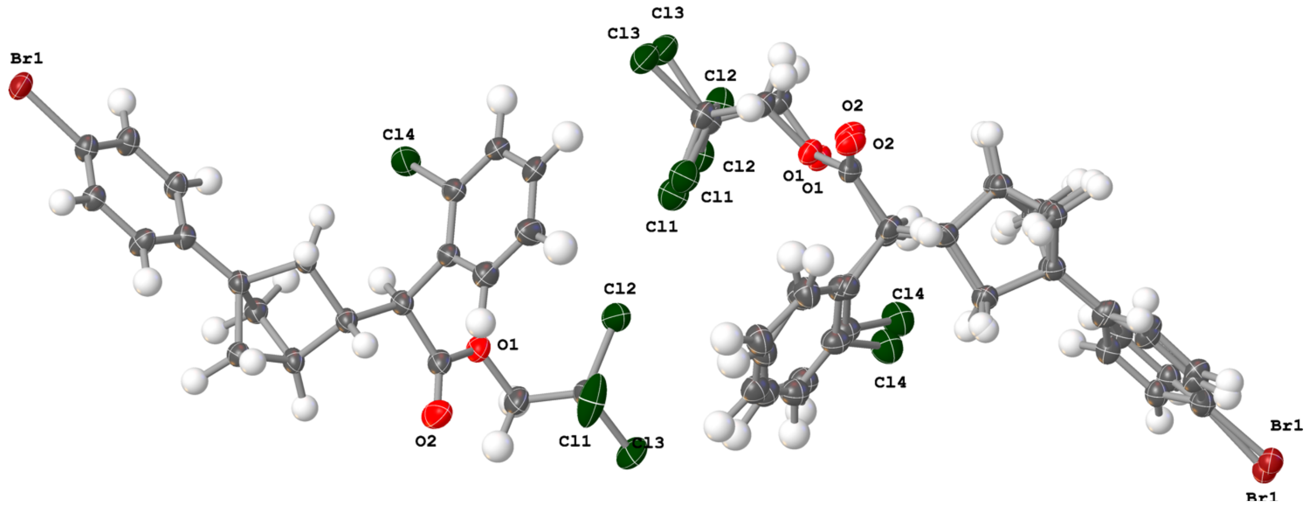

Figure 7 Thermal ellipsoidal (50% probability) representation of the asymmetric unit. This compound crystallizes with two independent molecules in the asymmetric unit. One of these molecules' disorders, while the other does not. It is interesting to note that two chemically identical molecules have been found to crystallize in two different (asymmetric) positions. Clearly one position gives optimal contacts while the second less so, and the molecule disorders. This observation is a result of the rigid constraints of translational symmetry together with the chiral, asymmetric shape of the molecule. The structure is chiral, space group P1, and the chiral atoms in this structure are: C3(S) and C13(S).

Data Plots: Diffraction Data

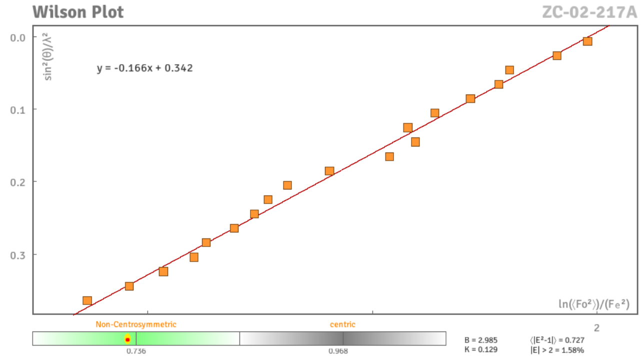

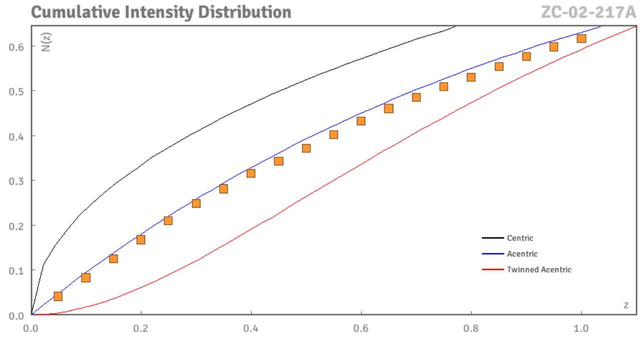

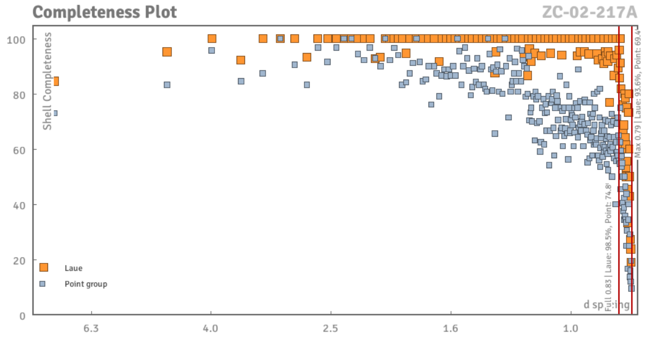

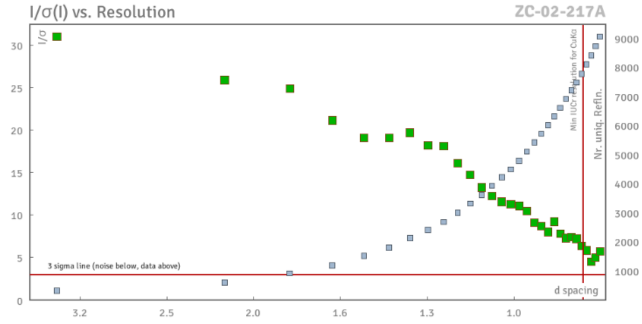

Data Plots: Refinement and Data

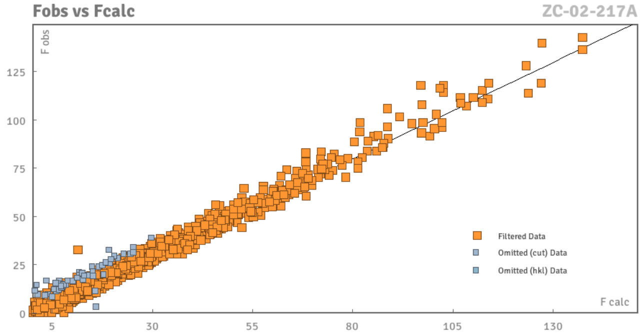

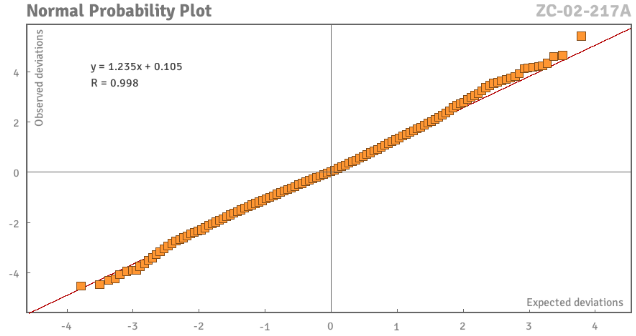

Reflection Statistics

Total reflections (after filtering)	17910	Unique reflections	6589
Completeness	0.694	Mean I/σ	14.39
hkl_{max} collected	(7, 13, 22)	hkl_{min} collected	(-7, -13, -23)
hkl_{max} used	(7, 13, 22)	hkl_{min} used	(-7, -13, -23)
Lim d_{max} collected	100.0	Lim d_{min} collected	0.77
d_{max} used	9.83	d_{min} used	0.79
Friedel pairs	2171	Friedel pairs merged	0
Inconsistent equivalents	7	R_{int}	0.0519
R_{sigma}	0.0526	Intensity transformed	0
Omitted reflections	0	Omitted by user (OMIT hkl)	143
Multiplicity	(2180, 1610, 1140, 763, 422, 240, 117, 62, 36, 19, 27, 15, 13, 10, 0, 1)	Maximum multiplicity	16
Removed systematic absences	0	Filtered off (Shel/OMIT)	0

There are no images if the crystal on the diffractometer, but the inclusion of these images has been requested from the GUI. Please unitck the relevant box if you don't have these images!

Table 7: Fractional Atomic Coordinates (×10⁴) and Equivalent Isotropic Displacement Parameters ($Å^2×10^3$) for zc-02-217a. U_{eq} is defined as 1/3 of the trace of the orthogonalised U_{ij}.

Atom	x	y	z	U_{eq}
Br1_1	13182(6)	17559.3(19)	9243.9(14)	35.2(3)
C1_1	9936(17)	11440(7)	8697(4)	28.0(16)
C2_1	9845(16)	10710(8)	7898(4)	28.0(19)
C3_1	8852(15)	9267(8)	7977(4)	28.7(17)
C4_1	8666(17)	9515(7)	8767(4)	28.4(17)
C5_1	7512(16)	10671(8)	8827(5)	32(2)
C6_1	11094(16)	10582(8)	9127(5)	30.6(19)
C7_1	10772(16)	12923(7)	8836(5)	29.0(19)
C8_1	13103(16)	13639(8)	9175(7)	34(2)
C9_1	13861(10)	15015(6)	9306(6)	32(2)
C10_1	12200(6)	15676(6)	9102(5)	30(2)
C11_1	9831(9)	15002(7)	8772(6)	34(2)
C12_1	9143(16)	13629(8)	8655(6)	32(2)
C13_1	6738(16)	8457(8)	7410(5)	32.1(18)
C14_1	5633(18)	7085(9)	7519(5)	32.4(19)
C15_1	2290(20)	5168(9)	7213(6)	44(3)

Atom	x	y	z	U_{eq}
C16_1	2120(20)	4242(10)	6504(6)	45(3)
C17_1	6788(15)	8403(8)	6643(4)	30.1(18)
C18_1	5760(20)	9097(10)	6192(5)	33(2)
C19_1	6000(20)	9001(12)	5476(6)	39(2)
C20_1	7360(20)	8245(12)	5204(6)	43(2)
C21_1	8510(30)	7583(13)	5644(6)	44(3)
C22_1	8250(20)	7685(12)	6355(6)	38(2)
Cl1_1	4986(8)	4201(4)	6371(3)	74.0(15)
Cl2_1	569(9)	4647(4)	5770(2)	57.9(10)
Cl3_1	477(9)	2626(3)	6565(2)	63.6(11)
Cl4_1	4062(8)	10116(4)	6520(2)	44.2(7)
O1_1	3339(13)	6512(6)	7173(4)	35.1(17)
O2_1	6800(19)	6502(8)	7851(6)	53(3)
Br1_3	-3695(8)	-7532(2)	839(2)	33.9(5)
C1_3	-307(16)	-1428(7)	1293(4)	29.8(12)
C2_3	1111(16)	-716(8)	2088(4)	29.7(18)
C3_3	1515(15)	733(7)	2004(4)	32.6(16)
C4_3	315(18)	507(7)	1218(4)	30.2(14)
C5_3	-2031(16)	-577(8)	1167(5)	30.9(15)
C6_3	1266(17)	-634(8)	853(5)	31.2(15)
C7_3	-1109(13)	-2911(7)	1160(3)	28.1(15)
C8_3	-43(14)	-3717(8)	753(4)	28.0(15)
C9_3	-770(10)	-5090(6)	650(4)	30.4(14)
C10_3	-2631(4)	-5653(3)	964.9(15)	30.6(13)
C11_3	-3764(10)	-4885(6)	1376(4)	30.4(14)
C12_3	-2986(15)	-3520(8)	1466(5)	28.0(15)
C13_3	872(16)	1624(8)	2579(5)	36.2(15)
C14_3	1730(20)	3085(9)	2553(5)	37(2)
C15_3	1270(20)	5243(9)	2857(6)	35(2)
C16_3	660(20)	5944(10)	3528(6)	37(3)
C17_3	1431(16)	1509(9)	3335(4)	30.0(16)
C18_3	-130(20)	896(19)	3732(6)	33.6(19)
C19_3	610(30)	910(20)	4460(6)	36(3)
C20_3	2920(20)	1540(20)	4802(7)	34(3)
C21_3	4520(30)	2190(20)	4430(7)	37(3)
C22_3	3760(20)	2200(20)	3712(6)	38(3)
Cl1_3	1881(12)	5459(6)	4295(3)	48.3(12)
Cl2_3	-2403(9)	5683(6)	3451(4)	47.5(10)
Cl3_3	1997(12)	7682(4)	3622(3)	51.3(12)
Cl4_3	-3061(11)	-27(9)	3305(4)	60.5(16)
O1_3	320(20)	3829(7)	2767(6)	37.6(19)
O2_3	3230(30)	3449(9)	2200(9)	44(3)
Br1_2	-3827(10)	-7543(2)	721(3)	33.9(5)
C1_2	-307(16)	-1428(7)	1293(4)	29.8(12)
C2_2	1111(16)	-716(8)	2088(4)	29.7(18)
C3_2	1515(15)	733(7)	2004(4)	32.6(16)
C4_2	315(18)	507(7)	1218(4)	30.2(14)
C5_2	-2031(16)	-577(8)	1167(5)	30.9(15)
C6_2	1266(17)	-634(8)	853(5)	31.2(15)
C7_2	-1109(13)	-2911(7)	1160(3)	28.1(15)
C8_2	302(16)	-3688(8)	912(5)	28.0(15)
C9_2	-467(10)	-5063(6)	775(5)	30.4(14)
C10_2	-2711(6)	-5659(4)	895(2)	30.6(13)
C11_2	-4192(10)	-4921(6)	1141(5)	30.4(14)
C12_2	-3358(16)	-3549(8)	1272(5)	28.0(15)
C13_2	872(16)	1624(8)	2579(5)	36.2(15)
C14_2	800(20)	2961(9)	2445(5)	37(2)
C15_2	-520(20)	4877(10)	2771(6)	35(2)

Atom	x	y	z	U_{eq}
C16_2	-30(20)	5693(11)	3532(6)	37(3)
C17_2	1773(16)	1706(9)	3344(4)	30.0(16)
C18_2	610(20)	1040(15)	3814(6)	33.6(19)
C19_2	1750(30)	1091(18)	4513(6)	36(3)
C20_2	4090(30)	1813(18)	4758(7)	34(3)
C21_2	5320(30)	2490(20)	4310(7)	37(3)
C22_2	4170(20)	2436(16)	3617(6)	38(3)
Cl1_2	2610(11)	5564(7)	4042(4)	48.3(12)
Cl2_2	-2398(11)	5273(6)	3965(4)	47.5(10)
Cl3_2	372(15)	7382(5)	3461(4)	51.3(12)
Cl4_2	-2345(14)	37(11)	3516(5)	60.5(16)
O1_2	-695(19)	3517(8)	2782(6)	37.6(19)
O2_2	2290(30)	3613(10)	2174(11)	44(3)

Table 8: Anisotropic Displacement Parameters (×10[4]) for zc-02-217a. The anisotropic displacement factor exponent takes the form: $-2\pi^2[h^2a^{*2} \times U_{11} + ... + 2hka^* \times b^* \times U_{12}]$

Atom	U_{11}	U_{22}	U_{33}	U_{23}	U_{13}	U_{12}
Br1_1	40.6(7)	22.1(6)	39.7(7)	2.3(5)	8.5(5)	6.2(5)
C1_1	25(3)	28(2)	26(3)	2(2)	4(2)	3.6(18)
C2_1	31(5)	23(3)	26(3)	4(2)	4(3)	3(2)
C3_1	30(3)	24(3)	29(3)	4.6(18)	4(2)	4(2)
C4_1	23(3)	28(3)	29(3)	4(2)	2(2)	1(3)
C5_1	26(3)	31(3)	35(5)	7(3)	6(3)	5(2)
C6_1	24(3)	29(3)	33(4)	5(2)	0(3)	1(3)
C7_1	23(3)	28(2)	33(5)	3(2)	8(3)	4.4(17)
C8_1	25(3)	26(3)	47(6)	8(3)	2(3)	3.6(18)
C9_1	25(3)	26(3)	43(6)	8(3)	6(3)	3.9(19)
C10_1	30(3)	28(4)	29(5)	2.0(19)	5(3)	7.7(18)
C11_1	30(3)	31(3)	36(5)	0(3)	4(3)	8(2)
C12_1	27(3)	31(3)	35(6)	0(3)	4(3)	8(2)
C13_1	33(4)	25(3)	32(2)	5.9(18)	1(2)	1(2)
C14_1	37(3)	24(3)	31(4)	4(2)	7(3)	2(2)
C15_1	56(7)	25(3)	43(4)	7(2)	6(4)	-2(2)
C16_1	52(4)	33(4)	41(4)	5(2)	-2(3)	8(3)
C17_1	33(4)	22(4)	31(2)	5(2)	1(2)	4(3)
C18_1	37(4)	25(4)	34(3)	7(2)	0(3)	6(4)
C19_1	52(6)	27(5)	35(3)	7(3)	4(3)	8(4)
C20_1	55(5)	29(5)	42(4)	7(4)	7(4)	11(4)
C21_1	60(7)	35(6)	40(4)	9(3)	12(3)	18(5)
C22_1	44(5)	33(5)	39(4)	8(3)	7(4)	14(4)
Cl1_1	33.3(16)	52(2)	113(4)	-30(2)	13.8(18)	5.0(14)
Cl2_1	74(2)	35.0(17)	47.6(19)	5.9(14)	-17.8(17)	6.1(16)
Cl3_1	88(3)	25.6(15)	63(2)	11.3(14)	11(2)	-8.8(16)
Cl4_1	47.5(17)	44.9(17)	41.8(16)	7.0(13)	1.6(13)	22.9(14)
O1_1	38(3)	24(3)	38(4)	6(2)	5(3)	1(2)
O2_1	57(5)	30(4)	59(5)	9(4)	-12(4)	5(4)
Br1_3	34.6(6)	29.7(6)	34.2(9)	3.6(4)	3.5(6)	8.1(4)
C1_3	29.7(18)	29.7(13)	29(2)	4.3(11)	5.4(14)	9.4(10)
C2_3	34(4)	25(2)	30(2)	4.6(14)	4(2)	11(2)
C3_3	41(3)	25(2)	30.0(17)	4.4(12)	2.7(16)	11(2)
C4_3	30.5(19)	30.9(18)	29.3(18)	5.9(12)	5.8(14)	10.0(14)
C5_3	30.4(18)	30.7(17)	32(3)	5.8(15)	6.3(14)	10.2(14)
C6_3	31(2)	31.2(18)	32(2)	6.0(13)	6.9(19)	10.2(14)
C7_3	23(3)	29.7(13)	31(4)	4.2(11)	6(3)	9.3(10)
C8_3	23(3)	29.9(13)	31(4)	4.2(11)	5(3)	9.4(10)

Atom	U_{11}	U_{22}	U_{33}	U_{23}	U_{13}	U_{12}
C9_3	30(2)	29.9(13)	30(3)	4.1(10)	5(2)	8.9(9)
C10_3	30.2(19)	30.0(14)	30(2)	4.4(10)	5.0(15)	9.2(9)
C11_3	30(2)	29.9(13)	30(3)	4.1(10)	5(2)	8.9(9)
C12_3	23(3)	29.9(13)	31(4)	4.2(11)	5(3)	9.4(10)
C13_3	49(3)	26(2)	32.4(16)	4.3(12)	6.3(15)	13(2)
C14_3	49(4)	26(2)	34(4)	4(2)	1(3)	12(2)
C15_3	44(4)	21(3)	40(3)	5(2)	9(2)	8(2)
C16_3	40(4)	26(4)	41(3)	1.4(17)	8.9(19)	6.4(19)
C17_3	29(2)	28(2)	32.5(16)	5.7(13)	8.0(13)	6.9(14)
C18_3	33(2)	31(3)	36(2)	7.4(18)	10.8(16)	5.9(17)
C19_3	35(4)	32(7)	37(2)	9(2)	9(2)	2(4)
C20_3	35(4)	32(6)	32(4)	7(4)	11(2)	3(4)
C21_3	33(4)	42(8)	30(3)	10(4)	6(2)	-2(4)
C22_3	31(2)	49(6)	30(3)	11(3)	7.0(19)	0(2)
Cl1_3	47(3)	45(2)	50(3)	9(2)	9.3(18)	10(2)
Cl2_3	42(2)	43(3)	53(3)	5.6(19)	11.1(17)	7.2(16)
Cl3_3	71(3)	27(2)	51(3)	6.0(17)	13(3)	8.0(19)
Cl4_3	24(3)	65(2)	66(4)	-19(3)	10(2)	-10(2)
O1_3	48(4)	21(3)	41(4)	5(2)	5(3)	8(3)
O2_3	58(6)	27(3)	48(5)	9(3)	13(5)	16(3)
Br1_2	34.6(6)	29.7(6)	34.2(9)	3.6(4)	3.5(6)	8.1(4)
C1_2	29.7(18)	29.7(13)	29(2)	4.3(11)	5.4(14)	9.4(10)
C2_2	34(4)	25(2)	30(2)	4.6(14)	4(2)	11(2)
C3_2	41(3)	25(2)	30.0(17)	4.4(12)	2.7(16)	11(2)
C4_2	30.5(19)	30.9(18)	29.3(18)	5.9(12)	5.8(14)	10.0(14)
C5_2	30.4(18)	30.7(17)	32(3)	5.8(15)	6.3(14)	10.2(14)
C6_2	31(2)	31.2(18)	32(2)	6.0(13)	6.9(19)	10.2(14)
C7_2	23(3)	29.7(13)	31(4)	4.2(11)	6(3)	9.3(10)
C8_2	23(3)	29.9(13)	31(4)	4.2(11)	5(3)	9.4(10)
C9_2	30(2)	29.9(13)	30(3)	4.1(10)	5(2)	8.9(9)
C10_2	30.2(19)	30.0(14)	30(2)	4.4(10)	5.0(15)	9.2(9)
C11_2	30(2)	29.9(13)	30(3)	4.1(10)	5(2)	8.9(9)
C12_2	23(3)	29.9(13)	31(4)	4.2(11)	5(3)	9.4(10)
C13_2	49(3)	26(2)	32.4(16)	4.3(12)	6.3(15)	13(2)
C14_2	49(4)	26(2)	34(4)	4(2)	1(3)	12(2)
C15_2	44(4)	21(3)	40(3)	5(2)	9(2)	8(2)
C16_2	40(4)	26(4)	41(3)	1.4(17)	8.9(19)	6.4(19)
C17_2	29(2)	28(2)	32.5(16)	5.7(13)	8.0(13)	6.9(14)
C18_2	33(2)	31(3)	36(2)	7.4(18)	10.8(16)	5.9(17)
C19_2	35(4)	32(7)	37(2)	9(2)	9(2)	2(4)
C20_2	35(4)	32(6)	32(4)	7(4)	11(2)	3(4)
C21_2	33(4)	42(8)	30(3)	10(4)	6(2)	-2(4)
C22_2	31(2)	49(6)	30(3)	11(3)	7.0(19)	0(2)
Cl1_2	47(3)	45(2)	50(3)	9(2)	9.3(18)	10(2)
Cl2_2	42(2)	43(3)	53(3)	5.6(19)	11.1(17)	7.2(16)
Cl3_2	71(3)	27(2)	51(3)	6.0(17)	13(3)	8.0(19)
Cl4_2	24(3)	65(2)	66(4)	-19(3)	10(2)	-10(2)
O1_2	48(4)	21(3)	41(4)	5(2)	5(3)	8(3)
O2_2	58(6)	27(3)	48(5)	9(3)	13(5)	16(3)

Table 9: Bond Lengths in Å for zc-02-217a.

Atom	Atom	Length/Å
Br1_1	C10_1	1.905(5)
C1_1	C2_1	1.569(9)
C1_1	C5_1	1.542(11)
C1_1	C6_1	1.546(11)
C1_1	C7_1	1.498(9)
C2_1	H2A_1	1.0980

Atom	Atom	Length/Å
C2_1	H2B_1	1.0980
C2_1	C3_1	1.550(10)
C3_1	H3_1	1.0980
C3_1	C4_1	1.509(10)
C3_1	C13_1	1.480(11)
C4_1	H4_1	1.0980
C4_1	C5_1	1.544(11)
C4_1	C6_1	1.558(10)
C5_1	H5A_1	1.0980
C5_1	H5B_1	1.0980
C6_1	H6A_1	1.0980
C6_1	H6B_1	1.0980
C7_1	C8_1	1.385(11)
C7_1	C12_1	1.400(11)
C8_1	H8_1	1.0780
C8_1	C9_1	1.388(10)
C9_1	H9_1	1.0780
C9_1	C10_1	1.390(5)
C10_1	C11_1	1.390(5)
C11_1	H11_1	1.0780
C11_1	C12_1	1.386(10)
C12_1	H12_1	1.0780
C13_1	H13_1	1.0980
C13_1	C14_1	1.514(11)
C13_1	C17_1	1.460(11)
C14_1	O1_1	1.345(12)
C14_1	O2_1	1.219(12)
C15_1	H15B_1	1.0980
C15_1	H15A_1	1.0980
C15_1	C16_1	1.500(13)
C15_1	O1_1	1.436(11)
C16_1	Cl1_1	1.751(11)
C16_1	Cl2_1	1.736(11)
C16_1	Cl3_1	1.793(10)
C17_1	C18_1	1.397(11)
C17_1	C22_1	1.398(12)
C18_1	C19_1	1.387(13)
C18_1	Cl4_1	1.747(10)
C19_1	H19_1	1.0780
C19_1	C20_1	1.362(15)
C20_1	H20_1	1.0780
C20_1	C21_1	1.392(15)
C21_1	H21_1	1.0780
C21_1	C22_1	1.377(13)
C22_1	H22_1	1.0780
Br1_3	C10_3	1.906(5)
C1_3	C2_3	1.570(9)
C1_3	C5_3	1.541(11)
C1_3	C6_3	1.545(11)
C1_3	C7_3	1.498(9)
C2_3	H2A_3	1.0980
C2_3	H2B_3	1.0980
C2_3	C3_3	1.548(10)
C3_3	H3_3	1.0980
C3_3	C4_3	1.507(10)
C3_3	C13_3	1.478(11)
C4_3	H4_3	1.0980
C4_3	C5_3	1.544(11)

Atom	Atom	Length/Å
C4_3	C6_3	1.559(10)
C5_3	H5A_3	1.0980
C5_3	H5B_3	1.0980
C6_3	H6A_3	1.0980
C6_3	H6B_3	1.0980
C7_3	C8_3	1.383(11)
C7_3	C12_3	1.402(11)
C8_3	H8_3	1.0780
C8_3	C9_3	1.390(10)
C9_3	H9_3	1.0780
C9_3	C10_3	1.390(5)
C10_3	C11_3	1.390(5)
C11_3	H11_3	1.0780
C11_3	C12_3	1.385(10)
C12_3	H12_3	1.0780
C13_3	H13_3	1.0980
C13_3	C14_3	1.529(11)
C13_3	C17_3	1.465(11)
C14_3	O1_3	1.343(12)
C14_3	O2_3	1.221(12)
C15_3	H15A_3	1.0980
C15_3	H15B_3	1.0980
C15_3	C16_3	1.501(13)
C15_3	O1_3	1.442(11)
C16_3	Cl1_3	1.751(11)
C16_3	Cl2_3	1.736(12)
C16_3	Cl3_3	1.791(10)
C17_3	C18_3	1.394(12)
C17_3	C22_3	1.398(12)
C18_3	C19_3	1.388(13)
C18_3	Cl4_3	1.748(10)
C19_3	H19_3	1.0780
C19_3	C20_3	1.361(15)
C20_3	H20_3	1.0780
C20_3	C21_3	1.392(15)
C21_3	H21_3	1.0780
C21_3	C22_3	1.378(13)
C22_3	H22_3	1.0780
Br1_2	C10_2	1.904(5)
C1_2	C2_2	1.570(9)
C1_2	C5_2	1.541(11)
C1_2	C6_2	1.545(11)
C1_2	C7_2	1.498(9)
C2_2	H2A_2	1.0980
C2_2	H2B_2	1.0980
C2_2	C3_2	1.548(10)
C3_2	H3_2	1.0980
C3_2	C4_2	1.507(10)
C3_2	C13_2	1.478(11)
C4_2	H4_2	1.0980
C4_2	C5_2	1.544(11)
C4_2	C6_2	1.559(10)
C5_2	H5A_2	1.0980
C5_2	H5B_2	1.0980
C6_2	H6A_2	1.0980
C6_2	H6B_2	1.0980
C7_2	C8_2	1.386(11)
C7_2	C12_2	1.398(11)

Atom	Atom	Length/Å
C8_2	H8_2	1.0780
C8_2	C9_2	1.387(10)
C9_2	H9_2	1.0780
C9_2	C10_2	1.390(5)
C10_2	C11_2	1.390(5)
C11_2	H11_2	1.0780
C11_2	C12_2	1.387(10)
C12_2	H12_2	1.0780
C13_2	H13_2	1.0980
C13_2	C14_2	1.505(11)
C13_2	C17_2	1.456(11)
C14_2	O1_2	1.352(12)
C14_2	O2_2	1.223(12)
C15_2	H15A_2	1.0980
C15_2	H15B_2	1.0980
C15_2	C16_2	1.499(13)
C15_2	O1_2	1.435(11)
C16_2	Cl1_2	1.752(11)
C16_2	Cl2_2	1.736(12)
C16_2	Cl3_2	1.794(11)
C17_2	C18_2	1.398(12)
C17_2	C22_2	1.400(12)
C18_2	C19_2	1.387(13)
C18_2	Cl4_2	1.748(10)
C19_2	H19_2	1.0780
C19_2	C20_2	1.361(15)
C20_2	H20_2	1.0780
C20_2	C21_2	1.393(15)
C21_2	H21_2	1.0780
C21_2	C22_2	1.376(13)
C22_2	H22_2	1.0780

Table 10: Bond Angles in ° for zc-02-217a.

Atom	Atom	Atom	Angle/°
C5_1	C1_1	C2_1	101.3(6)
C5_1	C1_1	C6_1	87.2(6)
C6_1	C1_1	C2_1	100.2(6)
C7_1	C1_1	C2_1	114.1(5)
C7_1	C1_1	C5_1	123.4(6)
C7_1	C1_1	C6_1	125.1(7)
C1_1	C2_1	H2A_1	112.2
C1_1	C2_1	H2B_1	112.2
H2A_1	C2_1	H2B_1	109.8
C3_1	C2_1	C1_1	97.8(6)
C3_1	C2_1	H2A_1	112.2
C3_1	C2_1	H2B_1	112.2
C2_1	C3_1	H3_1	107.0
C4_1	C3_1	C2_1	100.6(6)
C4_1	C3_1	H3_1	107.0
C13_1	C3_1	C2_1	114.5(7)
C13_1	C3_1	H3_1	107.0
C13_1	C3_1	C4_1	119.9(6)
C3_1	C4_1	H4_1	120.3
C3_1	C4_1	C5_1	101.6(6)
C3_1	C4_1	C6_1	101.3(6)
C5_1	C4_1	H4_1	120.3
C5_1	C4_1	C6_1	86.7(5)
C6_1	C4_1	H4_1	120.3
C1_1	C5_1	C4_1	82.9(6)
C1_1	C5_1	H5A_1	114.8
C1_1	C5_1	H5B_1	114.8
C4_1	C5_1	H5A_1	114.8
C4_1	C5_1	H5B_1	114.8
H5A_1	C5_1	H5B_1	111.9
C1_1	C6_1	C4_1	82.3(6)
C1_1	C6_1	H6A_1	114.9
C1_1	C6_1	H6B_1	114.9
C4_1	C6_1	H6A_1	114.9
C4_1	C6_1	H6B_1	114.9
H6A_1	C6_1	H6B_1	112.0
C8_1	C7_1	C1_1	122.1(7)
C8_1	C7_1	C12_1	118.1(7)
C12_1	C7_1	C1_1	119.7(7)
C7_1	C8_1	H8_1	119.1
C7_1	C8_1	C9_1	121.7(7)
C9_1	C8_1	H8_1	119.1
C8_1	C9_1	H9_1	120.8
C8_1	C9_1	C10_1	118.3(6)
C10_1	C9_1	H9_1	120.8
C9_1	C10_1	Br1_1	119.8(4)
C11_1	C10_1	Br1_1	118.2(4)
C11_1	C10_1	C9_1	122.0(5)
C10_1	C11_1	H11_1	121.1
C12_1	C11_1	C10_1	117.9(6)
C12_1	C11_1	H11_1	121.1
C7_1	C12_1	H12_1	119.0
C11_1	C12_1	C7_1	121.9(7)
C11_1	C12_1	H12_1	119.0
C3_1	C13_1	H13_1	103.1
C3_1	C13_1	C14_1	114.7(7)
C14_1	C13_1	H13_1	103.1
C17_1	C13_1	C3_1	119.9(6)
C17_1	C13_1	H13_1	103.1
C17_1	C13_1	C14_1	110.4(6)
O1_1	C14_1	C13_1	115.5(8)
O2_1	C14_1	C13_1	122.1(8)
O2_1	C14_1	O1_1	122.2(8)
H15B_1	C15_1	H15A_1	108.2
C16_1	C15_1	H15B_1	109.7
C16_1	C15_1	H15A_1	109.7
O1_1	C15_1	H15B_1	109.7
O1_1	C15_1	H15A_1	109.7
O1_1	C15_1	C16_1	109.7(8)
C15_1	C16_1	Cl1_1	110.5(7)
C15_1	C16_1	Cl2_1	112.5(8)
C15_1	C16_1	Cl3_1	106.5(7)
Cl1_1	C16_1	Cl3_1	109.1(6)
Cl2_1	C16_1	Cl1_1	110.5(6)

Atom	Atom	Atom	Angle/°
Cl2_1	C16_1	Cl3_1	107.7(5)
C18_1	C17_1	C13_1	127.5(8)
C18_1	C17_1	C22_1	116.6(8)
C22_1	C17_1	C13_1	115.5(7)
C17_1	C18_1	Cl4_1	120.3(7)
C19_1	C18_1	C17_1	122.2(9)
C19_1	C18_1	Cl4_1	117.5(7)
C18_1	C19_1	H19_1	120.3
C20_1	C19_1	C18_1	119.4(10)
C20_1	C19_1	H19_1	120.3
C19_1	C20_1	H20_1	119.8
C19_1	C20_1	C21_1	120.3(10)
C21_1	C20_1	H20_1	119.8
C20_1	C21_1	H21_1	120.1
C22_1	C21_1	C20_1	119.9(10)
C22_1	C21_1	H21_1	120.1
C17_1	C22_1	H22_1	119.3
C21_1	C22_1	C17_1	121.4(9)
C21_1	C22_1	H22_1	119.3
C14_1	O1_1	C15_1	117.0(8)
C5_3	C1_3	C2_3	101.2(6)
C5_3	C1_3	C6_3	87.2(6)
C6_3	C1_3	C2_3	100.2(6)
C7_3	C1_3	C2_3	113.9(5)
C7_3	C1_3	C5_3	123.5(6)
C7_3	C1_3	C6_3	125.1(7)
C1_3	C2_3	H2A_3	112.2
C1_3	C2_3	H2B_3	112.2
H2A_3	C2_3	H2B_3	109.8
C3_3	C2_3	C1_3	97.7(6)
C3_3	C2_3	H2A_3	112.2
C3_3	C2_3	H2B_3	112.2
C2_3	C3_3	H3_3	106.6
C4_3	C3_3	C2_3	100.8(6)
C4_3	C3_3	H3_3	106.6
C13_3	C3_3	C2_3	114.9(7)
C13_3	C3_3	H3_3	106.6
C13_3	C3_3	C4_3	120.5(6)
C3_3	C4_3	H4_3	120.3
C3_3	C4_3	C5_3	101.7(6)
C3_3	C4_3	C6_3	101.2(6)
C5_3	C4_3	H4_3	120.3
C5_3	C4_3	C6_3	86.6(5)
C6_3	C4_3	H4_3	120.3
C1_3	C5_3	C4_3	82.9(6)
C1_3	C5_3	H5A_3	114.8
C1_3	C5_3	H5B_3	114.8
C4_3	C5_3	H5A_3	114.8
C4_3	C5_3	H5B_3	114.8
H5A_3	C5_3	H5B_3	111.9
C1_3	C6_3	C4_3	82.3(5)
C1_3	C6_3	H6A_3	114.9
C1_3	C6_3	H6B_3	114.9
C4_3	C6_3	H6A_3	114.9
C4_3	C6_3	H6B_3	114.9
H6A_3	C6_3	H6B_3	112.0
C8_3	C7_3	C1_3	122.5(7)
C8_3	C7_3	C12_3	118.0(7)

Atom	Atom	Atom	Angle/°
C12_3	C7_3	C1_3	119.4(7)
C7_3	C8_3	H8_3	119.1
C7_3	C8_3	C9_3	121.8(7)
C9_3	C8_3	H8_3	119.1
C8_3	C9_3	H9_3	120.8
C8_3	C9_3	C10_3	118.3(6)
C10_3	C9_3	H9_3	120.8
C9_3	C10_3	Br1_3	119.9(4)
C9_3	C10_3	C11_3	121.9(5)
C11_3	C10_3	Br1_3	118.1(4)
C10_3	C11_3	H11_3	121.0
C12_3	C11_3	C10_3	117.9(6)
C12_3	C11_3	H11_3	121.0
C7_3	C12_3	H12_3	119.0
C11_3	C12_3	C7_3	121.9(7)
C11_3	C12_3	H12_3	119.0
C3_3	C13_3	H13_3	104.8
C3_3	C13_3	C14_3	112.8(7)
C14_3	C13_3	H13_3	104.8
C17_3	C13_3	C3_3	119.4(7)
C17_3	C13_3	H13_3	104.8
C17_3	C13_3	C14_3	108.9(6)
O1_3	C14_3	C13_3	115.1(8)
O2_3	C14_3	C13_3	120.6(8)
O2_3	C14_3	O1_3	121.9(8)
H15A_3	C15_3	H15B_3	108.3
C16_3	C15_3	H15A_3	109.9
C16_3	C15_3	H15B_3	109.9
O1_3	C15_3	H15A_3	109.9
O1_3	C15_3	H15B_3	109.9
O1_3	C15_3	C16_3	109.1(8)
C15_3	C16_3	Cl1_3	110.4(7)
C15_3	C16_3	Cl2_3	112.2(8)
C15_3	C16_3	Cl3_3	106.4(7)
Cl1_3	C16_3	Cl3_3	109.2(6)
Cl2_3	C16_3	Cl1_3	110.6(6)
Cl2_3	C16_3	Cl3_3	107.9(6)
C18_3	C17_3	C13_3	127.8(8)
C18_3	C17_3	C22_3	116.9(8)
C22_3	C17_3	C13_3	115.1(7)
C17_3	C18_3	Cl4_3	120.4(7)
C19_3	C18_3	C17_3	122.1(9)
C19_3	C18_3	Cl4_3	117.3(7)
C18_3	C19_3	H19_3	120.3
C20_3	C19_3	C18_3	119.4(10)
C20_3	C19_3	H19_3	120.3
C19_3	C20_3	H20_3	119.8
C19_3	C20_3	C21_3	120.4(10)
C21_3	C20_3	H20_3	119.8
C20_3	C21_3	H21_3	120.1
C22_3	C21_3	C20_3	119.8(10)
C22_3	C21_3	H21_3	120.1
C17_3	C22_3	H22_3	119.3
C21_3	C22_3	C17_3	121.3(9)
C21_3	C22_3	H22_3	119.3
C14_3	O1_3	C15_3	116.2(8)
C5_2	C1_2	C2_2	101.2(6)
C5_2	C1_2	C6_2	87.2(6)

Atom	Atom	Atom	Angle/°
C6_2	C1_2	C2_2	100.2(6)
C7_2	C1_2	C2_2	113.9(5)
C7_2	C1_2	C5_2	123.5(6)
C7_2	C1_2	C6_2	125.1(7)
C1_2	C2_2	H2A_2	112.2
C1_2	C2_2	H2B_2	112.2
H2A_2	C2_2	H2B_2	109.8
C3_2	C2_2	C1_2	97.7(6)
C3_2	C2_2	H2A_2	112.2
C3_2	C2_2	H2B_2	112.2
C2_2	C3_2	H3_2	106.6
C4_2	C3_2	C2_2	100.8(6)
C4_2	C3_2	H3_2	106.6
C13_2	C3_2	C2_2	114.9(7)
C13_2	C3_2	H3_2	106.6
C13_2	C3_2	C4_2	120.5(6)
C3_2	C4_2	H4_2	120.3
C3_2	C4_2	C5_2	101.7(6)
C3_2	C4_2	C6_2	101.2(6)
C5_2	C4_2	H4_2	120.3
C5_2	C4_2	C6_2	86.6(5)
C6_2	C4_2	H4_2	120.3
C1_2	C5_2	C4_2	82.9(6)
C1_2	C5_2	H5A_2	114.8
C1_2	C5_2	H5B_2	114.8
C4_2	C5_2	H5A_2	114.8
C4_2	C5_2	H5B_2	114.8
H5A_2	C5_2	H5B_2	111.9
C1_2	C6_2	C4_2	82.3(5)
C1_2	C6_2	H6A_2	114.9
C1_2	C6_2	H6B_2	114.9
C4_2	C6_2	H6A_2	114.9
C4_2	C6_2	H6B_2	114.9
H6A_2	C6_2	H6B_2	112.0
C8_2	C7_2	C1_2	121.8(7)
C8_2	C7_2	C12_2	118.2(7)
C12_2	C7_2	C1_2	120.0(7)
C7_2	C8_2	H8_2	119.2
C7_2	C8_2	C9_2	121.7(7)
C9_2	C8_2	H8_2	119.2
C8_2	C9_2	H9_2	120.8
C8_2	C9_2	C10_2	118.3(6)
C10_2	C9_2	H9_2	120.8
C9_2	C10_2	Br1_2	119.7(4)
C9_2	C10_2	C11_2	122.1(5)
C11_2	C10_2	Br1_2	118.2(4)

Atom	Atom	Atom	Angle/°
C10_2	C11_2	H11_2	121.1
C12_2	C11_2	C10_2	117.8(6)
C12_2	C11_2	H11_2	121.1
C7_2	C12_2	H12_2	119.1
C11_2	C12_2	C7_2	121.9(7)
C11_2	C12_2	H12_2	119.1
C3_2	C13_2	H13_2	101.4
C3_2	C13_2	C14_2	116.4(7)
C14_2	C13_2	H13_2	101.4
C17_2	C13_2	C3_2	120.7(6)
C17_2	C13_2	H13_2	101.4
C17_2	C13_2	C14_2	111.4(6)
O1_2	C14_2	C13_2	115.3(8)
O2_2	C14_2	C13_2	122.2(9)
O2_2	C14_2	O1_2	121.0(8)
H15A_2	C15_2	H15B_2	108.2
C16_2	C15_2	H15A_2	109.7
C16_2	C15_2	H15B_2	109.7
O1_2	C15_2	H15A_2	109.7
O1_2	C15_2	H15B_2	109.7
O1_2	C15_2	C16_2	110.0(8)
C15_2	C16_2	Cl1_2	110.5(7)
C15_2	C16_2	Cl2_2	112.6(8)
C15_2	C16_2	Cl3_2	106.4(7)
Cl1_2	C16_2	Cl3_2	109.0(6)
Cl2_2	C16_2	Cl1_2	110.5(6)
Cl2_2	C16_2	Cl3_2	107.6(6)
C18_2	C17_2	C13_2	127.8(8)
C18_2	C17_2	C22_2	116.5(8)
C22_2	C17_2	C13_2	115.4(7)
C17_2	C18_2	Cl4_2	120.3(7)
C19_2	C18_2	C17_2	122.3(9)
C19_2	C18_2	Cl4_2	117.3(7)
C18_2	C19_2	H19_2	120.3
C20_2	C19_2	C18_2	119.5(10)
C20_2	C19_2	H19_2	120.3
C19_2	C20_2	H20_2	119.9
C19_2	C20_2	C21_2	120.2(10)
C21_2	C20_2	H20_2	119.9
C20_2	C21_2	H21_2	120.0
C22_2	C21_2	C20_2	119.9(10)
C22_2	C21_2	H21_2	120.0
C17_2	C22_2	H22_2	119.2
C21_2	C22_2	C17_2	121.6(9)
C21_2	C22_2	H22_2	119.2
C14_2	O1_2	C15_2	116.8(8)

Table 11: Torsion Angles in ° for zc-02-217a.

Atom	Atom	Atom	Atom	Angle/°
Br1_1	C10_1	C11_1	C12_1	178.5(9)
C1_1	C2_1	C3_1	C4_1	-0.8(7)
C1_1	C2_1	C3_1	C13_1	129.2(7)
C1_1	C7_1	C8_1	C9_1	-179.7(9)
C1_1	C7_1	C12_1	C11_1	-179.9(9)
C2_1	C1_1	C5_1	C4_1	66.0(6)

Atom	Atom	Atom	Atom	Angle/°
C2_1	C1_1	C6_1	C4_1	-67.4(6)
C2_1	C1_1	C7_1	C8_1	-99.7(10)
C2_1	C1_1	C7_1	C12_1	83.5(10)
C2_1	C3_1	C4_1	C5_1	44.9(7)
C2_1	C3_1	C4_1	C6_1	-44.0(7)
C2_1	C3_1	C13_1	C14_1	-175.1(7)
C2_1	C3_1	C13_1	C17_1	49.8(10)
C3_1	C4_1	C5_1	C1_1	-67.3(6)
C3_1	C4_1	C6_1	C1_1	67.7(6)
C3_1	C13_1	C14_1	O1_1	158.6(8)
C3_1	C13_1	C14_1	O2_1	-26.8(13)
C3_1	C13_1	C17_1	C18_1	-100.8(11)
C3_1	C13_1	C17_1	C22_1	71.7(11)
C4_1	C3_1	C13_1	C14_1	-55.4(9)
C4_1	C3_1	C13_1	C17_1	169.5(7)
C5_1	C1_1	C2_1	C3_1	-43.5(7)
C5_1	C1_1	C6_1	C4_1	33.5(5)
C5_1	C1_1	C7_1	C8_1	136.8(10)
C5_1	C1_1	C7_1	C12_1	-39.9(11)
C5_1	C4_1	C6_1	C1_1	-33.5(5)
C6_1	C1_1	C2_1	C3_1	45.7(7)
C6_1	C1_1	C5_1	C4_1	-33.8(5)
C6_1	C1_1	C7_1	C8_1	23.8(11)
C6_1	C1_1	C7_1	C12_1	-153.0(10)
C6_1	C4_1	C5_1	C1_1	33.6(6)
C7_1	C1_1	C2_1	C3_1	-178.2(7)
C7_1	C1_1	C5_1	C4_1	-164.9(7)
C7_1	C1_1	C6_1	C4_1	163.3(6)
C7_1	C8_1	C9_1	C10_1	1.4(17)
C8_1	C7_1	C12_1	C11_1	3.2(16)
C8_1	C9_1	C10_1	Br1_1	-178.2(9)
C8_1	C9_1	C10_1	C11_1	-0.2(16)
C9_1	C10_1	C11_1	C12_1	0.5(16)
C10_1	C11_1	C12_1	C7_1	-2.1(17)
C12_1	C7_1	C8_1	C9_1	-2.9(16)
C13_1	C3_1	C4_1	C5_1	-81.5(8)
C13_1	C3_1	C4_1	C6_1	-170.4(7)
C13_1	C14_1	O1_1	C15_1	174.9(7)
C13_1	C17_1	C18_1	C19_1	177.5(10)
C13_1	C17_1	C18_1	Cl4_1	-3.2(14)
C13_1	C17_1	C22_1	C21_1	-178.4(11)
C14_1	C13_1	C17_1	C18_1	122.4(10)
C14_1	C13_1	C17_1	C22_1	-65.1(10)
C16_1	C15_1	O1_1	C14_1	-104.5(9)
C17_1	C13_1	C14_1	O1_1	-62.2(9)
C17_1	C13_1	C14_1	O2_1	112.4(11)
C17_1	C18_1	C19_1	C20_1	-2.4(18)
C18_1	C17_1	C22_1	C21_1	-5.0(16)
C18_1	C19_1	C20_1	C21_1	-1(2)
C19_1	C20_1	C21_1	C22_1	0(2)
C20_1	C21_1	C22_1	C17_1	2(2)
C22_1	C17_1	C18_1	C19_1	5.0(15)
C22_1	C17_1	C18_1	Cl4_1	-175.6(8)
Cl4_1	C18_1	C19_1	C20_1	178.3(10)
O1_1	C15_1	C16_1	Cl1_1	68.7(10)
O1_1	C15_1	C16_1	Cl2_1	-55.3(10)
O1_1	C15_1	C16_1	Cl3_1	-173.1(7)
O2_1	C14_1	O1_1	C15_1	0.3(14)

Atom	Atom	Atom	Atom	Angle/°
Br1_3	C10_3	C11_3	C12_3	-179.95(14)
C1_3	C2_3	C3_3	C4_3	-0.6(7)
C1_3	C2_3	C3_3	C13_3	130.5(7)
C1_3	C7_3	C8_3	C9_3	178.4(4)
C1_3	C7_3	C12_3	C11_3	-178.5(4)
C2_3	C1_3	C5_3	C4_3	66.0(6)
C2_3	C1_3	C6_3	C4_3	-67.3(6)
C2_3	C1_3	C7_3	C8_3	-106.7(7)
C2_3	C1_3	C7_3	C12_3	72.3(7)
C2_3	C3_3	C4_3	C5_3	44.7(7)
C2_3	C3_3	C4_3	C6_3	-44.1(7)
C2_3	C3_3	C13_3	C14_3	169.2(7)
C2_3	C3_3	C13_3	C17_3	39.5(9)
C3_3	C4_3	C5_3	C1_3	-67.2(6)
C3_3	C4_3	C6_3	C1_3	67.7(6)
C3_3	C13_3	C14_3	O1_3	147.6(8)
C3_3	C13_3	C14_3	O2_3	-15.3(14)
C3_3	C13_3	C17_3	C18_3	-101.9(16)
C3_3	C13_3	C17_3	C22_3	84.1(15)
C4_3	C3_3	C13_3	C14_3	-70.0(10)
C4_3	C3_3	C13_3	C17_3	160.3(7)
C5_3	C1_3	C2_3	C3_3	-43.7(7)
C5_3	C1_3	C6_3	C4_3	33.6(5)
C5_3	C1_3	C7_3	C8_3	130.0(7)
C5_3	C1_3	C7_3	C12_3	-51.0(8)
C5_3	C4_3	C6_3	C1_3	-33.5(5)
C6_3	C1_3	C2_3	C3_3	45.5(7)
C6_3	C1_3	C5_3	C4_3	-33.9(5)
C6_3	C1_3	C7_3	C8_3	16.7(8)
C6_3	C1_3	C7_3	C12_3	-164.2(7)
C6_3	C4_3	C5_3	C1_3	33.6(6)
C7_3	C1_3	C2_3	C3_3	-178.4(7)
C7_3	C1_3	C5_3	C4_3	-165.2(6)
C7_3	C1_3	C6_3	C4_3	163.5(6)
C7_3	C8_3	C9_3	C10_3	0.4(2)
C8_3	C7_3	C12_3	C11_3	0.6(3)
C8_3	C9_3	C10_3	Br1_3	179.95(14)
C8_3	C9_3	C10_3	C11_3	-0.09(11)
C9_3	C10_3	C11_3	C12_3	0.09(11)
C10_3	C11_3	C12_3	C7_3	-0.4(2)
C12_3	C7_3	C8_3	C9_3	-0.6(3)
C13_3	C3_3	C4_3	C5_3	-82.8(8)
C13_3	C3_3	C4_3	C6_3	-171.7(7)
C13_3	C14_3	O1_3	C15_3	170.6(9)
C13_3	C17_3	C18_3	C19_3	-176.9(15)
C13_3	C17_3	C18_3	Cl4_3	8(2)
C13_3	C17_3	C22_3	C21_3	179.2(19)
C14_3	C13_3	C17_3	C18_3	126.7(14)
C14_3	C13_3	C17_3	C22_3	-47.3(16)
C16_3	C15_3	O1_3	C14_3	-139.1(10)
C17_3	C13_3	C14_3	O1_3	-77.6(10)
C17_3	C13_3	C14_3	O2_3	119.5(12)
C17_3	C18_3	C19_3	C20_3	0(3)
C18_3	C17_3	C22_3	C21_3	5(3)
C18_3	C19_3	C20_3	C21_3	1(3)
C19_3	C20_3	C21_3	C22_3	0(4)
C20_3	C21_3	C22_3	C17_3	-3(4)
C22_3	C17_3	C18_3	C19_3	-3(3)

Atom	Atom	Atom	Atom	Angle/°
C22_3	C17_3	C18_3	Cl4_3	-178.4(16)
Cl4_3	C18_3	C19_3	C20_3	175.8(18)
O1_3	C15_3	C16_3	Cl1_3	59.3(11)
O1_3	C15_3	C16_3	Cl2_3	-64.5(11)
O1_3	C15_3	C16_3	Cl3_3	177.8(9)
O2_3	C14_3	O1_3	C15_3	-26.7(16)
Br1_2	C10_2	C11_2	C12_2	179.6(2)
C1_2	C2_2	C3_2	C4_2	-0.6(7)
C1_2	C2_2	C3_2	C13_2	130.5(7)
C1_2	C7_2	C8_2	C9_2	-178.6(4)
C1_2	C7_2	C12_2	C11_2	178.7(3)
C2_2	C1_2	C5_2	C4_2	66.0(6)
C2_2	C1_2	C6_2	C4_2	-67.3(6)
C2_2	C1_2	C7_2	C8_2	-90.5(8)
C2_2	C1_2	C7_2	C12_2	90.9(7)
C2_2	C3_2	C4_2	C5_2	44.7(7)
C2_2	C3_2	C4_2	C6_2	-44.1(7)
C2_2	C3_2	C13_2	C14_2	-168.9(7)
C2_2	C3_2	C13_2	C17_2	50.7(10)
C3_2	C4_2	C5_2	C1_2	-67.2(6)
C3_2	C4_2	C6_2	C1_2	67.7(6)
C3_2	C13_2	C14_2	O1_2	153.1(8)
C3_2	C13_2	C14_2	O2_2	-40.6(15)
C3_2	C13_2	C17_2	C18_2	-96.9(11)
C3_2	C13_2	C17_2	C22_2	76.1(11)
C4_2	C3_2	C13_2	C14_2	-48.1(10)
C4_2	C3_2	C13_2	C17_2	171.5(8)
C5_2	C1_2	C2_2	C3_2	-43.7(7)
C5_2	C1_2	C6_2	C4_2	33.6(5)
C5_2	C1_2	C7_2	C8_2	146.2(7)
C5_2	C1_2	C7_2	C12_2	-32.3(8)
C5_2	C4_2	C6_2	C1_2	-33.5(5)
C6_2	C1_2	C2_2	C3_2	45.5(7)
C6_2	C1_2	C5_2	C4_2	-33.9(5)
C6_2	C1_2	C7_2	C8_2	32.9(8)
C6_2	C1_2	C7_2	C12_2	-145.6(8)
C6_2	C4_2	C5_2	C1_2	33.6(6)
C7_2	C1_2	C2_2	C3_2	-178.4(7)
C7_2	C1_2	C5_2	C4_2	-165.2(6)
C7_2	C1_2	C6_2	C4_2	163.5(6)
C7_2	C8_2	C9_2	C10_2	-0.3(3)
C8_2	C7_2	C12_2	C11_2	0.03(11)
C8_2	C9_2	C10_2	Br1_2	-179.5(3)
C8_2	C9_2	C10_2	C11_2	0.5(3)
C9_2	C10_2	C11_2	C12_2	-0.4(2)
C10_2	C11_2	C12_2	C7_2	0.15(10)
C12_2	C7_2	C8_2	C9_2	0.0(3)
C13_2	C3_2	C4_2	C5_2	-82.8(8)
C13_2	C3_2	C4_2	C6_2	-171.7(7)
C13_2	C14_2	O1_2	C15_2	169.7(8)
C13_2	C17_2	C18_2	C19_2	172.9(10)
C13_2	C17_2	C18_2	Cl4_2	-4.1(16)
C13_2	C17_2	C22_2	C21_2	-173.9(9)
C14_2	C13_2	C17_2	C18_2	120.9(11)
C14_2	C13_2	C17_2	C22_2	-66.1(11)
C16_2	C15_2	O1_2	C14_2	-123.6(11)
C17_2	C13_2	C14_2	O1_2	-63.0(11)
C17_2	C13_2	C14_2	O2_2	103.3(15)

Atom	Atom	Atom	Atom	Angle/°
C17_2	C18_2	C19_2	C20_2	0.1(8)
C18_2	C17_2	C22_2	C21_2	0.0(4)
C18_2	C19_2	C20_2	C21_2	-0.1(10)
C19_2	C20_2	C21_2	C22_2	0.1(10)
C20_2	C21_2	C22_2	C17_2	0.0(8)
C22_2	C17_2	C18_2	C19_2	0.0(4)
C22_2	C17_2	C18_2	Cl4_2	-177.1(14)
Cl4_2	C18_2	C19_2	C20_2	177.2(14)
O1_2	C15_2	C16_2	Cl1_2	57.8(11)
O1_2	C15_2	C16_2	Cl2_2	-66.3(11)
O1_2	C15_2	C16_2	Cl3_2	176.0(8)
O2_2	C14_2	O1_2	C15_2	3.2(17)

Table 12: Hydrogen Fractional Atomic Coordinates (×10[4]) and Equivalent Isotropic Displacement Parameters (Å[2]×10[3]) for zc-02-217a. U_{eq} is defined as 1/3 of the trace of the orthogonalised U_{ij}.

Atom	x	y	z	U_{eq}
H2A_1	8621.43	10978.25	7495.36	34
H2B_1	11627.89	10888.98	7763.2	34
H3_1	10282.64	8783.31	7930.1	34
H4_1	8019.9	8678.77	9014.11	34
H5A_1	5965.41	10532.43	8390.06	38
H5B_1	7155.54	11013.7	9366.26	38
H6A_1	11402.08	10909.58	9721.15	37
H6B_1	12667.89	10361.4	8948.71	37
H8_1	14361.48	13114.86	9342.34	41
H9_1	15691.81	15556.09	9559.28	39
H11_1	8568.09	15529.19	8612.84	41
H12_1	7299.5	13089.74	8416.86	39
H13_1	5371.31	8970.55	7502.17	39
H15B_1	496.88	5062.55	7332.44	53
H15A_1	3397.73	4916.43	7653.77	53
H19_1	5127.26	9520.29	5136.71	47
H20_1	7550.27	8158.93	4646.29	51
H21_1	9592.85	6991.2	5427.51	53
H22_1	9205.8	7200.4	6696.73	46
H2A_3	35.1	-929.61	2496.52	36
H2B_3	2803.92	-965.83	2218.21	36
H3_3	3444.49	1134.92	2036.33	39
H4_3	283.5	1354.28	969.47	36
H5A_3	-2980.93	-383.29	1609.31	37
H5B_3	-3246.19	-892.13	631.63	37
H6A_3	660.48	-960.58	260.23	37
H6B_3	3199.38	-484.28	1024.09	37
H8_3	1391.93	-3267.01	508.57	34
H9_3	88.28	-5702.72	333.01	36
H11_3	-5203.72	-5337.29	1617.74	36
H12_3	-3849.57	-2907.49	1780.67	34
H13_3	-1091.59	1372.06	2440.37	43
H15A_3	491.78	5550.91	2379.45	42
H15B_3	3220.1	5503.7	2908.42	42
H19_3	-643.74	421.38	4751.08	43
H20_3	3510.68	1543.39	5364.15	41
H21_3	6357.64	2687.39	4702.12	44
H22_3	4990.27	2751.05	3436.83	46
H2A_2	35.1	-929.61	2496.52	36

Atom	x	y	z	U_{eq}
H2B_2	2803.92	-965.83	2218.21	36
H3_2	3444.49	1134.92	2036.33	39
H4_2	283.5	1354.28	969.47	36
H5A_2	-2980.93	-383.29	1609.31	37
H5B_2	-3246.19	-892.13	631.63	37
H6A_2	660.48	-960.58	260.23	37
H6B_2	3199.38	-484.28	1024.09	37
H8_2	2041.36	-3212.2	823.81	34
H9_2	647.57	-5654.75	580.15	36
H11_2	-5932.02	-5398.98	1228.49	36
H12_2	-4475.45	-2957.51	1463.99	34
H13_2	-1040.96	1127.61	2519.3	43
H15A_2	-2198.95	4945.49	2462.18	42
H15B_2	933.53	5262.88	2499.47	42
H19_2	801.13	563.28	4859.25	43
H20_2	4988.34	1858.58	5301.9	41
H21_2	7169.33	3062.42	4506.25	44
H22_2	5145.19	2967.04	3275.3	46

Table 13: Atomic Occupancies for all atoms that are not fully occupied in zc-02-217a.

Atom	Occupancy
Br1_3	0.539(4)
C1_3	0.539(4)
C2_3	0.539(4)
H2A_3	0.539(4)
H2B_3	0.539(4)
C3_3	0.539(4)
H3_3	0.539(4)
C4_3	0.539(4)
H4_3	0.539(4)
C5_3	0.539(4)
H5A_3	0.539(4)
H5B_3	0.539(4)
C6_3	0.539(4)
H6A_3	0.539(4)
H6B_3	0.539(4)
C7_3	0.539(4)
C8_3	0.539(4)
H8_3	0.539(4)
C9_3	0.539(4)
H9_3	0.539(4)
C10_3	0.539(4)
C11_3	0.539(4)
H11_3	0.539(4)
C12_3	0.539(4)
H12_3	0.539(4)
C13_3	0.539(4)
H13_3	0.539(4)
C14_3	0.539(4)
C15_3	0.539(4)
H15A_3	0.539(4)
H15B_3	0.539(4)
C16_3	0.539(4)
C17_3	0.539(4)
C18_3	0.539(4)

Atom	Occupancy
C19_3	0.539(4)
H19_3	0.539(4)
C20_3	0.539(4)
H20_3	0.539(4)
C21_3	0.539(4)
H21_3	0.539(4)
C22_3	0.539(4)
H22_3	0.539(4)
Cl1_3	0.539(4)
Cl2_3	0.539(4)
Cl3_3	0.539(4)
Cl4_3	0.539(4)
O1_3	0.539(4)
O2_3	0.539(4)
Br1_2	0.461(4)
C1_2	0.461(4)
C2_2	0.461(4)
H2A_2	0.461(4)
H2B_2	0.461(4)
C3_2	0.461(4)
H3_2	0.461(4)
C4_2	0.461(4)
H4_2	0.461(4)
C5_2	0.461(4)
H5A_2	0.461(4)
H5B_2	0.461(4)
C6_2	0.461(4)
H6A_2	0.461(4)
H6B_2	0.461(4)
C7_2	0.461(4)
C8_2	0.461(4)
H8_2	0.461(4)
C9_2	0.461(4)
H9_2	0.461(4)
C10_2	0.461(4)
C11_2	0.461(4)
H11_2	0.461(4)
C12_2	0.461(4)
H12_2	0.461(4)
C13_2	0.461(4)
H13_2	0.461(4)
C14_2	0.461(4)
C15_2	0.461(4)
H15A_2	0.461(4)
H15B_2	0.461(4)
C16_2	0.461(4)
C17_2	0.461(4)
C18_2	0.461(4)
C19_2	0.461(4)
H19_2	0.461(4)
C20_2	0.461(4)
H20_2	0.461(4)
C21_2	0.461(4)

Atom	Occupancy
H21_2	0.461(4)
C22_2	0.461(4)
H22_2	0.461(4)
Cl1_2	0.461(4)
Cl2_2	0.461(4)
Cl3_2	0.461(4)
Cl4_2	0.461(4)
O1_2	0.461(4)
O2_2	0.461(4)

Citations

CrysAlisPro (ROD), Rigaku Oxford Diffraction, Poland (?).

CrysAlisPro Software System, Rigaku Oxford Diffraction, (2024).

O.V. Dolomanov and L.J. Bourhis and R.J. Gildea and J.A.K. Howard and H. Puschmann, Olex2: A complete structure solution, refinement and analysis program, *J. Appl. Cryst.*, (2009), **42**, 339-341.

Sheldrick, G.M., Crystal structure refinement with ShelXL, *Acta Cryst.*, (2015), **C71**, 3-8.

Sheldrick, G.M., ShelXT-Integrated space-group and crystal-structure determination, *Acta Cryst.*, (2015), **A71**, 3-8.

9. References

(1) Ji, S.; Li, X.; Wang, Y.; Zhang, D.; Lv, J.; Shi, Y.; Yang, D. Triphenylamine-Mediated Intramolecular Remote Cyano Migration via Electron Donor–Acceptor Complex Photoactivation. *Org. Lett.* **2025**, *27*, 7892-7897.
(2) Li, S.; Zhang, C.; Wang, S.; Yang, W.; Fang, X.; Fan, S.; Zhang, Q.; Li, X.-X.; Feng, Y.-S. Cooperative Photoredox and N-Heterocyclic Carbene Catalysis Suzuki–Miyaura-Type Reaction: Radical Coupling of Aroyl Fluorides and Alkyl Boronic Acids. *Org. Lett.* **2024**, *26*, 1728-1733.
(3) Scaringi, S.; Mazet, C. Kinetically Controlled Stereoselective Access to Branched 1,3-Dienes by Ru-Catalyzed Remote Conjugative Isomerization. *ACS Catal.* **2021**, *11*, 7970-7977.
(4) Kleinmans, R.; Apolinar, O.; Derosa, J.; Karunananda, M. K.; Li, Z.-Q.; Tran, V. T.; Wisniewski, S. R.; Engle, K. M. Ni-Catalyzed 1,2-Diarylation of Alkenyl Ketones: A Comparative Study of Carbonyl-Directed Reaction Systems. *Org. Lett.* **2021**, *23*, 5311-5316.
(5) Zou, S.; Gao, B.; Huang, Y.; Zhang, T.; Huang, H. Palladium-Catalyzed Hydrocarbonylative Cyclization of 1,5-Dienes. *Org. Lett.* **2019**, *21*, 6333-6336.
(6) Wu, J.; Yang, X.; He, Z.; Mao, X.; Hatton, T. A.; Jamison, T. F. Continuous Flow Synthesis of Ketones from Carbon Dioxide and Organolithium or Grignard Reagents. *Angew. Chem. Int. Ed.* **2014**, *53*, 8416-8420.
(7) Levterov, V. V.; Panasiuk, Y.; Shablykin, O.; Stashkevych, O.; Sahun, K.; Rassokhin, A.; Sadkova, I.; Lesyk, D.; Anisiforova, A.; Holota, Y.; Borysko, P.; Bodenchuk, I.; Voloshchuk, N. M.; Mykhailiuk, P. K. 2-Oxabicyclo[2.1.1]hexanes: Synthesis, Properties, and Validation as Bioisosteres of ortho- and meta-Benzenes. *Angew. Chem. Int. Ed.* **2024**, *63*, e202319831.
(8) Frisch, M. J.; Trucks, G. W.; Schlegel, H. B.; Scuseria, G. E.; Robb, M. A.; Cheeseman, J. R.; Scalmani, G.; Barone, V.; Petersson, G. A.; Nakatsuji, H.; Li, X.; Caricato, M.; Marenich, A. V.; Bloino, J.; Janesko, B. G.; Gomperts, R.; Mennucci, B.; Hratchian, H. P.; Ortiz, J. V.; Izmaylov, A. F.; Sonnenberg, J. L.; Williams; Ding, F.; Lipparini, F.; Egidi, F.; Goings, J.; Peng, B.; Petrone, A.; Henderson, T.; Ranasinghe, D.; Zakrzewski, V. G.; Gao, J.; Rega, N.; Zheng, G.; Liang, W.; Hada, M.; Ehara, M.; Toyota, K.; Fukuda, R.; Hasegawa, J.; Ishida, M.; Nakajima, T.; Honda, Y.; Kitao, O.; Nakai, H.; Vreven, T.; Throssell, K.; Montgomery Jr., J. A.; Peralta, J. E.; Ogliaro, F.; Bearpark, M. J.; Heyd, J. J.; Brothers, E. N.; Kudin, K. N.; Staroverov, V. N.; Keith, T. A.; Kobayashi, R.; Normand, J.; Raghavachari, K.; Rendell, A. P.; Burant, J. C.; Iyengar, S. S.; Tomasi, J.; Cossi, M.; Millam, J. M.; Klene, M.; Adamo, C.; Cammi, R.; Ochterski, J. W.; Martin, R. L.; Morokuma, K.; Farkas, O.; Foresman, J. B.; Fox, D. J. Gaussian 16 Rev. C.01. **2016**.
(9) Humphrey, W.; Dalke, A.; Schulten, K. VMD: Visual molecular dynamics. *J. Mol. Graph.* **1996**, *14*, 33-38.
(10) Momma, K.; Izumi, F. VESTA: a three-dimensional visualization system for electronic and structural analysis. *J. Appl. Crystallogr.* **2008**, *41*, 653-658.
(11) Dapprich, S.; Komáromi, I.; Byun, K. S.; Morokuma, K.; Frisch, M. J. A new ONIOM implementation in Gaussian98. Part I. The calculation of energies, gradients, vibrational frequencies and electric field derivatives1Dedicated to Professor Keiji Morokuma in celebration of his 65th birthday.1. *J. Mol. Struct. THEOCHEM* **1999**, *461-462*, 1-21.
(12) Vreven, T.; Byun, K. S.; Komáromi, I.; Dapprich, S.; Montgomery, J. A., Jr.; Morokuma, K.; Frisch, M. J. Combining Quantum Mechanics Methods with Molecular Mechanics Methods in ONIOM. *JCTC* **2006**, *2*, 815-826.
(13) Zhao, Y.; Truhlar, D. G. The M06 suite of density functionals for main group thermochemistry, thermochemical kinetics, noncovalent interactions, excited states, and transition elements: two new functionals and systematic testing of four M06-class functionals and 12 other functionals. *Theor. Chem. Acc.* **2008**, *120*, 215-241.
(14) Hay, P. J.; Wadt, W. R. Ab initio effective core potentials for molecular calculations. Potentials for K to Au including the outermost core orbitals. *J. Chem. Phys.* **1985**, *82*, 299-310.

(15) Roy, L. E.; Hay, P. J.; Martin, R. L. Revised Basis Sets for the LANL Effective Core Potentials. *J. Chem. Theory Comput.* **2008**, *4*, 1029-1031.
(16) Hariharan, P. C.; Pople, J. A. The influence of polarization functions on molecular orbital hydrogenation energies. *Theoretica chimica acta* **1973**, *28*, 213-222.
(17) Hehre, W. J.; Ditchfield, R.; Pople, J. A. Self—Consistent Molecular Orbital Methods. XII. Further Extensions of Gaussian—Type Basis Sets for Use in Molecular Orbital Studies of Organic Molecules. *J. Chem. Phys.* **1972**, *56*, 2257-2261.
(18) Rappe, A. K.; Casewit, C. J.; Colwell, K. S.; Goddard, W. A., III; Skiff, W. M. UFF, a full periodic table force field for molecular mechanics and molecular dynamics simulations. *J. Am. Chem. Soc.* **1992**, *114*, 10024-10035.
(19) Falivene, L.; Cao, Z.; Petta, A.; Serra, L.; Poater, A.; Oliva, R.; Scarano, V.; Cavallo, L. Towards the online computer-aided design of catalytic pockets. *Nat. Chem.* **2019**, *11*, 872-879.
(20) Lu, T.; Chen, F. Multiwfn: A multifunctional wavefunction analyzer. *J. Comput. Chem.* **2012**, *33*, 580-592.
(21) Mitoraj, M. P.; Michalak, A.; Ziegler, T. A Combined Charge and Energy Decomposition Scheme for Bond Analysis. *J. Chem. Theory Comput.* **2009**, *5*, 962-975.
(22) Lu, T.; Chen, Q. Simple, Efficient, and Universal Energy Decomposition Analysis Method Based on Dispersion-Corrected Density Functional Theory. *J. Phys. Chem. A* **2023**, *127*, 7023-7035.
(23) Bickelhaupt, F. M.; Houk, K. N. Analyzing Reaction Rates with the Distortion/Interaction-Activation Strain Model. *Angew. Chem. Int. Ed.* **2017**, *56*, 10070-10086.
(24) Brunard, E.; Boquet, V.; Saget, T.; Sosa Carrizo, E. D.; Sircoglou, M.; Dauban, P. Catalyst-Controlled Intermolecular Homobenzylic C(sp3)–H Amination for the Synthesis of β-Arylethylamines. *J. Am. Chem. Soc.* **2024**, *146*, 5843-5854.
(25) Berry, J. F. The role of three-center/four-electron bonds in superelectrophilic dirhodium carbene and nitrene catalytic intermediates. *Dalton Trans.* **2012**, *41*, 700-713.
